# Global Analysis of Proline-Rich Tandem Repeat Proteins Reveals Broad Phylogenetic Diversity in Plant Secretomes

**DOI:** 10.1371/journal.pone.0023167

**Published:** 2011-08-02

**Authors:** Aaron M. Newman, James B. Cooper

**Affiliations:** 1 Biomolecular Science and Engineering Program, University of California Santa Barbara, Santa Barbara, California, United States of America; 2 Department of Molecular, Cellular, and Developmental Biology, University of California, Santa Barbara, California, United States of America; The Centre for Research and Technology, Hellas, Greece

## Abstract

Cell walls, constructed by precisely choreographed changes in the plant secretome, play critical roles in plant cell physiology and development. Along with structural polysaccharides, secreted proline-rich Tandem Repeat Proteins (TRPs) are important for cell wall function, yet the evolutionary diversity of these structural TRPs remains virtually unexplored. Using a systems-level computational approach to analyze taxonomically diverse plant sequence data, we identified 31 distinct Pro-rich TRP classes targeted for secretion. This analysis expands upon the known phylogenetic diversity of extensins, the most widely studied class of wall structural proteins, and demonstrates that extensins evolved before plant vascularization. Our results also show that most Pro-rich TRP classes have unexpectedly restricted evolutionary distributions, revealing considerable differences in plant secretome signatures that define unexplored diversity.

## Introduction

Composed primarily of polysaccharides, plant cell walls provide critical structural support for terrestrial plant life, and play important roles in plant growth, development, and interactions with microbes. In addition, ligno-cellulosic plant cell walls provide the dietary fiber that facilitates mammalian digestion, represent an important source of textile materials and combustible biofuels, and play a major role in the global carbon cycle. Biochemical studies indicate that wall polysaccharide composition differs among plant lineages, and two distinct wall types have been characterized in higher plants to date [1], [2]. Gymnosperms and most angiosperms are known to have Type I cell walls that have a distinctly different polysaccharide composition from Type II cell walls found only in a taxonomic group of advanced monocots that includes the grasses [3]. Generally missing from current systems-level views of the plant cell wall [1], [4], [5], however, are the secreted structural (glyco)protein elements containing 4-trans-hydroxyproline (Hyp) first discovered more than fifty years ago [6], [7].

Nearly ubiquitous in green plants, Hyp-rich glycoproteins, commonly known as HRGPs or extensins [8], [9], compose up to 10% of the cell wall mass of higher plants [6], [7], and have been shown to play critical roles in cell wall structure and function [10]–[13]. Post-translationally modified from Pro-rich polypeptides, secreted HRGPs are generally grouped into three broad classes based on primary sequence architectures and glycosylation profiles [9], [14]–[16], and these three classes, the extensin glycoproteins, the proline-rich proteins (PRPs), and the highly glycosylated arabinogalactan proteins (AGPs), have been hypothesized to form a phylogenetic continuum ranging from green algae throughout land plants [9], [17].

Known HRGPs have highly biased amino acid compositions, and like numerous structural proteins found throughout nature, extensins and PRPs also have highly repetitive, tandem repeat (TR), sequence architectures. These sequence characteristics, along with extensive post-translational modifications often leading to insoluble cross-linked HRGP networks, have hindered broad phylogenetic analysis of plant HRGPs. Since commonly applied sequence analysis methods, such as BLAST [18] or Hidden Markov Models [19], have considerable limitations for analyzing sequences with biased amino acid content [15], several groups have used simple compositional filters or regular expression queries (based on previously characterized sequence motifs) to identify Pro-rich proteins from plant sequence databases. For example, Schultz et al. (2002) [20] and Ma and Zhao (2010) [21] identified AGPs in *Arabidopsis* and rice, respectively, using a biased amino acid filter (50–55% or 35% Pro/Ala/Ser/Thr composition depending on protein length), and Graham et al. (2004) [22] used the pattern, PPV(E/Y/V)K, to identify novel PRPs in legumes. Showalter et al. (2010) [15] developed BIO OHIO, a software tool that implements a variety of compositional and regular expression filters, to identify previously defined HRGP groups in the *Arabidopsis* genome. Unfortunately, these approaches all rely upon prior knowledge of repeat patterns and/or composition, limiting their utility for analyzing novel Pro-rich TRPs on a global multi-genomic scale.

In this study, we leveraged computational methods specifically developed to analyze large sequence databases for TR and TRP content without prior knowledge, and report a considerable expansion in our knowledge of the number and diversity of Pro-rich TRP sequence classes targeted to plant secretomes. In contrast to previous analyses that have been limited to a handful of higher plant species (e.g., [15], [22]), we analyzed millions of plant secretome sequences spanning a broad phylogenetic range. Based on the inherent differences in primary sequence architecture among secreted Pro-rich TRPs, we propose a new taxonomy and nomenclature for 31 distinct classes of secreted Pro-rich TRPs. In addition to furthering our understanding of the phylogenetic distributions of canonical HRGP TRPs, our data indicate that nearly half of the identified TRP classes have very narrow phylogenetic distributions. Such diverse Pro-rich structural TRPs reveal phylogenetic distinctions that define an abundance of previously unrecognized secretome signatures.

## Results and Discussion

### Identification and classification of Pro-Rich TRPs in plant secretomes

**Figure 1:**
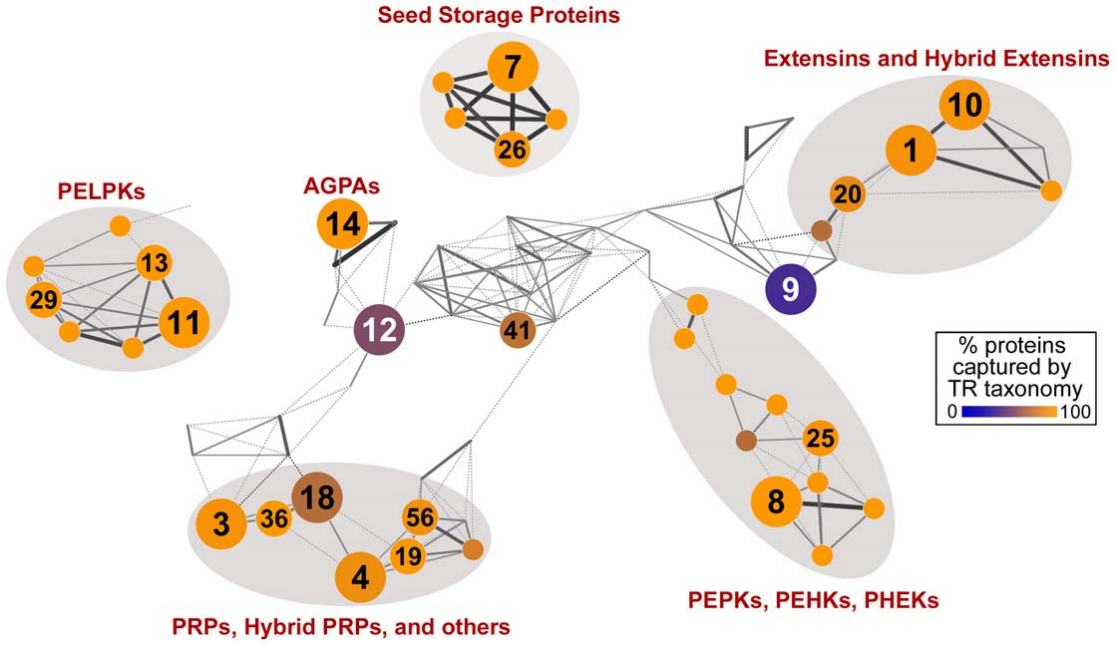
Cluster landscape of Pro-rich TR motifs from plant secretome sequences. Each node represents a TR cluster, node labels denote the original cluster identifier (see Tables S9, S10), and edge thickness represents the fraction of times each pair of TR clusters was co-clustered over ensemble re-sampling (see *Materials and Methods*) (also see “pairwise affinity” defined in Figure 4 of [49]). Thin, dotted, edges indicate a co-clustering of <10%. Large labeled nodes in the network denote clusters containing secreted TRPs found in at least ten species and twenty protein sequences (Table S10) while intermediate size labeled nodes satisfy one of these two criteria. Smaller unlabeled nodes do not meet either criterion, but are shown due to their similarity in motif content to larger, neighboring nodes. Major TRP classes from Tables S6, S7 and S8 are indicated around corresponding TR motif super-classes (circled in gray). Node color represents the retention rate of the TR taxonomy (Tables S3, S4, S5), defined as the proportion of all protein sequences corresponding to each cluster that are captured by TR class definitions (for class definitions, see Table S11; for a quantitative version of the taxonomy retention rate, see Table S10). For a high-resolution version of this network showing all individual TR consensus sequences, see Figure S1, and see Figure S2 for the same high-resolution network also showing TR super-classes. For details of network construction, see *Materials and Methods*. This network was rendered using Cytoscape 2.6.0 [50].

To explore the global diversity of Pro-rich TRPs, we constructed a database containing 8.3 M full and partial protein sequences, from genome and EST projects, spanning 36,815 plant and green algal species (Table S1; note that to expand sequence diversity, most of data analyzed in this work are plant EST sequences). These data were analyzed for TR content using XTREAM (Newman and Cooper, 2007), and ∼210,000 Pro-rich TR motifs were identified, of which ∼90,000 are unique (Table S2, footnoted). All TR-containing sequences with an N-terminal Met were analyzed for the presence of a secretory signal peptide using SignalP 3.0 [23] (Table S2). From these sequences, unique TR motifs that compose protein domains of at least 100 amino acids, or 50 amino acids and at least 1/3 of the protein length (Table S2), were clustered without prior knowledge into TR classes based on sequence similarity. Though necessarily arbitrary, these TR length criteria ensured that all analyzed TRs are sufficiently long to compose significant structural domains in their corresponding proteins. The results of this cluster analysis are presented in Figure 1 as a network diagram depicting the landscape of the most abundant Pro-rich TRs in plant secretomes, where nodes correspond to TR motif clusters and edges reflect sequence similarity. A high-resolution view of this network, together with the individual TR motif consensus sequences, is shown in Figure S1. Using this network, we identified 38 distinct TR motif classes (Table S3, Table S4, and Table S5) that define 31 unique classes of secreted Pro-rich TRPs (Table S6, Table S7, and Table S8; also see Text S1, Text S2, and Text S3). Most of these TRP classes map to one of five large TR motif super-classes (Figures 1 and S2), and are distinctly different in TR sequence architecture from previously recognized HRGP groups [14].

To illustrate the sequence architectures of these TRP classes, representative examples of aligned TR domains are shown in Dataset S1, and multiple sequence alignments of the N-terminal and C-terminal regions of selected TRP classes are shown in Dataset S2 and Figure S3. Aside from two heterogeneous classes (called EXTM and SPAP), all of the TRP classes we identified are highly conserved in sequence both within and outside of the Pro-rich TR domain, generally including the predicted N-terminal signal peptide (Dataset S2 and Figure S3). Complete data outputs of the TR motif cluster analysis are available in the supporting information online (Table S9 and Table S10), and all identified TRP sequences, including the TR motifs corresponding to each of the 38 TR classes, are available via a web database called PlantPro20Fam (http://jimcooperlab.mcdb.ucsb.edu/plantpro20fam).

### Extensins: “extensively” distributed in the embryophytes

**Figure 2:**
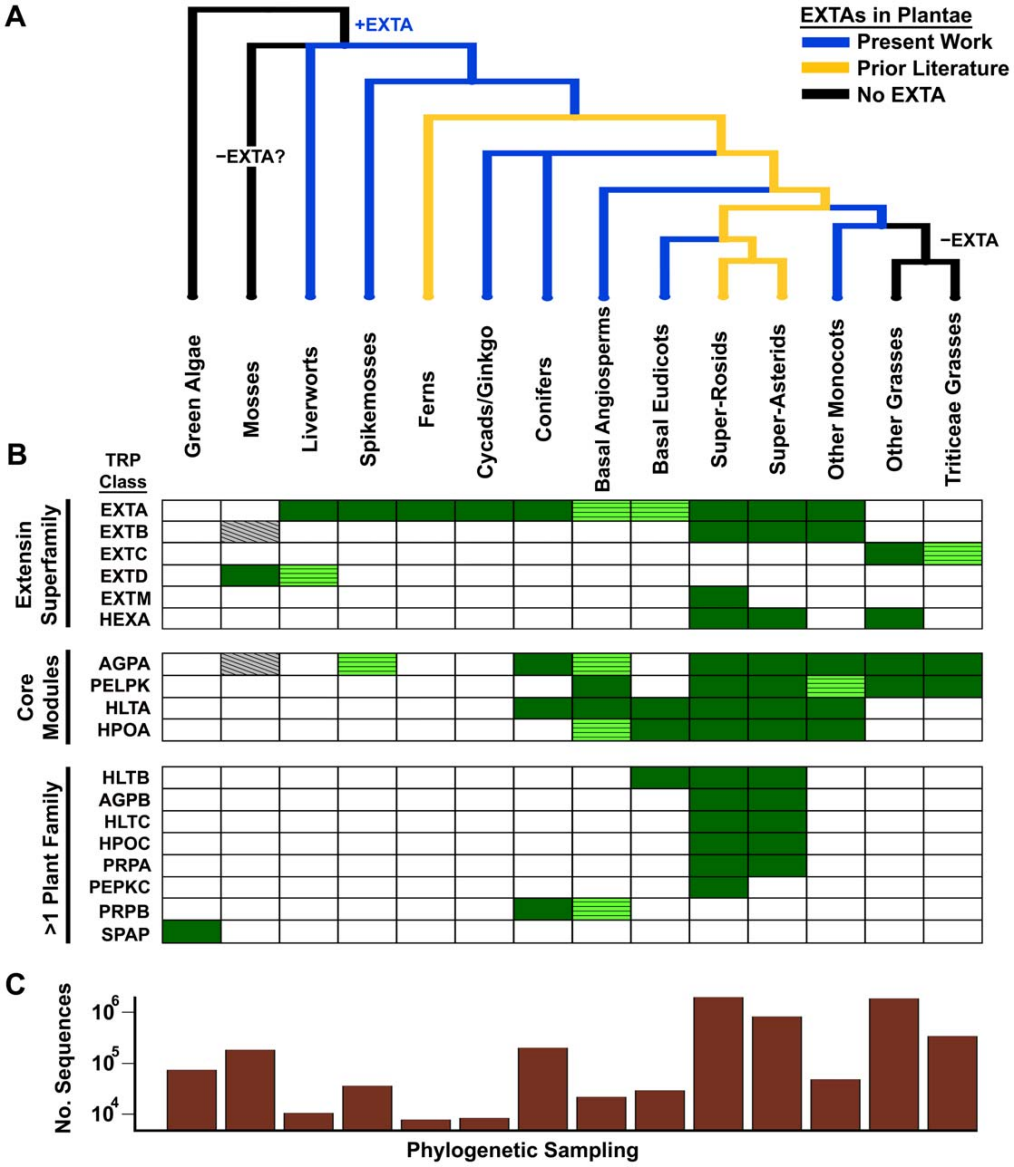
Phylogenetic distribution of Pro-rich TRP classes targeted to plant secretomes. (A) Dendrogram showing the evolutionary relationships among major plant divisions as well as the distribution of EXTAs identified in this study and in previous literature [14], [25], [26]. (To reflect results of a recent phylogenetic analysis [52], the large divisions, super-rosids and super-asterids are shown rather than rosids, asterids, and other phylogenetic groups). (B) Heat map showing phylogenetic distributions of 18 secreted Pro-rich TRP classes, 17 of which are represented by more than a single plant family (abbreviated names are described in Text S2, and Tables S6, S7, S8). TRP classes are divided into the *extensin superfamily*, the non-extensin *core modules*, and the less conserved TRP classes found in *>1 plant family*. Dark-green rectangles represent TRP classes in which at least one known protein sequence or full-length ORF with a predicted secretion signal was found in the corresponding TRP class and plant phylogenetic group. Light-green rectangles with a horizontal line pattern represent TRP classes in which at least one *putative* member is present in the corresponding phylogenetic group (e.g. lacking a full-length sequence). Gray rectangles with a diagonal line pattern represent the *putative* moss AGPA and EXTB sequences (the AGPA is not predicted to be GPI-anchored; the EXTB sequence is not predicted to be secreted and has a TR domain that encompasses only half of the sequence). (C) Phylogenetic sampling bias of all ESTs and protein sequences (from Table S1) either captured by our TR taxonomy (Tables S3, S4, S5) or representing secreted Pro-rich TRPs (Table S2), shown as a log-scale histogram.

**Figure 3:**
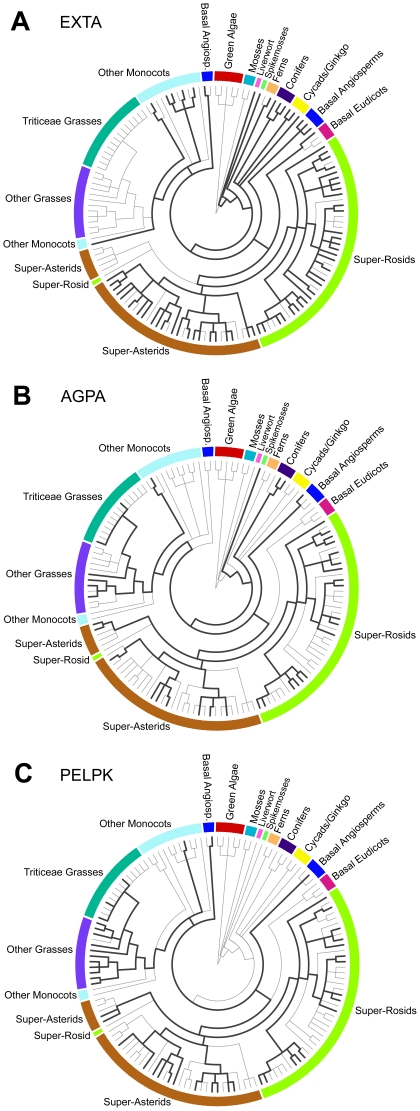
Phylogenetic distributions of three core module plant Pro-rich TRP classes. (A–C) Circular trees show genera distributions, with major plant divisions (same as Figure 2A) labeled around each tree. Versions of trees showing all genera are illustrated in the Figures S4 (EXTA), S7 (AGPA), and S8 (PELPK). The 143 leaves in these trees represent the genera that are either captured by our TR taxonomy (Tables S3, S4, S5) or represented by at least one secreted Pro-rich TRP in our analysis (Table S2). Taxonomic data were downloaded from NCBI, and trees were rendered using PhyloWidget [53].

Extensins, the first widely studied class of wall HRGPs, are generally defined as highly basic glycoproteins composed of canonical Ser-Pro_4_ TR motifs, in which nearly every Pro residue is 4-trans-hydroxylated and O-arabinosylated [9], [14], [17]. In addition, extensin TR motifs are known to contain isodityrosine (Idt) crosslinking motifs: either YxY and/or VYK [24], [25]. Previous work found Hyp-Arabinosides to be ubiquitous in green plants [8]. However, due to the difficulty in characterizing full-length extensin glycoproteins, the evolutionary origins and distribution of these wall structural molecules has remained obscure [9], [14], [25]. Our global analysis identified YxY-containing extensins, referred to as Extensin type alpha (EXTA) (Text S1), throughout most land plant divisions (Figures 2A, 3A, and Figure S4). In addition to the core eudicots and ferns [14], [25], [26], EXTAs are found in at least some non-grass monocots, including onion, orchids, and asparagus, and in a diversity of non-flowering vascular plants, ranging from gymnosperms to *Selaginella*, a member of the oldest extant vascular plant group [27]. Furthermore, although missing in mosses, transcripts encoding EXTAs were found in the non-vascular liverwort species *Marchantia polymorpha* demonstrating that EXTAs predate the evolution of plant vasculature (Text S3, also see PlantPro20Fam).

While green algae are known to secrete HRGPs [28], we found no evidence for EXTAs in Chlorophytes. Rather, we identified a wide variety of high molecular weight, low-complexity SP_n_-containing proteins with highly heterogeneous architectures generally lacking Tyr (see PlantPro20Fam), including the SPAP_2_ containing mating-type agglutinins of *Chlamydomonas* [28] (Table S6). These algal Pro-rich TRs generally compose limited sequence domains within large multi-domain non-TRPs, consistent with the hypothesis that the extensin class of HRGPs evolved in plants during the colonization of terrestrial ecosystems.

Given the widespread occurrence of EXTAs in land plant secretomes, we next examined EXTA TR domains for evidence of structural conservation. We found that TR periodicities in EXTAs are highly conserved, with periods of 10 and 16 residues occurring in nearly two-thirds of EXTA TRs (Figure S5). In addition, at least one of these two periodicities occurs in every major plant lineage containing an EXTA sequence (Figure 2A). Circular dichroism data previously showed that carrot EXTA forms an extended polyproline II (PPII) helix and appears rod-like when imaged by electron microscopy [29]. Because of the trilateral symmetry of PPII helices, TR periodicity will determine the topological regularity of amino acid side groups along an extended PPII helix [30]. Both 10 mers and 16 mers have periodicities of length *n*+1, where *n* is a multiple of 3. Lacking intramolecular Idt [25], *n*+1 (and *n*−1) TR repeats are predicted to have a spiraling pattern along the PPII helix whereby each repeat is rotated by 120° with respect to its upstream repeat. As shown in Figure S5, ∼92% of EXTA TRs have periodicities of *n*+1 (∼77%) or *n*−1 (∼15%), implying that natural selection has maintained such a genetically encoded structural feature in EXTAs throughout land plant evolution.

In addition to conserved periodicity, most full-length EXTA sequences also have a conserved C-terminal SP_n_ motif with a Tyr residue at or near the C-terminus (Dataset S2A; also see PlantPro20Fam online), as was previously noted for 18 of 20 *Arabidopsis* extensins [12]. Notably, this terminal SP_n_,Y domain may or may not be contiguous with the EXTA TR domain.

As a class, EXTAs have a number of conserved sequence features, including SP_n_,Y-containing TRs with basic isoelectric points (e.g., K-rich), and the general occurrence of an adjacent or non-contiguous C-terminal SP_n_,Y domain. Allowing for the substitution of Thr for Ser, we identified three additional TRP classes containing all of these conserved EXTA sequence features that we term extensin subtypes beta, gamma, and delta (Dataset S2, A–D). Based on EST data (Table S13), each of these four EXT classes is expressed in all major plant organs at all stages of development (including both sporophyte and gametophyte for moss EXTDs). In addition, our analysis revealed a broadly distributed Hybrid EXtensin class (HEX type alpha) that includes the leucine-rich repeat HEXs previously called LRXs and PEXs and studied in *Arabidopsis* and rice [31] (Dataset S2E), as well as a miscellaneous extensin-like class that generally lacks C-terminal SP_n_,Y (EXTM) (Text S2).

Among the additional extensin classes, type beta (EXTB) differs from EXTAs by the general absence of YxY motifs and the presence of the potential crosslinking motif VYK (and variants V/I-Y/H-K/H) (Text S2). Unlike EXTAs, EXTBs include the P1-type extensins, while like EXTAs, EXTBs are typically composed of TRs with periods that are not a multiple of 3 (Figure S6). Although found predominantly in the non-grass angiosperms and absent from grasses (Figure 2B), a putative EXTB was also identified in moss. The completed *Arabidopsis* and *Medicago* genomes each contain a single EXTB protein, compared to eighteen and ten EXTAs, respectively. If a comparably low ratio of EXTB to EXTA proteins is common in other genomes, future genome and deep sequencing projects may reveal a much broader phylogenetic distribution of EXTBs.

Previously called THRGPs, sequences that compose the extensin type gamma (EXTC) class are uniquely found in grass secretomes and have been well characterized in maize [32], [33]. In addition to SP_1_ (and sometimes SP_2_), EXTC TR domains are characterized by TP_2_TY motifs (Table S3) that are highly homologous to hydroxylated SP_n_,Y motifs conserved within the other extensins. Like EXTAs and EXTBs, EXTCs are also basic (K-rich) and have a C-terminal SP_n_,Y domain (Dataset S2C) indicating that these grass TRPs are *bona fide* members of the extensin superfamily.

Although moss sequences in our database lack EXTAs (Figure 2, A and B), analysis of the fully sequenced genome of *Physcomitrella patens*, together with EST data from several moss families, revealed a previously unrecognized extensin class, type delta (EXTD). Defined by the presence of TRs with SP_2_ and SPVYX motifs (where X = K/E/T/S; Table S3), and a C-terminal SP_n_,Y domain (see Dataset S2D), EXTDs share conserved sequence features with other extensins, and like EXTBs, many EXTDs have at least one basic VYK crosslinking site. A partial sequence encoding a homologous EXTD, composed of SPPVYXAPP TRs with a carboxy terminal TP_2_,Y domain, was also found in the liverwort *M. polymorpha* indicating that the distribution of EXTDs may extend to all bryophytes (GenBank accession BJ851426, also see PlantPro20Fam).

### Four conserved classes of non-extensin Pro-Rich TRPs

Like EXTAs, four non-extensin TRP classes were found broadly distributed throughout plant evolution and expressed throughout plant development, presumably representing important structural “core modules” (Figures 2B and 3, Table S13). One of these classes is defined by TRs enriched in a T/S-P_3_A motif that was previously identified in two *Arabidopsis* AGPs (AGP7 and AGP9, [34]). Based on its broad distribution (Figures 3B and S7), we refer to this TRP class as TR-AGP type alpha (AGPA). Though most AGPs are not TRPs [34], most AGPA sequences have a predicted GPI-anchor (like the classical AGPs), including AGPAs present in the major divisions of higher plants: conifers, eudicots, non-grass monocots, and grasses (76% of AGPA sequences in Text S3). A highly conserved glutamine residue found at the N-terminus of mature AGPAs (predicted signal peptide removed) is consistent with the possibility of a common ancestor.

A second TRP core module class, characterized by PELPK-containing repeats (Figures 1 and S2), is distributed throughout grass and non-grass angiosperms (Figures 3C and S8). Multiple sequence alignment analysis of PELPKs revealed conserved non-TR N-terminal and C-terminal sequence features consistent with the hypothesis that the PELPKs are an orthologous TRP class that arose sometime before or during the emergence of basal angiosperms. Although evidence for PELPK proteins was detected in sixty-four flowering plant species and 45 genera (Figures 3C and S8), only two PELPKs have been previously reported (At5g09520 and At5g09530 in *Arabidopsis*, called putative PRPs by [14], PRP9 and PRP10 by [15], and PELPK2 and PELPK1 by [35]). Recently, At5g09530 was shown to localize to seed aleurone cells and xylem cell walls, and found to be expressed in response to pathogen attack and stress [35]. Using publicly available *Arabidopsis* microarray analysis tools [36], [37] we also found that both *Arabidopsis* PELPK genes are up-regulated during seed development and expressed in radicle, hypocotyl, and adult root tissues, and both are up-regulated in procambial tissue in response to osmotic stresses or ABA treatments. Together, these data are consistent with the hypothesis that PELPKs are a large, widely distributed class of plant cell wall proteins with potentially diverse physiological functions.

Also broadly distributed in higher plants, we found two large groups of TRP classes each defined by the fusion of a distinct Pro-rich TR domain to highly conserved Cys-rich domains, either the Lipid Transfer (LT) protein domain or the Pollen Ole e I (PO) domain [38], [39]. PO domains are considered a “domain/protein of unknown function” (termed DUF1210 in the Pfam database), while LT domains have been found in small, secreted proteins known to play roles in plant growth, defense and reproduction [40], [41]. Among six subtypes of hybrid LT TRPs and three subtypes of hybrid PO TRPs identified in our analysis (Table S7), two classes represent core modules in higher plant secretomes, HLT type alpha (HLTA) and HPO type alpha (HPOA). HLTAs have a Pro-rich TR domain containing variations on the motif, PPVTLPPVVK (Table S5), and are found in 46 genera of non-grass seed-bearing plants (Figure S9), while HPOAs have a TR domain containing variations on the motif, PPPVPVYKKPL (Table S5), and are found in 37 genera from primitive to advanced non-grass angiosperms (Figure S10).

### Most Pro-rich TRP classes have narrow phylogenetic distributions

Remarkably, 81% of secreted Pro-rich structural TRP classes that we identified have very limited phylogenetic distributions (Figure 2B, Table 1), with nearly half of the 31 TRP classes restricted to individual plant families (Table 1). For example, the “classical” PRPs, defined by the conserved TR pentapeptide P_2_V(Y/E/H)K, are common throughout the Fabaceae but are limited to only three additional eudicot families in our database (see PRPA in Figure 2B and Text S2). A similar but distinct TRP class (PRPB) is found in conifers (Table S7, Text S2). Two additional TRP classes are specific to legumes, and two other classes are only found in Brassicaceae or *Populus* species (Table 1). Remarkably, the PEHK class, exclusive to the grape family, has 18 known and predicted genes arranged in a sequence continuum across chromosome five in *V. vinifera*. Such a physical arrangement indicates that this gene family is likely a product of recent tandem gene duplication (Figure S3). (Several other Pro-rich TRP genes identified in this work also occur in closely linked gene clusters, including four different extensin gene clusters in *Arabidopsis*, and the PELPK genes in *Arabidopsis*, rice and *Sorghum bicolor* (see Text S2.) Finally, the grass family, known to have a unique Type II cell wall architecture [3], has a strikingly large group of unique Pro-rich TRP classes (Table 1). Importantly, all plant families with a unique TRP class have at least one fully sequenced genome. The apparent absence of taxonomically restricted TRP classes in other analyzed plant families, however, may result from the unavoidable phylogenetic sampling bias of currently available sequence data (e.g., Figure 2C). Likewise, it is possible that some of the Pro-rich TR classes identified in this work span broader phylogenetic ranges than described.

**Table 1:** Plant family-specific Pro-rich TRP classes.

TRP Class	Plant Family	General Division(s)
HLTD	Brassicaceae (e.g. *Arabidopsis*)	Super-Rosids (Eudicots)
PEHK	Vitaceae (grape family)	Super-Rosids (Eudicots)
PHEK	Fabaceae (legumes)	Super-Rosids (Eudicots)
KPIP	Fabaceae	Super-Rosids (Eudicots)
HLTF	Salicaceae (Populus species)	Super-Rosids (Eudicots)
AGPC	Poaceae (grasses)	Monocots
MPAV	Poaceae	Monocots
PEPKB	Poaceae	Monocots
EXTC	Poaceae	Monocots
HPOB	Poaceae	Monocots
PEPKA	Poaceae	Monocots
HLTE	Poaceae (Non-Triticeae)	Monocots
QRA (LMW glutenins)	Poaceae (Triticeae tribe)	Monocots
QRB (HMW glutenins)	Poaceae (Triticeae tribe)	Monocots

Each TRP class is described in the supporting information (Text S1 and S2; also see Tables S6, S7, S8).

In some cases, family-specific TRP classes correlate with distinct known secretome functions indicating that phylogenetically restricted TRPs, in general, may represent important evolutionary innovations. For example, the PHEK class, only found in Fabaceae, includes an “early nodulin” thought to be involved in legume-specific symbioses with rhizobia bacteria [42], while two unique TRP classes targeted to seed storage vacuoles in the Triticeae tribe of grasses (glutenins and gliadins) are known to impart the elastomeric properties that typify cereal grain flours [43]. Though most TRP classes identified in our analysis have not yet been functionally characterized, like many TRPs throughout nature (e.g., mammalian mucins, spider silks, insect resilins, mollusc biomineralization proteins, mussel adhesive proteins), these plant structural molecules are likely to have evolutionarily important biomechanical properties that may underlie previously unexplored cell wall diversity.

Proteins with highly biased amino acid composition, like the TRP classes identified in this work (Figure S2), represent one important component of the poorly explored “dark matter” of the protein sequence universe [44]. In this study, we developed algorithms for TR identification, architecture modeling, and clustering that do not depend upon prior knowledge of TR motifs. Applying these methods to a global computational analysis of Pro-rich structural TRPs in plant secretomes, 31 TRP classes with distinct TR architectures were identified, including all previously characterized extensin and PRP TRPs. In addition to showing that one TRP class, Extensin type alpha, is the most broadly conserved Pro-rich TRP class in plant secretomes, we identified an extensin superfamily that includes several additional TRP classes with well-defined, characteristic EXT sequence features. Beyond extensins, four TRP “core modules” in land plants were identified, along with more than twenty additional classes of Pro-rich TRPs, all of which have characteristic TR architectures and limited phylogenetic distributions (e.g. the “canonical” PRPs composed of P_2_V(Y/E/H)K TRs). Taken together, our results document large-scale diversity in cell wall extensins, identify conserved core modules and distinct phylogenetic signatures in higher plant secretomes, and provide a rational taxonomy and nomenclature for the diversity in secreted Pro-rich structural proteins. In addition, this work should have applications for developing comprehensive system-level cell wall models, which have heretofore focused almost exclusively on wall polysaccharide synthesis and architecture.

## Materials and Methods

### Master Database

To study the global diversity of Pro-rich TRPs targeted to plant secretomes, we built a large database containing both expressed sequence tag (EST) data and known/hypothetical protein sequences representing diverse plant species (Table S1). All available plant EST assemblies and singletons were downloaded from two online repositories, the Gene Index Project (called “TCs”) hosted at the Dana-Farber Cancer Institute (downloaded 10/06/09 from http://compbio.dfci.harvard.edu/tgi/plant.html) [45] and the TIGR Plant Transcript Assemblies database (called “TAs”) (release 07/10/07, downloaded from ftp://ftp.tigr.org/pub/data/plantta/) [46]. We also downloaded and extracted all known and predicted plant protein sequences from the NCBI Non-Redundant (NR) protein sequence database (release 10/04/09 from http://ftp.ncbi.nlm.nih.gov/blast/db/FASTA/). ESTs were translated into forward reading frames (Table S1), and complete taxonomies (i.e. phylum, class, family, etc.) were downloaded for all sequence data from NCBI (http://www.ncbi.nlm.nih.gov/taxonomy). Of all plant species represented in the master database, 95% are severely under-sampled (at most 1–10 proteins and/or ESTs), and 0.6% of all species are represented by at least 1000 individual sequence samples (236/36,815 species). Notably, due to a lack of genome sequence data, most sequences analyzed in this work necessarily originate from specialist EST databases, not NCBI NR (see Table S1). For details of additional sequence data analyzed in this work, see Text S4.

### PlantPro20 Database Construction

We used XSTREAM, a TR identification and architecture modeling tool [47], to process the master database for TR content. The following algorithm parameters were used: Minimum Copy Number (*minC*): 2, Minimum Character Identity (*i*): 0.7, Minimum Consensus Matching (*I*): 0.8, Maximum Consecutive Gaps (*g*): 3, Minimum Domain Length (*minD*): 12 amino acids, and default parameters (refer to [47] for details of these parameters). All identified TR motifs (Table S1) were subsequently filtered for Pro-rich TRs, defined as TRs having at least 20% proline in their consensus sequences. The consensus sequence is a representative copy of the entire TR domain identified and modeled by XSTREAM.

A new database, PlantPro20, was created to house all Pro-rich TRs along with additional sequence and phylogenetic data (Table S2). Due to the high quality of TC ESTs, PlantPro20 was initially populated with Pro-rich TRs derived from translated TC sequences and singletons. Next, we added Pro-rich TRs identified from TA sequences representing plant species not already present in PlantPro20, and finally, all NR sequences with Pro-rich TRs were added (see Table S2). The longest sequence stretch containing an N-terminal methionine and downstream stop codon was identified for each EST. ORFs (full-length or partial ORFs, including NR sequences) were then individually scanned for a secretion signal and GPI anchor using SignalP 3.0 [23] and PredGPI [48] web servers, respectively. For secretory peptide identification, we required a minimum HMM signal peptide probability of 0.7 or a ‘Yes’ prediction for all Neural Network score components (i.e. max. C, max. Y, max. S, mean S, D). A minimum specificity of 99.5 (1-false positive rate) was used for the identification of GPI-anchored proteins. To focus on TR domains long enough to compose structural domains in TRPs, all TRs were additionally filtered for *TR Modules*, defined as TR domains that span at least 100 amino acids, or cover at least 33% of their parent protein sequence and span at least 50 amino acids (see Table S2).

### AutoSOME-TR

We implemented an unsupervised clustering method, called AutoSOME [49], within a software framework tailored to the unique properties of TR sequences. This new method, called AutoSOME-TR effectively clusters TR sequences with either high or low complexity amino acid compositions, captures sequence context dependency among adjacent residues, and recognizes TR phase variation using cyclical permutations (e.g., by defining a single equivalence class of TR consensus sequences: PVYK = VYKP = YKPV = KPVY).

Prior to clustering, each TR domain is converted into a dipeptide compositional vector (of length 400 to accommodate all possible dipeptides). We found empirically that such dipeptide vectors provide for better discrimination of low-complexity sequences than single amino acid vectors (length 20). As an example, given a TR domain ‘PVPVKPVPVK’ with consensus sequence ‘PVPVK’, the dipeptide vector would contain four copies of ‘PV’, two copies of ‘VP’, two copies of ‘VK’ and one copy of ‘KP’. These compositional vectors are subsequently normalized by TR domain length (10 in the previous example) and used as input for the AutoSOME-TR algorithm. Importantly, the use of dipeptide compositional vectors allows AutoSOME-TR to both capture context dependence among adjacent residues and mitigate issues due to TR phase variation.

Clustering of TRs is accomplished in two major phases. First, the entire input data set of dipeptide compositional vectors is clustered using the AutoSOME algorithm, and the resulting compositional clusters are output to memory and disk. Immediately thereafter, each TR compositional cluster is evaluated by a series of quality control procedures designed to enforce a user-specified threshold of internal homology. In brief, for each TR cluster, an alignment procedure performs an all-against-all comparison of each TR consensus sequence to all cyclical permutations of every other TR consensus sequence in the cluster, and an aggregate score records the total number of character matches. The TR consensus sequence alignment with maximal score is then used to build a master consensus sequence, which is compared to its constituents to determine a consensus error. The same consensus generation and error procedures as described for TRs in [47] are used here. Next, a procedure is invoked to flag and remove consensus sequences that are unlikely to belong to the cluster, and a consensus error is recomputed. If the lowest of the two consensus errors satisfies the user-defined homology threshold *E* ( = 0.4, by default), the corresponding TR cluster is output to file. Otherwise, AutoSOME-TR is rerun on the original compositional cluster, including any discarded consensus sequences from the cleansing step, to identify finer-grained partitions. This second stage is recursively repeated until each TR cluster either meets a stricter *E* ( = 0.3, by default) or the size of the cluster is below a minimum size threshold also modifiable by the user (10 by default). Software and pseudocode for our AutoSOME-TR implementation are available upon request.

### TR Cluster Analysis

Of the 1240 unique TR Module consensus sequences found in protein sequences with a predicted signal peptide (Table S2), 997 TR Module motifs were clustered by AutoSOME-TR. Each of these 997 TRs has periodicity>3, and lacks an ‘X’ character or stop codon. The following clustering parameters were used: 100 ensemble iterations for individual AutoSOME runs, *P*<0.01, self-organizing map (SOM) grid dimensions of 20×20 nodes for stage-one clustering and 10×10 nodes for stage-two clustering (see *AutoSOME-TR* for description of clustering stages), SOM topology = circle, and cartogram resolution of 64×64 (parameters are defined and discussed in Newman and Cooper, 2010). AutoSOME-TR identified 104 TR clusters containing 816 distinct TR Module consensus sequences and 181 singleton TR consensus sequences. See Table S9 for a comprehensive list of all clustered TR motifs, along with their corresponding proteins and plant taxa, and see Table S10 for TR cluster summary statistics.

### Construction of TR Module Cluster Network

Clusters with at least three members (73 of 104 clusters) were further analyzed for inter-cluster similarity using the AutoSOME fuzzy cluster network approach described in [49]. Both singleton TRs and TR clusters with two members (Table S10) were analyzed separately. To prevent the abundance of proline residues from skewing fuzzy clustering results, proline residues were removed from TR consensus sequences prior to fuzzy clustering. As input to the AutoSOME algorithm, all TR consensus sequences (missing proline) from each cluster were individually converted into dipeptide compositional vectors, and then averaged together to form a compositional vector representing each TR cluster. The input data set of TR compositional vectors was then unit-variance normalized over each column, converted into an all-against-all distance matrix using Pearson's correlation, and clustered by AutoSOME using a p-value threshold of 0.01, 500 ensemble iterations, SOM grid size of 12×12 nodes, SOM topology = circle, and cartogram resolution of 32×32 (parameters are described in Newman and Cooper, 2010).

The resulting fuzzy cluster network was rendered using Cytoscape 2.6.0 [50]. TR Module clusters (containing proline) were superimposed onto the network using a custom Cytoscape plug-in, and the Organic layout algorithm was used for spatial organization of clusters. Initially, the network display was highly interconnected, proving too dense for visual cluster analysis. Network density was significantly decreased by removing *insignificant* edges, defined as edges having cluster confidence less than 0.04, where confidence = 1 denotes a co-clustering of 100% over all ensemble iterations (see [49]). This step resulted in the removal of 93% of all edges (2582 of 2775) and two nodes (clusters 42 and 60). The resulting cluster similarity network, showing 73 TR Module clusters and representing 747 unique TR Module consensus sequences, is depicted in high-resolution detail in Figure S1 (also see Table S10 for cluster network statistics). Original cluster identifiers (Tables S9, S10) are provided in Figure S1 next to the TR Module clusters.

### Development of TR Taxonomy and Nomenclature

To focus on TR Module clusters representing a broad range of species and/or large number of protein sequences, a simple filter was applied to all cluster results, including the cluster network (Figure S1). Clusters that passed the filter contain TR Modules that are together present in ≥20 secreted proteins **or** are present in secreted proteins that together span ≥10 species. These clusters are shown as numbered nodes in Figure 1 (also see Table S10). Small, unlabeled nodes in Figure 1 are also shown as they have similar TR content to clusters that passed the filter.

In contrast to previous work that relied upon prior knowledge of TR sequences and composition (e.g. [15], [22]), we used unsupervised methods to identify clusters of abundant TR elements within the Pro-rich TR Module landscape (Figures 1 and S1), and subsequently devised a series of regular expression definitions to formally capture these prominent TR motifs. Regular expressions were used since, when rigorously defined, they allow for robust character matching with minimal noise. Every TR cluster satisfying the species and sequence filtration criteria or corresponding to a visible node in Figure 1 was manually analyzed for motif homogeneity, both internally and compared to neighboring clusters in the network of Figure S1 (see Table S10 for all analyzed TR clusters). While our goal was to map one unique regular expression definition per TR cluster, some clusters were split to better capture motif diversity (e.g. cluster 3 in Figure S1), and other clusters, if highly related in TR content, were combined (e.g. PELPK clusters in Figure S2). Every regular expression was fine-tuned for maximum specificity and sensitivity across the entire PlantPro20 database (Table S2), resulting in a final set of 38 distinct and highly specific regular expression definitions (Table S11).

Due to the unique structural and biochemical properties of proline residues, such as the ability to disrupt alpha helices, form polyproline helices [30], and serve as a substrate for post-translational modification [9], we further classified each of the TR classes by proline sequence architecture. Based on prominent proline backbones observed in the cluster network of Figure S1, we selected the following proline backbone categories: SP_n_, TP_n_, interspersed P_1_, regular P_2_ blocks, interspersed P_2_/P_1_, regular P_2_ and P_3_ blocks, and interspersed P_2_/P_3_/P_1_.

Finally, rather than name each TR class using arbitrary alphanumeric nomenclature (e.g. class I, II, or A, B, etc.), or a generic name like ‘PRP motif’, each TR class was named using the single letter amino acid code of a prevalent motif or sub-motif. By capturing an inherent property of the TR class within its name, this approach to nomenclature should eliminate ambiguity.

### TRP Taxonomy and Nomenclature

Using our TR taxonomy, the relevant published literature, multiple sequence alignments, and the presence or absence of conserved non-TR domains (identified using BLAST [18] and SMART [51]), all Pro-rich TRPs captured by the TR taxonomy were also classified and named. The TRP nomenclature that we developed includes both concise (3–5 letters) and descriptive names. When applicable, names were chosen to indicate hierarchical membership to larger classes, or “superfamilies” (e.g. all extensins are called ‘EXTs’). Greek characters were used for superfamily sub-types, such as “extensin type alpha,” or “TRP-AGP type gamma,” and as a general rule, increasing Greek characters (i.e. alpha, beta, gamma, etc.) indicate decreasing phylogenetic coverage in the PlantPro20 database (e.g. 10 plant families, 3 families and 10 species, 3 families and 5 species, etc.) for a given TRP superfamily.

### Sequence Redundancy and Revisions

A significant amount of sequence redundancy was observed for full-length and partial protein sequences in the PlantPro20 database. To provide the research community with an uncluttered protein sequence resource, we attempted to eliminate overlapping sequences representing the same TRP gene sequence. Since single-read EST sequencing is prone to errors, particularly within TR regions (e.g. frameshifts), further computational assembly of identified complete/partial ORFs was bypassed, and instead, all sequences with hits to the TR taxonomy (i.e. ESTs, NR sequences, genomic sequences) were manually curated using basic alignment tools and online sequence databases. The well-annotated *Arabidopsis* genome was used as a benchmark to calibrate and fine-tune the accuracy of manual sequence assembly and clustering. Multiple Sequence Alignments (MSAs) of NR and genome sequences, translated ESTs, and 5′/3′ UTRs of ESTs were all analyzed, when possible. If significant sequence homology was observed among all aligned proteins and/or transcript regions as judged by manual inspection, a master sequence was chosen to represent the group. When selecting a master sequence, precedence was given to genomic sequences, followed by NR sequences, and then translated ESTs. If more than one candidate master sequence of the same sequence type was identified (e.g. all ESTs), the longest sequence was designated as the master. In the absence of genomic or NR sequences, the “best” EST was chosen, where “best” is arbitrarily defined as the longest sequence stretch without observed errors or ambiguities (e.g. ‘X’ characters). To further increase the quality of the curated protein list, in some cases, the master sequence was revised on the basis of additional sequence information (Table S12). Revisions included extending master sequences using significantly overlapping ESTs from the same species found by NCBI BLAST analysis and correction of suspected EST frameshifts by pairwise alignments of reading frames. All curated master sequences are provided in Text S3. For all master, partial, and redundant sequences, refer to the PlantPro20Fam online database.

### PlantPro20Fam Web Database

The TRP sequence data classified and named in this work, along with all unclassified secreted Pro-rich TRPs within the PlantPro20 database, are freely available via an online repository (http://jimcooperlab.mcdb.ucsb.edu/plantpro20fam). This web resource, called PlantPro20Fam, provides a facile interface for exploring the diverse Pro-rich TR architectures and phylogenetic diversity of Pro-rich TRPs spanning >250 species ranging from green algae to flowering plants.

## Supporting Information

Figure S1 **Cluster landscape of Pro-rich TRs from plant secretomes.** High-resolution network representation of TR cluster results. Only unique consensus sequences corresponding to each TR domain are shown. Consensus motifs within each cluster were aligned as described in the *Materials and Methods*. To display amino acid physical chemical properties as RGB colors, we developed a three-dimensional representation of the following seven parameters: hydrophobicity [54], alpha helix, beta sheet, beta turn, and coil conformational parameters [55], Van der Waals volume, and isolectric point [56]; Principal Components Analysis (PCA) was used to reduce these seven property scales into three dimensions (components 1, 2, and 3), and these components were normalized into R, G, and B color elements, respectively.
(TIF)

Figure S2 **Super-classes of Pro-rich TRs, related to** **Figure 1** **.** Super-classes of TRP clusters shown in Figure 1 are indicated using the network of Figure S1.
(TIF)

Figure S3 **Evolutionary relationships among 18 PEHK loci from the** ***V. vinifera*** **genome.** Major protein domains are indicated below the alignment, including part of the TR domain (for the TR taxonomy, see Table S5), and evolutionary relationships among corresponding PEHK loci are shown on the left (bootstrap values shown above branch points). Ten PEHK genes inferred by homology searching are indicated by an asterisk on the right side of the alignment while eight previously discovered PEHK loci are given as NCBI gene identifiers. For readability, only part of the original multiple sequence alignment of PEHK protein sequences is shown (aligned residues between positions 70 and 210 were removed; rendered with JalView [57] and aligned using MUSCLE [58]). Methods: tBLASTn [18] was used to search the *V. vinifera* genome (12× database compiled 02/17/10, http://www.genoscope.cns.fr/cgi-bin/blast_server/projet_ML/blast.pl) for genes encoding proteins similar to the eight previously reported PEHKs [59], [60], resulting in the identification of 10 putative PEHK genes. To analyze evolutionary relationships among all 18 PEHK genes, a multiple sequence alignment was built for all 18 known and predicted coding regions using CLUSTALW [61], and an evolutionary tree was constructed using the Neighbor-Joining method, with 10000 bootstrap iterations. Branches with <50% bootstrap confidence are collapsed. The Jukes-Cantor method was used to estimate evolutionary distance, and positions with alignment gaps and missing data were not considered during pairwise sequence comparisons. MEGA4 was used for the entire phylogenetic analysis [62].
(TIF)

Figure S4 **Phylogenetic tree of the EXTA class, related to** **Figure 3A** **.** Full-length and/or partial EXTAs from 112 species and 72 genera are shown (orange-colored branches). The 143 leaves in these trees represent the genera that are either captured by our TR taxonomy (Tables S3, S4, S5) or represented by at least one secreted Pro-rich TRP in our analysis (Table S2). The evolutionary relationships among genera were downloaded from the NCBI taxonomy browser, and the tree was rendered using PhyloWidget [53].
(TIF)

Figure S5 **EXTA TRs have two major periodicities.** The periodicity distribution of 690 out of 698 EXTA TR domains is shown below (not shown are periods 44 (1×), 51 (1×), 53 (5×), and 76(1×)). Red, green, and blue-colored bars represent periods of length *n*, *n*−1, and *n*+1, respectively, where *n* is defined as all periodicities that are a multiple of 3. To ensure accurate characterization of TR periodicities, XSTREAM [47] was used to model the architectures of all 698 EXTA TR domains in the PlantPro20 database (Table S2) without the inclusion of gap characters (otherwise, XSTREAM was run the same as described in *Materials and Methods*).
(TIF)

Figure S6 **EXTB TRs have two major periodicities.** This histogram shows the periodicity distribution of 172 out of 173 EXTB TR domains (one instance of period = 40 is not shown). The histogram is colored identically to Figure S5. XSTREAM [47] was used to model all EXTB TR architectures the same as described in Figure S5.
(TIF)

Figure S7 **Phylogenetic tree of the AGPA class, related to** **Figure 3B** **.** AGPAs were found as partial or complete sequences in 38 plant genera (orange-colored branches and leaves). The tree was built identically to the tree in Figure S4.
(TIF)

Figure S8 **Phylogenetic tree of the PELPK class, related to** **Figure 3C** **.** PELPKs (partial or complete sequences) were found in 45 plant genera (orange-colored branches and leaves). The tree was built identically to the tree in Figure S4.
(TIF)

Figure S9 **Phylogenetic tree of the HLTA class.** HLTAs were found as partial or complete sequences in 46 plant genera (orange-colored branches and leaves). The tree was built identically to the tree in Figure S4.
(TIF)

Figure S10 **Phylogenetic tree of the HPOA class.** HPOAs (partial or complete sequences) were found in 37 plant genera (orange-colored branches and leaves). The tree was built identically to the tree in Figure S4.
(TIF)

Table S1 **Summary statistics of plant master database.** Primary sequence data used to develop the TR and TRP taxonomies in this work. For further details, see *Materials and Methods* in the primary text.
(DOC)

Table S2 **Summary statistics of PlantPro20 database.** Details of Pro-rich TRs with at least 20% proline content (see *Materials and Methods* in the primary text).
(DOC)

Table S3 **Ser/Thr-(Pro)_n_ TR classes and corresponding TRP classes.**
(DOC)

Table S4 **Interspersed (Pro)_1_ and (Pro)_1_/(Pro)_2_ TR classes and corresponding TRP classes.**
(DOC)

Table S5 **TR classes with regular (Pro)_2_ blocks or (Pro)_2_ interspersed with (Pro)_1_ and/or (Pro)_3_, and corresponding TRP classes.**
(DOC)

Table S6 **Ser/Thr-(Pro)_n_–containing TRP classes.**
(DOC)

Table S7 **PRP and Hybrid PRP TRP classes.**
(DOC)

Table S8 **Additional Pro-rich TRP classes.**
(DOC)

Table S9 **Full details of all Pro-rich TR clusters found in the PlantPro20 database.**
(XLS)

Table S10 **Summary statistics of all identified Pro-rich TR clusters with respect to fuzzy cluster diagrams and the TR taxonomy.**
(XLS)

Table S11 **38 TR regular expression queries corresponding to TR taxonomy.** Each regular expression was used to scan TR consensus sequences in PlantPro20 (Table S2) to identify protein sequences with similar TR content. Amino acids enclosed in square brackets represent more than one possible match (e.g. [ST] means either serine or threonine is a match), ‘.’ denotes a wildcard character, ‘∧’ preceding an amino acid in closed square brackets denotes ‘NOT’ (e.g. [∧P], meaning that proline is excluded), and numbers in closed curly brackets indicate how many times a particular residue must be repeated for a match (e.g. P{2,4} means proline must be found 2–4 times in tandem for a match). Finally, when more than one motif is a match, each motif is separated by ‘|’ and the entire expression is bound by square brackets (e.g. [AP|ST] means either AP or ST is a match). For example, the following regular expression for TR class tp3a, ‘[TS]P{3,4}[VA]{1,2}[TS].P and not [HY]’, should be read as: T or S, followed by 3–4 repeats of P, followed by 1–2 repeats of V or A, followed by T or S, any character followed by P, and never a match of H or Y.
(DOC)

Table S12 **Documentation for revised master sequences.** Several master sequences in the curated sequence list (Text S3) were derived from ESTs representing partial ORFs or ESTs with evidence of a frameshift. We attempted to revise these master sequences based on additional database searching, multiple sequence alignment, or combining reading frames with evidence of a frameshift. Actions taken to “fix” these sequences are given below. The number immediately following some sequence identifiers in *Revision(s)* denotes the forward reading frame used to revise the master sequence.
(DOC)

Table S13 **EST tissue/organ source data for all 31 TRP classes.**
(XLS)

Text S1 **TR and TRP taxonomies.**
(DOC)

Text S2 **Additional details of plant Pro-rich TRP classes.**
(DOC)

Text S3 **Manually curated list of Pro-rich TRPs.** A list of 912 non-redundant TRPs (“master sequences”) catalogued in the TRP taxonomy (Tables S6, S7, S8), 907 of which have a predicted signal peptide [23].
(DOC)

Text S4 **Additional genome sequence data.**
(DOC)

Dataset S1 **TR architectures of representative proteins from 31 Pro-rich TRP classes.** TR architectures of representative protein examples for each of 31 TRP classes (Tables S6, S7, S8) are illustrated. All TRs were identified and aligned using XSTREAM [47] using the same parameters described in *Materials and Methods*. Each protein is shown N-terminus to C-terminus, from left to right and top to bottom. Major sequence features, in addition to TR domains, are indicated. For TR domains classified by the TR taxonomy (see Tables S3, S4, S5), the corresponding TR class is given in parentheses, e.g. TR_Domain1(mtp2). The scale-bar on top shows the number of amino acids from left to right in a given row. All 31 representative proteins are listed in alphabetical order and all images were rendered with JalView [57].
(PDF)

Dataset S2 **Representative multiple sequence alignments.** Multiple sequence alignments of the 50 N-terminal and 50 C-terminal amino acids of full-length ORFs from 26 of 31 TRP classes (see Tables S6, S7, S8) are shown. Sequences were preprocessed to remove most of the TR domain to increase alignment quality. (PHEK and PRPB classes are not shown due to low quantities of complete ORFs; both EXTM and SPAP are heterogeneous protein classes, and are therefore not shown; PEHKs are shown in Figure S3). Each aligned TRP sequence (obtained from Text S3) has a predicted secretion signal, and is either derived from a genome sequence project, the NR database, or an EST, in which case a predicted stop codon was required. All sequence alignments were created with MUSCLE [58] and rendered using JalView [57].
(PDF)
