# Supplementary figures and images for: Global Analysis of Proline-Rich Tandem Repeat Proteins Reveals Broad Phylogenetic Diversity in Plant Secretomes
Source: PLoS One. 2011 Aug 2;6(8):e23167. doi: 10.1371/journal.pone.0023167 (PMC3149072; doi:10.1371/journal.pone.0023167)

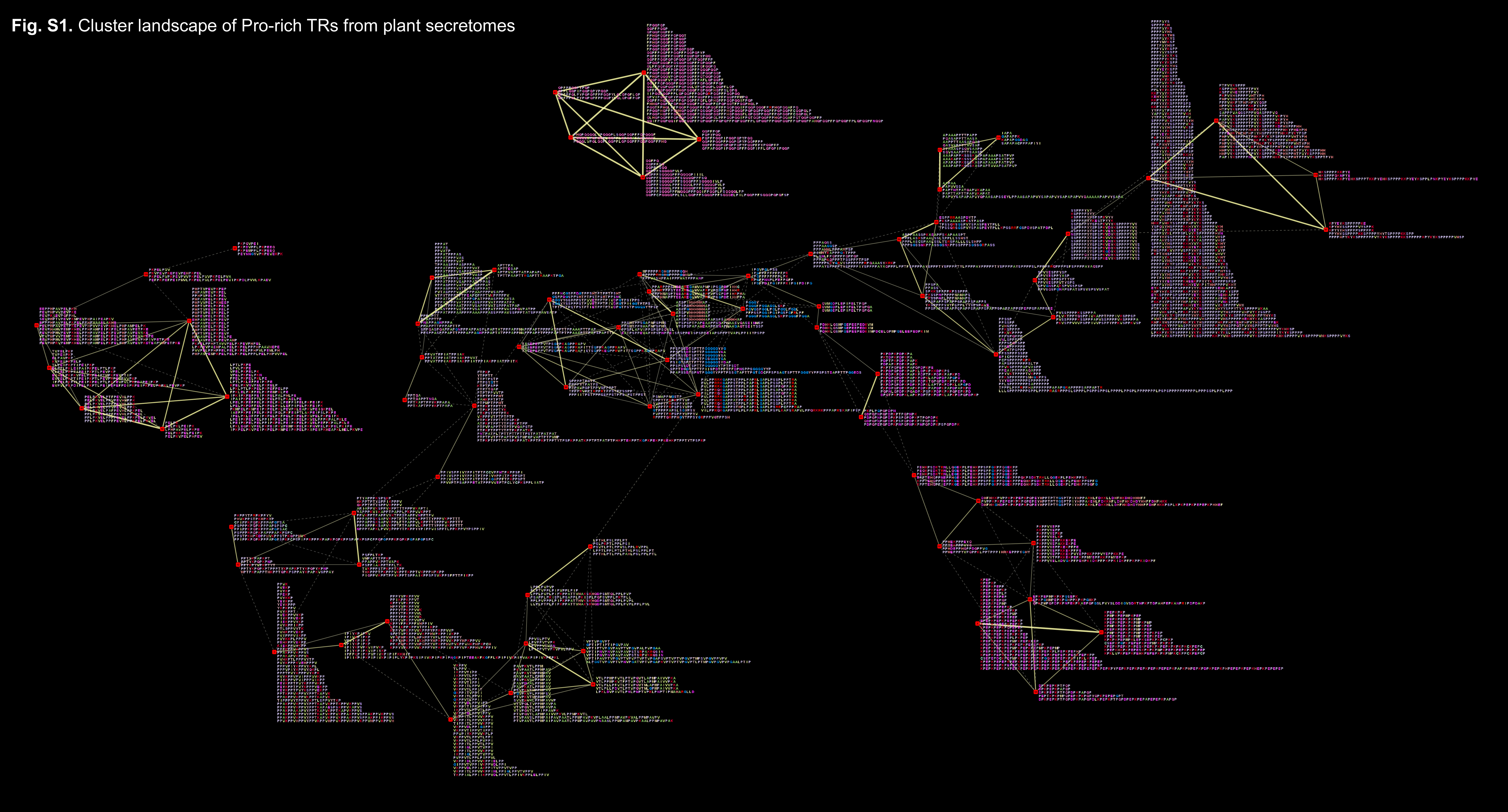

Supplement: Figure S1 — Cluster landscape of Pro-rich TRs from plant secretomes. High-resolution network representation of TR cluster results. Only unique consensus sequences corresponding to each TR domain are shown. Consensus motifs within each cluster were aligned as described in the Materials and Methods . To display amino acid physical chemical properties as RGB colors, we developed a three-dimensional representation of the following seven parameters: hydrophobicity [54], alpha helix, beta sheet, beta turn, and coil conformational parameters [55], Van der Waals volume, and isolectric point [56]; Principal Components Analysis (PCA) was used to reduce these seven property scales into three dimensions (components 1, 2, and 3), and these components were normalized into R, G, and B color elements, respectively. (TIF) [file pone.0023167.s001.tif]

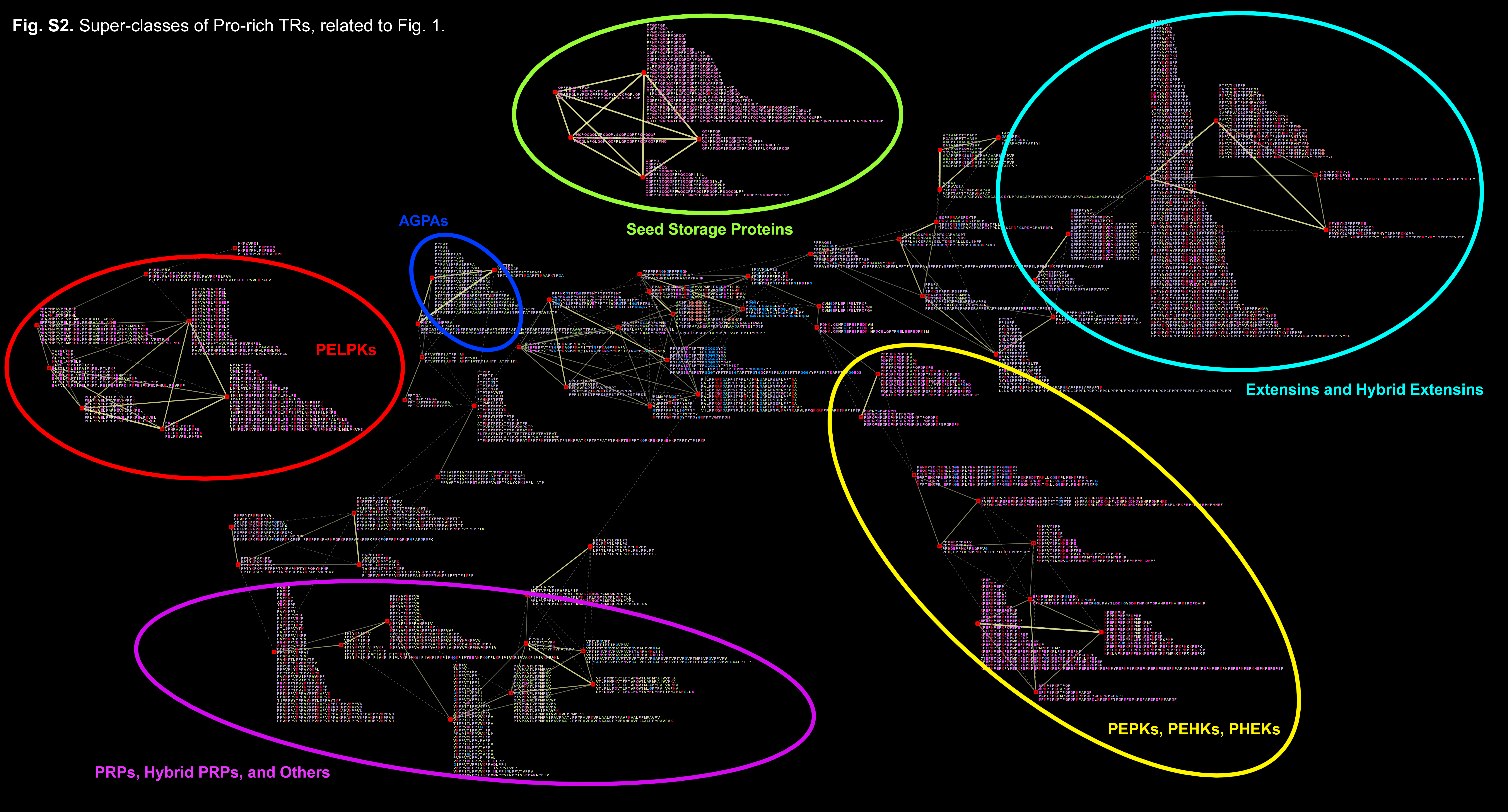

Supplement: Figure S2 — Super-classes of Pro-rich TRs, related to Figure 1 . Super-classes of TRP clusters shown in Figure 1 are indicated using the network of Figure S1. (TIF) [file pone.0023167.s002.tif]
