# Supplementary material for: Global Analysis of Proline-Rich Tandem Repeat Proteins Reveals Broad Phylogenetic Diversity in Plant Secretomes
Source: PLoS One. 2011 Aug 2;6(8):e23167. doi: 10.1371/journal.pone.0023167 (PMC3149072; doi:10.1371/journal.pone.0023167)
Supplement: Table S1 — Summary statistics of plant master database. Primary sequence data used to develop the TR and TRP taxonomies in this work. For further details, see Materials and Methods in the primary text. (DOC) [file pone.0023167.s011.doc]

**Table S1. Summary statistics of plant master database.**

| **Primary Database** | **No. Species** | **No. Sequences** | **Translated**  **Sequences** | **No. TR Motifs** | **No. Sequences**  **with TR Content** |
| --- | --- | --- | --- | --- | --- |
| TC^a^ | 47 | 3,443,001 | 10,329,003 | 835,854 | 647,763 |
| TA^b^ | 254 | 4,134,956 | 12,404,868 | 849,937 | 668,022 |
| NR (plants only)^c^ | 36,790 | 697,190 | 697,190 | 82,653 | 52,234 |
|  |  |  |  |  |  |
| **Master Database** | **36,815** | **8,275,147** | **23,431,061** | **1,768,444** | **1,368,019** |

Primary sequence data used in this work. For further details, see *Materials and Methods* in the primary text.

^a^ Gene Index Project (downloaded 10/06/09 from http://compbio.dfci.harvard.edu/tgi/plant.html)

^b^ TIGR Plant Transcript Assemblies (final release 07/10/07; ftp://ftp.tigr.org/pub/data/plantta/)

^c^ Plant sequences from NCBI NR (downloaded 10/04/09; ftp.ncbi.nlm.nih.gov/blast/db/FASTA/)
