# Supplementary material for: Global Analysis of Proline-Rich Tandem Repeat Proteins Reveals Broad Phylogenetic Diversity in Plant Secretomes
Source: PLoS One. 2011 Aug 2;6(8):e23167. doi: 10.1371/journal.pone.0023167 (PMC3149072; doi:10.1371/journal.pone.0023167)
Supplement: Table S2 — Summary statistics of PlantPro20 database. Details of Pro-rich TRs with at least 20% proline content (see Materials and Methods in the primary text). (DOC) [file pone.0023167.s012.doc]

**Table S2. Summary statistics of PlantPro20 database.**

| **Database** | **Plant Taxonomy** | | | | **No. Sequences** | **No. Secreted Sequences^a^** | **No. TR Motifs** | **No. TR Modules^b^** | **No. TR Modules in Secreted Sequences** | **Secreted TRPs^c^** |
| --- | --- | --- | --- | --- | --- | --- | --- | --- | --- | --- |
|  | **Orders** | **Families** | **Genera** | **Species** |  |  |  |  |  |  |
| TC | 18 | 21 | 40 | 47 | 125,021 | 2429 | 161,234 | 6574 | 1535 | 1501 |
| TA | 51 | 76 | 135 | 190 | 23,343 | 633 | 30,574 | 1689 | 393 | 395 |
| NR | 44 | 81 | 176 | 320 | 11,509 | 1254 | 18,265 | 1051 | 617 | 596 |
|  |  |  |  |  |  |  |  |  |  |  |
| **PlantPro20** | **61** | **113** | **250** | **462** | **159,873** | **4316** | **210,073^d^** | **9314^e^** | **2545^f^** | **2492** |

Details of Pro-rich TRs with at least 20% proline content (see *Materials and Methods* in the primary text).

^a^ ORFs (full or partial sequence with N-terminal Met) with a predicted secretion signal

^b^ TR Module is defined as a TR with length≥100 amino acids or length≥50 amino acids and length≥33% of the protein sequence.

^c^ Secreted TRP defined as a protein sequence^a^ with at least one Pro-rich TR Module^b^.

^d^ Using 100% consensus sequence identity, 43% of all TR motifs (89,938/210,073) are unique.

^e^ Using 100% consensus sequence identity, 56% of all TR Modules (5122/9314) are unique.

^f^ 49% of all TR Modules in secreted sequences (1240/2545) are unique.
