# Supplementary material for: Global Analysis of Proline-Rich Tandem Repeat Proteins Reveals Broad Phylogenetic Diversity in Plant Secretomes
Source: PLoS One. 2011 Aug 2;6(8):e23167. doi: 10.1371/journal.pone.0023167 (PMC3149072; doi:10.1371/journal.pone.0023167)
Supplement: Table S3 — Ser/Thr-(Pro)n TR classes and corresponding TRP classes. (DOC) [file pone.0023167.s013.doc]

**Table S3. Ser/Thr-(Pro)_n_ TR classes and corresponding TRP classes.**

| **Proline**  **Class** | **TR Class**  **Name** | **Major TRP Class** | **Representative TR Motif(s)** | **Common**  **Period(s)** |
| --- | --- | --- | --- | --- |
|  |  |  |  |  |
| SPn | Unclassified | SPn | Diverse, Low-Complexity | - |
| SP_1_ | spap2 | SPAP | SPAPP | 5 |
|  | sp,tp2ty | EXTC | SPKPTPPTYTP SPKPPTPKPTPPTYTP | 11  16 |
| SP_2_ | sp2,spvyx | EXTD | SPPYSPSPVYE  SPPSPTYSPSPVYK | 11  14 |
|  | sp2,yxy | EXTA | SPPYEYK  SPPSYSYPAPEYK | 7  13 |
|  | sp2,vyk | EXTB | SPPVHKYPPPTPVYK | 15 |
|  | sp2yvp2y | HLTE | SPPYVPPYVPPTPRP | 15 |
| SP_2,3_ | sp2,3-misc | EXTM | SPPSPTPPVYSPPKHPHYPPK | 21 |
| SP_3_ | sp3,yxy | EXTA | SPPPVHYEYK  SPPPYDYKPPYYYSTP | 10  16 |
|  | sp3vyk | EXTB | SPPPVYKPPPVY | 12 |
| SP_4_ | sp4vh | HEXA | SPPPPVH | 7 |
| SP_4+_ | sp4+,vyk | EXTB | SPPPPVYK SPPPPTPVYK | 8  10 |
|  | sp4+,yxy | EXTA | SPPPPVYKYK  SPPPPSPSPPPPYYYK | 10  16 |
|  |  |  |  |  |
| TP_2_ | mtp2 | AGPB | MTPPSPT MTPPSAAPMPSPMSPPS | 7  17 |
| TP_3_ | mtp3 | AGPC | MTPPPMP MTPPPMPM | 7  8 |
|  | tp3a | AGPA | TPPPA | 5 |

Underlined characters correspond to the prominent sub-motif represented by the TR class name. For explicit TR class definitions, see Table S11.
