# Supplementary material for: Global Analysis of Proline-Rich Tandem Repeat Proteins Reveals Broad Phylogenetic Diversity in Plant Secretomes
Source: PLoS One. 2011 Aug 2;6(8):e23167. doi: 10.1371/journal.pone.0023167 (PMC3149072; doi:10.1371/journal.pone.0023167)
Supplement: Table S4 — Interspersed (Pro)1 and (Pro)1/(Pro)2 TR classes and corresponding TRP classes. (DOC) [file pone.0023167.s014.doc]

**Table S4. Interspersed (Pro)_1_ and (Pro)_1_/(Pro)_2_ TR classes and corresponding TRP classes.**

| **Proline**  **Class** | **TR**  **Class**  **Name** | **Major TRP Class** | **Representative TR Motif(s)** | **Common**  **Period(s)** |
| --- | --- | --- | --- | --- |
|  |  |  |  |  |
| P_1_ | pqq | QRA | PQQPFPQQ  PQQPYPQPQPFP | 8  12 |
|  | gqq | QRB | GQQGYYPTSPQQPGQ  GQQGYYPTSPQQPGQGQQPGQGQQPGQ | 15  27 |
|  | kpip | KPIP | KPIPIVKPIP | 10 |
|  | pelpk1 (-His) | PELPK | PELPKPEL  PELPKPELPKV | 8  11 |
| P_1_/P_2_ | pelpk2 (+His) | PELPK | PELPKPELPHPAV  PELPKPELPPHPAV | 13  14 |
| P_1_ | pepk | PEPKA | PEPK  PEPKPEPKPEPMPK | 4  14 |
|  | pdpk | PEPKB | PDPKPEPKPAPQ  PDPKPEPKPTPQPEPKPAPQ | 12  20 |
| P_1_/P_2_ | pepk  -embed1 | HPOB | PEPKPQPEPEPDHFHKDHDYHHFFDHFH-  KKPMPPKPK | 37 |
|  | pepk  -embed2 | PEPKC | PEPKPPVYEPPKKEK | 15 |

Underlined characters correspond to the prominent sub-motif represented by the TR class name. For explicit TR class definitions, see Table S11.
