# Supplementary material for: Global Analysis of Proline-Rich Tandem Repeat Proteins Reveals Broad Phylogenetic Diversity in Plant Secretomes
Source: PLoS One. 2011 Aug 2;6(8):e23167. doi: 10.1371/journal.pone.0023167 (PMC3149072; doi:10.1371/journal.pone.0023167)
Supplement: Table S5 — TR classes with regular (Pro)2 blocks or (Pro)2 interspersed with (Pro)1 and/or (Pro)3, and corresponding TRP classes. (DOC) [file pone.0023167.s015.doc]

**Table S5. TR classes with regular (Pro)_2_ blocks or (Pro)_2_ interspersed with (Pro)_1_ and/or (Pro)_3_, and corresponding TRP classes.**

| **Proline**  **Class** | **TR**  **Class**  **Name** | **Major TRP Class** | **Representative TR Motif(s)** | **Common**  **Period(s)** |
| --- | --- | --- | --- | --- |
|  |  |  |  |  |
| P_2_ | p2vtl | HLTA | PPVTLPPVVK | 10 |
|  | p2vtp | HLTF | PPVTPPIKPPK PPVTPPIKPPKPPIKPPK | 11  18 |
|  | p2vyk | PRPA | PPVYK  PPVYKPPVEK | 5  10 |
|  |  |  |  |  |
| P_2_/P_1_ | p2vk | HPOC | PPVK | 4 |
|  | p2vtv | HLTC | PPVTVPKLPV | 10 |
|  | p2mpav | MPAV | PPMPAVPTVPAVTL | 14 |
|  |  |  |  |  |
| P_2_/P_3_ | p2hek1 | PHEK | PPHEKPPPEYQ PPHEKPPHEHPPPEYQ | 11  16 |
|  | p2hek2 | PHEK | PPHEKPPHEKPPPVYE | 16 |
|  |  |  |  |  |
| P_2_/P_3_/P_1_ | p3vyk | PRPB | PPPVYKPPIK  PPPVYKPPVPVIPPPVVK | 10  18 |
|  | p3vpvyk | HPOA | PPPVPVYKKPL PPPVPVYKPPVVKPL | 11  15 |
|  | p2tvk | HLTD | PPTVKPPPSTPKPPTK | 16 |
|  | p2yxpkp2 | HLTB | PPYVPKPPVVK | 11 |
|  | p3ehk1 | PEHK | PPPEHKPPVEK | 11 |
|  | p3ehk2 | PEHK | PPPEHKPSDKTRNLLEGEKPLPEHKPPSPFGKPPQGEK | 38 |

Underlined characters correspond to the prominent sub-motif represented by the TR class name. For explicit TR class definitions, see Table S11.
