# Supplementary material for: Global Analysis of Proline-Rich Tandem Repeat Proteins Reveals Broad Phylogenetic Diversity in Plant Secretomes
Source: PLoS One. 2011 Aug 2;6(8):e23167. doi: 10.1371/journal.pone.0023167 (PMC3149072; doi:10.1371/journal.pone.0023167)
Supplement: Table S6 — Ser/Thr-(Pro)n–containing TRP classes. (DOC) [file pone.0023167.s016.doc]

**Table S6. Ser/Thr-(Pro)_n_–containing TRP classes.**

| **Abbrev.**  **Name** | **Name** | **Major Phylogeny** | **No. Master Sequences Identified^a^**  **(No. ESTs)** | **Example(s) of Previously Published Sequence(s)** | **Reference(s)** |
| --- | --- | --- | --- | --- | --- |
|  |  |  |  |  |  |
| *Simple SP_n_ Proteins* | |  |  |  |  |
| SP_n_ | SP_n_ unclassified proteins | Ubiquitous | - |  |  |
| SPAP | SPAP domain protein | Green Algae, Mostly *Chlamydomonas* | 23 (1) | e.g. Plus/Minus Agglutinins | [1] |
| *Extensins* | |  |  |  |  |
| EXTA | Extensin, type alpha | Liverworts thru Non-Grass Angiosperms | 155 (98) | Extensin P2-type, P3-type | [2,3] |
| EXTB | Extensin, type beta | Non-Grass Angiosperms | 16 (11) | Extensin P1-type | [2,3] |
| EXTC | Extensin, type gamma | Grasses | 9 (4) | ZmTHRGP | [4] |
| EXTD | Extensin, type delta | Mosses | 8 (7) | *None Found* | *None Found* |
| EXTM | Extensin, type miscellaneous S/T-P_2,3_ | Mostly Rosids | 10 (6) | AtPRP1, AtPRP3 | [5] |
| HEXA | Hybrid Extensin, type alpha | Eudicots and Grasses | 15 (2) | LRXs, PEXs | [6] |
| *TR Arabinogalactan Protein(-Like)* | |  |  |  |  |
| AGPA | TR-AGP, type alpha | Conifers, Angiosperms | 89 (74) | AtAGP7, AtAGP9 | [7] |
| AGPB | TR-AGP, type beta | Eudicots | 13 (11) | *None Found* | *None Found* |
| AGPC | TR-AGP/Pfam022298 fusion, type gamma | Grasses | 4 (2) | *None Found* | *None Found* |

^a^ Master sequences are the non-redundant protein sequences in Text S3. The number of ESTs shown in parentheses refers to the number of master sequences in Text S3 predicted from ESTs (these sequences were not found to be represented in NR or any genome sequence data set).

**References**

1. Lee JH, Waffenschmidt S, Small L, Goodenough U (2007) Between-species analysis of short-repeat modules in cell wall and sex-related hydroxyproline-rich glycoproteins of *Chlamydomonas.* Plant Physiol 144: 1813-1826.
2. Smith JJ, Muldoon EP, Willard JJ, Lamport DTA (1986) Tomato extensin precursors P1 and P2 are highly periodic structures. Phytochemistry 25: 1021-1030.
3. Held MA, Tan L, Kamyab A, Hare M, Shpak E, et al. (2004) Di-isodityrosine is the intermolecular cross-link of isodityrosine-rich extensin analogs cross-linked *in vitro.* J Biol Chem 279: 55474-55482.
4. Kieliszewski MJ, Lamport DTA (1987) Purification and partial characterization of a hydroxyproline-rich glycoprotein in a graminaceous monocot, *Zea mays*. Plant Physiol 85: 823-827.
5. Fowler TJ, Bernhardt C, Tierney ML (1999) Characterization and expression of four proline-rich cell wall protein genes in Arabidopsis encoding two distinct subsets of multiple domain proteins. Plant Physiol 121: 1081-1091.
6. Baumberger N, Doesseger B, Guyot R, Diet A, Parsons RL, et al. (2003) Whole-genome comparison of leucine-rich repeat extensins in *Arabidopsis* and rice. A conserved family of cell wall proteins form a vegetative and a reproductive clade. Plant Physiol 131: 1313-1326.
7. Schultz CJ, Johnson KL, Currie G, Bacic A (2000) The classical arabinogalactan protein gene family of *Arabidopsis*. Plant Cell 12: 1751-1768.
