# Supplementary material for: Global Analysis of Proline-Rich Tandem Repeat Proteins Reveals Broad Phylogenetic Diversity in Plant Secretomes
Source: PLoS One. 2011 Aug 2;6(8):e23167. doi: 10.1371/journal.pone.0023167 (PMC3149072; doi:10.1371/journal.pone.0023167)
Supplement: Table S7 — PRP and Hybrid PRP TRP classes. (DOC) [file pone.0023167.s017.doc]

**Table S7. PRP and Hybrid PRP TRP classes.**

| **Abbrev.**  **Name** | **Name** | **Major Phylogeny** | **No. Master Sequences**  **Identified^a^**  **(No. ESTs)** | **Example(s) of**  **Previously Published Sequence(s)** | **Reference(s)** |
| --- | --- | --- | --- | --- | --- |
| PRPA | Proline-Rich Protein type alpha | Carrot, Legumes, *Populus*, *Triphysaria* Species | 32 (17) | MtPRPs | [1-3] |
| PRPB | Proline-Rich Protein type beta | Conifers, Basal Angiosperm | 2 (2) | Douglas fir PHRGP | [4] |
| *Hybrid PRPs* | |  |  |  |  |
| HLTA^+^ | Hybrid PRP/Lipid Transfer Protein type alpha | Conifers,  Non-Grass Angiosperms | 67 (64) | St2 | [5] |
| HLTB^+^ | Hybrid PRP/LTP type beta | Eudicots | 29 (24) | MsPRP2 | [6] |
| HLTC^+^ | Hybrid PRP/LTP type gamma | Higher Eudicots | 7 (6) | *None Found* | *None Found* |
| HLTD^+^ | Hybrid PRP/LTP type delta | Brassicaceae | 11 (8) | BnPRP | [7] |
| HLTE^+^ | Hybrid PRP/LTP type epsilon | Non-Triticeae Grasses | 6 (4) | ZmHyPRP | [8] |
| HLTF^+^ | Hybrid PRP/LTP type phi | Populus Species | 3 (3) | *None Found* | *None Found* |
| HPOA^++^ | Hybrid PRP/Pollen Ole e I type alpha | Non-Grass Angiosperms | 28 (22) | AtPRP2, AtPRP4 | [9] |
| HPOB^++^ | Hybrid PRP/Pollen Ole e I type beta | Grasses | 5 (2) | OsPRPs | [10,11] |
| HPOC^+^ | Hybrid PRP/Pollen Ole e I type gamma (AGP) | Higher Eudicots | 14 (9) | DcAGP1, AGP31 | [12, 13] |

^a^ same as Table S6

^+^ N-terminal Pro-rich TR domain; ^++^ C-terminal Pro-rich TR domain

**References**

1. Chen J, Varner JE (1985) Isolation and characterization of cDNA clones for carrot extensin and a proline-rich 33-kDa protein. Proc Natl Acad Sci USA 82: 4399-4403.
2. Wilson RC, Cooper JB (1994) Characterization of PRP1 and PRP2 from *Medicago truncatula.* Plant Physiol 105: 445-446.
3. Wilson RC, Long F, Maruoka EM, Cooper JB (1994) A new proline-rich early nodulin from *Medicago truncatula* is highly expressed in nodule meristematic cells. Plant Cell 6: 1265-1275.
4. Kieliszewski M, de Zacks R, Leykam JF, Lamport DTA (1992) A repetitive proline-rich protein from the gymnosperm douglas fir is a hydroxyproline-rich glycoprotein. Plant Physiol 98: 919-926.
5. Dvořáková L, Cvrčková F, Fischer L (2007) Analysis of the hybrid proline-rich families from seven plant species suggests rapid diversification of their sequences and expression patterns. BMC Genomics 8: 412.
6. Deutch CE, Winicov I (1995) Post-trancriptional regulation of a salt-inducible alfalfa gene encoding a putative chimeric proline-rich cell wall protein. Plant Mol Biol 27: 411-418.
7. Goodwin W, Pallas JA, Jenkins GI (1996) Transcripts of a gene encoding a putative cell wall-plasma membrane linker protein are specifically cold-induced in *Brassica napus*. Plant Mol Biol 31: 771-781.
8. José-Estanyol M, Ruiz-Avila L, Puigdomenech P (1992) A maize embryo-specific gene encodes a proline-rich and hydrophobic protein. Plant Cell 4: 413-423.
9. Fowler TJ, Bernhardt C, Tierney ML (1999) Characterization and expression of four proline-rich cell wall protein genes in Arabidopsis encoding two distinct subsets of multiple domain proteins. Plant Physiol 121: 1081-1091.
10. Wang R, Chong K, Wang T (2006) Divergence in spatial expression patterns and in response to stimuli of tandem-repeat paralogues encoding a novel class of proline-rich proteins in *Oryza sativa*. J Exp Bot 57: 2887-2897.
11. Gothandam KM, Nalini E, Karthikeyan S, Shin JS (2010) OsPRP3, a flower specific proline-rich protein of rice, determines extracellular matrix structure of floral organs and its overexpression confers cold-tolerance. Plant Mol Biol 72: 125-135.
12. Baldwin TC, Domingo C, Schindler T, Seetharaman G, Stacey N, et al. (2001) DcAGP1, a secreted arabinogalactan protein, is related to a family of basic proline-rich proteins. Plant Mol Biol 45: 421-435.
13. Liu C, Mehdy M (2007) A nonclassical arabinogalactan protein gene highly expressed in vascular tissues, AGP31, is transcriptionally repressed by methyl jasmonic acid in *Arabidopsis*. Plant Physiol 145: 863-874.
