# Supplementary material for: Global Analysis of Proline-Rich Tandem Repeat Proteins Reveals Broad Phylogenetic Diversity in Plant Secretomes
Source: PLoS One. 2011 Aug 2;6(8):e23167. doi: 10.1371/journal.pone.0023167 (PMC3149072; doi:10.1371/journal.pone.0023167)
Supplement: Table S8 — Additional Pro-rich TRP classes. (DOC) [file pone.0023167.s018.doc]

**Table S8. Additional Pro-rich TRP classes.**

| **Abbrev.**  **Name** | **Name** | **Major Phylogeny** | **No. Master Sequences**  **Identified^a^**  **(No. ESTs)** | **Example(s) of**  **Previously Published Sequence(s)** | **Reference(s)** |
| --- | --- | --- | --- | --- | --- |
| PELPK | PELPK domain protein | Angiosperms | 107 (90) | At5g09520, At5g09530 | [1,2] |
| PEPKA | PEPK type alpha | Grasses | 22 (16) | WPRP1 | [3] |
| PEPKB | PEPK type beta | Grasses | 17 (14) | *None Found* | *None Found* |
| PEPKC | PEPK type gamma | Rosids, Mostly Malvaceae | 8 (4) | GhPRP1, GhPRP2 | [4,5] |
| KPIP | KPIP domain protein | Legumes | 10 (7) | *None Found* | *None Found* |
| MPAV | MPAV domain protein | Grasses | 39 (26) | *None Found* | *None Found* |
| PEHK | PEHK domain protein | Grape Family | 22 (14^b^) | VvPRPs | [6,7] |
| PHEK | PHEK domain protein | Legumes | 6 (1) | ENOD2 | [8] |
|  |  |  |  |  |  |
| *Seed Storage Proteins* | |  |  |  |  |
| QRA | Gln-rich type alpha | Triticeae Grasses | 112 (3) | Gliadins, LMW subunits of Glutenin | [9] |
| QRB | Gln-rich type beta | Triticeae Grasses | 23 (0) | HMW subunits of Glutenin | [9] |

^a^ same as Table S6

^b^  4 ESTs + 10 tBLASTn genome predictions from *V. vinifera* (see Figure S3).

**References**

1. Johnson KL, Jones BJ, Schultz CJ, Bacic A (2003) Non-enzymic cell wall (glyco)proteins. In: Rose JKC, editor. The Plant Cell Wall, Vol. 8. Florida: CRC Press. pp. 111-154.
2. Showalter AM, Keppler B, Lichtenberg J, Gu D, Welch LR (2010) A bioinformatics approach to the identification, classification, and analysis of hydroxyproline-rich glycoproteins. Plant Physiol 153: 485-513.
3. Raines CA, Lloyd JC, Chao S, John UP, Murphy GJP (1991) A novel proline-rich protein from wheat. Plant Mol Biol 16: 663-670.
4. Orford SJ, Timmis JN (1997) Abundant mRNA specific to the developing cotton fibre. Theor Appl Genet 94: 909-918.
5. Tan H, Creech RG, Jenkins JN, Chang Y, Ma D (2001) Cloning and expression analysis of two cotton (*Gossypium hirsutum* L.) genes encoding cell wall proline-rich proteins. DNA Seq 12: 367-380.
6. Davies C, Robinson SP (2000) Differential screening indicates a dramatic change in mRNA profiles during grape berry ripening. Cloning and characterization of cDNAs encoding putative cell wall stress response proteins. Plant Physiol 122: 803-812.
7. Thomas P, Lee MM, Schiefelbein J (2003) Molecular identification of proline-rich protein genes induced during root formation in grape (*Vitis vinifera* L.) stem cuttings. Plant Cell Environ 26: 1497-1504.
8. Franssen HJ, Nap JP, Gloudemans T, Stiekema W, Van Dam H, et al. (1987) Characterization of cDNA for nodulin-75 of soybean: A gene product involved in early stages of root nodule development. Proc Natl Acad Sci USA 84: 4495-4499.
9. [Shewry](http://jxb.oxfordjournals.org/search?author1=Peter+R.+Shewry&sortspec=date&submit=Submit) PR, [Halford](http://jxb.oxfordjournals.org/search?author1=Nigel+G.+Halford&sortspec=date&submit=Submit) NG (2002) Cereal seed storage proteins: structures, properties and role in grain utilization. J Exp Bot 53: 947-958.
