# Supplementary material for: Global Analysis of Proline-Rich Tandem Repeat Proteins Reveals Broad Phylogenetic Diversity in Plant Secretomes
Source: PLoS One. 2011 Aug 2;6(8):e23167. doi: 10.1371/journal.pone.0023167 (PMC3149072; doi:10.1371/journal.pone.0023167)
Supplement: Table S11 — 38 TR regular expression queries corresponding to TR taxonomy. Each regular expression was used to scan TR consensus sequences in PlantPro20 (Table S2) to identify protein sequences with similar TR content. Amino acids enclosed in square brackets represent more than one possible match (e.g. [ST] means either serine or threonine is a match), ‘.’ denotes a wildcard character, ‘∧’ preceding an amino acid in closed square brackets denotes ‘NOT’ (e.g. [∧P], meaning that proline is excluded), and numbers in closed curly brackets indicate how many times a particular residue must be repeated for a match (e.g. P{2,4} means proline must be found 2–4 times in tandem for a match). Finally, when more than one motif is a match, each motif is separated by ‘|’ and the entire expression is bound by square brackets (e.g. [AP|ST] means either AP or ST is a match). For example, the following regular expression for TR class tp3a, ‘[TS]P{3,4}[VA]{1,2}[TS].P and not [HY]’, should be read as: T or S, followed by 3–4 repeats of P, followed by 1–2 repeats of V or A, followed by T or S, any character followed by P, and never a match of H or Y. (DOC) [file pone.0023167.s021.doc]

**Table S11. 38 TR regular expression queries corresponding to TR taxonomy.**

| **TR Class** | **Regular Expression** |
| --- | --- |
| spap2 | SPAPPS |
| sp,tp2ty | PKPT.P[PTSA]Y[TAK] |
| sp2,spvyx | SP{1,2}VY.SPP[^P] and not PPP |
| sp2,yxy | SPPY.YK and not PPP |
| sp2,vyk | TPVY.SPP[^P] and not PPPP |
| sp2yvp2y | PPTPRPSP |
| sp2,3-misc | PPV[HYQ][^P^H^L^T] and [ST]PP |
|  | and not PPPP\|VYKPP\|PKPP\|PVY.SP\|Y.Y\|PP[YH]EK |
| sp3,yxy | SPPP[^P] and Y.YK and not PPPP |
| sp3,vyk | SPPP and VYK and not PPPP|Y.Y |
| sp4vh | SPPPPVH and not K|Y |
| sp4+,vyk | SPPPP and [VI][YH][KH] and not Y.Y |
| sp4+,yxy | SPPPP and Y.Y and not [^Y]YY[^Y] |
| mtp2 | MTPP[^P] |
| mtp3 | MTPPP |
| tp3a | [TS]P{3,4}[VA]{1,2}[TS].P and not [HY] |
| pqq | PQQP|FSQ and not G |
| gqq | GQQ and Y |
| kpip | KPIP[IK] |
| pelpk1 | PE[ILVM]PK|KPE[ILVM]P and not H |
| pelpk2 | PE[ILVM]PK|KPE[ILVM]P and H |
| pepk | PEPK|KPEP[^P] and not FH{1,2}K and not PE[ILV]P|PVY|P[ND]PK |
| pdpk | P[ND]PK and not PPVY |
| pepk-embed1 | PEPK|KPEP[^P] and FH{1,2}K and not PE[ILV]P|PVY|P[ND]PK |
| pepk-embed2 | KPPVY[TE] and E and not G |
| p2vtl | PP[VIL][TDN][ILV]PP and KP |
| p2vtp | PPIKPPKPPVT |
| p2vyk | PPV[EYH]K and not P.P |
| p2vk | K[AP]PVK and not PPV[EYH]K|KK |
| p2vtv | LP[IVL]PPVTV |
| p2mpav | P[MV]P[SA][IV]|TVPQ |
| p2hek1 | [HK]PPP[EV][HYK]Q |
| p2hek2 | PPV[HY] and PP[YH]EK |
| p3vyk | PPP[VI]YKPP and period>8 |
| p2vpvyk | PP[^P]P[VIL]YK and not SP|EY |
| p2tvk | KPPTP{0,2}VK |
| p2yxpkp2 | PP[HY][^P]PKPP and period>=8 |
| p3ehk1 | KPPPE[HY]KPP |
| p3ehk2 | GEKPLPEHK |

Each regular expression was used to scan TR consensus sequences in PlantPro20 (Table S2) to identify protein sequences with similar TR content. Amino acids enclosed in square brackets represent more than one possible match (e.g. [ST] means either serine or threonine is a match), ‘.’ denotes a wildcard character, ‘^’ preceding an amino acid in closed square brackets denotes ‘NOT’ (e.g. [^P], meaning that proline is excluded), and numbers in closed curly brackets indicate how many times a particular residue must be repeated for a match (e.g. P{2,4} means proline must be found 2-4 times in tandem for a match). Finally, when more than one motif is a match, each motif is separated by ‘|’ and the entire expression is bound by square brackets (e.g. [AP|ST] means either AP or ST is a match). For example, the following regular expression for TR class tp3a, ‘[TS]P{3,4}[VA]{1,2}[TS].P and not [HY]’, should be read as: T or S, followed by 3-4 repeats of P, followed by 1-2 repeats of V or A, followed by T or S, any character followed by P, and never a match of H or Y.
