# Supplementary material for: Global Analysis of Proline-Rich Tandem Repeat Proteins Reveals Broad Phylogenetic Diversity in Plant Secretomes
Source: PLoS One. 2011 Aug 2;6(8):e23167. doi: 10.1371/journal.pone.0023167 (PMC3149072; doi:10.1371/journal.pone.0023167)
Supplement: Table S12 — Documentation for revised master sequences. Several master sequences in the curated sequence list (Text S3) were derived from ESTs representing partial ORFs or ESTs with evidence of a frameshift. We attempted to revise these master sequences based on additional database searching, multiple sequence alignment, or combining reading frames with evidence of a frameshift. Actions taken to “fix” these sequences are given below. The number immediately following some sequence identifiers in Revision(s) denotes the forward reading frame used to revise the master sequence. (DOC) [file pone.0023167.s022.doc]

**Table S12.** **Documentation for revised master sequences.**

| **Master Sequence**  **Identifier** | **TRP**  **Class** | **Revision(s)** |
| --- | --- | --- |
| TA|CF349763 | AGPA | combined with TA473_128735 1 and TA474_128735 2 |
| TA|AJ776538 | EXTA | combine with TA2740_75702 1 |
| TA|CX173034 | EXTA | add N-term from CX167737 1 |
| TA|TA43_57577 | EXTA | add N-term from BB933897 2 |
| TC|TC106825 | EXTA | added N-term from combined with TC100937 2 |
| TC|TC74615 | EXTA | add to N-term using EE433992 3 |
| TC|TC7664 | EXTA | resolve frameshift |
| TC|ES906800 | EXTM | combine with ES913209 3 |
| NR|gi:255582327 | HLTA | truncate 2 N-term aas (MSA inspection) |
| TC|BQ995406 | HLTA | truncate 5 N-term aas (MSA inspection) |
| TA|CB086431 | HLTA | combine with gi|27907446 |
| TA|CO742613 | HLTA | combine with gi|110663997 |
| TA|CV013657 | HLTA | combine with gi|171355185 |
| TA|DT589832 | HLTA | truncate 3 N-term aas (MSA inspection) |
| TA|DY942919 | HLTA | combine with gi|90471486 |
| TA|TA1063_73275 | HLTA | incomplete, N-term homologous to H. petiolaris |
| TA|TA127_121541 | HLTA | S. squalidus 100% overlap |
| TA|TA1304_3415 | HLTA | combine with CK743556 and TA1305_3415 |
| TA|TA241_51953 | HLTA | truncate 2 N-term aas (MSA inspection) |
| TA|TA290_3605 | HLTA | combine with gi|33962640 |
| TA|TA2999_75702 | HLTA | combine with >gi|50065015 |
| TA|TA60_3802 | HLTA | truncate 1 N-term aa (MSA inspection) |
| TA|TA781_94328 | HLTA | truncate 2 N-term aas (MSA inspection) |
| TC|TC300655 | HLTA | combine with gi|57573962 |
| TC|TC4330 | HLTA | combine with CA910618 |
| TA|BE052756 | HLTB | combine with gi|21094713 |
| TA|DV035554 | HLTB | extend N-term using homologue TC8573 |
| TC|FG146646 | HLTB | combine with CK290748 |
| TA|TA1037_3818 | HLTB | combine with gi|56552948 and gi|115596969 |
| TC|TC12252 | HLTB | added N-term Meth using CK290747 |
| TC|TC143442 | HLTB | N-term extended using TC130554 |
| TC|TC31177 | HLTB | combine with gi|63064164 |
| TC|TC8296 | HLTB | combine with gi|15427438 |
| TC|TC8573 | HLTB | extend C-term using DV035554 homologue |
| TC|TC4085 | HLTC | combine with EY174149 |
| TC|CA206116 | HLTE | extend C-term using MSA and homology with CA280282 |
| TA|CD037772 | KPIP | combine with gi|149648155 |
| TC|TC14594 | KPIP | combine with TC8287 |
| TA|CB288675 | PEHK | combine with CB074676 |
| TA|CV093372 | PEHK | combine with CV095392 |
| NR|gi:242050802 | PELPK | truncate 38 N-term aas (MSA inspection) |
| TC|DW136235 | PELPK | sequence has in-frame stop codon (likely EST error) |
| TC|TC136239 | PELPK | combine with gi|260524132 |
| TC|TC4011 | PELPK | resolve frameshift and combine with EY020496 |
| TC|TC84382 | PELPK | truncate using gi|35043958 and gi|35022359 |
| TC|TC9771 | PELPK | resolve frameshift using EY020496 as a reference |
| TA|TA1034_4498 | PEPKA | combine with gi|226992764 |
| NR|gi:226508062 | PEPKB | truncate 1 N-term aa (MSA inspection) |
| TA|TA4766_47247 | PHEK | remove sequence past stop codon |
| TC|TC13275 | PHEK | remove sequence past stop codon |
| TC|TC134887 | PHEK | truncate upstream of premature stop codon |
| TC|TC136784 | PRP | combine with TC125828 and TC121140 |
| TC|TC5422 | PRP | combine TC3293 and TC5422 |

Several master sequences in the curated sequence list (Text S3) were derived from ESTs representing partial ORFs or ESTs with evidence of a frameshift. We attempted to revise these master sequences based on additional database searching, multiple sequence alignment, or combining reading frames with evidence of a frameshift. Actions taken to “fix” these sequences are given below. The number immediately following some sequence identifiers in *Revision(s)* denotes the forward reading frame used to revise the master sequence.
