# Supplementary material for: Global Analysis of Proline-Rich Tandem Repeat Proteins Reveals Broad Phylogenetic Diversity in Plant Secretomes
Source: PLoS One. 2011 Aug 2;6(8):e23167. doi: 10.1371/journal.pone.0023167 (PMC3149072; doi:10.1371/journal.pone.0023167)
Supplement: Text S1 — TR and TRP taxonomies. (DOC) [file pone.0023167.s024.doc]

**Text S1: TR and TRP taxonomies**

To analyze Pro-rich TRPs from plant secretomes, we devised a TR taxonomy as described in the *Materials and Methods*. This taxonomy is presented as a series of three large tables broadly organized by differences in proline backbone architectures: Table S3 summarizes TR classes with SP_n_ and TP_n_ proline architectures, including TRs that compose extensins; Table S4 lists TR classes with simple interspersed P_1_ architectures, such as TRs of the glutamine-rich seed storage proteins and novel ‘KPIP’ and ‘PELPK’ TR motifs; Table S5 contains TR classes that have at least one occurrence of P_2_, including the canonical PRP pentamer motif, P_2_V(Y/E/H)K, and several hybrid PRP classes. For a mapping of all 38 TR classes to their original cluster labels, see Table S10, and for all 38 regular expression class definitions, see Table S11.

Importantly, almost 80% of secreted TRPs (1403/1781 total sequences) corresponding to the TR clusters shown in Figure 1 are captured by the regular expressions that define the taxonomy (see Table S11). (We note that Cluster 9 in Figure 1 is minimally covered by the TR taxonomy. Since this cluster is rich in low-complexity SP_n_-containing TRs, we added a ‘Ser-(Pro)_n_ unclassified’ group to the taxonomy; see Table S3). In addition, the TR taxonomy covers two-thirds (1670/2492) of all secreted Pro-rich TRPs identified from plant secretome data (Table S2).

A second taxonomy was developed for the secreted TRPs composed of Pro-rich TRs catalogued within the TR taxonomy. A total of 31 structural TRP classes were classified and named, as described in *Materials and Methods*, and are presented in a series of three large tables: Ser/Thr-(Pro)_n_ proteins (Table S6), PRPs and hybrid PRPs (Table S7), and all other identified families (Table S8). The TR clusters associated with major TRP classes are shown in Figure 1 and Figure S2. Since a critical aspect of sequence data quality control involves eliminating sequence redundancy, an effort was made to identify unique protein sequences for each TRP class (see *Sequence Redundancy and Revisions* in *Materials and Methods*). We manually curated all full-length and partial sequences within the TRP taxonomy, and identified 912 non-redundant TRPs (907 with predicted signal peptides), all of which are provided in Text S3 (also see PlantPro20Fam online database). To illustrate the modular sequence architectures of the identified TRP classes, representative examples of aligned TR domains are provided in Dataset S1, and partial multiple sequence alignments of selected TRP classes are provided in Dataset S2 and Figure S3. For descriptive details of TRP classes that are either incompletely described or absent from the primary text, see Text S2. Finally, for an interactive online database containing all TR and TRP classes identified in this work, see the PlantPro20Fam online database*.*
