# Supplementary material for: Global Analysis of Proline-Rich Tandem Repeat Proteins Reveals Broad Phylogenetic Diversity in Plant Secretomes
Source: PLoS One. 2011 Aug 2;6(8):e23167. doi: 10.1371/journal.pone.0023167 (PMC3149072; doi:10.1371/journal.pone.0023167)
Supplement: Text S2 — Additional details of plant Pro-rich TRP classes. (DOC) [file pone.0023167.s025.doc]

**Text S2: Additional details of plant Pro-rich TRP classes**

Additional details of the identified Pro-rich TRP classes (and related references) are provided below; phylogenetic ranges are shown in Figure 2B and Table 1 in the primary text.

### **The Extensin Superfamily**

*EXTAs* through *EXTDs*

See primary text.

*EXTMs*

Our analysis identified a number of miscellaneous extensin-like proteins that did not readily fall into distinct subclasses based on the currently available sequence data. In this work, we tentatively refer to this mixed group of extensin-like sequences as "miscellaneous" type extensins, or EXTMs. More explicitly, the EXTM class currently represents outlier sequences that have extensin-like TRs enriched in S/T-P_2/3_ motifs but do not fit into another extensin class. Members of EXTM include hybrid TRPs like Arabidopsis PRP1 and PRP3 [1], and *bona fide* TRPs, like AtEPR1 (see Dataset S1H) [2]. Due to the diversity of EXTMs, reassignment of these sequences remains an important goal of future work.

*HEXAs*

A variety of sequences identified in our analysis have an extensin-like SP_4_ repeat domain, generally a C-terminal SPn,Y domain, and a conserved leucine-rich repeat domain. These proteins, designated Hybrid Extensin type alpha (HEXA), occur in higher angiosperms, and have been previously studied in *Arabidopsis* and rice [3,4]. Unlike typical extensins, the extensin-like repeats found in HEXAs are often widely divergent [3]. One exception, however, is the SP_4_H TR motif that occurs in eudicot HEXAs across eleven species (five full ORFs and six partial sequences in our analysis; see PlantPro20Fam online database), indicating that SP_4_H may play an important role in the eudicot clade of this hybrid extensin family.

**TR-AGPs**

On the basis of published literature and known sequence features, we identified four AGP(-like) TRP classes—three classes with S/T-P_n_ TR motifs, AGPA, AGPB, and AGPC, and one hybrid PRP/AGP family with a conserved cysteine-rich domain, HPOC (see Tables S4-S5). Unexpectedly, only a few previously described *Arabidopsis* AGPs were captured by the TR taxonomy (AtAGP7, AtAGP9, and AtAGP31) out of at least 48 (reviewed in [5]), and perhaps as many as 85 AGP genes [6] in the *Arabidopsis* genome. After analyzing sequence architectures of the AGP sequences absent from the PlantPro20 database (Table S2), we found that the missing AGPs are either non-repetitive, or have very short TR domains that do not satisfy our TR Module definition (see *Materials and Methods*). This result agrees with a previous study showing that AGPs do not have a canonical motif, but are instead comprised of low-complexity sequences generally containing P, A, S, and T residues in various combinations [7].

*AGPAs*

The most highly conserved AGP TRP class in the plant secretome is termed TR-AGP type alpha (AGPA). Most likely representing a “classical AGP” class, AGPAs were found in 57 species and 37 genera from higher plants (Figure 3B and Figure S7), and are defined by TRs enriched in a T/S-P_3_A motif (Table S3 and Dataset S1A). In our analysis, 76% of identified AGPA sequences have a predicted GPI-anchor, including AGPAs present in the major divisions of higher plants: conifers, eudicots, non-grass monocots, and grasses (68 of 89 non-redundant secreted AGPA protein sequences in Text S3). Only two previously reported *Arabidopsis* AGPs are in the AGPA class (AGP7 and AGP9) [7]. Although both *Selaginella* and *Physcomitrella* have AGPA-like sequences with predicted signal peptides (JGI ID: jgi|Selmo1|403392, and TIGR EST ID: TC40462, respectively), neither of these sequences were initially predicted to be GPI anchored (according to the PredGPI web server [8] used for this study). Using another GPI prediction tool, GPI-SOM [9], a putative GPI anchor signal was identified in the *Selaginella* AGPA-like sequence, consistent with a potentially earlier origin of AGPAs in embryophyte evolution. Clearly, additional sequence data will be needed to resolve the origin of AGPAs.

*AGPBs and AGPCs*

Prominent SP_n_ repeats were found in two previously unreported AGP-like classes restricted to higher angiosperms. These protein families, termed TR-AGP type beta (AGPB) and TR-AGP type gamma (AGPC), are specific to eudicots and grasses, respectively (Figure 2B, Table 1), have TRs enriched in methionine, and ~3/4 of the non-redundant proteins identified for each of these families have predicted GPI anchors (AGPB: 9/13 proteins, AGPC: 3/4 proteins). Found in four orders of eudicots and thirteen species, the AGPB family is defined by TRs containing a MTP_3_ motif (Table S3 and Dataset S1B). AGPC sequences are enriched in a MTP_2_ motif (Table S3 and Dataset S1C), were found in four species representing both Triticeae and non-Triticeae grasses, and have a pfam02298, Cu_bind_like, Plastocyanin-like domain.

**PELPKs**

The PELPKs are a large and highly conserved angiosperm TRP class defined by multiple copies of a ‘PELPK’ motif (Table S3 and, e.g., Dataset S1V). Multiple sequence alignment analysis of PELPKs revealed two conserved non-TR sequence features among PELPK proteins, an N-terminal motif [AEGD]ARxL[LE]E located between the C-terminus of the signal peptide and PELPK TR domain, and a C-terminal proline found in >90% of full length PELPK proteins (Dataset S2T). Located 878bp apart on chromosome five, both *Arabidopsis* PELPK loci are probably the product of a tandem gene duplication event. Likewise, PELPK loci identified in the sequenced genomes of rice and *Sorghum bicolor* are arranged in tandem gene clusters, indicating that at least some tandemly arranged PELPK paralogs may have arisen in the early stages of angiosperm evolution. Many flowering plant species have more than one distinct PELPK protein sequence. A minimum of two, and at most four, distinct PELPK protein sequences were found in eudicots with >100K original sequence samples (ESTs and proteins) while in monocots with >100K original sequence samples, between four and eight distinct PELPK proteins were identified (see PlantPro20Fam online database).

**Hybrid PRPs**

Our TRP taxonomy expands the known diversity of hybrid PRPs to a total of nine Hybrid Pro-rich TRP (Hybrid PRP) classes, including six Hybrid LT (HLT) classes and three Hybrid PO (HPO) classes (Table S7). Possibly reflecting distinct structural requirements of higher plant cell walls, all nine identified Hybrid PRP classes are restricted to the secretomes of gymnosperms and/or angiosperms. However, since a simple Pfam query retrieves LT and PO domains in non-Pro-rich proteins of lower plant taxa, the restriction of Hybrid PRP classes to higher plants may reflect sampling bias in the currently available sequence data.

# *HLTAs*

# Among the nine identified Hybrid PRP classes, HLT alpha (HLTA) has the broadest phylogenetic distribution, found in 94 species and 46 genera of non-grass seed-bearing plants (Figure 2B and Figure S9). As shown in Table S5, HLTAs have a Pro-rich TR domain containing variations on the motif, PPVTLPPVVK.

*HPOA*

A second highly conserved Hybrid PRP, called HPO type alpha (HPOA), is defined by a Pro-rich region composed of variations on the motif, PPVPVYKKPLP (Table S5). HPOAs were found in 48 species and 37 genera (including partial sequences, see Figure S10), ranging from primitive to advanced non-grass angiosperms, and have been previously studied in *Arabidopsis* (called AtPRP2 and AtPRP4) [1]. Although no full-length HPOAs were identified from basal angiosperm sequence data, one sequence with an HPOA TR region lacking a PO domain was found in the magnoliid species, *Liriodendron tulipifera* (a basal angiosperm), indicating that the PO domain was acquired sometime between the evolution of basal angiosperms and the common ancestor of basal eudicots and monocots.

*Other Hybrid PRPs*

Consistent with the major cell wall differences of Poaceae compared to most other higher plants, two Hybrid PRP classes, HLTE and HPOB, are uniquely found in the grasses. The other five Hybrid PRP classes (HLTB, HLTC, HLTD, HLTF, HPOC) were found exclusively in eudicot species within the PlantPro20 database (Table S2).

**PRPAs**

Previous work has used 'PRP' as the default name for Pro-rich proteins that are not clearly extensins or AGPs [5,6,10-19]. In this work, PRPAs are defined as TRPs enriched in the canonical PRP motif, P_2_V[YEH]K (Table S5). Full or partial length traditional PRPAs were found in the majority of legume species from the PlantPro20 database (17 of 25 species), as well as in carrot [20]. We also found PRPAs in several *Populus* species, which are closely related to legumes, where the PRPA sequences have the canonical P_2_VYK motif embedded within an 11-mer TR motif, PPVYKPPKIEK. Unexpectedly, the exact PRPA decamer seen in legumes was also identified in two root parasite species, *Triphysaria pusilla* (TIGR EST: TC9426) and *Triphysaria versicolor* (TIGR EST: TC5422), possible indicating (i) a horizontal gene transfer event that may stem from the facultative parasitic relationship between *Triphysaria* and leguminous plant roots (see [21]), (ii) convergent evolution, or (iii) a common ancestor of traditional PRPAs that predates the emergence of core eudicots. Regardless of (i-iii), all thirty-one secreted PRPAs detected in this work have a conserved alanine residue at the C-terminus of the predicted signal peptide and all but one PRPA has N, D, or Q as the N-terminal residue of the predicted mature protein (Dataset S2X; also see PlantPro20Fam online database).

# PRPBs

We also identified a TRP class composed of TRs with a P_3_ variation on the canonical PRP pentamer (see PRPA above). Previously identified in Douglas Fir [22], the corresponding proteins, termed PRP type beta (PRPB), are enriched in a P_3_[VI]YK motif (Table S5), and were found as full length and partial sequences in five conifer species in the PlantPro20 database. Like EXTCs, conifer PRPB sequences terminate in SP_4_, YY, suggesting a potential evolutionary relationship with extensins. (We note that partial PRPB (or PRPB-like) sequences enriched in aspartate are also present in the basal angiosperm, *Amborella trichopoda*; see PlantPro20Fam online database)

**PEPK Classes**

Many TRP and TRP classes targeted to plant secretomes have TRs containing a PEPK motif. Proteins in the PEPK type alpha (PEPKA) TRP class are almost entirely composed of simple ‘PEPK’ TR motifs, and consistent with previous studies (e.g. [10]), PEPKAs are exclusively found in the grasses (Table 1). In addition, most PEPKAs have a C-terminal Asn residue, indicating common ancestry (Dataset S2U). Another class of PEPK-containing TRPs found in grasses has a simple PEPK motif embedded within a much larger C-terminal TR domain (e.g. [18]) (also see Table S4 and Dataset S1Q). Since all proteins in this class also have an N-terminal Pollen Ole e I (PO) domain, they are Hybrid PRPs and are called Hybrid PO type beta (HPOB). A third grass-specific and previously unreported PEPK class that we identified, called PEPK type beta (PEPKB), is characterized by Asp-rich variations on the simple PEPK motif (Table S4 and e.g., Dataset S1X). Finally, a fourth PEPK-containing TRP class, PEPK type gamma (PEPKC), is defined by a simple PEPK motif embedded within a larger PRPA-like TR motif (Table S4 and e.g., Dataset S1Y). Previously reported in the *Gossypium* genus [14,16], we also found a PEPKC sequence in *Theobroma cacao* and in a species from the Rhizophoraceae family, *B. gymnorrhiza*.

**PEHKs**

The PEHKs are a class of previously reported PRP-like TRPs in *Vitis vinifiera* [15,17]. In this study, PEHK TRPs were also identified in *V. hybrid cultivar*, *V. shuttleworthii*, and *V. riparia*. Two distinct, but related, PEHK TR motif classes compose PHEK TRPs (p3ehk1 and p3ehk2; see Table S5), and a phylogenetic analysis of 18 known and predicted PEHK genes from *V. vinifera*, the wine grape species, revealed evidence for a sequence continuum across chromosome five going from PEHKs composed of p3ehk1 TRs to PEHKs composed of p3ehk2 TRs (Figure S3). This result indicates that PEHKs are a product of tandem gene duplication in *V. vinifera*.

**PHEKs and KPIPs**

The PHEKs are a TRP class specific to the legumes and originally identified from cDNA cloning as the Early Nodulin, or ENOD, genes [23] proposed to play important roles in legume-Rhizobium symbiosis [24]. Another TRP class, the KPIPs, previously unreported, is also legume-specific.

**MPAVs**

MPAVs represent a novel grass-specific TRP class. In rice, 4 of 6 of the identified MPAVs are clustered on chromosome 7 (http://rice.plantbiology.msu.edu/). Intriguingly, MPAVs have a conserved N-terminal motif AARxLAD that is similar to the N-terminal motif found in the PELPKs (see *PELPKs*, above).

**SPAPs**

Consistent with previous studies (e.g. [25]), a wide variety of generally high molecular weight, low-complexity SP_n_-containing proteins were found in diverse algal secretomes (see PlantPro20Fam online database). SPAPs are a heterogeneous family of hybrid TRPs with SPAP_2_–containing TR motifs (Table S3 and e.g., Dataset S1AE). Examples of SPAP domain proteins include the reproductive plus and minus agglutinins of *Chlamydomonas* [26].

**QRAs and QRBs**

Glutamine-rich, type alpha (QRA) proteins and Glutamine-rich, type beta (QRB) proteins are the well-studied gliadin/LMW glutenin and HMW glutenin TRPs, respectively, that are restricted to the Triticeae tribe of grasses. These seed storage proteins have been thoroughly reviewed in the literature (e.g. see [27). Surprisingly, a full-length LMW seed storage protein sequence (Genbank id, CV144624) was found in the conifer species, *Pinus taeda*, a distant relative of Triticeae grasses that may have received this gene by a lateral transfer event.

**References**

1. Fowler TJ, Bernhardt C, Tierney ML (1999) Characterization and expression of four proline-rich cell wall protein genes in Arabidopsis encoding two distinct subsets of multiple domain proteins. Plant Physiol 121: 1081-1091.
2. Dubreucq B, Berger N, Vincent E, Boisson M, Pelletier G, et al. (2000) The Arabidopsis AtEPR1 extensin-like gene is specifically expressed in endosperm during seed germination. Plant J 23: 643-652.
3. Baumberger N, Doesseger B, Guyot R, Diet A, Parsons RL, et al. (2003) Whole-genome comparison of leucine-rich repeat extensins in *Arabidopsis* and rice. A conserved family of cell wall proteins form a vegetative and a reproductive clade. Plant Physiol 131: 1313-1326.
4. Ringli C (2010) The hydroxyproline-rich glycoprotein domain of the Arabidopsis LRX1 requires Tyr for function but not for insolubilization in the cell wall. Plant J 63: 662-669.
5. Johnson KL, Jones BJ, Schultz CJ, Bacic A (2003) Non-enzymic cell wall (glyco)proteins. In: Rose JKC, editor. The Plant Cell Wall, Vol. 8. Florida: CRC Press. pp. 111-154.
6. Showalter AM, Keppler B, Lichtenberg J, Gu D, Welch LR (2010) A bioinformatics approach to the identification, classification, and analysis of hydroxyproline-rich glycoproteins. Plant Physiol 153: 485-513.
7. Schultz CJ, Johnson KL, Currie G, Bacic A (2000) The classical arabinogalactan protein gene family of *Arabidopsis*. Plant Cell 12: 1751-1768.
8. Pierleoni A, Martelli PL, Casadio R (2008) PredGPI: a GPI-anchor predictor. BMC Bioinformatics 9: 392.
9. Fankhauser N, Mäser P (2005) Identification of GPI anchor attachment signals by a Kohonen self-organizing map. Bioinformatics 21: 1846-1852.
10. Raines CA, Lloyd JC, Chao S, John UP, Murphy GJP (1991) A novel proline-rich protein from wheat. Plant Mol Biol 16: 663-670.
11. José-Estanyol M, Ruiz-Avila L, Puigdomenech P (1992) A maize embryo-specific gene encodes a proline-rich and hydrophobic protein. Plant Cell 4: 413-423.
12. Deutch CE, Winicov I (1995) Post-trancriptional regulation of a salt-inducible alfalfa gene encoding a putative chimeric proline-rich cell wall protein. Plant Mol Biol 27: 411-418.
13. Goodwin W, Pallas JA, Jenkins GI (1996) Transcripts of a gene encoding a putative cell wall-plasma membrane linker protein are specifically cold-induced in *Brassica napus*. Plant Mol Biol 31: 771-781.
14. Orford SJ, Timmis JN (1997) Abundant mRNA specific to the developing cotton fibre. Theor Appl Genet 94: 909-918.
15. Davies C, Robinson SP (2000) Differential screening indicates a dramatic change in mRNA profiles during grape berry ripening. Cloning and characterization of cDNAs encoding putative cell wall stress response proteins. Plant Physiol 122: 803-812.
16. Tan H, Creech RG, Jenkins JN, Chang Y, Ma D (2001) Cloning and expression analysis of two cotton (*Gossypium hirsutum* L.) genes encoding cell wall proline-rich proteins. DNA Seq 12: 367-380.
17. Thomas P, Lee MM, Schiefelbein J (2003) Molecular identification of proline-rich protein genes induced during root formation in grape (*Vitis vinifera* L.) stem cuttings. Plant Cell Environ 26: 1497-1504.
18. Wang R, Chong K, Wang T (2006) Divergence in spatial expression patterns and in response to stimuli of tandem-repeat paralogues encoding a novel class of proline-rich proteins in *Oryza sativa*. J Exp Bot 57: 2887-2897.
19. Gothandam KM, Nalini E, Karthikeyan S, Shin JS (2010) OsPRP3, a flower specific proline-rich protein of rice, determines extracellular matrix structure of floral organs and its overexpression confers cold-tolerance. Plant Mol Biol 72: 125-135.
20. Chen J, Varner JE (1985) Isolation and characterization of cDNA clones for carrot extensin and a proline-rich 33-kDa protein. Proc Natl Acad Sci USA 82: 4399-4403.
21. Mach J (2010) A shot in the dark: how parasitic plants find host roots. Plant Cell 22: 995.
22. Kieliszewski M, de Zacks R, Leykam JF, Lamport DTA (1992) A repetitive proline-rich protein from the gymnosperm douglas fir is a hydroxyproline-rich glycoprotein. Plant Physiol 98: 919-926.
23. Franssen HJ, Nap JP, Gloudemans T, Stiekema W, Van Dam H, et al. (1987) Characterization of cDNA for nodulin-75 of soybean: A gene product involved in early stages of root nodule development. Proc Natl Acad Sci USA 84: 4495-4499.
24. Brewin NJ (2004) [Plant cell wall remodelling in the rhizobium-legume symbiosis](http://apps.isiknowledge.com/full_record.do?product=UA&search_mode=GeneralSearch&qid=1&SID=4D7A5ocdem4dI8E@Bj6&page=2&doc=20&colname=WOS). CRC Cr Rev Plant Sci 23: 293-316.
25. Waffenschmidt S, Woessner JP, Beer K, Goodenough UW (1993) Isodityrosine cross-linking mediates insolubilization of cell walls in *Chlamydomonas*. Plant Cell 5: 809-820.
26. Lee JH, Waffenschmidt S, Small L, Goodenough U (2007) Between-species analysis of short-repeat modules in cell wall and sex-related hydroxyproline-rich glycoproteins of *Chlamydomonas.* Plant Physiol 144: 1813-1826.
27. [Shewry](http://jxb.oxfordjournals.org/search?author1=Peter+R.+Shewry&sortspec=date&submit=Submit) PR, [Halford](http://jxb.oxfordjournals.org/search?author1=Nigel+G.+Halford&sortspec=date&submit=Submit) NG (2002) Cereal seed storage proteins: structures, properties and role in grain utilization. J Exp Bot 53: 947-958.
