# Supplementary material for: Global Analysis of Proline-Rich Tandem Repeat Proteins Reveals Broad Phylogenetic Diversity in Plant Secretomes
Source: PLoS One. 2011 Aug 2;6(8):e23167. doi: 10.1371/journal.pone.0023167 (PMC3149072; doi:10.1371/journal.pone.0023167)
Supplement: Text S3 — Manually curated list of Pro-rich TRPs. A list of 912 non-redundant TRPs (“master sequences”) catalogued in the TRP taxonomy (Tables S6, S7, S8), 907 of which have a predicted signal peptide [23]. (DOC) [file pone.0023167.s026.doc]

**Text S3: Manually curated list of Pro-rich TRPs.**

A list of 912 non-redundant TRPs (“master sequences”) catalogued in the TRP taxonomy (Tables S6-S8), 907 of which have a predicted signal peptide (SignalP 3.0). (The five TRPs without a predicted signal peptide are three *Arabidopsis* EXTAs, At5g06640, At4g08410, At1g23720, and two *Selaginella moellendorffii* EXTAs, jgi|Selmo1|448871 and jgi|Selmo1|422873). For all TRP motif architectures and the raw sequence data used to build each master sequence, see the PlantPro20Fam website (http://jimcooperlab.mcdb.ucsb.edu/plantpro20fam). Below, TRP classes are each preceded by ‘@’, and all master sequence headers have the following format: master sequence identifier||plant taxa||TRP class. Each ‘master sequence identifier’ is represented by ‘source|identifier’. Abbreviations for ‘source’: TC=The Gene Index Project, TA=TIGR Transcript Assemblies, NR=NCBI NR database, jgi=Joint Genome Institute, LOC=locus, and chr=chromosome. In some cases, we revised the master sequence (as described in *Materials and Methods*). Revisions are indicated by ‘(revision)’, and are documented in detail in Table S12. TRP classes are listed in the following order: EXTA, EXTB, EXTC, EXTD, EXTM, HEXA, AGPA, AGPB, AGPC, PRPA, PRPB, HLTA, HLTB, HLTC, HLTD, HLTE, HLTF, HPOA, HPOB, HPOC, PELPK, PEPKA, PEPKB, PEPKC, KPIP, MPAV, PEHK, PHEK, QRA, QRB, SPAP.

**@ EXTA Class [155 Proteins, 64 Species]**

>TC|TC7664(revision)||Alliaceae_A.cepa||EXTA
MLIHIGDPLRGRLLPTLAAALAILVAYNNVGSVSADPYIYSSPPPPYVYSSPPPPVHSPP
PPYEYKSPPPPKKSPPPPYHYLSPPPPYLYKSPPPPVKSPPPLYMYKSPPPPVKSPPPPY
LYKSPPPPAKSPPPPYMYKSPPPPVKSPPPPYLYKSPPPPVKSPPPSYHYQSPPPPVKSP
PPPYVYHSPPPPVKSPPPPYMYKSPPPPSPSPPPPYVYKSHPPPSTSPSSPYVYKSPPSP
FTSPPPPYHYKSPPP

>TC|TC7670||Alliaceae_A.cepa||EXTA
MVDHRKAWLTPHIVLVVAVCLFATIAESTDKPYIYSSPPPPYHYKSPPPPSPSPPPPYHY
KSPPPPSPSPPLPYHYKSPPPPPSSPPPPYHYKSPPPPSPSPPPLYHYKSPPPPSPSPLP
PYHYKSPPPPSPSPPPPYHYKSPPPPSPSPPPPYHYKSPPPPSPSPPPPYHYKSPPPPSP
SPPPPYHYKSPPPPSPSPPPPYHYKSPPPPSPSPPPPYHYKSPPPPSPSPPPPYHYKSPP
PPSPSPPPLYHYKSPPPPSPSPPPPYHYKSPPPPSPSPPLPYHYKSPPPPYHYKSPPPPS
PSPPPPYHYKSPPPPSPSPPPPYHYKSPPPPSPSPPPPYHYKSPPPPSPSPPPPYHYKSP
PPPSPSPPPPYIYKSPPPPSPSPPPPSHPHHPYYYTSPPPPIY

>NR|gi:119711||Apiaceae_D.carota||EXTA
MGRIARGSKMSSLIVSLLVVLVSLNLASETTAKYTYSSPPPPEHSPPPPEHSPPPPYHYE
SPPPPKHSPPPPTPVYKYKSPPPPMHSPPPPYHFESPPPPKHSPPPPTPVYKYKSPPPPK
HSPAPVHHYKYKSPPPPTPVYKYKSPPPPKHSPAPEHHYKYKSPPPPKHFPAPEHHYKYK
YKSPPPPTPVYKYKSPPPPTPVYKYKSPPPPKHSPAPVHHYKYKSPPPPTPVYKSPPPPE
HSPPPPTPVYKYKSPPPPMHSPPPPTPVYKYKSPPPPMHSPPPPVYSPPPPKHHYSYTSP
PPPHHY

>TA|TA204_51953||Arecaceae_E.guineensis||EXTA
MDRRGRDEASMACLVSTLMVVFVSLSLPLXXQANYHYSPPPSPTPSKEHPYPYKSPPPPP
PHVYYYKSPPPPPPHHVYKYKSPPPPPYHYV

>TA|TA5868_4222||Asteraceae_C.tinctorius||EXTA
MAILLITLCVVVVSLSLPSSTTATYSYSSPPSPSPEKYPPPPTVYKSPPSPPPNKPYDYK
SPPPPPSKKEYEYKSPPPPVPKSLYEYKSPPPPPLGHKSPPRKKPYEHKSPPPLKKPCGH
KSPPPTKNPYEHKSPPPQKNPYAHKSPPLPNKPYEYTSPPPPQKPYEHKSSPPPPKKPYE
HKSSPPPTKKSYEYKSPPPPKKPCEYKTPPPPQKPYEYKSPPPKKPYEYKSPPPPKKTNE
HESSPPPIKKPYEHKSPCPPKKSYEYKSSPPPYLVHKSPSPSPPKKPYEHKSLPPSPKKP
YEY

>TA|EH724883||Asteraceae_C.maculosa||EXTA
MSQMATLLITLSMVIVSLSLPSSTTATYSYSDPPSPPSKKYPPPPIVYKSPPLPSPKKPY
EYKSPPPPSKKEYEYKSPPPPRKSPYEYKSPPPPLVHKSPPQKKPYEHKSPPPQKKPCGH
KSPPPTKKPCEHKSPPPPQKSYEYMSPPPRKPYEHKSSPPPPKKPYEHKSSPPPPKNPCE
YKSPPPPKKPYEHKSSPPPPQKSYKSPPPPKKPYEHKSSPPPPQKSYKSPPPPKKPYVYE
SPPLPPQVHK

>TC|BQ970240||Asteraceae_H.annuus||EXTA
MGTDRKMSSIVTLLATLVVGILSSLPSLTTANYPYSSPPPPPKEHYVYNSPPPPPPKKSP
PPPPPKHPYVYTSPPPPPPKEHYVYKSPPPPPPKEHYVYQSPPPPPPKKSPPPPPKHHYV
YKSPPPPPPKKSTTTTT

>TC|TC59353||Asteraceae_H.annuus||EXTA
MKHSYNTRLMKLYMICLSSVLSFFTTTTFAMPSMSTTRCILHVHHRCIPHVRRLCCTLHV
PHRYILSVPQRCILQCPPPPPPKTHYKYKSPPPPPPKTHYKYKVPTTTSGGETLLVQVPT
TTSAKETLRVQVPTTTSAKETLRVQVTTTTSAKDIL

>TC|TC48945||Asteraceae_H.annuus||EXTA
MRAPVGGGAPFRVRLWPIVIAFTIFGVANLVAVSADKPYLYSSPPPPYEYKSPPPPVHSP
PPPYEYKSPPPPVHSPPPPYVYKSPPPPVHSPPPPYVYKSPPPPVHSPPPPYVYKSPPPP
VHSPPPPYEYKSPPPPVHSPPPPYVYKSPPPPVYSPPPPYVYKSPPPPVHSPPPPYVYKS
PPPPVHSPPPPYEYKSPPPPVHSPPPPYVY

>TA|DW097634||Asteraceae_L.perennis||EXTA
MLCSSYVLLSSFDLILLVDYHYSSPPPPVKSPPPPYHYTSPPPPVKSPPPPYHYSSPPPP
VKSPPPPYHYTSPPPPIKSPPPPYHYTSPPPPVKSPPPPYHYTSPPPPVKSPPPLYHYIS
PPPPG

>TC|DY982350||Asteraceae_L.sativa||sp4+,yyy-containing
MEARSQKVNTSIFIFFFFFFFILLASHLSLAAPTTTWVGSKYQIECTMCAACDNPCDQQP
PPSPPPPQPVYPPPPPPPTSTGGGYYYSPPPPTSGTGGGYYYSPPPPSQGVYYYYPPPAS
YKNYPTSTPPNPIMNYYPYYYYNPPMPNSSAVSLAGATTMLLINMLFICFFSL

>TC|DW135161||Asteraceae_L.sativa||EXTA
MGSLGVLRRWPQLVYAFAFFLLATIIIADKPYAYNSPLPKNFKVTPPVKDPWKKSPYVYK
SPPPVKKTWPHLPYLHKSPPPSYIYKSPPPPSYNYKSPPPPPKYIYKSPPPPSPSPPPPY
VYKSPPPPSPSPPPPYVYKSPPPPSPSPPPPYVYKSPPPPSPSPPPPYVYKSPPPPSPSP
PPPYVYKSPPPPSPSPPPPYVYKSPPPPSPSPPPPYVYKSPPPPSPSPPPPYVYKSPPPP
SPSPPPPYVYKSP

>TA|TA1214_50225||Asteraceae_T.officinale||EXTA
MGTKVKMPQMSTLLVSLALVIISLFLPSFATATYPYSYPPPPKHDYVYKSPPPPPPKKHY
VYKSPPPPHHAHKSPPPPPPKNPYIYKSPPPPPKKHDVYKSPPHHPPKKHYLYKSPPPPP
VHKSPPPPPPKKPYIYKSPPPPPPVHKSPPPPPPKEPYIYKSPPPPPPKKHYVYKSPPPP
PPIHKSPPPTPPKKPYVYKSPPPPPPKKYYVYKSPPPPPPVHKSPPPPPPKKPYIYKSPP
PPSPKKSYGYKSPPPPAPKKSYGYKCPPPSPQPHYLYNSPPPPHYL

>LOC|AT1G20190||A.thaliana||EXTA||EXPANSIN 11 domain (fragment, putative extensin fusion)
MTAALSTALFNDGASCGECYRITCDHAADSRWCLKGASVVITATNFCPPNFALPKKHYVY
KSPPPPVKHYSPPPVYHSPPPPKKHYVYKSPPPPVKHYSPPPVYHSPPPPKKHYVYKSPP
PPVKHYSPPP

>LOC|AT2G43150||A.thaliana||EXTA||EXT21
MATPAWSHAKAQWVVAMLALLVGSAMATEPYYYSSPPPPYEYKSPPPPVKSPPPPYEYKS
PPPPVKSPPPPYYYHSPPPPVKSPPPPYVYSSPPPPVKSPPPPYYYHSPPPPVKSPPPPY
YYHSPPPPVKSPPPPYYYHSPPPPVKSPPPPYYYHSPPPPVKSPPPPYYYHSPPPPVKSP
PPPYLYSSPPPPVKSPPPPVYIYASPPPPTHY

>LOC|AT5G35190||A.thaliana||EXTA||EXT13
MRSPRVMERVHCMVYVVVLSAIAATVTAYPYSSHQTPQYNSPVHKHESSYSPKKYSPYYS
ASPLPPLQYRRQGPKYTPHPKPYLFNSPPPPYYSPSPKEDYKSPPPPYVYNSPPPPYYSP
SPKVDYKSPPPPYVYNSPPPPYYSPSPKVEYKSPPPPYVYNSPPPPYYSLSPKVDYKSPP
PPYVYNSPPPPYYSPSPKVDYKFSPPPYVYNSPSPPYYSPSPKVDYKSPPPPYVYNSPPP
PYFSPSPKVDYKSPPPPYVYSSPPPPPYYSPSPEVSYKSPPPPPYYSPSLEVSYKSPPPL
FVYNFPPPPPFYSPSPKVSYKSPPAPYVSKTPNY

>LOC|AT4G08370||A.thaliana||EXTA||EXT20
MVNPSNWPSLLMLVLAFYVVVVPTSAQCKYSPQSPPPQPYVYSPPLPSPYVYKSPPPSPY
LYSSPPPPPYVYNSPPPPPPYIYNSPPRPPYVYKSPPPPPFVYSSPPPPTYIYNSPPPPP
YVYKSVPRITFIYSSPPPPPYVYNSAPRIPFIYSSPPPPPYVYNSAPRVLFIYSSPPPPP
YVYNSPPPPPYVYESVPRIPFIYSSPPPPPYVYNSAPRIPFIYSSPPPPPYVYNSAPRVP
FIYSSPPPPPYVYKSVPRIPFIYSSPPPPPYVYNSAPRIPFIYSSLPPPPYVYNSAPRVP
FIYSSPPPPPYVYNSAPRIPFIYSSPPPHHMFTSLFLIFHHLQLSTTPSI

>LOC|AT4G13390||A.thaliana||EXTA||EXT12
MISLRMKGLGHCLVYVVVFSVIAAIVTAYDSPSSTPQYTSPYPPKNYSPYLSESPPPPPP
QYRRQEPKYTPHPEPNVYDSPTPLPYYFPFPKLDIKSPPPPSVYTFSPPQLYYSPSPKVE
YKSPPPPYVYSSLPPLTYYSPSPKVIYNSPPPPYIYSSPPPPPYYSPSPKVDYKSPPPPY
VYSSPPPPPYYSPSPKVEYKSPPPPYVYSFPPPPPYYSPSPKVGYKSPPAPYVYSSPPPP
PYYSPSPKVNYKSPPPPYVYSSPPPPPYSPSPKVEFKSPPPPYIYNSPPPPSYYSPSPKI
DYKSPPPPYVYSSPPPPTYYSPSPRVDYKSPPPPYVYNSLPPPYVYNSPPPPPYYSPSPT
VNYKSPPPPYVYNSPPPPPYYSPFPKVEYKSPPPPYIYNSPPPPPYYSPSPKITYKSPPP
PYIYKTPYY

>LOC|AT1G21310||A.thaliana||EXTA||EXT3,EXT5
MGSPMASLVATLLVLTISLTFVSQSTANYFYSSPPPPVKHYTPPVKHYSPPPVYHSPPPP
KKHYEYKSPPPPVKHYSPPPVYHSPPPPKKHYVYKSPPPPVKHYSPPPVYHSPPPPKKHY
VYKSPPPPVKHYSPPPVYHSPPPPKKHYVYKSPPPPVKHYSPPPVYHSPPPPKKHYVYKS
PPPPVKHYSPPPVYHSPPPPKKHYVYKSPPPPVKHYSPPPVYHSPPPPKKHYVYKSPPPP
VKHYSPPPVYHSPPPPKKHYVYKSPPPPVKHYSPPPVYHSPPPPKKHYVYKSPPPPVKHY
SPPPVYHSPPPPKKHYVYKSPPPPVKHYSPPPVYHSPPPPKKHYVYKSPPPPVKHYSPPP
VYHSPPPPKKHYVYKSPPPPVKHYSPPPVYHSPPPPKEKYVYKSPPPPPVHHYSPPHHPY
LYKSPPPPYHY

>LOC|AT4G08380||A.thaliana||EXTA||EXT22
MANPSNWPSLLMVILALYAVAAHTSAQYPYSPPSPPPYVYSSPPPYTYSPPPSPYVYKSP
PYVYSSPPPYAYSPPPSPYVYKSPPYVYSSPPPYAYSPPPSPYVYKSPPYVYSSPPPYAY
SPPPSPYVYKSPPYVYSSPPPYVYSSPPPYAYSPPPYAYSPPPSPYVYKSPPYVYSSPPP
YAYSPPPSPYVYKSPPYVYSSPPPYAYSPPPYAYSPPPSPYVYKSPPYVYSSPPPYAYSP
PPSPYVYKSPPYVYSSPPPYAYSPPPSPYVYKSPPYVYSSPPPYAYSPPPSPYVYKSPPY
VYSSPPPYAYSPPPSPYVYKSPPYVYSSPPPYAYSPPPSPYVYKSPPYVYSSPPPYAYSP
PPSPYVYKSPPYVYSSPPPYTYSPPPYAYSPPPPCPDVYKPPPYVYSSPPPYVYNPPPSS
PPPSPSYSYSSPPPPIY

>LOC|AT5G06630||A.thaliana||EXTA||EXT9
MRSSRTMGLMNCMVYVVVLSAIAATVTSYPYSSPHTPAYDSPSYEHKGPKYAPHPKPYVY
SSPPPPYYTPSPKVNYKSPPPPYVYNSPPPPYYSPSPKVYYKSPPPPYVYSSPPPPYYSP
SPKVYYKSPPPPYVYSSPPPLYYSPSPKVYYKSPPPPYVYSSPPPPYYSPSPKVYYKSPP
PPYVYSSPPPPYYSPSPKVYYKSPPPPYVYSSPPPPYYSPSPKVYYKSPPPPYVYSSPPP
PYYSPSPKVYYKSPPPPYVYSSPPPPYYSPSPKVYYKSPPPPYVYSSPPPPYYSPSPKVY
YKSPPPPYVYSSPPPPYYSPSPKVYYKSPPPPYVYSSPPPPYYSPSPNVYYKSPPPPYVY
SSPPPPYYSPSPKVHYKSPPPPYVYSSPPPPYYSPSPKVHYKSPPPPYVYSSPPPPYYSP
SPKVTYKSPPPPYVYKTPYY

>LOC|AT1G26250||A.thaliana||EXTA||EXT18
MASPNWPSLLMVVLALYSMVAYTSAQYSPTPTPYSPLPPYVYNSPPPYVYNSPSPPPYVY
KPPPYIYSSPPPPPYVYSSPPPPPYVYNSPPPPPYVYSSPPPPPYVYKSPPPPPYVYSSP
PPPPYVYKSPPPPPYVYSSPPPPPYVYSSPPPPPYVYKSPPPPPYVYSPPPPPPYVYQSP
PPPPYVYSSPPPPPYVYKSPPPPPYVYSSPPPPPYVYKSPPPPPYVYSSPPPPPYVYKSP
PPPPYVYSSPPPPPYVYKSPPPPPYVYSSPPPPPYVYKSPPPPPYVYSSPPPPPYVYSSP
PPPPYVYSSPPPPPYVYKSPPPPPYVYTSPPPPPYVYKSPPPPPYVDSYSPPPAPYVYKP
PPYVYKPPPYVYNYSPPPAPYVYKPPPYVYSYSPPPAPYVYKPPPYVYSYSPPPAPYVYK
PPPYVYSSPSPPPYYSSPSPPLY

>LOC|AT1G26240||A.thaliana||EXTA||EXT19
MANPNGWPSLLMLVIALYSVSAHTSAQYTYSPPSPPSYVYKPPTHIYSSPPPPPYVYSSP
PPPPYIYKSPPPPPYVYSSPPPPPYIYKSPPPPPYVYSSPPPPPYIYKSPPPPPYVYSSP
PPPPYVYKSPPPPPYVYNSPPPPPYVYKSPPPPPYVYSSPPPPPYVYKSPPPPPYVYSSP
PPPPYVYKSPPPPPYVYSSPPPPPYVYKSPPPPPYVYSSPPPPPYVYKSPPPPPYVYSSP
PPPPYVYKSPPPPPYVYSSPPPPPYVYKSPPPPPYVYSSPPPPPYVYKSPPPPPYVYSSP
PPPPYVYKSPPPPPYVYSSPPPPPYVYKSPPPPPYVYNSPPPPPYVYKSPPPPPYVYSSP
PPSPYVYKSPPPPPYVYSSPPPPPYVYKSPPPPPYVYSSPPPPPYVYKSPPPPPYVYSSP
PPPPYVYKSPPPPPYVYSSPPPPPYVYKSPSPPPYVYKSPPPPPSYSYSYSSPPPPIY

>LOC|AT4G08400||A.thaliana||EXTA||EXT7
MGVIRTSRTMGLGQCMVYVVVLSAIAATVTSYPYTSPQTPHYNSPSHEHKIPKYTPHPKP
SIYSSSPPPSYYSPSPKVDYKSPPPSYVYSSPPPPYYSPSPKVDYKSLPPPYVYSSPPPP
YYSPSPKVNYKSPPPPYVYSSPPPPYYSPSPKGDYKSPPPPYVYSSPPPPYYSPSPKVDY
KSPPPPYVYSSPPPPYYSPTPKVDYKSPPPPYVYSSPPPPYYSPSPKVDYKSPPPPYVYS
SPPPPYYSPSPKVNYKSPPPPYYRSPPPPYYSPSPKVDYKSPPPPYVYSSPPPPYYSPSP
KVDYKSPPPPYVYSSPPPPYYSPTPKVDYKSPPPPYVYSSPPPPYYSPSPKVDYKSPPPP
YVYSSPPPPYYSPSPKVDYKSPPPPYVYNSPPPPYYSPSPKVDYKSPPPPYIYNSPPPPY
YSPSPKVNYKTPPPPYVYSSPPPPYYSPSPKVNYKSPPPPYVYSSPPPPYYSPSPNVDYK
SPPPPYVYSSPPTPYYSPSSKVTYKSPPPPYVY

>LOC|AT2G24980||A.thaliana||EXTA||EXT6
MGIENCIVYVVVLSAIAATVTSYPYSSPQTPSYNSPSYEHKGPKYAPHPKPYVKSSPPPQ
YYTPSPKVNYKSPPPPYVYSSPPPPYYSPSPKVDYKSPPPPYVYSSPPPPYYSPSPKVDY
KSPPPPYVYSSPPPPYYSPSPKVDYKSPPPPYVYNSPPPPYYSPSPKVDYKSPPPPYVYS
SPPPPYYSPSPKVYYKSPPPPYVYSSPPPPYYSPSPKVYYKSPPPPYVYSSPPPPYYSPS
PKVYYKSPPPPYVYSSPPPPYYSPSPKVYYKSPPPPYVYSSPPPPYYSPSPKVYYKSPPP
PYVYSSPPPPYYSPSPKVYYKSPPPPYVYSSPPPPYYSPSPKVYYKSPPPPYVYSSPPPP
YYSPSPKVYYKSPPPPYVYSSPPPPYYSPSPKVYYKSPPPPYVYNSPPPPYYSPSPKVYY
KSPPPPYVYSSPPPPYYSPSPKVYYKSPPPPYVYSSPPPPYYSPSPKVYYKSPPPSYVYS
SPPPPYYSPSPKVYYKSPPPSYVYSSPPPPYYSPSPKVYYKSPPPPYVYSSPPPPYYSPS
PKVTYKSPPPPYVYKTPYY

>LOC|AT5G49080||A.thaliana||EXTA||EXT11
MGLRQFLVYVVVLSAIAATVTSYPYSSPQTPQYNFPSHQHKSPKYTPHSKPYIYNSPPPP
YYSPSPKVNYKSPPPPYVYSSPPPPYYTPSPKVDYKSPPPPYEYSSPPPPYYSPSPKIDY
KSPPPPYVYSSPPLPYYSPSPKVDYKSPPPPYVYSSPPPPYYSPTPKVDYKSPPPPYVYS
SPPPPYYSPSPKVDYKSPPPPYVYSSPPPPYYSPSPKVDYKSPPPPYVYSSPPPPYYSPT
PKVDYKSPPPPYVYSSPPPPYYSPSPKVDYKSPPLPYVYSSPPPPYYSPSPKVDYKSPPP
PYVYSSPPPPYYSPSPKVDYKSPPPPYVYSSPPPPYYSPSPKVDYKSPPPPYVYSSPPPP
YYSPTPKVDYKSPPPPYVYSSPPPPYYSPSPKVDYKSPPPPYVYSSPPPPYYSPSPKVDY
KSPPPPYVYSSPPPPYYSPSPKVDYKSPPPPYVYNSPPPPYYSPSPKVDYKSPPPPYVYS
SPPPPYYSPSPKVDYKSPPPPYVYSSPPPPYYSPSPKVDYKSPPPPYVYSSPPPPYYSPS
PKVDYKSPPPPYVYNSPPPPYYSPSPKVDYKSPPPPYVYSSPPPPYYSPSPKVTYKSLPP
PYVYKAPYY

>LOC|AT5G06640||A.thaliana||EXTA||EXT10
MFKYNIKPKQINKNKEMGMMRSSRTMGLGHCMVYVVVLSAIAATVTSYPYSSPYSSPQTP
HYNSPSHEHKSPKYAPHPKPYVYISPPPPSYYSPSPKVNYKSPPPPNVYNSPPPPYYSPS
PKVDYKSPPPPYVYSSPPPPYYSPSPKVDYKSPPPPYVYSSPPPPYYSPSPKVEYKSPPP
PYVYNSPPPPYYSPSPKIEYKSPPPPYVYSSPPPPYYSPSPKVDYKSPPPPYVYNSPPPP
YYSPSPKVDYKSPPPPYVYSSPPPPYFSPSPKVEYKSPPPPYVYNSPPPPYYSPSPKVEY
KSPPPPYVYSSPPPPYYSPSPKVYYKSPPPPYVYSSPPPPYYSPSPKVDYKSPPPPYVYS
SPPPPYYSPSPKVDYKSPPPPYVYSSPPPQYYSPSPKVAYKSPPPPYVYSSPPPPYYSPS
PKVAYKSPPPPYVYSSPPPPYYSPSPKVDYKSPPPPYVYSSPPPPYYSPSPKVEYKSPPP
PYVYSSPPPPYYSPSPKVEYKSPPPPYVYSSPPPPYHSPSPKVNYKSPPPPYVYSSHPPP
YYSPSPKVNYKSPPPPYVYSSPPPPYYSPSPKVNYKSPPPPYVYSSPPPPYYSPSPMVDY
KSTPPPYVYSFPPLPYYSPSPKVDYKSPPLPYVYSSPPPLYYSPSPKVHYKSPPPPYVYN
SPPPPYYSPSPKVTYKSPPPPYVYKAPYY

>LOC|AT4G08410||A.thaliana||EXTA||EXT8
MFMIKYNIKPKQRKKIVAMGVIRTSRTMGLGQCMVYVVVLSAIAATVTSYPYTSPQTPHY
NSPSHEHKIPKYTPHPKPSIYSSSPPPSYYSPSPKVDYKSPPPSYVYSSPPPPYYSPSPK
VDYKSLPPPYVYSSPPPPYYSPSPKVNYKSPPPPYVYSSPPPPYYSPSPKGDYKSPPPPY
VYSSPPPPYYSPSPKVDYKSPPPPYVYSSPPPPYYSPTPKVDYKSPPPPYVYSSPPPPYY
SPSPKVDYKSPPPPYVYSSPPPPYYSPSPKVNYKSPPPPYVYGSPPPPYYSPSPKVDYKS
PPPPYVYSSPPPPYYSPSPKVNYKSPPPPYVYGSPPPPYYSPSPKVDYKSPPPPYVYSSP
PPPYYSPSPKVDYKSPPPPYVYSSTPPPYYSPSPKVDYKSPPPPYVYSSPPPPYYSPSPK
VDYKSPPPPYIYSSTPLPYYSPSPKVDYKSPPPPYVYSSPPPPYYSPSPKVDYKPPPPPY
VYSSPPPPYYSPSPKVDYKSPPPPYVYSFPPPPYYSPSPKVDYKSPPPPYVYSSPPPPYY
SPSPKVNYKSPPPPYVYSSPPPPYYSPSPKVEYKSPPPPYIYSSPPPPYYAPSPKVDYKS
PPPPYVYSSPPPPYYSPSPKVDYKSPPPPYVYSSPPPPYYSPSPKVNYKSPPPPYVYSSP
PPPYYSPSPKVDYKSSPPQYVYSSPPTPYYSPSPKVTYKSPPPPYVY

>LOC|AT3G54590||A.thaliana||EXTA||EXT2
MGPSAHLISALGVIIMATMVAAYEPETYASPPPLYSSPLPEVEYKTPPLPYVDSSPPPTY
TPAPEVEYKSPPPPYVYSSPPPPTYSPSPKVDYKSPPPPYVYSSPPPPYYSPSPKVDYKS
PPPPYVYNSPPPPYYSPSPKVDYKSPPPPYVYSSPPPPYYSPSPKVEYKSPPPPYVYSSP
PPPYYSPSPKVDYKSPPPPYVYSSPPPPYYSPSPKVEYKSPPPPYVYSSPPPPYYSPSPK
VDYKSPPPPYVYSSPPPPYYSPSPKVDYKSPPPPYVYSSPPPPYYSPSPKVDYKSPPPPY
VYSSPPPPYYSPSPKVDYKSPPPPYVYSSPPPPYYSPSPKVDYKSPPPPYVYSSPPPPTY
SPSPKVDYKSPPPPYVYSSPPPPYYSPSPKVEYKSPPPPYVYSSPPPPTYSPSPKVYYKS
PPPPYVYSSPPPPYYSPSPKVYYKSPPPPYVYSSPPPPYYSPSPKVYYKSPPPPYVYSSP
PPPYYSPSPKVYYKSPPPPYVYSSPPPPYYSPSPKVYYKSPPPPYVYSSPPPPYYSPSPK
VHYKSPPPPYVYSSPPPPYYSPSPKVHYKSPPPPYVYNSPPPPYYSPSPKVYYKSPPPPY
VYSSPPPPYYSPSPKVYYKSPPPPYVYSSPPPPYYSPSPKVYYKSPPPPYYSPSPKVYYK
SPPHPHVCVCPPPPPCYSPSPKVVYKSPPPPYVYNSPPPPYYSPSPKVYYKSPPPPSYYS
PSPKVEYKSPPPPSYSPSPKTEY

>LOC|AT1G23720||A.thaliana||EXTA||EXT15
MVAASYEPYTYSSPPPPLYDSPTPKVDYKSPPPPYVYSSPPPPLSYSPSPKVDYKSPPPP
YVYSSPPPPYYSPSPKVEYKSPPPPYVYSSPPPPYYSPSPKVDYKSPPPPYVYSSPPPPY
YSPSPKPTYKSPPPPYVYNSPPPPYYSPSPKVEYKSPPPPYVYSSPPPPYYSPSPKVDYK
SPPPPYVYNSPPPPYYSPSPKPTYKSPPPPYIYSSPPPPYYSPSPKPVYKSPPPPYVYSS
PPPPYYSPSPKPAYKSPPPPYVYSSPPPPYYSPSPKPIYKSPPPPYVYNSPPPPYYSPSP
KPAYKSPPPPYVYSFPPPPYYSPSPKPVYKSPPPPYVYNSPPPPYYSPSPKPAYKSPPPP
YVYSSPPPPYYSPSPKPTYKSPPPPYVYSSPPPPYYSPSPKPVYKSPPPPYIYNSPPPPY
YSPSPKPSYKSPPPPYVYSSPPPPYYSPSPKLTYKSSPPPYVYSSPPPPYYSPSPKVVYK
SPPPPYVYSSPPPPYYSPSPKPSYKSPPPPYVYNSPPPPYYSPSPKVIYKSPPHPHVCVC
PPPPPCYSHSPKIEYKSPPTPYVYHSPPPPPYYSPSPKPAYKSSPPPYVYSSPPPPYYSP
APKPVYKSPPPPYVYNSPPPPYYSPSPKPTYKSPPPPYVYSSPPPPYYSPTPKPTYKSPP
PPYVYSSPPPPYYSPSPKPTYKSPPPPYVYSSPPPPYYSPAPKPTYKSPPPPYVYSSPPP
PYYSPSPKPTYKSPPPPYVYSSPPPPPYYSPSPKVEYKSPPPPYVYSSPPPPYYSPSPKV
EYKSPPPPYVYSSPPPPPYYSPSPKVEYKSPPPPYVYSSPPPPTYYSPSPKVEYKSPPPP
YVYNSPPPPAYYSPSPKIEYKSPPPPYVYSSPPPPSYSPSPKAEYKSPPPPSLYY

>LOC|AT3G54580||A.thaliana||EXTA||EXT17
MGPSTHLICALGVVIMATMVAAYDPYTDSSPPPLYSSPLPKIEYKTPPLPYIDSSPPPTY
SPAPEVEYKSPPPPYVYSSPPPPTYSPSPKVEYKSPPPPYVYSSPPPPTYSPSPKVEYKS
PPPPYVYSSPPPPTYSPSPKVEYKSPPPPYVYSSPPPPTYSPSPKVEYKSPPPPYVYSSP
PPPTYSPSPKVEYKSPPPPYVYSSPPPPYYSPSPKVEYKSPPPPYVYSSPPPPTYSPSPK
VDYKSPPPPYVYSSPPPPYYSPSPKVEYKSPPPPYVYSSPPPPTYSPSPKVDYKSPPPPY
VYSSPPPPYYSPSPKVDYKSPPPPYVYSSPPPPTYSPSPKVEYKSPPPPYVYSSPPPPTY
SPSPKVEYKSPPPPYVYSSPPPPTYSPSPKVEYKSPPPPYVYSSPPPPTYSPSPKVEYKS
PPPPYVYSSPPPPYYSPSPKVEYKSPPPPYVYSSPPPPTYSPSPKVDYKSPPPPYVYSSP
PPPYYSPSPKVYYKSPPPPYVYSSPPPPYYSPSPKVYYKSPPPPYVYSSPPPPYYSPSPK
VYYKSPPPPYYSPSPKVYYKSPPPPYVYSSPPPPYYSPSPKVYYKSPPPPYVYSSPPPPY
HSPSPKVQYKSPPPPYVYSSPPPPYYSPSPKVYYKSPPPPYVYSSPPPPYYSPSPKVYYK
SPPPPYVYSSPPPPYYSPSPKVYYKSPPHPHVCVCPPPPPCYSPSPKVVYKSPPPPYVYS
SPPPPHYSPSPKVYYKSPPPPYVYSSPPPPYYSPSPKVHYKSPPPPYYAPTPKVHYKSPP
PPYVYSSPPPPYYSPSPKVHYKSPPPPYVYSSPPPPYYSPSPKVEYKSPPPPYVYSSPPP
PYYSPSPKVDYKSPPPPYVYSSPPPPYYSPSPKVDYKSPPPPYVYSSPPPPYYSPSPVVD
YKSPPPPYVYSSPPPPYYSPSPKVEYKSPPPPYVYKSPPPPSYSPSPKTEY

>LOC|AT3G28550||A.thaliana||EXTA||EXT16
MGPSAHLVYAIGVIIMATMVAAYEPYTDSSPPPYSVPLPKVEYKSPPLPDVYSSPPPPLE
YSPAPKVDYKSPPPPYYSPSPKVEYKSPPPPYVYNSPPPPYYSPSPKVDYKSPPPPYVYS
SPPPPIYSPSPKVDYKSPPPPYVYSSPPPPYYSPSPKVEYKSPPSPYVYNSPPPSYYSPS
PKVDYKSPPPPYVYSSPPPPYYSPSPKVVYKSPPPPYVYSSPPPPYYSPSPKVDYKSPPP
PYVYSSPPPPYYSPSPKIVYKSPPPPYVYSSPPPPYYSPSPKVDYKSPPPPYVYSSPPPP
YYSPSPKVVYKSPPPPYVYSSPPPPYYSPTPKVDYKSPPPPYVYSSPPPPYYSPSPKVDY
KSPPPPYVYSSPPPPYYSPSPKIVYKSPPPPYVYSSPPPPYYTPSPKVVYKSPPPPYVYS
SPPPPYYSPSPKVDYKSPPPPYVYSSPPPPYYSPSPKVVYKSPPPPYVYSSPPPPYYSPS
PKVDYKSPPPPYVYSSPPPPYYSPSPKVLYKSPPPPYVYSSPPPPYYSPSPKVVYKSPPP
PYVYSSPPPPYYSPSPKVVYKSPPPPYVYSSPPPPYYSPSPKVVYKSPPPPYVYSSPPPP
YYSPSPKVYYKSPPSPYHAPSPKVLYKSPPHPHVCVCPPPPPCYSPSPKVVYKSSPPPYV
YSSPPPPYHSPSPKVHYKSPPPPYVYSSPPPPYYSPSPKVHYKSPPPPYVYSSPPPPYYS
PSPKVVYKSPPPPYVYSSPPPPYYSPSPKVVYKSPPPPYVYSSPPPPYYSPSPKVVYKSP
PPPYVYSSPPPPYYSPSPKVVYKSPPPPYVYSSPPPPYYSPSPKVVYKSPPPPYVYSSPP
PPYYSPSPKVVYKSPPPPYVYSSPPPPYYSPSPKVDYKSPPPPYVYSSPPPPYYSPSPKV
DYKSPPPPYVYSSPPPPYYSPSPKVDYKSPPPPYVYSSPPPPYYSPAPKVDYKSPPPPYV
YSSPPPPYYSPSPKVDYKSPPPPYYSPSPKVDYKSPPPPYVYSSPPPPSYSPSPKTEY

>TC|EE431728||Brassicaceae_B.napus||EXTA
MRSPKIMGLGHCMVYVVVFSAIVAAAYPYESPPTTQEYPPVHKTKSPYPPKKNSPYYSAP
PSPPPQYRRQGPKYTPHPKPYIYSSPPPPSYYSPSPKVEYKSPPPPYVYSSPPPPPYYSP
SPKVDYKSPPPPYVYSSPPPPPYYSPSPKVEYKSPPPPYVYNSPPPPPYYSPSPKVEYKS
PPPPYVYSSPPPPPYYSPSPKV

>TC|EV126638||Brassicaceae_B.napus||EXTA
MATPAWSHARGQWVIAMLALLVGSAIATEPYYYSSPPPPYEYKSPPPPVKSPPPPYEYKS
PPPPVKSPPPPYYYHSPPPPVKSPPPPYVYQSPPPPVKSPPPPYYYHSPPPPVKSPPPPY
VYNSPPPPVKSPPPPYYYHSPPPPVKSPPPPYYYHSPPPPVKSPPPPYYYHSPPPPVKSP
PPPYYYHSPPPPVKSPPPPYYYHSPPPTHY

>TC|ES907651||Brassicaceae_B.napus||EXTA
MRPSSRMGPSAHITYALGVIIMATMVAAYEPYSLPPLPSYSPSPKVEYNTPPLPYIHSSS
PPPPLYYSPSPKVDYKSPPPPYVYSSPPPPYYSPSPKVDYKSPPPPYVYSSPPPPPYYSP
SPKVEYKSSPPPYVYISPPPPPYYSPSPKVDYKSPPPPYVYSSPPPPSYYSPSPKVEYKS
PPPPYVYSSPPPPPYYSPSPKVDYKSPPPPYVYSSPPPYVYSSPPPPYYSPSPKVEYKSP
PPPATYY

>NR|gi:22001410||Brassicaceae_B.napus||EXTA
MGSPVASFAASLLVLTISPLTSVYQSTANYFYSSPPPPVKHYEYKSPPPPVMHYSLPQVY
HSPPPPKKHTVNKSPPPPVKQYSPPWLRSPPPPRKDYVYKSPPPPVKQYSSPPPNKYYVY
QSPPPPVKHYSPPSVYHSPPPPKNQYVYKSPPPPVKHYTPPVYHSGPPPKKHYMYKSPPP
PVMHYSLPQVYHSPPPPKKHYVYKSPPPPVKHYSPRLVYHSPPPPKKKYVYKSPPPPVRH
YFPPHHLYLYKSPPPPYHY

>TC|TC75747||Brassicaceae_B.napus||EXTA
MAYLAATLLVLTVSLTFVSQSTANYFYSSPPPPVKHYEYKSPPPPVKHYPPPPVYHSPPP
PKKHYEYKSPPPPVYHSPPPPKKHYVYKSPPPPVKHYSPPPVYHSPPPPKKDYVYKSPPP
VKHYSPPPVYHSPPPPKKHYVYKSPPPPVKHYTPPPVYHSPPPPKKHYVYKSPPPPVMHY
SPPPVYHSPPPPKKHYVYKSPPPPVKHYPPPVYHSPPPPKKHYVYKSPPPPVKHYSPPPV
YHSPPPPKKHYVYKSPPPPVKHYSPPPVYHSPPPPKEKYVYKSPPPPPVHHYSPPHHPYL
YKSPPPPYHY

>TC|TC74615(revision)||Brassicaceae_B.napus||EXTA
MGSPMASLAATLLVLALSLGFVSETTANYYYSSPPPPVKHYTPPVYKSPPPPVKHYSPPV
YKSPPPPKKDYEYKSPPPPVKHYSPPPVYKSPPPPKKHYEYKSPPPPVYKSPPPPVYHSP
PPPKKHYEYKSPPPPVYKSPPPPVYHSPPPPKKHYEYKSPPPPVYKSPPPPVYHSPPPPK
KHYEYKSPPPPVYKSPPPPVYHSPPPPKKHYEYKSPPPPVYKSPPPPVYHSPPPPKKHYE
YKSPPPPVYKSPPPPVYHSPPPPKKHYEYKSPPPPVYKSPPPPVYHSPPPPKKHYEYKSP
PPPVYQSPPPPVYHSPPPPKKHYEYKSPPPPVYSPPPPVHYSPPHHPYLYKSPPPPYHY

>TC|TC93179||Brassicaceae_B.napus||EXTA
MANPNWPSLLMLVLALFTIVVHSSAQYSPPSPPPYAYSSPWLPPYVYKSPPLPPYVYNSP
PPPPYVYNSPPPPPYVYSSPPPPPYVYKSPPPPPYVYSSPPPPPYVYKSPPPPPYVYSSP
PPPPYVYKSPPPPPYVYPSPPPPPYVYKSPPPPPYVYSSPPPPPYVYKSPPPPPYVYSSP
PPPPYVYKSPPPPPYVYSSPPPPPYVYNSPPPPYVYSSPPPPPYVYKSPPPPPYVYKSPP
PPPYVYSSPPPPPYVYKSPPPPPYVYSSPPPPPYVYKSPPPPPYVYSSPPPPPYIYKSPP
PPPYVYSSPPPPPYVYKSPPPPPYVYSSPPPPPYVYKSPPPPPYVYSSPPPPPYVYKSPP
PPPYVYSSPPPPSYIYSSPPPPSYSYSYSSPPPPIY

>TA|AM391405||Brassicaceae_B.oleracea||EXTA
MATPACSHARGQWVVAILALLVGSAIATEPYIYSSPPPPYVYKSPPPPVASPPPPYEYKS
PPPPVKSPPPPYEYKSPPPPVKSPPPPYYYHSPPPPVKSPPPPYVYHSPPPPVKSPPPPY
YYHSPPPPVKSPPPPYVYHSPPPPVKSPPPPYYYHSPPPPV

>TA|TA1916_3712||Brassicaceae_B.oleracea||EXTA
MGSPMASLAATLLVLALSLGFVTETTANYYYSSPPPPVKHYTPPVYKSPPPPVKHYSPPV
YKSPPPPKKDYEYKSPPPPVKHYSPPPVYKSPPPPKKHYEYKSPPPPVYKSPPPPVYHSP
PPPKKHYEYKSPPPPVYKSPPPPVYHSPPPPKKHYEYKSPPPPVYKSPPPPVYHSPPPPK
KHYEYKSPPPPVYKSPPPPVYHSPPPPKKHYEYKSPPPPVYQSPPPPVYHSPPPPKKHYE
YKSPPPPVYSPPPPVHYSPPHHPYLYKSPPPPYHY

>NR|gi:89257638||Brassicaceae_B.oleracea||EXTA
MGLGHCMVYVVVFSAIVAAAYPYESPPTTQKYPPVHKTKSPYPPKMNSPYYSAPPSPPPQ
YRRQGPKYTPHPKPYIYSSPPPSSYYSPSPKVEYKSPPPPYVYSSPPPPPYYSPSPKVDY
KSPPPPYVYSSPPPPPYYSPSPKVEYKSPPPPYVYNSPPPPPYYSPSPKAEYKSPPPPYV
YSSPPPPPYYSPSPKVEYKSPQPPYVYSSPPPPPYYSPSPKVDYKSPPPPYVYSSPPPPP
YYSPSPKVDYKSPPPPYVCSSPPPPPYYSPSPKVDYKSPPPPPPYYSPSPKFEYKSPPPP
YVYSSPPPPPYYSPSPKVEYKSPPPPYVYNSPPPPPYYSPSPKVDYKSPPPPYVYSSPPP
PPYYSPSPKVYYKSPPPPYVYSSPPPPPYYSPSPKMVYKSPPPPYVYSSPPPPPYYSPSP
KVNYKSPPPPYVYSSPPPPPYYSPSPKVNYKSPPPPYVHSSPPPPPFYSPSPKAEYKSPP
PPYVYSSPPPPPYYSPSPKVDYKSPPPPYVYSSPPPPPYYSPSPKVYYKSPPPPPYVYKM
PYY

>TA|DN777975||Brassicaceae_T.salsuginea||EXTA
MGSPMASLAATLLVLAFSLCFVSETTANYYYSSPPPPVKHYTPPVYKSPPPPVKHYSPPP
VYKSPPPPKKHYEYKSPPPPKKDYEYKSPPPPVKHYSPPPVYKSPPPPKKDYEYKSPPPP
VKHYSPPPVYKSPPPPKKHYEYKSPPPPVYKSPPPPVYHSPPPPKKHYEYKSPPPPVYQS
PPPLV

>TA|CO037051||Casuarinaceae_C.glauca||EXTA
MANPWPQLAYVVAVLLLATNVAADYEPYTYESPPPPPYMYKSPPPPYMYKSPPPPYMYKS
PPPPYMYKSPPPPPPSPPPPYVYKS

>TC|TC9988||Convolvulaceae_I.nil||sp4+,yvy-containing
MLASVATMESLMATILVTLLSLAAYSSLCSAHSEYSSPKHPAWFQEPPELPPLPEHPYPK
HPPVYNYNSPPPPPPVYHYSSPPPPSPPVYKSPPAEPYKPPCVCHPSPRPPPPHPVYWSP
PPHNPYVYKSPPPPVNPYVYKSPPPPPVNPYVYKSPPPPPPIY

>TA|BW992988||Cupressaceae_C.japonica||EXTA
MELSTPARGALVFCKIFFLFVLSILCFAIQSSATYGHPKPPYAYISPPPPSPIYKPPVIP
PPVIKPPPPHTISPVLPPPIVKPPPKPKPPPHHISPIYPPPVVKPPPVIPPPIIKPPPKP
KPPPYVYKSPPPPHKVSPVYPPPKSPPKPTPKPPPVIPPPIVKPPPLIYPPPYLYKSPPP
PYHKISPVYPPPKSPHLPKPKPPI

>TA|TA945_58031||Cycadaceae_C.rumphii||EXTA
MVAPMGTLCKFFLLALIASICVANGSSASYHNAPAPKPYIYKSPPPPSPVYKPPTPPSSP
SPVYHKPPYAYTSPPPPVYKAAPIPKPPIVSRPPPVHKPPPYIYKSPPPPPYHKVSPIYK
PPQSPYHKPSPTYHHHKPSPVVYAPPKSPPPSSTPYLYKSPPPPYHQPAPVYKPPYHKPS
PVYKPPTHPIYKPPTTPSPSPPYYKPLPPVHPAPPVYKPPTPSSPPPKSYLYKSPPPPTA
SPPPPTHYYYKSPPPPIY

>TA|CK649576||Euphorbiaceae_M.esculenta||EXTA
MGSPMASIIATILVATLSLSFPSETSANYPYHSPSPPKKSPLPPVHPPSYHYKSPPPPVY
SPPPSPPYKYKSPPPPPPVYKYKSPPPPPPVYSPPPPKKPSVKISSSL

>NR|gi:255544846||Euphorbiaceae_R.communis||EXTA
MSPKMANLVYYALAMCLIANIVAAEYQPYYSSPPPLPYKYMSPPPPSPSPPPPYYYQSPP
PPSPSPPPPYYYKSPPPPSPSPPPPPSPSPPPPYYYKSPPPPSLSPHPLTTINDTPMLRM
ASETEARGIKAILDQYSSNHELGD

>NR|gi:255576353||Euphorbiaceae_R.communis||EXTA
MGAWRQPGNLSSMIHVVVLCLVATTAVADLKYPSYEYKLPPPHKKVYYPPYYYKSPPPPS
PSPPPPYYYTSPPPRKKYPSPSPLLPYHYHSPPPPKKSTHPTYYYNSPPPPYYYNSPPPP
SSSPPPLYYYDSPPPPKKSLSPPYHYQSPSPLSPSPPPPYYYASPSPTPKKSPSPLSYYK
SPPPPSLSPPLSYYYQSLPPPNYFSPPPYY

>TC|TC323844||Fabaceae_G.max||EXTA
MQLPKYIAASFFCLICSSIIYYGKAEAHQVNFYVSPPPPSPSPPPPYHYTSPPPPSPAPA
PKYIYKSPPPPVYIYASPPPPIYK

>NR|gi:255645675||Fabaceae_G.max||EXTA
MGTRQWPRLIIALAFCLMSMSVAADDKPYYGQPSNYYPHPTPPTYRQINPPYYYKSPPYY
YKSPPPPSPSPPPPYVYKSPPPPSPSPPPPYIYKSPPPPSPSPPPPSPYVYKSPPPPSPS
PSPPPSHSPPPPHHPYLYNSPPPPVY

>TC|FK018532||Fabaceae_G.max||EXTA
MTVGSYDPPRRGHLGPQMAIALLIVLISFNVGSVAGDAYIYSSPPPPPYEYKSPPPPSPS
PPPPYYYKSPPPSPSPHPHPPYYYKSPPPPPPTYYYKSPPPPSYYYKSPPPPSPSPPPYY
YKSPPPPSPSPPSPYYYKSPPPTKSPPPPVYIYASPPPPIYK

>TC|TC301541||Fabaceae_G.max||EXTA
MGSLMAYATLTLVLAIIVSLSLPSQTSADDKYDYSSPPPPEKPYKYKSPPPPVYKYKSPP
PPPPEKPYKYPSPPPPPVYKYKSPPPPYKYPSPPPPPKKPYKYPSPPPPIYKYKSPPPPY
KYPSPPPPPYKYPSPPPPVYKYKSPPPPDYKYKSPPPPYKYPSPPPTPKKPYKYSSPPPP
YHY

>TC|TC301532||Fabaceae_G.max||EXTA
MTARGSDPIRGRLRPQMALALAIALISITLVSVNADGYPYYSPPPPYQYTSPPPPPYLYK
SPPPPSPSPPPPYYYKSPPPPSPSPPPPYYYKSPPPPSPSPPPPYYYKSPPPPEKSPAPT
PYYYKSPPPPSPSPPPPYYYKSPPPPSPSPPPPYYYKAPPPPSASPPPAYYYKSPPPPSP
SPTPPYYYKTAPPPSPSPPPPYYYKSPPPPEKPPAPTPYYNKSPPPPSPTPYYYKSPPTP
SPTPYYYKSAPPPPPYYYKSAPTPSPAPYYYKSPPPPPPYYYKSPPPPPYYYKSSSSSIT
NSLLLQVSTTSLSNSVLL

>TC|TC330438||Fabaceae_G.max||EXTA
MGSLMASATLTLVLAIVSLSLPTQILADNYLYSSPPPPKKYPPVSPPYHYPSPPPPPKYY
YHSPPPPSPSPPKKPYYYHSPPPPSPTPPKKPYYYHSPPPPPPKKKPYYYHSPPPPSPPP
PKKPYYYHSPPPPSPSPPKKPYYYHSPPPPSPPPPKKPYYYHSPPPPSPPPPYKKPYYYH
SPPPPSPPPPKPYYYHSPPPPPPKKKPYYYHSPPPPPPKKKPYYYHSPPPPHPYPHPHPH
PHPHPHPPYVYPSPPPPPKKPYKYPSPPPPVHHYPHPHPPFVHYSPPPSPKKPYKYPSPP
PPVHPPYTPHPVYHSPPPPPTNKPYIYASPPPPYHY

>TC|TC283611||Fabaceae_G.max||EXTA
MGSPMASLTLTIALTIISLTLPSQTLADNYIYSSPPPPKHSPPPPYYYHSPPPPKHSPPP
PYYYHSPPPPKHSPPPPYYYHSPPPPKHSPPPPYYYHSPPPPKHSPPPPYYYHSPPPPKH
SPPPPYYYHSPPPPKHSPPPPYYYHSPPPPKHSPPPPYYYHSPPPPKHSPPPPYYYHSPP
PPKHSPPPPYYYHSPPPPKHSPPPPYYYKSPPPPPKKPYKYPSPPPPVYKYKSPPPPYKY
PSPPPPPYKYPSPPPPVYKYKSPPPPVYKYKSPPPPYKYPSPPPPPYKYPSPPPPVYKYK
SPPPPVYKYKSPPPPYKYPSPPPPPYKYPSPPPPVYKYKSPPPPVYKYKSPPPPYKYPSP
PPPPYKYPSPPPPVYKYKSPPPPYKYPSPPPPPYKYPSPPPPVYKYKSPPPPVHSPPPPH
YIYASPPPPYHY

>TC|BW594926||Fabaceae_L.japonicus||EXTA
MTARGSGPIRGRLWPQMAMALAIVFISTTVVSVAADGYLYSSPPPSPSPPPPYHYASPPP
PSPSPAPTYIYKSPPPPVKLPPPPYHYTSPPPPSPSPAPTYIYKSPPPPTKSPPPPVYIY
ASPPPPIYK

>TA|TA798_47247||Fabaceae_L.japonicus||EXTA
MGSLMASISITLALTIMLFSFPSEISANHYAYSSPPPPPKPYSYHSPPPPVHSPPPPPKK
HYKYSSPPPPVHTYPHPHPVYHSPPPPVHTYPHPHPVYHSPPPPPPHEKPYKYASPPPPP
VHTYPHPVYHSPPPPVHSPPPPHYYYKSPPPPYHH

>TC|TC45723||Fabaceae_L.japonicus||EXTA
MGSLMVSATLTLVLAIVSLTLPSQISADGYIYSSPPPPKEYPPVSPPYHYHSPPPPVYSP
PPPKYYYKSPPPPPKKPYKYPSPPPPVHVYPKPTYHSPPPPPKKPYKYPSPPPPVHVYPK
PTYHSPPPPPPKKPYKYPSPPPPVYYPPKHPYHYKSPPPPPKKPYKYPSPPPPVHVYPKP
TYHSPPPPPHKPYKYPSPPPPSHPLPHPYPHPHPVPHPVYHSPP

>TA|CA411436||Fabaceae_L.albus||EXTA
MGSLLASASFGLVLAIIFLSLPSEANYAYHSPPPPYKYHSPPPPVHTYHPHPVYHSPPPS
EHKHYPHPHPHPLPVHHSPPPPTPHKKPYKYPSPPPPEHKYHHPHPIYHSPPPPTPHRKP
YKYPSPPPPEHKYPHPIYHSSPPPSPHKKPYKYPSPPPPPVHTYPPHTPHSIYNSPPPPH
YYYKSPPPSHHY

>NR|gi:49182258||Fabaceae_L.angustifolius||EXTA
MGTSQWPRLIYAVAFCLIATIVVADYHPYNAGQPYYYYQPPSYYYQSPPPPSPSPSPPPP
YVYKSPPPPSPSPPPPYAYKSPPPPSPSPPPPYLYKSPPPPSPSPPPPYVNKSPPPPSSS
PPPPYIYKSPPPPSPSPPPPSPSPPPPSPSPPPPSPSPPPPYVYKSPPPPSPSPPPPSPS
PPPPYHPYLYSSPPPPVY

>TA|CO513118||Fabaceae_M.sativa||EXTA
MRSSMASATLTLLLAIVSLTIPSQISANSYLYSSPPPPSKVYPPVSPPPKVYPPVSPPYH
YSSPPPPKVYPPHSPPPKVYPPVSPPYHYSSPPPPKKYPSHSPPPKVYPPVSPPYHYSSP
PPPKVYPPVSPPPKVYPPVSPPYHYSSPP

>LOC|Medtr4g087180||Fabaceae_M.truncatula||EXTA||Extensin-2 precursor, putative chr04
MGTSFGLRHLPQLIYAIAFCVIASSVAADDYKPYYGGQPNNNYPQPTPPSHGQQPPYYFK
SPPYYYKSPPPPSPSPPPPYVHKYPPYYYKSPPPPSPSPPPPYVYKSPPPPSPXXXXXXX
XXXXXXXXXXXXXXXXXXX

>LOC|AC231338_19||Fabaceae_M.truncatula||EXTA||Extensin-like region
MASITLTIAFTIISLSLPSQISANNYVYSSPPPPSKPYKYASPPPPVYKYKSPPPPVYSP
PPPVYSPPPPVYKYKSPPPPVYSPPPPVYKYKSPPPPVYSPPPPVYKYKSPPPPVYSPPP
PVYKYKSPPPPVYSPPPPVYKYKSPPPPVYSPPPPVYKYKSPPPPVYSPPPPVYKYKSPP
PPVYSPPPPVYKYKSPPPPVYSPPPPVYKYKSPPPPVYSPPPPVYKYKSPPPPVYSPPPP
VYKYKSPPPPVYSPPPPVYKYKSPPPPAYSPPPPHYIYSSPPPPYHH

>LOC|AC231338_13.1||Fabaceae_M.truncatula||EXTA||Extensin-like region
MGSQMASITLTIALAIISLTLPSQTSANSYIYSSPPPPPKPYYYHSPPPPVHSPPPPYHY
SSPPPPPKKPYKYASPPPPVYKYKSPPPPVYKYKSPPPPPKKPYKYPSPPPPVYKYKSPP
PPVYKYKSPPPPVYKYKSPPPPVYKYKSPPPPVYKYKSPPPPPKKPYKYPSPPPPVYKYK
SPPPPVYKYKSPPPPVYKYKSPPPPVYKYKSPPPPPKKPYKYPSPPPPVYKYKSPPPPVY
KYKSPPPPVYKYKSPPPPVYKYKSPPPPPKKPYKYPSPPPPVYKYKSPPPPVYSPPPVYK
YKSPPPPVYSPPPPYKYKSPPPPPYKYPSPPPPVYKYKSPPPPVYKYKSPPPPPKKPYKY
PSPPPPVYKYKSPPPPVYSPPPPVYKYKSPPPPVHSPPPPYKYKSPPPPPYKYPSPPPPV
YKYKSPPPPVYKYKSPPPPPKKPYKYPSPPPPVYKYKSPPPPVYKYKSPPPPVHSPPPPH
YVYSSPPPPVYSPPPPHYIYSSPPPPYHS

>LOC|Medtr4g087340||Fabaceae_M.truncatula||EXTA||Extensin-like region chr04
MGTSIEPRNLPRLMYTIAFCMIAISTFATADDKPYYGGQQPYYNYPQPTTPPSYYYKPPS
YNNYQSPPLYEYKSPPPYDHKFPPYNYYKSPPPSPPPPFAYKFPPYNYQSPPPYEHKFPP
YNYKSPPPYDHKFPPYNYYKPPPPSPPPPYDYKLPPYYYKSPPPPSPSPPPPYVYKSPPP
PPYVYKSPPPPPYVYPSPPPPSPPPPYVYKSPPPPPYVYKSPPPPPYVYPSPPPPSPPPP
YVYKSPPPPPYVYKSPPPPPYVYKSPPPPPYVYPSPPPPSPPPPYVYKSPPPPPYVYKSP
PPPPYVYPSPPPPSPPPPYVYKSPPPPPYVYPSPPPPSPPPPYIYKSPPPPPYVYKSPPP
PPYVYPSPPPPSPPPPYIYKSPPPPPYVYTPPPYVYKSPPPPSPSPPPPYVYKSPPPPPY
VYKSPPPPPYVYESPPPPQYVYKSPPPPPYVYESPPPPSPSPPPPYYYKPIPPPYVYESP
PYIYKSPPPAPYVYKSPPYVYKSPPTPYKPYQYSSPPPPVY

>TC|TC137319||Fabaceae_M.truncatula||EXTA
MTVKASSPKRGHNWPPMAMALAIVLISTSVVSAAADSYIYSSPPPPYYYKSPPPPSPSPP
PPYYYKSPPPPSPIPKTPYYYKSPPPPSPSPPPPYYYKSPPPPSPSPPPPYYYKSPPPPS
PSPPPPYYYKSPPPPSPSPPPPYYYKSPPPPSPIPHTPYYYKSPPPPSPSPPPPYYYKSP
PPPSPIPHTPYYYKSPPPPKVLPPPYYYNSPPPPVAYPHPHPYHHALIVRVVGKVYSFRC
YDWEYPAKSHNKKHLQGAVVEVTCKAGSKIVKAYGKTKNNGKYAITVKDFDYVKYGSTVC
KAALYAPPKGSPFNIPTKLNQGTKLYLKSKDKYEVVLKAKPFAYASKKHFKECEKPKPSP
TPYYYKSPPPPSPVYKYNSPPPPVHYYSPPYTYKSPPPPVKAAPTPYYYKSPPPPAPVYK
YNSPPPPVHYYSPPYTYKSPPPPVKAAPTPYYYKSPPPPAPVYKYNSPPPPVHYYSPPYT
YKSPPPPVKVAPTPYYYKSPPPPAPVYKYNSPPPPVHYYSPPYTYKSPPPPVKAALGFRF
VVYDYYTRCWDLALKFCFCHCSVMASF

>LOC|Medtr2g117580||Fabaceae_M.truncatula||EXTA||Extensin-like region chr02
MGTPLIYAIAFCLIAISISINVAAYKPYYASQPNNYSPPSTKHVEYPRSQKTPYYYAKHP
HLNHKLTPLYKHKSPLSTKHAEYPQSKYTPKHAKHSHPYPKLTPLYAYKSPPPPKYAKHP
QSQYTLYHAKHSHLYPKLTPLYKHKSPLPTKHVEYPQSKYTPEHAKHSHPYPKLTPIYTY
KSPPPPKYAKYPQSKYTSHHAKHSHLYPKLTQLYTYKSPPPPKYAKYPQSQYTPHHAKHS
HLYPKLTPLYTYKSPPPPKHAKYPQSQYTPHHAKHAHLYPKLAPLYTYKSPPPPSPSPPP
PYIYKSPPPPSPSPPPPYVYKSPPPPSPSPQPKYVYKSPPPPSASLPLSYIYKSPPPPSP
SPPPPYIYKSPPPPSPSLPPPYVYKSPPPPSPSPPPPYVYKSPPPPSASLPLSYIYKSPP
PPSPSPPPPYVHKSPPHPSPSPPPPYVYKSPPPPSPSPPPPYVYKSPPPPYASLPLSYVY
NSPPPPSPSPPPPYVYKSPPPPSPSPPPPYVYKSPPPPSPSPPPPYAYKSPPPPSPSPPP
PYVYKSPPPPSSSPPPPYIHKSPSPSRHVLKHPAYHLPYLYNSPPPPPRVY

>TC|CV537755||Fabaceae_P.vulgaris||EXTA
MRSLMASATVTLVLAILSLSLPSQISADTYLYSSPPPPKKPYYYHSPPPPSPPPPKPYYY
HSPPPPSPPPPKPYYYHSPPPPSPPPPKPYYYHSPPPPSPPPPKPYYYHSPPPPSPPPPK
PYYYHSPPPPPKKKPYYYHSPPPPSPPPPKPYYYHSPPPPSPPPPTPYYYHSPPPPSPPP
PKPYYYHSPPPPPKKKPYYYHSPPPPSPPPPKKPYYYHSPPPPKKPYKYSSPPPXVHPY

>TC|TC19108||Fabaceae_P.vulgaris||EXTA
MGFPMASLILSTLALTLISLFPSQTLADNYIYSSPPPPPKPYYYQSPPPPKHSPPPPYYY
HSPPPPKHSPPPPYYYHSPPPPKHSPPPPYYYHSPPPPKHSPPPPYYYHSPPPPKHSPPP
PYYYHSPPPPKHSPPPPYYYHSPPPPKHSPPPPYYYHSPPPPKHSPPPPYYYHSPPPPKH
SPPPPYYYHSPPPPKHSPPPPYYYHSPPPPKHSPPPPYYYHSPPPPKHSPPPPYYYHSPP
PPKHSPPPPYYYHSPPPPKHSPPPPYYYHSPPPPKNPYKYSSPPPPVYKYKSPPPPYKYP
SPPPPPYKYSSPPPPVYKYKSPPPPVYKYKSPPPPVYKYNSPPPPVYKYKSPPPPVYKYN
SPPPPYKYPSPPPPPYKYPSPPPPVYKYKSPPPPVYKYKSPPPPYKYPSPPPPPYKYPSP
PPPVYKYNSPPPPVYKYKSPPPPVYKYKSPPPPYKYPSPPPPPYKYSSPPPPVYKYKSPP
PPVYKYKSPPPPYKYPSPPPPPYKYSSPPPPVYKYKSPPPPVYKYKSPPPPYKYPSPPPP
VYKYKSPPPPVYKYNSPPPPVHSPPPPHYIYASPPPPYHY

>NR|gi:15021742||Fabaceae_P.sativum||EXTA
MRSLMASAALILALAMLFLSFPSEISANQYSYSSPPPPVHSPPPPKDPHHYSSPPPPPPP
VHTYPHPHPVYHSPPPPVHTYPHPHPVYHSPPPPTPHKKPYKYPSPPPPPVHTYPHPHPV
YHSPPPPPTPHKKPYKYPSPPPPPAHTYPPHVPTPVYHSPPPPAYSPPPPAYYYKSPPPP
YHH

>NR|gi:8132441||Fabaceae_P.sativum||EXTA
MGSQMTSITLTIALTILSLTLPSQANNYLYSSPPPPPKPYYYQSPPPPVHSPPPPYHYSS
PPPPVHSPPPPYHYSSPPPPPKKSYKYSSPPPPIYKYKSPPPPVHSPPPPYHYSSPPPPP
KKSYKYSSPPPPVYKYKSPPPPVYKYKSPPPPVKKPYKYSSPPPPVYKYKSPPPPVYKYK
SPPPPVYKYQSPPPPKKSYKYPSPPPPVYKSPPPPYKYQSPPPPPYKYSSPPPPVYKYNS
PPPPYKHISPPTPGKPFKFPPPPTPIYKYKSPPPVYSPPPVYKYKSPPPPVYSPPPPHYV
YSSPPPPVYSPPPPHYIYASPPPPYHY

>TA|BB932621||Fabaceae_T.pratense||EXTA
MGSKGVESIIILSISLLFISTFVSANRVRPPPPPPPPPPPPYEYKSPPPPPTPVYKYKSP
PPPSPTYEYKSPPPPTPSC

>TA|BB939302||Fabaceae_T.pratense||EXTA
MAFSAAVQIFLILGLLATSCLAQAPTAAPTKPPTATPAPKAQPPAAATPPTATPPTATPP
AAPPPPPPPPPPPPPPPPVYKYKSPPPPVYKYKSPPPPVYKYKSPPPPVYKYTSC

>TA|BB931536||Fabaceae_T.pratense||EXTA
MTAMASSPKRGHNWPPMAMALAIVLISTTVVSAAADSYIYSSPPPPYEYKSPPPPSPSPP
PPYVYKSPPPPPYEHKAPSYEYKSPPPPSPSPPPPYVHKSPPYVYKSPPPPPPPPPSA

>TA|TA1507_57577||Fabaceae_T.pratense||EXTA
MRSLMASTTFTLLVALVSLTLPSLISANSYLYSSPPPPKVYPPVSPPPKVYPPVYSPPPK
VYPPVSPPPKVYPPVSPPYHYSSPPPPVYSPPKKPYHYSSPPPPVYSPPPPYHYSSPPPP
PKKPYKYSSPPPPEHHVSPKPYYHSPPPPKKPYYYHSPPPPVYSPPPPYHYSSPPPPPKK
PYYYHSPPPPVYSPPPPYHYT

>TA|TA367_57577||Fabaceae_T.pratense||EXTA
MTARGSDPQRGRFRPEMAMALLIVLISTTVVSVAADGYPYNSPPPPYEYKSPPPPYEHKA
PPYEYKSPPPPSPSPPPPYVYKSPPPPPYEHKAPPYEYKSPPPPSPSPPPPYYYKSPPPP
SPSPPPPYYYKSPPPPSPSPPPPYYYKSPPPPSPSPPPPYYYKSPPPPPPPYEHKAPPYE
YKSPPPPSPSPPPPYYYKSPPPPSPSPPPPYYYKSPP

>TA|TA43_57577(revision)||Fabaceae_T.pratense||EXTA
MGSQMASITLTIALAIITLCLPSQISANNYIYSSPPPPPKPYYYHSPPPPVHSPPPPYHY
SSPPPPPKKPYKYASPPPPVYKYKSPPPPVYKYPSPPPPVYKYKSPPPPVYKYKSPPPPP
KKPYKYPSPPPPVYKYKSPPPPVYKYKSPPPPVYKYKSPPPPVYKYKSPPPPPKKPYKYP
SPPPPVYKYNSPPPPYKYPSPPPPPYKYPSPPPPVYKYKSPPPPVYKYKSPPPPVYKYKS
PPPPVYKYKSPPPPPKKPYKYPSPPPPVYKYNSPPPPYKYPSPPPPPYKYPSPPPPVNK

>NR|gi:791148||Fabaceae_V.unguiculata||EXTA
MTAGSYGPSKGRLRPQMAIALAIVLLSFNVASVAADAYTHSPPPPPPYVYSSPPPPSLSP
PPPYKYKDPHYEYKSPPPPSPSPPPPYYYKSPPPPSPSPPPPYYYKSPPPPSPSPPPPYY
YKSPPPPRPSPPPPYYYKSPPPPSPSPPPPYYYKSPPPPSPSPPPPSYYYKSPPPPSPSP
PPPYYYKSPPPPSPSPPPPYYYKSPPPPSPSPPPPYYYKSPPPPPYEHKDPYYQYKSPPP
QF

>NR|gi:1015937||Fabaceae_V.unguiculata||EXTA
MGTRQWPRLILAFAICLMAITVAADDYKPYYGQPWNNYPKQTPPYYYNAPPYYYKSPPPP
SPSPPPPPYVHKYPPYYYKSPPPPSPSPPPPYVYKSPPPPSPSPPPPYYYKSPPPPSPSP
PPPYYYKSPPPPSPSPPPPYYYKSPPPPSPSPPPPYVYKSPPPPSPSPPPPYYYKSPPPP
SPSPPPPYYYKSPPPPSPSPPPPYYYKSPPPPSPSPPPPYVYKSPPPPSPSPPPPYYYKS
PPPPSPSPPPPYYYKSPPPPSPSPPPPYYYKSPPPPSPSPPPPYYYKSPPPPSPSPPPPY
YYKSPPPPSPSPPPPYYYKSPPPPSPSPPPPYYYKSPPPPSPSPPPPYVYKSPPPPSPSP
PPPYYYKSPPPPSPSPPPPYYYKSPPPPSPSPPPPYYYKSPPPPSPSPPPPYYYKSPPPP
SPSPPPPYVYKSPPPPSPSPPPPYYYKSPPPPSPSPPPPYYYKSPPPPSPSPPPPYYPYL
YNSPPPPAY

>TA|DR064702||Ginkgoaceae_G.biloba||EXTA
MAFFAALLCLVASAAIPHVHANHYAYKSPPPPPYLYKSPPPPSVPSPSYHYKSPPPPTPV
YKYKSPPPPLYQYKSPPPPSPVYKYKSPPPPSHPHKPPHPSHPVYKYKSPPPPHHVEKPY
HYKSPPPPLYKYKSPPPPAYHYKSPPPPSPVYKYKSPPPPHHIVKPGSPYHYKSPPPPVY
KYKSPPPPPYHYKSPPRPPPVYKYKSPPPPTYHYKFPPP

>TC|ES813279||Malvaceae_G.hirsutum||EXTA
MMVPRGNPARGRFWPQMLMAMAVAVVVLASNVGSVSADAYIYASPPPPYEYKSPPPPSPS
PPPPYVYKSPPPPSPSPPPPYVYKSPPPPPKHEEKPYEYKSPPPPPKEKPYEYKSPPPPS
PSPPPPYEYKSPPPPSPSPPPPYVYKSPPPPPKHEEKPYEYKSPPPPPKEKPYEYKSPSC
RIRHELH

>TC|TC12253||Malvaceae_T.cacao||EXTA
MTGPRADPVRGRFWPQVLVALAIVVVASNVGSVSADAYPYSSPPPPYEYKSPPPPSPSPP
PPYEYKSPPPPPPYEYKSPPPPSPSPPPPYHYHSPPPPKHEEKPPYYYKFPPPPSPSPPP
PYYYESPPPPKHEEKPPYYYKSPPPPSPSPPPPYYYESPPPPPKHEEKPPYYYKSPPPPS
PSPPPPYYYKSPPPP

>TA|TA3523_3197||Marchantiaceae_M.polymorpha||EXTA
MELLSRSRGDCAQMSRSTAMVMFTLMAVLVLLCSGAEASAGSRKLRGYYQPPPIYEYKPP
PTYEYKSPPPPVYEYKSPPPPVYEYKSPPPPVYQYKSPPPPVYEYKSPPPPVYEYKSPPP
PVYQYKSPPPPVYQYKSPPPPVXXYKSPPPPVYXXXSXXLQCTSTXLHLPRXTSTSLPPX
GYXXXSXLX

>TA|TA602_3197||Marchantiaceae_M.polymorpha||EXTA
MESGARRGSVPSTTIAMFAVIAVLLLHISAAEAGPGSRKLRTFKFSFPPYGHKSPPYEYK
SPPYEYKPPVEYKPPPYEYKSPPYEYKPPVEYKPPPYEYKSPPYEYKPPMEYKPPPYEYK
SPPYEYKPPMEYKPPPYEYKSPPYEYKPPVEYKPPPFELKPPPYEYKSPPYEYKPPVEYN
PPPYEYKSPPYEYKPPVEYKPPPYEYKPPVEYKPPPYEYKPPVEYKPPPYEYKSPPYEYK
PPVEYKPPPYEYKSPPYEYKPPVEYKPPPYEYKSPPHY

>TC|DR171228||Orobanchaceae_T.pusilla||EXTA
MRNPGRLRQWRHLAYAVAFCLIATGVGANESYNHGNPHEKSKPHYPHKSPPPPTYYYKPP
PPPSKSPPPPTYYYKPPPPPLKSPPPPTYYYKSPPPPSKSPPPPTYYYKSPPPPSKSPPP
PY

>TC|TC7449||Orobanchaceae_T.pusilla||EXTA
MMETLLRLPHWHHFAYALVFCLIAINVVADKPNKPTYVYKSPPPPFSYIYKSPPRPSSSP
PPYIYKSPPPPSSSPPPPYVYKSPPPSSSPPPPYVYKSPPPPLPSPTPPYVYKSPPPPSP
SPPPPYVYKSPPPPSPSPQPPYVYKSPPPPSPSPPPPYIYKSPPPPSPSPPPPYVYKSPP
PPSPSPPPTYVVQVSPSTISITATFHIFYKVPHHPPFSNLLPPXFTF

>TC|DR171228||Orobanchaceae_T.versicolor||EXTA
MRNPGRLRQWRHLAYAVAFCLIATGVGANESYNHGNPHEKSKPHYPHKSPPPPTYYYKPP
PPPSKSPPPPTYYYKPPPPPLKSPPPPTYYYKSPPPPSKSPPPPTYYYKSPPPPSKSPPP
PY

>TC|TC3014||Orobanchaceae_T.versicolor||EXTA
MMETLLRLPHWHHFAYALVFCLIAINVVADKPNKPTYVYKSPPPPFSYIYKSPPRPSSSP
PPYIYKSPPPPSSSPPPPYVYKSPPPSSSPPPPYVYKSPPPPLPSPTPPYVYKSPPPPSP
SPPPPYVYKSPPPPSPSPQPPYVYKSPPPPSPSPPPPYIYKSPPPPSPSPPPPYVYKSPP
PPSPSPPPTYVVQVSPSTISITATFHIFYKVPHHPPFSNLLPPXFTF

>TA|BU668767||Pedaliaceae_S.indicum||EXTA
MATLIATLMVAIVALCFPMITTANYYYSSPPPPKKYYPSPSPPPPYHYKSPPPPPXVHKY
KSPPPPPKKPYVYKSPPPPPPVHKSPPPPPPKKPYVYKSPPPPPKKPYVYKSPPTT

>TA|AM168592||Pinaceae_P.abies||EXTA
MALFIMAVFCFMVSAIVPLVEANSYYYKSPPPPPSPSPPPPPYHYKSPPPPSPSTPPPYH
YNSPPPPSPSPPPPYHYNSPPPPSPSPPPPYHYNSPPPPSHSPPPPYHYKSPPPPSPSPP
PPYHYKSPPPPPYHYKSPPPPSPSPPPPYHYK

>TC|EX306338||Pinaceae_P.glauca||EXTA
MAFLLALLCLMASSTAVFAQYISPPPIPYYYKSPPPPPPSPPPPPPYYYYSPPPPPPSPP
PPPPYYYNSPPPPPPSPPPPPPPYYYYNSPPPPYYYKSPPPYYYNSPRPPPYYYKSPSPY
YYNSPPPSPYYYKSPSPPPYYYNSPPPPAYYYTTPSPPPYYYKPPYYYSTPSPPPYYYKP
PYYYTTPSPPPYSYKSPPY

>TC|DV985544||Pinaceae_P.glauca||EXTA
MMERWSFFDCLRRKTEVLLLVAFFAAHAFMFSLVAASSHSFEGDLNFYYKYKEGDYYKYK
SSSEKDGDYYKYKSSPKYFYKSPPPPSPSPPPPYYYNSPPPPFPSPPPPYYYKSPPPPSP
SPPPPYYYKSPPPPSPFPLPTYYNKSPPPPSPSPPPPYYYKSPPPLFLSPPLPYYYKSPP
PSFPSPPPPYYYKSPPPPSQSSPPPYYYKSPPPPSPSLD

>TC|TC77783||Pinaceae_P.glauca||EXTA
MALFIMAVFCFMVSAIVPLVEANSYYYKSPPPPSPSPPPPPYHYKSPPPPSPSPPPPYHY
SSPPPPSPSPPPPYHYNSPPPPSPSPPPPYHYNSPPPPSHSPPPPYHYKSPPPPSPSPPP
PYHYKSPPPPSPSPPPPYHYKSPPPPSPSPPPPYHYKSPPPPSPSPPPPYHYKSPPPPSP
SPPSSYHYKSPPPPSPSPRPSYHYKSPPPPPYHYKSPPPPSPSPPPYHYKSPPPPSPSPP
PPYHYKSPPPPSPSPPPYHYKYTSPPPPPYHYKYTSPPPPIY

>TA|AL750953||Pinaceae_P.pinaster||EXTA
MALFITALFCFMASAIVPLVEANSYYYKSPPPPTPSPPPPYHYKSPPPPSPSPPPPYHYS
SPPPPSPSPPPPYHYNSPPPPSPSPPPPYHYKSPPPPSHSTPPPYHYNSPP

>TA|TA3605_71647||Pinaceae_P.pinaster||EXTA
MALFIMALLCLLASALPHSVQANFYSYKSPPPPHPSYRYNSPPPPSPSPPPPYHYNSPPP
PSPSPPPPYHYSSPPPPSPSPPPPYHYSSPPPPSPSPPPPYHYKSPPPPSPSPPPPYHYK
SPPPPSPSPPPLYHYKSPPPPTPSPPPPYYYKSPPPPSPSPPPPYQYKSPPPPSPSPPPP
YHYKSPPPPSSSPPPPYHYKSPPPPSPSPPPPYHYKSPPPPSPSPPPPYHYKSQP

>TC|TC87278||Pinaceae_P.taeda||EXTA
MAYTVLVSASVFLAALWWAAWLPAQPVHSSELPAAPHYMSPPPPPPSHHGHRHHRKSPPP
PLMSYISPPPPPPHYVYKSPPPPPPPHYVYKSPPPPPPPPHFVYKSPPPPHSCPPRAHRK
SPPPPFFTYKSPPPPFYFASPPPPRHSSSPTFPPYHYYYQSPPPPPPLRHNLFFPPYNYW
YPPPPSVQSIST

>TC|CO362760||Pinaceae_P.taeda||EXTA
MSLFCLMASVILPSVEANNYAYTSPPPPSPFLPPYYYKSPPPPFASPPPPYHYNSPPPPP
PSPPPPYYYKSPPPPSPSPPPPYHYKSPPPPSPSPPPPYYYKSPPPPSPSPPPPYHYKSP
PPQSPSPPPPYHYNSPPPPSPLPPPPYYYKSPPPPSPLPPPPYYYKSPPPPSPSPPPPYY
YKSPPPPSPSPPPPYYYKSPPPPSPSPPPPYLYKSPPPPSPSPPTP

>TC|TC102928||Pinaceae_P.taeda||EXTA
MALFIMALLCLLASALPHSVQANFYSYKSPPPPHPSYRYNSPPPPSPSPPPPYHYNSPPP
PSPSPPPPYHYSSPPPPSPSPPPPYHYSSPPPPSPSPPPPYHYKSPPPPSPSPPPPYHYK
SPPPPSPSPPPLYHYKSPPPPTPSPPPPYYYKSPPPPSPSPPPPYQYKSPPPPSPSPPPP
YHYKSPPPPSSSPPPPYHYKSPPPPSPSPPPPYHYKSPPPPSPSPPPPYHYKSQP

>TC|TC106825(revision)||Pinaceae_P.taeda||EXTA
MALFITALFCFMAFAIVPLVEADSYYYKSPPPPSPSPPPPYHYNSPPPPSPSPPPPYQYK
SPPPPSHSPPPPYHYNSPPPPSPSPPPPYHYKSPPPPSPSPPPPYHYKSPPPPSPSPPPP
YHYNSPPPPSPSPPPPYHYKSPPPPSPSPPPPYHYKSPPPPSPSPPPPYHYKSPPPPSPS
PPPPYHYKSPPPPSPSPPPPYYYKSPPPPSPSPPPPYHYKSPPPPSHSAPPYHYKYTSPP
PPIY

>TA|AJ802865||Plantaginaceae_A.majus||EXTA
MAPLIVTLIVAIVALCFPSATNADPYVYASPPPPLKSPPPPVYEYKSPPPPVYKYKSPPP
PPKSPPPPVYKYKSPPPPVYKYKSPPPPPPVYKYKSPPPPVYKYKSPPPPPKSPPPPVYK
YKSPPPPVYKYKSPPPPPKSPPPPVYKYKSPPPPVYKYKSPPPPPKSPPPPVYKYESPPP
PVYKYKSPPPPVYKYKSPPPPPKSPPPPIYKYESPPPPVYKYKSPPPPPKS

>NR|gi:4033606||Pteridaceae_A.capillus-veneris||EXTA
MKMKMGASANIYKMQVLSFYALLVVVLGLLLAQAAQAHYHHVPYHHHHDSHWRHPHYHHH
KQRTPWHPHYPHKSPPPSPSSPPYKYKSPPPPSPSPPPPYIYKSPPPPSPSPPPPYIYKS
PPPPSPSPPPPYLYKSPPPPSPSPPPPYIYKSPPPPPCTPSPPPYFYSSPPPPSPSPPPP
YQYKSPPPPSHPSPPPYVYKSPPPPPY

>TA|TA3116_57918||Rosaceae_F.vesca||EXTA
MGALGQPRPYWLISVFYGLALCFLASTTVLANNPYTYSSPPPPQYHATPYPPPTYQPPPP
KYPKHPPYHYKSPPPPPPPKHVIHPPYHYKSPPPPSPSPPPPYVYKSPPPPSPSPPPPYV
YKSPPPPSPSPPPPYVYKSPPPPSPSPPPPYHYKSPPPPSPSPPPPYVYKSPPPPSPSPP
PPYVYKSPPPPSPSPPPPYVYKSPPPPSPSPPPPYVYKS

>TA|TA2000_57918||Rosaceae_F.vesca||EXTAM
MGKMGTSLVFSLLVALVAFASQTSANYPYSSPPPPASPPYHYKSPPPPPPPVHYAPPHHP
YYPPKSPPKPPVYSPPKHPHYPPKSPPSPTPPVYSPPKHPHYPPKSPPKPPVYSPPKHPH
YPPKSPPKPPVYPPPKTPVYHYKSPPPPSPPPPKVPYHYKSPPPPSPPPPKVPYHYKSPP
PPSPPPPKVPYHYKSPPPPPPHKKPYHPHHSPPPPKKPYHYKSPPPPPPPKKPYHYKSPP
PPPPHKKPYHPHHSPPPPKKPYHYKSPPPPTPVYSPPHHPYYPPKSPPKTPVYSPPKHPH
YPPKSPPSPTPPVYSPPKHPHYPPKSPPSPTPPKYSPPKHPHYPPKPPPSPTPPVYSPPK
HPHYPPKSPPYHYKSPPPPSPPPPVYSPPHIPYHYKSPPPPPPPPTKPYKPPTPPVHTPP
AYIYSSPPPPHHY

>TC|TC49978||Rosaceae_M.domestica||EXTA
MRSVGGGPSWGRFWPQMAVALAATLVVISSNVGSVEASAYAYSSPPPPPYVYSSPPPPPY
VYKSPPPPLPSPPPPYVYKSPPPASP

>TC|CN914498||Rosaceae_M.domestica||EXTA
MGKMVPSQVTSLVVTLLVAIVSLTLPSETSANYPYTSPPPPASPPYHYKSPPPPSPSPPV
HYSPPKHPYHPKSPPPPVYAPPKHPYHYKSPPPPKKPYHYKSPPPPYPTPSKYSPPKHPY
HYKSPPPPPKKPYHYKSPPPPSPTPPKYSPPKHPYHYKSPPPPKKPYHYKSPPPPPPPVT
TPSPPKKPYHYTSPPPPPPVYKSPPPPVY

>TA|EE488777||Rosaceae_P.cerasus||EXTA
MGKMGSSSVTSLVVTLLVAIVSLSLPSETSANYPYSSSPPPASPPYHYKSPPPPSPPPPK
HPYHYKSPPPPPPSPPKHPYHYKSPPPPSPSPPKHPYHYKSPPPPSPPKKPYHYKSPPPP
TPVYSPPPPPKKPYKPPTPPVHTAPPHPYIYSSPPPPHHY

>NR|gi:20420||Rosaceae_P.dulcis||EXTA
MGKMGSSSVTSLVVTLLVAIVSLSLPSETSANYPYSSPPPPVSPPYHYKSPPPPSPTPPV
HYSPPKHPYHYKSPPPPVHYSPPKHPYHYKSPPPPVYSPPKHPYHYKSPPPPSPSPPKHP
YHYKSPPPPSPSPPKHPYHYKSPPPPSPSPPKHPYHYKSPPPPSPSPPKHPYHYKSPPPP
SPPKKPYHYKSPPPPPSPTPPVYSPPKHPYHYKSPPPPSPPKKPYHYKSPPPPTPVYKPP
VYSPPPPPKKPYKPPTPPVHTAPPHPYIYSSPPPPHHY

>TC|DY646171||Rosaceae_P.persica||EXTA
MRTSGQLRHCPTMFYALALCLLATSVLATTPYTYSSPPPPKHLETPPTHYKSPPPPKYVK
HPPPPSPSPPPPYYKSPPPPSHSPPPPYVHKSPPPPTHSPPPPYAYKSPPPPSKSPPPPY
VHKSPPYVHKSPPPPSYSPPPPYHYKSPPPPSPSPPPPYHYNSPPPPVKSPPPPQYHYKS
PPPPKHY

>TC|TC7031||Rubiaceae_C.canephora||EXTA
MGSKAKAMGSGLTPLLATVLVVFVALGLPSQTAADDHYYKYSSPPPPYHYPSPPPPVHIP
PPHPVYKYKSPPPPPPHQVYKYKSPPPPPHPVYKYKSPPTTTTPSLQVKGTTTTSSSGTT
PN

>TC|TC35754||Rutaceae_C.clementina||EXTA
MGSQMAYLFATLLAISVSLSYLPLEASADHYYYTSPPPPKKYPPPVASPPYHYTSPPPPK
PVYHSPPQTPVYHSPPPTPVYHSPPPPKKPYEYKSPPPPTPVYHSPPPKPVYHSPPQTPV
YHSPPPVPVYHSPPPPKKPYKYKSPPPPTPVYHSPPPTPVYHSPPPPKKPYEYKSPPPPT
PVYHSPPPPKKPYKYKSPPPPTPVYHSPPPTPVYHSPPPPRTIXVQVSSTPTPVY

>TC|TC17970||Rutaceae_C.sinensis||EXTA
MGSQMAYLFATLLAISVSLSYLPLEASADHYYYTSPPPPKKYPPPVASPPYHYTSPPPPK
PVYHSPPQTPVYHSPPPTPVYHSPPPPKKPYKYKSPPPPTPVYHSPPPTPVYHSPPPPKK
PYKYKSPPPPTPVYHSPPPPKKPYKYKSPPPPTPVYHSPPPTPVYHSPPPPKKPYKYKSP
PPPTPVYHSPPPHHPYVYASPPPPYHY

>TC|TC19821||Rutaceae_C.sinensis||EXTA
MGSQMASLFATLLVISVSLSYFPLEASADHYYYTSPPPPKKYPPPVVSPPYHYTSPPPPK
PVYHSPPPTPVYHSPPPTPVYHSPPPKPVYHSPPPPKKPYKYKSPPPPTPVYHSPPPPKK
PYKYKSPPPPTPVYHSPPPTPVYHSPPPPKKPYVYKSPPPPTPVYHSPPPPKKPYKYQSP
PPPTPVYHSPPPPKKPYKYKSPPPPTPAYHSPPPPKKPYKYKSPPPPPVYTSPPHHPYVY
ASPPPPLHY

>TA|CX173034(revision)||Salicaceae_P.deltoides||EXTA
MGSPVACLLATLLVATISLSLPSESTATYKYSSPPPPPPPYHYKSPPPPPPVHSPPAPVH
PYKYKAPPPPPPGHKPPPPPPTHPYKYKSPPPPAPVHKPPPAPPVHPYKYKAPPPPPPEH
SAPAPPPPPTHPYKYKSPPPPPVHSPPPPTHPYKYKSTPPPPVHSPPPPPSPVYKYKSTP
PPPVYKSPPTTPA

>TA|CV130884||Salicaceae_P.deltoides||EXTA
MECRGKMGHLSPMIHAIAICLVATSVVAYEPYYYKSPPPPSQSPPPPYHYSSPPPPKKSP
PPPYHYTSPPPPKKSPPPPYHYSSPPPPKKSPPPPYHYTSPPPPKKSPPPPYHYSSPPPP
KKSPPPPYHYTSPPPPKKSPPPPYHYSSPSPPKKSPPPPYHYTSPPPPKKSPPPPYHYSS
PPPPKKSPPPPYHYTSPPPPKKSPPPPYHYSSPPPPKKSPPPPYHYTSPPPPKKSPPPPY
HYSS

>TA|TA2059_3696||Salicaceae_P.deltoides||EXTA
MGSPMASLLATLLLPTISLSLPSESTATYKYSSPPPPPPPYHYKSPPPPPPVHSPPPPVH
PYKYKSPPPPPPVHKPPPPPPTHPYKYKSPPPPPPVHKPPPPPPVHPYKYKSPPPPPPVH
SPPPPPPTHPYKYKSPPPPPPVHSPPPPPPTHPYKYKSPPPPPVHSPPPPTHPYKYKSPP
PPPVHSPPPPPSPVYKYKSPPPPPPVQKSPPLPPKKPYEYKSPPPPPPVHKSPPLPPKKP
YKYKSPPPPPVYKSPPPPPPVYKSPPPPPHKKPYKYKSPPPPPPVYKSPPPPPHKKPYKY
KSPPPPTQ

>TA|AJ776538(revision)||Salicaceae_P.euphratica||EXTA
MASLLVTLLVATISLSFPSESTANYKYSSPPPTKKSPPPPPPPYHYKSPPPPPPVHSPPP
PVHPYKYKSPPPPPPVHKPPPSPPKNPYKYKSPPPPPVHSSPPPTHPYKYKSPPPPPPVH
KSPPPPHYIYASPPPPHHY

>TA|TA6827_113636||Salicaceae_P.tremula||EXTA
MGSPMASLLATLLMATISLSLPSESTADYKYSSPPPPKKSSPPPPPPYHYKSPPPPPPVH
SPPPPVHPYKYKSPPPPPPVHKPPPSPPTHPYKYKSPPPPPVHKPRPYKYKSPPPPPSSP
PAPPPYHYKSPPPTPPVHTPPPPVHPYKY

>TC|TC127639||Salicaceae_P.trichocarpa||EXTA
MASLLVTLLVATISLSFPSESTANYKYSSPPPTKKSPPPPPPPYHYKSPPPPPPVHSPPP
PVHPYKYKSPPPPPPVHKPPPSPPKNPYKYKSPPPPPVHSPPPPTHPLQVQVSSTTTTST
QVSTTTSLHLRITPSTSPLLDHGAHRFKVKGRVSCNKEKDASRS

>NR|gi:224082422||Salicaceae_P.trichocarpa||EXTA
MRSSMASLTATLLLVAISLSLPSQTTANYEYSSPPPPKKSPPPPPPPYHYKSPPPPPPVH
SPPPPPHPYKYKSPPPPPPVHKSPPPPKKPYKYKSPPPPPVHSPPPPSHPYKYKSPPPPP
PVYKYKSPPPPPPVYKSPPPPPKKPYNTQVSSTTSSQEAIQIQVSSPTSYSSL

>NR|gi:224142917||Salicaceae_P.trichocarpa||sp4+,yky-containing
MDLLHSVMLYFSLALLLSSSSEATDTTFSRNSALWLTYKSPPPPFHNKHKSPPPPPHKYK
SPPPPHHKCKYSPPPPVYTYRSPPPPTPMHHKSPPPSPHMFKSPPPPPYRYISPPPPPPH
PPCHAYKYLSPPPPPSYKYASPPPPKHHHHHKHKWSPYPFITYMSPPPPHHNYPDYHYSS
PPPPPPIVAY

>TC|TC108756||Salicaceae_P.trichocarpa||EXTA
MRSSMASLTATLLLVAISLSLPSQTTANYEYSSPPPPKKSPPPPPPPYHYKSPPPPPPVH
SPPPPPHPYKYKSPPPPPPVHKSPPPPKKPYKYKSPPPPPVHSPPPPSHPYKYKSPPPPP
PVYKYKSPPPPPVYKSPPPPPEKPYKYKSPPPPPPVYKSPPPPPPEKPYKYKSPPPPPTP
VYKYKSPPPPPPVHKSPPPPPPKKPYKYKSPPPPPTPVYKYKSPPPPPPVHKSPPPPHYI
YASPPPPHHY

>NR|gi:224114153||Salicaceae_P.trichocarpa||EXTA
MENRGRMGHLSPMIHAIAICLVATSVVAYEPYYYKSPPPPSQSPPPPYHYSSPPPPKKSP
PPPYHYTSPPPPKKSPPPPYHYSSPPPPKKSPPPPYHYSSPPPPKKSPPPPYHYSSPPPP
KKSPPPPYHYSSPPPPKKSPPPPYHYSSPPPPKKSPPPPYHYSSPPPPKKSPPPPYHYSS
PPPPKKSPPPPYHYTSPPPPKKSPPPPYHYSSPPPPKKSPPPPYHYTSPPPPKKSPPPPY
HYSSPPPPKKSPPPPYHYSSPPPPKKSPPPPYHYSSPPPPKKSPPPPYHYSSPPPPKKSP
PPPYHYTSPPPP

>TC|TC106555||Salicaceae_P.trichocarpa||EXTA
MGSPMASLLATLLVPTISLSLPSESTATYKYSSPPPPPPPYHYKSPPPPPPVHSPPPPVH
PYKYKSPPPPPPVHKPPPPPPTHPYKYKSPPPPPPVHKPPPPPPVHPYKYKSPPPPPPVH
SPPPPPPTHPYKYKSPPPPPPVHSPPPPPPTHPYKYKSPPPPPVHSPPPPTHPYKYKSPP
PPPVHSPPPPPSPVYKYKSPPPPPPVHKSPPLPPKKPYKYKSPPPPPVHSPPPPPSPVYK
YKSPPPPPPVHKSPPLPPKKPYKYKSPPPPPVYKSPPPPPPVYKSPPPPPHKKPYKYKSP
PPPTPVYKYKSPPPPPPVHKSPPPPHYIYASPPPPHHY

>NR|gi:224128918||Salicaceae_P.trichocarpa||EXTA
MTGSSGGSLWGRLWPQLAVALVILFVSSNVGSVSGDAYVYSSPPPPYVYKSPPPPSPSPP
PPYEYKSPPPPSPHPPPTYVYKSPPPPSPSPPPPYIYKSPPPPSPSPPPPYEYKSPPPPS
SSPPPPYIYKSPPPPSPSPPPPYVYKSPPPPSPSPPPPYIYKSPPPPSPSPPPPYYYKSP
PPPSPSPPPPYVYKSPPPPSSSPPPPYYYKSPSPPSSSPPPPYYYKSPPPLSPSPPPPYY
YKSPPPPDPSPPPPYHYKSPPPPSPSPPPPYYYRSPPPPSSSPPPPYHYSSPPPPSPSPP
PPYYYKSPPPPSPSPPPPYYYKSPPPPSPSPPPPYVYKSPPPPSPSPPPPYYYHSPPPAM
KSPPLSVYIYASPPPPIHY

>TA|CX187427||Salicaceae_P.x||EXTA
MRSSMASLTATLLLVAISLSLPSQTTAKYEYSSPPPPKKSPPPPPPPYHYKSPPPSPPVH
SPPPPPHPYKYKSPPPPPPVHKSPPPPKKPYKYKSPPPPPVHSPPPPSHPYKYKSPPPPP
PVYKYKSPPPPPPVYKSPPPPPEKPYKYKSPPPPPVYKLPPPPPEKPYKYKSPPPPPTPV
YKYKSPPPPPPVHKSP

>TA|TA459_88036||S.moellendorffii||EXTA
MGERSSPLLLAVLVILGISSCGLTVFAARPLEEQKTLDWLHFPPLPPFPYFSHPSPGYKP
PSYSYPAPEYKSPPSYSYPTPEYKSPPSYSYPAPEYKSPPSYSYPAPEYKAPPTVPSSPA
PVYKPPTVPSTPAPVYKPPTPSPVYKPPTPVYKPPTPSPVYKSPPPPSSGY

>jgi|Selmo1|431330|fgenesh2_pg.C_scaffold_150000025||S.moellendorffii||sp3,yxy-containing
MNFFKSCSMLLLLLLVSAVRGDAKNFGFGIPKSTIDFFCWRPWLRRPWWFPNRLCPPNAP
PYCYKSPPPPPPPPKCEKSPPPYEYKSPRPPPKFEKSPPPPPPYECKSPPPPPKCEKSPP
PYEYKFPSPPPLPPYKYESPPPFRYKYESPPPPHNSPPLPPPPIYWYQSPPPLRELVTFC
PGL

>jgi|Selmo1|402661|fgenesh2_pg.C_scaffold_0000666||S.moellendorffii||EXTA
MKGVILGFAIESAMIFFKSCSMLLLLLVYTVTCDGSQEVLDRNTGSFGDAKNFGFGIPKS
TIDFFCWRPWLRRPWWFPNRLCPPNAPPYCYKSPPPPMELPTPPVESQPPPVESPSPPHH
QKSPPPPVESPPPPYQYKSPPPPPPPPKCGKTPPPPPPPPKCEKSSPPPPPKSPPPPPPK
REKCPPPYEYKSPPPPPPKCEKSPPPPAPYEYKSPPPPPSSPPSPLYKYESPPPPAYEYK
CAPPPPPPPHHQHVKSPPPPSLPIYWYQSPPPELVKK

>jgi|Selmo1|440537|estExt_fgenesh2_pg.C_110148||S.moellendorffii||EXTA||EXTA1
MGPSPTLVVFFVGVTLAALAGADEPPVYKYASPPPPTYYYKSPPPPVYKYKSPPPPVYSP
PPPVYKYKSPPPPVYSPPPPVYKYKSPPPPVHSPPPPVYKYKSPPPPVYSPPPPVYKYKS
PPPPVHSPPPPVYKYKSPPPPVYSPPPPVYKYKSPPPPVHSPPPPVYKYKSPPPPVHSPP
PPVYKYKSPPPPVYSPPPPVYKYKSPPPPVYSPPPPVYKYKSPPPPYHY

>jgi|Selmo1|229952|fgenesh1_pm.C_scaffold_0000137||S.moellendorffii||EXTA||EXTA2
MGTRLMASHSSIALGWALALVTIAALGEINVVSANYDYSSPPPPYVYKSPPPPSPSPPPP
YIYKSPPPPSPSPPPPYVYISPPPPSPSPPPPYIYKSPPPPSPSPPPPYVYKSPPPPSPS
PPPPYVYKSPPPPSPSYVYQSPPPPSPSRPPPYVYSSPPPPSPSPPPPYVYKSPPPPSPS
PPPPYVYKSPPPPSPSPPPPYVYKSPPPPSPSPPPPYVYKSPPPPSPSPPPPYVYQSPPP
PSPSPPPPYYYKSPPPPSPSPPPPYIYKSPPPPSPSPPPPYVYMSPPPPSPSPPPPYVYS
SPPPPPVYYYSSPPPPSYY

>jgi|Selmo1|408063|fgenesh2_pg.C_scaffold_8000089||S.moellendorffii||EXTA||EXTA3
MGTRLVASRSSIALGWALALVTIAALGEINVVSANYDYSSPPPPYVYKSPPPPSPSPPPP
YIYKSPPPPSSSPPPPYVYKSPPPPSPSPPPPYIYKSPPPPSPSPPPPYVYKSPPPPSPS
PPPPYVYKSPPPPSPSPPPPYVYQSPPPPSPSPPPPYVYSSPPPPSPSPPPPYVYKSPPP
PSPSPPPPYVYKSPPPPSPSPPPPYVYKSPPPPSPSPPPPYVYKSPPPPSPSPPPPYVYK
SPPPPSPSPPPPYVYQSPPPPSPSPPPPYYYKSPPPPSPSPPPPYIYKSPPPPSPSPPPP
YVYMSPPPPSPSPPPPYVYSSPPPPPVYYYSSPPPPSYY

>jgi|Selmo1|429269|fgenesh2_pg.C_scaffold_113000001||S.moellendorffii||EXTA||EXTA4
MGSCHFAMALLAAALVLLRSSQVQAFDFSFPPMPDFYKYLSPPPPPYYKYESPAPPYYKY
VSPPPPYYYKSSPPPYYKYESPPYKHESPPPPHYYYKSPPPPYYKYESPPYKYESPPPPP
YKYESPPPPPYKYESPPPPPYKYESPPPPPYKYESPPPPPYKYESPPPPPYKYESPPPPP
YKYESPPPPPYKYESPPPPPYKYESPPPPPYKYESPPPPPSNMSLCHHIRVSTTSLLEVQ
VLPPPPYYKYESPPPPYYKYKAPPPARPQRLLEKLMFIYNYN

>jgi|Selmo1|448871|estExt_fgenesh2_pg.C_1400013||S.moellendorffii||EXTA||EXTA5
GKVKITCGESYYYGYTNKHGIFRIELPHQSWEDASSCKAKIIKSSYIKSSCNVITDYRSG
ATGAKLKFKSKTEKELVLTAGPFIYATPEPSSVCFYTSPPYEYKSPPPPTPVYHYASPPP
PVYYYKSPPPPPYYYKSPPPPPPNYYYKSPPPPSPYYYKSPPPPPYYYKSPPPPPYKYES
PPPPPYKYESPPPPPYYYKSPPPPPYKYESPPPPVYKYETPPPPVYKYKSPPPPYKYESP
PPPPYKYESPPPPPYYYKSPPPPPYKYESPPPPPYKYESPPPPVYKYKSPP

>jgi|Selmo1|422106|fgenesh2_pg.C_scaffold_55000016||S.moellendorffii||EXTA||EXTA6
MGSCHFAMALLAAALVLLKSSQVQAFDFSFPPMPDFTPYYKYLSPPPPSYYKYESPAPPY
YKYVSPPPPYYYYKSPPPPYYKYESPPYKYESPPSPPYKYESPPPPPYKYESPPPPPYKY
ESPPPPPYKYESPPPPPYKYESPPPPPYKYESPPPPPYKYQSPPPPPYKYESPPPPPYKY
ESPPPPPYKYESPPPPPYKYESPPPPPYKYESPPPPPYKYQSPPPPPYKYESPPPPPYKY
ESPPPPPYKYESPPPPPYKYESPPPPPYKYESPPPPPYKYESPLPPPYKYESPPPPPYKY
EPPPPPPYKYQSPPPPPYNPPPPPYKYESPPPPPYKYQSPPPPSYKYESPPPPPYKYESP
PLPPYKYESPPPPPYKYESSPPPPYY

>jgi|Selmo1|422873|fgenesh2_pg.C_scaffold_59000002||S.moellendorffii||EXTA||EXTA7
MEKYRRNSVCVLVCRCKWDFFVAISSLISSKETPQATGYRFVNCKYAFRTRLEAEAVRAN
VRPIRVDCFSQLTRSPPFSLRGALKANTKHTKGRKETPSPGSDKLRALELGLDRLEKKER
WWRQEIQGSISVWDFDQYESPPYKYESPPPPHYYYKSPPPPHYYYKSPPPPYYKYESPPY
KYESPPPPPYKYESPPPPPYKYESPPPPPYKYESPPPPPYKYESPPPPPYKYESPPPPPY
KYESPPPPPYKYESPPPPPYKYKSPPPPPYKYESPPPPPYKYESPPPPPYKYESPPPPPY
KYESPPPPPYKYESPPPPPYKYESPPPPPYKYESPPPPPYYYKSPPPPYYKYESPPPPYY
KYESPPPPYYYYKSPPPPYYKYESPPPPYYKYVSPPPPAYKK

>jgi|Selmo1|449207|estExt_fgenesh2_pg.C_1690013||S.moellendorffii||EXTA||EXTA8
MAARSGGTRLHWLAVIQFVALVATVGNAATPALAEAEGGAETKTYTYSSPPPPSPVYEYK
SPPPPVYEYKSPPPPPPVYKYKSPPPPPPYYYKSPPPPPPYKYESPPPPPYKYESPPPPP
YYYKSPPPPPYKYESPPPPPYKYESPPPPPYKYESPPPPPYKYESPPPPPYKYESPPPPP
YKYESPPPPPYYYKSPPPPPYKYESPPPPPYYYKSPPPPPPYYYKSPPPPSPVYKYKSPP
PPPYKYESPPPPPYYYKSPPPPPYYYKSPPPPPPVYKYKSPPPPPYYYKSPPPPSPVYYY
KSPPPPAYYYKSPPPPVYYYKSPPPPAYHYQSPPPPSGKVKITCGESYYYGYTNKHGIFR
IELPHQSWEEASSCKAKIIKSSYIKSSCNVITDYRSGATGAKLKFKSKTEKELVLTAGPF
IYATPEPSSVCFYTSPPYEYKSPPPPTPVYHYASPPPPVYYYKSPPPPSPVYYYKSPPPP
SPVYYYKSPPPPPYYYKSPPPPPPKYYYKSPPPPSPYYYKSPPPPPYYYKSPPPPPYKYE
SPPPPPYKYESPPPPPYYYKSPPPPPYKYESPPPPVYKYESPPPPVYKYKSPPPPYKYES
PPPPPYKYESPPPPPYYYKSPPPPPYKYESPPPPPYKYESPPPPVYKYKSPPPPPYYYKS
PPPPPYKYESPPPPPYKYESPPPPPYYYKSPPPPPYKYESPPPPPYKYESPPPPVYKYKS
PPPPPYYYKSPPPPPYKYESPPPPPYKYESPPPPPYYYKSPPPPPYKYESPPPPPYKYES
PPPPPYYYKSPPPPPYKYESPPPPVYKYESPPPPVYKYKSPPPPYYYKSPPPPPYKYESP
PPPPYKYESPPPPPYYYKSPPPPPYKYESPPPPPYKYESPPPPPYYYKSPPPPPYKYESP
PPPPYKYESPPPPPYKYESPPPPPYKYESPPPPPYKYESPPPPPYKYESPPPPPYYYKSP
PPPEYQSPPYVYKSPPPPSY

>jgi|Selmo1|414941|fgenesh2_pg.C_scaffold_25000181||S.moellendorffii||EXTA||EXTA9
MRTSQVRGLGLVPLLLMVLVANAYDVDPPSPDYKSPPPPKYEYKSPPPPEYDPSPVYEYK
SPPPPKYEYKSPPPPLKYDPSPVYEYKSPPPPKYEYKSPPPPLKYDPSPVYEYKSPPPPK
YEYKSPPPPPKYDPSPVYEYKSPPPPKYEYKSPPPPPKYDPSPVYEYKSPPPPKYEYKSP
PPPKYDPSPVYEYKSPPPPKYEYKSPPPPKYDPSPVYEYKSPHPPNESSERRKKPKEKAC
HIAS

>jgi|Selmo1|405114|fgenesh2_pg.C_scaffold_3000337||S.moellendorffii||EXTA||EXTA10
MRTSQVRGLGLVPLLLMVLVANAYDVDPPSPDYKSPPPPKYEYKSPPPPEYDPSPVYEYK
SPPPPKYDPSPVYEYKSPPPPKYEYKSPPPPLKYDPSPVYEYKSPPPPKYEYKSPPPPLK
YDPSPVYEYKSPPPPKYDPSPVYEYKSPPPPKYEYKSPPPPPKYDPSPVYEYKSPPPPKY
EYKSPPPPKYDPSPVYEYKSPPPPKYEYKSPPPPKYDPSPVYEYKSPPPPKYEYKSPPPP
VKYDPSPVYEYKSPPPPKYEYKSPPPPKYDPSPVYQYKSPPPPKYEYKSPPPPPKYDPSP
VYQYKSPPPPKYEYKSPPPPPKYDPSPVYQYKSPPPPKYEYKSPPPPKYDPSPVYKYTSP
PPPKYEYKSPPPPKYDPSPVYKYTSPPPPKYEYKSPPPPKYDPSPVYKYKSPPPPLYYYK
SPPPPAKYKSPPPPKYDPSPVYEYKSPPPPKYDPSPVYEYKSPPPPKYDPSPVYEYKSPP
PPKYDPSPVYEYKSPPPPKYDPSPVYEYKSPPPPKYDPSPVYLYKSPPPPKYDPSPEYKS
PPPPAY

>jgi|Selmo1|405021|fgenesh2_pg.C_scaffold_3000244||S.moellendorffii||EXTA||EXTA11
MGPPRRTRMPMEATAALSLVVIALAFAAQVEASPYEYKSPPPPVYETPAPVYKYKSPPPP
PPVYKYASPPPPVYHAPAPVYKYKSPPPPVYHYSSPPPPVYKYKSPPPPVYHAPAPVYNS
PPPPVYKYKSPPPPVYHAPAPVYKYKSPPPPVYHYTSPPPPVYKYKSPPPPVYHAPAPVY
KYKSPPPPVYHYSSPPPPVYKYKSPPPPVYHAPAPVYKYKSPPPPVYHYSSPPPPVYKYK
SPPPPVYHAPAPVYKYKSPPPPVYHYSSPPPPVYEYKSPPPPVYHAPAPVYKYKSPPPPV
YKYQSPPPPVYHSPAPVEDDNKCCRIFLEIRTRVAAVETELLHRKREM

>jgi|Selmo1|442529|estExt_fgenesh2_pg.C_250099||S.moellendorffii||EXTA||EXTA12
MGPPRRTRMPMEATAALSLVVIALAFAAQVEASPYEYKSPPPPVYETPAPVYKYKSPPPP
PPVYNSPPPPVYKYKSPPPPVYHAPAPVYKYESPPPPVYKYSSPPPPVYKYKSPPPPVYH
APAPVYKYKSPPPPVYHYSSPPPPVYKYKSPPPPVYHAPAPVYKYNSPPPPVYKYSSPPP
PVYKYKSPPPPVYHTPAPVYKYESPPPPVYKYSSPPPPVYKYKSPPPPVYHVPAPVYKYE
SPPPPVYKYSSPPPPVYKYKSPPPPVYHAPAPVYKYKSPPPPVYKYSSPPPPVYKYKSPP
PPVYHAPAPVYKYKSPPPPVYHYSSPPPPVYKYKSPPPPVYHAPAPVYKYKSPPPPVYHY
SSPPPPVYKYKSPPPPVYHAPAPVYKYKSPPPPVYHYSSPPPPVYKYKSPPPPVYHAPAP
VYKYKSPPPPVYHYSSPPPPVYKYKSPPPPVYHAPAPVYKYKSPPPPVYKYQSPPPPVYH
SPAPVEDDNKCCRIFLEIRTRVAAVETELLHRKREM

>TC|CA523779||Solanaceae_C.annuum||EXTA
MASITRASSLTLFLSFNLLFFAIVSGTTDCFSCPYPPPYYYHSPPPPSPSPPPPYYYXSP
PPPVKSPPPPXYYNSPPPPVKSTPPPVYIYTSPPPPVHY

>NR|gi:11121502||Solanaceae_N.sylvestris||EXTA
MAKIVSLLATLVVALLSLSFPLECKANYYYSSPPPPTKKYVYSSPPPPVYKYKSPPPPLP
IYRSPPPPVYKYKSPPPPVYKYKSPPPPPPVYKSPPPPVYKYKSPPPPVYKYKSPPPPPP
VYKSPPPPVYKYKSPPPPVYKYKSPPPPPPVYKSPPPPVYKYKSPPPPPPVHKSPPPPIY
KYKSPPPPVYKYKSPPPPPPMYKSPPPPVYKHKSPPPPPPVYKYKSPPPPVYKYKSPPPP
PPVYKSPPPPIYKYKSPPPPPPVYKSPPPPVYKYKSPPPPPPVYKSPPPPVYKSPPPPYH
YYYTSPPPPHY

>TC|DW002936||Solanaceae_N.tabacum||EXTA
MAKIVSLLATLVVALLSLSFPLECKANYYYSSPPPPTKKYVYSSPPPPVYKYKSPPPPLP
IYRSPPPPVYKYKSPPPPVYKYKSPPPPPPVYKSPPPPVYKYKSPPPPVYKYKSPPPPPP
VYKSPPPPVYKYKSPPPPVYKYKSPPPPPPVYKSPPPPVYKYKSPPPPPPVHKSPPPPIY
KYKSPPPPVYKYKSPPPPPPVYKSPPPPVYKSPPPPYHYYYTSPPPHY

>TC|TC216853||Solanaceae_S.lycopersicum||EXTA
MRLFAGGPGRGRHYLPHILVAILALVDIVSADPYIYASPPPPYVYKSPPPPSPSPPPPYV
YKSPPPPSPSPPPPYVYKSPPPPSPSPPPPYVYKSPPPPSHSPPPPYYYKSPPPPSPSPP
PPYYYKSPPPPSPSPPPPY

>TC|TC215255||Solanaceae_S.lycopersicum||EXTA
MAKIAYLLTTLLVALVSLSFPSECKANYYYTSPPPPTPVYKYKSPPPPVYKYKSPPPPPP
IYKSPPPPIYKYKSPPPPPVYKYKSPPPPVYKYKSPPPPLETLHQTCAEVNSHLYQVKAV
AEEMGIGFLGTGFQPKWGLKDIPIMPKGRYEIMRNYMPKVGSLGLDMMFRTCTVQVNLDF
SSEADMIRKFRAGLALQPIATALFANSPFTEGKPNGYLSKRSHIWTRYR

>TC|TC213235||Solanaceae_S.lycopersicum||EXTA
MAKIAYLLTTLLVALVSLSFPSECKANYYYTSPPPPTPVYKYKSPPPPVYKYKSPPPPPP
IYKSPPPPIYKYKSPPPPPVYKYKSPPPPVYKYKSPPPPPPVYKYKSPPPPVYKYKSPPP
PPPVYKYKSPPPPIYKYKSPPPPVYKYKSPPPPVYKYKSPPPPVYKYKSPPPPVYKYKSP
PPPVYKYKSPPPPVYKYKSPPPPVYKYKSPPPPVYKYKSPPPPVYKYKSPPPPVYKYKSP
PPPVYKYKSPPPPVYKYKSPPPPVYKYKSPPPPVYKYKSPPPPVYKYKSPPPPVYKYKSP
PPPVYKYKSPPPPVYKYKSPPPPVYKYKSPPPPVYKYKSPPPPVYKYKSPPPPVYKYKSP
PPPVYKYKSPPPPIYKYKSPPPPVYKYKSPPPPIYKYKSPPPPVHKSPAPYYYTSPPPPS
HY

>TC|TC166047||Solanaceae_S.tuberosum||EXTA
MAKIAYLLTTLLVALVSLSFPSECKANYYYTSPPPPTPVYKYKSPPPPVYKYKSPPPPPP
VYKSPPPPVYKYKSPPPPVYKYKSPPPPPPVYKYKSPPPPVYKYKSPPPPPPVYKYKSPP
PPVYKYKSPPPPPPVYKYKSPPPPVYKYKSPPPPPPVYKYKSPPPPVYKYKSPPPPPPVY
KYKSPPPPVYKYKSPPPPTPVYKYKSPPPPVYKYKSPPPPTPVYKYKSPPPPVYKYKSPP
PPTPVYKYKSPPPPVYKYKSPPPPPPVYKYKSPPPPVYKYKSPPPPRLQVQVSTTSSIQV
QVSTTTRLQVQVSTTSSL

>NR|gi:225463897||Vitaceae_V.vinifera||EXTA
MGRGGRGPSMASLIATLLVVTISLSLPSETSANYHYSSPPPPYYYKSPPPPPPVYKYKSP
PPPPPVYSPPHHPPYKYKSPPPPPPVYKYKSPPPPPPVYSPPHHPPYKYKSPPPPPPVYS
PPHHPPYKYKSPPPPPPVYSPPHHPPYKYKSPPPPPPVYSPPHHPPYKYKSPPPPPPVYK
YKSPPPPHKKPYKYKSPPPPPYNLPSGTSADEYEYKSPPPYKYKSPPPPPPVYKYKSPPP
PPPVHSPPPPPPPYKYKSPPPPPPVYKYKSPPPPSPPYKYKSPPPPPYKYKSPPPPPLQY
KSPPPPPYKYKSPPPPPPVYKYKSPPPPVYKYKSPPPPPYMYKSPPPPPPVYKYKSPPPP
PPKYYYSSPPPPPPHHY

>NR|gi:1486265||Apocynaceae_C.roseus||EXTAB
MTPLFTALVIALVALCLPSQTTADYKYSSPPPPYHYSSPPPPVHSPPPPPVYKYKSPPPP
PPIHKSPPPPPYVYKSPPPPPPVYKYKSPPPPPPVHKYPPYIYKSPPPPPPIYKSPPPPV
YKSPPPPKNPYVYKSPPPPPPVYKYLHHLHQKKPYVYKSPPPPPFVHKSPPPPVYKSPPP
PKKPYVYKSPPPPPPIHKSPPPPYHYYYSSPPPPHHY

>TA|BQ791180||Brassicaceae_B.rapa||EXTAB
MGSPMASLAATLLVLALSLGFVSETTANYYYSSPPPPVKHYTPPVYKSPPPPVKHYSPPV
YKSPPPPKKDYEYKSPPPPVKHYSPPPSTSLHLLPRNITSTNHLLHRFTNLLLLRFTTLL
HHLKNTTSTNHLLHRFTSLLPLRSTTLLHHLRNTTSTNLLLHRVTSLLL

>TC|TC48903||Fabaceae_L.japonicus||EXTAB
MGSLMASISITLALTIMLFSFPSEISANHYAYSSPPPPPKPYSYHSPPPPVHSPPPPYEK
PHPVYHSPPPPYEKPHPVYHSPPPPPPHKPYKYPSPPPPPHKYPHPHPHPVYHSPPPPPP
KKHYKYSSPPPPVHTYPHPHPVYHSPPPPVHTYPHPHPVYHSAPPPPPHEK

>TC|TC16086||Fabaceae_P.vulgaris||EXTAB
MGSLMASASITLAFAIILFSLPSQISGNHYSYSSPPPPPVSSPPPPYHYPSPPPPVHKPY
PHPHPVYQSPPPPVHKPYPHPHPVYQSPPPPPHHYPHPHPHPHPHPIYHSPPPPPPKKPY
KYPSPPPPVHKPYPHPHPVYHSPPPPPKKSYKYSSPPPPVHRHRYPHPHPVYHSPPPPVP
TYPPHVPTPVYHSPPPSPHKKPYKYKSPPPPVPSPPPPRYYYKSPPPPPPYHY

>TC|DV703997||Rubiaceae_C.canephora||EXTAB
MGSKAIATGSRLTPLLTTVLVVFVALGLPLETAADYYKYSSPPPPPYHYSSPPPPVYSPP
PPPVYKLPPPSPLVYKSSPPPYHYSPPPVYSPPPSPVYKSPPPPPPVYNSPPPPVYKYKS
PPPPPPVYRISTATRLQVQVTPATSSSIQVSSATCLRVQVSSTTSSGVQVPTT

**@ EXTB Class [16 Proteins, 15 Species]**

>TC|TC4074||Alliaceae_A.cepa||EXTB MKSFTLCFALIAMLSLHFTNAVQTQYTIAPSQPPTHEPHHHVHHHHHESPPKPKHTEHHH
HSSSPPIVSVQRPSYHKPPPSVYLPPPVHHKPSPPTHLPPPIYHKPSPPVHLPPPVYHKP
SPPTHLPPPVYHKPSPPSHLPPPVYHKPSPPTHLPPPVYHKPSPPTHLPPPVYHKPSPPT
HLPPPVYHKPSPPTHLPPPIYHKPSPPTHLPPPTHTSPPIYHKPTPPAPRLPPIIYRPPS
IHPPATNFTQTFLSSFTFTAAPFT

>TC|CV301500||Amaranthaceae_B.vulgaris||EXTB
MGKLGGMASLVATLLVAFVSLSLPAQTIADYTYSSPPPPVHHEMPPKGHYSPLPPTPVYK
SPPVHTYPPPSPIYKSPPVHEYPPPTPVYKSPPVHKYPPPTPVYKSPPVHKYPPPTPVYK
SPPVHKYPPPTPVYKSPPVHEYPPPTPVYKSPPHTPIYKSPPVSHPTPSPYVYASPPPPH
HY

>TA|EL402234||Asteraceae_C.tinctorius||EXTB
MRTKGHRMATPLITLAVVIVSLSLPSLTTATYPYSSPPPPPPKKSPPPPTPHYVYKSPPP
PPPLYKSPPPPVYKSPPPPTPVYKSPPPPNKPYVYKSPPAPTPVYNIPPPPNNKASWKER
TVNNQVLRKKSSNKCCIFLQKKPFNKDTHDHEHDDYHHDNDNHHDHNYKHLLRLWIEKRG
RLKRPRPRLFLTRYFTNLQIVKPTKEAKTFGDISLCVYNS

>TC|NP524794||Asteraceae_H.annuus||EXTB
MKMSSITTLLITLAVVIVSLTLPSPTTATYHYSSPPPPPPKKSPPPPPKQQYVYKSPPPP
PVYKSPPPPVYKSPPPPVHKSPPPPVHKSPPPPVYKSPPPPKKPYVYKSPPPPPPVHKSP
PPPVYKSPPPPVYESPPPPVYKSPPPPVYKSPPPPVHKSPPPPVYKSPPPPKKPYVYKSP
PPPPPVHKSPPPPVYKSPTPPVHKSPPHPVYKSPLPVHKSPPPVYKSPPPPKKPYVYKSP
PPPVKKHPPPHYIYSSPPPPYHH

>TA|TA1913_43195||Asteraceae_L.perennis||EXTB
METKRKMCFNRAPLFISLVVVMISLSLPSSTTAAYPYSSPSPPPPKKSPPPPPSKHHYVY
KSPPPPPPVYKSPPPPVHKSPPPPEHKSPPPPVYKSPPPPVYKSPPPPTPVYKSPPPPKK
PYVHKSPPPPVYKSPPPPLHKSPPPPVYKSPPPPVHKSPPPPVYKSPPPPPVHKSSPPPT
PVYKSSPPPPKKHYVYKSPPPPPPVYKSPPPPSPKKPYVYKSPPPPTPVHKSSPPPTPVY
KSPPPPTPVYKSPPTPVKKYPPPHY

>TA|TA1819_75948||Asteraceae_L.saligna||EXTB
MGTKRKMSSTATLLISLAVVIVSLCLPSLTTAAYPYSSPPPPPPKKSPPPPPKHHYVYKS
PPPPPPVYKSPPPPVYKSPPPPVHKSPPPPTPIYKSPPPPKKPYVYKSPPPPPDHKSPPP
LVYKSPTPPVHKSSPPPVYKSPPPPVHKSPPPPVYKSPPPPVYKSPPPPVHKSPPPPVYK
SPPPPVYKSPPPPVHKSPPPPVYKSPPPPVHKSPPPPHYVYKSPPPPVYKSPPPLVHKSP
PPPVYKSPPPPLHKFPPPPK

>TC|TC27580||Asteraceae_L.sativa||EXTB
MSSSLSSFSSFFSSAAGASAAAAPPTPVYKSPPPPKKPYVYKSPPPPPPVHKSPPPPVYK
SPPPPAYKSPPPPVHKSPPPPVYKSPPPPVYKSPPPPHYVYKSPPPPVYKSPPPPVHKSP
PPPVYKSPPPPVHKSPPPPVYKSPPPPVHKSPPPPVYKSPPRPLHKSPPPPPPVHKSPPP
PVYKSPPPPAYKSPPPPVHKSPPPPVYKSPPPPVYKSPPPPHYVYKSPPPPVYKSPPP

>TA|DW172847||Asteraceae_L.virosa||EXTB
MGTKVKMSQMSSLLVSLAVVIISSYLPSFSTATYPYSSPPPPKQEYAYNSPPPRPPNEPY
KSPPPPPPVHNSPPPPPPKGHYVYKSPPPPPPPQVYKSPHSSPPKKHHVHKSPPPPPWVD
KSPPPPPPKKPYVYKSPPPPPPKKHHVHKSPPPPPPVHKSPPPPPP

>NR|gi:228065886||Boraginaceae_L.erythrorhizon||EXTB
MGTYTKGSSKMASLVTTLLVLVIAFTIPSETSANYKYSSPPPPKKYSPPPHHYHHKSPPP
PVYKSPPPPMHKSPPPPVYKSPPPPMHKSPPPPKKYSPPPPVYKSPPPPMHKSPPPPKKY
SPPPPVYKSPPPPMHKSPPPPKKYSPPPPVYKSPPPPMHKSPPPPKKYSPPPPVYKSPPP
PMHKSPPPPKKYSPPPPVHKPPPHWSHKYSPPPPVHKSPPHHYRYNLLHLPITLRFKEFI
FKEQDNN

>LOC|AT1G76930||Brassicaceae_A.thaliana||EXTB||EXT1,EXT4
MGAPMASFLVLAFSLAFVSQTTANYFYSSPPPPVKHYSPPPVYKSPPPPVKHYSPPPVYK
SPPPPVKHYSPPPVYKSPPPPVKYYSPPPVYKSPPPPVYKSPPPPVKHYSPPPVYKSPPP
PVKHYSPPPVYKSPPPPVKHYSPPPVYKSPPPPVKHYSPPPSYTTLHHHRFTTHLLQSYT
TLHHHRFTTHLLQLYTTPHHHPRSTTNTNLLLLRTPLLTSTPTLPLQITTSSSLLDYLIV
SNGVDDDTQENKESFGDIIKDTRSLTGELKRIHMSKEKVSHFNVFSLFINTLL

>TC|TC135050||Fabaceae_M.truncatula||EXTB
MKKMRSLMASASLTLALAILFLSFPSEISANKYAYSSPPPPHYSSPPPPPVHTYPKPVYH
SPPPPVHTYPKPVYHSPPPPVHKYPHPKPVYHSPPPPVHKYPHPHPVYHSPPPPVHKYPH
PHPVYHSPPPPPPKKSYKYPSPPPPPVHTYPKPVYHSPPPPVHHTYPKPVYHSPPPPVHT
YPHPKPVYHSPPPPVHTYPKPVYHSPPPPVHTYVPHPKPVYHSPPPPVHTYPPHVPHPVY
HSPPPPVHSPPPPHYYYKSPPPPYHN

>NR|gi:15021744||Fabaceae_P.sativum||EXTB
MRSLMASASLTLALAILFFSFPSEISANQYSYSSPPPPVHSPPPPKDPYHYSSPPPPPVH
TYPHPHPVYHSPPPPVHTYPHPHPVYHSPPPPTPHKKPYKYPSPPPPPVHTYPHPHPVYH
SPPPPHKKPYKYSSPPPPPVHTYPHPHPVYHSPPPPVHTYPHPHPVYHSPPPPPTPHKKP
YKYPSPPPPPAHTYP

>TC|GD101099||Solanaceae_C.annuum||EXTB
MGKMASLVATLLVVLVSLSLASESSANYHYSSPPPPKKPYHPSPTPYHPAPVHKSPPPPT
PVYKSPPPPKKPHYPPHTPVYKSPPPPKKPHYPPHTPVYKSPPPPTPVYKSPPHYPPHTP
VYKSPPPPTPVYKSPPPPKRPYYPPPTPYHPHTNL

>TC|TC19464||Solanaceae_C.annuum||EXTB
MASLVATLLVVLVSLSLASESSANYHYSSPPPPVHVYPSPPHHPVYKSPPPNHHHPVYKS
PPPHHHHPVYKSPPPPKKPYHPSPTPYHPAPVHKSPPPPTPVYKSPPPPKKPHYPPHTPV
YKSPPPPKKPHYPPHTPVYKSPPPPTPVYKSPPHYPPHTPVYKSPPPPTPVYKSPPPPKK
PYYPPHTPVYKSPPPPTPVYKSPPPPKRPYHPPPTPYHPTPVYKSPPPPTPVYKSPPSPV
KPYHPAPVYKSPPPPTPVYQSPPPPVKSYHPAPVYKSPPPPTPVYKSPPPVNPYHPSPTP
HHPAPVYKSPPPPTPVYKSPPPPYHH

>NR|gi:296617||Solanaceae_N.tabacum||EXTB
MGKMASLFASLLVVLVSLSLASESSANYQYSSPPPPKKSYLYKSPPPPVHVYPSPPHHPV
YKSPPPPKKPYYPPHTPVYKSPPPPTPVYKSPPPPKKPYYPPHTPVYKSPPPPKKPYYPP
HTPVYKSPPPPTPVYKSPPPPKKPYYPPHTPVYKSPPPPTPVYKSPPPHKKPYYPPHTPV
YKSPPPPKKPYYPPHTPVYKSPPPPTPVYKSPPPPKKPYYPPHTPVYKSPPPPTPVYKSP
PPPKKPYYPPYTPVYKSPPPPTPVYKSPPPPKKPYYPSPTPYHPAPVYKSPPPPTPVYKS
PPPHHPYVYASPPSPYHY

>NR|gi:296401||Solanaceae_S.tuberosum||EXTB
MGKMASLVATLLVVLVSLSLASESSANYQYSSPPPPVHVYPSPPHHPVYKSPPPHHHHPV
YKSPPPSEKPHYPPHTPVYKSPPPHHHHPVYKSPPPPTPVYKSPPPPKTPHYPPHTPVYK
SPPPHHHHPVYKFPPPPTPVYKSPPPPKDPHYPPHTPVYKSPPPPTPVYKSPPPPTPVYK
SPPPPTPVYKSPPPPVKPYHPAPVYKSPPPPTPVYKSPPVKPYHPAPVYKSPPPPTPIYK
SPPPPVKPYHPSPTPYHPKPVYKSPPPPTPVYKSPPPTHYVYSSPPPPYHY

**@ EXTC Class [9 Proteins, 7 Species]**

>TA|TA224_15368||Poaceae_B.distachyon||EXTC
MGGKAVLLVALLAVSLVVQIQADSGYTPTPVTPSPKPEKPPKGHKPPHHHHHAKPPAGSH
KPAPPTATPPTPAYKPPTATPPAPTPPKYTPSPKPPTPKPTPPTYTPTPKPPTPTPKPTP
PTYKPAPKPTPPTYKPTPKPSPPTYPPKPTPPTYKPAPKPTPPTYKPAPKPTPPTYKPAP
KPTPPTYKPDPKPTPPTYKPAPKPAPKPTPPNYKPAPKPTPPSYKPAPKPTPPAPKPTPP
TPPAYKPAPKPSPPTPAPPAYKPPTPTPPAYKTPTPSPPPPPYH

>NR|gi:38605837||Poaceae_O.sativa||EXTC
MGGKAALLMALVAISVVLEARADAGGYGGGYTPTPTPVKPAPKPEKPPKEHKPPHHHEPK
PEKPPKEHKPPAYTPPKPTPTPPTYTPTPKPTPPPYTPKPTPPAHTPTPPTYTPTPTPPK
PTPPTYKPQPKPTPAPYTPTPTPPTYKPQPKPTPPPTYKPQPKPTPTPYTPTPTPPSYKP
QPKPTPTPYTPTPTPPSYKPQPKPTPTPYTPTPTPPSYKPQPKPNPPPTYKPQPKPNPPP
TYKPAPPTYKPQPKPNPPPTYKPQPKPTPTPYTPPTYKPQPKPTPTPTPYTPTPKPNPPP
TYKPQPKPTPTPTPYKPQPKPTPSPYTPKPTPTPPTYTPTPTPPYHKPPPSYTPGPPPPY


>TC|TC46636||Poaceae_P.virgatum||EXTC
MGGKAALLLALVAVSLAVEIHADAGYGYGGGYGPTPTPTPATPTPKPEKPPKGGKPPKEH
GPKPEKPPKGDKPEKPPKGEKPPKPEKPPKGEKPEKPPKEHKPPSYTPSPKPTPPTYTPT
PKPAPPTPKPTPPTYTPSPKPTPPTYTPTPKPPAYTPTPKPTPPTYAPTPKPPTKPPTYT
PTPKPTPPTYSPSPKPKPTPTPPTPKPTPPTYTPSPKPKPKPTPPTYTPSPKPKPKPTPT
PTPKPTPPTYTPSPKPKPTPTPTPKPTPPTYTPTPKPTPPTYKPAPKPTYKPAPKPTPPV
SYTPSPPPPYYK

>TC|TC3784||Poaceae_P.virgatum||EXTC
MGGKAALLLALVAVSLAVEIHADAGYGGGYGPTPATPTPKPEKPPKGGKPPKEHKPPTYT
PSPKPTPPTYTPTPKPAPPTPKPTPPTYTPTPKPAPPTYTPTPKPPATKPPTPKPTPPTY
TPSPKPTPPTYTPTPKPPTYTPKPKPTPPTYTPKPKPTPPTYTPSPKPKPKPKPTPPTYT
PTPKPTPPTYTPSPKPKPTPPTYTPTPKPTPPTYTPSPKPKPTPPTYTPTPKPTPPTYTP
SPKPTPPTYTPTPKPTPPTYTPSPKPKPTPTPKPTPPTYKPTPKPNPPPYTPTPKPTPPP
TYKPAPKPTPPTYKPAPKPTPPVSYTPSPPPPYYK

>TC|TC112138||Poaceae_S.officinarum||EXTC
MMGGKAALLLALVAVSLAVEIQADAGYGYGGGYPTPTPPATPTPKPQKPPTKGPKPEKPP
KEHGHKPPKEHKPTPPTYTPSPKPTPPTYTPKPTPPKPSPPKPTPPTYTPTPTKPTPKPT
PPTYTPSPKPPATKPPTYPTPKPTPPTYTPTPKPPATKPSTPKPTPPTYTPTPKPPATKP
STPKPTPPTYTPTPKPPATKPPTPKPTPPVYTPSPKPPVTKPPTSPTPKPTPPTYTPTPK
PPATKPPTYTPTPPAYKPPTTTPPATKPPAYTPKPKPTPPTYKPAPKPTPTAYKPPTPPA
YKAPPVSHTPSPPPPYY

>NR|gi:119713||Poaceae_S.bicolor||EXTC
MMGGKAALLLALVAVTLAVVEIQADAGYGYGGGYPTPTPKPPAKGPKPEKPPTKGHGHKP
EKPPKEHKPTPPTYTPSPKPTPPPATPKPTPPTYTPSPKPKSPVYPPPPKASTPPTYTPS
PKPPATKPPTYPTPKPPATKPPTPPVYTPSPKPPVTKPPTPKPTPPVYTPNPKPPVTKPP
THTPSPKPPTSKPTPPVYTPSPKPPKPSPPTYTPTPKPPATKPPTSTPTHPKPTPHTPYP
QAHPPTYKPAPKPSPPAPTPPTYTPPVSHTPSSPPPPPPPPYY

>NR|gi:22092||Poaceae_Z.diploperennis||EXTC
MGGSGTAALLLALVAVSLAVEIQADAGYGYTPTPTPATPTPKPEKPPTKGPKPEKPPKEH
KPPKEHGPKPEKPPKEHKPTPPTYTPSPKPTPPTYTPTPTPPTPKPTPPTYTPAPTPHKP
TPTPKPTPTPPTYTPSPKPPTPKPTPPTYAPSPKPPATKPPTPKPTPPTYTPSPKPPTPK
PTPPTYTPSPKPTPPTYTPSPKPPTPKPTPPTYTPSPKPPATKPPTPKPTPPTYTPSPKP
PTPKPTPPTYTPSPKPPATKPTPPTYTPSPKPPTPKPTPPTYTPSPKPPTPKPTPPTYTP
SPKPPATKPPTPKPTPPTYTPTPKPPATKPPTYTPTPPVSHTPSPPPPYY

>NR|gi:22333||Poaceae_Z.mays||EXTC
MGGSGRAALLLALVAVSLAVEIQADAGYGYGGGYTPTPTPATPTPKPEKPPTKGPKPDKP
PKEHKPPKEHGPKPEKPPKEHKPTPPTYTPSPKPTPPTYTPTPPPTPKPTPPTYTPAPTP
HKPTPKPTPTPPTYTPSPKPPTPKPTPPTYTPSPKPPATKPPTPKPTPPTYTPSPKPPTP
KPTPPTYTPSPKPPTPKPTPPTYTPSPKPPTPKPTPPTYTPSPKPPATKPPTPKPTPPTY
TPSPKPPTPKPSPPTYTPSPKPPTPKPTPPTYTPTPKPPATKPPTYTPTPPVSHTPSPPP
PYY

>NR|gi:257041||Poaceae_Z.mays||EXTC
MGGSGRAALLLALVVVAVSLAVEIQADAGYGYGGGYTPTPTPATPTPKPEKPPTKGPKPD
KPPKEHGPKPEKPPKEHKPTPPTYTPSPKPTPPTYTPTPTPPKPTPPTYTPAPTPHKPTP
KPTPTPPTYTPTPKPPTPKPTPPTYTPSPKPPTPKPTPPTYTPSPKPPTPKPTPPTYTPS
PKPPATKPPTPKPTPPTYTPSPKPPTPKPTPPTYTPSPKPPTPKPTPPTYTPSPKPPTHP
TPKPTPPTYTPSPKPPTPKPTPPTYTPSPKPPTPKPTPPTYTPSPKPPTPKPTPPTYTPT
PKPPATKPPTYTPTPPVSHTPSPPPPYY

**@ EXTD Class [8 Proteins, 2 Species]**

>TC|TC33633||Funariaceae_P.patens||EXTD
MRRFQTLTLLAALLLLVVCSCSAARILEENHAAPQMKFKKGMMHHPPAPKLSLPPLPSIP
SFGKKCPPPPAPPVVESPPYVSPPSPVYMSPPYSPSPVYKSPPSPY

>TC|TC33610||Funariaceae_P.patens||EXTD
MKGFQALVLIGTLMLVACSCSAARILVEENRAASQMKFKGPSPTEKPKLPPLPTLPPKPK
LTLPPQPKLPSFGKKCPPPPASPVYTSPPYSPSPVYTSPPYSPSPVYKSPPPY

>TC|TC35495||Funariaceae_P.patens||EXTD
MKGFQVLRLVVAILVISSSLSLARVLDDGISNQKFKLPTLPSCPPPPESPVYESPPYASP
SPVYESPPYSPSPVYESPPSPTYSPSPVYKSPTYSPSPVYKSPPSPSYSPSPVYKSPPAG
GY

>TC|TC30811||Funariaceae_P.patens||EXTD
MKRFQVLTLVVALLVVSSRFSFARVLDDVNANQKFKLPKLPTLPSCPPPPESPVYESPPY
ASPSPVYESPPYSPSPVYKSPPSPTYSPSPVYKSPPSPTYSPSPVYKSPPSPTYSPSPVY
KSPPSPTYSPSPVYKSPPSPSYSPSPVYKSPPAGGY

>TC|TC42086||Funariaceae_P.patens||EXTD
MTAAPAMTVALLLVALLSCALPSEAGRALLTLGSQKNLKSLLPGCSPPPAPVDTPSYSPP
TYTPESPPYTPSYSPSPVYESPPVDQSPSYSPGSPSYSPPSTGYQTPPTYSPPLYLSPPT
YSPSPVYSSPPPYSPSPVYSSPSPVYKSPPVYQSPPAGGY

>jgi|Phypa1_1|97861|fgenesh1_pg.scaffold_336000032||Funariaceae_P.patens||sp2y,vyx-containing
MKGFQALVLIGTLMLVACSCSAARIFVEENRATRHMKFKNGLSPTEKPKLPPLPTLPPNP
ELTLPPQPKLPSFGKKCPPPSPVYESPPYSPSPVYTSPPYSSSHVYSHCRISSGHVLRVN
GGCDEAISFFNEALRESEGDYAELREFVYGREQCQLLRGQTMDVPRMLMG

>TA|CN205527||Pottiaceae_T.ruralis||EXTD
MKSSQALVLAMVLFSSCTIFTSARLLDESAFGEAKFKGHKHGLPKLPTLPSLPSCPPPPE
SPAPVYESPPYGSPSPVYDSPPYASPSPVYESPPYASPSPVYESPPYASPSPVYESTTNA
SHINRVQVQ

>TA|TA1249_38588||Pottiaceae_T.ruralis||EXTD
MKSAMALLVAALALLSSVLPADAGRAMLTYGTPTYTPPTYTPESPPYVPAYSPVYAPPVY
TPPVYTPPVYSSPPVVYSPSPVYESPPVTYSPPAPVYESPPVTYSPSPVYESPPVTYSPP
APVYESPPVVSSPSPVYESPPVTYSPPAPVYESPPVVSSPSPVYESPPVTYSPPAPVYES
PPVYTPPVVPSPPAGGY

**@ EXTM Proteins [10 Proteins, 6 Species]**

>TA|TA559_75948||Asteraceae_L.saligna||sp2,3-misc-containing
MRGSFLLLFLLVSGCLCYGNAKNVEVVGIGECADCKENNIDTIHAVSGLKVTIDCKLQDG
KFKTRGVGKLNEEGHFKISLPQEILKDEKLGEECYVQLHNAANAPCAIHSGLEASKISFL
SKSDKTHTFGPNGKLKFSSAVCTSAFFWPGYKHPSIPKPSFPKEHPWLKKFGHIFKKPCP
PLPPKILPPVPTIPIKKPCPPPVPVYKPTPKPEPPVYKPEPKPTPPVYKPEPKPTPPVYK
PKPTPPVYK

>TA|TA287_75947||Asteraceae_L.virosa||sp2,3-misc-containing
MRGSFILLFLLVSGCLCYGNAKNVEVVGIGECADCKENNIDTIHAVSGLRVTIDCKLEDG
KFKTRGVGKLNEEGHFKISLPQEILKDEKLGEECYVQLHNAANAPCAIHSGLEASKISFL
SKSDKTHTFGPTGKLKFSSAVCTSAFFWPGYKHPSIPKPSFPKDHPWLKKFGHIFKKPCP
PLPPKILPPVPTIPIKKPCPPPVPVYKPTPKPEPPVYKPEPKPTPPVYKPEPKPTPPVYK
PKPTPPVYKPEPKPTPPVYKP

>LOC|AT3G62680||Brassicaceae_A.thaliana||EXTM||PRP3
MAITRSSLAICLILSLVTITTADYYSPSSPPVYKSPEHKPTLPSPVYTPPVYKPTLSPPV
YTKPTIPPPVYTPPVYKHTPSPPVYTKPTIPPPVYTPPVYKPTLSPPVYTKPTIPPPVYT
PPVYKPTPDYTKPTIPPPVYTPPVYKPTPSPPVYKKSPSYSSPPPPYVPKPTYTPTTKPY
VPEILKAVDGIILCKNGYETYPILGAKIQIVCSDPASYGKSNTEVVIYSNPTDSKGYFHV
SLTSIKDLAYCRVKLYLSPVETCKNPTNVNKGLTGVPLALYGYRFYPDKNLELFSVGPFY
YTGPKAAPATPKY

>LOC|AT1G54970||Brassicaceae_A.thaliana||EXTM||PRP1
MAITRASFAICILLSLATIATADYYAPSSPPVYTSPVNKPTLPPPVYTPPVHKPTLPPPV
YTPPVHKPTLSPPVYTKPTLPPPAYTPPVYNKPTLPAPVYTPPVYKPTLSPPVYTKPTLL
PPVFKPTLSPPVYTKPTLSPTVYKPTLSPPVNNKPSLSPPVYKPTLSPPVYTKPTLPPPV
YKKSPSYSPPPPFAPKPTYTPPTKPYVPEIIKAVGGIILCKNGYETYPIQGAKAKIVCSE
RGSYEKSKNEVVIYSDPTDFKGYFHVVLTHIKNLSNCRVKLYTSPVETCKNPTNVNKGLT
GVPFSMYSDKNLKLFNVGPFYFTAGSKAAPATPRY

>LOC|AT2G27380||Brassicaceae_A.thaliana||EXTM||EPR1
MRVPLIDFLRFLVLILSLSGASVAADATVKQNFNKYETDSGHAHPPPIYGAPPSYTTPPP
PIYSPPIYPPPIQKPPTYSPPIYPPPIQKPPTPTYSPPIYPPPIQKPPTPTYSPPIYPPP
IQKPPTPTYSPPIYPPPIQKPPTPSYSPPVKPPPVQMPPTPTYSPPIKPPPVHKPPTPTY
SPPIKPPVHKPPTPIYSPPIKPPPVHKPPTPIYSPPIKPPPVHKPPTPTYSPPVKPPPVH
KPPTPIYSPPIKPPPVHKPPTPIYSPPVKPPPVQTPPTPIYSPPVKPPPVHKPPTPTYSP
PVKSPPVQKPPTPTYSPPIKPPPVQKPPTPTYSPPIKPPPVKPPTPIYSPPVKPPPVHKP
PTPIYSPPVKPPPVHKPPTPIYSPPVKPPPIQKPPTPTYSPPIKPPPLQKPPTPTYSPPI
KLPPVKPPTPIYSPPVKPPPVHKPPTPIYSPPVKPPPVHKPPTPTYSPPIKPPPVKPPTP
TYSPPVQPPPVQKPPTPTYSPPVKPPPIQKPPTPTYSPPIKPPPVKPPTPTYSPPIKPPP
VHKPPTPTYSPPIKPPPIHKPPTPTYSPPIKPPPVHKPPTPTYSPPIKPPPVHKPPTPTY
SPPIKPPPVHKPPTPTYSPPIKPPPVHKPPTPTYSPPIKPPPVHKPPTPTYSPPIKPPPV
QKPPTPTYSPPVKPPPVQLPPTPTYSPPVKPPPVQVPPTPTYSPPVKPPPVQVPPTPTYS
PPIKPPPVQVPPTPTTPSPPQGGYGTPPPYAYLSHPIDIRN

>TC|ES906800(revision)||Brassicaceae_B.napus||EXTM
MLSTMAIKSTSLAICLFFSLATIATAYYSPSSPPVHQSPEYKPTLSPPVYIPKPTLPPPV
YTPPVYKPTLPPPVYTPPVYKPTLPPPVYKKSPSYPHPSYVPKPTYTPPTKPYVPKPTYS
PPTKPYVPKPTYTPPTKPYVPKPTYTPPTKPYVPKPTYSPPTKPYVPKPTYTPPTKPYVP
KPTYTPPTKSYVPKPTYTPPTKPYVSKPTYTPPTKPYVPEILKVVDGIILCKNGYETYPI
QGAKAMIVCSEPGSYGKKDVVIYSDPTDSKGYFHVALTDIIKNLLHCRVKLYTSPV

>TC|TC101194||Brassicaceae_B.napus||EXTM
MRVPLINILSFFLIILFLSGSLFATGETVNQNSRYETDNYGYTPPSPTYGAPPSQPTPPT
YSPPIVPPPVQKPPTPTYSPPVKPPPVKPPTPIYSPPVMPPPVQQPPTPSYSPPVKPPPV
QKPPTPTYSPPVKPPPVQKPPTPTYSPPIKPPPVQKPPTPTYSPPIKPPPVQKPPTPTYS
PPVKPPPVQKPPTPTYSPPIKPPPVQKPPTPTYSPPIKPPPVQKPPTPTYSPPIKPPPVQ
KPPTPTYSPPVKPPPVNPPTPIYSPPIKPPPVQQPPTPSYSPPVKPPPVQKPPTPTYSPP
VKPPPVQKPPTPTYSPPVKPPPIQKPPTPIYSPPIKPPPVHHPFIDLRRLVLTLRVSDTT
LYSPQ

>TC|TC106349||Brassicaceae_B.napus||EXTM
MRVPLISILSFFLIILFLSGSLVATGETVNQNSRYTTDNYGYTPPSPTYEAPPSQPTPPT
YSPPIVPPPVQKPPTPIYSPPVTPPIQKPPTPIYSPPVKPPPVQKPPTPTYSPPVKPPPV
QKPPTPIYSPPIKPPPVQKPPTPIYSPPVKPPPVKPPTPIYSPPIKPPPVQQPPTPTYSP
PVKPPPVKPPTPTYSPPVKPPPVQKPPTPTYSPPVKPPPVKPPTPTYSPPVKPPPVQKPP
TPTYSPPIKPPPVQKPPHIKPPPVHKPPHIKPPPVQKPPHIKPPPVKKPPHIKPPPVQKP
PTPIYSPPIKPPPVQKPPPIKPPPVEKPPTPTYSPPIKPPPVQKPPTPTYSPPVKPPPMK
PPTPTYSPPVKPPPVQQPPTPTYSPPIKPPPVKPPTPTYSPPIKPPPVQQPPTPTYSPPV
KPPPVKPPTPIYSPPVIPPPIQKPPTPTYSPPILPPPVQKPPTPIYSPPQVGYGTPPPYA
HLSQP

>LOC|AC234701_2||Fabaceae_M.truncatula||EXTM||Plant lipid transfer/seed storage/trypsin-alpha amylase inhibitor
MGLKGVGSIFILFLHLIFVSTLVSAKSAPRRPPPPPQVTCSSTPPPVVAPSTPPSTPPIT
VAPSTSPVAVDPTTLPPSTPPSTPPTSTPPNVVAPSTPPPSTPPFTTPPTVAPSTPPLVH
APSTPPPSTPPSTPPTAIAPSTPQVVIDPTTLPPSTTHSTPPSVDAPSSPPSTPPTTVAP
STPPSLVDPTTPPPSTPPSTPPPVHDPSTPPSTPPIAVAPSTPPSVVNPTIPPPSTPPTT
PPPNPRTPPTNPRTPPTNPRTPPTTPPPNPRTPPTTPPTNPRTPPKNPMTPPTIPPPNPR
TPPTTLPPNPRTPPTTPPANPRTPPTRPPTNPRTPPTTSPPNPRTPPTNPMTPPTTPPAN
PRTPPTTPPTNPRTPPTTPPTNPRTPPTTPPPNPRTPPTNPMKPPTTPPPNPMTPPSTPP
PNPRTPPTTPSPNPRTPPTNPTTPPTNPRTPPTTPPTNPRTPPTIPPPIPRTPPTTPPPN
PRTPPTNPRTPPTTPPPNPRTPPSTPPPIPRTPPFTPPPIPRTPPTTPPSIPTTPPRNCP
LLSLNVCANLLNKFVINPGSNPCCSLISGLVDLDASVCLCTALKANVLGIIRPEINVDLE
VILNRCGRKATNYICRR

>TC|TC131960||Salicaceae_P.trichocarpa||EXTM
MAFKAVCLMVVAFVLVTAKASYMNEDFKEKAVYSKSVVPASTPAPPEVKSPTPAPPVVTP
STPLYKPPTPAPPVKTPPPAPPVNPPTPVKPPTTPAPPVYKPPSPAPPVNPPTPVPPVKP
PTTPAPPXXXXPSPAPPPVPPVKPPTT

**@ HEXA Class [15 Proteins, 7 Species]**

>LOC|AT3G22800||Brassicaceae_A.thaliana||HEXA||LRX6
MREDTFFFQWWFLVSGLSFIFLLPQAFTYHTPPINPCFAHPFLPPITNPRLLKAFTALQA
WKFTITSDPNGFTSNWCGPNVCNYTGVFCAPALDNPYVLTVAGIDLNHANIAGYLPLELG
LLTDLALFHINSNRFQGQLPKTLKCLHLLHELDVSNNKLSGEFPSVIFSLPSLKFLDIRF
NEFQGDVPSQLFDLNLDALFINDNKFQFRLPRNIGNSPVSVLVLANNDLQGSCVPPSFYK
MGKTLHEIIITNSQLTGCLNREIGLLNQLTVFDVSYNNLVGSLPETIGDMKSLEQLNIAH
NKFSGYIPESICRLPRLENFTYSYNFFSGEPPACLRLQEFDDRRNCLPSRPMQRSLAECK
SFSSYPIDCASFGCSPPSPPPPPPPPPPPPPPPPPPPPPPPPPPPPPYVYPSPPPPPPSP
PPYVYPPPPPPYVYPPPPSPPYVYPPPPPSPQPYMYPSPPCNDLPTPVHY

>LOC|AT4G33970||Brassicaceae_A.thaliana||HEXA||PEX4
MPFYKQPWVFSKVFVLAMAKPPSFGCCFFLLFFSFLSSSFVSFALTDTEAAFIVQRQLLT
LPDNGELPDDIEYEVDLKATFANTRLKRAYIALQAWKKAIFSDPFNTTGNWHGPHVCGYT
GVVCAPALDDSDVTVVAGVDLNGADIAGHLPAELGLMTDVAMFHLNSNRFCGIIPKSFEK
LKLMHEFDVSNNRFVGPFPNVVLSWPDVKYFDLRFNDFEGQVPPELFKKELDAIFLNDNR
FTSVIPESLGESPASVVTFANNKFTGCIPKSIGNMKNLNEIVFMDNDLGGCFPSEIGKLS
NVTVFDASKNSFIGRLPTSFVGLTSVEEIDISGNKLTGLVPHNICQLPNLVNLTYSYNYF
SGQGGSCVPGGSRKEIALDDTRNCLASRPEQRSAQECAVVINRPVDCSKDKCAGGSSTPS
KPSPVHKPTPVPTTPVHKPTPVPTTPVQKPSPVPTTPVQKPSPVPTTPVHEPSPVLATPV
DKPSPVPSRPVQKPQPPKESPQPDDPYDQSPVTKRRSPPPAPVNSPPPPVYSPPPPPPPV
HSPPPPVHSPPPPPVYSPPPPPPPVHSPPPPVFSPPPPVYSPPPPVHSPPPPVHSPPPPA
PVHSPPPPVHSPPPPPPVYSPPPPVFSPPPSQSPPVVYSPPPRPPKINSPPVQSPPPAPV
EKKETPPAHAPAPSDDEFIIPPFIGHQYASPPPPMFAGY

>LOC|AT1G12040||Brassicaceae_A.thaliana||HEXA||LRX1
MLFPPLRSLFLFTLLLSSVCFLQIKADHDDESDLGSDIKVDKRLKFENPKLRQAYIALQS
WKKAIFSDPFNFTANWNGSDVCSYNGIYCAPSPSYPKTRVVAGIDLNHADMAGYLASELG
LLSDLALFHINSNRFCGEVPLTFNRMKLLYELDLSNNRFVGKFPKVVLSLPSLKFLDLRY
NEFEGKIPSKLFDRELDAIFLNHNRFRFGIPKNMGNSPVSALVLADNNLGGCIPGSIGQM
GKTLNELILSNDNLTGCLPPQIGNLKKVTVFDITSNRLQGPLPSSVGNMKSLEELHVANN
AFTGVIPPSICQLSNLENFTYSSNYFSGRPPICAASLLADIVVNGTMNCITGLARQRSDK
QCSSLLARPVDCSKFGCYNIFSPPPPTFKMSPEVRTLPPPIYVYSSPPPPPSSKMSPTVR
AYSPPPPPSSKMSPSVRAYSPPPPPYSKMSPSVRAYPPPPPPSPSPPPPYVYSSPPPPYV
YSSPPPPPYVYSSPPPPPYVYSSPPPPYVYSSPPPPYVYSSPPPPPPSPPPPCPESSPPP
PVVYYAPVTQSPPPPSPVYYPPVTQSPPPPSPVYYPPVTNSPPPPSPVYYPPVTYSPPPP
SPVYYPQVTPSPPPPSPLYYPPVTPSPPPPSPVYYPPVTPSPPPPSPVYYPPVTPSPPPP
SPVYYPSETQSPPPPTEYYYSPSQSPPPTKACKEGHPPQATPSYEPPPEYSYSSSPPPPS
PTSYFPPMPSVSYDASPPPPPSYY

>LOC|AT4G13340||Brassicaceae_A.thaliana||HEXA||LRX3
MKKTIQILLFFFFLINLTNALSISSDGGVLSDNEVRHIQRRQLLEFAERSVKITVDPSLN
FENPRLRNAYIALQAWKQAILSDPNNFTSNWIGSNVCNYTGVFCSPALDNRKIRTVAGID
LNHADIAGYLPEELGLLSDLALFHVNSNRFCGTVPHRFNRLKLLFELDLSNNRFAGKFPT
VVLQLPSLKFLDLRFNEFEGTVPKELFSKDLDAIFINHNRFRFELPENFGDSPVSVIVLA
NNRFHGCVPSSLVEMKNLNEIIFMNNGLNSCLPSDIGRLKNVTVFDVSFNELVGPLPESV
GEMVSVEQLNVAHNMLSGKIPASICQLPKLENFTYSYNFFTGEAPVCLRLPEFDDRRNCL
PGRPAQRSPGQCKAFLSRPPVNCGSFSCGRSVSPRPPVVTPLPPPSLPSPPPPAPIFSTP
PTLTSPPPPSPPPPVYSPPPPPPPPPPVYSPPPPPPPPPPPPVYSPPPPPPPPPPPPPVY
SPPPPSPPPPPPPVYSPPPPPPPPPPPPVYSPPPPPVYSSPPPPPSPAPTPVYCTRPPPP
PPHSPPPPQFSPPPPEPYYYSSPPPPHSSPPPHSPPPPHSPPPPIYPYLSPPPPPTPVSS
PPPTPVYSPPPPPPCIEPPPPPPCIEYSPPPPPPVVHYSSPPPPPVYYSSPPPPPVYYSS
PPPPPPVHYSSPPPPEVHYHSPPPSPVHYSSPPPPPSAPCEESPPPAPVVHHSPPPPMVH
HSPPPPVIHQSPPPPSPEYEGPLPPVIGVSYASPPPPPFY

>LOC|AT1G62440||Brassicaceae_A.thaliana||HEXA||LRX2
MLLFPSTSLRLFFFLFLLFSSCFLQIRGDDDDDDISDDNIKVDPSLKFENPSLRQAYIAL
QSWKQAIFSDPFNFTANWNGSDVCSYNGIFCAPSPSSPKTRVVAGIDLNHADMAGYLPRE
LGLLTDLALFHLNSNRFCGEVPLTFKHMKLLFELDLSNNRFVGKFPNVVLSLPSLKFLDL
RYNEFEGSIPSKLFDKELDAIFLNHNRFMFGIPENMGNSPVSALVLADNDLGGCIPGSIG
LMGKTLNEIILSNDNLTGCLPPQIGNLKNVTVFDISFNRLSGPLPSSIGNMKSLEQLNVA
NNRFTGVIPSSICQLSNLENFTYSSNFFTGDAPRCVALLGDNVVVNGSMNCIDGKEDQRS
SKECSSPASRSVDCSKFGCNNFFSPPPPSFKMSPTVRVLPPPPPSSKMSPTFRATPPPPS
SKMSPSFRATPPPPSSKMSPSFRATPPPPSSKMSPSVKAYPPPPPPPEYEPSPPPPSSEM
SPSVRAYPPPPPLSPPPPSPPPPYIYSSPPPPSPSPPPPYIYSSPPPVVNCPPTTQSPPP
PKYEQTPSPREYYPSPSPPYYQYTSSPPPPTYYATQSPPPPPPPTYYAVQSPPPPPPVYY
PPVTASPPPPPVYYTPVIQSPPPPPVYYSPVTQSPPPPPPVYYPPVTQSPPPSPVYYPPV
TQSPPPPPVYYLPVTQSPPPPSPVYYPPVAKSPPPPSPVYYPPVTQSPPPPSTPVEYHPP
ASPNQSPPPEYQSPPPKGCNDSPSNDHHYQTPTPPSLPPPYYEDTPLPPIRGVSYASPPP
PSIPYY

>LOC|AT3G19020||Brassicaceae_A.thaliana||HEXA||PEX1
MTRRTMEKPFGCFLLLFCFTISIFFYSAAALTDEEASFLTRRQLLALSENGDLPDDIEYE
VDLDLKFANNRLKRAYIALQAWKKAFYSDPFNTAANWVGPDVCSYKGVFCAPALDDPSVL
VVAGIDLNHADIAGYLPPELGLLTDVALFHVNSNRFCGVIPKSLSKLTLMYEFDVSNNRF
VGPFPTVALSWPSLKFLDIRYNDFEGKLPPEIFDKDLDAIFLNNNRFESTIPETIGKSTA
SVVTFAHNKFSGCIPKTIGQMKNLNEIVFIGNNLSGCLPNEIGSLNNVTVFDASSNGFVG
SLPSTLSGLANVEQMDFSYNKFTGFVTDNICKLPKLSNFTFSYNFFNGEAQSCVPGSSQE
KQFDDTSNCLQNRPNQKSAKECLPVVSRPVDCSKDKCAGGGGGGSNPSPKPTPTPKAPEP
KKEINPPNLEEPSKPKPEESPKPQQPSPKPETPSHEPSNPKEPKPESPKQESPKTEQPKP
KPESPKQESPKQEAPKPEQPKPKPESPKQESSKQEPPKPEESPKPEPPKPEESPKPQPPK
QETPKPEESPKPQPPKQETPKPEESPKPQPPKQETPKPEESPKPQPPKQEQPPKTEAPKM
GSPPLESPVPNDPYDASPIKKRRPQPPSPSTEETKTTSPQSPPVHSPPPPPPVHSPPPPV
FSPPPPMHSPPPPVYSPPPPVHSPPPPPVHSPPPPVHSPPPPVHSPPPPVHSPPPPVHSP
PPPVHSPPPPVQSPPPPPVFSPPPPAPIYSPPPPPVHSPPPPVHSPPPPPVHSPPPPVHS
PPPPVHSPPPPVHSPPPPVHSPPPPSPIYSPPPPVFSPPPKPVTPLPPATSPMANAPTPS
SSESGEISTPVQAPTPDSEDIEAPSDSNHSPVFKSSPAPSPDSEPEVEAPVPSSEPEVEA
PKQSEATPSSSPPSSNPSPDVTAPPSEDNDDGDNFILPPNIGHQYASPPPPMFPGY

>NR|gi:255556233||Euphorbiaceae_R.communis||HEXA
MENLLRASGCFLFFTFLFSSFSTFSFALTDAEVSFITGRQLLSLKENEELPNEYEHEVDV
KVTFANQRLKRAYIALQAWKKAMYSDPFNTTSNWNGANVCAYNGVFCAPALDDPKLSVVA
GIDLNQADIAGFLPPELGLMTDLALFHINSNRFCGIVPESLSKLKFMYEFDISNNRFVGH
FPNVVLKWPNVKYIDIRFNNFEGCLPPEIFQMNLDALILNNNRFTCNIPDTIGNSTVSVV
VFAYNNFTGCIPHSIGNMPNLNEIIFTGNNLGGCFPAEIGILSNATVLDVSNNQFVGGLA
SSFSGLKNVEQLNLANNKLTGFVSETLCSLPSLSNFTFSSNYFKGEAKSCISSSNKDVVL
DDRNNCLPDRPYQKSARICYPVVSWPVDCSKDKCSGGGGSSTPSSPRPSTPSVPTPSTPY
VPTPSTPPSTSYGPTPSTPYVPTPSTPYVPTPSTPSPSPPQQEVPTPQTPSSEPYNPSPG
GHRESPTPSPEPSKPTSPTTSPSPSPEPSEPTPPATSPSPSSEPSEPTSPTTSPSPSSEP
LESTPPTTSPSLSPEPSEPTSPTTSPSPSPEPSEPTSPTTSPSPEYNSSEPSPTPSPESE
PQSPTSTPSPESPLSSPTSPTLEQSPSPPGQSPPPTTPEQSPSPPGQSPPPPTQSPPPPV
NSPPPPVYSPPPPSPPPPVHSPPPPVYSPPPPSPPPPVHSPPPPVHSPPPPVHSPPPPVY
SPPPPSPPSPPPPMHSPPPPVYSPPPPPVRSPPPPVHSPPPPVHSPPPPIYSPPPPRFSP
PPPRFSPPPPTSSPPPTPTVAAPPPEDFILPPNIGFQYSSPPPPMFPGY

>LOC|Medtr3g148740||Fabaceae_M.truncatula||HEXA||Leucine-rich repeat, N-terminal chr03
MQVQGWFLLFSFFLFSPTSFFSHAISNEEAASIARRQMLHLQENEDLPENYVDTYKTDLK
FPNPNLKSAYIALQAWKKAIYSDPTNFTSNWEGPNVCSYNGIFCAASLNDSKIQVVAGID
LNQADIAGYIPAEFGLLADIALLHINSNRFCGVLPKSFSKLKLLHELDISNNRFVGKFPC
HVLSIPDIKFIDIRFNEFEGEIPPELFNKTLDAIFINNNRFTSTIPENMGNSPASVIVFA
NNGISGCIPSSIGQMKNLNEFVVIGNNLTGCLPEDIGKLQQLAVFDVSENLFVGALPKTL
QGLSEVEVISIAHNKLTGSVPKSICSLPNLANFTFSYNYFNVEEEGCVPPGKEIELEDMD
NCIPNRPKQKTTNDCNVVISKPVDCTKGLCSSKPSQSNSPSNPPTEKPTPSVPKPQPQPT
PSQTPTPSTPKPTPSPQPKPTPSSPPPTPSTPEAEPPIEDDPHNEAPKRRTRSPPPPVQS
SPPPVHSPSPPVHSPPPPVHSPPPPVHSPPPPVHSPPPPVHSPPPPVHSPPPPVHSPPPP
VHSPPPPPVHSPPPPVHSPPPPVHSPPPPVHSPPPPPVHSPPPPVHSPPPPVHSPPPPVH
SPPPPVHSPPPPVHSPPPPVHSPPPPVHSPPPPVFSPPPPVHSPPPPTASPPPPVNAPPP
EDDIILPPHFGSSYASPPPPIIAGY

>LOC|Medtr6g102080||Fabaceae_M.truncatula||HEXA||Leucine-rich repeat chr06
MRKKQHYSSPSYVIVLFSFFLSLTTTLSSSIVSDGSHLTEAETQYIKHRQLLYYRDEFND
RGENVTVDPTLVFSNNRIKNAYVALQAFKQAILSDPRNCTVDWVGSDVCSYTNVFCAPAL
DNPKINTVAGIDLNHCDIAGYLPEELGLLTDLALFHVNTNRFCGTVPHKFEKLKILFELD
LSNNRFAGKFPEVVLRLPELKFLDLRFNEFEGTVPKELFDKDLDAIFINHNRFVFNLPEN
FGNSPVSVIVLANNRFHGCLPAGIGNMTRLNEIIAMNNGFQACLPEEVGLLKNLTVFDVS
FNKFLGPLPEKYGNAVGLEVLNVAHNYLSGQIPASICALPNLVNFTYSYNFFTGEPPQCL
VLPAADDRQNCLPARPRQRPAKTCKAFASHHVNCNAFRCKAFVPSLPSPPPSPVFPPPVV
TPPTPVFNPPPSPVLSPPPPSPPPPVFSPPPPVYSPPPPPPPVYSPPPPPPSPPPPSPPP
PPPPVYSPPPTPPSPPPTFSPHHPPTWFIYLLPPPLIPSPPPPTSPPSPLSLPLPTPPPS
PSITPSNHPLLPTRSQSTQTSQPLPSPPANMRLIDSHDHSPFLLSPFQPTSLHPHPPLPH
RPRPSPPPILHISFPRSNRPLTACPHPPLRMTTIFQTPSISLNTSASASQYVKTHSRPYI
VSPPTRTPWLSIDSTPLAVIFITLLLACHRSPPPPPHSPPPPVYPYLSPPPPPPVHSPPP
PVYSPPPPSPPPCVEPPPPPPPPCVEPPPPSSPAPHQTPYHPPPSPSPPPSPVYAYPSPP
PPVYTSPPPSPVYAYPSPPPPVYSSPPPPPVYEGPIPPVFGISYASPPPPPFY

>NR|gi:242053469||Poaceae_S.bicolor||HEXA
MTTPPPHNTTLPVLLFLLLLLATASSSAAPFASRGGLSASDAAHIRRRQLLQYHNDDGSD
DGGVVVDASYAFPNPRLRDAYVALQAWKRAILSDPYNVTGSWSGPDVCAYGGVYCAPSPQ
DPGLTVVASVDLNHADLAGHLPEALGRLADLAVLHLNSNRFCGLVPRSLHRLHALHELDL
SNNRFVGGFPDVVLRLPSLRYLDLRFNDFEGPVPGDLFDRPLDAIFINSNRFRFRIPDNV
GNSPASVLVLANNDFGGCLPASVANMSGTLDEIILMNTGLKSCVPPELAALTGLTVLDLS
FNKLMGAIPDELASLRSIQQLDLGHNRLVGDVPEGICRLPHLQNFTYSYNFITGEPPACL
HVKTLDDRRNCIPYRPDQRSPEQCSFFNNHHVNCDAFKCKKFVLPSPPLPPPSPPPPSPP
PPSPSPPPPSPPPPSPPPPSPSPPPPSPPPPSPPPPSPPPPSPPPPSPSPPPPSPPPPSP
PPPSPSPPPPSPPPPSPPPPSPSPPPPYYEVSPEERYLSPPPPAYTEVPPPPYYEVSPED
RYLSPPPPPVHHDPPPPPYYEVSPEDRYLSPPPPSPASLPKYDYSSPPPPRALPKLPVYD
YSSPPPPAAGSTP

>NR|gi:224123244||Salicaceae_P.trichocarpa||HEXA
MANPLRAFGCFFFFSLLFSSFSTFSLALTDAEASYIARRQLLTLNENSELPHEFEYEVDV
KITFANQRLRRAYIGLQAWKKAIYSDPFNTTGNWVGANVCAYNGVFCAPALDDPSLSVVA
GVDLNGADIAGHLPAELGLMTDVALFHINSNRFCGIIPESFSKLTLMYEFDVSNNRFVGD
FPSVVLSWPSLKYLDVRFNDFEGSLPPELFNKELDALFLNDNRFTSTIPETIGNSAVSVV
TFANNKFTGCIPHSVGKMANLNEVIFMGNDLGGCFPAEIGLLRNVTVFDASHNGFTGILP
PSFAGLKKVELLDLADNKLTGFVPENICKLPSLTNFTFSYNYFKGEAQACVPPSRKDIVL
DDTSNCLSDRPKQKSARTCYPVVSRPVDCSKDKFHSPPPPVQSPPPPPPVHSPPPPVQSP
PPPVHSPPPPVHSPPPPVQSPPPPVHSPPPPVHSPPPPVHSPPPPVHSPPPPVQSPPPPV
HSPPPPVHSPPPVQSPPPP

>NR|gi:224103499||Salicaceae_P.trichocarpa||HEXA
MAEPLRVLGCFFFFSFLLSSFSNFSLALTDAEASSIARRQLLTLHENGELPDDFEYEVDV
KETFANQRLRRAYIGLQAWKKAMYSDPFNTTGNWVGADVCAYNGVFCAPALDDSGLSVMA
GVDLNGADIAGYLPAELGLLTDVALFHINSNRFCGIIPKSFSKLTLMYEFDVSNNRFVGD
FPSVVLTLPSLKYLDIRFNDFEGSLPPELFNKDLDALFLNDNRFTSTIPETIGNSPVSVV
TFANNKFTGCIPHSIGKMTNLNEVIFMGNDLGGCFPAEIGLLGNVTVFDASHNGFTGILP
SSFAGLKKVELLDLADNKLTGFVPENICRLSSLTNFTFSYNYFKGEAQACVPPSRKDTVL
DDTSNCLSDRPKQKSARTCYPVVSRPVDCSKDKCSGGGVHSPPPSSQSLPPLVHSLPPPA
HSPPPSIHFPPPPVHSPPPPPVHSPPPPIYSPPPLVYSPPPPVHSPPPPVHSPPPPVQSF
PPPVHSPPPPVHSPPPPPVYSPPPPVHSPPPPVYSPPPLVQSPPPPVHSPPPPLHSPPPP
PVYSPPPPVHSPPPPVHSPPPPIQSPPPPVHSPPPPPIHSPPPPVQSLPPPPVNSPLPPV
HSPPPPVHSPTSPIHSHPPPVNSPPPPVQSLPPPPVNSPPPPVHSPTPPIHSPSPPLHSP
PPPIRSPPPPVFSPPPVIVSPPPPPPEEDFILPPNLGFQYASPPPPTFPGY

>NR|gi:127058169||Solanaceae_N.tabacum||HEXA
MRPPSRLHSLFISFLVISLLSFQIYGQEGDIDLDDIKGDADKLIFENPKLRVAYIALQTW
KAAMFSDPFNFTANWTGPNVCSYGGVFCAQSLTNDSIRVVAGIDLNHADIAGYLVPELGL
LTDLVLFHLNSNRFCGIVPKTFSHLKLLRELDLSNNRFVGGFPKVVLSLPSLKFLDLRFN
DFEGPVPSKLFDKDLDALFLNDNRFRFGIPENLGNSPVSVLVFANNNLGGCIPASIGKMG
NSLNELILMNDNLTSCLPMEIGMLKKLTVFDVSFNKIQGPLPSTVSRMRSVEQLNVAHNK
LTGVIPASICQLPRLQNFTFSFNYFKGEAPVCAATRSGRVIDGEENCIAGKEDQRSAKEC
SSDDAKPYDCRKSQCFSRSAISPVVKPRPKPTPKPKRPPVQNKPPAPKPSPPTKPIVPIE
SPPPPSSKSSGSHFKRSPPPPQSTPPPSPPPPPPVYSSSPSHQHRSPPPPTHKISPVTRH
APPPPPPPSPVYYHHPSPPPPQPVYYAPPTYKPQSPPPPPPPVHYEPPTYTPQSPPPPPP
VHYEPPPYTPQSPPPPPVHYEPPTYTPQSPPPPVYVTPSSPPPPVYEHPKPPTPTPSPPA
GYTPPQEPSHPAPPTPPCNETPPPPPSTPHWQPKPSPPPTYNPSPSYTQSPPPPPPTYTY
SSPPPPSSSPPPPTYYYSSPPPPPPSPPPPSPSPPPPSYEHVPLPPIVGVSYASPPPPVI
PYY

>TC|TC197827||Solanaceae_S.lycopersicum||HEXA
MRPPSRGVLISFFVITLLSYQISYVVGQEGDSDIGLDDIKANASKLSFENSRTRDAYIAL
QYWKTAMFSDPFNFTANWTGPNVCSYGGVFCAPSLMDDSIRVVAGIDLNHADIAGSLVAE
LGLLTDLVLFHLNSNRFCGVVPKTFSHLKLLRELDLSNNRFVGGFPKVVLSLPSLKFLDL
RFNDFEGPVPSGLFDKDLDALFLNDNRFRFGIPENLGNSPVSVLVFANNDLGGCIPASIG
KMGKTLNELILMNDNLTGCLPMEIGLLNKLTVFDVSFNKIQGSLPSTVSKMRSVEELNVA
HNKLTGVIPASICQLPRLQNFTYSFNYFTGEAPVCAATRSGDGQENCIVGKKNQRSAKEC
SSDDAKPYDCRKSKCYSPFATSPSTKPKPKPKPRGPPPPTWKSSGSHNKRSPPPPKSTPL
PPPAPYKKSPTYQHRSPPPPTHKISPVTHHSPPPPSPVYYHPSPSSPPPPVYYSPPPPVY
HEPPPTYKPKSPPPPPTPSYEHPKTPSPLPPTPSYEHPKTPPSHEHPKTPSPPTPSYEHP
KTPSHPTPPTPPCNEPPPPPPNSHWEPKPSTPYTYSSPPPPSPSP

>TC|TC213342||Solanaceae_S.lycopersicum||HEXA
MVALGCFLFFLVSFCSFSPSYFALSDIEAASIARRQLLSNNGQLSNTYESEMTINMKFEN
ARLKKAYVALQAWKKSIYSDPTNFTANWEGSNVCAYNGVFCDNALDDPNISVVAGIDLNH
ADIAGHLPVELGLLADVSLIHINSNRFCGIIPKSITNLTLLDEIDFSNNRFVGPFPDVVL
DLPKLNYLDLRFNDFEGQVPSALFEKNLDAILINNNRFHSTIPESLGNSNASVVVLANNK
FYGCIPSSIGKMGNSLDELVFTNNELSGCLPEEITKLTSLTLLDISGNKFVGSLPQDLKS
MQKVEIFDIASNKFMGNVPKNLCTLPSLKNFTFSKNYFESMDETCRPSESKQVKIDGNEN
CLGGRSEQRTEKECFPVVSKPVDCSKGHCGVSREGQSPKDPPKTVTPPKPSTPTTPKPNP
SPPPPKTLPPPPPKTSPPPPVHSPPPPPVASPPPPVHSPPPPVASPPPPVHSPPPPPVAS
PPPPVHSPPPPVASPPPPVHSPPPPVHSPPPPVASPPPPVHSPPPPVHSPPPPVHSPPPP
VHSPPPPVHSPPPPVASPPPPVHSPPPPVHSPPPPVHSPPPPVASPPPPVHSPPPPPPVA
SPPPPVHSPPPPVASPPPPVHSPPPPVASPPPPVHSPPPPVHSPPPPVHSPPPPVASPPP
ALVFSPPPPVHSPPPPAPVMSPPPPTFEDVALPPTLGSLYASPPPPIFQGY

**@ AGPA Class [89 Proteins (68 w/ GPI anchor), 58 Species (45 w/ GPI anchor)]**

>TC|TC5406||Amaranthaceae_B.vulgaris||AGPA
MDLKATLLLGLICITLSVVGGQSPAASPSQSTQPPPTTQPPPATQPPQASQPPPATQPPQ
ASQPPPAAQPPQATQPPQASQPPPATQPPTASPPTQTTTAPPPQVAQAPQQSPPPVATPP
PVAATPPPVVSSPPPPVATPPPATPPPVSAPPPVSTPPPAATPAPLSTPPAVAPVAATPG
PSLAASPPVPLSEAPGPSLLGAASPGPANAQADD

>TA|TA463_126914||Amaranthaceae_S.salsa||AGPA
MDLKATLLLGLICITFAAVGGQQSPATSPSQQQQPPVTTQPPPQGAQPPQGAQPPPQGAQ
PPQGAQPPPATQPPQAAQPPPATQPPQAAAPPQSTQSPPPVANAPQSPPPVPATPPPVAT
PPPVATPP

>TA|EL399685||Asteraceae_C.tinctorius||AGPA
MDRNAIFLTAFICIIFSSVGGQSPSSSPTATPAPPTTTAPPPQVASTPPPVATPPPVAST
PPATPPPVASPPPATPTPVASPPPATPPPVESPPPATPPPVSSPRPITPRPEASPPPALT
SSPPATVPASSPGVTPSLRPSPLS

>TC|TC1903||Asteraceae_L.serriola||AGPA
MDRNAVFLTAFICIVISSVGGQSPSSSPTTSPPQVVASPPPTTTAPPPQVASPPTSTPPP
VAAPPTVASPPPTTPPPVASPPPATPPPVASPPPATPPPVASPPPATPPPVASPPPAPLA
SPPAPVPVSSPTPSVTPTVAPSPLLSSPPAPPTGAPSPSLATDLSPAPSATDVSGVEKMG
SMVGSIVFGCALVYSLF

>TA|TA55_121540||Asteraceae_S.aethnensis||AGPA
MDRKAAILIAFICIVFSTVGGQSPSSSPTATPAPPTTTPPPTASPPQVTATPPPVSTPPP
VVTPVSAPPPATPPPVASPPPATPPPVASPPPATPPPVASPPPATPPPVASPPAPVPVAA
PTPSVTPAVAPSPLTLSPPAPPTGAPSPSLTTDLSPAPSATDVSGVEKIGSMVGSVVFGC
ALVYSLL

>TA|DY664112||Asteraceae_S.chrysanthemifolius||AGPA
MDRKAAILIAFICIVFSTVGGQSPSSSPTATPAPPTTTPPPTASPPQVTATPPPVSTPPP
VVTPVSAPPPATPPPVASPPPATPPPVASPPPATPPPVASPPPATPPPVASPPAPVPVAA
PTPSVTPAVAPSPLTLSPPAPPTGAPSPSLTTDLSPAPSATDVSGVEKIGSMVGSVVFGC
ALVYSLL

>TA|DV038792||Asteraceae_S.squalidus||AGPA
MDRKAAILIAFICIVFSTVGGQSPSSSPTATPAPPTTTPPPTASPPQVTATPPPVSTPPP
VVTPVSAPPPATPPPVASPPPATPPPVASPPPATPPPVASPPPATPPPVASPPAPVPVAA
PTPSVTPAVAPSPLTLSPPAPPTGAPSPSLTTDLSPAPSATDVSGVEKIGSMVGSVVFGC
ALVYSLL

>LOC|AT5G65390||Brassicaceae_A.thaliana||AGPA||AGP7
MNSKIIEAFFIVALFTTSCLAQAPAPSPTTTVTPPPVATPPPAATPAPTTTPPPAVSPAP
TSSPPSSAPSPSSDAPTASPPAPEGPGVSPGELAPTPSDASAPPPNAALTNKAFVVGSLV
AAIIYAVVLA

>LOC|AT2G14890||Brassicaceae_A.thaliana||AGPA||AGP9
MARSFAIAVICIVLIAGVTGQAPTSPPTATPAPPTPTTPPPAATPPPVSAPPPVTTSPPP
VTTAPPPANPPPPVSSPPPASPPPATPPPVASPPPPVASPPPATPPPVATPPPAPLASPP
AQVPAPAPTTKPDSPSPSPSSSPPLPSSDAPGPSTDSISPAPSPTDVNDQNGASKMVSSL
VFGSVLVWFMI

>TC|TC70639||Brassicaceae_B.napus||AGPA
MVSKMVQVFLIVALFATSALAQAPAPTPTITPPAATPPPVTTPPPVATPPPVATPPPAAT
PAPATTPPPAVTPAPATTQPSAAPSPSDVPAASPPAPEGPALSPGSLAPGPSDEAPAPSA
AFSNKAFIAGTAFTAVMYAAVFI

>TC|TC97087||Brassicaceae_B.napus||AGPA
MGRQFVIVAICIVLVAGVGGQAPSSPPTATPAPPTPTTPPPAATPPPVSAPPPVATSPPP
ATTAPPPATPPPVATPPPATPPPVATPPPVATPPPATPPPVATPPPAPLASPPAQVPALA
PTTKPDAPSSSPLSSPPSPATDAPGPSIESLSPGPSTDSNDQNGATKTVSSLVLGSVLVW
FMI

>TC|TC64293||Brassicaceae_B.napus||AGPA
MARQFAIVAICIVLIAGVGGQAPSSPPTTTPAPPTTTTPPPAATPPPVSAPPPVTTSPPP
ATTAPPPATPPPVASPPPATPPPVATPPPATPPPVASPPPATPPPVASPPPATPPPAPLA
SPPAQVPALAPTTPDAPSTSPSSSPPLPATDGPGPSAEGPGPSTDSNDQNGASKTVSSLV
LGSVLVWFMI

>TC|TC68566||Brassicaceae_B.napus||AGPA
MDSSLMSWSLLLAFALISPFSVNAQGPAASPATPTISTPPPTTTVPPPTTTALPPTTTVP
PPTATVPPPTTTASPPTTVVPPVSATPPPATPTTPPPAVTPTSSPPAPKAAPVISPATPP
PQPPQSPPVSAPTVSPPPAPASPPPASPPAPTSPPPAPASPPPALTPITLPPAPAPAKHK
KKHKHKRHHHAPAPAPTPPSPPSPPVLTDSQDTAPAPSPFQNANGGNALNQLQGRVGMWL
TTVMGTLVLMAMTA

>TA|AM061600||Brassicaceae_B.oleracea||AGPA
MGRQFVIVAICIVLVAGVGGQAPSTPPTATPAPPTPTTPPPAATPPPVSAPPPVTTSPPP
ATTAPPPATPPPVASPPPATPPPVATPPPVATPPPALLASPPAQVPALAPTTKPDTPSLS
PLSSPPSPATDAPGPSTESLSPGPSTDSNDQNGATKTVSSLVLGSVLVWFMI

>TA|DY029898||Brassicaceae_B.oleracea||AGPA
MARRFAIVAICIVLIAGVGGQAPSSPPTTTPAPPTTTTPPPAATPPPVSAPPPVTTSPPP
ATTAPPPATPPPVASPPPATPPPVATPPPATPPPVASPPPATPPPVASPPPATPPPAPLA
SPPAQVPALAPTTPEAPSTSPSSSPPLPATDGPGPSVEGPGPSTDSNDQNGASKTVSSLV
LGSVLVWFMI

>TA|CV545845||Brassicaceae_B.rapa||AGPA
MGSKSVQVFLIMALFATVVLAQAPAPTPTATPPPPVATPPPVATPPPVATPPPAATPAPA
TTPPSAAPSPSDAPSASPPAPEGPASSPSGLSPGPSEDAPAPSAAFSNKAFIAGTVFAAV
MYTAVLA

>TA|BG543263||Brassicaceae_B.rapa||AGPA
MARQFAVVAICIVLIGSVRGQAPSSPPTTTPAPPTTTTPPPAATPPPVSAPPPVTTSPPP
ATPPPVSTPPPVASPPPATHPPVASPPPVATPPPAPLASPPAQVPAAAPTTKPDAPSSSP
LSSPPSPATDAPRPSTTFLSPGPSTDSNDQNGASKMVSSLVLGSVLVRFVI

>TA|CX270955||Brassicaceae_B.rapa||AGPA
MGRQFVIVAICIVLVAGVGGQAPSSPPTATPAPPTPITPPPAATPPPVSAPPPVTTSPPP
ATTAPPPATPPPVATPPPVTAPPPATPPPVATPPPAPLASPPAQVPSLAPTTKPDAPSSS
PLSSPPSPATDAPGPSIESLSPGPSTDSNDQNGATKTVSSLVLGSVLVWFMI

>TA|TA2327_3711||Brassicaceae_B.rapa||AGPA
MARQFAIVAICIVLIAGVGGQAPSSPPTTTPAPPTTTTPPPAATPPPVSAPPPVTTSPPP
ATTAPPPATPPPVASPPPATPPPVATPPPATPPPVASPPPATPPPVASPPPATPPPAPLA
SPPAQVPALAPTTPDAPSTSPSSSPPLPATDGPGPSAEGPGPSTDSNDQNGASKTVSSLV
LGSVLVWFMI

>TC|DV126386||Euphorbiaceae_E.esula||AGPA
MTHQTSLILLSLICIVISGVTAQSPATSPTATPAPPTTTPPPTSAPPPVTQSPPPAATPP
PVSAPPPATPPPATPPPATPPPATPPPATPPPATPPPATPPPATPPPATPPPATPPPATP
PPATPPPATPPPAPLASPPALVPAPSPSKPKLKAPAPSPLALSPPSPPMGAPAPSLGALT
PAPAGSDMSGVSRQKIGGGFVLGCALSWLLL

>NR|gi:255581976||Euphorbiaceae_R.communis||AGPA
MALSRAMLMTTLMLGLLASSTIAQGPTMPPSMPPTTSASPPAATAAPSPMVSAPPPAAMT
PPPAATPMSPPPTMSSPSPSMSPTMSPPPTMSPTMPPTSSATPPSMAPESSPPEMGSPPE
PAGPAPTPGGMSPPSPAMVPSTPPVSTTPDSGAFVLGSSMAALLSFLGGVAVLFA

>TC|TC280162||Fabaceae_G.max||AGPA
MGSGAVQLFLILGLLASSCLAQAPGAAPSQPPTTTPSPPPPRSAPAPAPTTPATPPPATP
PPAATPPPAATPPPAATPTPAPAPPTAAPTPASSPAASPPSPSPTVTPSPTSPNTPPGPS
PGPSGSAEPPPPSAAFSASKAFIATSALAGTFVAMALVA

>TC|TC297034||Fabaceae_G.max||AGPA
METHLVLLLALIFTVVAGVGAQGPSTSPAPPTPQSSPPPIQSSPPPVPSSPPPAQSPPPA
STPPPAPLSSPPPASPPPSSPPPASPPPASPPPASPPPASPPPASPPPASPPPATPPPAS
PPPFSPPPATPPPATPPPALTPTPLSSPPATSPAPAPAKVVAPALSPSLAPGPSLSTISP
SGDDSGAEKLWSFQKMIGSLVFGCALLSLLF

>TC|TC308383||Fabaceae_G.max||AGPA
METHLVFLLGLIFTLVAGVGAQAQGPSTSPAPPTPQASPPPVQSSPPPVPSSPPPSQSPP
PASTPPPSPLSSPPPSSPPPSSPPPSSPPPASPPPSSPPPASPPPSSPPPSSPPPFSPPP
ATPPPATPPPAVPPPSLTPTVTPLSSPPVSSPAPSPSKVFAPALSPSLAPGPSLSTISPS
GDDSGAQNLWSFQKMIGSLVFGCALLSMLF

>TC|TC288369||Fabaceae_G.max||AGPA
MDRNGVLSLAFICIVVAGVGGQSPAAAPSNTPATPAAATPAQAPSTVPKSPAPVASPKSS
PPAATPASTPAASPTTTPAAPAPVTKPPAASPPPATPPPATPPPAPVPVSSPPAPVPVSS
PPAPVPVAAPTTPVAPSPAPKHKKKGKKHGAPAPSPLLGPPAPPTGAPGPSEDASSPGPG
TAANDESGAETTMCLEKVLGGLALGWATLVLVF

>TA|BM519762||Fabaceae_G.soja||AGPA
MGSGAVQLFLILGLLASSCLAQAPGAAPSQPPTTTPSPPPPRSAPAPAPTTPLFSPPAAT
PPPAATPTPAPAPPTAAPTPASSPAASPPSPSP

>TA|TA964_47247||Fabaceae_L.japonicus||AGPA
MKMMDTHNMHVVLVLGLICIVIASVGAQSPSNSPTTSPAPPTPTTPQPPAAQSSPPPAQS
SPPPVQSSPPPASTPPPAQSSPPPVSSPPPVQSTPPPAPASTPPPASPPPFSPPPATPPP
PAATPPPALTPVPATSPAPAPAKVKSKSPAPAPAPALAPVVSLSPSEAPGPSLSSLSPAL
SPAASDDSGVEKSWSMQKMIGSLVFGSAFLYLLI

>NR|gi:535586||Fabaceae_M.sativa||AGPA
MGTHHIVLVVGLICAVFSSVGAQQAPSTSPNSSPAPPTPPANTPPTTPQASPPPVQSSPP
PVQSSPPPVQSSPPPAQSTPPPVQSSPPPVSAPPPVQQSPPPTPLTPPPVQSTPPPASPP
PASPPPFSPPPATPPPATPPPATPPPALTPTPLSSPPATTPAPAPAKLKSKAPTLAPVLS
PSDAPAPGLSSLSPSISPSGTDDSGAEKLWSHKMVGLVFGCAFLYLLF

>TC|TC121104||Fabaceae_M.truncatula||AGPA
MGTHHIVLVVIGLICVVFSSVGAQQAPSTSPNSSPAPPTPPANTPPTTPQASPPPVQSSP
PPVQSSPPPLQSSPPPAQSTPPPVQSSPPPVQQSPPPTPLTPPPVQSTPPPASPPPASPP
PFSPPPATPPPATPPPATPPPALTPTPLSSPPATTPAPAPAKLKSKAPALAPVLSPSDAP
APGLSSLSPSISPSGTDDSGAEKLWSHKMVGLVFGCAFLSLLF

>TC|TC12094||Fabaceae_P.vulgaris||AGPA
MDHHFLFFLAFTCTVLAGVGAQAPSTSPTTSPAPPTPTTPQASPPPLQSSPPPAQSSPPP
LQSSPPPAQSPPPLSSPPPASPPPASPPPASPPPASPPPASPPPASPPPASPPPASPPPF
SPPPATPPPATPPPATPPPAVPPPALTPAPLSSPPATSPAPAPAKLLSPSLTPSLSPGPS
LSTISPSGDDSGAEKLWSSGSIVLGCALVFMMF

>TA|TA362_57577||Fabaceae_T.pratense||AGPA
MGTHIVLVVGLICAVFVSAGAQQPPSTSPNASPAPPTPPTNTPPTTPQASPPPVQSAPPT
AQSSPPPVQASPPPVQSAPPPVQQSSPPPVSSTPPPVQSSPPPAPVTPPPASPPPASPPP
FSPPPATPPPATPPPAVPPPALTPTPLSSPPATSPAPSPTKVKSKAPALAPVLSPSDAPA
PTLSSLSPAITPTSNDDSGAAEMLWSQKKMVGLVFGCAFLSLMF

>TC|TC40462||Funariaceae_P.patens||AGPA
MAQGRSSAMAVLVIVAVLAFSQAVTAKDYNVGGTLNWDFPPGTDVGYYDTWSSQQKFVAG
DSLTFTFDPRAHDVQIVTESEYTNCAMSSGKKYTSGKDAIPLTKPGKYYFICSFMGHCAM
GMKMKVVVATGSSTPVTPPTSPATPPVTPPTASTPPPAVSSPPPAVSTPPPGCSYSSSSC
SHSSPGYSSCSSCGSSHSSRKCPCSSSSH

>TA|TA5304_29729||Malvaceae_G.arboreum||AGPA
MKVCNKNLFLSALLCIAIAGVLGQAPSNPPTSTPAPPTPPASTPPPTTQPPPTPTATPPP
VSTPPPTSSPPPVTASPPPVSTPPPSSPPPATPPPASPPPATPPPASPPPATPPPASPPP
ATPPPATPPPATPPATPPPAPLASPPATVPAISPVQTPLTSPPAPPTEAPAPTLGAATPG
PAGTDTSGANQMWTVQKMMGSLAMGWALLNLMV

>TA|BF271831||Malvaceae_G.arboreum||AGPA
MMLNNKFLFLSALFCIAVAGVLGQAPPTPSNPPTSTPAPPTPPASTPPPTTQPPPTPTTT
SPPPASSPPATSSPPPVTASPPPVSTPPPATPPSVSSPPPASAAPATPPAATPPPASPPP
ATPPPASAPPATPAPATAAAATPPPAPLASPPATVPAPAPSKTKAKSPALSPSASSPPSA
SNEAPTPSLGACSPSPAETDTSGVEKTWATRKMVWTLVIGWGVLSLML

>TA|EE592834||Malvaceae_G.barbadense||AGPA
MKVCNKNLFLSALLCIAIAGVLGQAPSNPPTSTPAPPTPPASTPPPTTQPPPTPTATPPP
VSTPLPTSSPPPVTASPPPVSTPPPSSPPPATPPPASPPPATPPPASPPPATPPPASPPP
ATPPPATPPPATPPATPPPAPLASPPATVPAISPVQTPLTSPPAPPTEAPAPTLGAATPG
PAGTDTSGANQMWTVQKMMGSLAMGWALLNLMV

>TA|EE593154||Malvaceae_G.barbadense||AGPA
MMLNNKFLFLSALFCIAVAGALGQAPPTPSNPPTSTPVPPTPPASTPPPTTQPPPTPTTT
SPPPASTPPPTSSPPPVTASPPPVSTPPPATPPPVSSPPPASPPPATPPPATPPPATPPP
ASPPQATPPPASPPPATPPPATPPPAPLASPPATVPAPAPSKTKAKSPALSPSASSPPSP
STEAPTPSLGASSPGPAGTDTSGVEKTWSIRKMVWSLVIGWGILTLML

>TC|TC131919||Malvaceae_G.hirsutum||AGPA
MKVCNKNLFLSALLCIAVAGVLGQAPSNPPTSTPAPPTPPASTPPPTTQAPPTPTATPPL
VSTPPPTSSPPPVTASPPPVSTPPPSSPPPATPPPASPPPATPPPASPPPATPPPASPPP
ATPPPATPPPATPPPATPPPAPLASPPATVPAISPVQTPLTSPPAPPTEAPAPTLGAATP
GPAGTDTSGANQMWTVQKMMGSLAMGWALLNLMV

>TC|TC136510||Malvaceae_G.hirsutum||AGPA
MKVCNKNLFLSALLCIAIAGVLGQAPSNPPTSTPAPPTPPASTPPPTTQPPPTPTATPPP
VSTPPPTSSPPPVTASPPPVSTPPPSSPPPATPPPASPPPATPPPASPPPATPPPATPPP
ASPPPATPPPATPPPATPPATPPPAPLASPPATVPAISPVQTPLTSPPAPPTEAPAPTLG
AATPGPAGTDTSGANQMWTVQKMMGSLAMGWALLNLMV

>TC|TC131760||Malvaceae_G.hirsutum||AGPA
MMLNNKFLFLSALFCIAVAGALGQAPPTPSNPPTSTPVPPTPPASTPPPTTQPPPTPTTT
SPPPASTPPPTSSPPPVTASPPPVSTPPPATPPPVSSPPPASPPPATPPPATPPPATPPP
ASPPQATPPPASPPPATPPPATPPPAPLASPPATVPAPAPSKTKAKSPALSPSASSPPSP
STEAPTPSLGASSPGPAGTDTSGVEKTWSIRKMVWSLVIGWGILTLML

>TC|CO083838||Malvaceae_G.raimondii||AGPA
MKVCNKNLFLSALLCIAVAGVLGQAPSNPPTSTPAPPTPPASTPPPTTQAPPTPTATPPP
VSTPPPTSSPPPVTASPPPVSTPPPSSPPPATPPPASPPPATPPPASPPPATPPPASPPP
ATPPPATPPPATPPAATPPPAPLASPPATVPAISPVQTPLTSPPAPPTEAPAPTLGAATP
GPAGTDTSGAXQMWTVQKMMGSLAMGWALLNLMV

>TC|CO078859||Malvaceae_G.raimondii||AGPA
MMLNNKFLFLSALFCIAVAGALGQAPPTPSNPPTSTPVPPTPPASTPPPTTQPPPTPTTT
SPPPASTPPPTSSPPPVTASPPPVSTPPPATPPPVSSPPPASPPPATPPPATPPPATPPP
ASPPQATPPPASPPPATPPPATPPPAPLASPPATVPAPAPSKTKAKSPALSPSASSPPSP
STEAPTPSLGASSPGPAGTDTSGVEKTWSIRKMVWSLVIGWGVLTLML

>TC|TC3775||Malvaceae_T.cacao||AGPA
MMLNNKILFLSALLCIAVAGVLGQAPSNPPTSTPAPPTPPASTPPPTTQPPPTPTSTPPP
ASSPPPTSSPPPVTASPPPVSTPPPSSPPPATPPPVSSPPPASPPPATPPPASPPPASPP
PATPPPATPPPATSPPAPLASPPAAVPAPAPSKKKAKSPAPSPLSSPPAPPTEAPAPSLG
ASSPGPAGTDVSGVEKMWSVHRMVGSLVSGWALLSLML

>TC|TC61098||Pinaceae_P.glauca||AGPA
MARSAAMTMVLFLLAGFLVSSMAQTPSASPTKSPTTPAPTTTAAPPTTTAAPPTTTATPP
VSTPPPVASPPPVATPPPVATPPPVATPPPVATPVATPPSPALVPSPAVATPPPAATPIV
TPTASTPAVTPSAAPIASPAVSPSSTSGASVASTLEKTAILAAALAGVFFL

>NR|gi:6103625||Pinaceae_P.rubens||AGPA
MARSAAMTMVLFLLAGFLVSSMAQAPSASPTKSPTTPAPTTTAAPPTTTAAPPTTTATPP
VSTPPPVASPPPVATPPPVATPPPVATPVATPPSPALVPSPAVATPPPAATPIVTPTAST
PAVTPSAAPIASPAVSPSSTSGASVASTLEKTAILAAALAGVFFL

>NR|gi:224284986||Pinaceae_P.sitchensis||AGPA
MARSAAMTMVLFLLAGFLVSSMAQTPLASPTKSPTTPAPTTTAAPPTTTAAPPTTTATPP
VSTPPPVASPPPVATPPPVATPPPVATPVATPPSPVLVPSPAVATPPPAATPIVTPTAST
PAVTPTAAPIASPAVSPSSTSGASVASTLEKTAILAAALAGVFFL

>TA|TA2837_71647||Pinaceae_P.pinaster||AGPA
MARSTAMTMVLFLLAGFLVSSMAQSPSASPTKSPTTPAPTTTAAPTTTAAPPTVTATPPT
TTATPPVSTPPPVSSPPPVTSPPPAATPPPVATPVATPPSPVLVPSPAATPPPAAVPTAT
PPPAVSPTTASPTTSPGISPSSVSGASVTSNLEKAAILTAALAAFFL

>TC|TC93314||Pinaceae_P.taeda||AGPA
MARSTAMTMVLFLLAGFLVSSMAQSPSASPTKSPTTPAPTTTAAPTTTAAPPTITATPPT
TTATPPVSTPPPVSSPPPVTSPPPAATPPPVATPVATPPSPVLVPSPAATPPPAAVPTAT
PPPAVSPTTASPATSPGVSPSSVSGASVTSNLEKAAILTAALATVFFL

>TC|TC88187||Pinaceae_P.taeda||AGPA
MDVKVEGPPYSSGRRKRSALLCAGTMLLLAIVIALVLVFSLRKNDDHTNGYGYGGGTYGP
PYIFSPPFVSTPPIFSPPETPPPPAATTPLTPPPPPATTTPVTPPSSPVVSQTIMMACNG
SLYRNVCISSLSSYPGAANANLSQLAGIAVVLSLNEAERVSDYIADLKNKSNNGSDRQAL
QDCVELYQDTVQQLNSSASKLENMNSNSFADDIADVQTWVSAALTNPSTCLDGLGGANKN
IVPVVNAKTEKSTEFMSNALAVINKLSDVSGMGNYHGDSSKTRRLLFSRDLRPQIELQ

>TC|TC182382||Poaceae_H.vulgare||AGPA
MAPQLRPWGRLLALAALLAAAAAQSSPAAAPATPTPVPVPTAPAQSPTAPATPAPTATPV
TPAPTATPVAPAKPPPAVAPVKPPPVLAPVSAPPPVVTPPPVTPPPVKAPPPVTPPPVTA
PPPATTPPPAAAPAEAPAVLPPVATPPPVAEAPAVLPPAEAPSKSKNKHKRKTSGKKKSP
DPEPEPLRPPAPIGPEPNTVEDVFRTGTLRQQSGRERAA

>NR|gi:115434076||Poaceae_O.sativa||AGPA
MALLRQWRRGLALAALLALHLALAAAQSPAAAPAQPTPTPVPTAPAKSPPAPATPAPTAT
PTPPVAPAKAPPVAPAVAPVTPPPPTPKKAPPPPVTPPPVTPPPVTPPPVSPPPATPPPA
LPPSTPPPVAAPAEAPAALPPATTPPPVAEAPAELPPAEAPTKSKNKHRKKNKRGKKASA
PAPEPLSPPAPAALSPADNQADVSGPAPSAFDLNGSNRQYGQWGFVLQTVMAALLLSLAW

>NR|gi:125553237||Poaceae_O.sativa||tp3a-containing
MAGPARWALLLLLAVALLVPAALAAGGGGNGGASASTPNNGNGGNNGNNGNNGNNGNSGN
NGNNGGGNEKHEKSPPPPYHDSPPPPRASPPPPVYSPPPPPPRSSPPPPPVYSPPPPVSS
PPPPVPSPPPPVSSPPPPVPSPPPPVSSPPPPVSSPPPPVSSPPPPVSSPPPPVSSPPPP
VHSPPPPVSSPPPPASDVVYCTNTTRYPTCTSPAYCPSRCPKSCHMDCATCKTVCDCNLP
GAVCQDPRFIGGDGNTFYFHGRRDRDFCLLSDANLHINGHFIGNHVPGLKRDPTWVQAIA
VQFSGGHRLYVGARRTAVWDDDSDRLAVVFDGETVQLQRVAHARWESGSGLSVTRTKAAN
GVLVELDGVFKITANVVPITKEDSRIHRYGVTDDDCLAHLDLAFKFYALTDDVHGVLGQT
YRSSYVNRLDVSAKMPVMGGEKQFTSSGLFAADCAVARFGRAGDAGAVAIASDELVDVKC
STGLDGVGVVCKK

>TC|TC8447||Poaceae_P.virgatum||AGPA
MARFQLAALAMAMLFAAAAAQAPAATPTPAPRASPPPATPPPTPAPVSPPAQAPAVPPPA
PAPVTPAPAPEAAAPAPAEAPTPEASSPPAPAPMGPSPSPTSDVPPAPSAAAGVSPAAKW
AAAAAVAAVAAAFY

>TC|TC4099||Poaceae_P.virgatum||AGPA
MARFQLAALALAMLFAAAAAQAPAATPTPAPRASPPPATPPPTPAPVSPPAQAPAIPPPA
PGPATPAPAPEASAPAPAEAPTPATSSPPAPAPMAPSPSPTSDVPPPPSAAAGVSPAAKW
AAAAAAAAAVAAAFY

>TC|CA181626||Poaceae_S.officinarum||AGPA
MAAPLLWQWRRLAVLGALLALHLAAAVAQSPPAAPTTPAAPTTPAAPTTPAAPTTPATPT
TPAAPTTPAAPTTPAAPTTPAAPTTPAAPTTPAAPTTPATPAPTATPTTPAPTATPVAPA
KPPPVAPAVAPAKPPPVTPXPVTPPSHDAFQPKNAARRCLPTRPAGALPAGRATRAPGPK
GAPAFGLPFPPRKTPPRPRG

>TC|CA152295||Poaceae_S.officinarum||AGPA
MAPLLRPLVLALLIASCAAQQSPPAQPPATPNAPPSNSPPQAPQAPPAGNPPPAPTATPL
PAPTTTPPPAPPTTPPPAPTTPPPAPPTTPPPAPTTPPPSPPASPPPAPTTPPPSPPASP
PPAPATPPPSPPMAPPPATPPPPATPPPPAAAPTPAPTLPPVVTPAASPKSPKAPTPAAA
MSPAPSLSPTGTPTNEGLGASARAASFATVVALAGAGLA

>TC|TC318031||Poaceae_T.aestivum||AGPA
MARIQLVALALTMLFAATAAQAPAATPMPAPVAPVSPPPTMPPPTPAPVSPPPAVAPATP
PPAPAPVTPAPAPMVPAPTPSTMAPTPEISSPPEPSMMTPTPAPSMDAPTTPAGAAGTVH
PAAAWAATAALAAAAAFY

>TC|CK199193||Poaceae_T.aestivum||AGPA
MAPQLRSWGRLLALAALLAAAAAQSSPAAAPATPTPVAVPTAPAQSPPAPTTPAPTATPV
TPAPTATPVAPAKPPPAVAPVKPPPVVAPVSAPPPVVTPPPVTPPPVKAPPPWTPPRVGG
SPAXTPPPWAAPGRGASMCPPQVGXPPLRGRGSRVWWLRAEGSGHDLEHASRKNRERRGE
VGRVLGRIGLDARPAWCRSPPRSWGLFGAGPSREGLDGQ

>NR|gi:226497260||Poaceae_Z.mays||AGPA
MARFQLAALAMAMLFAAAAAQAPAATPTPAPKASPPPPATPPPTPPPSTERREICTAPCC
NLFVIRVEPLIPSFFFFSPFFFFHPNIHIPFGIRFSRTGGICSFVLLQCRTVARTDWLGW
LGGVVLRSSICIIPC

>TC|TC526268||Poaceae_Z.mays||AGPA
MASLLRPLVLVLLIASCAAQQSPPAQPPPTPNAPPANSPPQAPPAGNPPPAPQAPPAGNP
PPAPTATPPPAPTTPPPAPTTPPPAPKTPPPAPTTPPPAPTKPPPSPPASPPPAPTTPPP
AAPTPAPSVAPTPPPVATPAASPKSPKTPSPAAATSPAPSLSPAGTPTNEDSGASARAGG
FATPPPSPPMAPPPATPPPPATPPSCFE

>NR|gi:238010832||Poaceae_Z.mays||AGPA
MASLLRPLVLVLLIASCAAQQSPPAQPPPTPNAPPANSPPQAPPAGNPPPAPQAPPAGNP
PPAPTATPPPAPTTPPPAPTTPPPAPTTPPPAPTTPPPAPTTPPPSPPASPPPAPTTPPP
SPPASPPPAPATPPPSPPMAPPPATPPPPATPPPPAAPTPAPSVAPTPPPVATPAASPKS
PKTPSPAAATSPAPSLSPAGTPTNEDSGASARAVGFATVVALAGAGLAVLL

>NR|gi:226529413||Poaceae_Z.mays||AGPA
MAAPLLRQWRSLAVLGALLGLHLIAAVAQSPPTAPTTPAAPTTPAAPTTPATPTTPAAPT
TPAAPTTPATPTTPAAPTTPIAPTTPAAPTTPAATPVAPANPPPVPTTPAPTATPVAPAK
PPPVAPAVAPAKPPPVTPPPVTPPPATPPPATPPAVLPPAVLPPAAAPPPTATPPAEAPA
SLPPATTPPPVAEAPAELPPAEAPSKGKNKHKRRRKQHGKKEAPAEAPQPLSPPAPAAPS
PADLEDVSGPAPSADVNASCRQHQHWASVVVQTAMAALLLSLAW

>TA|DY674586||Rosaceae_F.vesca||AGPA
MGRNALFFLGFLCVAIAGGVGGQAPATSPTATPAPPTPTTPPPVTSSPPPAATTPPPVAA
SPPPVTASPPPATPPPVSAPPPATPPPATPPPVSAPPPATPPPATPPPATPPPATPPPAT
PPPATPPPATPPPAPLASPPASVPAPSPTVKSPALAPSPLALSPGPPAPPVGAPGPGGIE
AESPGPSQSDQSGVEKVWTMQKMVGSLLFGWALLSLML

>TC|TC29587||Rosaceae_M.domestica||AGPA
MDRKTLFFLGFVCIVFAGVGGQSPPANPPTTSTPAPPATPTTPPSNNQPPPSPPASTPPP
ASTPPPTSAPPPVATSPPPVTTSPPPSTPPPVSAPPPATPPPVNAPPPATPPPATPPPAT
PPPATPPPATPPPATPPPATPPPAPLASPPAQVPAPAPTVKSPALAPSPLALAPGPPAPP
VGAPAPALDSGAPGPSSSVDQVLLALLFTYS

>TA|CF349763(revision)||Rosaceae_R.hybrid||AGPA
MGRNALFFLGFLCIAIAGVGGQSPATSPTSTPAPPTPTTPPPVTSSPPPAATSPPPAAAS
PPPVTASPPPATPPPVSAPPPATPPPATPPPVSAPPPATPPPATPPPATPPPATPPPATP
PPATPPPATPPPATPPPATPPPAPLASPPALVPAPSPTVKSPALAPTPAALSPGPPAPPV
GAPGPSIEAASPGPSQSSDQSGVEKMWTMQKMVGSLLFGWALLSLM

>TC|TC1667||Rubiaceae_C.canephora||AGPA
MAASTTFAAVPLLVILAAAILGNTDAATHVVGDSLGWTVPSGSSVYPNWASQQTFKVGDI
LVFNFATGAHDVAVVPKASYDGCTSSNPISLQTVGPARINLTSPGQAYFICTFGQHCSLG
QKLTVSVSGGGAAPAPAPKATPPPASSPPSTPPSASTPPPSSTPPPASTPSTTPAPPTSA
PAPAKTPAAATPAPAPKTPAGPAPTPASVPAPSPASVPSSSPAPGSGXAPAPSSAPGPGX
APTPSSASGGAGGGSSSPPPSSAPVTATATATXXVMLLSFVXXXTC

>TC|TC16918||Rutaceae_C.clementina||AGPA
MVHKSSIFVVLLCIVVAGVGGQSPTGSPTATPAPPAQTTPPPNSNSQPPTSSQPPVTSSP
PPQATPPPASSPPPATPPPVSSPPPASPPPASPPPATPPPATPPPASPPPASPPPASPPP
ATPPPASPPPASPPPATPPPAPLASPPAAVPAPAPSKKKKPTLAPSPLSSPPSPPTGAPS
PTLNAVSPGPAQTTTDASGVEKLWSMEKMVGSAVFGWAVLCLLL

>TA|TA1210_85571||Rutaceae_C.reticulata||AGPA
MVHKSSIFVVLLCIVVAGVGGQSPTGSPTATPAPPAQTTPPPNSNSQPPTSSQPPVTSSP
PPQATPPPASSPPPATPPPVSSPPPASPPPASPPPAAPPPATPPPASPPPASPPPASPPP
ATPPPASPPPASPPPATPPPAPLASPPAAVPAPAPSKKKKPTLAPSPLSSPPSPPTGAPS
PTLNAVSPGPAQNTTDASGVEKLWSMEKMVGSAVFGWAVLCLLL

>TC|TC6490||Rutaceae_C.sinensis||AGPA
MVHKSSIFVVLLCIVVAGVGGQSPTGSPTATPAPPAQTTPPPNSNSQPPTSSQPPVTSSP
PPQATPPPASSPPPATPTPVSSPPPASPPPASPPPATPPPATPPPASPPPASPPPASPPP
ATPPPASPPPASPPPATPPPAPLASPPAAVPAPAPSKKKKPTLAPSPLSSPPSPPTGAPS
PTLNAVSPGPAQTTTDASGVEKLWSMEKVVGSAVFXWAVLCLLL

>TA|TA5667_37690||Rutaceae_P.trifoliata||AGPA
MVHKSSIFVVLLCIVVAGVGGQSPAGSPTATPAPPAQTTPPPNSNSQPPTSSPPPVTSSP
PPQATPSPASSPPPATPPPVSSPPPASPPPASPPPATPPPATPPPASPPPASPPPASPPP
ATPPPASPPPASPPPATPPPAPLASPPAAVPAPAPSKKKKPTLAPSPLSSPPSPPTGAPS
PTLNAVSPGPAQTTTDASGVEKLWSMEKMVGSAVFGWAVLCLLL

>TA|AJ778438||Salicaceae_P.euphratica||AGPA
MVRENAVIFLSLICIVIAGVSGQAPATSPTATPAPPTATTPTTSPPPATSTPPPVSAPPP
VTQSPPPATPPPVSAPPPASPPPATPLFFNTSSRHTSTSNTSSRHTTTCFSTTSNPSSSC
SSTSSIGCSASSCSSSSSQQA

>TA|BU888108||Salicaceae_P.tremula||AGPA
MVRRNAVIFLSLTCIVIAGVSGQAPATSPTATPAPPTATTPTTSPPPPASPPPATPPPAT
PPPATPPPATPPPATPPPASPPPATPPPATPPPAVLAAPPALVPAPAPSKSKLKAPAPSP
LASSPPAPPTGAPAPSLGASSPGPVGTDSSGAEKIWSLQKMVVSVALGSAFWLLL

>TC|AJ778438||Salicaceae_P.trichocarpa||AGPA
MVRENAVIFLSLICIVIAGVSGQAPATSPTATPAPPTATTPTTSPPPATSTPPPVSAPPP
VTQSPPPATPPPVSAPPPASPPPATPLFFNTSSRHTSTSNTSSRHTTTCFSTTSNPSSSC
SSTSSIGCSASSCSSSSSQQA

>NR|gi:118487314||Salicaceae_P.trichocarpa||AGPA
MMLKKAVILLSLICISIAGVSGQAPATSPTAAPAPPTPTSSPPPATTPPPVSAPPPVTQS
PPPATPPPVSAPPPATPPPATPPPATPPPATPPPATPPPATPPPATPPPAVPPPAPLAAP
PALVPAPAPSKPKLKSPAPSPLALSPPSPPTGAPAPSLGASSPGPAGTDMSGVEKMGSVQ
KMVLSLVFGSAFWLLT

>NR|gi:224060684||Salicaceae_P.trichocarpa||AGPA
MVRRNAVIFLSLICIVIGGVSGQAPATSPTATPAPPTATTPTTSPPPATSTPPPVSAPPP
VTQSPPPATPPPVSAPPPASPPPATPPPATPPPATPPPATPPPATPPPATPPPATPPPAV
PPPAPLASPPALVPAPAPAPSKSKLKAPAPSPLASSPPAPPTGAPAPSLGASSPGPVGTD
LSGAEKMWSLQKMVVSLALGSTFWLLL

>jgi|Selmo1|403392|fgenesh2_pg.C_scaffold_1000229||Selaginellaceae_S.moellendorffii||AGPA
MAHTSALIFLTSFIAFAASVAVAQTPTPPPPATPPPATPPPGTRPPPGTPPPPATPPPGT
PPPPATPATPGCFPGDATVQMYNGEIKFVRELNVGDKVEYFHINLVQNIYSDVYAFGHKD
ADAMSKFFQVHSASNMLELTAGHFVPVAVNGKLVYKRAEDLKVGDSLWESSQIIEIQKVE
KVGLYNPLTLSGSIMVNNVLASTHSEWFLDSIFDAVGATEYLPYAYQAVLAPVRAIYNIV
GKEIYAKGYAAIDSFTNIAEFGRKYGGSVALTFGVAGLVLASKV

>TC|TC12238||Solanaceae_N.benthamiana||AGPA
MDRKNAIWIGLICIIIAGVGGQAPAMSPSATPAPPTPTTPSPPTTPAPTGSPPSTQPPPA
SSPPPAVSSPPPTSTSPPPAVSASPQATPPPAATPPPVTAQPPASPPPPVSAPPPATPPP
VATPPPPATPPPSPPPPAAAPAPVATPPASTPAAAPTTVATSPVPSPLGVLSPPAPPMGA
PSPSTLPAFSPGPSVSPDQSGVESLRFSKMVMGSLVFCLLI

>TC|TC44925||Solanaceae_N.tabacum||AGPA
MDRKNAIWIGFICIIIAGVGGQAPATSPSATPAPPTPTTPSPPTTPAPTGSPPRTQPPPA
SSPPPAVSSPPPTSTSPPPAVSAPPQATPPPAATPPPVSAPPPASPPPPVSSPPPATPPP
VATPPPPATPPPSPPPPAAAPAPVATPPDSTPAAAPTTVATSPAPSPLGVLSPPAPPMGA
PSPSTLPAFSPGPSVATDQSGVESLRFSKMIMGSLVLCLLI

>TC|TC42134||Solanaceae_N.tabacum||AGPA
MMGKIAVLFGFICIVIAGVGGQAPPTPTTQAPPTPTTQAPPTPTTQAPPTPTTQAPPTPT
AQAPPTPTTQAPPTPTTQAPPTPTNQAPPTPTNTPAPPTPTNTPAPPTPTGSPPPVNTQT
PPAQPPPTASPPPTVSSPPPTVTSPPPAVQSTPPPAPATPPPAVSSPPPPATPPPPAAPS
PAPLASTPAPAPAKKLTSPAPSPSALSPPLPPTGAPALAPSLGALSPAPSATDQSGVEGL
KLSTIIVKSLVLGWGLLCFLM

>TC|CV297939||Solanaceae_P.hybrida||AGPA
MDRKHAIWIGFLCIVVAGVGGQAPATSPTTTPAPPTPTTPSPPTTPAPTGSPPPTQPPPT
ATPPPVASSPPPAASSPPPAVSSPPPAVSAPPQATPPPAASPPPPVSAPPPATPPVATPP
PPATPPPTAPPAPAPVATPPXWQHPSCCSXRRQLHHRHHLQCCH

>TC|TC207340||Solanaceae_S.lycopersicum||AGPA
MDGKNALWIGFLCIIVAGVGGQAPATSPTTSPAPPTSTTPTPTGSPLPATSSPPPAVSSP
PPSVSSPPASSPLPSASPPPPVSAPPPATPPPVSAPPPATPPPVSTPPPSPPPPAAAPAP
VATPPASAPAPTPTTKVATSPAPSPVGLLSPPAPPLGA

>TC|CK245799||Solanaceae_S.tuberosum||AGPA
MDRKNALWIGFLCIIVAGVGGQAPATSPTTGPAPPTSTTPAPTGSPPPAVSSPPPSVSSP
PATSPLPAASPPPPVSAPPPATPPPVSAPPPATPPPVSTPPPPAAAPAPVATPPASAPAP
TPTTKVATSPAPSPVGLLSPPAPPLGAPSPSGLSPAPSAPDQVLINLLKSQFHFLIP

>TC|CK271244||Solanaceae_S.tuberosum||AGPA
MDRKNALWIGFLCIIVAGVGGQAPATSPTTSPAPPTSTTPAPTGSPPPAVSSPPPSVSSP
PATSPLPAASPPPPVSAPPPATPPPVSAPPVSAPPPATPPPVSAPPPVSTPPPPAAAPAP
VATPPASAPAPTPTTKVATSPAPSPVGLLSPPAPPLGAPSPSGLSPAPSAPDQSGVEGNM
RFSNMIMGSLVFGWGLLYLLI

>TC|TC176745||Solanaceae_S.tuberosum||AGPA
MMGRNNAVLLGFMICIIVAGVGGQAPPTPPTQAPPTQAPPTQTPPTNQAPPTQAPPTQAP
PTQAPPTQAPPTQAPPTNQAPPTQTPPTPTNQAPPTPTNQAPPTPTTQAPPTPATQAPPT
PTGSPPPVNNQTPPAQPPPTASPPPTVSSPPPVVTSPPPAPPTPTASPPPPVNSPPPPAA
PSPAPLPSTPAPAPAKKLTSPAPSPLGFSPPAGAPALAPSLGALSPAPSATDQSGVEGLK
MSTMVVKSLVLGWGLLCFLM

>TA|CB289351||Vitaceae_V.aestivalis||AGPA
MDCKSLLVLGLVCIVFAGVGGQAPAGGPTATPAPPTPTTPAASPPTAVTSPPASSPPPVS
APPPATPPPATPPPATPPPATPPPATPPPATPPPATPPPATPPPATPPPATPPPATPPPA
TPPPATPPPAPLASPPAAIPAPAPSTKVKSPALAPSPLTNSPPAPPAEAPGPISGGISPA
PAANDV

>TA|TA1729_246827||Vitaceae_V.shuttleworthii||AGPA
MDCKSLLVLGLVCIVFAGVGGQAPAGGPTATPAPPTPTTPAASPPAAVTSPPASSPPPVS
APPPATPPPATPPPATPPPATPPPATPPPATPPPATPPPATPPPATPPPATPPPATPPPA
TPPPAPLASPPAAVPAPAPSTKVKSPALAPSPLTNSPPAPPAEAPGPISGGISPAPAAND
VNGVEKLWSMKKVAGSLAFGLALSLVI

>TA|TA1728_246827||Vitaceae_V.shuttleworthii||AGPA
MDCKSLLVLGLVCIVFAGVGGQAPAGGPTATPAPPTPTAPAASPPAAVTSPPASSPPPVS
APPPATPPPATPPPATPPPATPPPATPPPATPPPATPPPATPPPATPPPATPPPATPPPA
TPPPATPPPAPLASPPAAVPAPAPSTKVKSPALAPSPLTNSPPAPPAEAPGPISGGISPA
PAANDVNGVEKLWSMKKVAGSLAFGLALSLVI

>NR|gi:225429333||Vitaceae_V.vinifera||AGPA
MDCKSLLVLGLVCIVFAGVGGQAPAGGPTATPAPPTPTTPAASPPTAVTSPPASSPPPVS
APPPATPPPATPPPATPPPATPPPATPPPATPPPATPPPATPPPATPPPATPPPATPPPA
TPPPATPPPATPPPATPPPAHLASPPAAVPAPAPSTKVKSPALAPSPLTNSPPAPPAEAP
GPISGGISPAPAANDVNGVEKLWSMKKVAGSLAFGLALSLVI

>TA|TA1719_94328||Zingiberaceae_Z.officinale||AGPA
MASPKPALLAAISLALLATCIAQAPAASPTATPPTATPPPASSPPPVAAPPPATPPPVAT
PPPATPPPATPPPAAAPPPSPLPPTGSPAPATPPPAAPTPSGSPASSPSPSSPPAPSGPA
PSPSTPGSTPSDGNSGIVQEASMGLMALFGAVALLL

>TA|TA1434_94328||Zingiberaceae_Z.officinale||AGPA
MASPKPVLLAAIALALLATCIAQSPAASPTASPPTPTATPPPASSPPPAATPPPATPPPV
ATPPPVATPPPVATPPPVATPPPATPPPMATPPPATPPTAAPPPSPLPPSNSPAPATAPP
TPSPAASPGTSPPSPSSSPSPTAPASVPSPSTSPSDNGNSGVVHDVSIGLVALFGAVALL
L

**@ AGPB Class [13 Proteins (9 w/ GPI anchor), 11 Species (7 w/ GPI anchor)]**

>TC|TC48548||Asteraceae_H.annuus||AGPB
MAASHHILLLLSFAYLAAFSTAQAPSMSPMMMPMAPTMMPMTPPPTTMPMTPPPTMMPMT
PPPMMMPMGPPPMMMPMTPPPTMMPMSPGPSMMPDSPPAPMAPSMAPGPATMSPGPSMMP
ETPPNGAIMQYSSITMLAFCGGLLLLI

>TA|CF087353||Asteraceae_H.argophyllus||AGPB
MAASRHILLLLSFQYLAAFSTAQAPSTSPMMMPMAPTMMPMTPPPTMMPMTPPPTMMPMG
PPPMMMPMTPPPTTMPMGPPPMMMPMSPRPSMMPDSPPAPMPTSMAPGPPTMSPGPSMTP
ETPPNRAIIAVF

>TA|EL433738||Asteraceae_H.ciliaris||AGPB
MAASXHILLLLSFAYLAAFSTAQAPSMSPMMMPMAPTMPMTPPPTTIPMTPPPTMMPMTP
PPTMMPMTPPPMMMPMGPPPMMMPMTPPPTTMPMGPPPMMMPMSPGPSMMPDSPPSPAPM
APGPATMSPGPSMMPPPNGAIMQYSSITMLAFCGGLLLLI

>LOC|AT4G16980||Brassicaceae_A.thaliana||AGPB
MASSFSSQAFFLLTLSMVLIPFSLAQAPMMAPSGSMSMPPMSSGGGSSVPPPVMSPMPMM
TPPPMPMTPPPMPMTPPPMPMAPPPMPMASPPMMPMTPSTSPSPLTVPDMPSPPMPSGME
SSPSPGPMPPAMAASPDSGAFNVRNNVVTLSCVVGVVAAHFLLV

>TC|TC77477||Brassicaceae_B.napus||AGPB
MASSSQAFLLLTLSMVLVHFSLAQSPMMAPSGSMSMPPMPSGGSPMPMMTPPPMPMMTPP
PMPMMTPPPMAMAPPPMPMTPPPMPMAPMPMTPSSSPMSPPTTMAPSPETVPDMASPPMM
PGMDSSPSPGPMPPAMASPDSGAFNVRNDVVAISFLVAAHLLLV

>TC|TC86521||Brassicaceae_B.napus||AGPB
MMASSQALLLLTLTMVLIPFSLAQSPMMAPSGSMSMPPMPSGGSPMPMMTPPPMPMMTPP
PMPMTPPPMPMAPPPMPMTPPPMPMAPPPMSMATPPMMPMTPSSSPMSPPTTPMSPGPSP
ASVPDMASPPMPAMESSPSPGPGPMPPAMSSPDSGAFNVRIDVVALSFLVAAHLLLV

>TA|AM058006||Brassicaceae_B.oleracea||AGPB
MMASSQALLLLTLTMVLTPFSLAQSPMMAPSGSMSMPPMPSGGSPMPMMTPPPMPMMTPP
PMPMTPPPMPMTPPPMPMTPPPMSMATPPMMPMTPSSSPMSPPTTPMSPGPSPAISVPDM
ASPPTPAMESSPSPGPGPMPPAMSSPDSGAFNVRIDVVALSFLVAAHLLLV

>TA|BQ791389||Brassicaceae_B.rapa||AGPB
MASSQAFLLLTLSMVLVHFSLAQSPMMAPSGSMSMPPMPSGGSPMPMMTPPPMPMMTPPP
MPMMAPPPMAMAPPPMPMTPPPMPMAPMPMAPSSSPMSPPTTMAPSPETVPDMASPPMMP
GMDSFLLRDHATGNGL

>NR|gi:15866589||Brassicaceae_C.rubella||AGPB
MASSSSPQAFLLLALSMVLVPFSLAQAPMMAPSGSMSMPPMPSGGGGSMIPPPAMTPPPM
PMMTPPPMPMMTPPPMPMMTPPPMPMMTPPPMPMMTPPPMSMTPPPMPMMTPPPMSMTPP
PMPMASPPMMSMGPSPSPLTVPGMASPPMPSGMESAASPGPMPPPMAASPDSGAFNVRNN
VVAVSCVVGVVAAHLLLV

>TA|TA439_98038||Brassicaceae_T.halophila||AGPB
MASSQALLLVTLPMVLIHFSLAQSPMMAPSGSMSMPPMSSGRSSPMPMMTPPPMPMAPPP
MPMTPPPMPMAPPPMPMTPPPMMPMTPSSSPMAQPTTPMGPSPSPLSVPDMASPPMPPSA
MESAPSPGPMPPGMASQDSGAFNVRNNVVALSFVVGVAAAHLLIV

>TC|TC169461||Malvaceae_G.hirsutum||AGPB
MAGSSAMLVQLTLMIALLSCSMAQSPSASPTMSPSSSTTPSPVATPPPTTMTPPPAMTPM
SAPAPSTPMAPTPSTTPMAPTPSSAPEAPTPPAPAPPSMTPNSAPTTPPSAMTPGEGPPG
SAASTREYTMSLLALLGGVALFV

>TC|TC29273||Rosaceae_M.domestica||AGPB
MALTRAMLFLTMSLAVVAPTSSTPSTPITMAPSPMTMMAPPPMAMMTPPPMMTPPPMMTP
PPMMTPPPMMTPPPMMTSPPMMTPPPMMMPPPMMTPPPMMTPPPMMTPPPMMTPPPMMTP
PPPATPGPSIGAPPTPTQNSGFVHGSSMALVAFLGGLALLF

>TC|TC42632||Rosaceae_M.domestica||AGPB
MALTRAMLFLTMSLAVVAPTSSNPSTPITMAPSPMTMMAPPPMAMMTPPPMMTPPPMMTP
SPMMTPPPPMMMPPPMMTPPPMMTPPPPATPGPSIGAPPTPPPMMTPPPMMTPPPMMTPP
PMMTPPPMLTPGPSIGAPTQNSGFVHGSSMALVAFLGGLALLF

**@ AGPC Class [4 Proteins (3 w/ GPI anchor), 4 Species (3 w/ GPI anchor)]**

>TA|TA404_15368||Poaceae_B.distachyon||AGPC
MASSRGGVARLLCLAMAAAAASAAQFRVGEQRGWSVPDGGAEPYNSWAGRMRFVIGDQLL
FVYPKGSDSVLVVDAGAYGSCNTTAYTAKFEDGNTVVTLDRSGPFYFISGNEAGCKANQK
LEVVVLAAAHTPPPAPVSPSPSSMPPSPASPPSSMTPPMASPPSPGPSSMTPPMASPPSP
GPSSAPPMPAPAAGPGASAPAPSPTGAAAPADSPPAPGSPS

>TC|TC162836||Poaceae_H.vulgare||AGPC
MASARGVAGLLCFALVAAAASAAQYRVGEQRGWSVPGAGAEPLNSWAARMRFVIGDQLLF
VYPKDTDSVLLVDQAAYNACNTTTYVSKFQGGSTVFTLDRSGPFFFISGNEASCKAEQKL
IVVVLSVEHTPPGLLPPPPPPQPSMPPSSAPPMPSPMSPPSMTPPMPSPMSPPSMTPPSA
TPMPSPMSPPSMTPPSAAPMPSPMSPPSMTPPAGAPMPSPMSPPSMTPPSGAPMPSPMSP
PSMTPPSGAPMPSPMSPPSMTPPSGEPMPPTVAPVATPDSPPSPTGLAPSPGTPGAGGGS
SPGTPGSDTNSPPAPGAGGASATTPGSAAAPVTAGLIGTLAAGIGYAMLAI

>NR|gi:62861389||Poaceae_L.elongatum||AGPC
MARTCGALGLLCFALVAAAASAAQFRVGGQRGWSVPPAGAEPFNAWAERLRFIFGDQLLF
VYPKDTDSVLLVDQAAYNACNTTAYVSKFQGGSTVFTLDRSGPFFFISGNEASCKAEQKL
IVVVLSLDHTPPGLLPPPPPSMPSPPSPPSMTPPSAAPMPSPMSPPSMTPPSGAPMPSPM
SPPSMTPPSGAPMPSPMSPPSMTPPSAAPMPSPMSPPSMTPPSGAPTPSPMSPPSGVPAA
TPVSAPSPAGSVPSIAPGATPDSPPPPSTPGGGSSPGTPGSGTNSPPAPGADGADSTTPG
SAAAPAMAGLIGTLAAGIGYAMLAI

>NR|gi:62861391||Poaceae_T.aestivum||AGPC
MASPRGLAGLLCFALAAAAASAAQYRVGEQRGWSVPAAGAEPLNTWSARMRFIIGDQLLF
VYPKDTDSVLLVDQAAYNACNTTTYVSKFQGGSTVFTLDRSGPFFFISGNEASCKAEQKL
IVVVLSVDHTPPGLLPPPPPPSMPPSSAPPMPSPMSPPSMTPPSAAPMPSPMSPPSMTPP
SAAPMPSPMSPPSMTPPSAAPMPSPMSPPSMTPPSAAPMPSPMSPPSMAPPSSEPMPSPM
TPAAAPGTTPVSPASPAGPAPSPGTPGGGSSPGTPGSDTSSPPAPAADGANSTTPGSAAA
PVTAGLIGTLAAGIGYAMLAI

**@ PRPA Class [32 Proteins, 20 Species]**

>NR|gi:470322||Apiaceae_D.carota||PRPA
MNLLLILGVAIFAAPSLADSHSHPPIHKPPVYTPPVHKPPIHKPPVYTSPVHKPPIHKPP
VYTPPVHKPPIHKPPVYTPPVHKPPSEYKPPVEATNSVTEDHYPIHKPPVYKPPVQKPAP
EHKPPVHKPPIHKPPVHNTPSVTDDHYPAHPIHKPQPIHRPPVHKPPTEHKPPVHEPATE
HKPSPVYQPPKTEKPVPEHKPPHLPPIVVRPPPTHKPNPPYGHHPGHPPVENTGN

>TA|TA964_3818||Fabaceae_A.hypogaea||PRPA
MACLSWLMVILFVALILTPQGLADYYKPPYKHDEPPKYNHPIEKPPVYKPPVYKPPVYKP
PVYKPPVHKPPVYKPPVYKPPVHKPPVYNKPPYHHDKPPHGKYPPQHD

>TA|TA14_217475||Fabaceae_A.stenosperma||PRPA
MAPLSWLMVILFVALILTPQGLADYYKPPYKHDEPPKYKHPIEKPPVYKPPVYKPPVYKP
PVYKPPVHKPPVYKPPVYNKPPVYNKPPYHHDKPPHGKYPPQHD

>TC|TC300975||Fabaceae_G.max||p2vyk-containing
MANSYLSLLVLILVALLLTPQGLADESDSYYKPPVYKKPPVYKPKPKPPIHKPKPPVYKK
PPYGKYPPVQDNDHF

>NR|gi:131004||Fabaceae_G.max||PRPA
MASFVSFLVLLLAALILMPQGLATYYKPIKKPPVYKPPVYKPPVYKPPVYKPKPPVYKPK
PPVYKPPYKKPPYKKPPYGKYPPVEDNTHA

>NR|gi:255641352||Fabaceae_G.max||PRPA
MRNMASLSSSLVLLLAALILSPQVLADYEKPPIYKPPVYTPPVYKPPVEKPPVYKPPVYK
PPVEKPPVYKPPVYKPPIYKPPVYKPPVEKPPVYKPPVYKPPVYKPPVYKPPIEKPPVYK
PPVYKPPVYKPPVYKPPVYKPPVKKPPIYKPPYPKYPPGSN

>NR|gi:131001||Fabaceae_G.max||PRPA
MASLSSLVLLLAALILSPQVLANYENPPVYKPPTEKPPVYKPPVEKPPVYKPPVENPPIY
KPPVEKPPVYKPPVEKPPVYKPPVEKPPVYKPPVEKPPVYKPPVEKPPVYKPPVEKPPVY
KPPVEKPPVYKPPVEKPPVYKPPVEKPPVYKPPVEKPPVYKPPVEKPPVYKPPVEKPPVY
KPPVEKPPVYKPPVEKPPIYKPPVEKPPVYKPPYGKPPYPKYPPTDDTHF

>TA|CA785593||Fabaceae_G.soja||PRPA
MASLSSLVLLLAALILSPQVLANYENPPVYKPPTEKPPVYKPPVEKPPVYKPPVENPPIY
KPP

>TA|TA3703_3848||Fabaceae_G.soja||p2vyk-containing
MANSYLSLLVLILVALLLTPQGLADESDSYYKPPVYKKPPVYKPKPKPPIHKPKPPVFTS
PSHPFTRNPLMVSTHQFKTTITSKPCNKAC

>TC|TC41089||Fabaceae_L.japonicus||PRPA
MSDMATLRSLVLLLVALALTPQGFANYEKPPEYTPPIYKPPVEKPPVYKPPVEKPPVYKP
PVEKPPVYKPPVEKPPVYKPPVEKPPVYKPPVEKPPVYKPPVEKPPVYKPPVEKPPVYKP
PVEKPPVYKPPVEKPPVYKPPVEKPPVYKPPVEKPPVYKPPVEKPPVYKPPVEKPPVYKP
PVEKPPVYKPPVEKPPVYKPPVEKPPVYKPPAEKPPVYKPPVYKPPVYKPPVEKPPVYKP
PVEKPPVYKPPVEKPPVYKPPVEKPPVYKPPVEKPPGYKPPVEKPPVYKPPVEKPPSLQA
PS

>TA|TA424_3870||Fabaceae_L.albus||PRPA
MAYLPSFLLLLVALTLSPQGLANYEKPPEYKPPIGKPPVEKPPVYKPPVEKPPVYKPPVE
KPPVYKPPVEKPPVYKPPVVKPPVYKPPVVKPPVYKPPVEKPPVYKPPVEKPPIYKPPYE
KPPIYKPPYEKPPYGGHHPPASDGTR

>NR|gi:3914100||Fabaceae_M.sativa||PRPA
MASFSLSILVFFISALVLVPQGFAEYYLNPAYRPPQTKPPVNKPSHKEPPVHKPPHKEPP
VNKPRHKEPPVHKPPHKDPPVNKPPQKESPVHKPPRKEPPTHKHPPAEDNIHF

>NR|gi:3914441||Fabaceae_M.sativa||PRPA
MASSNFLVLLLFALFVIPQGLANYDKPPVYQPPVYKPPVEKPPVYKPPVEKPPVYKPPVY
KPPVEKPPVYKPPVVKPPVYKPPVYKPPVYKPPVEKPPVYKPPVYKPPVYKPPVVKPPVY
KPPVYKPPVEKPPVYKPPVYKPPVEKPPVYKPPVEKPPVYKPPVYKPPVYKPPVVKPPVY
KPPVYKPPVYKPPVEKPPVYKPPVYKPPVEKPPVYKPPVYKPPVEKPPVYGPPHHP

>LOC|Medtr4g031660||Fabaceae_M.truncatula||p2vyk-containing||Extensin-like protein chr04
MASFGFLVLLVSLMVTAHWYVSACIDCEKSPPRFGARCCDPREKTPVENLPVYKPSIEKP
PVYKPSAEKPLVDNPPAYKIPFEPPFYKPSIEKPQV

>NR|gi:3914459||Fabaceae_M.truncatula||PRPA
MASSNFLVLLLFALFAIPQGLANYEKPPVYQPPVYKPPVEKPPVYKPPVEKPPVYKPPVE
KPPVYKPPVYKPPVYKPPVVKPPVYKPPVYKPPVYKPPVYKPPVEKPPVYKPPVYKPPVV
KPPVYKPPVYKPPVEKPPVYKPPVVKPPVYKPPVYKPPVVKPPVYKPPVYKPPVYKPPVE
KPPVYKPPVYKPPVEKPPVYGPPHHP

>NR|gi:3914458||Fabaceae_M.truncatula||PRPA
MASSNFLVLLLFALFAIPRGLANYDKPPVYQPPVYKPPVEKPPVYKPPVEKPPVYKPPVE
KPPVYKPPVEKPPVYKPPVEKPPVYKPPVEKPPVYKPPVEKPPVYKPPVEKPPVYKPPVE
KPPVYKPPVEKPPVYKPPVEKPPVYKPPVEKPPVYKPPVEKPPVYKPPVEKPPVYKPPVE
KPPVYKPPVEKPPVYKPPVEKPPVYKPPVEKPPVYKPPVEKPPVYKPPVEKPPVYKPPVE
KPPVYKPPVEKPPVYKPPVEKPPIYKPPVEKPPVYKPPVEKPPVYKPPVEKPPVYKPPVE
KPPVYKPPVEKPPVYKPPVYKPPVYKPPVEKPPVYKPPVYKPPVEKPPVYKPPVYKPPVE
KPPVYGPPHHP

>TC|TC136784(revision)||Fabaceae_M.truncatula||PRPA
MASSNFLMLXLFALFVIPKGLANYEKPPEYHPPVENPPFYKPPVEKPPVYKPPIEQPPIY
KPPVENPPIYKPPVEKPPVYKPPFEKPPIYHPPVEIPPVYNPPVEHPPIYKPPVYKPPVE
QPPVYKPPVEKPPIYKPPVEKPPVYQPPVYKPPVEKPPVYKPPVEKPPVYKPPVEKPPIY
KPPVEKPPVYKPPVEKPPVYKPPVEKPPVYKPPVEKPPIYKPPVEKPPVYKPPVEKPPAY
KPPVEKPPVEKPPVYKPPVEKPPVYKPPVEKPPVYKPPVEKPPVYKPPVEKPPVYKPPVY
KPPVEKPPVYKPPVEKPPVYKPPVEKPPIYKPPVEKPPVYKPPVEKPPVYKPPVENPPVY
KPPFEKPPIYKPPIEYPPVYKPPFEKPPIYKPPVEKPPFYKPPFENPPFYIPPVEEPPVY
KPPFEKPPIYTPPF

>NR|gi:437310||Fabaceae_M.truncatula||PRPA
MASISFLVLLLFALYIIPQGLANYEKPPEYKPPVENPQFYKPHIEKPPVHKPLVEKPPTH
HPPIEKPPIYKPPVEKPPAYKPPVEHHPVYKPSVEKPPVHKPPVEKPPVHKPPVEKPPVH
KPPVEKPPVHKPPVEKPPVHKPPVEKLPVYKPSVEKPPVYKPPVEKPPLHKPLVEKPPVH
KPPVEKPPVHKPPVEKLPVYKPPVEKPPVYKPHVEKPPVNKPPVEKPPVHKPPVEKPPVH
KPPVEKLPVYKPHVEKPPVYKPLVEKPPLHKPPVEKTPMHKPPVEKPPVHKPPVEKPPVE
KLPVYKPPVEKPPVYKPHVEKPPLHKPPVEKPPVHKPPVEKPPVHKPPVEKLPVYKPPVE
KPPVYKPHVEKPPLHKPPVEKPPVHKPPVEKPPVHKPPVEKPPVHKPPVEKLPVYKPPVE
KPPVYKPPVEKPPVHKPPVEKPPLHKPQVEKPTEYKPPIEKFPVYKPPVEKPQVHKPPVE
KPPVHKSPVKKLPVYKPPAEKPPVYKPPVENPPVHKPLVEKPPVYKPPVEKPPVHKPPFE
KPPIYTPPL

>TC|TC15211||Fabaceae_P.coccineus||PRPA
MKNMASLSSLVLLLAALLLSPQGLANYDKPPVENPPVYNPPVEKPPVYKPPVEKPPVYKP
PVEKPPVYKPPVEKPPVYKPPVENPPVVQAPS

>TC|TC14665||Fabaceae_P.coccineus||PRPA
MASLSFLVLLFAALVLSPQGLANYDKPPVYKPPIQKPPVYKPPVKKPPVYKPPVEKPPVY
KPPVEKPPVYKPPVEKPPVYKPPFQKPPVYKPPYGKPLLHESQYEKAPLYKSPPLYKPPV
HEPPVYKPPV

>NR|gi:82581111||Fabaceae_P.vulgaris||PRPA
GIPASLSSLVLLLAALLLSSQGLANYDKPPVEKPPVYKPPVEKPPVYKPPVEKPPVYKPP
VEKPPVYKPPVEKPPVYKPPVEKPPVYKPPVEKPPVYKPPVEKPPVYKPPVEKPPVYKPP
VEKPPVYKPPVEKPPVYKPPVEKPPVYKPPVEKPPVYKPPVEKPPVYKPPVEKPPVYKPP
VEKPPVYKPPVEKPPVYKPPVEKPPVYKPPVEKPPVYKPPVEKPPVYKPPVEKPPVYKPP
VEKPPVYQPPYGKPPHPKYPPSTN

>NR|gi:6580688||Fabaceae_P.sativum||PRPA
MASLTFLLLLLLALIIPQGFANYEKPPVYRPPVETPPIYKPPVEKPPVYKPPVYKPPVEK
PPVYKPPVEKPPVYKPPVKKPPVYKPPVEKPPVYKPPVEKPPTYKPPVEKPPVYKPPVEK
PPTYKPPVERPPVYKPPVEKPPTYKPPVEKPPVYKPPVEKPPTYKPPVEKPPAYKPPVEK
PPVYKPPVGVSHQFTRLHSRSHQTNQVPYDGTCV

>TA|TA1824_57577||Fabaceae_T.pratense||PRPA
MASSSFLVLFLFALFAIPQGLANYENPPVYTPPIVKPPVYKPPVEKPPVYKPPVEKPPVY
KPPVEKPPVYKPPVEKPPVYKPPVYKPPVEKPPVYKPPVEKPPVYKPPVYKPPVYKPPVY
KPPVYKPPVEKPPVYKPPVYKPPVEKPPVYKPPVYKPPVYKPPVYKPPVEKPPVYKPPVE
KPPVYKPPVEKPPVYKPPVEKPPVYKPPVEKPPVYKPPVEKPPVYKPPVYKPPVYKP

>NR|gi:8096255||Fabaceae_T.repens||PRPA
MASSSFLVLLLFALFAIPQGLANYEKPPVYTPPVYTPPIVKPPVYKPPVEKPPVYKPPVE
KPPVYKPPVEKPPVYKPPVEKPPVYKPPVEKPPVYKPPVEKPPVYKPPVVKPPVYKPPVV
KPPVYKPPVYKPPVVMPPVYKPPVYKPPVYKPPVVKPPVYKPPVYKPPVVKPPVYKPPVV
KPPVYKPPVYKPPVVKPPVYKPPVYKPPVVKPPVYKPPVYKPPVVKPPIYKPPVVKPPVY
KPPVEKPPVYKPPVYKPPVVKPPVYKPPVYKPPVVKPPVYKPPVEKPPVYKPPVEKPPVY
KPPVEKPPVYKPPVEKPPVYKPPVEKPPVYEPPHHPKYPPAGL

>NR|gi:1167892||Fabaceae_V.sativa||PRPA
LKMASFLLSTLVFFLAALILVPQGLAQYHLNPVYEAPVNGPPVNKPPQKETPVQKPPQKE
PPVHKSPRNEPPRHKPPHKKSHLHVTKRSYGKHATEEHSIHF

>TC|TC9426||Orobanchaceae_T.pusilla||PRPA
MRVHSHCQAFFLLSLLLLSAVNFCSANDTSFEVVGTGECADCTENHFKTIHAYSGLGVSI
DCKVKNGDMKRIGLTELDEQGNFKVSLPKDLVDNDLKLKHECYAQLHSAAAIPCPAHNGL
EASKIIYKSQTKNGKHVFVPTKPLQFSAALCSSKFFWPYFKWPPLPPLKHFGHPWLFPPL
PPLYIPKPEPPPVPVYEPPTPVYTPEPTPPTPVYTPEPTPPTPVYTPEPPTPVYKPPVEK
PPVYKPPVEKPPVYKPPVDKPPVYKPPVEKPPVYKPPVYKPPVEKPPVYKPPVEKPPVYK
PPVEKPPVYKPPVYKPPVEKPPVYKPPVEKPPVYKPPVEKPPVYKPPVEKPSVYKPPVEK
PPVYKPPVEKPPVYKPPVEKPPVYKPPVEKPLPPPVPIYYPPVDKPLPPIPQYPIPPITK
KPCPPLPELPPFPIPPKYFHHPKFGNYPPLPPIFPHA

>TA|TA2193_75702||Salicaceae_P.euphratica||PRPA
MQMTSLLMLFVGVVVLVTPSFADPDKPPTYGPAPPYLKPSKVEKPFPEHKPPVYKPPKIE
KPPVYKPPKIEKPPVYKPPPVYKPPKIEKPPVYKPPVIVKPPPFHKPLPPYGHYPGHPPV
ENAEYIKPKN

>TA|CK114945||Salicaceae_P.tremula||PRPA
VLFVGVVVLVSPSFADGDKPPEYETAPPYLKPPKVEKPFPEHKPPVYKPPKIEKPPVYKP
PKIEKPPVYKPPKIEKPPVYKPPKIEKPPVYKPPKIFKPPVYKPPKIEKPPVYKPPKIEK
PPVYKPPKIEKPPVYKPPKIEKPPVYKPPKIEKPPVYKPPVIVKPPPFHKPLPPHGHYPG

>TA|CA925538||Salicaceae_P.tremuloides||PRPA
MQITSLLVLFVGVVVLVTPSFADGDKPPEYEHAPPYLKPPKVEKPFPEHKPPPKIEKPPV
YQPPKIEKPPVYKPPKIEKPPVYKPPKIEKPPVYQPPKIEKPPVYKPPPQWQR

>TC|TC106063||Salicaceae_P.trichocarpa||PRPA
MQMTSLLMLFVGVVVLVTPSFADPDKPPTYGPAPPYLKPSKVEKPFPEHKPPVYKPPKIE
KPPVYKPPKIEKPPVYKPPPVYKPPKIEKPPVYKPPVIVKPPPFHKPLPPYGHYPGHPPV
ENAEYIKPKN

>NR|gi:224107867||Salicaceae_P.trichocarpa||PRPA
MQITSLLVLFVGVVVLATPSFADYYKPPKYEPKPPYFKPPKVEKPFPEHKPPVYKPPKIE
KPPVYKPPPVYKPPIEKPPVYKPPPVYKPPIEKPPVYKPPPVYKPPIEKPPVYKPPIEKP
PVYKPPKIEKPPVYKPPKIEKPPVYKPPKIEKPPPFHKPLPPYGHYPGHPPVENAEYIKP
KN

>TC|TC98448||Salicaceae_P.trichocarpa||PRPA
MQITSLLVLFVGVVVLVTPSFADGDKPPEYETAPPYLKPPKVEKPFPEHKPPVYKPPKIE
KPPVYKPPKIEKPPVYKPPKIEKPPVYKPPKIEKPPVYKPPKIEKPPVYKPPKIEKPPVY
KPPKIEKPPVYKPPKIEKPPVYKPPKIEKPPVYKPPKIEKPPVYKPPVIVKPPPFHKPLP
PYGHYPGHPPVENAEYIKPKN

**@ PRPB Class [2 Proteins, 2 Species]**

>TC|TC78072||Pinaceae_P.glauca||PRPB
MEFAIPGGMKFCKFLVILLLSISCFASPTSATYGHAAPKPYPYTSPPPPYKPPVIPPPVI
KPPPVYPPPQDHKPPPHGISPVHPPPKWPSPPVYTPPVITPPIHKPPIKPPPIYKPPIKP
PPVYKPPIKPPPVYKPPVIPPPIHKPPIKPPPVYKPPVIIPPVHKPPIKPPPVYKPPVKP
PPVYKPPPVYKPPIKPPPVHKPPPHEISPVYPPPITTPKPPPIHKPPTVPVIPPPVSKPP
PIYKPPVPVIPPPVIKPPPVYKPPVPVIPPPVIKPPPIYKPPVPVIPPPVIKPPPVYKPP
VPVIPPPVIKPPPIYKPPVPVIPPPVIKPPPVYKPPVPVIPPPVVMAVALAAAVVTEPVP
VLVRATVLALVPVTVLEMVAAMEELLVGVIKFMINNITRKYVYDLHGMRSILLNKINRPW
HLFAYPSCLRDLIVHQIHI

>TC|TC93219||Pinaceae_P.taeda||PRPB
MEFGIPGGMKFCKSLVIFLLSISCLFASQTSASYGHPAPKPYAYISPPPPVIPPPVIKPP
PVITPPVTKPPPVYPPPHVVPKPPPHEISPVHPPPKSPSPPVYTPPVIHPPIHKPPIHKP
PIKPPPVYTPPIKPPPVYTPPVIHPPEHKPPIKPPPVYIPPIKPPPVYIPPIKPPPVYVP
PIKPPPVYVPPIKPPPVYVPPIKPPPVYKPPIKTPPGHKPPPHGISPVHPPPLVKPKPPP
VYKPPVPVIPPPVTKPPPVYKPPVPVIPPPVTKPPVYKPPVPVIPPPVVKPPPVYKPPVP
VIPPPVVKPPPVYKPPVPVIPPPVVKPPPVVKPPPVYKPPVPVIPPPVVKPPPVYKPPVP
VIPPPVVKPPPVYKPPVPVIPPPVVKPPPVYS

**@ HLTA Class [67 Proteins, 58 Species]**

>TA|DT589832(revision)||Agavaceae_Y.filamentosa||HLTA
MDFTRLSPYLLILTLSFLSSPSQTLGNCGTCSPAIGPLPLPPVTVPPVIEKPPITLPPVI
EKPPIILPPIIEKPPITIPVINPPATKTPPCSPPKPKPKPKPTPKPCPPTPAAPTPAAPP
SCPIDTIKIGACLDVLGAVVHVGDPAAECCPLITGITSVQAAVCLCTIIKANALGINVFF
PIALELLVTCGKTPPPGYKCV

>TA|DT597086||Agavaceae_Y.filamentosa||HLTA
MDSTKLSALLLILTVFISTTPPTLSCGYCPPSTKNPNPPKSKPPKSHPPKTTNPPKGGPI
VLPPIIGKPPVTIPPVIGKPPVTLPPFIGKPPVVVGPPSAGNAPCPPGVTPTPGTPTTPT
TPPAGPATCPIDTLKLGACVDLLGGLVHIGLGDPVVNQCCPLLQGLIEIEAAVCLCTTIR
LRLLNINIFLPLALQLLITCGKTPPPGYTCTI

>TA|CN451764||Amaryllidaceae_L.longituba||HLTA
MDSTQLSSILLILAIFISAIPPTFSSANCSPTKKPTPPNHKHPKPHPPKSKPPKGPIVLP
PIIGKPPITVPPIIGKPPITVPPVIGKPPITLPPIIGKPPITLPPVIGKPPVTVPPVIGK
PPVTVPPVIGNPPCPPTPA

>TA|TA141_4058||Apocynaceae_C.roseus||HLTA
MDSSSKITALVLISMLFVSSAAPILDCGSCTPSAPTHKKKPKVPKSPGLLPPIIKPPVDL
PPVTLPPIVKPPVTLPPIVKPPITLPPIVKPPVDLPPVTLPPVKPPLELPPVTLPPAIKP
PITLPPILKPPVTLPPVTVPPVTVPPITNPPAKGKPCPTPAPGKKATCPIDTLKLGACVD
LLGGLVHIGLGDPAVNKCCPIISGLAEIEAAVCLCTTLKLKALNLKLYVPVALQLLITCG
KTPPPGYTCSL

>TA|TA241_51953(revision)||Arecaceae_E.guineensis||HLTA
MDSSKLSALLLLMLFISSTPIALACRNCPTPPTPKTPETPKGPITIPPINIKPPITLPPI
IGKPPITLPPFIGKPPITLPPLIPPIIGMAPPTVGTPPPANKPGCV

>TA|EL380689||Asteraceae_C.tinctorius||HLTA
MESSKKLSALFLISLFLVSSATPVDLDCGPCDKPKPKPKPPTGKPPVVKPPSKHPPHVKP
PGKVPPVVKPPIKVPPVVKPPIKVPPVKPPITIPPVKPPVELPPVTLPPVKPPVDLPPVI
PPVKPPVDLPPVTLPPVKPPVDLPPVIPPVKPPVDLPPVTLPPVKPPVDLPPVIPPVKPP
VDLPPVIPPVKPPVDLPPVIPPVKPPVDLPPVTLPPVKPPVTLPPVKPPVDLPPVIPPIV
KPPIDLPPVIPP

>TC|TC39832||Asteraceae_H.annuus||HLTA
MESSKQQLTALLLISMFFISTATPIALDCVPCGDKPPPHHHHHPKPPTGKPPKSPKPPTG
KPPSTHPPHVKPPHVKPPVVKPPITHPPVVKPPVVKPPVVKPPVVKPPITLPPVVKPPIT
LPPVVKPPITLPPVVTPPVVTPPVTVPPVLNPPSGGKPGTPCPPPPFVPTPAPTCPIDSL
KLGACVDLLGGLVHIGLGDPVVNQCCPVLAGLVELEAAVCLCTTIKLKLLNINLYLPLAL
QLLITCGKTPPPGYTCSI

>TA|TA1063_73275(revision)||Asteraceae_H.argophyllus||HLTA
MESSKQQLTALLLISMFFISTATPIALDCVPCGDKPPPHHHHHPKPPTGKPPKSPKPPTG
KPPSTRPPHVKPPHVKPPVVKPPITHPPVVKPPVVKPPITLPPVVKPPITQPPVVKPPIT
LPPVVTPPVVTPPITVPPVLNPPSGGKPGTPCPPPPFGPTPAATCPIDSLKLGACVDLLG
GLVHIGLGDPVVNQCCPVLAGLVELEAAVCLCTTIKLKLLNINLYLPLALQLLITCGKTP
PPGYTCSI

>TA|TA3827_73275||Asteraceae_H.argophyllus||HLTA
MESSKKLMAFFLISMFLVSSANPVDLDCHCGKPTPTPKPPVVKPPVVKPPVVKPPVVKPP
VVKPPVVKPPVVKPPVVKPPVVKPPVVKPPVTLPPVVKPPITLPPVVKPPVTLPPVTPPP
VVKPPITLPPVPLPPVVKPPVTLPPVTLPPVVTPPIVTPPTAKPCPPPMSTPAPPATCPI
DTLKLGACVDLLGGLVHIGLGDPAVNKCCPIIAGLAELEAAVCLCTTLKLKALNLSIYVP
IALELLVTCGKTPPPGYTCSL

>TA|TA399_73280||Asteraceae_H.ciliaris||HLTA
QKLTALFFISLFLVSSASPVLDCGHPKTTTKKPPVVKTPVSNPPNIKLPVPLPPLPIPPV
KPPVDLPPLPIPPVKPPVDRPPLPNLPPVKPPVDLPPLPDLPPVKPPVELPPLPVPPIKP
PVDLPPLPLPPLPLPPVITPPVVTPPTEKPCPPPKVTPKSSATCPLDTLKLGACVDLLKG
LVHINLGDPAVNQCCPVIAGLAELEAAVCLCTTLKVKLLNLKIYVPLALELLVTCGKTPP
PGFTCPH

>TA|DY942919(revision)||Asteraceae_H.petiolaris||HLTA
MESSKQQLTALLLISMFLISTATPIALDCVPCGHHPSKPPTGKPPSTHPPIVKPPITLPP
VVKPPITLPPVVKPPITLPPVVNPPVTLPPVVKPPITLPPVVKPPITLPPVVNPPVTLPP
VVKPPITLPPAVKPPITLPPVVNPPVTLPPVVKPPITLPPVLNPPVILPPILNPPVILPP
VLNPPSGGTPGTPCPPGPFVPTPAATCPIDALKLGACVDLLGGLVHIGLGDPVVNQCCPV
LAGLVELEAAVCLCTTLKVKLLNLNIYLPLALQLLVTCGKTPPPGYTCAL

>TA|EL512395||Asteraceae_H.tuberosus||HLTA
MESSKQQLTALLLISMFLISTATPIALDCVPCGHPPSKPPTGKPPSTHPPIVKPPSTHPP
IVKPPITLPPVVKPPITLPPVVKPPITLPPVVNPPVTLPPVVKPPITLPPVVNPPVTLPP
VVKPPITLPPVLNPPIGGTPGTPCPPGPFVPTPAATCPIDALKLGACVDLLGGLVHIGLG
DPVVNQCCPVLAGLAELEAAVCLCITLKVKLLNLNIYXPLALQLLVTCGKTPPPGYTCAV

>TA|TA882_43195||Asteraceae_L.perennis||HLTA
MESSKKLTALFFVSLFLVSSANPVLDCGSCDKPKPPAEKPPVVKPPVKPPVTLPPIVKPP
ITLPPVVKPPITLPPVVKPPVTLPPIKPPVTLPPIKPPVTLPPIKPPVTLPPIKPPVTIP
PIKPPVTLPPIKPPVTLPPIKPPVTLPPIKPPVTLPPIKPPVTLPPVKLPPIKPPVTLPP
IKPPVTLPPVKLPPIKPPVTIPPIKPPVTLPPVPLPPIVKPPVTLPPITLPPILKTPVTL
PPVTPPVVTPPGVTPPKGEPCPPPKGAPSPVASETCPIDILKLGACVDLLGGLVNIGLGD
PAVNKCCPIIAGLAELGAAVCLCTTLKVNLLNLNIYLPVALQLLVTCGKTPPPGYTCSV

>TA|DW061873||Asteraceae_L.saligna||HLTA
LQLILFLTVEVVTSQNHPTECRSLVKPPVKPPVTLPPVVKPPITLPPVVKPPVTLPPIKP
PVTLPPVKPPVTLPPIKPPVTLPPIKPPVTLPPIKPPVTLPPIKPPVTLPPIKPPVTLPP
IKPPVTLPPIKPPVTLPPIKPPVTLPPIKPPVTLPPVKPPVTLPPIKPPVTLPPIKPPVT
LPPVTLPPVKPPVTLPPIKPPVTLPPIKPPVTLPPLPLPPLRPPVTLPPIKPPVTLPPLP
LPAIQPVVPPIVTTPVVTPPV

>TA|TA4298_75948||Asteraceae_L.saligna||HLTA
MESSKQLTALLLISMFFISSAAPIALDCVPCGDKPPHHHNKPIKPPVTLPPVKPPVTLPP
IKPPVTLPPIKPPVTLPPVTLPPVKPPVTLPPIKPPVTLPPIKPPVTLPPLPLPPLKPPV
TLPPIKPPVTLPPLPLPPLPPVVPPIVTPPVVTPPVVTPPVVTPPEGEPCPPPKGASPPG
ASETCPIDILKLGACVDLLGGLVNIGLGDPAVNKCCPIIAGLAELEAAVCLCTTLKVKLL
KPNIYLPIALQLLVTCGKTPPRGTLALSKI

>TC|BQ995406(revision)||Asteraceae_L.serriola||HLTA
MESSKQLTALLLISMFFISSAAPIALDCVPCGDKPPHHHNKPPKSPKPPTGKPPTTHPPV
VKPPVVTPPITLPPIVKPPITLPPVVNPPSTKPGTPCPPPPFVPAPAATCPIDALKLGAC
VDLLGGLIHIGLGDPVVNKCCPILAGLVELEAAVCLCTTLKLKLLNINIYLPLALQLLVT
CGKTPPPGYTCSI

>TC|TC1776||Asteraceae_L.serriola||HLTA
MESSKKLTALFFISLFMVSSANPVLDCGSCDKPKPPTEKPPVVKPPVKPPVKPPVTLPPV
VKPPVTLPPVVKPPVTLPPIKPPVTLPPVKPPVTLPPVKPPVTLPPIKPPVTLPPIKPPV
TLPPIKPPVTLPPVKPPVTLPPIKPPVTLPPIKPPVTLPPVTLPPIKPPVTLPPVKPPVT
LPPIKPPVTLPPVKPPVTLPPIKPPVTLPPLPLPPLKPPVTLPPLPLPLLKPPVTLPPIK
PPVTLPPLPLPPLPPIVPPIVTPTVGTP

>TA|TA788_75947||Asteraceae_L.virosa||HLTA
MESSKQLTALLLISMFFISSATPIALDCVPCGDKPPHHHNKPPKSPKPPTGKPPTTHPPV
VKPPKIHPPVVKPPVVRPPIVKPPITLPPIVKPPVVTPPVTLPPIVKPPIVTPPITLPPV
VTPPITLPPIVKPPITLPPVVNPPSTKPGTPCPPPPFVPAPAATCPIDALKLGACVDLLG
GLIHIGLGDPVVNQCCPILAGLVELEAAVCLCTTLKLKLLNINIYLPLALQLLVTCGKTP
PPGYTCSI

>TA|TA19_121540||Asteraceae_S.aethnensis||HLTA
MESSKQLTALLLISMFFISTATPIALDCVPCGGKGKPGHHGPSKPPTHKPPTTGKPPSIH
PPHVRPPIVKPPVVKPPVVTPPITLPPVVKPPITIPPVVKPPITLPPVVTPPITLPPILN
PPVLNPPSGGKPGTPCPPPPFVPAPAATCPIDALKLGACVDLLGGLVHIGLGDPVVNQCC
PVLAGLVELEAAVCLCTTLRIKLLNINIYLPLALQLLVTCGKTPPPGYTCSI

>TA|DY659904||Asteraceae_S.cambrensis||HLTA
MESSKQLTALLLISMFFISTATPIALDCVPCGGKGKPGHHGPSKPPTHKPPTSKPPSIHP
PHVRPPIVKPPVVKPPVVTPPITLPPVVKPPITLPPVVKPPITLPPVVTPPITLPPVLNP
PVLNPPSGGKPGTPCPPPPFVPAPAATCPIDALKLGACVDLLGGLVHIGLGDPVVNQCCP
VLAGLVELEAAVCLCTTLKIKLLNINIYLPLALQLLVTCG

>TA|TA127_121541(revision)||Asteraceae_S.chrysanthemifolius||HLTA
MESSKQLTALLLISMFFISTATPIALDCVPCGGKGKPGHHGPSKPPTHKPPTTGKPPSIH
PPHVRPPIVKPPVVKPPVVTPPITLPPVVKPPITIPPVVKPPITLPPVVTPPITLPPVLN
PPVLNPPSGGKPGTPCPPPPFVPAPAATCPIDALKLGACVDLLGGLVHIGLGDPVVNQCC
PVLAGLVELEAAVCLCTTLKIKLLNINIYLPLALQLLVTCGKTPPPGYTCSI

>TA|TA26_121554||Asteraceae_S.squalidus||HLTA
MESSKQLTALLLISMFFISTATPIALDCVPCGGKGKPGHHGPSKPPTHKPPTTGKPPSIH
PPHVRPPIVKPPVVKPPVVTPPITLPPVVKPPITIPPVVKPPITLPPVVTPPITLPPILN
PPVLNPPSGGKPGTPCPPPPFVPAPAATCPIDALKLGACVDLLGGLVHIGLGDPVVNQCC
PVLAGLVELEAAVCLCTTLRIKLLNINIYLPLALQLLVTCGKTPPPGYTCSI

>TA|TA20_76276||Asteraceae_S.vulgaris||HLTA
MESSKQLTALLLISMFFISTATPIALDCVPCGGKGKPGHHGPSKPPTHKPPTSKPPSIHP
PHVRPPIVKPPVVKPPVVTPPITLPPVVKPPITIPPVVKPPITLPPVVTPPITLPPVLNP
PVLNPPSGGKPGTPCPPPPFVPAPAATCPIDALKLGACVDLLGGLVHIGLGDPVVNQCCP
VLAGLVELEAAVCLCTTLKIKLLNINIYLPLALQLLVTCGKTPPPGYTCSI

>TA|TA103_50225||Asteraceae_T.officinale||HLTA
MESSKQLTALLLISMFFISSAAPIALDCAPCGDKPPHHHHKPPKSPKPPTGKPPTAHPPV
VRPPKTHPPVVKPPVVKPPVVKPPVVKPPVVRPPVVRPPITLPPVVKPPVVTPPITLPPV
VTPPIVKPPITLPPVVTPPIVKPPITLPPVVNPPTTKPGTPCPPPPFVPVPAPAATCPID
ALKLGACVDLLGGLVHIGLGDPVVNQCCPILAGLVELEAAVCLCTTLKIKLLNINIYLPL
ALQLLITCGKTPPPGYTCSI

>TA|CO037491||Casuarinaceae_C.glauca||HLTA
MELSKVYAVFLIFMLFISSATPILGCGYCGDPPSRHKSHKGRHSKGKSPKGPITHPPIVK
PPVTLPPVTVPPIKPPVTLPPIVKPPVTLPPIVKPPVTLPPIVKPPVTLPPIVKPPVTLP
PIVKPPVTLPPIVKPPVTLPPISTPPVI

>TC|TC6629||Convolvulaceae_I.nil||HLTA
MEVSKVTALLLISMLFISSATPILACGSCGKPSHKPKKPTPKPPITIKPPVTLPPVVKPP
VTVPPITLPPVVKPPVPIPPITLPPVVKPPVPIPPITLPPVVKPPVTVPPITLPPVVKPP
VPIPPITLPPVVKPPVTVPPITLPPVVTPVTPGPAKPCPPAAATCPIDTLKLGACVDLLG
GLVHVGLGDPAVNECCPILKGLVELEAAACLCTTLKIKALNLSIYVPLALQLLVTCGKTP
PPGYTCAL

>NR|gi:255582327(revision)||Euphorbiaceae_R.communis||HLTA
MESTKILALLFICMFFTSSAATAVNPDCGACGHHKPPKHGGHKGGGGGLLPPIVKPPINL
PPVIPPVVKPPINLPPVIPPVVKPPINLPPVIPPVVKPPINLPPVIPPVVKPPINLPPVI
PPVIKPPINLPPVTVPPVITNPPKGSGGKPCPPPPAKAATCPIDTLKLGACVDLLGGLVH
IGLGDPVVNQCCPVLQGLVELEAAVCLCTTLKLKLLNLNIYVPLALQLLVTCGKTPPPGY
TCTL

>TA|EG984006||Fabaceae_C.tetragonoloba||HLTA
MESSSKHHRIYAFLFICMIFISSVTPILGCGYCGKPPPKKHKPSRKPKHPIVKPPVTVPP
IVKPPVTVPVPPVTVPPIVKPPVTIPPVTVPPITVPPVTVPPIVKPPGILPPVTLPPVLN
PPSKGGSTTPYPCPPPPAKATCPIDTLKLGACVDLLGGLVHIGVGDPVVNQCCPVLQGLV
ELEAAVCLCTTLKLKLLNLXIYVPLALQLLVACGKSPPPGYTCSX

>TC|TC300655(revision)||Fabaceae_G.max||HLTA
MESSKIYAYLFLCMLFLSSATPILGCGYCGKPPKKHKPGKTPKTPTVPVVKPPVTIPPIV
KPPVTIPPIVKPPVTIPPIVNPPVTIPPIVNPPVTIPPIVNPPVTVPPVVLPPIVKPPGF
LPPILNPPSTPTPSPGKGGNTPCPPPKSPAQATCPIDTLKLGACVDLLGGLVHIGLGDPV
ANQCCPVLQGLVELEAAVCLCTTLKLKLLNLNIYVPLALQLLVACGKSPPPGYTCSL

>TC|TC4330(revision)||Fabaceae_P.coccineus||HLTA
MESSKIHAYLFLSMLFISSATPILGCGYCGKPPKKQNHGKKPKTPVVKPPVTLPPISPIV
KPPVTVPPITVPPVTVPPIVKPPISLPIPPVTVPPVLNPPTTPTTPGKGGNTPCPPPNSP
AQATCPIDTLKLGACVDLLGGLVHVGVGDPAANQCCPVLQGLVELEAAACLCTTLKLKLL
NLNIYVPIALQLLVACGKSPPPGYTCSL

>TA|TA60_3802(revision)||Grossulariaceae_R.americanum||HLTA
MDSSKISALLFIFMIFISSATPILGCGSCKKPPVHHKHPKKPKTPKGPITIPPIVKPPIH
LPPVIPPIVKPPIHLPPVIPPVVKPPITLPPVIPPIVKPPITLPPVVTPPITLPPVVKPP
ITLPPIVKPPITLPPIVPPVLTPPTTKPCPPPPSKPTPTPAPSPTSPTCPIDTLKLGACV
DLLGGLVHIGLGDPVANECCPVLSGLVEIEAAVCLCTTLKLKLLNLNIYVPIALQLLLTC
GKTPPPGYTCSI

>TA|TA1304_3415(revision)||Magnoliaceae_L.tulipifera||HLTA
MDSSKISALLLIFMLFVSSASPILACTYCKNPPKHKSPKGKPPKGPINLPPVIVKPPITR
PPVVGKPPITLPPVIGKPPVTLPPVIGKPPITLPPVIGKPPVTLPPVIGKPPITLPPVIG
KPPITLPPVIGKPPITLPPVIGKPPITLPPVIGKPAMTLPPVIGKPPITLPPVIGKPPWT
LPPVIGKPPITLPPVIGKPPITLPPVIGKPPITLPPVIGKPPITLPPVTLPPVLPPVIGT
PPTTKPPGCGCSPPPKEETCPIDTLKLGACVDLLGGLVHIGLGDPVVNTCCPLLQGLVEI
EAAVCLCTTIKLKLLNLKIFVPIALQLLATCGKTPPPGFTCSL

>TC|TC136058||Malvaceae_G.hirsutum||HLTA
MDSTKISALLFICMLFISSATPALGCRSCRKAHPKAKPIKKPHPVKPPIHVKPPVTVPPV
TVPPVTVPPVTVPPIVKPPVTVPPVTVPPVKPPVELPPIAKPPITVPPVTVPPVKPPVDL
PPIAKPPITVPPVTVPPVTVPPVKPPVDLPPIAKPPITVPPVTVPPVKPPVDLPPVTVPP
VKPPVDLPPVTVPPVKPPVELPPIAKPPITVPPVTVPPVKPPVDLPPIAKPPITVPPVTL
PVPPVTVPPVTVPPVKPPVDLPPIAKPPITVPPVTVPPVKPPVDLPPIAKPPITVPPVTV
PVPPVTVPPVTVPPVKPPVDLPPIAKPPITVPPVTVPVPPVTVPPVKPPINLPPVTVPPV
TNPPSGKPCPPPPSTKATCPIDTLKLGACVDLLGGAVHVGVGDPVVNACCPVLKGLVELE
AAVCLCTTLKLKLLNLKIYAPIALQLLIQLWENSPLPVTLCSL

>TC|TC1980||Malvaceae_G.raimondii||HLTA
MDSTKISALLFICMLFISSATPALGCRSCRKAHPKAKPIKKPHPVKPPIHVKPPVTVPPV
TVPPVTVPPVTVPPIVKPPVTVPPVTVPPVKPPVELPPIAKPPITVPPVTVPPVKPPVDL
PPIAKPPITVPPVTVPPVTVPPVKPPVDLPPIAKPPITVPPVTVPPVKPPVDLPPVTVPP
VKPPVDLPPVTVPPVKPPVELPPIAKPPITVPPVTVPPVKPPVDLPPIAKPPITVPPVTL
PVPPVTVPPVTVPPVKPPVDLPPIAKPPITVPPVTVPPVKPPVDLPPIAKPPITVPPVTV
PVPPVTVPPVTVPPVKPPVDLPPIAKPPITVPPVTVPVPPVTVPPVKPPINLPPVTVPPV
TSPPSGKPCPPPPSTKATCPIDTLKLGACVDLLGGAVHVGVGDPVVNACCPVLKGLVELE
AAVCLCTTLKLKLLNLKIYAPIALQLLISCGKTPPPGYTCSL

>TC|TC2538||Malvaceae_T.cacao||HLTA
MDNTKISALLLICMLFISAATPILGCGTCGKPPPKHRKPKGKSPKGPITIPPIVKPPINL
PPVTVPPIVKPPVTVPPVTLPPVVKPPINLPPLIPPIVKPPINLPPVTVPPITTPPSGKS
CPPPPATATCPIDTLKLGACVDLLGGLVHIVIGDPVVNECCPVLSGLVELEAAVCLCTTL
KLKLLNLNIYVPLALQLLITCGKTPPPGYTCSL

>TA|TA1214_77108||Nymphaeaceae_N.advena||HLTA
MASSPSKLYTLFLILMITTPPILACGSCSKHHPKKSHKNPPKGGVTVPPVIVKPPITVPP
VIVKPPITIPPVIVKPPIIYPPVITPPVTTPPVYTPPVITPPVTSPPVYTPPVVTPPSVP
CPPPPQPPATCPIDTLKLGACVDLLGGLVHIGVGDPVTNHCCPILGGLVELEAAVCLCTT
IKLKLLNINIILPLALKLLVTCGKTPPPGFTCPN

>TA|TA1220_77108||Nymphaeaceae_N.advena||HLTA
MSSPPPPPKLYALFLILIIIISTTPSILACGYCSPPKRGYKKPPKGGVTVPPVIVKPPIT
LPPVIVKPPITLPPVIVKPPITLPPVIVKPPITLPPVIVKPPITLPPITLPPIVKPPITL
PPITLPPIVKPPITLPPIIKPPITLPPVVTPPILNPPVYTPPVVTPPILNPPVYTPPVVT
PPSVPCPSPPQKPATCPIDTLKLGGCVDLLGGLVHIGLGDPTANECCPILAGLAELEAAV
CLCTTIKLKLLNINIILPIALNLLATCGKSPPPGFTCPT

>TA|CB032483||Orchidaceae_P.equestris||HLTA
MDSSSNVSVLLLILILSFSSSFQVAFGCGECPTPTTPKPAPKEGPITLPPIIGNPPVTLP
PIIGKPPVTLPPITGKPPITLPPVIGKPPVTLPPIIVKPPVTLPPINVKPPVTLPPVIAN
PPITKPPATGTSGCSPPPAAKKTCPLDILKFGACLDLLGNVIQIGDPTVSECCPLIKGLT
GIEAAACLCTTIKLKLLNIYVLYPIAVGSTDKLWAKXARLPGLHLLFGSKFQKEGTILES
PILVR

>TA|CO742613(revision)||Orchidaceae_P.violacea||HLTA
MDSSSKVSVFLLILILSFSSSFHVAFGCGECPTPATPKPAPKEGPITGNPPVTLPPITVK
PPVTLPPITVKPPVTLPPITVKPPVTLPPVIVKPPVTVPPVIVNPPTTXPPATGTPGCSP
PPAAKKTCPLDVLKFGACLDLLGNVIQIGDPAVSECCPLIKGLTGIEAAXCLCTTIKLKL
LNIYVLFPIAVDLLISCGKTPPXGYTCY

>TA|TA1614_3467||Papaveraceae_E.californica||HLTA
MESIKVSALLLLCMIFISSVTPILGCGYCNDPKHKPKTPKKKHPKTPSGKPPVVKPPVKP
PVTTPPIVKPPVTIPPIVKPPVTIPPIVKPPVTIPPIVKPPVTIPPIVKPPITIPPIGKP
PITLPPGLNPPVTLPPIGEPPITLPTVLKPPV

>TA|CK743910||Papaveraceae_E.californica||HLTA
MDSPKVSAFLLICMLFISSVTPILACGYCSDPTHKPKIPKKKAPIVKPPVVVYPPVVKPP
VTIPPIVKPPVTIPPIVKPPVTIPPIQKPPVTIPPVIGAPVTRPPGTGNPPNTNVPCPPT
RPSSPASTCPIDTLKLGACVDLLGGLIHIGLGDPTVNECCPVLQGLVELEAAXCLCTTLK
LKLLNLNIFVP

>TC|EX307191||Pinaceae_P.glauca||HLTA
MDSTKISALLFICMIFISSATSILGCHSCGKPKNKHPKTPKGSITLPPIVKPPVTLPPTV
KPPVTLPPQPVPPIVKPPVTLPPLPVPPIAKPPVTLPPLPVPPIAKPPVTLPPLPVPPIA
KPPVTLPPLPVPPIVKPPVTLPPLPVPPIVKPPVTLPPLPVPPIVKPPVTLPPLPLPPVL
PVPPVTLPPLPLPPVLPVPPVITNPPKGKPCPPPPSSKDTCPIDTLKLGACVDLLGGLVH
VGLGDPVVNQCCPVLKGLVELEAAVCL

>TA|EE683819||Plumbaginaceae_L.bicolor||HLTA
MDSSRMSAILIICMLFISSVTPILGCGSCGHHGGKKPHGKPIKPVTKPGKGGPVTLPPII
SKPPVTLPHLPPVPPVVTPPVTLPPVKPPIDLPPVVKPPIEVPPVKPPIDLPPAVKPPIE
LPPVKPPIDLPPVVKPAIDLPPVKPTIDLPPVGIPPVLPKATTPS

>TC|TC25688||Ranunculaceae_A.formosa||HLTA
MDSSKLSALLLICMLFISSVTPILGCDYCKKHQPKKKPSKGPGVKPPVKLPPVKLPPIVK
PPVTLPPVTLPPIKPPVTLPPVTLPPIKPPVTLPPVTLPPIKPPVTLPPVVKPPVELPPV
TLPPIVKPPVELPPVTLPPIVKPPVELPPVTLPPVVGGGGSPKPCPSPPKKDTCPIDTLK
LGACVDLLGGLVHIGLGDPAVNTCCPVLQGLVEVEAAACLCTTLKIKLLNLKVYVPLALQ
LLISCGKTPPPGYTCTI

>TA|TA2161_57918||Rosaceae_F.vesca||HLTA
MESSKLNALFFIFIIVISSAAPILGCPTCGKKKPPPKHTKPKPPKGPIKVPPIKVPPVTI
PPIVKPPITIPPVIPPVVKPPITIPPVIPPVVKPPITIPPIVKPPIEIPPVIPPVVKPPI
TLPPVVIPPVIPPIVKPPVEIPPITTKPPSKGTPCPPPPGKPAPKDTCPIDTLKLGACVD
LLGGLVHIGLGDPVVNECCPVLSGLVELEAAVCLCTALKIKLLNLNIFVPIALQLLVTCG
KSPPPGFTCSI

>TA|TA564_3747||Rosaceae_F.x||HLTA
MESSKLNALFFIFIIVISSAAPILGCPTCGKKKPPPKHTKPKTPKGPIVPPIKVPPVTIP
PIVKPPVTIPPVIPPIVKPPITIPPIVKPPIEIPPVIPPVVKPPITLPPVVIPPVIPPVV
KPPVEIPPITTKPPPSKGTPCPPPPGKPAPKDTCPIDTLKLGACVDLLGGLVHIGLGDPV
VNECCPVLSGLVELEAAVCLCTALKIKLLNLNIFVPIALQLLVTCGKSPPPGFTCSI

>TC|TC29062||Rosaceae_M.domestica||HLTA
MAESSKLHALFLICLLFISSASPILGCGYCGKPKHKPIKPTKPPKGPIVIPPIHVKPPGV
KVPPVTVPPIVKPPVTIPPIVKPPVTNPPSPGTPCPPPPGKPSPASKDTCPIDTLKLGAC
VDLLGGLVHIGLGDPAVNECCPVLQGLAELEAAVCLCTTLKIKLLNLNIFVPLALQLLVT
CGKNPPPGYTCSL

>TC|TC29553||Rosaceae_M.domestica||HLTA
MAESSKLHALFLICLLFISSASPILGCGTCGKPKHKPVKPKPKTPKGPIVVPPIHVKPPG
VKIPPVTVPPIVKPPVTIPPIVKPPVTIPPIKPPVTIPPIKPPVTIPPIVKPPVTIPPIV
KPPVTIPPVTIPPIKPPVTLPPVIPPIVKPPVTIPPVTIPPIKPPVTLPPIIPPGIIPPV
IGGPPATKPPSPGTPCPPPPGNTCPIDTLKLGACVDLLGGLVHVGQGDPVVNECCPVLQG
LVELEAAVCLCTTLKIKLLNLNIFVPLALQLLVTCGKSPPPGFTCSL

>TC|AJ873054||Rosaceae_P.persica||HLTA
MAESSKLHALFFICLLFISSATPILGCGTCGKPPPKHKPKPKTPKGPIPPIHVKPPVVKP
PVTIPPIVKAAVTIPPIVKTPVTIPPIVKPPVTIPPIVKPPVTIPPIV

>TC|TC369||Rubiaceae_C.canephora||HLTA
MDSSKITALLLLSMLLIASANPVDPDCGSCSPKPKTPKGPITLPPVVKPPVTLPPVTLPP
VVKPPITLPPIVKPPVTLPPVTLPPVLNPPVTLPPVTLPPVLNPPVTLPPVTLPPVAKPP
VTLPPVTLPPVVNPPITLPPITLPPVTMPPVTTKPPKAKPCPPPPVKPKKPKQAKCPVDT
LKMGACADLLGGVMHIGFGDPVVNECCPILSGLVDVEAAVCLCTTLKLKALNLEMFVPIA
LELLVYCGKTPPPGYTCSL

>TA|CB086431(revision)||Rubiaceae_H.centranthoides||HLTA
MDSSKITALLLLSMLLLATAKPTPDCGKKPHKPGKPHKPKSPKPPVTLPPVVKPPITLPP
VVKPPITLPPVTVPPVTVPPIKPPVTLPPVTLPPVVKPPITLPPVTVPPVTVPPITVPPV
TGPPKTPCPSPPVTKPPETCPIDTLKLGACVDLLGGLVHIGLGDPVVNECCPVLSGLVEL
EAAVCLCTTLKIKLLNLQIFVPIALQLLVTCGKTPPPGLTCSL

>TA|TA490_219667||Rubiaceae_H.terminalis||HLTA
MDSSKITALLLLSTLLLATANPTLDCGECEKPPKKPHKPKRPVTSPPVTVPPITVPPVVK
PPITLPPVTLPPLPPVTLPPVVKPPINLPPVTQPPVTVPPVTGSPKTPCPSPPVTKPPET
CPIDTLKLGGCVDLLGGLVHSGLSDPAVNECCPILSGLVELEAAACLCTTLKLKLMNLNI
YVPIAMQLLVTCGKTPPPGYTCSL

>TA|DY306342||Rutaceae_C.reshni||HLTA
MDSSKLSALLIICMLFISTATPILGCGTCGKPHPKHRKPRQPRPIGLPPIVKPPVPLPPV
TLPPVTVPPIKPPVDLPTLPPVTLPPVLPNPPSARKPCPCSYTPPPPAAATCPIDTLKLG
ACVDLLGGLVHIGLGDPVVNQCCPVLQGLVELEAAVCLCTTLKLKLLNLNIYLPLALQLL
VTCGKTPPAGFTCTL

>TA|TA6262_37690||Rutaceae_P.trifoliata||HLTA
MDSSKLSALLIICMLFISTATSHSWLWHRVASRNPKHRKPRQPRPIGLPPIVKPPVPLPP
VTLPPVTVPPIKPPVHLPTFPPVTLPPVLPNPPSTWKPCPCSYTPPPPPPAAATCPIDTL
KLGACVDLLGGLVHIGLGDPVVNQCCPVLQGLVELEAAVCLCTTLKLKLLNLNIYLPLAL
QLLLTCGKTPPAGFTCTL

>TA|TA3268_3696||Salicaceae_P.deltoides||HLTA
MDSTKISALLFICMIFISSATSILGCHSCGKPKNKHPKTPKGSITLPPIVKPPVTLPPLP
VPPIVKPPVTLPPLPVPPIVKPPVTLPPLPVPPIVKPPVTLPPLPVPPIVKPPVTLPPLP
LPPVLPVPPVTLPPLPLPPVLPVPPVITNPPKGKPCPPPPSSKDTCPIDTLKLGACVDLL
GGLVHVGLGDPVVNQCCPVLKGLVELEAAVCLCTTLKIKALNLNIYVPLALQLLVTCGKT
PPPGYTCSL

>TA|TA2999_75702(revision)||Salicaceae_P.euphratica||HLTA
MDSTKISAFLSLCMFLISSAAPTLDCGSCGKHPKNKHPKTPKGPITLPPLPVPPIVKPPV
TLPPLPVPPIVKPPVTLPPVTLPPVTVPPATTKPPNGKPCPPPPSPKDTCPIDTLKLGAC
ADLLGGLVHIGLGDPVVNQCCPVLKGLVELEAAVCLCTTLKIKALNLNIYVPLALQLLVT
CGKTPPPGYTCSL

>NR|gi:224087088||Salicaceae_P.trichocarpa||HLTA
MDSTKISAFLFLCMIFISSAAPTLDCGSCGKHPKNKHPKTPKAPITLPPLPVPPIVKPPV
TLPPLPVPPIVKPPVTLPPVTLPPVTVPPITVPPVTTKPPKGKPCPPPPSPKDTCPIDTL
KLGACVDLLGGLVHIGLGDPVVNQCCPVLTGLVELEAAVCLCTTLKIKALNLNIYVPLAL
QLLVTCGKTPPPGYTCSL

>TC|TC121126||Salicaceae_P.trichocarpa||HLTA
MDSTKISALLFICMIFISSATSILGCHSCGKPKNKHPKTPKGSITLPPIVKPPVTLPPIV
KPPVTLPPLPVPPIVKPPVTLPPLPVPPIVKPPVTLPPLPVPPIVKPPVTLPPLPVPPIV
KPPVTLPPLPVPPIVKPPVTLPPLPVPPIVKPPVTLPPLPIPPVLPVPPVTLPPLPIPPV
LPVPPVTLPPLPIPPVLPVPPVTLPPLPIPPVLPVPPVTVPPVITNPPKGKPCPPPPSSK
DTCPIDTLKLGNCVDLLGGLVHVGLGDPVVNQCCPVLKGLVELEAAVCLCTTLKIKALNL
NIYVPLALQLLVTCGKTPPPGYTCSL

>TC|TC16692||Solanaceae_C.annuum||HLTA
MEFSKITSLLFICMLLLSSLTPILGCGYCGKPSHKPKTKKPKTPSPIVKPPINLPPIGIP
PVTVPPVVKPPINLPPIGLPPVTVPPVVKPPITLPPVVKPPINLPPIGIPPVTVPPVVKP
PITLPPVVKPPIKLPPIGLPPVTVPPVITPSPKGKKPCPPTTKATCPIDTLKLGACVDLL
GGLVHIGLGDPAVHECCPIISGLAELEAAACLCTTLKLKLLNLKIYVPLALQLLVTCGKT
PPPGYTCSI

>TC|TC49975||Solanaceae_N.tabacum||HLTA
MESSKITCLLFISMLFLSSFTPILGCGYCGKPSHKPKKPKTPSPSIKPPINLPPIGIPPV
TVPPIVKPPINLPPVVPPVIKPPIGIPPVTVPPVVKPPINLPPVVPPVIKPPIGIPPVTV
PPVIKPPINLPPIIPPVIKPPIGIPPVTVPPVVKPPIGIPPIVKPPINLPPIGIPPVTVP
PITVPPVTPSPKGKPCPPPTTTKATCPIDTLKLGACVDLLGGLVHIGIGDPAVNECCPIL
QGLVELEAAACLCTTLKVKLLNLKIYVPLALQLLVTCGKTPPPGYTCSL

>TC|TC205859||Solanaceae_S.lycopersicum||HLTA
MEFSKITSLLFISMLFLSSFTPILGCGYCGKPSHKPKKPKVPTPIVKPPVDLPPIGIPPI
VKPPVNLPPIGIPPIVKPPVKLPPIGIPPIVKPPVILPPVGIPPIVKPPVKLPPVGIPPI
VKPPVDLPPVGIPPVTVPPIVKPPVDLPPIGIPPVTVPPVIKPSPKGKKPCPPTTKATCP
IDTLKLGACVDLLGGLVHIGLGDPAVNECCPILSGLVELEAAACLCTTLKVKLLNLKIYV
PLALQLLVTCGKSPPPGYTCSI

>TC|TC183428||Solanaceae_S.tuberosum||HLTA
MEFSKITSLLFISMLFLSSLTPILGCGYCGKPSHKPKKPKVPSPIVKPPIHLPPIGIPPV
TVPPIVKPPVDLPPIGIPPVTVPPIVKPPVDLPPVGIPPVTVPPVKPPVDLPPIGLPPIV
KPPIGLPPIGIPPVIVPPVVKPSPKGKKPCPPTTKATCPIDTLKLGACVDLLGGLVHIGL
GDPAVNECCPILSGLVELEAAACLCTTLKVKLLNLKIYVPLALQLLVTCGKSPPPGYTCS
I

>TA|CV013657(revision)||Theaceae_C.sinensis||HLTA
MESSKFTALLFISMLFISSTTPILGCGICGQPPKHKPGKVKPPKGPITVPPIVNPPITVP
PIVKPPVTVPPTVKPPVTLPPIPKPPVTLPPVTLPPIVNPPITLPPGILPPIKPLPPVTV
PPVTNPPTTKPCPPPPPKPCLPCLPPPPATCPIDTLKLGACVDLLGGLVHIGLGDPVVNE
CCPVLQGLVELEAAVCLCTTLKLKVLNLNIFVPLALQLLVTCGKTPPPGFTCTL

>TA|TA290_3605(revision)||Vitaceae_V.aestivalis||HLTA
MDSSKLSALFLIFMLFISSATPILGCAPCSKPPPQHKPPFHRKPPPHHKPPKGKPPITRP
PIVKPPITVPPVVPPIVKPPITVPPITVPPIVKPPITVPPITVPPITVPPIVKPPVTVPP
VTVPPITVPPIIKPPITVPPVVTPPVNKPPVGTPCPPPPASATCPIDTLKLGACVDLLGG
LVHIGLGDPVANECCPVLSGLVELEAAVCLCTTLKIKLLNLNIYVPLALQLLITCGKTPP
PGYTCTV

>NR|gi:225445607||Vitaceae_V.vinifera||HLTA
MDSSKLSALFLIFTLFISSATPILGCAPCSKPPPQHKPPFHHKPPSHHKPPKGKPPITRP
PIVKPPITVPPVVPPIVKPPITLPPVVPPIVKPPITVPPITVPPIVKPPITVPPITVPPI
VKPPITVPPITVPPIVKPPVTVPPITVPPIIKPPITVPPVVTPPVNKPPVGTPCPPPPAS
ATCPIDTLKLGACVDLLGGLVHIGLGDPVANECCPVLSGLVELEAAVCLCTTLKIKLLNL
NIYVPLALQLLITCGKTPPPGYTCTV

>TA|TA780_94328||Zingiberaceae_Z.officinale||HLTA
MASSNSRVVFIGSMLLVSSLLLPLTSDGAATCPPSSKPKYPKPPRHPHKPPKTKPPVIGK
PPYVHPPIGKPPITIPPVIGKPPVTVPPVIGKPPITVPPVIGNPPVTVPPVIGNPPVTVP
PVIGYPPITGPPVIQPPPVGGRTPCPPPPPLLPPANPPPASCPADSLKIGACVDLLGGLV
HIGLGDPVANQCCPLLQGLVELEAAVCLCTTIKLKLLNIKIYLPLALQLLLTCGKAPPPG
YTCSL

>TA|TA781_94328(revision)||Zingiberaceae_Z.officinale||HLTA
MDSSKSRGVVVFIGSMLLFSSLLLPLASAGAACPPTSKPKSPKAPRHPHKPPKTKPPVIG
KPPHVRPPIIGKPPITVPPIIGRPPITVPPIIGKPPITVPPVIGNPPVTVPPVIGNPPVT
VPPVIGYPPITGPPVIQPPPGGRSTPCPPPPPPPLPPVSPPPASCPADSLKIGACVDLLG
GLVHIGLGDPVANQCCPVLQGLVELEAAVCLCTTIKLKLLNINIYLPLALQLLLTCGKAP
PPGYTCAI

**@ HLTB Class [29 Proteins, 27 Species]**

>TC|TC8296(revision)||Amaranthaceae_B.vulgaris||HLTB
MGAYAYPYVVSLLVVLYFATFFTSLACPYCPYLPPPPKPPTSACPPPEHPPHTKPPPHTP
SGKPPSYPHKPPHSSHKPPQHSPHKPPQHSPYKPPYHTPPYTPKPSPPFVTPPYTPPPHG
FKPPIVTPPYTPPYSPKPPHHHPYKPPHYTPKPPKVAPPYTPKPTPHTPPPHGFKPPIVT
PPYTPPYTPKPPIVSPPYTPKPPVTPPYTPKPPIVSPPYSPPYTPKPPVVSPPYSPPYTP
KPPVVSPPYTPTPPIVSPPVVSPPYTPTPPIITPSPPSPVPSPETPCPPPPPPVPCPPPS
TPVQPTCSIDTLKLNACVDVLGGLIHIGIGSGAKGACCPILGGLVGLDAAVCLCTTIRAK
LLNINIILPLALQVLADCGKSPPAGFQCPSSY

>TA|DW167156||Asteraceae_L.virosa||HLTB
MRKLQWGFLVMLLLNLETLSIACPTCEPPSPPTKPPYLPPIMVKPQPSPPKFTPPVYKPP
PQSSPPSFPKPPTTSPAPPTIKPPYSPKPPFIHPPTVKPPTTTPSPTPPTTKPPSVHPPI
VKPPYFPKPPSVHPPVVKPPYFPKPPFLHPPVVKPPYFPKPPSVHPPVVKPPYFPKPPFG
HPPIVKPPYFPKPPFVHPPIVKPPFIHPPIDNPPLVRPPFVHPPIVKPPFVHPPFWPKPP
LLFPPEEIPPKIYPPSHPM

>TA|DR402466||Asteraceae_T.kok-saghyz||HLTB
MGLLKWGIFVILLLSLDTLSIACPTCEPPSPATKPPHAPPIIVKPPPSSPKLTPPVVKTP
PKSPPYFPKPPTTTPTPAPPTIKPPYFPKPPFVHPPIVRPPYVPKPPVVHPPVIKPPSIH
PPFVHPPIVKPPFVHPPVVKPPYVPNPPFVHPPIVPPIIVKPPPSSPKSPPPVVKTPPKS
PPYFPKPPTTTPTPAPPTIKPPYFPKPPFVHPPIGRPPYVPKPPVVHPPVIKPPSV

>TA|DV035554(revision)||Convolvulaceae_I.batatas||HLTB
MKNHLGKVLLLLLLQWGSLLVAHACPYCPYPPSPPKLHPKPPHPPKVPKHPPKVFPKPSP
CPLKPPVVHPPHVPKPPVVHPPHVPKPPVVHPPYIPKPPVVHPPHIPKPPVVHPPYIPKP
PVVHPPPIVPTPPVVTPPYVPKPPVVTPPVLPAPPPPVVITPPPYVPKPPAATPPPPVEK
PCPPPPPPVPCPPPPPPAQPTCPIDALKLGACVDLLGGLIHIGIGG

>TC|TC8573(revision)||Convolvulaceae_I.nil||HLTB
MKNHLGKVLLLLLLQWGSLLVAHACPYCPYPPSPPKVHPKPPLPPKVPKHPPKVHHEPPP
CPPKSPVVHPPHVPKPPVVHPPHVPKPPVVHPPHVPKPPVVHPPYIPKPPVVHPPYIPKP
PVVKPPPIVPTPPVVTPPYVPKPPVVTPPVLPPPPPVVITPPPYVPKPPVATPPPPVEKP
CPPPPPPVPCPPPPPPAQPTCPIDALKLGACVDLLGGLIHIGIGG

>TA|TA2504_3983||Euphorbiaceae_M.esculenta||HLTB
MAKYAFGNLLIILLHIGTLLSSLACPYCPYPTPPPKPPKYPPKFPPKHPPKVKPPPHHKP
PKPPKPPVHKPPYIPKPPVHKPPHVPKPPIVKPPTTPKPPHVPKPPITPKPPIVHPPYVP
KPPVVKPPPLVPKPPVVKPPYYPKPPIVYPPKPPTLPPKPPPYYPKPPIVYPPKPPTLPP
KPPVTPSPPTLPPKPPVTPTPPTLPPKPPVVPTPPTLPPKPPVVPTPPTLPPKPPVTPTP
PYYPSPPVTPAPPKPPVTPPVKPPSPPTETPCPPPPPPPVPCPPPPPKQETCPIDTLKLG
ACVDVLGGLVHIGVGSSAKDACCPVLQGLVDLDAALCLCTTIKAKLLNINIIIPIALEVL
VDCGKTPPPGFKCSA

>NR|gi:255559090||Euphorbiaceae_R.communis||HLTB
MAKYALANLFILLLNLGTLLTSLACPYCPYPTPPSKPPPGHPPKYPPRHPPKVKPPFHPK
PPKQPPHVKPPHPKPPHVPKPPIVKPPTIPKPPIVRPPYIPKPPVVNPPPFVPKPPVVNP
PPFVPKPPVVNPPPFVPKPPIVYPPNPPVTPPSSPPYIPKPPVVTPPIKPPTPPTLPPKP
PVITPPTKPPTPETPCPPPPPPPVPCPPPPPKQETCPIDTLKLGACVDVLGGLIHIGIGS
SAKDACCPVLQGLVDLDAALCLCTTIKAKLLNLNIIIPIALEVLVDCGKNPPPGFQCPA

>TA|TA1037_3818(revision)||Fabaceae_A.hypogaea||HLTB
MGNYVVTTILILLFNFSTLINLVISCPYCPYPTPKPPKHHDHPKLPPKHPPRVKPPIHKP
PKHNNPTPTTPCPPPKSPKPRPPYVPKPPVIVHPPYVPKPPYVPKPPVVRPPYVPKPPVV
TPPYVPKPPVVTPPYVPKPPPMVTPPYVPKPPVVTPPYVPTPSPPYVPKPSPPVVTPPYT
PRPPIIVHPPYVPLPPVVPTPPIISPPPYVPTPPVVTPPTPPSETPCPPPPPPAQPTRPI
DSLKLGACVDVLGGLIHIGIGGSAKQTCCPVLEGLLDLDAAVCLCTTIRAKLLNINIIIP
IALQLLIDCGKTPPDGFKCSPN

>NR|gi:255644756||Fabaceae_G.max||HLTB
MGKFALANILLVLLLNLSTLLNVLACPYCPYPSPKPPKKPPVVKPPVHKPPKPCPPPKSS
PKPPHVHPPYVPKPPHYPKPPVHPHPPHVPKPHPKPPVHPHPPYVPKPPPPVVPYPPPAQ
PTCPIDTLKLGACVDVLGGLIHIGIGSSAQQTCCPVLAGLVDLDAAVCLCTIIRAKILNI
NIIIPIALQLLIDCGKTPPDGFKCADS

>TC|TC333072||Fabaceae_G.max||HLTB
MGKIALANVLLVLLLNLSTLLNVLACPYCPYPSPKPPPKKPPIVKPPVHKQPKPCPPPKS
SPKPPHVHPPYVPKPPHYPKPPVHPHPPHVPKPHPKPPLHPHPPYVPKPPVVKPPYVPKP
PVVNPPYVPKPPVVPVTPPYVPKPPIVNPPYVPKPPVVPVRPPYIPKPPVVPVTPPYVPK
PPIVYPPVVPTPPIVTPPIVYPPVVPVPPLPPIVTPPNPPSETPCPPPPPPPVVPYPPPA
QPTCPIDSLKLGACVDVLGGLIHIGIGSSAKQTCCPVLAGLVDLDAAVCLCTTIRAKILN
INIIIPIALQLLIDCGKTPPDGFKCADS

>TC|TC48726||Fabaceae_L.japonicus||HLTB
MAKHSFAIVLILLLNLSTLLNIVLACPYCPYPAPSKPPKHPIVKPPVHKPPQHPPIVKPP
VHKPPQHPPIVKPPVHKPPKYPPSHGPEPCPPPKPSPKPPHYPKPPVVKPPHVPKPPVVK
PPHVPKPPIVKPPIVYPPPYVPLPPVVPSPPIVTPPTPTPPIVTPPTPTPPVVTPPSPPT
ETPCPPPPPVVPSPPPAQPTCPIDTLKLGACVDLLGGLIHIGIGGSAKQTCCPVLAGLVD
LDAAVCLCTTIRAKLLNINIIIPIALQLLLDCGKTPPDGFKCAAT

>TA|TA139_47247||Fabaceae_L.japonicus||HLTB
MAKHSFAIVLILLLNLSTLLNIVLACPYCPYPAPSKPPKHPIVKPPVHKPPQHPPIVKPP
VHKPPQHPPIVKPPVHKPPKYPPSHGPEPCPPPKPSPKPPHYPKPPVVKPPHVPKPPVVK
PPHVPKPPIVKPPYVPKPPHVPKPPIVKPPYVPKPPVVKPPPYVPKPPVVSPPYVPKPPV
VPVTPPYVPKPPVVPVTPPYVPKPPIVYPPPYVPKPPIVKPPIVYPPPYVPTPPIVKPPI
VYPPPYVPLPPVVPSPPIVTPPTPTPPIVTPPTPTPPVVTPPSPPTETPCPPPPPVVPSP
PPAQPTCPIDTLKLGACVDLLGGLIHIGIGGSAKQTCCPVLAGLVDLDAAVCLCTTIRAK
LLNINIIIPIALQLLLDCGKTPPDGFKCAAT

>NR|gi:3818416||Fabaceae_M.sativa||HLTB
MANYALANVFILLLNLSTLLIVLACPYCPYPSPKPPTHHPPIVKPPVHKRRKYSPTPKPP
VHKPPRYPPKPSPCPPPSSTPKPPHVPKPPHHPKPPVVHPPHVPKPPVHPPYVPKPPIVK
PPIVHPPYVPKPPVVKPPPYVPKPPVVRPPYVPKPPVVPVTPPYVPKPPVVRPPYVPKPP
VVPVTPPYVPKPPIVKPPIVFPPHVPLPPVVPSPPPYVPSPPIVKPPIVFPPHVPLPPVV
PVTPPYVQPPPIVTPPTPTPPIVTPPVVSPPTPPSETPCPPPPLVPYPPTPPAQQTCSID
ALKLGACVDVLGGLIHIGIGGSAKQTCCPLLQGLVDLDAAICLCTTIRLKLLNINLVIPL
ALQVLIDCGKTPPEGFKCPAY

>TC|TC139010||Fabaceae_M.truncatula||HLTB
MANYAIANVLILLLNLSTLLNVLACPYCPYPSPKPPSHHPPIVKPPVHKPPKHSPTPKPP
VHKPPRYPPKPSPCPPPSSTPKPPHHPKPPAVHPPHVPKPHPPYVPKPPIVKPPIVHPPY
VPKPPVVKPPPYVPKPPVVRPPYVPKPPVVPVTPPYVPKPPIVFPPHVPLPPVVPSPPPY
VPTPPIVKPPIVFPPHVPLPPVVPVTPPYVQPPPIVTPPTPTPPIVTPPTPPSETPCPPP
PLVPYPPPPAQQTCSIDALKLGACVDVLGGLIHIGIGGSAKQTCCPLLQGLVDLDAAICL
CTTIRLKLLNINLVIPLALQVLIDCGKTPPEGFKCPAS

>TC|TC511||Fabaceae_P.coccineus||HLTB
MGKFALPNLLLFLLFNFTTLLNVLACPYCPYPSPKPPKHHPIVKPPPVHKPPKPCPPPHS
SPKPPHVNPPYVPKPPHYPKPPVHPHPPYVPKPHPKPPLHPHPPYVPLPPVVKPPPYVPK
PHPKPPLHPHPPYVPLPPVVKPPPYVPKPPYVPNPPVVTPPYIPKPPVVTPPYIPKPPVV
PVIPPYIPKPPVVPVTPPYI

>TC|TC10515||Fabaceae_P.vulgaris||HLTB
MGKFALPNLLLFLLFNFTTLLNVLACPYCPYPSPKPPKHHPIVKPPPVHKPPKPCPPPHS
SPKPPHVNPPYVPKPPHYPKPPVHPHPPYVPKPHPKPPLHPHPPYVPLPPVVKPPPYVPK
PPYVPNPPVVTPPYIPKPPVVTPPYIPKPPVVPVIPPYIPKPPVVPVTPPYIPKPPVVPV
TPPYVPKPPVVPVRPPYVPKPPIVYPPVIPVPEPTPPTVTPPSPPSETPCPPPPPPVVPY
PPPAQPTCPIDSLKLGACVDVLGGLIHIGIGSSAKQTCCPVLAGLVDLDAAVCLCTTIRA
KILNINIIIPIALQLLIDCGKTPPDGFKCADS

>NR|gi:2578444||Fabaceae_P.sativum||HLTB
MANFAIANVLILLLNLSTLLNVLACPYCPYPSPKPPTHKPPIVKPPVHKPPKPQPCPPPS
SSPKPPHVPKPPHYPKPPAVHPPHVPKPPAVHPPHVPKPPVVHPPIVHPPYVPKPPVVKP
PVVKPPHVPKPPVVPVTPPYIPKPPIVFPPHVPLPPVVPVTPPYVPKPPIVFPPHVPLPP
VVPVTPPYVPKPPIVFPPHVPLPPVVPVTPPYVPLPPVVPVTPPFVPTPPIITPPTPTVP
VPSPPSETPCPPPPPTVVPYPPPAQPTCSIDALKLGACVDVLGGLIHIGIGGSAKQTCCP
LLQGLVDLDAAVCLCTTIRLKLLNINLVIPLALQVLIDCGKTPPEGFKCPSS

>TA|BE052756(revision)||Malvaceae_G.arboreum||HLTB
MGMHSLPYLIVLFLNLGALLTSLGCPECYRHPSPPPKCPPPGYPPKHHHPPIVKPPYHPK
PPKHHPPKPPKPPVVNPPYHPKPPKPPVVKPPYHPKPPKPPVVKPPYHPKPPKHPPHHPK
PPVVKPPYIPKPPIVKPPPYTPKPPVVKPPYIPKPPVVKPPPYTPKPPVVKPPPYTPKPP
VVKPSPYTPKPPYYPIPPVISPPTLAPKPPVYPSHQ

>TC|TC143442(revision)||Malvaceae_G.hirsutum||HLTB
MGMHSLPYLIVLFLNLGALLTSLGCPECYHHPSPPPKCPPPHYPPKHHHPPIVKPPYRPK
PPKHHPPKPPKPPVVNPPYHPKPPKPPVVKPPYHPKPPKPPMVKPPYHPKPPKHPPHPPK
PPVVKPPYVPKPPIVKPPPYTPKPPVVKPPPYTPKPPVVKPPPYAPKLPVVKPPPYTPKP
PYYPIPPVISPPTLPPKPPVYPSPPIVNPPPYTPKPPVVKPPPYTPKPPYYPIPPVISPP
TLPPKPPVYPSPPIVNPPTPPILPPPIVNPPTPPILPPPSPPIVNPPTPPIVKPPSPGTP
CPPPPPPAQQTCPIDTLKLGACVDVLGGLVHIGIGSSAKDTCCPVLQGLLDLDAAICLCT
TIKANLLNINIIIPIALQVLIDCGKTPPPGFQCPAQ

>TC|TC9||Malvaceae_G.raimondii||HLTB
MGMHSLPYLIVLFLNLGALLTSLGCPECYHHPSPPPKCPPPHYPPKHHHPPIVKPPYHPK
PPKHHPPKPPKPPAVNPPYHPKPPKPPVVKPPVVKPPYHPKPPKPPVVKPPYHPKPPKHP
PHPPKPPVVKPPYVPKPPIVKPPPYTPKPPVVKPPPYTPKPPVVKPPPYTPKPPVVKPPP
YTPKPPVVKPPPYAPKPPVVKPPPYAPKPPVVKPPPYAPKPPVVKPPPYTPKPPYYPIPP
VISPPTLPPKPPVYPSPPIVNPPPYTPKPPVVKPPPYTPKPPYYPIPPVISPPTLPPKPP
VYPSPPIVNPPTPPILPPPIVNPPTPPILPPPSPPIVNPPTPPIVKPPSPGTPCPPPPPP
AQQTCPIDTLKLGACVDVLGGLVHIGIGSSAKDTCCPVLQGLLDLDAAICLCTTIKAKLL
NINIIIPIALQVLIDCGKTPPPGFQCPAQ

>TC|TC6098||Malvaceae_T.cacao||HLTB
MMGKLNLANLIILLLNLGALLTSLACPECPHPTPPPKCPPPKYPPKHPPIVRPPFHPKPP
KHPPHPPKPPKPPVVKPPYVPKPPVVKPPPHIPKPPYVPKPPVVKPPPHAPKPPYVPKPP
VVKPPPYTPKPPYVPKPPVVKPPPYTPKPPVVKPPPYTPKPPVVKPPPYPPKPPVVKPPY
VPVPTPPYVPKPPPYVPKPPIVPVPKPPYVPKPPVVKPPPYYPIPPVISPPILPPKPPVY
PSPPIVKPPTPPVLPPTPPVYPSPPIVKPPTPPILPPKPPVYPSPPIVKPPTPPILPPTP
PIFPSPPIVKPPPIEKPCPPPPPPLPYPPPPAQQTCSIDTLKLGACVDVLGGLVHVGIGS
SAKDTCCPVLQGLLDLDAAIFLCTTIKAKLLNINIIIPIALQVLVDCGKTPPAGFQCPA

>TC|TC24140||Ranunculaceae_A.formosa||HLTB
MGKFIVANLLFILLNLSAILTSLACPYCPYSPPKHPKIPPRHPPKVKPPSVPKHPPPVVH
PPHVPKTPSKPPHVPKPPIVKPPCPPPPKSKSPPIVKPPHVPKPPIVKPPSAPTVPKPPP
VVKPPYVPKPPIVKPPSPPTVPMPPPVVNPPYVPKPPIVKPPPSPPIVTPSPPVPPPPPK
PTQETCPIDTLKLGACVDLLGGLIHIGIGGSAKDTCCPVLQGLVDLDAAICLCTTIKAKL
LNINIIIPIALEVLIDCGKNPPPGFKCPA

>TC|TC44790||Rosaceae_M.domestica||HLTB
MEINHALALLLVLILNLGSLLTSLANSYCPPPTYPPVPHLPPKHPPHVTPPYTPKPPVVL
PPPYTPKPPVVLPPPYTPKPPVVLPPPYTPKPPVVLPPPYTPKPPVVLPPPYTPKPPVVL
PPPYTPKPPVVLPPPYTPKPPVVLPPPYTPKPPVVLPPPYTPKPPVVLPPPYTPKPPVVL
PPPYTPKPPVVLPPPYTPKPPVVLPPPSPPVVVPSPPSTPTPTPVVPVKPPPPTETPCPP
PPPKVVPPPTSPPPKSPPKDTCPIDTLKLGACVDVLGGLIHIGIGSSAKDACCPVLQGLV
DLDAAICLCTTIKAKLLNINLIIPIALQVLIDCGKTPPSGFQCPA

>TC|TC2313||Rubiaceae_C.canephora||HLTB
MGNHNFARWFVLLLHLGTLLSSVACPYCPYPTPPHKPPHRPKPPAVKPPYTPKPPAVKPP
HKPKPPAVKPPYTPKPPAVKPPHKPKPPIVKPPIIVHPPYIPKPPFVHPPFIPKPPIIIP
PHPPIVKPPPFIPKPPVVLPPYVPKPPIVKPPPYVPKPPVVLPPYIPKPPIVKPPPYVPK
PPIVKPPPYVPRPPIVKPPPYVPKPPVISPPFVPKPPVVSPPYVPKPPVVVTPPPYVPKP
PVVSPPFVPKPPEVSPPYVPKPPEVSPPYVPKPPVVVTPPPYVPKPPPTPQPTPCPPPPP
TPVPCPPPPKKPEICPIDTLKLSACVDLLGGLVHIGIGSNVKSTCCPVLEGLVDLDAAVC
LCTTIKAKLLNINILIPIALEVLVDCGKTAPPGFQCSAD

>TC|TC31177(revision)||Rutaceae_C.clementina||HLTB
MGRKHPLANFLILLLNLGTLLSSLACPYCPYPTPPPPCPPTFPPKHPPHVKPPFHPKPPH
HKPPHVKPPHKPPYVPKPPYTPRPKPPYTPKPPYTPTPKPPYFPKPPYTPRPKPPYAPKP
PYTPTPKPPYFPKPPYTPRPKPPYAPKPPYTPTPKPPYFPKPPYTPRPKPPYVPKPPYTP
TPKPPYFPKPPYTPRPKPPYVPKPPYTPTPKPPYVPKPPYVPSPPYTPRPRPPYVPSPPY
TPSPKPPYVPSPPYTPSPTPPYVPSPPYTPSPTPPYVPSPPYTPSPSPPPPPSGTPCPPP
PPPTCPIDTLKLGACVDVLGGLVHIGIGSSAKDACCPVLQGLLDLDAALCLCTTIKLKLL
NINLIIPIALEVLIDCGKHPPPGFQCPA

>TA|CV709691||Rutaceae_P.trifoliata||HLTB
MGRKHPLANFLILLLNLGTLLSSLACPYCPYPTPPPPCPPTVPPKHPPLVKPPFHPKPPH
HKPPHVKPPHKPPYVPKPPYTPRPKPPYAPKPPYTPTPKPPYFPKPPYTPRPKPPYVPKP
PYTPRPKPPYVPKPPYTPRPKPPYVPKPPYTPRPKPPYVPKPPYTPRPKPPYVPKPPYTP
KPPYVPSPPYTPRPKPPYVPSPPYTPSPKPPFVPKPPYTPSPTPPYVPSPPYTPSPPY

>TC|TC12252(revision)||Solanaceae_N.benthamiana||HLTB
MEKFNVARVLLFLLQLGTLFIAHACPYCPYPPSTPKHPKLPPKHPSPRVKPPSTPHHPKQ
PPHVKPPSTPTHPKHPPHVKPPSTPKTPKHPTPKPCPPPLPPHVKPPHVKPPYVPKPPVV
HPPPTVSPPYVPKPPSPTPPVVSPPVTPKPPAPTPPVVSPPFIPKPPVVSPPFTPKPPAP
TPPVVSPPFVPNPPVVIPPPYVPNPPVVTPPTVKPSPPPPSPCPPPPPTIVPSPPAQQTC
PIDALKLGACVDVLGGLIHIGIGGSAKQTCCPLLMGLVDLDAAICLCTTIRLKLLSINII
LPIALQVLVDDCGKYPPKDFKCPSS

>TC|FG146646(revision)||Solanaceae_N.tabacum||HLTB
MEKFNVARILLFLLQLGTLFIAHACPYCPYPPSTPKHPKLPPKHPSPKVKPPSTPHHPKQ
PPHVKPPSTPTHPKYPPHVKPPSTPKTPKHPTPKPCPPPRPPHVKPPHVKPPYVPKPPVV
HPPPTVSPPYVPKPPSPTPPVVSPPVTPKPPAPTPPVVSPPVIPKPPVVSPPFTPKPPAP
TPPVVSPPVIPKPPVVSPPFTPKPPAPTPPVVSPPFVPNPPVVTPPPYVPNPPVVTPPIV
KPSPPPPSPCPPPPPTIVPSPPAQQTCPIDALKLGACVDVLGGLIHIGIGGSAKQTCCPL
LMGLVDLDAAICLCTTIRLKLLSINIILPIALQVLVDDCGKYPPKDFKCPSS

>NR|gi:157355516||Vitaceae_V.vinifera||HLTB
MGKHGLATWLVILLNFATLLTSLACSYCPSPTPPKPPKVKHPLPPLPPKHPPHVKPPHTP
MPPKPPAVKPPYVPNPPVVEPPYVPKPPVLNPPHVPKPPIVRPPVVKPPYVPKPPVVQPP
PPPVVHPPPPPTPCPPPPPPPKGRPPPQQTCPIDTLKLGACVDLLGGLVHIGIGSSAKDT
CCPVLQGLVDLDAAVCLCTAIKVKLLNVNIIIPIALQVLVGCGKTPPSGFQCPA

**@ HLTC Class [7 Proteins, 7 Species]**

>TC|TC8315||Amaranthaceae_B.vulgaris||HLTC
MDSSKSSALLFICMLFISSVTPILGCGYCGEPTHKPGKGGHHKPGKTPPGSVPKGPLPPV
TVPKVPIPPVTVPKVPIPPVTVPKVPIPPVTVPKVPVLPPVLPIPPVTVPKVPVLPPVLP
VPPVTVPKVPVLPPVLPIPPVTVPKVPGLPPLPLPPVLNPPSGPSGSPPSSPSGPCPPGT
PGSTPSGQPTCSIDILKLGACVDLLGGLVHIGLGDPAVNKCCPILQGLASSEARVCLCTS
LKLKLLNLNLYVPIALQLLLTCGKTPPPGFTCSV

>LOC|AT2G10940||Brassicaceae_A.thaliana||HLTC
MDSSKLSSLSLCLFLICIIYLPQHSLACGSCNPRKGGKHSPKAPKLPVPPVTVPKLPVPP
VTVPKLPVPPVTVPKLPVPPVTIPKLPVPPVTVPKLPVPPVTVPKLPVPPVTVPKLPVPP
VTVPKLPVPPVTVPKLPVPPVTVPKLPLPPISGLPIPPVVGPNLPLPPLPIVGPILPPGT
TPPATGGKDCPPPPGSVKPPSGGGKATCPIDTLKLGACVDLLGGLVKIGLGDPAVNKCCP
LLKGLVEVEAAACLCTTLKLKALDLNLYVPVALQLLLTCGKNPPPGYTCSI

>TC|TC64375||Brassicaceae_B.napus||HLTC
MESSKLSSLSLCFILICIIFFPQQSFSCGSCNHRKGGKPSIKPPVTVPKLPVPPVTVPKL
PVPPVTVPKLPVPPVTVPKLPVPPVTVPKLPVPPVTVPKLPVPPVTVPELPLPPVGGLPI
PPVGGLPIPPVGGLPIPPVGGLPIPPVGGLPTLPLPPLPIVGPLLPPGTNPPTPGGKDCP
PPPGSHKPPSGTGKATCPIDTLKLGACVDLLGGLVKIGLGDPAANKCCPLLKGLVEVEAA
ACLCTTLKLKALNLKLYVPVALQLLLTCGKNPPPGYTCSI

>TA|TA2271_3712||Brassicaceae_B.oleracea||HLTC
KLSSLSLCFILICIIFFPQQSFSCGSCNHRKGGKPSIKPPVTVPKLPVPPVTVPKLPGPP
VTVPKLPVPPVTVPKLPVPPVTVPELPLPPVGGLPIPPVGGLPIPPVGGLPIPPVGGLPI
PPVGGLPTLPLPPLPIVGPIFPPGTNPPTPGGKDCPPPPGSHKPPSGTGKATCPIDTLKL
GACVDLLGGLVKIGLGXXAAXXCCPLLKGLVEVEXXACLCTTLK

>TA|TA2302_3711||Brassicaceae_B.rapa||HLTC
MESSKLSSLSLCFILICIIFFPQQSFSCGSCNHRKGGKPSIKPPVTVPKLPVPPVTVPKL
PVPPVTVPKLPVPPVTVPKLPVPPVTVPKLPVPPVTVPKLPVPPVTVPELPLPPVGGLPI
PPVGGLPIPPVGGLPIPPVGGLPIPPVGGLPTLPLPPLPIVGPLLPPGTNPPTPGGKDCP
PPPGSHKPPSGTGKATCPIDTLKLGACVDLLGGLVKIGLGDPAANKCCPLLKGLVEVEAA
ACLCTTLKLKALNLKLYVPVALQLLLTCGKNPPPGYTCSI

>TA|TA480_57577||Fabaceae_T.pratense||HLTC
MESSKFYAYFIICMLFISSATPILGCGTCGNPPKKHKPGKKPIVKPPSVKPPVTIPPVTV
PPTLPVPNLPIPPLPHLPVPPVTVPNLPVPPVTVPNLPVPPVTVPNLPVPPVIPHLPNLP
VPPVTVPNLPVPPVTVPNLPIPPVLNPPSTGGSTPKGSTPKGSTPKGNCPPKTTPVKNTC
PIDTLKLGACVDLLGGLVHIGLGDPTANKCCPILQGLAEIEAAACLCTTLKLKLLNLNIY
VPLALQLLLACGKTPPPGYTCSL

>TC|TC4085(revision)||Orobanchaceae_T.pusilla||HLTC
MESSNKLLALILISMLSISSATDPIVEPECGYCSKPKPDKPSPKDPKLPVPPVTVPKLPV
PKLPVPPVTVPKLPVPKLPVPKLPVPPVTVPKLPVPKLPVPKLPVPPVTVPKLPVPKLPK
LPVPPVTVPKLPVPKLPKLPVPPVTVPKLPVPKLPKLPVPPVTVPKLPVPPVTVPKLPIP
PVTVPKLPVPLPNLPVPPVTEPKLPPSGKAGCPPPSSTTETCPIDTLKLGACVDLLGGLV
HIGLGDPTANECCPVISGLVEVEAAACLCTALKVKALNLEVYVPLAFQLLFTCGKTPPPG
YTCSI

**@ HLTD Class [11 Proteins, 4 Species]**

>NR|gi:42565109||Brassicaceae_A.thaliana||HLTD
MGSRSQNLSFLVLLLLGFVAVSYACDCTPPKPSPAPHKPPKHPVKPPKPPAVKPPKPPAV
KPPTPKPPTVKPHPKPPTVKPHPKPPTVKPHPKPPTVKPPHPKPPTKPHPHPKPPIVKPP
TKPPPSTPKPPTKPPPSTPKPPTTKPPPSTPKPPHHKPPPTPCPPPTPTPTPPVVTPPTP
TPPVITPPTPTPPVVTPPTPTPPVITPPTPTPPVITPPTPTPPVVTPPTPTPPVVTPPTP
TPPTPIPETCPIDTLKLGACVDVLGGLIHIGLGKSHAKAECCPVLGGLLDLDAAVCLCTT
IKLKLLNIDLVLPIALELLLDCGKTPPSDFKCPA

>LOC|AT3G22142||Brassicaceae_A.thaliana||HLTD||structural constituent of cell wall
MGFRTRNLSFLILLLLNFFAATYARECSPPKPSPKPHKPPKHSVVPPKPPAVKPHPHPKP
PTIKPPPPKRHPHPKPPTVKPHPHPKPPTKPHPHPKPPTKPHPHPKPPTIKPPPHPKPRP
HPKPPNVKPHPHPKPPTKPHPHPKPPTKHHPHPKPPTIKPPPKPPSVKPPPSTPKPPTTN
PPPSTPQPPTHKPPPCTPTPPVASPPMATPPTQMPPIATPPIAKSPVATPPIATPPTATP
PITIPPVATPPITTPPIANPPIIMPPIATPPVAAPPITNPPISKPPVTTPPTTTPPIAKP
PIATPPISTPPAATPPAATPPITTLPPAKPPVAISPIVTPPVTPIAQPPVATPPTATPPV
ATPPIATPPTSKSPISTPPISESPVATPPTATSPIKTPPPAKPPVATPPIAKSPIATPPT
ATPPVATPPIEKPPVATPPTTTPPTATPPVAKPPVETPPIATPPTAKPPISTPPISKPPV
ATPPAATPPITTPTPVKPPVATPPLAIPPVAKPPVVTPPTATPPIATPPIAKSPVATPPT
ATPPVATPPIAKPPVVTPPTTTPPTATPPVAKPPVATPPIATPPTAKPPISTPPISKSPV
ATPPAATPPITTPPPAKPPVATPPIATPPIAKPPVATPPTATPPIATSPVAKPPVATPPI
KTPPPAKPPVAIPPIATPPVAKPPVATPPTATPPIATPPIATPPVVTPPTATPPVATPPI
AKPPTTIPPTATPPVAMPPIATPPTAKPPIATPPIAIPPVAKPPVVTPPTATPPIATPPI
AKSPVATPPTATPPVATPPIAKPPVATPPTTAPPTATPPVAKPPVATPPIATPPTAKPPI
LTPPISKPPVATPPAATPPITTPPPAKPPVATPPIATPPIAKPPVATPPTATPPIATSPV
AKPPVAIPPIKTPPPAKPPVAIPPIATPPVAKPPVATPPTATPPIATSPIATPPVVTPPT
ATSPVATPPIAKPPTTTPPTATPPVAMPPIATPPTAKPPVATPPIANPPVEKPPVATPPI
AKPPTVLPPIAKPPVETSPTATPPTATPPVAIPPVVKPPVAIPPITKPPVATPPVTNPPT
AMPPIVTPPPIVTPPIAKSPIATPPVSTPPIAKPPIATPPVATTPIAKPPIATPPTANPP
VANPPIAKSPIAKPPIATPPTAMPSIATPPIGKPPVATPPMAKPPVASPPIATPPIIKPP
VATPPITKPPVATPPVATPPIAKPPVATSPIETPPVAKPPVTTPPVATPPIVKPPIVTPP
IATPPIAKSPIAPPPIGTPPIAKPPVATPPTATPPVATSPIAKPPVATPPPATPPVAKPP
VATPPTVTPPVATPPIAKPPGARPPVATPPVATPPIAKSPVATPPMTKPPVASSPIATPP
IAKTPIATPPTTMPKTCPIDTLKLGSCVDLLGGLVHIGIGKSAKEKCCPVVEGLVDLDAA
VCLCTTIKAKLLNIDVILPIALEVLLNCGKNPPPGFKCPA

>TC|TC64575||Brassicaceae_B.napus||HLTD
MGSRTQNLSFLILLFLGFLAVSFACDCSPPKPSPHPHKPPKHPSKPPKPPAVKPPKPPTV
KPHPHPKPPTKPPTVKPHPHPKPPTKPHPTPKPPTKPPTVKPPPSIPKPPTKPPTVKPPP
STPKPPTKPPTVKPPPSTPKPPTHKPPPVVTPPSPCPPPPVVTPPTPTPPVVTPPTPTPP
KPETCPIDTLKLGACVDVLGGLIHIGLGKTHAKEKCCPVLGGLVDLDAAVCLCTTIKSKT
SQHRPYH

>TC|TC93463||Brassicaceae_B.napus||HLTD
MALSLLLVFTFLQAPTNVVSAASKEESNALVSLPTSPTSPAIKPPSPSYKPPSFPTTPIK
PPTITPPVKPPTTPVPPTSPPTYKPPTVKPPTTTPVKPPPVQPPYKPPTPPVKPPTLSPV
KPPPAYKPPTPTVKPPTTPSVQPPMYKPPTPPVKPPTIPPVKPPTAPVKPTPIPPYKAPP
VKPTPPPPAKPPVNPIPSPPVNAPPVKPPSKPPTPPPVRPRINCVSLCGTRCGQHSRKNV
CMRACVTCCYRCKCVPPGTYGNKE

>TC|TC75207||Brassicaceae_B.napus||HLTD
MGSRTQNLSFLILLFLGFLAVSFACDCSPPKPSPHPHKPPKHPSKPPKPPAVKPPKPPTV
KPHPHPKPPTKPPTVKPHPHPKPPTKPHPTPKPPTKPPTVKPPPSTPKPPTKPPTVKPPP
STPKPPTKPPTVKPPPSTPKPPTHTPSPVVTPPSPCPPPPPSPCPPPPVVTPPTPSPPVV
TPPTPTPPKPETCPIDTLKLGACVDVLGGLIHIGLGKTHAKEKCCPVLGGLVDLDAAVCL
CTTIKAKLLNIDLIIPIALEVLVACGKTPPPGFKCPA

>TC|TC85796||Brassicaceae_B.napus||HLTD
MGILHKQNLSFLILLLLGFCAVSYACDCSDPPKSSPHPVKPPKHPIKPPKPPTVKPPQHT
PKPPAVKPPHHTPKPPHHTPKPPTVKPPHHTPKLPPHTPKPPTVKPPHHTPKPPTVKPPA
PYTPSPPPYTPKPPTVKPPPPYTPSPTPSPPPPTPTPPVVTPPPPPTPTPETPCPPPPPP
PTPCPPPPPAPTPEPETCSIDALKLGACVDVLGGLIHIGLGKSYAKATCCPVLGGLVGLD
AAVCLCTTIRAKLLNIDLIIPIALELLVDCGKTPPRDFKCPAPQKKTSLLA

>NR|gi:1155068||Brassicaceae_B.napus||HLTD
MGSHTQNLSFLILLLLGFLAVSFACECSPPKPSPRPHKPPKHPVKPPKPPAAKPPKPPAV
KPPKPPTKPPTLKPHPHPKPPTVRPHPHPKPPTKPHPIPKPPTIKPPPSTPKPPTKPPTV
KPPPSTPKPPTKPPTVKPPPSTPKPPTKPPTVKPPPSTPKPPTHKPPTVCPPPTPTPTPP
VVTPPTPPTPTPPVVTPPTPAPPVVTPPTPTPPVVTPPTPTPPVVTPPTPPVVTPPTPTP
PVVTPPTPPVVTPPTPTPPVVTPPTPPVVTPPTPTPPVVTPPTPTPPKPETCPIDTLKLG
ACVDVLGGLIHIGLGGSSAKKECCPVLGGLVDLDAAVCLCTTIKAKLLIVDLIIPIALEL
LIDCGKTPPPGFKCPS

>TA|TA3126_3711||Brassicaceae_B.rapa||HLTD
MGLLHKQNLSFVILLLGFLVVSYACDCGDPPKPSPHPVKPPKHPVKPPKPPTVKPPPHTP
RPPTVKPPHTPSPPHSFPPYTPKPPTVTPPPPYTPSPPPYTPKPPTVKPPPQPTPTPSPP
PPVRQATTSANPQRRHARHHRHRYHHQPHVFYTFSVNPEPETL

>TA|TA2187_3711||Brassicaceae_B.rapa||HLTD
MGSRTQNLSFLILLFLGFLAVSFACDCSPPKPSPHPHKPPKHPSKPPKPPAVKPPKPPTV
KPHPHPKPPTKPPTVKPHPHPKPPTKPHPTPKPPTKPPTVKPPPSTPKPPTKPPTVKPPP
STPKPPTKPPTVKPPPSTPKPPTHTPPPVVTPPSPCPPPPPSPCPPPPVVTPPTPSPPVV
TPPTPTPPKPETCPIDTLKLGACVDVLGGLIHIGLGKTHAKEKCCPVLGGLVDLDAAVCL
CTTIKAKLLNIDLIIPIALEVLVACGKTPPPGFKCPA

>TA|TA2103_3711||Brassicaceae_B.rapa||HLTD
MGSHTQNLSFLILLLLGFLAVSFACECSPPKPSPRPHKPPKHPVKPPKPPAAKPPKPPAV
KPPKPPTKPPTLKPHPHPKPPTVRPHPHPKPPTKPHPIPKPPTVKPPPSTPKPPTKPPTV
KPPPSTPKPPTKPPTVKPPPSTPKPPTKPPTVKPPPSTPKPPTHKPPTVCPPPTPTPTPP
VVTPPTPPTPTPPVVTPPTPAPPVVTPPTPTPPVVTPPTPTPPVVTPPTPPVVTPPTPTP
PVVTPPTPPVVTPPTPTPPVVTPPTPPVVTPPTPTPPVVTPPTPTPPKPETCPIDTLKLG
ACVDVLGGLIHIGLGGSSAKKECCPVLGGLVDLDAAVCLCPPSKPNFSLSTLLSHCS

>TA|DN774241||Brassicaceae_T.salsuginea||HLTD
MGSRTQHLSFVSFILLGFLAVSFACDCSPPKPSPHKPPKHPVKPPKPPVVKPKPPVKPPK
PPTKPPTVKPQPHPKPPTKPPTVKPHPPVKPPTKPPTVKPHPHPKPPTKPHPTPKPPTVK
PPTKPPTVKPPPSTPKPPTHKPPPTPCPPPKKPPTVKPPPSTPKPPTHKPPPTPC

**@ HLTE Class [6 Proteins, 5 Species]**

>TC|TC42393||Poaceae_P.virgatum||HLTE
MATSVRVLLLLAAVVLPVPGTLAWSNCPPPAPGGGGGHGPGRPWYPAPGSGGSGSPTRPS
PGSGSGGGGGHGKPPSSCPPCNPPYTPPTPRPSPPYVPPYTPPTPRPSPPYVPPYTPPTP
RPSPPYVPPYTRPTPRPSPPYVPPSPPYVPPSPPYVPPAPPYVPPTPPAGRTCPIDALKL
NACVDVLSGLIHLVIGREAKSKCCPLVQGVADLDAALCLCTTIRARLLNLNIYLPVALEL
LITCGKHAPPGFKCPPLYD

>TC|TC46939||Poaceae_P.virgatum||HLTE
MATTSAASVLVLLMMFAAVVLPGTLASSNCPPPAPGGGGGHGPRRPWYPAPGSGGGSGSR
PYPGSGSGGGHGKPPHHGKPPKHHGKPPSSCPPCNPPYTPPTPRPSPPYVPPYTPPTPRP
SPPYVPPYTPPTPRPSPPYVPPTPPYVPPYVPPSPPYVPPTPPYVPPYVPPSPPYVPPSP
PYVPPTPPYVPPYVPPSPPYVPPYVPPSPPYVPPTPTPTPPAGRTCPIDALKLNACVDVL
SGLIHLVIGREAKSKCCPLVQGVADLDAALCLCTTIRARLLNINIYLPVALELLITCGKH
APPGFKCPPLYD

>TC|CA206116(revision)||Poaceae_S.officinarum||HLTE
MATSTSAPVLLLLAAAVVLLLPSSLAWTSNCPPTAPGSGGHGHPSYPAPGSGSPTWPSPG
SGSSGGHSRPPHHGKPPKHHHGPPSNCPPCNPPYVPPTPRPSPPYVPPYVPPTPRPSPPY
VPPYVPPTPRPSPPYVPPYVPPTPRPSPPYVPPYVPPTPRPSPPYVPPYVPPTPRPSPPY
VPPYVPPTPRSSPPYVPAYVPPTPPYFPPYVPATPAYVPPTPPGLRTCPIDTLKLNACFD
FLNRLIHLGVGQEAMSKCC

>NR|gi:242094854||Poaceae_S.bicolor||HLTE
MATSTSVPVLLLLAAATGLLLPSSLASTSNCPPPAHGGSGGGGRVQPSYPAPASGSPKGY
RSGGGHSHPPHHGKPPKRHHGPPSNCPPCNPPYVPPTPRPSPPYVPPYVPPTPRPSPPYV
PPYVPPTPRPSPPYVPPYVPPTPRPSPPYVPPYVPPTPRPSPPYVPPYVPPTPRPSPPYV
PPYVPPTPRPSPPYVPPYVPPTPRPSPPNVPPSPPYVPPYVPPTPPYVPPTPPASRTCPI
DTLKLNACVDVLSGLIHLVIGQEARSKCCPLVQGIADLDAALCLCTTIRLRLLNINIYLP
IALNLLITCGKHPPSGFQCPPLYD

>TA|BF588202||Poaceae_S.propinquum||HLTE
SVPVLLLLAAATGLLLPSSLAWTSNCPPPAHGGSGGGGRVQPSYPAPASGSPKGYRSGGG
HSHPPHHGKPPKRHHGPPSNCPPCNPPYVPPTPRPSPPYVPPYVPPTPRPSPPYVPPYVP
PTPRPSPPYVPPYVPPTPRPSPPYVPPYVPATPRPSPPYVPPSPP

>NR|gi:226499476||Poaceae_Z.mays||HLTE
MATSTSVPVLLLLAAAAGLLLLPGSLAWQSSNCAPGAPGSGSPTWPSPGSGSGGGHSKPP
KHHHGKPPKCPPCNPPYVPPTPRPSPPYVPPYVPPTPRPSPPYVPPYVPVPPTPRPSPPY
VPPYVPVPPTPRPSPPYVPPYVPVPPTPRPSPPYVPPTPRPPTPPYVPPTPPYVPPTPRP
SPPPYVPPYVPPTPRPSPPYVPPYVPPTPPAVRTCPIDTLKLNACVDVLSGLIHLVIGQE
ARSKCCPLVQGVADLDAALCLCTTIRARLLNINIYLPIALNLLITCGKHAPSGFQCPPLY
D

**@ HLTF Class [3 Proteins, 3 Species]**

>TA|TA2014_3696||Salicaceae_P.deltoides||HLTF
MAKFAVANLLILLLNLGALLTSLACPTCPYTPHPKPPKRPPIKPPKPPVTPPIKPPKPPI
KPPKPPVTPPIKPPKPPIKPPKPPVTPPVIPIPPTLPPPKPPVTPPVIPTPPILPPPEPP
VIPTPPIVKPPPTPPKQETCPIDTLKLGACVDVLGGLVHIGIGSSAKDECCPLLEGLVDL
DAAVCLCTVIKAKLLNINLILPIALELLVDCGKNPPEGFKCPS

>TA|AJ774742||Salicaceae_P.euphratica||HLTF
MAKFAVANLLILLLHLGALLTSIACPSCPKPPKRPPIKPPKPPVTPPIKPPKPPIKPPKP
PVTPPIKPPKPPVKPPKPPVTPPIKPPKPPVTPPIKPPKPPVTPPVIPIPPTLPPPKPPV
TPPVIPTPPILPPPEPPVTPTPPIVKPPPTPPKQETCPIDTLKLGACVDVLGGLPHVGVG
SSAKDECCP

>TC|TC89836||Salicaceae_P.trichocarpa||HLTF
MAKFAVANLLILLLNLGALLTSLACPTCPYTPHPKPPKRPPIKPPKPPVTPPIKPPKPPI
KPPKPPVTPPIKPPKPPIKPPKPPVTPPVIPIPPTLPPPKPPVTPPVIPTPPILPPPEPP
VIPTPPIVKPPPTPPKQETCPIDTLKLGACVDVLGGLVHIGIGSSAKDECCPLLEGLVDL
DAAVCLCTVIKAKLLNINLILPIALELLVDCGKNPPEGFKCPS

**@ HPOA Class [28 Proteins, 25 Species]**

>TC|TC6439||Alliaceae_A.cepa||HPOA
MRTRFLLSILALLAFVNFCYSEADANTEKLVFVVGKSECLDCAKKNLKVEDAFQGLQVAI
KCRTSKGEYKLTTSGKLDKNGDYKVQLPSELVGEKNNLNQECFAQLHSVSNAPCPNKNGL
NPSKLIVKSTTEGVHTLSTSSNLPFSSATCTSAFFWPLPKWHPFYKPFPKHPLPSYPPLP
IYKKPCPPLPYYKPPVPAYEPPVPVYKPPTPVYEPPAPVYKPPTPVYEPSVPVYKPPTPV
YKPPVPVYKPPTPVYKPPTPVYKPPVPVYKPPTPVYKPPVPVYKPPTPVYKPPVKKPCPP
PVVEPPPVKKPCPPPVVEPPPVKKPCPPPVVEPPPVKKPCPSTCRGASHQ

>TA|DY803609||Asteraceae_T.officinale||HPOA
MRGSLFLLVLLFSGSLYYGNAKSVEVVGIGECADCKENNIETSHALSGLKVSVDCKQEKG
NFKTRGIAHMNEEGHFKVSLPQELLKDGKLAQECYVQLHNAANAPCAVHSGLEASKLTLL
SNSDESHTFGTTGKLKFSSSVCTSAFFWPPYKHPSIPVPTIPKDHPWFKKFHHMLPPLPK
IPFKKPCPPLPPLPPKILPPLPPKVLPPLPPVPTIPIKKPCPPPVVISKPPPVPVYKPTP
KPEPPVYKPEPKPKPPVYK

>LOC|AT2G21140||Brassicaceae_A.thaliana||HPOA||PRP2
MRILPKSGGGALCLLFVFALCSVAHSLSRDVKVVGDVEVIGYSEISKIKIPNAFSGLRVT
IECKAADSKGHFVTRGSGEVEETGKFHLNIPHDIVGDDGTLKEACYAHLQSAFGNPCPAH
DGLEASKIVFLSKSGANHVLGLKQSLKFSPEVCISKFWHMPKFPLPPPLNLPPLTFPKIK
KPCPPIYIPPVVIPKKPCPPKVAHKPIYKPPVPIYKPPVPIYKPPVVIPKKPCPPKIHKP
IYKPPVPIYKPPVVIPKKTFPPLHKPIYKHPVPIYKPIFKPPVVVIPKKPCPPLPKFPHF
PPKYIPHPKFGKWPPFPSHP

>LOC|AT4G38770||Brassicaceae_A.thaliana||HPOA||PRP4
MRILPEPRGSVPCLLLLVSVLLSATLSLARVVEVVGYAESKIKTPHAFSGLRVTIDCKVN
KGHFVTKGSGNIDDKGKFGLNIPHDIVSDNGALKEECYAQLHSAAGTPCPAHDGLESTKI
VFLSKSGDKHILGLKQNLKFSPEICVSKFFWPMPKLPPFKGFDHPFPLPPPLELPPFLKK
PCPPKYSPPVEVPPPVPVYEPPPKKEIPPPVPVYDPPPKKEVPPPVPVYKPPPKVELPPP
IPKKPCPPKPPKIEHPPPVPVYKPPPKIEKPPPVPVYKPPPKIEHPPPVPVHKLPKKPCP
PKKVDPPPVPVHKPPTKKPCPPKKVDPPPVPVHKPPPKIVIPPPKIEHPPPVPVYKPPPK
IEHPPIYIPPIVKKPCPPPVPIYKPPVVIPKKPCPPPVPVYKPPVVVIPKKPCPPLPQLP
PLPKFPPLPPKYIHHPKFGKWPPLPPHP

>TC|TC75752||Brassicaceae_B.napus||HPOA
MRILSGRGGGAFCLLFVIVLCSAVRSLGRDVDVVGFAESNKKIKSPHSYLGLRVTIECKA
AETKDHFVTRGSGEVDETGKFTLNIQHDDMFGEDGNLKEACYAQLHSASGNPCPAHDGLE
ASKIVILSKDGEKHVLGIKQNLKFSPELCFPKFLWDMPKFPLSPPLKLPPFPKIKKPCPP
KIKLPPFLPIYKPPVVIPKKPCPPKIAHKPSVPIYKPPVPIYKPPVPIYKPPVVIPKKPC
PPLPKPIYKPPVHIYKPPVVIPKKPCPPKAPVHYKPIYKPPVPIYKPPVVIPKKPCPPKA
KVPIYKPPVPIYKPIYKPPVVIPKKPCPPLPKLPPFPPKYIPHPKFGKWPPFPSHP

>TC|TC71296||Brassicaceae_B.napus||HPOA
MRILPEPRGSVPCLLLLLSVVFSATLSLARVVEVVGNAESKIKTPHAFSGLRVTIDCKVN
KGHFVTKASGNIDEDGKFGLKVPTHDIVSEDGALKEECYAQLHSAVGTPCPAHDGLESNQ
IVFLYKSGDKHVLGLKQNLKFSPELCVSKFFWPMPKFPPFKGFDHPFPLPPPLELPPFPK
PCPPPPVPVYEPPPKVEHPPPVPVYKPPPKVEHPPPVPVYKPPPKVEHPPPVPVYKPPPK
VEHPPPVPVYKPPPKVEHPPPVPVYKPPPKVEHPPPVPVYKPPPKVEHPPPVPVHKPPTI
PKKPCPPKSPKIELPPPVPVHKPPTKKPCPPKPPKKVDPPPVPVHKPPPKIVLPPPVPIH
KPPKKPCPPKAPKIKLPPPVPVYKPPPKIEHPPIYVPPVIPKKPCPPKAPKIDPPPVPVY
KPPPKIEHPPIYVPPVIPKKPCPPPVPIYVPPVVIPKKPCPPLPPLPKFPPLPPKYIHHP
KFGKWPPLPTHP

>TA|TA2996_3712||Brassicaceae_B.oleracea||HPOA
MRILPEPRGSVPCLLLLLSVVFSATLSLARVVEVVGNAESKIKTPHAFSGLRVTIDCKVN
KGHFVTKASGNIDEDGKFGLKVPTHDIVSEDGALKEECYAQLHSAVGTPCPAHDGLESNQ
IVFLYKSGDKHVLGLKQNLKFSPELCVSKFFWPMPKFPPFKGFDHPFPLPPPLELPPFPK
PCPPPPVPVYEPPPKVEHPPPVPVYKPPPKVEHPPPVPVYKPPPKVEHPPPVPVYKPPPK
VEHPPPVPVYKPPPKVEHPPPVPVYKPPPKVEHPPPVPVYKPPPKVEHPPPVPVHKPPTI
P

>TA|TA1223_3988||Euphorbiaceae_R.communis||HPOA
MWIRPVSRGAFLCFYLSLVLAATFCYGDEGNTVEVVGIGECADCAESNVKTSQAVSGLRI
TIDCKPENGEFKTRGVGELDEEGRFTVGLPSHLVKKGKLKEECYAQLHSASAAPCPTHSG
LESSKIVLKSKNNGKHTFGLAGKLRFSSVTCTSAFLWPHFKYPPLPKFPPLHKWKFPPLK
NFGHHPYLFPPKVFPPFPPKVFPPFPPKVYPPFPPKVYPPFPPKVYPPFPPKVFPPKPLP
PPVPVYKSPPKEFPPPVPVYKPPPKEFPPPVPVYKPPPKVLPPPVPVYKPPPKVLPPPVP
VYKPPPKVYPPPVPVYKPPPAPIKPLPPPVPTKKPCPPVKKPFPPKPPVYKPKPKPPVYK
PPPVPIYTPTPKPPVYKPPPVPIYTPTPKPPVYKPPPVPTYKPKPPTFYKPLPPIPKIPP
FHKKPCPPLPKLPPFPKIPPKYFHHHKFGKKWPPLPPFSPIH

>LOC|Medtr4g100690||Fabaceae_M.truncatula||HPOA
MQIFTLRQRALLCSWLFVVFLVLGFCYGGDHSTVEVVGLGECADCIQNNIKTSQAFSGLH
VTIDCKEANGHFKTRGAGELDKNGNFKVSLPQEIVNEGELKEECYAQLHSATAAPCPAHD
GLQNTKIVIKSKSDDKHTLSTAAAGKLKFSSATCTSKFFWPLFKHPLFPKLPHPDLPQFP
PKVFPTFPPKIFHKHPLFPPVPIYKKPPFSHIPIYKPPFPHIPIYKKPCPPPVPIYEKPI
PPPTPVYEKPLPPPTPVYEKPLPPPVPVYHKPLPPPTPVYEKPLPPPVPVYHKPLPPPTP
VYHKPLPPPVPVKKPCPPPKVEHPVLPPAPVYKPPPVPKPQPPPVPVKKPCPPPKVEHPV
LPPVPVYKPPPVPKALPPPVPIKKPCPPKIEHPVLPPVPIYKPPVVIPKPPVVPIYKPPV
ILPPFKKPPCPPLPTLPPLPPKSFFHHPKFGKLPPKSFFHHPKYGKWPPLPPKSFFHHPK
YFGKWPPIPHKSFFHHPKFGKWPPINPKSLFHHPKFGSWPPLSPHN

>TA|TA31_57577||Fabaceae_T.pratense||HPOA
MQIFTMNQRAVVCLWLSVCFLVLGFCYGDHSTVEVVGLGECADCEKNNIKTSQAFSGLHV
TIDCKEANGHFKTRGFGELDKNGNFKVSLPQDIVKEGELKEECYAQLHSATATPCPAHDG
LQNSKIVINSKSDDKHTLSTAAAGKLKFSSATCASEFFWHFYKHKHPLFPHVPIYKKPFP
HIPIYKPPFPHIPIFKKPFPPHEPIYEKPLPPPTPVYEKPVPPPTPVYEKPLPPPTPVYE
KPLPPPTPVYEPPPTPVYHKPLPPPTPVYEKPLPPPTPVYHKPHPPPVPVKGCPPKVEHP
ILPPAPVYKPPPVPKPQPPPVPVKKPCPPKVEHPILPPVPIYKPPVVVPKPPVVPIYKPP
VPVYKPPVPVYKPPVPIYKPPVPVYKPPVVIKPLPPXPKYPP

>TA|TA1237_3415||Magnoliaceae_L.tulipifera||p2vpvyk-containing
MSLLPHLLLLLLGVVALTTPSQAGYDYKPPEKKKPPPTPVYKLPPFKKPPPVYKPKPPPT
PVYKPPPVYKPKPPPTPVYKPPPVYKPKPPPTPTYKPPLVYKPKPPPTPVYKKPPFVKPP
PKKTPPSPYYKPPYKKPPPYGHPN

>TC|TC162759||Malvaceae_G.hirsutum||HPOA
MENLEFRGRALVAFTVWSLLFVTTFCNANGDGKTVEVVGVGECTDCKENNLDTTQAFSGL
RVTIDCKPENGDHFKTRGSGKLDKQGNFKVSLPQHLFKDGKLNEHCYAQLRTVSSPQPCP
SINGLESSKLVFKSTTDQKHQFGLKENLKFSPITCVSASFWPHHHHNFPPLPKLPPLPPL
KNFHHHYPHPPIYKKPLPPPVYKPHPVPVYKKPLPPPVPVYKPPVYKPHPVPVYKKPLPP
PVPVYKPPVYKPHPVPVYKKPLPPPVPIYKPKPFPPIPHKPLPPLPKIPPFTKKPCPPLP
KLPPKYFHHPKFGKWPPLPPFAPHHP

>TC|TC2435||Malvaceae_G.raimondii||HPOA
MENLEFRGRALVAFTVWSLLFVTTFCNANGDGKTVEVVGVGECTDCKENNLDTTQAFSGL
RVTIDCKPENGDHFKTRGSGKLDKQGNFKVSLPQHLFKDGKLNEHCYAQLRTVSSPQPCP
SINGLESSKLVFKSTTDQKHQFGLKENLKFSPITCVSASFWPHHHHNFPPLPKLPPLPPL
KNFHHHYPHPPIYKKPLPPPVYKPHPVPVYKKPLPPPVPVYKPPVYKPHPVPVYKKPLPP
PVPVYKPPVYKPHPVPVYKKPLPPPVPIYKPKPFPPIPHKPLPPLPKIPPFTKKPCPPLP
KLPPKYFPPPKFGKWPPLPPFAPHHP

>TC|TC25327||Ranunculaceae_A.formosa||HPOA
MRILSPASSGGTLKVTLVLLLLSFCYCHEKSVEVIGVGECSDCAQNNIENKHAVSGLPVA
IDCKAADGTFKTRGVGELDEEGKFKVVLPSEIVKDNGELEEECFAQLHSASSSPCATNNG
LEALKIVFKSKDNEKITFHPAGKLVFSPNTCTSAHLWPHFKYPPLPKLPPFPKVHPWYKK
PVPKMFNFPPHPPLVFPPFHKPPVYTKPPPTPVPSYSPPVYTKPPSPPVPTYNPPVYTKP
PVSPPVPIYKPPVYTKPPPVPKYKPPVYKKPCPPKSKPPVHKKPHPPSVPKYKPPVYKPH
PPSVPKYKPPVYKPLPPPVPIYKPPVYKPLPPPVPIYKPPVYKPLPPPVPIYKPPVHKKP
LPPPVPIYKPPVYKPLPPPVPIYKPPVYKPLPPPVPIYKPPVYKPLPPPVPAYKPPVVTK
PPPVPVYTKPPHPYFKPLPPFTKAPCPPFPKLPPFPKIPPTHPKFGHWPPLPPYSPHP

>TA|TA2040_57918||Rosaceae_F.vesca||HPOA
MQIRPAFHGALFGLWICLFTSTLCYGDDKTIEAAGQPGKVDFSELKTTDAAELNEEGKFA
VLHKVPILKKPFPKKPYFKKPPLHKIPTFKKPIPVFKIPPFKKHPSPIPQEEKPFLKKPF
FHPIPKFKKPYFHPIPVYKKPVSPPIPIYKKPPVPVYKIFPPIPVYKKPYFHPIPVYKKP
VFPPIPIYKKPPVPVYKIFPPIPVYKKPFFHPVPVYKKPYYPIPKYKKPCPPHVAP

>TC|TC34620||Rosaceae_M.domestica||HPOA
MPIRVAFQGALLGLWFCFFAASFCYSNGETIEVTGEQAAAKAFSELQRAEAAEVNEEEKF
KLPPHKPFFKKPAPPHIFPPKPFFKPLPPPAPVYKIPPYKKGHPPVPIVEEKPFLKKPIF
HPLPKFKKPLPPPIPVYKKPLPPPIPVYKKPLPPPVPVYKKPFPHPILKKPIPKFKKPLP
PPIPVYKKPLPPPIPVYKKPFPHPIFKKPIPKFKKPFPPHPFLGKPFPPHPFLGKPLPPT
VP

>TC|TC2588||Rubiaceae_C.canephora||HPOA
MRGVSHLLFLLVSIFFVVSFCRADDKTVQVVGAGECADCKDFKIKTSQAFSGLRVTVDCK
LKNGEVTRRGSGELDSEGKFRVSVPREILKDGKLDEECFAQLHSASATPCPAHNGLEANK
IVAKPDTDGKLTFGPAESVKFTSALCASAFLWPFFKNPPLPTGHPWFTHPPLPKLPFPPI
YKKPLPPIPIYKKPLPPPIPIYKKPLPPPVPIIKPPPIIKPLPPPVPIYKPPVYKKPLPP
PVPIYKPPVVKKPCPPPVPIYKPPVYQKPLPPPVPIYKPPVYQKPLPPPVPIYKPPVYQK
PLPPPVPIYKPPVYQKPLPPPVPIYKPPVPIYKPP

>TA|AJ775425||Salicaceae_P.euphratica||HPOA
MRILPVFRGALLCFYVSLVFGAAFCYADDSTVEVVGIGECADCAQSNMKTVHAFSGLKVT
IDCKPENGEFKTRGVGELDEEGKFKVSLPNDVVKDGKLKEECYALPPHVPIYKPKPKPKP
PIFKPPPVPIYKPRPKPPIFKPLPPPIPIYKPLPPAVPIYKPLPPIPKIPPFHKKPCPPL
PKLSPYPKLPPKY

>TC|TC92906||Salicaceae_P.trichocarpa||HPOA
MRILPVFRGALLCFYVSLVFAAAFCYADDSTAEVVGIGECADCAQSNIKTVHAFSGLKVT
IDCKPENGEFKTRGVGELDEEGKFKVSLPNDVVKDGKLKEECYAQLHSASAAPCPAHNGL
ESSRIVFKSKTDEKHIFGLAGKLKFSPVTCTSAFLWPHPPITKPLPLPTWKLPPLKNFHH
PYLFPPKVYPPLPPKVFPPLPPKVFPPIYKKPPLPHVPIYKPKPPIFKPPPVPIYKPKPK
PPIFKPLTPPTPIYKPLPPPVPIYKPLPPIPKIPPFHKKPCPPLPKLPPYPKIPPKYFHH
PKFGKWPPLPPYSPIH

>TC|TC24018||Solanaceae_C.annuum||HPOA
MYRMGLQSLCGRVLMGLSLSLLLLCSFCIADDKTIKVVGFGECADCKENNINTIHALSGL
HVSIDCKLENGEIKTRGEGELDKDGKFEVSLPKEMMKDGKLKEECYAQLHSASAAPCPAH
NGIESSKIVAIQTDGKHTLKPAGNLKFSTPLCTSAFLWPHFKYPPLPPFPKDHPWMKKFP
PWPPLNHWGHPFPIPPLPPLFKKPCPPPVVKPLPPPVPVYNPTPEPPVVKPLPPPVPVYK
PKPIPPPVPVYKPKPIPPPVPVYKPKPIPPPVPVYKPKPKPPPVPVYKPKPEPPVPKPKP
PPIKKPCPPKVPTYKPKPKPEPPVVKPLPPPVPVYVKPLPPPVPIYKKPCPPLIPKIPPF
HHIPLPPPVPIYKPPIVKPLPPPVPVYVKPLPPPVPIYKPPIVKPLPPPVPVYVKPLPPP
VPIYKKPCPPLKPFPKLPPFHHPLFPPLPPKVPHP

>TC|TC13506||Solanaceae_N.benthamiana||HPOA
MGLQSLCGRVLLGLSLTLLLSSFCFADDNIIKVFGIGECADCKENNINTIHAFSGLHVSI
DCKLENGQIKTRGEGELDKDGKFEVSLPKEMIKDGKLKEECYAQLHSASAAPCPAHNGIE
SSKIVIIKTDGKHSLKPTGNVKFSTALCTSAFLWPHFNYPPLPTLPPFPKDHPWMKHFPN
LPPWKHFNHPFPLPPIPPIFKKPCPPPIPKPNPPPVPVYNPTPEPPIVKPLPPPAPIYKP
KPKPEPPIVKPNPPPVPVYKPKPKPPSVPIYKPKPKPEPPVVKPNPPSVPIYKPKPKPPV
KKPCPPKPKPKPPVKKPCPPKPKPKPPVKKPCPPKPKPKPEPPVVKPLPPPVPVYKPPVV
KPLPPPAPVYEPPVVKPMPPPVPIYKPIPPFYKKPCPPLPPFPKLPPFHHPFFPPLPPSI
PHHP

>TC|TC40389||Solanaceae_N.tabacum||HPOA
MGLQSLCGRVLLGLSLTLLLSSFCFADDNTIKVFGIGECADCKENNINTIHAFSGLHVSI
DCKLENGEIKTRGEGELDKDGKFEVSLPKEMIKDGKLKEECYAQLHSASAAPCPAHNGIE
SSKIVITKTDGKHSLKPAGNVKFSTALCTSAFLWPHFKYPPLPKLPPFPKDHPWMKHFPN
LPPWKHFNHPFPLPSIPPIFKKPCPPPIPKPNPPPVPVYNPTPEPPVVKPLPPPVPIYKP
KPKPEPPVVKPNPPPVPVYKPKPKPPSVPIYKPKPKPEPPVVKPNPPSVPIYKPKPKPPV
KKPCPP

>TA|TA1376_62890||Solanaceae_S.habrochaites||HPOA
MGLQSLCGRVLLGLSLSLLLSTFCIADDNTIKVVGFGECADCKENNINTIHALSGLHVSI
DCKLENGEIKTRGEGELDQDGKFEVSLPKEMMKDGKLKEECYAQLHSASAAPCPAHNGIE
SSKIVSIKTDGKHTLKTAGNLKFSTPLCTSAFLWPYFKYPQLPPFPKDHSWMKKFPKLPP
FPPMPPLGHPFPLPPIPPFFKKPCPPPLEKPLPPPVPVYNPTPEPPVVKPLPPPVPVYKP
KPKPPPVPVYKPKPKPPPVPVYKPKPKPPPVPVYKPKPEPPVKKPCPPSVPKPKPPPVKK
PCPPKVPTHKPKPKPEPPVVKPLPPPVPVYKPPVVKPLPPPVPVYKPPIVKPLPPPVPIY
KKPSPPLPPFPKIPPFHHPLFPPLPPKVPHS

>TC|TC193197||Solanaceae_S.lycopersicum||HPOA
MGLQSLCGRVLLGLSLSLLLSTFCIADDNTIKVVGFGECADCKENNINTIHALSGLHVSI
DCKLANGEIKTRGEGELDQDGKFEVSLPKEMMKDGKLKEECYAQLHSASSAPCPAHNGIE
SSKIVSIKTDGKNTLKTAGNLKFSTPLCTSAFLWPYFKYPQLPPFPKDHSWMKKFPKLPP
FPPMPPLGHPFPLPPIPPFFKKPCPPPLEKPLPPPVPVYNPTPKPTPVVKPLPPPVPVYK
PKPKPPPVPVYKPKPKPPPVPVYKPKPKPPPVPVYKPKPEPPVKKPCPPSVPKPKPPPVK
KPCPPKVPTHKPKPKPEPPVVKPLPPPVPVYKPPVVKPLPPPVPVYKPPVVKPLPPPVPI
YKKPCPPLPPLPPIVKPLPPPVPIYVPPIVKPLPPPVPIYKKPCPPLPPFPKIPPFHHPX
FXXTPT

>NR|gi:3402282||Solanaceae_S.tuberosum||HPOA
MGLQSLCGRVLLGLSLSFLLSSFCIADDKTIKVVGFGECADCKENNINTIHALSGLHVSI
DCKLENGEIKTRGEGELDQDGKFEVSLPKEMMKDGKLKEECYAQLHSASAAPCPAHNGIE
SSKIITIKTDGKHTLEPAGNLKFSTPLCTSAFLWPYFKYPPLPQLPPFPKDHPWMKKFPN
LPPFPKDHPWMKKFPKLPPFPPMPPLKHWGHPFPLPPIPPLFKKPCPPPLVKPPVPVYNP
TPKPTPEPPVVKPLPPPVPVYKPKPKPPPVPVYKPKPKPPPVPVYKPKPKPPPVPTYKPK
PEPPVKKPCPPSVPKPKPPPVKKPCPPKVPTYKPKPKPEPPVVKPLPPPVPVYKPPVVKP
LPPPVPVYKPPVVKPLPPPVPVYKPPVVKPLPPPVPIYKKPCPPFPHLPPLPPIVKPLPP
PVPIYVPPVVKPLPPPVPIYEPPVVKPLPPPVPIYKPPFYKKPCPPLPPFPKIPPFHHPL
FPPLPPKIPHP

>TA|TA1723_246827||Vitaceae_V.shuttleworthii||HPOA
MQVLPISRGALLCFWVSLLFSASFCHGSVQKVEVVGIGECADCKQRNIKTSQAFSGLRVT
IDCKLAKGEFKTRAVGELDEEGKFKVSLPEEIVKDGELKEECFAQLHSASATPCPAHNGL
ESSKLILKTKVDGKHTFGPAGKLKFSPATCTSAFLWPYYKHPHLPKVSWPKLPPYSKSHP
WYKPKPKIYLPPIKFPPLPPKVYPPFTFPPLPPKVFPPLPPKVFPPPVPIYKKPLPPPVP
VYKKPLPPPVPVYKKPLPPPVPVYKKPLPPPVPVYQKPLPPPVPVYKKPLPPPVPIYKKP
LPPPVPIYKKPLPPPVPIYYKPLPPPVPVYKKPLPPPVPIYKKPLPPPVPVYKKPLPPPV
PVYKKPLPPPVPVYKKPLPPPVPVYKKPCPPEIPKILPPPIPIYKKPLPPLVPIYKPIPP
ISKPLPPFPPIYKKPWPPIPQVPPLPKFPPVHKFPPKYFHHPKFGFGSPVPPYSSHP

>NR|gi:225427928||Vitaceae_V.vinifera||HPOA
MGSPPICGGTLLGFWVFLLFSVIFCYGSADQFEVVGVVECADCSESRIKASPAFSGLGVT
IDCKLANGEFKTRAVGDVSNEGKFKVSLPEEMVKDGYQLKEECFAQLHSASAAPCPDAQS
GLEASKIVLKAKTIGKKNVFGPIANLKFSSLTCTSALWWSFYKHPPLPKLPLPFPKAYPP
PWKKFGHVYKKPLPPSIPIYKKPLPSPVHKKLLPPKVLIHKKPLPPKVLKPNKPFPPPSP
VYKKPFPPSVPVFKKPIPPPVPVYKRLPPPPAPVYQKRLPPPVPVFKKPCPPPVPIAKKL
LPPHVPIYKKPLLPPTPIYNKPNPPPFYKWSIHKPIPPVYKLLPPIPPLYKKPCPPILKP
PHYLPKLPPKLFPFPKFGPYPPIQPYFPHP

>NR|gi:147821777||Vitaceae_V.vinifera||HPOA
MQVLPISRGALLCFWVSLLFSASFCHGSVQKVEVVGIGECADCKQRNIKTSQAFSEMEGI
MASITSSWSPLSFHIQNQYSSGMKMACQKLNRKANLKLLPFRSWPVRSILHVAWFVFKYM
KNVILTPDGLRVTIDCKLANGEFKTRAVGELDEEGKFKLHSASATPCPAHNGLESSKLIL
KTKVDGKHTFGPAGKLKFSPATCTSAFLWPYYKHPQLPKVSWPKLPPYSKSHPWYKPMPK
IYLPPIKFPPLPPKVYPPFTFPPLPPKVFPPPVPIYKKPLPPPVPVYKKPLPPPVPVYKK
PLPPPVPVYKKPLPPPVPIYKKPLPPPVPIYKKPLPPPVPIYKKPLPPPVPIYKKPLPPP
VPIYKKPLPPPVPVYKKPLPPPVPVYKKPLPPPVPVYKKPLPPPVPIYKKPLPPPVPIYK
KPLPPPVPVYKKPLPPPVPVYKKPLPPPVPVYKKPLPPPVPVYKKPCPPEIPKILPPPIP
IYKKPLPPFVPIYKPIPPISKPLPPFPPIYKKPWPPIPQVPLPKFPPVHKFPPKYFHHPK
FGFGSPGIIRGLGFPIWTQPICERGTSRPLGGTAIFCELLPLQCADCAESNINTSQALSG
LGLTIDCKLENGEFKTRKVGDINQEXKLQFKEEYLTQFLSASAAPCPAQSGLQASKVLPI
FQWAGLGFFISLLFAVSFCHGNNQEVMVVGTGECADCDHSNIKTSQAFSGLGVTIECKLE
SGEFKTRAVGELDEEGEFKLPLPKEIVKGGKLKEECFAQLHSASAKPCPIRNGPEASKII
LKTTNNGKQVFAPAGKLKFSPVTCTSAFLWPFYKYPPLPTLPHWPKPLPKFYLPPFSPVP
TPITYPAPEADPPVSHPPPPPVYKQQIPPSVPPYTAPSPPQVYDQPSPQPVYYEPHPPPV
PIYHKPLPPPVRVYGQPFPSPVPVYKKPLPPPVPIYKKPPLPSLPPPVPVHKKSLPPPVP
KPPLPPPVPVSQEPLPPPSPVYNEPLPPSPVPVLKKPIPPIVEKPLPPPVPIFKKPALPP
PVPVYKKPPLPPPPPPVPVYGEPLPPPVPAFKKPYPPPLPIVEKPLPPPIPIHKPIPPIS
KPAPTPEVLPAPPPIYSPKPPILNPTPKPKVLPPPQPVPITKKPLPPPVPIQKPVPAAQN
PPPVYKKPLPPIPKAPALPKLPPLPKTPPKYFYHPKYGLGNAKPPSSSHP

**@ HPOB Class [5 Proteins, 5 Species]**

>NR|gi:115481164||Poaceae_O.sativa||HPOB
MAGAPRGLVLLGVCAVLMVLAVSGEAASVVIGTAKCADCTRKNMKAEDAFKNLQVAIKCK
NGNGEYESKAAGKLDGSGAFSVPLDTDLHSSDCIAQLHSATNEPCPGQEPSKIVPLSEGT
FVTVAGKTSYPSALCASATICGPIKKKIIDHFHKKPVPPKPDPKPEPPKPKPEPEHPILD
HFHKKEKDFFDHFHKKPVPPKPEPKSEPKPQPKPQPAPEYHNPSPPAKH

>TC|TC12532||Poaceae_P.virgatum||HPOB
MAGAPRGLLLGVCAALMVVAIANAANGNAASVIVGLAKCADCTRKNLKAEAAFKGLQVAI
KCKNSKGDYESKAVGELDGSGAFNVPLTTDLHGADCFAQLHSAVGTPCPGQEPSRIVPQS
ESHFVVVPGKTNNPSAECASVTICGPIKKHFMDHFHKKPVPPKPKPEPKPQPEYHPPTPT
YGSPTPIYHPPARHLFDKKKLLDHFHKDHDHHPFLDHFHKKPVPPKPKPEPKPQPEYHPP
TPTYGSPTPIYHPPARHLFDKLLDHFHKDHHHHHLFDHFHKKPVPPKPEPKPQPEYHPPT
PTYGSPTPIYHPPTKHLSDKKHWLDHFHKEHEHHHFFDHFHKKPVPPKPKPEPMPKPQPE
YHPPTPTYSSPTPTYGSPTPVYHPPAKH

>TC|TC97814||Poaceae_S.officinarum||HPOB
MAAALGGLLPGVCAVLMVIAVASAASSETSSLVVGLAKCADCTRKNMKAEAAFKGLEVAI
KCKNSKGEYETKAMGKLDGSGAFSIPLSTDLHAADCVARLHSAAGTPCPGQEPSRIVPQS
SEGNFVVVPGKTDYPSAECASATLLGPIKKHLLDHFHKKTVPPKPKPEPKPQPEYHHPTP
TYGSPTPIYHPPARQLFDKKHILDHFHKDHDYHHFFDHFHKKPVPPKPKPEPKPKPEPKP
QPEYHSPTPTYSSPTPIYHPPARHLFDKKPLLDHFHKDHDYHHFFDHFHKKPMPPKPKPE
PKPQPEPEPDHFHKGHDYHHFFDHFHKKPVPPKPKPEPKPQPEPEYHSPPTYGSPTPIYH
PPARHLFDKKPLLDHLHKDHNYHHFFDHFHKKPMPPKPKPEPKPQPEPEYHHPTPTYGSP
TPIYHPPSLDKKPLLDHFHKDHDYHHFFDHXHKKPVLTKPKPEPKPQAEPEYQPPTPT

>NR|gi:242039895||Poaceae_S.bicolor||HPOB
MAAVHGGLLPGIFAVLMVIAVASAASSEASSVVIGLAKCADCTRKNMKAEAAFKGLEVAI
KCRNSKGEYESKAIGKLDVSGAFSIPLSTDMHAADCVAQLHSAAGTPCPGQEPSRIVPQS
SDGNFVVVPGKTDYPSAECASATLCGPIKKHLLDHFHKKPVPPKPKPEPKPKPEPKPQPK
YHSPTPTYRSPTPIYHPPARQLFDKKHMVDHFHKDHDYHHFLDHFHKKPSPLKPKPEPKP
EPEPKPHHFFDHFHKKPVPPKPKPEPKPQPEYHPPTPTYGSPTPIYHPPARHLFDKKPLL
DHFHKDHDYHHFFDHFHKKPVPPKPKPEPKPQPKPQPEPDHFHKGHDYHHFFYHFHKKPV
PPKPKPEPKPQPEPEYHPPTPTYGSPTPIYHPPARHLFDKNPLHDDFHKHHDYHHIFDHF
HKKPVPPKPKPQPKPEYHPPTPTYGSPTPIYHPPVVKEISFDKKHFLDHFHKDHDYHKFF
DHFHKKPVPPKPKPEPKPKPEPEYHPPAPTYASPTPIYHPPAKN

>NR|gi:162461641||Poaceae_Z.mays||HPOB
MAADALRGLLPGVYAVLMVIAVTSAASSDASSVVVGLAKCADCTRKNLKAEAAFKGLEVA
IKCKNSKGEYESKAMGKLDGFGAFSIPLSTDLHGADCVAQLHSAAGNPCPGQEPSRIVPQ
SSKGNFVVVPGKTDYPSKECASATLCGSIKKHLLDHFHKKPVPPKPKPEPKPELEPKPEP
KPEPENHPPTPTYGSSTPIYHPPSRQLFDKKHMFDNFHKHHDYHHFFDHFHKKPVPPKPK
PEPKPEPKPKPQPEYHSPTPTYGSPTPIYHPPARHLFDKKPLLDHFHKDHDYHHFLDHFH
KKPVPQKPKLEPKPQPKPEYHPPTPTYVSPTPIYHPPAETEMKYDKKHFDHFHKDHDYHH
FFDHFHKKPVPPKPKPEPEPKPKPQPKPEYKPPMPTYGSPTPIYHPPAKKEVAFDKKHFL
DHFHKDHDYHHFFDHFHKKPVPPKPKPEPKPEPKPQPEPEYHPPTPTYGSPTPIYHPPAK
H

**@ HPOC Class [14 Proteins, 12 Species]**

>NR|gi:11322245||Apiaceae_D.carota||HPOC
MTALSPKPIGLLHLALLFSTFVFALGTATIAQPPTALAPAPAPHHHKPGGHHHHHKHAPT
PAPAPLTKPPTHAPAPAPVKPPVKPPVQPVKPPVKAPSHAPTPLPARKLVAVQGVVYCKP
CNYTGVETLLGATPLLGAVVKLQCNNTKYPLVVQGKTDKNGYFSLNAPKTITTYGVHKCR
VFVVSSPEKKCDKPTNLRYGVKGAILEKSTKPPVSTKTPATFEMFSVGPFAFEPSTKKPC
SH

>TA|EH724638||Asteraceae_C.maculosa||HPOC
MEFRVLFSMALLSSCLFINTVFCDDDGVDDNVSLAHAPSYQPAAAPAPHHHHHHHHHHQK
LAASPPMAHAPSKAPVYAPIQPPTQAPVKTPSKAPVKTPSKAPVQPPTKAPVQPPSSSPA
PVHAPLPARKLVAVQGVVYCKACKYKGVDTLLGATPLQGAEVLLTCNNTKYPLRVKGTSD
KNGYFFIKPPKTLTTYGFHKCNVRLMTSPMASCNEPTDLHGGVKGSMLVPNKKPRVSNPD
AHPLPYEVFTVGPLAFE

>LOC|AT5G14920||Brassicaceae_A.thaliana||HPOC||gibberellin-regulated family protein
MALSLLSVFIFFHVFTNVVFAASNEESKALVSLPTPTLPSPSPATKPPSPALKPPTPSYK
PPTLPTTPIKPPTTKPPVKPPTTSVTPVKPPVSTPPIKLPPVQPPTYKPPTPTVKPPSVQ
PPTYKPPTPTVKPPTTSPVKPPTTPPVQSPPVQPPTYKPPTSPVKPPTTTPPVKPPTTTP
PVQPPTYNPPTTPVKPPTAPPVKPPTPPPVRTRIDCVPLCGTRCGQHSRKNVCMRACVTC
CYRCKCVPPGTYGNKEKCGSCYANMKTRGGKSKCP

>LOC|AT1G28290||Brassicaceae_A.thaliana||HPOC||AGP31
MGFIGKSVLVSLVALWCFTSSVFTEEVNHKTQTPSLAPAPAPYHHGHHHPHPPHHHHPHP
HPHPHPPAKSPVKPPVKAPVSPPAKPPVKPPVYPPTKAPVKPPTKPPVKPPVSPPAKPPV
KPPVYPPTKAPVKPPTKPPVKPPVYPPTKAPVKPPTKPPVKPPVYPPTKAPVKPPTKPPV
KPPVSPPAKPPVKPPVYPPTKAPVKPPVSPPTKPPVTPPVYPPKFNRSLVAVRGTVYCKS
CKYAAFNTLLGAKPIEGATVKLVCKSKKNITAETTTDKNGYFLLLAPKTVTNFGFRGCRV
YLVKSKDYKCSKVSKLFGGDVGAELKPEKKLGKSTVVVNKLVYGLFNVGPFAFNPSCPK

>TC|TC90718||Brassicaceae_B.napus||HPOC
MGFLGKSVLVCLIALWCFTSSAFTEEVNHVTQTPSSAPAPAPYHHGHHHHHPHPPHPHHP
HPHPPAKAPVKPPVYPPTKAPVKPPVYPPAKAPVKPPTKPPVKPPVSPPAKPPVKPPVYP
PTKAPVKPPVYPPTKAPTKPPTKPPVKPPVSPPAKPPVKPPVYPPKFNRSLVAVQGTVFC
KSCKYASYDSLTGAKPVEGAKVRLVCKSKKNIVAETETDKNGYFMLLAPKTVTNFGFRRC
RAYLVKSKDYKCNKVSKLFGGDVGAVLKPVKTPGKSSVVINKLTYGVFNVGPFAFDPVCS
K

>TC|TC71449||Brassicaceae_B.napus||HPOC
MGFLGKSVLLSILAIWFFTSCAFTEEVNHVTQTPSSAPAPSPYHHGHHHPHPPHHHHPHP
HPPAKAPVKPPVSPPSKPPVKTPVYPPTKSPVKPPTKPPVKAPVSPPAKPPVKPPVYPPT
KAPVKPPVKPPVKPPVSPPAKPPVKPPVYPPTKAPVKPPVKPPVKPPVSPPAKPPVKPPV
SPPAKPPVKPPVYPPTKAPVKPPTKAPVKPPVSPPTKPPVSPPAKPPVRPPVYPPKFNRS
LIAVQGTVFCKSCQYASSDSLIGAKPVEGAVVRLLCKSKKNVVAETKTDKNGYFLLLGPK
TVTNYGFRGCRVYLVKSKDYKCNKVSKLFRGDVGAVLKPEKRKGKSAVVINQLIYGIFNV
GPFAFDPVCPK

>TA|TA3066_3712||Brassicaceae_B.oleracea||HPOC
MGFLGKSVLLSLLAIWFFTSCAFTEEVNHVTQTPSSAPAPSPYHHGHHHPHPPHHHHPHP
HPPAKAPVKPPVSPPSKPPVKTPVYPPTKSPVKPPTKPSVKPPTKPPFKPPTKPPVKPPV
SPPAKPPVKPPVYPPTKAPAKPPVKPPVKPPVSPPAKPPVKPPVYPPTKAPVKPPVKPPV
KPPVSPPAKPPVKPTVSPPAKPPVKPPVYPP

>TA|TA2657_3711||Brassicaceae_B.rapa||HPOC
MGFLGKSILSSLLAIWFFTSCAFTEEVNHVNQTPSSAPAPSPYHHGHHHPHPPHHHHPHP
HPPAKAPVKPPVSPPSKPPVKPPVYPPTKSPVKPPTKPPVKPPVSPPAKPPVKPPVYPPT
KAPVKPPVKPPVKPPVSPPAKPPIKPPVSPPAKPPVKPPVYPPTKAPVKPPTKAPAKPPV
SPPAKPPVSPPAKPPVKPPVKPPVYPPKFNRSLIAFQGTVFCKS

>TA|DN778269||Brassicaceae_T.salsuginea||HPOC
MGFLGKSVLVSLIALWCFTSSAFTEEVNHVTQTPSSAPAPAPYHHGHHHPHPPHHHHPHP
HPHPPAKAPIPPPAKPPVKPPTKAPVKPPVKPPVKPPAKAPVYPPTKAPVKPPVKPPVMP
PVSPPAKPPVKPPVYPPTKAPVKPPTKAPVKPPVSPPTKPPVSPPAKPPVMPPVYPPKFN
RSLVAVRGT

>NR|gi:255646492||Fabaceae_G.max||HPOC
MAKALASVLLFLLVAVSNVFAVELELETLPPSCPHPHPVHSPTPAPLHPPANAPHPHHHH
HHHPPAPAPAPAPVPSPSHHNYPPTPAPAKPPTHHHHHHPPAPVNPPPVPVHPPVKPPVP
VHPPVKPPVPVHPPVKPPVPAHPPVKPPVVPVHPPVKPPVVPVHPFPRSFVAVQGVVYVK
SCKYAGVDTLLGATPLLGAVVKLQCNNTKYKLVQTSKFDKNGYFYIEAPKSITTYGAHKC
NVVLVSAPYGLKASNLHGGVTGALFRPEKPFLFKRLPFVLYTVGPLAFEPNCH

>TC|DV709394||Rubiaceae_C.canephora||HPOC
MASFSAKIPVLMQLSMLLLISSSVFADQHEHSKWPAHPPAEAPEHHKGHHHHHHHHHPPT
YPPVKPPVHPPVKPPVHPPVKNLVHPQGYPHGKTRIHPGVNPHEPPPLKAA

>TC|TC25156||Solanaceae_C.annuum||HPOC
MGFVPTKSIGFIVALLLVTSFTVLGQNVDGIVTSELAPHSSVSSPPVEAPKPHKGGHHHH
HHHHQHSPAPAPPPSHSSPPPSPPVKPPYPPAHSPSKPPSPPVKPPVKPPTPPAHSPSKP
PSPPVKPPVKPSPPVKPPTPPAHSPSKPPSPPVKPPVKPSPPVKPPVKPYPPVKPPTPPP
MRMFVGVRGVVYCKSCKYRGVDTLLGASPIQGAVVKLACNNTKFHLTSLGTTDKNGYFYI
QPKWLTTAGYHKCKVFLAKSSKAECSVPTDFHNGQSGAILVPAPPSPMASSKPVNNSEPG
VKLFDVGPFAFEPSKNLPCKH

>NR|gi:312836||Solanaceae_N.alata||HPOC
MAKALVLFQLSVLLLSSFTVVLSEEEGIGGWFVDKHHDHLSPAQAPKPHKGHHHPKHSPA
PSPTKPPTYSPSKPPVKPPVKPPTKPPTYSPSKPPAKAPVKPPTPSPYPAPAPITRKPVA
VRGLVYCKPCKFRGVKTLNQASPLLGAVVELVCNNTKKTLVEQGKTDKNGFFWIMPKFLS
SAAYHKCKVFLVSSNNTYCDVPTDYNGGKSGALLKYTPLPKPPAASSLPVKLPTFDVFTV
GPFGFEPSKKVPCKK

>TC|TC40573||Solanaceae_N.tabacum||HPOC
MAKALVLFQLSVLLLSSFTVVLSQEEGIGGWFIDKHHGHFPPAEAPKAHKGYHHPKYSPA
PSPSYTPSKPPTKPPTYSPSKPPAKPPVKPPTKPPTYSPSKPPAKPPVKPPTPSPYPAPI
TRKPVAVRGLVYCKPCKFRGVKTLNQASPLLGAVVKLVCNNTKKTLVEQGKTDKNGFFWI
MPKLLSSGAYHKCKVFLVSSNNTYCDVPTDYNGGKSGALLKYTPLPKPPAASSLPVKPPT
YDVFTVGPFGFEPSKKVPCKK

**@ PELPK Class [107 Proteins, 61 Species]**

>TC|TC7802||Aizoaceae_M.crystallinum||PELPK
MASRLFLFGVMAVACMISTATMPVAARRLLENSVPEIPKPELPKPEVPQVPEIPKPTVPE
LPKPTLPELPKPVVPELPKPTLPELPKPNVPDLPKPTLPELPKPKVPELTKPTVPELPKP
TVPELPKPTLPELPKPSVPEVPKPTMPELPKPTIPEVPKPTLPELPKPVVPEVPKPTLPE
MPKPTVPEVPKPTLPEMPKPVVPEVPKPAVPEMPKLTVP

>TC|CF438076||Alliaceae_A.cepa||PELPK
HLLAVLLFLFSNSITNDAVRMLLEAPTIPKPELPSLPDIPTLPKPELPKLPPLPEMPSLP
KLEIPALPELPKLPSITKTEETPVVPEIPTLPKQEVPSLPKIPSLPKLELPPLPEIPSLP
KFELPPLPQLPTLPEISAHPSAAP

>TA|EL403734||Asteraceae_C.tinctorius||PELPK
MAHSHLTSFLLILVSITLVSIICAEARNLLEILEVPKPEFPVLPKPEVPEIPKPTVPEIP
KPTLPEIPKPTIPKIPKPKIPESPKPTLPEIPKPTVPEIPKPTIPEIPKPTVPEIPKPTV
PQIAKPTIPEIPKPTVPEILKPTLPEIPKPTIPDIPKPVVPEISKPTVPEISKPAVSEIP
QPTIPEIPKPMLPVLPKPELSTLPKPEVPKVPEIHDLPKPTLPTIPVLPKDFPIPSQPHP

>TA|EH761007||Asteraceae_C.solstitialis||PELPK
MAHSHLTSFLLILVSISLASICTDGIICAEARKLLEIPEIPKPELPVLPKPTVPEIPKPT
LPDIPKPTIPEIPKPTAPEIPKPTLPEIPKPTVPEIPKPNIPEIPKPTVPEIPKPTVPEI
PKPTVPEIPKPTIPEIPKPIVPEITKPTVPEIPKPTIPDIPKPTVPEIPKPTLPEIPNPI
VPEIPKPTVPEIPKPTLPEIPKPIVPEIPIPTLHEIPKPTPPDIPKPIVPEIPK

>TC|TC41677||Asteraceae_H.annuus||PELPK
MACYKGSSFIMILLFVTLVSTGGGKRFAEARNLLEVPDLPKPTLPEIPKPTLPVIPKPIL
PELPKPIIPEIPKPTLPEVPKPEVPELPKPTIPEIPKPILPEVPKPEVPVVPKPEVPELP
KPTIPEIPKPTLPEVPKPEVPVVQKPEVPELPKPTIPELPKPTFPEIPKPELPTLPKPEV
PKVPEVHELPKPTIPELPKDFPIPSLPHP

>TA|EE606571||Asteraceae_H.argophyllus||PELPK
MACYKVSPFIMILLFVTLVSTGGGKRFAEARNLLEVPDLPKPTLPEIPKPTLPVIPKPTL
PELPKPIIPEIPKPTLPEVPKPKVPELPKPTIPVIPKPILPEVPKPEVPVVPKPEVPELR
KPTIPEIPKPTLPEVPKPEVPLVQKPEVPELPKPTIPEFPKPTFPEIPKPEHPPLPKPQV
PKVPEVHDLPKPTIPELPKDFPIPSLPHP

>TA|EE655026||Asteraceae_H.exilis||PELPK
MANSHLNSFLMILVTVTLASIDGGNMVSEARNLLEVPEFPKPQVPEIPKATLPDIPKPKL
PEVPKPEVPVLPKPEIPELPKPIVPENPKPTFPEIPKPTFPEIPKPELPTLPKPEVPKVP
EFHDIPKPTLPTIPGLPKDLPLPHP

>TA|TA4140_400408||Asteraceae_H.exilis||PELPK
MAHSTLNSFLVILVTLTLASINGGKIVSEARNLLEVPQFQKPEVPEIPKPKLPEVPKPEI
PELPKPEIPELPKPKIPELPKPEVHVLPKPDIPELPKPTLPEIPKPELPTLPKPEVPKAP
EFHDLPKPKLPEVPKPEVPVLPKPEIPELLPKPTLPEIPKPIFPEIPKPEIPKPELPTLP
KPEVPKMPEFHDVPHP

>TA|EE635704||Asteraceae_H.exilis||PELPK
MACYKVSSFIMILLFITLVSTGGGKRFAEARNLLEVPDLPKPTLPEIPKPTLPVIPKPTL
PELPKPIIPEIPKPALPEVPKPEVPELPKPTIPEIPKPILPEVPKPEVPVVPKPKVPELP
KPTIPEIPKPTLPEVPKPEVPVVQKPEVPELPKPTIPEFPKPTFPEIPKPELPTLPKPEV
PKVPEVHDLPKPTIPELPKDFPIPSLPHP

>TA|EL468946||Asteraceae_H.tuberosus||PELPK
MAHSTLNSFLVILVTLTLASINGGKMVSEARNLLEVPQFEKPEVPEIPKPKLPEVPKPEV
PVLPKPDIPELPKPTLPELPKPTLPEIPKPELPTLPKPEVPKAPEFHDLPKPKLPEVPKP
EVPVLPKPEIPELPKPTLPEIPKPIFPEIPKPTIPEIPKPELPTLPKPEVPKVPEFHDVP
HP

>TA|EL462848||Asteraceae_H.tuberosus||PELPK
MACYKVSLFIMILLFVTSVSTGGGKRFAEARNLLEVPDLPKPTLPEIPKPTLPVIPKPTL
PQLPKPIIPEIPKPTLPEVPKPEVPVVPKPEVPELPKPTIPEIPKPTLPKVPKPEVPVVP
KPDLPELPKPTIPEIP*PTLPEVPKPKVPVVQKPEVPELPKPIIPEFPKPTFPEIPKPEL
PTLPKPEVPKVPEVHDLPKPTIPELPMDLPIPSLPHP

>TA|DW102733||Asteraceae_L.perennis||PELPK
MLTHKYPSFLIPLLIITLTSMRSSIIVAEARNLLEIHLPDLSNFPEIPKPELPDLPEFPK
PELPNLPEIPKPELPHLPSMEVPKVSDLPIPKFPDLPNDFPIPSERP

>TA|DW080571||Asteraceae_L.perennis||PELPK
MERYHLNSIILILVSVSLASISGNIMTKARKLLENPEVPMLPKLEVPVLPKPDLPVLPKP
ELPVLPKPELPVIPKPELPVVPKPELPVVPKPEVPVVPKPEIPVVPKPEFPVFPKLELPV
VPKPEVPVVPKPEVPVVPKPELPVVTKPELPEVPKPELPVVPKPELPVVPKPELPEFPN

>TA|TA1053_75948||Asteraceae_L.saligna||PELPK
MVPLKYPSFIIPLLLMTLTSMNSSIIVAEARNLLEIHLPDLPNLPEIPKPELPDLPEFPK
PELPNLPEIPKPELPHLPSFEVPEVSDLPFPKFPDLPNDFPIPSEIP

>TA|TA4832_75948||Asteraceae_L.saligna||PELPK
MAPSHHNSIILILVSLSLAFINGNIIAEARNLLEIPELPKPEVPVVPKPELPVAPKPELP
VVPKPELPVAPKLEFPVFPKPELPVVPKPELPVVPKPELPVAPKPELPVVPKPELPVIPK
PELPVAPKPELPIVPKPVLPVIPKAELPVAPKPELPVVPKPEVPIVPKPEIPVIPKPELP
IAPKPELPVVPKPEVPVVPKPELPVAPNPELPTEPKLELPVVPKPELPVVPKPELPVAPK
PELPVVPKAELPVVPKAELPVVSKPELPVVPKPELRWFQSRASCG

>TC|TC24832||Asteraceae_L.sativa||PELPK
MVPLKYPSFIIPLLLITFTSMNSSIIVAEARNLLEIHLPDLPNLPEIPKPELPDLPEFPK
PELPNLPEIPKPELPHLPSLEVPEVSDLPFPKFPDLPNDFPIPSEIP

>TC|DW144590||Asteraceae_L.sativa||PELPK
MGRSHQNFIILILFSVSLASFSGNIIAEARKLAEIPELPMVPKPELPVFPKPELPVFPKP
ELPVLPKPELPVIPKPEIPVLPKPELPKPELPVLPKPELPVVPKPELPVVLKPEFPVVPK
PEIPVLPKPELPVLPKPELPEVPKPDVPVVPKPAVVPEFPKPTFPVIPKPTLPELPKDFP
IPSVTHP

>TC|DW136235(revision)||Asteraceae_L.sativa||PELPK
MAPSHLNSILLILVSLS*AFINGNIIAEARNLLEIPELPKPEVPVDPKPEVPEVPVVPRP
EFPVAPKIVVSKPELLVAPKLEFPVVPKPELPVVPKPELPIAPKPEVPVVPKPELPLIPK
PELPIAPKPEVPIVPKPELPVIPKAELPVAPKPELPVVPKPELPVVPKPELPVVPKPELP
VAPKPEVPVVPKPELPMVPKPEVPEVPKPEPPLIPKPELPVAPKPELPIVPKPELPVIPK
LSFPLHQSPS

>TC|TC4994||Asteraceae_L.serriola||PELPK
MVPLKYPSFIIPLLLITFTSMNSSIIVAEARNLLEIHLPDLPNLPEIPKPELPDLPEFPK
PELPNLPEIPKPELPHLPSLEVPEVSDLPFPKFPDLPNDFPIPSEIP

>TC|BQ989458||Asteraceae_L.serriola||PELPK
MGRSHQNFIILILFSVSLASFSGNIIAEARKLKEIPELPVVPKPELPVFPKPELPVFPKP
ELPVLPKPELPVIPKPEIPVLPKPELPKPELPVLPKPELPVVPKPELPVVLKPEVPVVPK
PEIPVLPKPELPRLPKPELPEVPKPDVPVVPKPAVVPEFPKPTFPVIPKPTLPELPKDFP
IPSVTHP

>TA|TA545_75947||Asteraceae_L.virosa||PELPK
MGRSHLNSIILILVSVSLASFSGNIIAEARKLAEIPDLPVVPKPELPVFPKPELPVFPKP
ELPVLPKPELPVIPKPEIPVLPKPELPVLPKPELPVLPKPELPVVPKPELPVLLKPEVPV
VPKPEIPVLPKPELPVLPKPELPEVPKPDVPVVPKPEVPEFPKPTFPVIPKPTLPELPKD
FPIPSVTHP

>TA|DW160404||Asteraceae_L.virosa||PELPK
MTPYHLNSIVLILVSLSLAFISGNIIAEARNLLEVHVIPKPELPVAPKPELPVVPKPEVP
VVPKPELPVAPKPELPMVPKPEVPVVPKPEVPVVPKPELPVVPKPEVPVVPKPEVPVFPK
PELPVVPKPELPVAPKPELPPNPEVPVVPKPELPVVPKPELPVAPKPELPNPEVPVVPKP
ELPDVLKHELPVVTKPELPVPPKSELPVVPKAEVPVVPKPELHVVPKPELPVAPKPE

>TA|DR401593||Asteraceae_T.kok-saghyz||PELPK
MVRSILMILVLVTLASITGNIVAEARNLLEVPKPTLPDILKPTIPEIPKPIIPEFPKPTL
PEMPKPVIPEIPKPTLPDMPKPVIPEVPKPTLPEIPKPVIPEISNPTLPEIAKPTLPEMP
KPVIPDLPKPTLPEIPKPTLPEMPKPVIPEVSKPTLPEMPKPVIPEVPKPTLPDMPKPTL
PEFPKPTIPEMPKPVVPE

>LOC|AT5G09520||Brassicaceae_A.thaliana||PELPK
MTLKKSFSASLLSPFLIICLIALLSVPVSVGARRLLEEPKPEIPTFPELPKPEMPKLPEF
PKLELPKLPEIPKPEMPKLPEIQKPELPTFPELPKMPEFPKFDFPKLPELPKPEETKVPA
FTMPKFPGSP

>LOC|AT5G09530||Brassicaceae_A.thaliana||PELPK
MALMKKSLSAALLSSPLLIICLIALLADPFSVGARRLLEDPKPEIPKLPELPKFEVPKLP
EFPKPELPKLPEFPKPELPKIPEIPKPELPKVPEIPKPEETKLPDIPKLELPKFPEIPKP
ELPKMPEIPKPELPKVPEIQKPELPKMPEIPKPELPKFPEIPKPDLPKFPENSKPEVPKL
METEKPEAPKVPEIPKPELPKLPEVPKLEAPKVPEIQKPELPKMPELPKMPEIQKPELPK
LPEVPKLEAPKVPEIQKPELPKMPELPKMPEIQKPELPKMPEIQKPELPKVPEVPKPELP
TVPEVPKSEAPKFPEIPKPELPKIPEVPKPELPKVPEITKPAVPEIPKPELPTMPQLPKL
PEFPKVPGTP

>TC|TC93827||Brassicaceae_B.napus||PELPK
MALMKKSLFALLLSSPLLIICLTSLLTYPASVGARRLVEEIPKPEIPKLPELPHMEIPKL
PELPHPELPKFPELPKLPELPHPELPKFEVPKFPEHPKLEIPKLPEFPKPELPKIPEIPK
HEGTKLMETPKVPEIPKPELPKVPEIPKPELPKVPEIPKPELPKIPEIQKPEAPKLPELP
KVPEIQKPELPKVPEIPKPELPKMPEIPKPELPKMPEVPKLPELPKVPGTH

>TA|AM060470||Brassicaceae_B.oleracea||PELPK
MALMKKSLFALLLSSPLLIICLTALLTYPASVGARRLVEEIPKPEIPKLPELPHMEIPKF
PELPHPELPKFPEMPHAELPKFPELPHPELPKFEVPKLPELPKLEIPKLPEFPKPELPKI
PEIPKHEGTKLMETPKVPEIPKPELPKVPEIPKPELPKVPEIPKPELPKI

>TA|TA2845_4120||Convolvulaceae_I.batatas||PELPK
MAQYHLNPSFLLLFFITVSPAVVNMVQTEARNLLEITLPQLPFPEIPTILPKPEIPEIPK
PELPTLPKPEFPEIPHPELPSIPKLPEFPAIPKPELPAFPKPELPTKP

>TC|BJ566003||Convolvulaceae_I.nil||PELPK
MAAHHPNPSCFFLLFLITLSAVAIGTIQTEARHLLETTSPELPKPQIPTLPKPEVPEIPK
PKLPEIPKPELPQFPKPQIPAIPKPQVPEIPKPKMPEIPKPQVPEVPKPQVPEIPKPKLP
EISKPQVPDIPKPQVPEIPKPKMLEIPKPQVPEVPKPQVPETPKPKMPEISKPQVPDIPK
PQXREIPKPKMPEFPKPQVPEVPKPRXRKYQSQKWLESLNL

>TC|DV125222||Euphorbiaceae_E.esula||PELPK
MANLGISSNTVFPILLVICLSLTSCKIQVGARHLLEVPEIPKPELPKPELPKLPELPKPE
SPKIPELPKVPELPKLPELPKPELPKIQELPKPELPKAIELPKLPELPKLPELPKPELPK
IPELPKPELTHTPTFPKETLAP

>NR|gi:255563290||Euphorbiaceae_R.communis||PELPK
MACFCFRSSFFLLLLIALSIHVDARQLLETKLPEVPKPEFPKPELPKPELPTLPKTELPE
LPKPEVPKLPELQVPELPKPEVPKVPELPKLEIPKVPELPKFPELPKLEVPKVLKMPKPE
MPKVPELPKPELPPLPHFPELPKPTLPITPTVPKDIKPPQSTTTP

>NR|gi:255646404||Fabaceae_G.max||PELPK
MATCGLSTVIFSFVVLMLSSASSHTMVAGARNLLESTLPKPEVPQLPKPELPPLPKVPEL
PKAELPKIPTFPKPELPKVPELPKLEKPKVPELPKVSEIPKVPKLPKAPEMFKVSELPKL
ELPKVAEISKILELSKPELPKIPKLPKIHELSKVPELSNPELSKVSELPKVPELPKVPEL
SKPELPKVPELPKVRELPKPEFSKVPELPKVHELPKPEIPKILEFPKVPQFPKAFSTSNP

>NR|gi:255646086||Fabaceae_G.max||PELPK
MATYSWSTEIFSFLVLTLLSTSSHTMVAGARNLLESTLSKPEVPTLPKPHELPPLPEIPE
LPKFESPKIPALPKPELPKVPELSKPDMSKVPELPKVPERLKVPEISKILELSKPELPKG
PELLKPELPSVPNIPKVPELPKPELPEVPKLPKPELPKVPELPKPELPKVPEIPELPNLE
LPKVTQLPKSRLPKVPEIPKVPEFPKPELPKVPELSKPELPKAPEIPKVPEFPKPELPKV
PELPKPELPKIPEIPKVPEFPKPELPKVPEVPKPELSKVPKLPKSELPKVPEIPELPKSE
LPKIPEIPKVPEFPKVPKAFSTTNP

>TC|TC48013||Fabaceae_L.japonicus||PELPK
MASISLSTIIFPFFVLTLFSANSHMMVAGARILLESSEPHVPQLPKPELPLLQKPALPEV
PELPKPELPKPELPKVNVPELPKPELPKVPELAKPELPKINVPELPKPELPKVNVPELPK
PELPEFKVPELSKPELPKLNVPELPNPEMPKVPELPKPELPKIHVPELPKPEIPKVPELP
KPELPKVNAPEFPKSEMFKVPELPKPELPKINVPELPKHEMPKVPEISKAIPTPINP

>TA|CO514519||Fabaceae_M.sativa||PELPK
MAYINFSKIIFPLIILAILSARSITMVVGARNLLESNIPEVPKLDFPPIPKPELPKIPEL
PKPELPKVPELPKPELPKVPELPKPELPKFNVPELPKPEFPKVPELPKVPELPKFPELPK
PELPKVPELSMPEIPKIPELPKPELPKFNALELPKPEQPKVPELPKHELPKVS

>TC|TC128281||Fabaceae_M.truncatula||PELPK
MAYINLSKIIFSLMVLAILSTKSNTMVLGARNLLESNIPEVPKLDFPPIPKPELPTIPEL
PKPELPKVPELPKPELPKFNVPELPKLEFPKLPELPKVPELPKFPELPKPELPKVPELSM
PEIPKIPELPKPELPKLNAPELPKLEQPKVPELPKHELPKVSELPKPDIPKVPELPKPEL
PKVPELPKPELPKVPELPKPEIPKVPELPKLELPKVPELAKPELPKFNVPELPKPELPKV
PETPKGVPTNTP

>TC|TC136239(revision)||Fabaceae_M.truncatula||PELPK
MASINLSKFIFPFMLLALFSTNGNTRVAGARNLLESNIPQFPKLDFPPLPKPSLPELPKP
NFPNVPELPKPNVPELPKPDLPNVPEMPKLEIPKVPELPKPELPKFNVPELPKPELPKIT
ESPKLEQPKVPELPKPEIPKVPEMPKHELPKFNVPELSKPELPKIPESPKLEQPKVPELT
KPEIPKVPEMSKPELPKFNIPELPKPELPKAPELPKSEVPKVPELQKHEFPKAPELHEPR
LSKVSELPKPELPKIPELPKPQLPKTPELTKPEFPKVPELPKPELPRIPEMPKPNLPKVP
ELPKLEQPKVPELTKPEIPKVPELPKLELPKVPETPKVVFTTTP

>TC|TC13222||Fabaceae_P.vulgaris||PELPK
MATCSLSRVIFSFVFLVLLSASSHTMVLGARYLLESTLSKPEVPQLPKPELPKIPELLPK
PELPKVPELPKPELSKVPELPKLELPKVSELPKPELPKVPELPKPELPKVPELPKPELPK
VPELPKPELSKVPELPKPELPKVPELPKIPELPKPELTKVPEVPKPELTK

>TA|CK751569||Lauraceae_P.americana||PELPK
MASHTTFLLSFLVAFSLFGKDTTFAARHLLDIEAPKFEVPEFPHLPDLPKFEVPKLPQVP
DLLHLPEVPKIEVPQFPHLPEVPKIEVPQLPHLPEVPKEVPHLPEVPKVEEPEVPHLPEV
PKVEEPEVPHLPEFPKPTFPTIPTLPKFQGHP

>TC|TC133335||Malvaceae_G.hirsutum||PELPK
MASHRLPFFVLPFLFVTLSLMSSNTVLVGAWRLLETSVPEIPKPELPKIPSFPKVELPKP
ELPEIPKPEIPKMPELPKPELPKVPELPKPELPKVPEFPKVPELPKPELPKVPEFPKVPE
LPKPEFPKVPELKKPEEVKVPELPKVPEMPKAPKLSKSEAPKVPGLPKPELPKVPEVPKP
ELPKAPELPKVPEVPKPELPKAPELPKIPELTKPELPKIPEVPKPELPKVPELPKPEIPK
LPNLPKPALSRPLPD

>TC|TC1179||Malvaceae_G.raimondii||PELPK
MAYHPLPFFVLPFLLITLSSMSSNTVLVGARRLLETSVPEIPKPELPDMPSFPKVELPKP
ELPEIPKPELPKMPELPKPELPKVPELPRPELPKVPEFPKVPELPKSELPKVPEFPKVPE
LPKPEFPKIPELKKSEEVKVPEIPNAPKLSKSEAPKVPELPKPEFPKVPEVPKPELPKAP
KLPKPELPKVPELPKPELPKVPELPKPELPKPELPKAPELPKPESPKVPELSKSELPKVP
EVPKPELPKAPELPTIPEVPKPELPKA

>TC|TC10972||Malvaceae_T.cacao||PELPK
MASHYLSTLTLPLLLITFWSMSGDSAILVQARNLLEVTLPEIPEHPKPELPHLLPFPKVE
LPPLPEFPELPKPELPKLPELFKHFLPTAPTPAEDMSDPKLIPSHSTTTP

>TC|TC9012||Malvaceae_T.cacao||PELPK
MACYRFPFFILPFLLITLSLRSSNTILVGARLFLETSVPEKPELPKPELPEIPPFPKVEL
PKPELPDFPKPEIPKVPELPKPELPKTPELPKVPELPKPELPKVPELPKPELPKLPQLQK
PEEVKVPKLPKAPELPKVPELPKPESPKVPELPKAPELPKVPELPKPESPKVPELPKPEL
PKVPELPKPELLQVPELPKPELPK

>TC|TC3105||Orobanchaceae_T.pusilla||PELPK
MAKNHSYPFFLLFHIVLSSIACNTTPGKARHLLETASLPNFPKPEIPLPKFPSLPKPELP
TFPNPELPTLPKPEIPTLPKPEIPTLAKVELPSLPKSEIAELPKPKIPELPKPEIQQLPK
FELPTLPKPEIPQLTKSEVPEFPKLPELAKPTLPQVPKFP

>TC|TC9771(revision)||Orobanchaceae_T.pusilla||PELPK
MAKDHSFPSLFLLSLVTLSSIVCYTIPGEARHLPESALPDVPKPDIPLPKIPALPKPELP
TFPKPEIPTLPKLEIPTLPKFDIPTLPKPEIPGLPKSEMPTLAKAELPAQPKPVIPQLQK
PELPTLPKPELPQLPKAELPTIPKPELPKLEIPQLPKPELPKLPELPKLPELPKQTLPKS
P

>TC|TC4011(revision)||Orobanchaceae_T.versicolor||PELPK
MAKDHSFPSLFLLSLVTLSSIVCYTIPGEARHLLESALPDVPKPDIPLPKIPALPKPELP
TFPKPEIPTLPKLEIPTLPKFDIPTLPKPEIPGLPKSEMPTLAKAELPAQPKPVIPQLPK
PELPTLPKPELPHLPKAELPTIPKPELPKLEIPQVPKPELPKLPELPKLPELPKQTLPKS
P

>TA|AJ801862||Plantaginaceae_A.majus||PELPK
MAYNSHPACFYLLLLIALISITGNTFQVEARHLLESTLPELPKPEVPTLPKPELPTLPKP
EFPSLPKPELPKLPEKPQLPTLPKPELPVLPKPELPAVPKSELPTSVKAEVPTLPKPEIP
AVPKPEIPELPKSAIPAVPKPEIPTPPKPEIPAMPKPEIPAVPKPEIPAVPKPELPALSK
PELPKVPELPKTPELPKPALPQVPKYP

>TA|CN819511||Poaceae_A.sativa||PELPK
MASRNTIAVLLGLLVSCVAMSGAARILQEEAVPSKEEEHKPELPPLPKFELPPFPEVHLP
PKPELPKVELPPFPEVHLPPKPELPTFPEVHLPAKPELPKVEVPPKPEMPTIPELHFPEP
EAKP

>TA|DV483696||Poaceae_B.distachyon||PELPK
MDAKSRVSSVFFLVVLLLSCSCMTRAARYLEEKVPKEEVPKMPELPHPVVPEVPKKPEES
HPVVPELPKPELPHPVMPEVPKMPEVPHVSIPKVPKSEIPHLVVPEVPKAPEVPHPTVPE
VPKMPEVPHLSTPEAPKVSEVPHPTMSGVPKMPELSHPVMPEVPKVSEVPHPAVPEVPKM
PEVPHPAIPEMPKASELPHPEVPEAPKIREVPHPAVPELPKMPEMPHLTMPEVPKLPEVP
HVSIPEIPHPAIREVPKHELPPVPKVEVPPKP

>TA|DN481167||Poaceae_E.tef||PELPK
MFNMASKRAVSRLAFLVAVLLLCSSMSSAARLLEETKPKEEYPPHPTVPEIPKPELPPHP
TVPEHPKPELPPHSVVPELPKPELPPHPVVPELPKPELPHPVPELPKPELPHPVVPKPE

>TC|TC12364||Poaceae_F.arundinacea||PELPK
MVFRIATMPSLLALLVVLLLSRSGTGDAGRHLAEYPPASPLPKPEPVAPLPSAPEVLPKP
ELPGPLPTPEVLPKPELPPLPIPEVAPTLPGDPWLPLPDVEPKPELPPLPKVEEPPRPEL
PPLPIGEIPPVPVLPPLPIGELPPETEPELPPKPVLPPLPGGELPPKPEPELPPKTEEPP
KPVLPPLPAGELPPKPEPELPSKAEEPPKPVLPPLPTGEFPPKPEPELPPKTKEPPKPVL
PPLPTGELPPKLGPELPPKTEGHRSRCCRHYPQVSFHRNQSPSCHRSRCCHHFPQVSFHQ
SQTQSCHRRQRSRSSQCFRHSWQVSFHRSRSLNCHQRQRSQRSRCFRHSPRANFHRSPSP
SYHRSRYCRHSHQASFHQSQSLSCHRRKKSR

>TC|TC165281||Poaceae_H.vulgare||PELPK
MALKNSAVLLLGLLLSCVAMSSAARILEETVPSKEEHLPPKPELPKVELPPFPEVHLPPK
PELPKVELPPFPEMYLPPKPELPKMELPPVPEVHMPPKPELPKVELPTFPEVHIPSKPEM
PKVELPPKPEMPTVPGFHLPEPEAKP

>TC|TC173769||Poaceae_H.vulgare||PELPK
MLCKNTMASLVFIVALLLSCSSMSSAARHLEEVVPKKEYPPHPIIPELPKPEIPPHPAMP
ELPKPELPHPLVPEVPHPVVPETPKEPEVPHPVVPETPKEPEVSHPVVPEVPKHELPPHP
AMPELPKPELPHPAVPEVPHPIVPETPKEPEVPHPMVPEVPKHELPPHPAMPELPKPELP
HPAVPEVPKEHELPHPIAPEVPKEPEVPHPVVPEVPKEHELPHPAMPELPKPEMPHHVVP
EIQKEPPVSHPEVPKEPQVPHPAVPEVPKHEMPPFPISELPPKPEAKP

>LOC|LOC_Os07g40890.1||Poaceae_O.sativa||PELPK||Chr7, 24480482-24480874
MASKNSMSSSLLFLMALLLSWSSISSAARYLEEEAAPKEEYPELPKPELPPHLAVPELPK
PELPHGAAVPEFPKVPELPHPEVPELPKPELPEHPAVPELPKPELPSLPKVELPPLPKPE
FHFPEPEAKP

>LOC|LOC_Os07g40870.1||Poaceae_O.sativa||PELPK||Chr7, 24476646-24477047
MASSHTKPSILLLAAALLLLSCSSIGGAARYLEEAAPAAAAAAEEEEHPAHPAVPEIPKP
ELPELPKVPELHHPVVPELPKPELPKIPEVPHLAVPELPKPEVPEIPKAELPPLPKFELP
PKPEFHFPEPEAKP

>LOC|LOC_Os07g40830.1||Poaceae_O.sativa||PELPK||Chr7, 24465856-24466314
MSSSSSSSSALLLMAALLLSCGAMGSTARHLEEKAPHFPAVPELPPHPELPELPKPELPP
PLPELPRPVVPELPPHPAVPELPPLPKPELPPHPVVPEMLPHPVVPELPHYPAVPGFPKH
GLPPKPELPPLPTAELPPEHEVHDPEPETKQP

>TC|CR281225||Poaceae_O.sativa||PELPK
MSSSVLLFLVALLLSCSTMGNATRRLEVEYPAAHPAVPELPKPELPAHPVVPELPKHEEP
PGTSTARLWRSFRSLNWRRRTPAVPELAKHEEPPHPVVPELAKPEVPHPVVPEFAETPKS
QRSPSCRSWRSPRCLNLLCRSCRNTKSTTTRCAGSAKAEVSHLWCRVARNPVVAKPPGTT
LTTAHTKNLLGKT

>LOC|LOC_Os07g40850.1||Poaceae_O.sativa||PELPK||Chr7, 24469421-24470047
MSSSVLLFLVALLLSCSTMGNAARRLEVEYPAAHPAVPELPKPELPPHPVVPELPKHEEP
PPHPHPAVPELPKPELPPPHPAVPELPKHEEPPHPVVPELPKPEVPHPAVPELPKHEEPP
HPVVPELPKHEEPPHPVVPELPKPEVPHPAVPELPKHEEPPHPVVPELPKPEVPHPVVPE
LPKPEVPHPTVPEHEQPPKPESHYPEKP

>LOC|LOC_Os07g40860.1||Poaceae_O.sativa||PELPK||Chr7, 24471743-24472534
MVFKKNAMSSSVLFLAALLLLSSSSMSSAARWLEEEYPPHPTVPELPKPEVPPHPAVPEL
PKHEEPPHPVVPELPKHEEPPHPVVPELPKPELPPHPVVPELPKHEEPPHPAVVPELPKH
EEPPHPAVVPEFPKHEEPPHPAVPELPHPAVPEIPHPAVPELPKHEEPPHPVVPELPKPE
VPHAAVPELPKPELPPHPAVPELPKHEEPPHPVVPELPKHEEPPHPVVPELPKPEEPHHP
EVPEHEQPPKPESHYPEVPMAKP

>TC|TC18443||Poaceae_P.virgatum||PELPK
MASRTAAVPSLALLLALLLSCAVTMSSAARRLEEEAAPKEEEPEFPPHLSVPELPVPEHE
LPPLPKVELPPLPVAHLPPKPELPPLPKVELPPKPELPAIPEFHFPEPEAAKP

>TC|TC35492||Poaceae_P.virgatum||PELPK
MVSKKTMSSLAFLVALLLACSSMSSAARYLEETKPEYPPHPAVPEIPKPEYPPHPTVPEI
PKPELPPHPTVPEIPKPELPPHPTEPEHPKPELPPHPTLPELPKPELPPHPTVPEHPVPE
VPKPELPQPAVPEVPHPVPEVPKPELPHPAVPEVPKPELPPHPEVPELPKPEAPHVPEAP
KHELPPHPEVPELPKPEAPHVPEAPKHELPPLPEPELPKPESHYPVPEAKP

>TC|CA136904||Poaceae_S.officinarum||PELPK
MFQPNMASRTGVPVLVLLALLLSCAVMRSTARRLEEAAPKQEEEFPPHLPVPELPLPVPE
HELPPFQDVHLPPKPELPPFPEVELPPKPELPPKPEMPSIPEFHFPEPEAKP

>TC|TC84382(revision)||Poaceae_S.officinarum||PELPK
MAAKSTMVTSLVLLMALLLSCSGISAAARLLEEAAPKEEYPQPATPELPKPELPPHPTVP
ELPKPEVPAHPELPPHPTELPKPEVPAEHLPELPKPELPPPHPAAVHEPELPKPEVPEHP
AVPELPKPELPPHPAVPELPKPEVPAHPTVPELPTHPTYPEVPEVPKHELPPKPESHYPV
PETKP

>TC|TC91904||Poaceae_S.officinarum||PELPK
MVFKRTMSPSLAFLMALLLSCSTMSSAARYLEETRPAEEYPPHPTAPEIPKPELPPHPTV
PELPKPELPPHPTLPELPKPELPPHPTVPEHPKPELPPHPTVPEHPKPELPPHPTVPEAP
KPELPHPAVPELPHPAVPEVPKPELPHPAVPEVPKPEMPHPAAPVGPKPELPHPAVPEVP
KPELPHPDVPKPELLXHATAPEVPEVPKHE

>TC|CA132584||Poaceae_S.officinarum||PELPK
MASKTTMRSLVLLMGLLLLSCSGKSSAARLLEEVAPKEEYPHPEVPELPKPELPPHPTMP
ELPKPELPPHPVVPELPKPEVLVYPAVPEVPKPELPPHPPVPELPKPEVPEHPAASVPEV
PKPELPPHPTVPELPKPEVPEHPAAAVPELPKPELPPHPTVPELSKPEVPEHPAAVPELP
KPEVPEHPAVRELPKHELPPLPEPELAVTPKGRE

>TC|TC78131||Poaceae_S.officinarum||PELPK
MGSSNTSTASALLLVALLLCCSGMSSAARLLEEAPPKEEHPHPAVPELPKPELPPHPTDV
VPPELPKPELPPHPTVPELPKPEVPHPVPEQPKPELPPHPAVPELPKPEVPQTVPEQPKA
ELPPHPAVPELPKPEVPHPVPELPKPEVTPHPAVPELPKPEVPHPVAPELPKPELPSHPT
VPEVPEVPNHELPPQPKAELPTKPGGPLPGAGSSNNHERTYTVPPGYLHILPHVHGG

>TC|BE586616||Poaceae_S.cereale||PELPK
MASRNTVVFLLGLFLSSVAMSSAARMLDEEMAPSKGEDHQPELPTLPKVELPPFPEVHLP
PKPELPKVELPTFPEVHLPPKPELPTFPEVHLPAKPELPKVELPPKPEMPTIPEFHFPEP
EAKP

>TC|BE438513||Poaceae_S.cereale||PELPK
MLCKNTMVSLVFLVALLLSCSSMSSAARHLAEAVPKKEYPPHPIIPELPKPELPPHPAMP
ELPKPELPHPLVPEVPPPVVPETPNESEVPHAVVPEVPKHELPPHPAMPELPKPELPHPA
VPETPKEPKVPHPMVPEMPKHELPPHPTMSEIPKPELPHPAMH

>LOC|Sb02g038460.1||Poaceae_S.bicolor||PELPK||Chr2, 72717137-72718030
MAAKSTMIPSLVLLIALLLSCSGISGAARLLEEAEYPHPATPAELPKPELPPHPTVPELP
KPEVPAHPATPELPKPEIPEHLPELPKPELPPPHPAAVPELPKPEVVPEHPAVPELPKPE
LPPHPAVPELPKPEVPAAHPTVPELPKPELPPHPTVPELPKPEVPKHELPPKPESHYPVP
ETKP

>LOC|Sb02g038480.1||Poaceae_S.bicolor||PELPK||Chr2, 72723269-72723829
MSSLALVLALLLSSGAIGSAARQLQELPPLPKPEIPPRPDLPPLPKPEEQPPLPKPELPV
PPQPLPKPELPVPPQPLPVPPQPLPKPELPVPPEPLPKPELPVPPQPLPKPEVPVTPEPL
PKPELPVPPQPLPKPELPVPPEPLPKPELPVPPEPLPKPELPVPPQPLPKPELPPPVLAG
ELPPKP

>LOC|Sb02g038440.1||Poaceae_S.bicolor||PELPK||Chr2, 72711620-72712523
MASSSRSTVSSFLLVALLLCCSRMSSAARLLEEAPPKEEYPHPAVPELPKPELPPHPTDV
VPPEVPHPVPEQPKPELPPHPAVPELPKPEVPHTVPEQPKAELPPHPAVPELPKPEVPHP
VPELPTPELLPPHPAVPELPKPEVPPHPVAPELPKPELPPHPTVPELPHPDVPEVPNHEL
PPLPKAELPPKPEGHYPEPEAKP

>LOC|Sb02g038450.1||Poaceae_S.bicolor||PELPK||Chr2, 72714216-72714892
MASKSTMHSLVLLMGLLLLSCSGTSSAARLLEEVAAPKEEYPHPEVPELPKPELPPHPTV
PEFPKPELPPHPVLPELPKPEVPVHPAVPEKLPKPELPKPEVPEHPAAVPELPKPELPPH
PTPTVPELPKPEVPEHPAAVPELPKPELPPHPTVPELPKPEVPEHPAAVPELPKPEMPEH
PTVPELPPLAEPELPVPPKAESHYPEPETKP

>LOC|Sb02g038470.1(revision)||Poaceae_S.bicolor||PELPK||Chr2, 72719604-72720631
MSSSLAFLMALLLSCSTMSSAARYLEETKPAEEYPPHPTVPEIPKPELPPHPTVPELPKP
ELPPHPTVPELPKAELPPHPAVPEHPKPEPPPHPTVPEHHPTVPELPKPELPHPTVPEVP
KPELPHPAVPELPHSAVPEVPKPELPHPAVPEVPKPELPPHPAAPEVPKPEMPHPAVPEV
QKPELPHPEVPKPELPPHPTVPEVPEVPKHELPPKPESHYPVPEAKP

>TA|EH277081||Poaceae_S.alterniflora||PELPK
MATMSSLVFVLALLISCGSMNDAARRLDEVPKLDVPPMPKPEEQPRPEMPPLPEPEVQPK
PELPPLPKPEEQPKPEMSPLPKPEEQPKPEMPPLPKPEEEPKPEMPLLPKPDDQPKTEMP
PLLKPEEPPKPEMPPLPKPDEQPKPEMPSLPKPEEQPKPEMPPLSKPEQQPKPELPQLPK
PDMPRPEPAEPKP

>TA|EH277583||Poaceae_S.alterniflora||PELPK
MASKRAMSFGIAFLVALLFCSSMGSAARYLEETKPKEEYPPHPVMPEIPKSELPPHPVVP
ELPKPELPPHPVVPEHPKPEMPPHPEVPELPKPEVPKKPELPHPAVPELPHHPVMPEMPK
PELPHPVPEAPKPELPHPELPHPAVPEVPKTPELMPHPELPKPEVPEHPVVPELPHVPEL
PKPEMPHVPEVPKHELPPQPEH

>TC|TC312590||Poaceae_T.aestivum||PELPK
MASRNTAAFLVGLLLSCVPMSSAARILEEETAPSKGEEHLPELPMLPKVELPPFPEVHLP
PKPELPKVELRSFPEVHLPSKPELPTFPEVHLPAKPELPKVELPPKPEMPTIPEFHFPEP
EAKP

>TC|CA627346||Poaceae_T.aestivum||PELPK
MASSSRSTVFSLLLLVALLLSCSGMSSAARLLEEAPPKEEHPHPAVPELPEPELPPHPTD
VVPPELPKPELPPHPAVVPELPKPEVPHPVPEQPKPELTPHPAAVPELPKPEVPHPTASL
SSRNRXAAPXPELPSPKCNAPTAS

>TC|TC296350||Poaceae_T.aestivum||PELPK
MALKNSAVLLIGLLLSCVATSSAARILEETVPSKEEHQPEVPPLPKVELPPFPEVHLPPK
PELPKVELPPVPEVHLPPKPKLPKVELPPVPEVHMPPKPELPKVELPSVPEVHLPPKPEL
PKVELPTFPEVHMPPKPEMPKVELPPKPEMPTVPVFHVPESEPKP

>TC|TC346707||Poaceae_T.aestivum||PELPK
MLCKNTFLVFLVALLLSCSSMSSAARHLEEAVPKKENPPHPIVTELPKPQLPLHPAMPEL
PKPEPPHPLVPEVPHPVVPETPKEPEVPHPVVPEMPKHELPPHPAMPELPKPELPHPAVP
EVPKDTEAPHPIVPKEPEVTHPKVPEVPKHELPPHPAMPEIPKPELPHPVVPEVPKETQG
PHPIVPEVPKELDLPRPVMPEVSHPVVSEVPKEPELPHSAMPELPKPEVPHHGVPEVPSE
PHVPHPEVPKEPELPHPAVPEVPKHEMPLPPKAELPPKPEFHFPEPKTKP

>TA|BE428778||Poaceae_T.turgidum||PELPK
MLCKNTMASLVFLVALLLSCSSMSSAARHLEEAVPKKQYPPHPIVPELPKPELSPHPAVP
ELPKPEPPHPLVPEVPHPMVPETPKEPEVSHPMVPEVPKHELPPHPAMPELLKPELPHPA
V

>TA|AJ610900||Poaceae_T.turgidum||PELPK
MASRNTAAFLVGLLLSCVPMSSAARILEEETAPSKGEEHLPELPMLPKVELPPFPEVHLP
PKPELPKVELRSFPEVHLPSKPELPTFPEVHLPAKPELPKVELPPKPEMPTIPEFHFAEP
EAKP

>LOC|GRMZM2G102138||Poaceae_Z.mays||PELPK||Chr2, 206601194-206602014
MPFDMVSRRTMPSSSLAFLVALLLLSCSTTMSSAARYLLEETKPAPHPTTVPEIPNNKPE
LPPHPTVPEVPKPELPPHPTTVPELLEPELPPHPTVPELPKPELPPPHPTVPELPLPKPE
LPPHPTTVPELPKPELPQHPAVPELPKPELPPHAPAVTEEPAVPKHEFPPLPMPELPPKP
ESHYPAVPDAKP

>LOC|GRMZM2G152189||Poaceae_Z.mays||PELPK||Chr2, 206594946-206596126
MASKSTMRSLVLIMGLLLLSCSGMSSAARLLEEMAPKEEYPHPEVPELPKPELPPHPTVP
ELPKPDELPPHPVVPELPKPEVPVHPAVPELPKPEIPPHSTVPELPKPEMPELPKPEVPE
HPAVPELPKPELPPHPTVPELPKPEVPELPKPEVPEHPAVPELPKHELPPHPTVPELPKP
EVPELPKPEVPEHPAVPELPKPELPPHPTVPELPKPEVPAHTAVPEPEPELPKHELPPLP
EPELPLPPKAESHYPVPGMAKP

>LOC|GRMZM2G019373||Poaceae_Z.mays||PELPK||Chr7, 157894704-157895905
MASSSRSTVFSLLLLVALLLSCSGMSSAARLLEEAPPKEEHPHPAVPELPEPELPPHPTD
VVPPELPKPELPPHPAVVPELPKPEVPHPVPEQPKPELTPHPAAVPELPKPEVPHPTAVP
ELPKPEVPHPVPELPKPEVPPHPTAVPELPKPEVPYPVPELPKPELTPHPAAVPELPKSE
VPHPVPELPKPEVPPQPTAVPELPKPEVPHPVPELPKPELPPHPAVPELPKPEVPHQVAP
ELPKPELPPHPTVPKLPHPEVPEVPNHELPPLPKAELPPKPEGHYPEPEAKP

>NR|gi:226501316||Poaceae_Z.mays||PELPK
MSSLALALALLLLSGDIGGAARQLQELPKPDLPPLPKPEVPPRPDLPPLPKPEEQPPLPK
PELPVPPQPLPIPPQPLPKPELPVPPEPLPVPPQPLPVPPQPLPKPEAELPVPPEPLPVP
PQPLPVPPQPLPKPEAELPVPPEPLPVPPQPLPVPPQPLPNPPELPVPPEPLPVPPQPLP
KPDDMPVPPEPLPKPELPVPPQPMPKPELPVPPQPLPKPDMPVPPESLPKPELPVPPEPL
PKPELPVPPEPLPKPELPVPPEPLPKPELPPPVLPGELPPKPELVPLPPKAEIMPPLPKP

>TC|TC33010||Rosaceae_M.domestica||PELPK
MVVDHGFRPAFVLPLVLVFTFSPMSSKTMIAGARCLLETSLPHVPELPKPELPPLPNFPT
LPKPELPPLPKPELPKFPNPELPHELPKPELPQLPKPELPKFSKPELPHELPKPELPPLP
KSELPKFPKPELPPLSKAENVPKLEGLKLAEVPHVPTLPAVPQLPKPELPPLPEFPTFPK
PELPPLPKPELPKLPAIPTLPHLPTELPKPTFPSIPSLTPPHKTTLP

>TA|CX301353||Rutaceae_C.aurantium||PELPK
MAYQSFPSFFLTLLLVTLSLMWYNINIVDARHLLEVALPEIPKPELPKVELPPFPKPELP
KVELPPLSKPELPKVELPPLTKPELPEIPKPELPKVPELPEFPKPALPKKLEVPEIPKPE
LPELPEFPEIPKPELPELPELPSFPHLPEFPKPTHSTSNP

>TC|BQ623883||Rutaceae_C.sinensis||PELPK
MAYQSFPSFFLTLLLVTLSSMWYNINIVDARHLLEVALPEIPKPELPKVELSPFPKPELP
KVELPPLSKPELPKVELPPLTKPELPEIPKPELPKVPELPEFPKPALPKKLEVPEIPKPE
LPELPEFPEIPKPELPELPELPSFPHLPEFPKPTHSTSNP

>TC|CN183056||Rutaceae_C.sinensis||PELPK
MASFSFSSVMLPLLLIALSSMSFNIDLIAARHLLEATVPEIPKPKLPKVELPPLPKPEIP
QIPKPELPEIPKPELPKKPQLPEIPKPELPKKPELPEIPKPELPKEPELPEISKAEVPKE
PEVPEHPKPELPKVPEVSKELEVPEIPKPELPKVPEVPKEPEVPEHPKPELPKVPEVPKE
PEVPELPKPELPKVPKVPKEPEVPEHPKPELPKVP

>TA|TA3054_309804||Rutaceae_C.x||PELPK
MAYKSFPSFFLTLLLITFSLMCNNINAVGARLLLETALPEIPKPELPKFELPPLSKPELP
KVELPPLSKPELPEIPKPELPKVPELPEIPESKLPKKLEVPEIPQPELPEFPEPELPELP
EFPEIPKPELPNLPELPFFPHLPEIAKPTHSTSNP

>TA|TA2776_3696||Salicaceae_P.deltoides||PELPK
MASLPFLTFLSPLLLITLSMVDNTSRTEARRILETTLPKVPELPKPELPELPPLPKVELP
TLPKPELPVLPKPEFPELPKPEVPKLPELPSFPHFPELPKTTLPTIPALPKDIKPPQSTT
SP

>TA|TA2259_3696||Salicaceae_P.deltoides||PELPK
MANHIFPLFISTLIVVMSMSLIISQTILVEARQLLEVTLPELPKPEFPELPKPELPKLPE
FPIPELPKFEIPKLPELPKPEFPELPKPELPKLPEFPIPELPKFEKPKLPELPSFPHFPD
LTKPTLPTIPSHSTTSP

>TA|AJ774925||Salicaceae_P.euphratica||PELPK
MANHNFPLFISPLIVIMSMSLIISQTILVEARQLLEVTLPELPKPEFPELPKPELPKLPE
FPMPELPKFEIPKLPELLKPEFPELPKPELPKLPEFPKPELPKFEIPKLPELPSFPHFPD
LTKPTVPTIPSHSTTSP

>NR|gi:224140717||Salicaceae_P.trichocarpa||PELPK
MANHNFPLFILRFFPLFVISSLSLMNSQTILVEARQLLEVTLPELPKPELPKLPPLPEFP
KPELPELPEFEIPKLPELPPFLHFPELPKPALPTIPRGINPSHSTTSP

>NR|gi:224140719||Salicaceae_P.trichocarpa||PELPK
MAYRHFPSSILPLMVISMSLMNSQTILVEARQLLEAPLPELPKPELPKPELPELPKPEFP
ELPPKPELPKFEVPQLPELPTFPHLPELPKPTLPTIPKDINPSHSTASP

>TC|TC106101||Salicaceae_P.trichocarpa||PELPK
MASLPFLTFLSPLLLITLSMVDNTSRTEARRILETTLPKVPELPKPELPELPPLPKVELP
TLPKPELPVLPKPEFPELPKPEVPKLPELPSFPHFPELPKTTLPTIPALPKDIKPPQSTT
SP

>TC|TC93681||Salicaceae_P.trichocarpa||PELPK
MANHIFPLFISPLIVIMSMSLIISQTILVEARQLLEVTLPELPKPEFPELPKPELPKLPE
FPIPELPKFEIPKLPELPKPEFPELPKPELPKLPEFPKPELPKFEIPKLPELPPFPHFPD
LTKPTLPTIPSHSTTSP

>TA|BP750003||Solanaceae_N.sylvestris||PELPK
MAYRHNQSFFLVLLVALSLTSCNTIQAEARHLLEVTLPELPKPELPEIPTLPKLEFPEIP
KPELPTLPQPELPEFPKPELPTLPMPELPPMPKLEFPTIPKPELPTLSKPAMAQKP

>TC|TC53204||Solanaceae_N.tabacum||PELPK
MAYHHNPSFSLLLLVTLSLTSSYVIQAEARHLLEVTMPELPKPELPHLPEIPTLPKPEFP
EIPKPELPTLPKAELPEIPKPEFPTLPKPELPTMPKLEIPAIPKPELPTLPKPEIPQVPK
KP

>TC|CV300828||Solanaceae_P.hybrida||PELPK
MAQHYQFSSVLLLSFLSLCFVHSNITGATARRLLETPFPEIPKPEFPKVPALPKPEIPTV
PKPELPAVPKPEVPTLPKPEVPAVPKPELPAVQKPELPTLPKPEIPAVPKPELPAVPKPE
LPTEPKPEIPNYAXSLSYXTFPXT

>TC|TC205219||Solanaceae_S.lycopersicum||PELPK
MAYKYIPSFFLLLFVTLFLTSSYVIQVEARNLLEVTIPELPKPELPHLPEIPTLPKLEFP
EIPKPELPTLPKPEFPEIPKPEFPSFSKVELPTLPKLEIPVIPKLELPTLSKPKMP

>TC|TC209972||Solanaceae_S.lycopersicum||PELPK
MAHYYNPSFFFLLFVTSFLTSDYVIRSDARHLLEITLPKLPKPELPHLPEIPTLPKPEFP
EIPKPELPTLPKPELPKIPKPEFPTLPKPELPALPKLEIPVIPKPELPIFPKLDIPQVPK
KP

>TC|TC214240||Solanaceae_S.lycopersicum||PELPK
MAQHYHVSSLLLLAFLNIFFIHGNISGATARHLLETPVPEIPKPQLPKVPAIPTVPKPEL
PAIPKPELPTLSKPQLPTLPKPKMPEIPTMPKSELPSMPKLEIPPLKKSEIPAVPKTEVP
PVMKKPEVPTLPKPELPSLPKPEIPELPKPKVPELPKLKIPTMPKPEVPTMPKHEIPKPK
VQELPKPEIPTTPKPEIPELPKTKVPELPKPEVPTMPKPEIPELP

>TC|TC173370||Solanaceae_S.tuberosum||PELPK
MAYKYTPSFFLLLFVTLFLTSSYVIQAQARNLLEVTIPELPKQELPHLPEIPTLPKLEFP
EIPKPELPTLPKPELTHLPEIPTLPKLEFPEISKPELPTLPKPEFPEIPKPEFPPFPKAE
LPTLPKLEIPVIPKPELPTLSKPKIPQVPKKP

>TA|TA1774_189793||Tamaricaceae_T.hispida||PELPK
MAISYRSSILVTMLSAVAFCMMSTNTIPVSARHMLETAQPELPKAELPPLPKVELPPLPK
VELPAVPQLPEPKLPEIPKVEIPKLPELPRLPEVPKLPEIPKLEVPKLPELPKVTFPAIP
SKP

>NR|gi:147807305||Vitaceae_V.vinifera||PELPK
MARHHEPSILLPLLLITLSLMSGKEVLASRHLLETTLPTVPELPKVELPPLPTLPTLPKF
ELPPLPKVELPPLPHVPTLQEPQLPTLPKPELPTVPHVPALEKPELPKPTLPTIPTLPKD
IPFPSLSPPHSTTSP

>NR|gi:147776523||Vitaceae_V.vinifera||PELPK
MAHHHDQSILLPLLLITLSLMSCKEVFASRHLLEETTLPKVPELPKPELPPLPTLPTFPK
PELPPLPHIXTLPESTLPTLPKPELPTVPHVPALEKPELSPLPKLEVPKLPEVPPLPHLP
DLPKPTLPTVPTLPKDIPFPSLSPPHSITSP

**@ PEPKA Class [22 Proteins, 15 Species]**

>TA|TA1034_4498(revision)||Poaceae_A.sativa||PEPKA
MARHRLLALLLIGVVAASAFHQAAAAGRGLAAVDKLPEPEPKPEPKPEPMPQPEPKPEPK
PEPMPKPLPKPEPKPEPKPEPMPKPEPKPEPLPKPEPKPEPKPEPMPKPEPKPEPMPKPE
PMPKPEPKPEPKPEPMPKPEPKPEPKPEPMPKPEPKPEPKPEPMPKPEPKPEPKPEPMPK
PEPKPEPMPKPEPKPEPMPKPEPKPEPKPEPMPKPEPKPEPKPEPMPKPEPKPEPKPEPM
PKPEPKPEPMPKPEPKPEPKPEPKPKPEPKPEPKPQPKPKPMPKPEPKPDPKPKPEPMPE
PKPKPEPKPEPKPEPLPKPEPKPEPEPKPEPPPKGKPPSTDI

>TC|TC6211||Poaceae_F.arundinacea||PEPKA
MARHRLLAVLLVGVVAASAFLQAAAAGRGLAAVEKLPEPEPKPVPYPEPKPEPKPEPMPK
PMPQPEPKPKPEPKPEPKPEPKPEPKPEPMPKPEPKPEPLPKPEPKPEPKPEPKPEPLPK
PEPKPMPKPEPLPKLEPKPEPKPEPKPEPKPKPEPKPEPLPKPEPKPEPKPEPKPEPMPK
PEPKPEPKPMPEPKPEPKPMPEPKPEPKPEPKPKPEPKPEPKPEPMPKPEPKPEPKPEPK
PEPKPKPEPKPEPMPEPMPKPEPMPKPEPKPEPKPEPMPKPEPKPEPKPEPEPKPEPPPK
GKPPSTDI

>TC|TC166910||Poaceae_H.vulgare||PEPKA
MARHRLLAVLLIGVVAASTLNQVVAAGRGLAAVENFAELEPKPEPKAEPMSKPAPQPEPM
SKPAPQPEPEPCKPEPMPKPKPKPKQKPGEKPEPEPKPKPMPKPKPEPCSKPKPGPKPEP
MPKPEPKPEPKPKPEPKPKPPPKCKPPATHN

>TC|TC157836||Poaceae_H.vulgare||PEPKA
MARHSLHAVLMLLVGVVAASTFHQSAAAGRGLAAVEKFADLEPKPKPQQMPKPIPKPEPK
PHPKSDPKPMPKPMPKPKPKPEPKPKPILKPEPKPMPKPEPKPKPMPKPEPKPEPKPKPM
PKPEPKPEPKPMPKPEPKPQPMPKPEPKPEPPPKHKPPTAYN

>TC|TC165053||Poaceae_H.vulgare||PEPKA
MARHSLLAVLLVGLLAASGFSLAAGAGRGLAEKLPEPEPKPTPYPEPKPMPKPEPMPKPE
PTPMPKPEPMPKPEPKPMPKPEPMPKPEPKPEPKPEPMPKPEPKPEPMPKPEPKPEPKPE
PMPKPEPKPEPMPKPEPKPEPKPEPMKPEPKPMPKPEPKPEPKPEPMPKPEPKPEPKPEP
MPKPEPKPEPKPEPMPKPEPKPEPKPEPMPKPEPKPEPKPYPMPKPEPKPEPKPEPMPKP
EPKPEPKPEPMPKPEPKPEPKPEPMKPEPKPMPKPEPKPEPKPEPMPKPEPKPEPMPKPE
PKPEPMPKPEPKPYPMPKPEPKPQPMPKPEPKPEPEPKPEPPPKGKPPMTDN

>TA|TA902_4521||Poaceae_L.multiflorum||PEPKA
MARHRLLALLLVGVVAASAFHQAGAAGRGLADVDKLPEPEPKPVPYPEPKPEPKPEPTPK
PMPQPEPKPKPEPKPEPKPEPKPEPMPKPEPKPEPKPEPLPKPEPKPEPKPEPKPEPLPK
PEPKPMPKPEPKPEPLPKPEPKPEPKPEPKPEPKPKPEPKPEPKPEPKPDPKPE

>NR|gi:50511379||Poaceae_O.sativa||PEPKA
MARRSPCLTAAVLLLGALAVASALVDEAAAAGQGLGHGARFMSKQGRAMYEKPPELEPKP
KPKPKPHPKHESKPEPKPEPKPEPKPYPEPKPETKPELKPEPKPNPEPKPEPKPEPKPEP
KPYPEPKPKPKPEPKPEPKPEHKPEPKPEPEPKPYPKPKPEPKPGPKPEPKPEPKPHPEP
KPEPKPKPVPHPEPKPEPKPEPKPHPEPKPEPKPEPKLHPKPEPKPHPEPEPKLKPEPKP
EPKPEPEPKPEPKPEPKPEPKPYPKPKPEPKPVPKPKPIPHPGPKPKPKPDPKLEPKPHP
EPKPHPMPEPEPKPKPEPKPEPKPYPEPKPKLKPEPKPGPKPIAPPNKHKPPHMPPATNQ

>NR|gi:125551325||Poaceae_O.sativa||PEPKA
MARRSPCLAVAMLLLGALAVASAFIDEAAAAGRGLGHGARFMSKQGRVTYEKPPEPEPKP
KPKPHPKPTPKPKPKPEPEPKPVPKPEPKPEPKPEPKPEPKPEPKPYPEPKPEPKPEPKP
EPKPEPKPEPEPKPEPKPEPKPEPKPYPEPKPEPKPEPKPEPKPEPKPKPEPKPHPEPKP
DPKPEPKPHPEPEPKPEPKPEPKPHPEPEPKPEPKPEPKPEPKPEPKPEPKPEPKPEPKP
KPEPKPYPEPKPKPEPKPEPKPEPKPEPKPEPKPEPKPEPEPKPKPEPKPHPEPEPKPEP
KPEPKPEPKPEPKPEPKPEPEPKPEPKPEPKPEPKPYPEPKPDPKPEPKPHPEPKPEPKP
QPEPKPEPKPEPKPEPKPEPKPEPKPYPEPKPEPKPEPKPEPKPEAPPKKHKPPHIPPAT
DQ

>TC|TC1782||Poaceae_P.virgatum||PEPKA
MRMRCTLVVWVSLVAMAAAAGGGDDKPEPWPVITPKPKPQPQATQPKPTPQPYQPKPQPE
PGHPKPTPEPNPTPEPTKPKPMPQPEPKPTPQPYQPKPQPEPGPTPEPTKPKPMPQPEPK
PKPRPQPKPEPQPGPGKPKPKPPAYSPGTPGP

>TC|TC17540||Poaceae_P.virgatum||PEPKA
MATRRLSFPCCLLLAVLLLGAVAAATAFFDEVAAAAGVGLGHGARFARKPGRAAAEKPLP
EPQPLPHPKPEPKPEPKPEPLPHPEPKPEPKPEPEPKPLPHPEPKPEPHPKPVPKPEPKP
EPKPEPKPEPKPEPKPLPEPKPEPKPEPKPEPKPKPEPKPEPKPEPKPMPEPKPEPKPEP
KPDPKPEPKPKPEPKPEPKPKPKPKPEPKPEPKPKPEPKPEPKPEPEPKPEPKPEPKPEP
KPEPKPKPEPKPEPKPEPKPDPKPEPKPEPKPEPKPEPKPKPEPKPEPKPKPEPMPEPKP
KPDPPHIPPAADN

>NR|gi:18873729||Poaceae_S.hybrid||PEPKA
MAAPRRLSSCCLLFAVLLGAVLATATAFFDEAAAAGVGLGHGARFARKHGRAAAELPQPE
PQPKPEPKPEPQPQPEPLPKPEPQPKPTPKPEPKPKPEPVPKPKPEPKPEPEPKPHPKPE
PKPEPEPKPEPKPEPKPEPKPEPKPEPLPKPEPQPEPKPEPKPEPVPKPEPKPEPKPEPK
PEPKPEPKPEPLPKPEPEPKPEPKPEPLPKPEPKPEPKPEPKPEPLPKPE

>TC|TC111539||Poaceae_S.officinarum||PEPKA
MATKHLALAILVLLSIGMATAGGSRKLGYAPGGGGGGGGSGGGGGGEPKPEPKPKPKPEP
KPEPLPKPEPKPEPKPEPLPKPEPKPEPKPEPKPEPLPKPEPKPEPLPKPEPKPEPKPEP
LPKPEPKPGPKPKPLPKPEPKPKPKPEPLPKPEPKPKPEPKPEPKPEPEPEPEPKPEPKP
EPKPEPEPEPKPEPEPKPEPKPEPKPEPKPEPKPEPKPEPKPQPKPEPEPKPEPKPKPDP
PHIPPAADN

>TC|TC110238||Poaceae_S.officinarum||PEPKA
MAAPRRLSSCCLLFAVLLGAVLATATAFFDEAAAAGVGLGHGARFARKHGRAVAELPQPE
AQPKPEPKPEPQPQPEPLPKPEPQPKPTPKPEPKPRGEAGPKPKPEPEPTPHPRPEPIPE
PEPEPKPEPKPEPKPEPKPEPKPEPEPKPEPKPEPKPKPKPEPVPKPEPKSEPKPEPKPE
PKPEPKPEPLPKPEPEPKPKPKPEPKPEPLPKPEPKPEPEPKPEPLPKPEPKPEPKPKPK
PEPLPKPEPKPEPKPEPKPEPLPKPKPKPGPKPKPLPKPEPKPKPKPEPLPKPEPEPKPE
PKPEPKPKPEPKPEPKPEPEPEPKPEPEPKPEPKPEPKPEPKPEPKPEPKPEPKPKPEPK
PKPKPEPQPKPEPKPQPKPEPEPKPEPKP

>TC|BE704847||Poaceae_S.cereale||PEPKA
MARHGLLAVLLVGLVAASGFSQAAAAGRGLAEKLPTPYPEPKPEPKPEPMPKPEPMPKPE
PKPLPKPEPMPKPEPKPLPKPEPMPKPEPKPEPKPEPMPKPEPKPEPKPEPMPKPEPKPE
PKPEPMPKPEPKPEPKPYPMPKPEPKPEPKPEPMPKPEPKPEPKPEPMPKPDPKPEPMKP
EPKPMXNP

>NR|gi:148727868||Poaceae_S.bicolor||PEPKA
MAATRRLSSCCLLLAVLLGAVAGTATAFFVDEAAAAGVGLGHGGRFARKHGRAAAELPQP
EPQPKPEPQPQPQPLPQPEPKPEPKPEPKPRPEPKTEPHPEPSPKPEPKPEPQPKPITKP
EPKPKPEPMPKPMPEPKPKPKKPEPKPKPEPKPEPKPEPEPEPKPEPLPKPEPKPEPKPK
PEPKPEPKPEPKPEPMPKPKPEPKPEPKPEPKPEPMPKPEPKPEPKPKPKPEPKPEPKPE
PKPEPLPPKPEPKPEPKPEPKPEPKPEPKPEPLPKPEPKPEPKPEPKPKPLPKPEPKPEP
LPKPEPKPEPKPEPKPEPLPKPEPKPEPKPKPEPKPEPKPEPKPEPLPKPEPKPKPEPKP
EPKPKPEPKPEPEPKPEPKPEPKPEPKPEPKPKPEPKPEPKPKPNPPHIPPAADN

>TA|TA3637_132711||Poaceae_S.propinquum||PEPKA
MAATRRLSSCCLLLAVLLGAVAATATAFFVDEAAAAGVGLGHGGRFARKHGRAAAELPQP
EPQPKPEPQPQPQPLPQPEPKPEPKPEPKPRPEPKTEPHPEPSPKPEPKPEPQPKPMPKP
EPKPKPEPMPKPMPKPKPKPKKPEPKPKPEPKPEPKPESKPEPKPEPLPKPEPKPEPKPE
PEPKPKPEPKPEPEPKPEPEPMPKPKPEPKPEPKPEPKPEPIRKPEPKPEPKPKPKPEPN
PEPKPEPKPEPLPPKPEPKPEPKPEPKPEPKPEPKPEPLPKPELKLEPKPEPKPEPLPKP
EPKPEPKPEPKPEPKPEPKPEPLPKPEPKPKPEPKPEPKPKPEPKPEPEPKPEPKPEPKP
EPKPEPKPKPEPKPEPKPKPDPPHIPPAADN

>TA|TA134_29706||Poaceae_S.alterniflora||PEPKA
MATPRVSSCCLLLLTVLLGAVAAATAFDEAAAAGVGLGHGARFARKPGRVAAEKPEPQPM
PQPKPESKPEPMPHPEPKPEPEPKPMPRPEPKPKPKPEPKPNPRPHPRSHPKPMPEPEPK
PKPLPEPGMKPGPKPEPKPEPKPEPKPEPEPKPEPKPMPKPEPKPEPKPEPEPKPEPKPE
PKPKPEPKPIPKPEPKPEPKPEPEPKPEPKPE

>TC|TC283777||Poaceae_T.aestivum||PEPKA
MARHRLLAVLMLLVGVVAASTFHQAAAAGRGLAVLEKFADLEPKPKPKPEPMPKQMPKPE
PESKPKPKPMPKPEPKPKPMPKPEPKPEPKHKPMPKPEPKPKPMPKPEPKPEPMPKPEPK
PKPEPKPKPPPKHKPPTAYN

>TC|TC282852||Poaceae_T.aestivum||PEPKA
MARHRLLAVLLIGIVAASAFNQAAAAGRGLAAVEKFAELEPKPEPKPEPMAKPMPPHPEP
EPCKPEPMPKPKPKPEPKPGQKPDPEPKPKPMPKPKHKHCSKPKPGPKPEPMPKPEPKPE
PEPKPESKPKPPPKCKPPTAHN

>NR|gi:21842||Poaceae_T.aestivum||PEPKA
MARHSLLAVLLVGLVAASGFSQAAAAGRGLAEKLPEPEPKPTPYPEPKPQPKPEPMPKPE
PMPKPEPKPLPKPEPMPKPEPKPLPKPEPMPKPEPKPEPKPEPIPKPEPKPEPKPDPMPK
PEPKPEPKPEPMPKPEPKPEPKPEPMPKPEPKPEPKPEPMKPEPKPMPKPEPKPEPKPEP
MPKPEPKPEPKPEPIKPEPKPMPKPEPKPEPKPEPMPKPEPKPEPKPEPMPKPEPKPEPK
PEPMPKPEPKPEPKPYPMPKPEPKPEPKPEPMPKPEPKPEPKPEPMPKPEPKPEPKPEPK
PEPMPKPEPKPEPKPEPMPKPEPKPEPKPEPMPKPEPKPKPLPKPEPKPEPMPKPEPKPE
PEPKPEPPPKGKPPMTDN

>TA|TA1966_4568||Poaceae_T.monococcum||PEPKA
MARHSLLAVLLVGLVAASGFSQAAAAGRGLAEKLPEPEPKPMPYPEPKPEPKPEPMPKPE
PMPKPEPKPLPKPEPMPKPEPKPLPKPEPMPKPEPKPEPKPEPMPKPEPKPEPKPDPMPK
PEPKPEPKPEPMPKPEPKPEPKPEPMPKPEPKPEPKPEPMKPEPKPMPKPEPKPEPMPKP
EPKPEPKPYPMPKPEPKPEPKPEPMPK

>NR|gi:162463364||Poaceae_Z.mays||PEPKA
MAATRRLSCFLLAVLLAGVAAATAFDEAAAAGFGLGHGARFARKHGRAAAEMPQPEPQPK
TKPEPHMQPLPQPEPKPKPMPHPEPKPEPQPKPNPEPQPMPKPQPKSKPEPLPTPKPEPK
PEPKPEPKPEPKIKPKPKPEPKPEPKPEHKPEPKPEPKPKPKPEPKPEPQPKPEPKPEPK
PEPKPEPKPEPQPKPEPKPEPKPEPKPEPQPKPEPKPEPKPEPKPEPKPEPKPEPKPEPK
PEPRPEPKPEPKPEPKPKPDPKPEPQPKPEPKPEPKPEPKPEPKPEPKPEPKPEPKPEPR
PEPEPKPEPKPEPKPKPEPEPQPKPEPKPDPKPEPKPEPKPEPQPKPEPKPEPKPQPEPK
PEPKPKPDPPHIPPAAIN

**@ PEPKB Class [17 Proteins, 11 Species]**

>TA|DV867187||Poaceae_A.stolonifera||PEPKB
MRLSALPLCWLLALVVANVASLADGGVQVMGDPKPEPKPEPAPQPQPDPKPTPQPEPKPD
PQTDPKPVPKPDPKQDPQPDPKPVPKTDPKPKPDPKPSPQPDPKTDPQPDPKPTPKPKPG
PKPAPQPDPKPDPKPQPPEQSSSQPKLPPQSPPSGNFN

>TC|TC6359||Poaceae_F.arundinacea||PEPKB
MVATMRPSALPLCLLLALVVANVASLADGRVQVMTDPKAPEPEPKPEPQPDPKPAPQLDP
KPDPKPVPKPDPKPDPKPAPKTDPQPDPKPAPKTDPKPKKDPKPSPQPDPKTDPQPDPKP
TPKPKPGPEPAPQPGPKPPQPPEHVASEPPNSPN

>TC|TC8348||Poaceae_F.arundinacea||PEPKB
MRPSALSLCLLLALVVANVASLADGRVQVMGEPLEPIPELTPQPQAQVMGEPLEPIPELI
PQPQAQVMGDPLEPIPELTPQPQAQVMGDPLEPIPELTPQPQADTKPTPQPDPKPVPKLD
PKPDPQPDPKPVPTTDRKPKPDPNPTPQPGPKPDPRPQPPEQSSSQPKQPPLSPPGYFN

>TC|TC11999||Poaceae_F.arundinacea||PEPKB
MTMSQRQSSPPLLLLVALALLLLPAFAVAAGRMLDDLPERYLPDRPGRPRPRPIPKDPQP
DPNPQPLPGPKPDPNPQPLPGPKPDPNPQPLPGPQPDPNTKPEPESQPDPNPKPLPGPQP
DPNPKPEPEPQPDPNPNPLPGPQPDPNPQPEPEPQPDPNPKPLPGPQPDPNPKPEPEPNP
QPLPGPLPDPKPHPLPGPQLDPNPNPQPLPGPQLDPNAEPKPPQQEKYVKAGVQEEPLG

>TC|TC160831||Poaceae_H.vulgare||PEPKB
MRAPAFSLCLLLALVAANVSSLALAGRVPELKLEPSLEPKPTPQPDPKPDPRTEPKLAPK
PDPTPKPDPKLPPKPDPQPDPKPAPKSDPTPKPDPKPAPKPDPAPKPDPRPDPQPDPKPV
PKPDPKPTPGPKPAPGPGPKPDPKPQPPEQSSSQPKQPPRSPPGYFN

>TC|TC166063||Poaceae_H.vulgare||PEPKB
MRSLGRTSVCFHLALLLLLAMVTADDPSLADGGRLIDPKPSPKPNPKPMPQPSPKTPLPN
PKPTTPVKPEPNPTPDPDVKPQPKPMPRPNPKPTPVKPEPKPDPDAKPHPKPMPQPNPKP
TPDEKPKPEPQPGSFPVYSLDTVADRANPKPDPQPNPRTNPEKPDRDVKPQPKPKPKPQP
NPNPTPAKPEPKPQPGPSKPTPLDSLSVRSVKGNPKPDPHPNPKPTPKPTPKPQPNPNPQ
PGSSKPKPPVYARRAGSDKPLN

>TA|DY895754||Poaceae_L.chinensis||PEPKB
MRPSAFSLCLLLALAATNVASLALAGRVPELKHEPSLEPKPTPQPDPKPDPQTDPKLAPK
PDPTPKPDPKLAPKPDPQPDPKPAPKPDPTPKPDPKLAPKPDPTPKPDPKPDPQPDPKPG
PKPDPKPTPDPKPAPSPSPKPDPKPQPPEQSSSQPKQPPRSPSGYFN

>TA|AU250571||Poaceae_L.multiflorum||PEPKB
MVATMRPSALPLCLLLALVVANVASLADGRVQVMADPKAPEPEPKPEPQPDPKPAPQLDP
KPDPKPVPKPDPKPDPKPVPKTDPQPDPKPAPKTDPQPDPKPAPKTDPKPKKDPKPSPQP
DPKTDPQPDPKPTPKPKPGPEPAPQPGPKPPQPPEHEASEPPNSPN

>NR|gi:115471749||Poaceae_O.sativa||PEPKB
MRRSILSLCFHLALVIALAANVPDIANGRVIEAKSDPKPADPKPKPDPTPKPQPETKPSP
QPNPQPNPQPDPKPSPQPDPKPTPQPEPKQDPQPNPQPDPKQSPQPDPKPTPQPNPKQDP
QPNPQPDPKPTLQPNPKQDPQPNPQPNPKPTPQLDPKQDPQPNPQPSPKADPKPNPKPKP
QPEPSPNPKPEPKPEPKPEPSPNPKPNPNPKPEPQPDPKPEPKPQPEPSQPKLPPLSPAI
AIIVPGN

>TC|TC1753||Poaceae_P.virgatum||PEPKB
MRSALLSLCFHLALAATLLAASAPGLACGRVIDPKPRPQPTPQPDPKPVPGPDPKTEPKP
APQPGPKPEPQPDPKPEPKPEPQPDPKPEPHPAPQPGPKPDPNPKPAPQPDPKPEPQPGP
NPIPQHDPKPEPEPAPQPDPKPEPQPDPNPTPQPDPKPEPEPQPDDPNNPDAQPAGLKPE
PQAEQPSSSSVVHVVPRPSTKPTLEPKRKPEPSPKPTPDPEPRPQPEPSKPEPEPSPKPT
PEPKPQPEPSKPEPQPSKPEPAPLSSRIGTATTTMEGN

>NR|gi:242079587||Poaceae_S.bicolor||PEPKB
MRSGLLSLCFHLALAITLATSVPDLVHSRVIDLKLQPKPIPQPEPKPAPQPDPKPEPKPT
PHPDPKPTPQPDPKPEPKPAPQPEPKPAPQPDPKPEPKPTPQPKPKPTPQPDPQPEPKPT
PQPDPKPEPKPTPQPDPKPEPKPTPQPPSSQPEPKPEPEPSPKPTPEPKPQLEPQPSPKP
QPEPSKPEPQPRPKPTPEPKPQPEPSKPEPPSLPPPIGAGLMEGN

>TC|CA278231||Poaceae_S.officinarum||PEPKB
MAKRRRSSSLPLALLPPLLLLLAAGSLAVATALDSSPEARRRGRSLQQLPTPLPQPVPQP
QPDPNPQPQPGPLTQPLPLPNPNPQPQPLPQPDPNPQPQTKPLPLPKTEPNPKTQPQPLP
KTEPNPQA

>TC|TC73659||Poaceae_S.officinarum||PEPKB
MRSGLLSLCFHLALAITLAANVPGLAHSRIIDLKPQPKPTPQPEPKPAPQPDPKPEPKPT
PQPDPKPAPQPDPKPEPKPSPQPEPKPAPQPDPKPEPKPTPQPEPKPAPQPDPKPEPKPT
PQPEPKPDPKPTPQPDPKPTPQPPFPQPEPKPEPEPSPKPTPGTKPQPEPQPSPKPQPEP
SKPEPQPMPKPTPDPKPKPEPEPSKPEPPSLSPPIGTGIMEGN

>TC|TC278028||Poaceae_T.aestivum||PEPKB
MRATAFSLCLLLALVAANVASLAVAGRVPGLKHEPSLEPKPTPQPDPKPDPQTDPKLAPK
PDPTPKPDPKLAPKPEPKPDPQPDPKPVPKPDPTPKPDPKPAPKPDPTPKPDPKPAPKPD
PAPKPDPKPDPQPDPKPGPKPDPKPTPDPKPAPGPSPKPDPKPQPPEQSSSQPKQPPRSP
SGFFN

>TC|CJ666632||Poaceae_T.aestivum||PEPKB
MRTRPSSSSLLVAPLLLMLLPAFALAAGRHTLITRDPQYGPRANPNPEPLPGKQPNPNXQ
PLPDPQPLPGPRPDPNPQPLPDPKPNPNPQPLPEPQPDPNAQPLPGPHPDPNPQPLPGQN
LQRLMNPRXNPNPXLLPNPRPNPNPRPLPGPQPDPNPQPLXDPNSKPLPDPQPNPNPQPL
PGXQPDPNPXPFPDPNPKPLPNPQPNPNPQPLPGPQPNPXPQPLXDPXPKXLXDPQXNPN
PQPLPGPQXDPKPHKPLXKPKP

>TC|TC304000||Poaceae_T.aestivum||PEPKB
MRSLGRTSLCFHLALLLLLAVVRADDPSLADGGRLIDPKPSPKPIPKLMPQPNPKMPHPN
PNPNPNPKPTTPAMPEPNPIPDPDAKPQPKPMPQPDPKPTPEKPKAEPQPGSLPVYSLDD
IGSDRTNPKPDPHPNPKANPRKPDPDAKPQPKPMPQPNPKPNPEKPKPKPQSGSLPVYSL
DGVSDRTNPKPNPQPNPRTNPAKPDPEVKPQPQPKPQPNPKPTPAKPDPEIKPQPQPNPK
PTPAKTEPEPRPGPSKSTPLDSLSVRFDKVNPKPDPHPTPKPTPKPQPNPNPHPGPSKPK
PPVYARRVGSDKPLN

>NR|gi:226508062(revision)||Poaceae_Z.mays||PEPKB
MRSGLLSLCFHLALAITLAASVPGLARSRVIDIEPQLKPTLQLEPKHGVSQPDRNQEPKP
TPQPEPKPEPKPEPKPAPQSDTKSDPKPAPQSDPKPAPQPDLKPEPKPTPQPDPKPSPQP
DPEPKPKPAPQPEPKPEPKPTPQPDPKPGPQPDPKPEPKPTPQPGPEPKPKPAPQPEPKP
TPQPDPKPEPKPTPQPDPKPEPKPTPQPPFPQPEPQPDPKPQPEPSKPDPKPQPEPSKPD
PQPEPKPTPEPKPQPEPSKPEPPSSLPPPFGTGVVEGN

**@ PEPKC Class [8 Proteins, 6 Species]**

>TA|TA4908_29729||Malvaceae_G.arboreum||PEPKC
MMSTTHLLVFLLGVVTLTTPTFGTYESPNYGKPPTPEFKPPKVKPPPYEPKPPVYEPPKK
EKPEPKPPVYAPPKKEKPGPKPPVYEPPKKEKPEPKPPVYTPPKKEEPKPKPPVYEPPKK
EKPEPKPPIYTPPKKEKPEPKPPVYEPPKKEKPEPKPPVYTPPKKEKPEPKPPVYEPPKK
PPMYEPKPPKPPVYTPPKKEKPEPKPPMYEPPKKPPMYEPKPPKPPVYTPPKKEKSEPKP
TMYQPPNNPPYMSQTT

>NR|gi:45644504||Malvaceae_G.barbadense||pepk-embed2-containing
MMATTHLLILLLGVVALATPSFGTYESPNYGKPPAPVHNPPKLKPPPYEHKPPVYEPPKK
EKPEPKPPVYVPPKKEKPEPKPPVYKPSKKPPVYEPKPPKPPVYAPPKKERPEPKPPVHE
SPKKTTIWSLSRTPSIGEASIERFPRLTIPDN

>NR|gi:119888004||Malvaceae_G.hirsutum||PEPKC
MSTTHLLVFLLGVVTLTTPTFGTYESPNYGKPPTPEFKPPKVKPPPYEPKPPVYEPPKKE
KPEPKPPVYAPPKKEKPGPKPPVYEPPKKEKPEPKPPVYTPPKKEEPKPKPPVYEPPKKE
KPEPKPPIYTPPKKEKPEPKPPVYEPPKKEKPEPKPPVYTPPKKEKPEPKPPVYEPPKKP
PMYEPKPPKPPVYTPPKKEKPEPIPPMYEPPKKPPMCEPKPPKPPVYTPPKKEKSDPSQP
CINLPQPTIYEPNHLSHH

>NR|gi:2829206||Malvaceae_G.hirsutum||PEPKC
MMSTTHLLVFLLGVVTLTTPTFGTYESPNYGKPPTPVFKPPKVKPPPYEPKPPVYEPPKK
EKPEPKPPVYAPPKKEKHGPKPPMYEPPKKEKPEPKPPVYTPPKKEEPKPKPPVYEPPKK
EKPEPKPPIYTPPKKEKPEPKPPVYEPPKKEKPEPRPPVYTPPKKEKPEPKPPVYEPPKK
PPMYEPKPPKPPVYTPPKKEKPEPKPPMYEPPKKPPMYEPKPPKPPVYTPPKKEKPEPKP
PMYQPPNNPPIYEPKPPKPPVYAPPKEEKPKPKPPVYDAPAHEPPYGHYPGHPPLGKPQ

>NR|gi:14582310||Malvaceae_G.hirsutum||PEPKC
MMSTIHLLILLLGAVAFATPSFGTYENYGKPPTPVYKPPKVKPPPYEHKPPVYEPPKKEK
PEPKPPVYTSPKKDKPEPKPPVYEPPKKEKPEPKSPVYTPPKKERPEPKPPVYEPPKKEK
PKPKPSVYTPPKKEKPEPKPLVYEPPKKEKPEPKPPVYTPPKKEKPEPKPPMYEPPKKEK
LEPKPLVYAPPKKEKPEPKPPVYEPPKKPPVYEPKPPKPPVYASPKKEKPEPKPPMYEPP
NKPPMYEPKPQKSPVYVPPKKEKPEPKPPMYEPPKKPPVHKPPYGHYPRHLHWGSLNREA
PSASHPHELS

>TC|CO088717||Malvaceae_G.raimondii||PEPKC
TTHLLILLLPLVALATPSFGTYESPNYGKPPAPVHNPPKLKPPPYEHKPPVYEPPKKEKP
EPKPPVYTPPKKEKPEPKPPVYEPPKKEKPEPKPPVYTPPKKEKHEHKPPVYEPPKKEKP
EPKPPVYAPPKKEKPELKPPVYKPPKKPPVYEPKPPKPPVYAPPKKEKPEPKPPVYESPK
KPPYGHYPGHPPLEKPQ

>TC|TC2143||Malvaceae_T.cacao||PEPKC
MSLTHLLVLLLGVVVLATPSLGTYELPGYGKPPTPVYKPPEVKPPPIYKPPPVYEPPKKP
EPQPPVYEPPKKEKPEPKPPVYEPPKKEKPEPKPPVYEPPKKPPVYEPPKKPPVYEPKPP
KPPVYEPKPPKPPVYEPPKKPPVYEPKPPKPEPKPPVYEPPKKPPVYEPPKKPPVYEPKP
PKPPVYEPKPPKPPVYEPKPPVYEPKPPKPPVYEPPKKPPVYEPPKKPPVYEPKPPKPPV
YEPKPPKPPVYEPPKKEKPEPKPPVYEPPKKPPVYEPKPPKPPVYEPKPPKPPVYEPPKK
EKPEPKPPVYEPPKKPPVYEPPKKPPVYEPPYGHYPGHPPMGKP

>TA|TA2068_39984||Rhizophoraceae_B.gymnorrhiza||PEPKC
MQLTYLLVLLLGAVALSTTPSFADYEPPKEKPFPEPKPPVYEPPKEKPFPEPKPPVYKPP
KEKPLPEPKPPVYEPPKEKPFPAPKPPVYGPPKKEKPPLEPKLPVYEPSKEEKPFPEPKP
PVYEPPKKEKPFPEPKGPKPPVDKPPKKEEPFPKPRGPKPPVYEPPKKGKK

**@ KPIP Class [10 Proteins, 7 Species]**

>TA|CD037772(revision)||Fabaceae_A.hypogaea||KPIP
MHNTFMRFLIPLLVLATFCLANSAIATSQVSENEVKLGDQELAKTKPEVHNEEEKFLFHH
HKSLFKKHIPIVKPIPVYKPIPKPIPIXEAIYKPVPIYKPIPKPVIVKKPVPIPVFKPVP
KPIPIVKPIPVFKPIPKPIPIVKPIYKPVPIYKPIPKPIFVKPYPKKPYFPPQNSP

>NR|gi:255635993||Fabaceae_G.max||kpip-containing
MLLYRVPLLLFVCFCFANSAAVSANEASTGTEVTIDAKPSDKKLKGTEPFPKEKKTFSIP
FFKPVPKLVPIVKPIPMPFPKPLPLPIPVFQKPIPKWIPDVKPIPKPIPIPVFRPVPVPV
KPIPKPSPTIEKPKPCPIAKKPVPTPVVKKPLPKPIPIVKSIPKPSPTVKKPLPIPSIKP
VPKSIPIAKPIPIPVFKPIPKPFPIEPLPIPVIKPIPTPIPIPLFKPIPSISYSGKALPI
PYMLYP

>NR|gi:255636435||Fabaceae_G.max||KPIP
MRMRKQSTMQCALFRFWVPLLLLASFSYAPSVLATTEISGNQVNMDGKVVNEELGKPNLK
GQDEEEKFKGLFPKPIPIVKPFPKLIPIIKPIPKPIPVVKPIPIPLYKPFPKSIPIVKPI
PKPIPNGKPIPTEEAKFKGFFPKPIPIVKPIPVAKPIPIVKPFPKLIPIIKPIPKPIPIV
KPIPIPVYKPIPKSIPIVKPIPNGKPIPTEEAKFKGFFLKPIPIVKPIPVAKSIPIVKPI
PITVYKPITKSFPTVKPIPKPIPILKPIPKPISIVKPISKPFIVQKPIPAVEPEKFLKPK
PFFKKPLPKFPLNPKFKKPLLPPFPIHKAIPTP

>TA|TA2966_3848||Fabaceae_G.soja||KPIP
MRKPSSLQSALLRFGVPLLLVITFCYATSTAAATTQVSGNEELPKTNLNGHDEEAKFGFF
HHKPIFKKHIPIPVYKPVPKPFPVYKPIPKPVPVPVYKPIPKPVYKPIPKPVPVYKPIPK
PVPVPVYKPIPKPVYKPIPKPVYKPIPKPVPVYKPIPKPVPVPVYKPIPKPVYKPIPKPV
YKPIPKPVPVYKPIPKPVPVYKPIPKPVYKPIPKPVPVYKPIPKPVPIYKPIPIFKPIPK
PVPIYKPIPIFKPVPKPVPIYKPIPIFKPVPTPIPFFKPIPKPVPIVKPIPIPVFKPIPK
PFPIVKPIP

>TC|TC44511||Fabaceae_L.japonicus||KPIP
MRNPSTLQRAFVLFCLPLLLVATFGYATEVSGNEVKTDGKVAQEEVAKTSQEGPNEEAKF
KGFFHHKPIIFKKPFPFVKPIPKPFPFVKPIHKPFPFVKKPIPKPIFVKKPIPFYKPVPK
VIPVLKPIPIYKPVPKVIPVPIYKPVPKFIPVVKPIPILKPVPKVIPIVKPIPFFKPVPK
VIPIVKPIPIHKPIPTP

>TA|TA736_3879||Fabaceae_M.sativa||KPIP
MRKSSNMQRPLLAFWVPLLLLVTFCYATSVVARNDPSGNQVNTDGKLANEEATKTNLEGH
NEEEKFKGFFHLKHKLKGYFHQKPIFKKPIPFYKPIHKDIPIYKPIPKYVPTYKPIHKPI
PIYKPIPKVIPIYKPVPIYKPIPKVIPIYKPVPIYKPIPKVIPIYKPIPKVVPIVKPIH

>LOC|Medtr8g040150||Fabaceae_M.truncatula||KPIP
MRKASNLQRPLLAFWVPLLLLVTFCYATSVVARNDPSGNQVNTDGKLANEEATKTNLEGH
NEEEKFKGLFHLKHKLKGYFHQKPIFKKPIPFYKPIHKDIPYYKPIPKYVPIYKPIHKPI
PIYKPIPKVIPIYKPIPKVIPIYKPVPIYKPIPKVIPIYKPIPIYKPIPKVIPIVKPIH

>TC|CB539927||Fabaceae_P.vulgaris||kpip-containing
MTKGFTLQCALLFFFLATFSYAPSVLATTQITGNEVNTDGKVSNEEFAKASIEGDDEEAK
FKGFFPKPIHILKPIPKPIPIVKPTVKPFPFPVYKPTSKPIPIVKPILKPIPNEEAKFKG
FFPTKQIPVVKPIPKIIPVVKPVPVKVYKPVPKLVPIVKPIPILKPIPKQIPILK

>TC|TC9121||Fabaceae_P.vulgaris||KPIP
MRMHSTLQSAVLRFVPLLLVITFCYAVTSAATTQVSGNEVNTNGIASEELTKTKHYEEEK
FFHHHKPLFKKPIPFIKPVPKPIPFVKPIPIYKPYPKPVPVPVYKPIPVYKPVPIYKPIP
KPVPVYKPVPIYKPVPKPVPIYKPIPKPVPVYKPIPKPVPVYKPVPIYKPIPKPVPIYKP
IPKPVPVYKPIPKPVPVYKPIPKPVPVYKPVPIYKPIPKPVPIYKPIPKPVPIYKAYSKT
GSSIQACSNSSV

>TC|TC14594(revision)||Fabaceae_P.vulgaris||KPIP
MRMYCALLCFWFPLLLLSNLASVFATTQISGNEVNKYGNNVEGHDEESKFKGLFTKPIPF
VKPILKPIPVIKPIPKPIPVIKPILKPIPVIKPILKPIPVIKPILKPIPVIKPILKPIPV
IKPIPKPVPVIKPIPIPVFNKPVPKSISIKPTPKPIPDIKPIPSEEVKFKGLFPKPIPKI
KAIPKVIPVVKPIYIPVVKPLPKLVPIVKPIPILKPKLVPIVKPIPILKPIPKPFTVKKP
VPTVESEELLKPKPLFKKYIPKIPFHHQFKKPFVAPLPIHKPIPTP

**@ MPAV Class [39 Proteins, 8 Species]**

>TA|DV479832||Poaceae_B.distachyon||p2mpav-containing
MAGNRSFCLALVMSCALLIAGSSSTCHAARRLADDTTPAAAAAPAAVPTVTFPSVPSVTL
PTVPQVTLPPIPSIAVTLPPMPKVTVSPMPAIVVPKVTMAPMPAIVVPKVTLPPMPFVPS
VNVPMPTLAPPPSA

>TA|DV488241||Poaceae_B.distachyon||MPAV
MAAAGTCSFLAIAMALALLSGNMTHAGRLLADTTEAAAPTASPAAVPGIPLPKPPVPTVP
TMPTVPAVTIPQLTVPPMPAVPAVTVPQLTVPPMPAVPAVTVPQVTLPPMPAIVVPKVTM
PPMPSISIPSIPKVALPPMPSIPSVTVPMPFLAPPPSA

>TC|TC190717||Poaceae_H.vulgare||MPAV
MASSASMLAMIMACALLLAGSTCDAARNLADTTPEAAVPAASTVPGLPTVPTDTVTLMPP
MPSVTFPTVPQVTLPPMPAIVVPKAVLPPMPKVTLPTVPQVTMAPMPAIVVPKVTLPPLP
LVPNVNVPMPFAAPPPSA

>TC|BM817160||Poaceae_H.vulgare||MPAV
MASTTSFFVMIMACTLLASTTCHAARHLADTTPVATTPAAAAVPGLPAVPTMPSLPPMPA
VPTVPALTVPPMPTLPTVPQVTLPPMPAVPAMPKVTMPPMPAIVVPKVTMAPMPAVVMPK
VTMPPMPAIPSMPKMTLPPTPSIPAVNVPMPFLAPPPST

>TC|TC188414||Poaceae_H.vulgare||MPAV
MAVSTCSLLAVAMALALLSGNMAHASRLLADTTEAASPTATPAAVPGIAAPKPPVPTMPT
MPPVPTVPTVTTPQVTVPPMPAVPAITVPQLTIPPMPTVPAITVPQVTLPPMPAVPTVTM
PPMPAVVVPKVTMPPMPAIPSISIPKMTLPPMPSIPTVNVPMPFLAPPPSA

>TC|TC177318||Poaceae_H.vulgare||MPAV
MASTSMSFVAMALLMAAMLLAGGGNTCHAARLLADVPALPEPTLPTLPPVPVVPTVPAVP
GGVVPTVPTVPTVPGVPTVPMPTVPGGVVPTVPTVPTIPTVPTVPGVPTVPTVPGVPTVP
LPAVPGAVVPTVPTVPGVPTLSLPSMPTIPGLPNVPLPPMPSIPGDLPKLPVPLPPMPSI
PGDLPKLPVPLATMP

>TC|TC165795||Poaceae_H.vulgare||MPAV
MASTSTSFVAMALLMAVMLSTCHAARLLADVPALPIPTLPPVPVVPTVPAVPGGVVPTVP
TVPNVPTVPTVPGIPTAPTVPTVPGVPAIPTVPGVPAVPVPGAAVPTIPAVPTVPGVPTV
PLPAVPGAAVPTVPTAPSVPMVPTVPGVPALPMPSVPGVPNVPLPPMPSIPDLPKVPLPP
MPVVPDLAKVPLPPMPVGPGGPKCHWRRRRWSGVPAVP

>NR|gi:115470547||Poaceae_O.sativa||MPAV
MASSSNLLAMAIAVAAVLLAGNGNTGHAARHLADTTEAPAAAIPAVPAMPKPTIPTIVPA
VTLPPIPAVPKVTLPPMPAIPTVPAVTMPPMPAVPAVPAVTLPPMPAVPTVPPNTVVVPA
AVVPALPKVALPPMAAVPNVPMPFLAPPPKA

>NR|gi:108711400||Poaceae_O.sativa||MPAV
MASSVSFMATAMAMACVLLAASSSRTCYAARMLADTPATAAAAAAPPAALPVLPAVPALP
TTLPPMPAIPAVPQAALPPMPAVPTVPAVVPNAAALPPMPAVPAIPAVPKVSLPPMPAVP
AVTLPPMPAGIPAVPAVTLPPMPSIPTVNVPMPFQAPPPSA

>NR|gi:115459480||Poaceae_O.sativa||MPAV
MAATSGSCLAFVMALALIGTNVSYAARLLADATESATPTASPAAVPGSIPAVPKLPVPTA
MPPMPAVPAATVPQVTMPPMPAVPAVTLPPMPAVPAVTVSTVTVPPMPAVPKVTLPPVPA
VVVPKVTMPPIPAGIPKVALPPMPAIPAANVPVPFLAPPPSA

>NR|gi:115470545||Poaceae_O.sativa||MPAV
MASTSGLLATVLAMAVLLAGSSSSCQAARHLADATPPAAVPVPTVPAVTLPPMPAIPAVP
AATLPPMPAVPTVPNAALPPMPAVPKVALPPMPAVPAVPTVPAVPAVPAASLPPMPAVPA
VPNAVLPPMPAVPKVTLPPMPSMPAVPKVTLPPMPSVPMPFLAPPPSA

>NR|gi:115470541||Poaceae_O.sativa||MPAV
MASNSVLFATVLAMAVVLTGSSSCQAARLLADATPPAVPAVTLPPMPAIPAIPAATLPPI
PAVPTVPNAALPPMPAVPKVALPPMPAVPAVPTVPAVALPPMPAVPAVPTATLPPMPAVP
AVPNAVLPPIPAVPKVTLPPMPSIPAVPKVTLPPMPSVPMPFLAPPPSA

>NR|gi:115470543||Poaceae_O.sativa||MPAV
MASTSGLLATVLAMAVLLAGSSRSCQAARHLADATPPAAVPVPTVPAVTLPPMPAIPAVP
AATLPPMPAVPIVPNTALPPMPAVPKVALPPMPAVPAVPTVPAVTVPPMPAVPAVPAATL
PPMPAVPAVPAASLPPMPAMPAVPNAALPPMPAVPKVTLPPMPSMPAVPKVTLPPMPSVP
MPFLAPPPSA

>TC|TC14785||Poaceae_P.virgatum||MPAV
MASAARFLAVVMACALLGSNSCYASRHLADTTPAAAAPAAAVPGLPAVPTLPTVPQRPAA
TVPPVTLPPMPVVPAVTVPQVALPPMPVVPTVAVPQVALPPMPAVPSVPKVALPPIPAVP
SVPKVALPPMPSIPNANIPMPFLAPPPSA

>TC|TC29166||Poaceae_P.virgatum||MPAV
MASSSPIFATALVMALVLASSATSHAARRLADTAPAAAPAAVVPGIPAVPKPPVPTVPAV
PTVPTVTVPPVPKVPAATLPPMPAVPAVPAATLPPMPAVPTTVPNAALPPMPAVPKVTLP
PMPAVPKVTLPPMPSIPSGVPVPFLAPPPSA

>TC|TC12769||Poaceae_P.virgatum||MPAV
MASGSAVFATALVMALLLAGSTTSHAARRLADTAPAAAPAAVPAGIPAVPKPPVPTTVPA
VALPPMPTVPTVPAATVPPVPQVPAVPAATLPPMPAVPAVPAATLPPPVPKVTLPPMPAV
PKVALPPMPAAPKVTLPPMPSVPGVPMPFLAPPPSA

>TC|TC4793||Poaceae_P.virgatum||MPAV
MASGSALFAAALVMALVLAGSTSSHAARRLADTAPAAAPAAVPGIPAAPKPPVPTVPAVA
LPPMPAVPTVPAATVPPVPKVPAVPAATLPPMPAVPAIPAVPTTVPSAALPPMPAVPAVP
KVTLPPMPAVPKVTLPPMPAVPKVTLPPMPSVPSVPMPFLAPPPSA

>TC|BE587697||Poaceae_S.cereale||MPAV
MASTASFLAMIMAFALLSSSTCHGARNLTDTTPPAAPTAAPIPGIPAVPTLPAVPPMPAV
PTVPAVTMPPMPTVPTVPQVTLPPMPAVPVPTVVPAVSVPPMPTMPTVPQVTVTLLPMPA
VPSVPKMTMPPMPAIVVP

>TC|BE587222||Poaceae_S.cereale||MPAV
MAVSTCSFLAVAMALALLSGNMAHAGRLLADTTEAASPTATPAAVPGIPAPKPPVPTMPT
MPPVPTVPAVTTPQVTVPPMPAVPAATVPQFTVPTMPAVPAITVPQVTLPPMPAVPAVTV
PKVMLPPMPAVVVPKVTMPPMPAIPSI

>NR|gi:242047370||Poaceae_S.bicolor||MPAV
MASTSSLPAVALVVALMLVLGNSGTCHAARLLADATAPAAAPAAAVPGIPALPKPTTVPA
IPTVPVVTMPPMPAVPAVTVPSMPAVPAVTLPPMPAVPSVPAVPAMPNAAALPPVPAAVV
PKLTTLPPMPSVPSIPKVTLPPTPSSIPGVPTMPFLAPPPKA

>TC|TC112059||Poaceae_S.bicolor||MPAV
MVSAANFLAMVVTCALLMTSRDTCHAARHLADTTAAAAAPAAAAAAVPGLPAVPTMPQAA
LPPMPAVPAVTVPQVTLPPMPPAPKVTVPQVTLPPIPAAPKVTVPPQVALPPIPAVPSSV
LPKLTLPPMPAAVVPSVGPKVALPPMPSVPAVNVPMPFAAPPPSA

>NR|gi:242079523||Poaceae_S.bicolor||MPAV
MARSGPGCRLLVLLLALTAFNGSFAARHLLDTSAAPQAAPAQPSIPAVPTKLPPVPSIPI
VPTTLTLPPMPSIPAVPTTAIPPIPSIPIPKSALPPTAAGAIPSLPNPVIPTTVPAIPKV
QVTLPPMPSIPTTVPSIPTMIPTTIPTIPGLQVPPLTAPSPQTTSP

>NR|gi:242042928||Poaceae_S.bicolor||MPAV
MASSASALFVKAALVMALMLAGSTTCHAARYLADSTSAAAPAAVVPTVPAVPAAAALPPL
AAVPTTVPAVTVPPKPTVPVVVVPAATLPPMPAVPAATLPPMPAVPAVPNAALPRMPAVP
AVPKATLPPPMPAVPAVPKVTLPPMAAVPKVTVLPPMPSIPGVPKPFLAPPPSA

>NR|gi:242042930||Poaceae_S.bicolor||MPAV
MAASATDPLATALVMAAVALMLTATTTHAARHLADTTPAAAPAAVVPGIPAVPKPPVVPT
VPAVVALPPMPAVPTTVPAVGAVPPIPAVPAVPAATTLPPMPSVPAVPAATLPPMPKVPT
VPTAAALPPMPAIPSVPKVALPPVVPSSVPKVTLPPMPSVPKVTLPPMPSIPGVPMPFLA
PPPSA

>TC|TC300038||Poaceae_T.aestivum||MPAV
MASNTSLLAVIMACTLLLTGHTCQGARHLADTTPAAAPTAAVPSLPAVPTLPAVPTDTVT
LLPPMPAVTLPTVPQVTLPPMPSVVVPKAVLPPMPKVTMAPMPAIVVPKVTLPPMPFVPN
VNVPMPFLAPPPSV

>TC|TC322244||Poaceae_T.aestivum||MPAV
MASAVSSLFFATALVMMALMIMLEGSTTCHAARHLADTTTAPTAAPTAIPAIPAMPKPPV
VPTVPAATLPPIPAVPTSTVPALGVPPIPALPKIPAVPPMPAVPALPKVTVTLPPPMPAV
VVPKVGRLAADARRA

>TC|CV764168||Poaceae_T.aestivum||MPAV
MASNASMLAVIMACALLLAGSTCHAARNLAGTTLAGAAPAASAVPGLPAVPTLPAVPTDT
VTLMPPVPSVTLPTVPQVMLPPMPAIVVPKAVLPPMPKVTLPTVPQVTMAPMPAIVVPKV
TLPLLPFVPNVNVPMPFAAPPPSA

>TC|TC289837||Poaceae_T.aestivum||MPAV
MASNASMLAVIMACALLLAGSMCHAARNLADTTPAAAAPAASAVPGLPAMPTLPAVPTDT
VTLMPPMPSVTLPTVPQVTLPPMPSIVVPKAVLPPMPKVTLPTVPQVTMAPMPAIVVPKV
TLPPLPFIPNVNVPMPFAAPPPSA

>TC|CV766758||Poaceae_T.aestivum||MPAV
MAHATWPTLLLRLPLPLLLSLASQRCRPCRQLCPQCHAMTVPPMPTVPTVPHMALPPMPA
MPVPTVPAATVPPMPTMPTVPQVTLPPMPIVPSMPKMTMPPMPAIVVPKLTMPPMPAIVV
PNVTMPQIPTIPSINVPMPLVAAPPSA

>TC|TC289507||Poaceae_T.aestivum||MPAV
MASTTRFLAMIMACTLLASTTCHAARHLADTTPAAAPPAAAAVPGLPTVPTMPTLPPMPA
VPTVPALTVPPMPTVPTVPQVTLPPMPAVPAVPKVTMPPMPAIVVPKVTMAPMPAVVVPK
VTMPPMPAIPSMPKMTLPPMPSIPTVNVPMPFLAPPPST

>TC|TC291967||Poaceae_T.aestivum||MPAV
MASTASFLAAIMVCAVLSSNTCHGTRNLADTTPPASPTAAPVPGIPAVPTLPAVPTVPAV
TVPQVTLPPMPAVPVPTVPAVTVPPMSTMPTVPQVTLPPLPVVPSVPKMTMPPMPSIVVP
KLTMPPMPAIVVPKVTMPQIPAIPSINVPMPFLAPPPSA

>TC|CA626862||Poaceae_T.aestivum||p2mpav-containing
MARGGSGCRLLALLLVLTAFNGSFAARHLLDTAAAPEATPAQPSIPAVPTKLPPVPTIPA
VPTTLTLPPMPSIPEVPKMTIPPIPSIPIPKLALPPTAAGAIPSLPNPVVPTTVPSYFCS
PGYPATMPSIPTTLPSXPTTIPTTILQSGSSASXTPIPQTQP

>TC|TC342811||Poaceae_T.aestivum||MPAV
MALLLAVMLSTLHSAPLLADFPAFPTPMLPPVARCPYSPPPCQPASFPTVPTVPGVPTVP
TIPTVPGVPTVPTVPGVPTVPVPGAAVPTIPAVPTLPGVPTVPLPAVPGAAVPTVPTAPS
VPTVPTVPGVPTLPMPSVPGIPDLPLPPMPSIPDLPKVPLPPMPSVPGVPKVPLPPVPSV
PGVPAVP

>TC|TC323432||Poaceae_T.aestivum||MPAV
MASTSTSFVAMALVTAVMLSTCHAARLLADVPALPTPMLPPVPVVPTVPAVPGGVVPTVP
TVPNVPTVPTVPGIPTVPTVPTVPGVPTVPTVPGVPTVPVPGAVVPTIPAVPTVPGVPTV
PVPGAVVPTVPTAPSVPTVPTVPGVPTLPMPSVPGVPNVPLPPMPSVPDLPKVPLPPMPS
VPGVPKVPLPPVPSVPGVPAVP

>TC|TC304035||Poaceae_T.aestivum||MPAV
MASTSTSFVAMALVMAVMLSTCHAARLLADVPALPTPMLPPVPVVPTVPALPGGVVPTVP
TVPNVPTVPTVPGVPKVPTVPTVPGVPTVPTVPGVPTVPVPGAALPTIPAVPTVPGVPTV
PVPGAVVPTVPTAPSVPTVPTVPRVPTLPMPSVPGVPTLPLPPMPSIPEPPQGAPGLRSP
PFPGVPQGAPGPRSPSGSPGGPARAISFLGPFH

>TC|TC296724||Poaceae_T.aestivum||MPAV
MASTPTSFVAVALLMAVMLSTCRAARLLADVPALPEPTLPTLPPVVPTVPALPGGVVPTV
PTVPEVPTVPLPTVPSGVVPTIPTVPTVPEVPTVPTVPGVPAVPLPTVPGGVVPTVPTVP
TVPGVPTVPLPAVPGAVEPTVPTVPSVPTLPLPTMPSIPSLPNLPLPPMPSIPGDLPKVP
VPLPPIPSIPGDLPKLPVPLPPMPSLPDLPKVPLAPMPPSLGCPDTPASVRVSGRAVFPL
TDD

>NR|gi:226510147||Poaceae_Z.mays||MPAV
MASAVSSLFFATALVMMTLMIMLEGSTTCHAARHLADTTTAPTAAPTAIPAIPAMPKPPV
VPTVPAATLPPIPAVPTSTVPALGVPPIPALPKIPAVPPMPAVPALPKVTVTLPPPMPAV
VVVPKVGALPPMPAVPKLTLPPMPSIPGVPMPFVAPPPSA

>NR|gi:226530501||Poaceae_Z.mays||MPAV
MASAARFLAVAVVVACALLASNNCHAARHLADTAAAPAAAAIVPGLPAVPTLPTVQPVPQ
VALPPMPAVPAVTVPQMTLPPMPAAPKVAVPLPPMPAIPSVLPKLTLPPMPAVVPSVVPK
VALPPMPSVPTVPSVVPKVALPPMPPVPTVNVPMPFAAPPPSA

>NR|gi:226502160||Poaceae_Z.mays||MPAV
MARGGSGCRLLALLLVLTAFNGSFAARHLLDTAAAPEATPAQPSIPAVPTKLPPVPTIPA
VPTTLTLPPMPSIPEVPKMTIPPIPSIPIPKLALPPTAAGAIPSLPNPVVPTTVPAISAV
PVTLPPMPSIPTTLPSIPTTIPTTIPTIPGLQVPPFTAPSPQTTSP

**@ PEHK Class [22 Proteins, 5 Species]**

>TA|CB288675(revision)||Vitaceae_V.aestivalis||PEHK
MSPTYLLVVLLGLVVLTAPSLADYPKHPPFEKPPPEHKPPMEKPPPEHKPPVEKPPPEHK
PPVEKPPPEHKPPVEKPPPEHKPPVEKPPPEHKPPVEKPPPEHKPPVEKPPPEHKPPVEK
PPPEHKPPVEKPPPEHKPPVEKPPPEHKPPVEKPPPEHKPPVEKPPPEHKPPVEKPPPEH
KPPVEKPPPEHKPPVEKPPPEHKPPGGRSHLLNTSLQWRSHHPEHKPSGWRSHLLN

>TA|CF201982||Vitaceae_V.hybrid||p3ehk1-containing
MSPTYLLVLFLGVVVLTTPSLADYPKPPPIKKPPPEHKPPVEKPPPEHKSPTKPPKGEKP
LPEHKPPTPIGKPPKGEKPPHYGHNPGHPPVESKEDSYKPPHKIEPPSAPMKKPEAPGKK
PSTPPHKPPHKPPSPSHPN

>TA|CK317675||Vitaceae_V.riparia||PEHK
MSPTYLLVVLLGLVVLTALSLADYPTHPPFEKPPPEHKPPVEKPPPEHKPPVEKPPPEHK
PPVEKPPPEHKPPVEKPPPEHKPPVEKPPPEHKPPVEKPPPEHKPPVEKPPPEHKPPVEK
PPPEHKPPVEKPPPEHKPPVEKPPPEHKPPVEKPPPEHKPPVEKPPPEHKPPVEKPPPEH
KPPVEKPPPEHKPPVEKPPPEHKPPVEKPPPEHKPPVEKPPPEHKPPVEKPLLN

>TA|TA2283_246827||Vitaceae_V.shuttleworthii||PEHK
STSLLVLLLGLVVLTTSSLADYPEHPPLQKPPTEHQPPTKPPNGEKPLPEHKPPSPFGKP
PQGEKPPPQHKPSDKTRKLLQVEKPLPEHKPPVKKPPTEQQPPTEPAKGENPVPEHKPPS
PFGKPPQGEKPPPEQKPSDKTRSLLQGEKPAVKKPPTEHQPPTEPAKGEKPLPEHKPPSP
FGKPPQGR

>NR|gi:225433075||Vitaceae_V.vinifera||PEHK
MSPTYLLVLFLGVVVLTPPSLADYPKPPPTKKPPPEHKPPVEKPPPEHKSPTKPPKGEKP
LPEHKPPTPIGKPPKGEKPLPEHKPPTPVGKPPKGEKPPHYGHNPGHPPVESKEDSYKPP
HKIEPPSAPKKPQAPEKKPPTPPHKPPHKPPSPSHPN

>NR|gi:225433086||Vitaceae_V.vinifera||PEHK
MSPTYLLVLFLGLVVLTTPSLADYPKPPPTKKPPPEHKPPVEKPPPEHKSPTKPPKGEKP
LPEHKPPTPIAKPPMGEKPLPEHKPPTPVGKPPKGEKPPHYDHNPGHPPVESKEDSYKPP
HKIEPPSAPMKKPEAPGKKPSTPPHKPPHKPPSPTHPN

>NR|gi:225433084||Vitaceae_V.vinifera||PEHK
MSTTYLLVLLLGMVVLTTPSLGDYPEHPPFEKPPPEHKPPVEKPPPEHKPPTKPPQGEKP
LPEHKPPTPIGKPPKGEKPPPGHKPTPFEKPPKGEKPLPEHKPPTPVGKPPKGEKPLPEN
KPPTPVGKPPKGEKPPHYGHNPGHPPAENAEDSYKPPRKIKPPSTPAKKQPAPGKKLPTS
PHKPPLKPPSPIHPN

>NR|gi:225433063||Vitaceae_V.vinifera||PEHK
MSSACSLVLFLGLVVLTTPSLANDHKPPPYEHKPPLPVYKSPPLGKPPPEYKPPTPVYKP
PPVEKPPTPVYRPPPVEKPPPEYKPPTPVYKPPPVEKPPPEYKPPTPVYKPPPVEKPPPE
YKPPTPVKPPPPPKHKTPTLPPRVVRPPPTPKPPTLPPIIVRPPPTKEPSPPQGHYPGHP
PVETPPSTPYKKPPTPEKKPWAPHHKHFKAPPPIHAN

>NR|gi:225433106||Vitaceae_V.vinifera||PEHK
MSSTSLLVLLLGLVVLTTSSLADYPKHPPLQKPPTEHQPPIKPPNGEKPLPEHKPPSPFG
KPPQGEKPPPQHKPSDKTRKLLQVEKPLPEHKPPVKEPPTEHQPPNEPPKGEKPVPEHKP
PSPFGKPPQGEKPPPEQKPSDKTRNLLQGEKPLPEHKPPSPFGKPPQGEKPPPEHKPSDK
TRNLLEGEKQLPEHKPPSPLGKPPKGQKPPPSGHNPGHPPAESAEDSYKPPQKITPPSTP
TKKPPAPTQKPPHKPPSPTHPN

>NR|gi:7406661||Vitaceae_V.vinifera||PEHK
MSPTCLLVLLLGMVVLTSTPSLAHYPKPPPFQKPPPEHKPPGEKPSPEHKPPTKPPKGEK
PLPEHKPLTPIGKPPKGEKPPPEHKPPTPIDKPPKGKKPLPEHKPPTPIGKPPKGEKPPP
EHKPPTPIGKPSKGEKPLPEHKPPTPIGKLPKGEKPPPEHKPPTPISKPPKGEKPLPEHK
PPTPIGKPPKGEKPLPEHKPPTPIGKPPKGEKPLPEHKPPTPIGKPPKGEKPPSPFGKPP
TGEKPPHYGHTPGHPPAHSYKPPQKIKPPPTPPHKPPHKPPSPTYPN

>NR|gi:225433073||Vitaceae_V.vinifera||PEHK
MSPTYLLVVLLGLVVLTAPSLADYPTHPPFEKPPPEHKPPVEKPPPEHKPPVEKPPPEHK
PPVEKPPPEHKPPVEKPPPEHKPPVEKPPPEHKPPVEKPPPEHKPPVEKPPPEHKPPVEK
PPPEHKPPVEKPPPEHKPPVEKPPPEHKPPVEKPPPEHKPPVEKPPPEHKPPVEKPPPLH
KPPVEKPPPEHKPPVEKPPPEHKPPVEKPPPEHKPPVEKPPPEHKPPVEKPPPEHKPPVE
KPPPEHKEKPLPEHKPPTPVGKPPKGEKPPHCGHNPGHPPAENAEDSYKPPRKIKPPSTP
AEKQPGPGKKLPTPPHKPPHKPPTPTHPN

>NR|gi:44888879||Vitaceae_V.vinifera||PEHK
MSSTCLLVLLLGLVVLTTSSLADYPKHPPLQKPPTEHQSPTKPPNGEKPLPEHKPPTPFG
KPPQGEKPPPQHKPSDKTRKLLQVEKPLPEHKPPVKKPPTEQQPPTEPPKGEKPVPEHKP
PSPFGKPPQGEKPPPEQKPSDKTRNLLQGEKPLPEHKPPSPFGKPPQGEKPPPEHKPSDK
TRNLLQGEKPLPEHKPPSPFGKPPQGEKPPPEQKPYDKTRNLLQGEKPLPEHKPPSPFGK
PPQGEKPPPEQKPSDKTRNLLQGEKPLPEHKPPSPFGKPPQGEKPPPEHKPSDKTRNLLE
GEKQLPEHKPPSSLGKPPKGQKPPAPTQKPSHKPPSPTHPN

>chr5:7812099,7812947||Vitaceae_V.vinifera||PREDICTED

MSSTSLLVLLLGLVVLTTSSLADYPKHPPLQKPPTEHQPPTKPPNGEKPLPEHKPPSPFG

KPPQGEKPPPQHKPSDKTRKLLQVEKPLPEHKPPVKKPPTEHQPLTEPAKGEKPVPEHKP

PSPFGKPPQGEKPPPEQKPSDKTRNLLQGEKPAVKKPPTEHQPPTEPAKGEKPLPEHKPP

SPFGKPPQGEKPPPEHKPSDKTRNLLEGEKQLPEHKPPSPLGKPPKGQKPPPSGHNPGHP

PAESAKDSYKPPQKITPPSTPTKKPPAPTQKPPHKPPSPTHPN

>chr5:7797145,7797930||Vitaceae_V.vinifera||PREDICTED

MSSTSLLVLLLGLVVLTTSSIADYPKHPPLQKPPTQHQPPTKPPHGEKPLPEHKPPSPFG

KPPPGEKPPPQHKPSDKTRKLLQVEKPLPEHKPPVKEPPTEHQPPNEPPKGEKPVPEHKP

PSPFGKPPQGEKPPPEQKPSDKTRNLLQGEKPLPEHKPPSPFGKPPQGEKPPPEHKPSDK

TRNLLEGEKQLPEHKPPSPLGKPPKGQKPPPSGHNPGHPPAESAEESYKPPQKNMPPSTP

TKKPPAPTQKPPHKPPSPTHPN

>chr5:7762548,7763171||Vitaceae_V.vinifera||PREDICTED

MSPTYLLVVLLGLVVLTAPSLADYPQHPPFKKPPPEHKPPVEKPPPEHKPPVEKPPPEHK

PPVEKPPPEHKPPVEKPPPEHKPTPLEKPPKGEKPLPEHKPPTPVGKPPKGEKPPHYGHN

PGHPPAENAEDSYKPPTPVGKPPKGEKPPHYGHNPGHPPAENAEDSYKPPRKIKPPSTPA

EKQPGPGKKLPTPPHKPPHKPPTPTHPN

>chr5:7815994,7816560||Vitaceae_V.vinifera||PREDICTED

MSSTYLLVLLLGLVVLTTPSLADYPKHPPVHKPPTEHQPPVEKPPTEHQPPTKPPKGEEP

LPEHKPPSPFGKPPQGEKPPPEHKPSDKTRKLLGGEKPLPEHKPASPFGKPPKGEKPLPE

HKPASPLGKPPKGEKPPHYGHNPGHPLAESAEDSYKPPQTIKPPSTPTKKPPVPGKKPPH

KPPSPTHPN

>chr5:7654010,7654600||Vitaceae_V.vinifera||PREDICTED

MSPTYLLVLLLGMVVLTNPSLADYPEHPPFEKPPPEHKPPVEKPPPEHKPPVEKPPPEHK

PPVEKPPPEHKPTPLEKPPKGEKPLPEHKPPTPVGKPPKGEKPPHYGRNPGHPPAENAED

SYKPPTPVGKPPKGEKPPHYGHNPGHPPAENAEDSYKPPRKIKPPSTPAEKQPGPGKKLP

TPPHKPPHKPPTPTHPN

>chr5:7697710,7698228||Vitaceae_V.vinifera||PREDICTED

MSPTYLLVVLLGLVVLTAPSLADYPKHPPFEKPPPEHKPPVEKPPPEHKPPVEKPPPEHK

PPVEKPPPEHKPPVEKPPPEHKPTPLEKPPKGEKPLPEHKPPTPVGKPPKGEKPPHYGHN

PGHPPAENAEDSYKPPTPVGKPPKGEKPPGPGKKLPTPPHKPPHKPPTPTHPN

>chr5:7713410,7713913||Vitaceae_V.vinifera||PREDICTED

MSPTYLLVLFLGVVVLTIPSLADYPKPPPIKKPPPEHKPPVEKPPPEHKPPVEKPPPEHK

SPTKPPKGEKPLPEHKPPTPIGKPPKGEKPLPEHKPPTPVGKPPKGEKPPHYGHNPGHPP

VESKEDSYKPPHKIEPPSAPKKPQAPEKKPPTPPHKPPHKPPSPSHPN

>chr5:7756575,7757165||Vitaceae_V.vinifera||PREDICTED

MSPTYLLVVLLGLVVLTAPSLADYPKHPPFEKPPPEHKPPVEKPPPEHKPPVEKPPPEHK

PPVEKPPPEHKPTPLEKPPKGEKPLPEHKPPTPVGKPPKGEKPPHYGHNPGHPPAENAED

SYKPPTPVGKPPKGEKPPHYGHNPGHPPAENAEDSYKPPRKIKPPSTPAEKQPGPGKKLP

TPPHKPPHKPPTPTHPN

>chr5:7708325,7708741||Vitaceae_V.vinifera||PREDICTED

MSPTYLLVLFLGLVVLTTPSLADYPKPPPTKKPPPEHKPSVEKPPPEHKSPTKPPKGEKP

LPEHKPPTPIGKPPKGEKPPHYGHNPGHPPFESKEDSYKPPHKIEPPSAPMKKPEAPGKK

PSTPPHKPPHKPPSPSHPN

>chr5:7603555,7603971||Vitaceae_V.vinifera||PREDICTED

MSSTYLLVLLLGVVVLTTSSLADYPKRPPVERPPIEHKPPTPIGKPPGGVKPPPKHKPPP

GHGPPTSVGKPPEEEEPLSGHKPPIPVGKPPKGHKPGHPPVENAEEDSHKMPPKMMPPKI

KPPPKKKPPHKPPTPGYPN

**@ PHEK Class [6 Proteins, 5 Species]**

>NR|gi:255647698||Fabaceae_G.max||PHEK
MTCVHSLVLFLFGVVILTTPVLANFFPPIYEPPPIEKPPIYEPPPSYEPPPTENPPPLYK
PPFYPPPVYQPPYEKPPPVYQPPYEKPPPVYQPPYEKPPPVYQPPYEKPPPVYQPPNEKP
PPEYQPPIYNPPPYGHYPPSKKK

>NR|gi:128421||Fabaceae_G.max||PHEK
MTSVLHYSLLLLLLGVVILTTPVLANLKPRFFYEPPPIEKPPTYEPPPFYKPPYYPPPVH
HPPPEYQPPHEKTPPEYLPPPHEKPPPEYLPPHEKPPPEYQPPHEKPPHENPPPEHQPPH
EKPPEHQPPHEKPPPEYEPPHEKPPPEYQPPHEKPPPEYQPPHEKPPPEYQPPHEKPPPE
HQPPHEKPPEHQPPHEKPPPEYQPPHEKPPPEYQPPQEKPPHEKPPPEYQPPHEKPPPEH
QPPHEKPPPVYPPPYEKPPPVYEPPYEKPPPVVYPPPHEKPPIYEPPPLEKPPVYNPPPY
GRYPPSKKN

>NR|gi:1754989||Fabaceae_L.luteus||PHEK
MPSLHSLIFLLLLGVVITPPVLANYKQPTYEPPTPIFEPPPFETLPPVYEPPPVYHPPRE
KPSIENPPPSYEKPPPHEKPPHEKSPPEYSPPHEEPTPEFPPSHEKSPPLYPSPHEKPPI
ECPPPHDKLPPIYEPPNEKPPPLHPPPHEKPPIDYPPPHEKPSPVYEPPYEKPPILHPPP
HEKPPPLYESPSEKPPHVHPPPPHKKPPIGYPPSYEKPPPVYEPPYEKSPPVHQPPYETP
PIENPPHEKPPIHESPYEKPPPVHPPPHDKPSIEYPPPHEKPPVYEPPYDKPPPVHPPPH
EKPPIEYPPPHEKPPVHEPPYEKPPPEHSPPHDKPPIEYHPPHEKPPIEYPPPHEKPPIE
YPPPHEKPPIEYPPPHEKPSIEYPPPHEKSPVYEPPYEKSPPVHPPPDEKPPIEYPPPHE
KPPVHEPPYKKPPPHDKPPIEYPPPHEKPPVYEPPYEKSPPVHPPPHEKPPIVHPPPHEK
PPLFEPPFEKPPPVHPPPVHPPPHEKPPIEYPPPHTKPPIEYPPPHVKPPIQYPPPHEKP
PIEYPLPHEKPPVYEPPYEKPPPVHPPPHEKPPIEYSPPHEKPPVHEPPYEKPPLVHPPP
HDKPPIEYHPPHEKPPIEYPPPHEKPPIEYPPPHEKPPIEYPPPHEKPPVYEPPYEKPPP
VHPPPDEKPPIEYPPPLEKPPVHEPPYEKPPPVQPPPHDKPPIEYPPPHEKPPVYEPPYE
RSPPVHPPSHEKPPFVYPPPHEKSPMHEPPYEKAPPVHLPPHEKPPIEYAPPHEKPPVEY
PPPHVKPPVEYPSPAEKPPTHEKPPIEFPPHEKPPVYEPPYEKPPSVCPPPHEKPPHHEK
RSALYPPHHRKPPNHHKPLPYKKPPFYKSHQWKSHQATSPNLMPSSTSFQKNDE

>NR|gi:2773251||Fabaceae_M.amurensis||PHEK
SLVLLLLGVVVTTPVLANYKPPIYEPPPTEKPPTYEPPEIEKPPPVYQPPPVHYPPPHVK
PPPEYEPPPEYQPPPEYQPPHEKPPPEYQPPHEKPPPEYQPPHEKPPPEYPPPHEKPPPE
FPPPYEKPPPEYQPPYEKPPPLYPPPHEKPPIEYPPPHEKPPPVYQPPYEKPPIEYPPPH
EKPPPVYQPPYEKPPPVYPPPHEKPPIEYPPPHEKLPPVYQPPYEKPPPTYPPPHEKPPI
EYPPPHEKPPYEKPPPEYQPPYEKPPPLYPPPHEKPPIEYPPPHEKPPPVYQPPYEKPPI
EYPPPHEKPPPVYQPPYEKPPPVYPPPHEKPPIEYPPPHEKLPPVYQPPYEKPPPTYPPP
HEKPPIEYPPPHEKPPYEKPPPLYPPPYEKPPPLYPPPHHHKPPHHEKPPFYKPPVYEPP
PLEKPPPVEKPPSYKPPPYRHYPPSKN

>TC|TC137258||Fabaceae_M.truncatula||PHEK||ENOD2 (partial)
MASMHSLAILLLGVVMLTTPVLAEYYKPPTYEPPIEKPPIYEPPPTEEPPPVYKPPIIHP
PPNYKPPAHTPPIYHPPHEKPPPVYEPPYEKPPHEEPPREYQPPRENPSPEYEPPHHGKP
PYENPPPEYKPPYEKPPPEYQPPHHEKPPPEYQPPHEKPPPEYTPPYE

>NR|gi:21373||Fabaceae_S.rostrata||PHEK
MSSLHYSLVTLLLLGVVMLTTPVLANYYEPPPIEKPPTYEPPPTYEKPPPVYKPPIFPPP
YEKPPPVYSPPYEKPPPVYPPPYEKPPPVYPPPYEKPPPEYQPPHEKPPPEYQPPHENPP
PEYQPPHEKPPPEYQPPHEKPPPEYQPPHEKPPPEYQPPHEKPPPEYQPPQEKPPPVYPP
PYEKPPHEKPPSYEKPPPYEKPPHEKPPYEKPPHEKPPHEKPPYDKPPYEKPPHEKPPHE
KPPHEKPPPEYKPPHEKPPPEYKPPHEKPPPYEKPPHEKPPPEYKPPHEKPPPPEYPPYV
KPPPEYKPPHEKPPGYNPPPYGHYPPSKKN

**@ QRA Class [112 Proteins, 43 Species]**

>TC|CV144624||Pinaceae_P.taeda||QRA
MKTLLILTILAMAITIGTANIQVDPSGQVQWLQQQLVPQLQQPLSQQPQQTFPQPQQTFP
HQPQQQVPQPQQPQQPFLQPQQPFPQQPQQPFPQTQQPQQPFPQQPQQPFPQTQQPQQPF
PQQPQQPFPQTQQPQQPFPQLQQPQQPFPQPQQQLPQPQQPQQSFPQQQRPFI

>NR|gi:209971947||Poaceae_A.bicornis||QRA
MKTLLTLTILAMATTIATANMQIDPSSQVQWPQQQPLPQPQQPFCQQPQQTIPQPHQTFP
HQPQQTFPQPQQTYPHQPQQQFPQTQQQQQPFPQPQQTFPQQPQLSFPQQPQQPFPEPQQ
PQQQFPQSQQPQQPFPQTQQQFPQPQQPQQSFPQQQQPLIQLSLQQQMNPCKNFLLQQCN
PVSLVSSLMSMILPRSDCQVMQQQCCQQLAQIPQQLQCAAIHSVAHSIIMQQEQQQGVQI
LRPLFQLAQGLGIIQPQQPAQLEGIRSLVLKTLPTMCNVYVSPDCSTINAPFASIVVGIG
GQ

>NR|gi:209972039||Poaceae_A.bicornis||QRA
MKTLLILTILAMTVTIGTANMQVDPSGQVQWTQQQPVPLPQQPFSQQPQQTFPQPQQTFP
HQPQQQIPQPQQPQQQFLQPQQPFPQQPQQPYPQQPQQPFPQTQQPQQLFPQSQQPQQPY
PQQPQQPFPQTQQPQQQFPQSQQPQQPFPQPQQPQQSFPQQQPPFIQPSLQQQLNPCKNL
LLQQCRPVSLVSSLWSMIWPQSDCQVMRQQCCQQLAQIPQQLQCAAIHSVVHSIIMQQEQ
QQQQQQQQQQQQGLRIPLPLYRQQQVGQGTLVQGQGIIQPQQPAQLEAIRSLVLQTLPTM
CNVYVPPECSIIKAPFASIVTGIGGQ

>NR|gi:183229578||Poaceae_A.columnaris||QRA
MKNFLVFALLAVVATSTIAQMETSCIPGLERPWQQQPLPPQQSFSQQPPFPQQQQPLPQQ
PSISQQEPPFLQQQGPPFSQQQQPVLPQQSPFSQQQQLVLPPQQQQQQLVQQQIPIVQPS
ILQQLNPCKVFLQQQCSPIAMPQRLARSQMWQQSSCHVMQQQCCQQLQQIPEQSRYEAIR
AIIYSIILQEQQQDFVQPQRQQPQQSGQSVSQSQQQSQQQLGQCSFQQPQQQLGQQPQQQ
QQQQVLQGTFLQPHQIAHLEVMTSIALRTLPSMRSVNVPLYSSTTSVPFGVGTGVGAY

>NR|gi:182676648||Poaceae_A.comosa||QRA
MKTFLVFALLAVAATSAIAQISQQQQPPFSQQQQPSLLQQQQPSLLQQQQPSLLQQQQPP
FSQQQQPQFSQQQQPQFSQQQQSPFSQQQQLPFSQQQQPPFSHQQQLPFLQQQQQPFSHQ
QQIPVIQPSILQQLNPCKVFLQQQCIPVAMQRCLARSQMLQQSSCHVMQQQCCQQLRQIP
QQSRHESIRAIIYSIILQQQQQQQQQQQQGQSFIQPQQQQPQQLGQCVSQPQQQSQQQLG
QQPQQQQLAQGTFLQPHQIAQLEVMTSIALRTLPTMCSVNVPLYGTTTSVPFGVGTGVGA
Y

>NR|gi:182676654||Poaceae_A.comosa||QRA
MKTLLIFALLVVAATSAIAQMETSCIPGLERPWQQQLLPPQQTFPQQPLFSQQQQQPFPQ
QPSFSQQQPPFWQQQPPFSQQQPILPQQPPFSQQQQPVQPQLPPFSQQQQLVLPPQQPPF
PQQQQQQQQQLVQQQIPVVQPSILQQLNPCKVFLQQQCSPVAMPQRLARSQMLQQSSCHV
MQQQCCQQLPQIPQQSRYEAIRAIIYSIILQEQQQVQGSIQPQQQQPQQLGQCVSQPQQQ
LQQQLGQQPQQQQLAQGTFLQPHQIAQLEVMTSIALRILPTMCSVNVPLYRTTTSVPFGV
GTGVGAY

>NR|gi:182676656||Poaceae_A.comosa||QRA
MKTLLIFALLAVAATSAIAQMETSCIPGLERPWQQQPLPPQQTFPQQPLFSQQQQQPFPQ
QPSFSQQQPPFWQQQPPFSQQQPILPQQPPFSQQQQPVQPQQPPFSQQQQLVLPPQQPPF
PQQQQQQQQQQLVQQQIPVVQPSILQQLNPCKVFLQQQCSPVAMPQRLARSQMLQQSSCH
VMQQQCCQQLPQIPQQSRYEAIRAIIYSIILQEQQQVQGSIQPQQQQPQQLGQCVSQPQQ
QLQQQLGQQPQQQQLAQGTFLQPHQIAQLEVMTSIALRILPTMCSVNVPLYRTSTSVPFG
VGTGVGAY

>NR|gi:182676642||Poaceae_A.comosa||QRA
MKTVLVCALLAIAATSAIAQLPISQQQQPPFSQQQQPPFSQQPQISQQQLQQQQQQPLPQ
QEQAPFLQQKQPPFSQQQQPPFSQQQQPPISQRPQISQQQQPPCSQQQQPPFSQQQPSFS
QQQQPQISQQQQPPFSQQQQIPVIHPSILQQLNPCKVFLQQQCSPVAMQRGLARSQMLQH
GSCHVLRQQCCQQLPQIPEQSRYEAIRAIVYPIILQEQQQSQGFIQPQQQQPQQSAQCVS
QPQQQSHQQLGQQPEQQQLGQQPQQQQQQQQRQQQQVLLGTFLQPHQIAQLEAMTSIALR
TLPRMCSVNVPLYGTASSVSFGVGTGVGAY

>NR|gi:182676644||Poaceae_A.comosa||QRA
MKTVLVCALLAIAATSAIAQLPISQQQQPPFSQQQQPPFSQQPQISQQQLQQQQQQPLPQ
QEQAPFLQQKQPPFSQQQQPPFSQQQQPPISQQPQISQQQQPPCSQQQQPPFSQQQPSFS
QQQQPQISQQQQPPFSQQQQIPVIHPSILQQLNPCKVFLQQQCSPVAMQRGLARSQMLQH
GSCHVLRQQCCQQLPQIPGQSRYEAIRAIVYSIILQEQQQGQGFIQPQQQQPQQSAQCVS
QPQQQSHQQLGQQPEQQQLGQQPQQQQQQQQQQQQQVLLGTFLQPHQIAQLEAMTSIALR
TLPRMCSVNVPLYGTASSVSFGVGTGVGAY

>NR|gi:182676640||Poaceae_A.comosa||QRA
MKTFLVCALLAIAATSAIAQLPISQQQQPPFSQQQQPPSSQQPQISQQQLQQQQQPLPQQ
EQAPFLQQQQPPFSQQQQPPFSQQQQPPISQQPQISQQQQPPCSQQQQPPFSQQQPSFSQ
QQQPQISQQQQPPFSQQQQIPVIHPSILQQLNPCKVFLQQQCIHVAMQRGLARSQMLQHG
SCHVLRQQCCQQLPQIPEQSRYEAIRAIVYSVILQEQQQSQGFIQPQQQQPQQSAQCVSQ
PQQQSHQQLGQQPEQQQLGQQPQQQQQQQQQQQQQQQQQVLLGTFLQPHQIAQLEAMTSI
ALRTLPRMSSVNVPLYGTASSVSFGVGTGVGAY

>NR|gi:182676638||Poaceae_A.comosa||QRA
MKTFLVCALLAIAATSAIAQLPISQQQQPPFSQQQQPPFSQQPQISQQQLQQQQQPLPQQ
EQAPFLQQQQPPFSQQQQPPFSQQQQPPISQQPQTSQQQQPPCSQQQQPPFSQQQPSFSQ
QQQPQISQQQQPPFSQQQQIPVIHPSILQQLNPCKVFLQQQCIHVAMQRGLARSQMLQHG
SCHVLRQQCCQQLPQIPEQSRYEAIRAIVYSIILQEQQQSQGFIQPQQQQPQQSAQCVSQ
PQQQSHQQLGQQPEQQQLGQQPQQQQQQQQQQQQLQQQQQVLLGTFLQPHQIAQLEAMTS
IALRTLPRMCGVNVPLYGTASSVSFGVGTGVGAY

>NR|gi:182676646||Poaceae_A.comosa||QRA
MKTFLVCALLAIAATSAIAQIPISQQQQPPFSQQQQPPLSQQPQISQQQQQPLLSQQEQA
PFLQQQQLPFSQQQQPPFSQQQQPPFSQQQQPPISQQPQILQQQQPPFSQQQQPPCSQQQ
QPPFSQQQTPFSQQQQPQISQQQQPPFSQQQQILVIHPSILQQLNPCKVFLQQQCSPVAM
QRGLARSQMLQHGSCHVLRQQCCQQLPQIPEQSRYEAIRAIVYSIILQEQQQGQGFIQPQ
QQQPQQSAQCVSQPQQQSQQQLGQQPEQQQLGQQPTQLQQVLLGTFLQPHQIAQLEAMTS
IALRTLPRMCSVNVPLYGTASRVSFGVGTGVGAY

>NR|gi:256862028||Poaceae_A.cylindrica||QRA
MKTFLIFALLAVAATSAIAQMETSHIPGLEKPSQQQPLPLQQILWYHQQQPIQQQPQPFP
QQPPCSQQQQPPLSQQQQPPFSQQQPPFSQQELPILPQQPPFSQQQQPQFSQQQQPFPQQ
QQPLLLQQPPFSQQRPPFSQQQQQPVLPQQPPFSQQQQQQPVLPQQPILPQQPPFSQHQQ
PVLPQQQIPYVQPSILQQLNPCKVFLQQQCSPVAMPQSLARSQMLWQSSCHVMQQQCCQQ
LPRIPEQSRYDAIRAIIYSIVLQEQQHGQGFNQPQQQQPQQSVQGVSQPQQQQKQLGQCS
FQRPQQQQLGQWPQQQQVPQGTLLQPHQIAQLELMTSIALRTLPMMCSVNVPVYGTTTSV
PFGVGTQVGAY

>NR|gi:122831072||Poaceae_A.geniculata||QRA
MKNFLVFALLAVVATSTIAQMETSCIPGLERPWQQQPLPPQQSFSQQPPFPQQQQPLPQQ
PSISQQEPPFLQQQGPPFSQHQQPVLPQQTPFSQQQQLVLPPQQQQQQLVQQQIPIVQPS
ILQQLNPCKVFLQQQCSPIAMPQRLARSQMWQQSSCHVMQQQCCQQLQQIPEQSRYEAIR
AIIYSIILQEQQQGFVQPQRQQPQQSGQSVSQSQQQSQQQLGQCSFQQPQQQLGQQPQQQ
QQQQVLQGTFLQPHQIAHLEVMTSIALRTLPTMCSVNVPLYSSTTSVPFGVGTGFGAY

>NR|gi:122831074||Poaceae_A.geniculata||QRA
MKNFLVFALLAVVATSTIAQMETSCIPGLERPWQQQPLPPQQSFSQQPPFPQQQQPLPQQ
PSISQQEPPFLQQQGPPFSQQQQPVLPQQTPFSQQQQLVLPPQQQQQQLVQQQIPIVQPS
ILQQLNPCKVFLQQQCSPIAMPQRLARSQMWQQSSCHVMQQQCCQQLQQIPEQSRYEAIR
AIIYSIILQEQQQGFVQPQRQQPQQSGQSVSQSQQQSQQQLGQCSFQQPQQQLGQQPQQQ
QQQQVLQGTFLQPHQIAHLEVMTSIALRTLPTMCSVNVPLYSSTTSVPFGVGTGVGAY

>NR|gi:147743928||Poaceae_A.geniculata||QRA
MKTLLVFALLTVAATSAIAQMETSCIPSLERPWQQQPLPPQQTFPQQPLFSQQQQQPFPQ
QSSFSQQQPPFWQQQPPFSQQQPILPQQPPFSQQQQPVQPQQPPFSQQQQLVLPPQQQHQ
QLVQHQIPVVQPSILQQLNPCKVFLQQQCSPVAMPQRLARSQMLQQSSCHVMQQQCCQQL
PQIPQQSRCEAIRAIIYSIILQEQQQVQGSIQAQQQQPQQLGQCVSQPQRQLQQQLEQQP
QQQQLAQGTFLQPHQIAQVEVMTSIALRILPTMCSVNVPLYRTTTSVPFGVGTGVGAY

>NR|gi:122831070||Poaceae_A.geniculata||QRA
MKNFLVFALLAVVATSTIAQMETSCIPGLERPWQQQPLPPQQSFSQQPPFPQQQQPLPQQ
PSISQQEPPFLQQQGPPFSQQQQPVLPQQSPFSQQQQLVLPLQQQQQLVQQQIPIVQPSI
LQQLNPCKVFLQQQCSPIAMPQRLARSQMWQQSSCHVMQQQCCQQLQQIPEQSRYEAIRA
IIYSIILQEQQRGFVQPQRQQPQQSGQSVSQSQQQSQQQLGQCSFQQPQQQLGQQPQQQQ
QQQQQVLQGTFLQPHQIAHLEVMTSIAPRTLPTMCSVNVPLYSSATSVPFGVGTGVGAY

>NR|gi:123204221||Poaceae_A.geniculata||QRA
MKTFLVFALIAVVATSAIAQMETSCISGLERPWQQQPLPPQQSFSQQPPFSQHQQQPLPQ
HPSFSQQQPPFSQQQPILSQQPPFSQQQQPVLPQQSPFSQQQQLVLPPQQQQQQLVQQQI
PIAQPSVLQQLNPCKVFLQQQCSPVAMPQRLARSQMWQQSSCHVMQQQCCQQLPQIPEQS
RYEAIRAIIYSIILQEQQQGFVQPQQQQPLQSGQGVSQSQQQSQQQLGQCSFQQPQQQLG
QQPQQQQQQVLQGTFLQPHQIAHLEAVTSIALRTLPTMCSVNVPLYSATTSVPLGVGIGV
GAY

>NR|gi:147743926||Poaceae_A.geniculata||QRA
MKTFLVFALLAVAATSAIAQISQQQQPPFSQQQQPSLLQQQQPPFSQQQQPPFSQQQQPP
FSQQQQQQFSQQQQPQFSHQQQSPLSQQQQLPFSQQQQPPFSHQQQPPFLQRQQLPFSHQ
QQIPVIQPSILQQLNPCKVFLQQQCIPVAMQRCLARSQMLQQSSCHVMQQQCCQQLRQIP
QQSRHESIRAIIYSIILQQQQQQQQQQQQQQQQQQQQQQQQQQQGQSFIQPQQQQPQQLG
QCVCQPQQQSQQQLGQQPQQQQLAQGTFLQPHQIAQLEVMTSIALRTLPTMCSVNVPLYG
TTTSVPFGIGTGVGAY

>NR|gi:147743916||Poaceae_A.juvenalis||QRA
MKTFLVFALLALAAASAVAQISQQQQQPPFSQQQQPPFLQHQQPPFSQQQQSPFSRQQEQ
QQQPPFSQQQPILPQQPPFSQQQQPVLPQQSPFSQQQQLVLPPQQQHQQLLQQQIPIVQP
SVLQQLNPCKVFLQQRCSPVAMPQRLARSQMWQQSSCHVMQQQCCQQLPQIPEQSRYEAI
RAIIYPIILQEQQQGFAQPQQQQPQQSGQGVSQSQQQSQQQLGQCSFQQPQQQLSQQPQQ
QQQQVLQGTFLQPHQIAHLEVMTSIALRTLPTMCSVNVPLYSSTTSVPFGVGTGVGAY

>NR|gi:209971963||Poaceae_A.longissima||QRA
MKTLLILTILAMAVTIGTANMQVDPSGQVQWPQQQPVLLPQQPFSQQPQQPFPQPQQPQK
SFPQQQPPFIQPSLQQQLNPCKNLLLQQCRPVSLVSSLWSMIWPQSDCQVMRQQCCQQLA
QIPQQLQCAAIHSVVHSIIMKQEQQQQQQQQQQQQGLRIPLPLYQQQQVGQGTLVQGQGI
IQPQQPAQLEAIRSLVLQTLPIMCNVYVPPECSIIKAPFASIVTGIGGQ

>NR|gi:209971953||Poaceae_A.longissima||QRA
MKTLLILTILAMAITIATANMQVDPSGQVQWPQQQPFPQPHQPFSQQPQQIFPQPQQTFP
HQPQQQFPQPQQPQQQFLQPQQPFPQQPQQPYPQQPQQPFPQIQQPQQPFPQSKQPQQPF
PQPQQQFPQPQQPQQSFPQQQPSLIQQSLQQQLNPCKNFLLQQCKPVSLVSSLWSIILPR
SDCQVMRQQCCQQLAQIPQQLQCAAIHSVVHSIIMQQEQQEQLQSGQILVPLSQQQQVGQ
GILVQGQGIIQPQQPAQLEVIRSLVLQTLPTMRNMYVPPYCSTIRAPFASIVAGIGGQ

>NR|gi:122831076||Poaceae_A.longissima||QRA
MKTFLVFALLAVVATSAIAQMDTSCIPSLERPWQQQPLPPQQAFPQQPPFSQQQLFPQQP
SFSQQQPPFSQQQPILPQRPPFSQQQQPVLPQQLPFSKQQQLVLPPQQQHQQLLQQQIPI
VHPSILQQLNPCKVFLQQQCSPVAMPQRLARSQMWQQSSCHVIQQQCCQQLPQIPEQSRY
EAIRAIIYSIILQEHQQGFVQPQQQQPQQSGQGVSQSQQQSQQQLGQCSFQQPQQQLGQQ
PQQQQVLQSTFLQPHQIAHLEVMTSIALRTLPTMCSVNVPLYSSTTRVPFGVGTGVGAY

>NR|gi:122831078||Poaceae_A.longissima||QRA
MKTFLIFALLAVAATSAIAQMETNHIPSLEKSLQQQPLPLQQILWYHQQQPIQQQPIQQQ
PQPFPQQPPCSQQQQPLLSQQQQPPFSQQQPPFSQQQQPVLPQQPPVSQQQQPPVSQQQQ
PPFSQQQQQFPQQQQPLLPQQPPFSQQQPPFSQQQQQPVLPQQQIPSVQPSILQQLNPCK
VFLQQQCSPVAIPQSLARSQMLWQSSCHVMQQQCCQQLPQIPEQSRYDAVRAIIYSIVLQ
EQQHGQGLNQPQQQQPQQSVQGVSQPQQQQKQLGQCSFQQPQQQQLGQWPQQQQVPQGTL
LQPHQIAQLEVMTSIALRTLPTMCSVNVPVYGTTTIVPFGVGTRVGAY

>NR|gi:163860186||Poaceae_A.markgrafii||QRA
MKNFLVFALLAVVATSTIAQMETSCIPGLERPWQQQPLPPQQSFSQQPPFPQQQQPLPQQ
PSISQQQPPFLQQQGPPFSQQQQPVLPQQSPFSQQQQLVLPPQQQQQQQLVQQQIPIVQP
SILQQLNPCKVFLQQQCSPIAMPQRLARSQMWQQSSCHVMQQQCCQQLQQIPEQSRYEAI
RAIIYSIVLQEQQQGFVQPQQQQPQQSGQSVSQSQQQSQQQLGQCSFQQPQQQLGQQPQQ
QQQQQVLQGTFLQPHQIVHLEVMTSIALRTLPTMCSVNVPLYSSTTSVPFGVGTGVGAY

>NR|gi:256862034||Poaceae_A.markgrafii||QRA
MKTFLVFALLAIAATSAIAQMETSRVPGLEKPWQQQPLPPQQQPPCSQQQQPFPQQQQPI
IILQQSPFSQQQQPVLPQQQPVIILQQPPFSQQQQPVLPQQPPFSQQQQQQQQQQQQQPP
FSQQQQQQQPVLPQQPPFSQQQQPPFSQQQQPVLPQQPSSQQPPFPQQHQQFPQQQIPVV
QPSVLQQLNPCKVFLQQQCSHVAMSQRLARSQMWQQSSCHVMQQQCCQQLPQIPEQSRSE
AIRAIVYSIILQEQQQGFVQPQQQQPQQSGQGVSQHQQQSQQQQQLGQCSFQQPQQLQQL
GQQPQQQQIPQGIFLQPHQISQLEVMTSIALRTLPTMCGVNVPLYSSTTIMPFSIGTGVG
GY

>NR|gi:147743920||Poaceae_A.neglecta||QRA
MKTFLVFALLAVVATSAIAQMETSCIPGLERPWQQQPLPPQQSFSQQPPFSQQQPFPQQP
SFSRQQPPFSQQQPILLQQPPFSQQEQPVLPQQSPFSQQQQLVLPPQQQHQQFVQQQIPI
IQPSILQQLNPCKVFLQEQCSPVAPQRLARSQMWQQGSCHVMQQQCCQQLPQIPEQSRYE
AIRAIIYSIVLQEQQQGFVQPQQQQPQQSSQGVSQSQQQSQQQLGQCSFQQPQQQLGQQP
QQQQQQVLQGTFLQPHQIAHLEVMTSIALRTLPRMCSVNVPLYSSTTSVPFGVGIGVGAY

>NR|gi:147743924||Poaceae_A.neglecta||QRA
MKTFLVFALLAVVATSAIAQMETSCIPGLERTWQQQPLPPQQSFSQQPPFSQQQPFPQQP
SFSRQQPPFSQQQPILLQQPPFSQQEQPVLPQQSPFSQQQQLVLPPQQQHQQFVQQQIPI
IQPSILQQLNPCKVFLQEQCSPVAPQRLARSQMWQQSSCHVMQQQCCQQLPQIPEQSRYE
AIRAIIYSIVLQEQQQGFVQPQQQQPQQSSQGVSQSQQQSQQQLGQCSFQQPQQQLGQQP
QQQQQQVLQGTFSQPHQIAHLEVMTSIALRTLPRMCSVNVPLYSSTTSVPFGVGIGVGAY

>NR|gi:147743918||Poaceae_A.neglecta||QRA
MKTFLVFALLAVVATSAIAQMETSCIPGLERPWQQQPLPPQQSFSQQPPFSQQQPFPQQP
SFSRQQPPFSQQQPILLQQPPFSQQEQPVLPQQSPFSQQQQLVLPPQQQHQQFVQQQIPI
IQPSILQQLNPCKVFLQEQCSPVAMPQRLARSQMLQQSSCHVMQQQCCQQLPQIPQQSRY
EAIRAIIYSIVLQEQQQGFVQPQQQQPQQSSQGVSQSQQQSQQQLRQCSFQQPQQQLGQQ
PQQQQQQVLQGTFLQPHQIAHLEVMTSIALRTLPRMCSVNVPLYSSTTSVPFGVGIGVGA
Y

>NR|gi:147743922||Poaceae_A.neglecta||QRA
MKTFLVFALLAVVATSAIAQMETSCIPGLERPWQQQPLPPQQSFSQQPPFSQQQPFPQQP
SFSRQQPPFSQQQPILLQQPPFSQQEQPVLPQQSPFSQQQQLVLPPQQQHQQFVQQQIPI
IQPSILQQLNPCKVFLQEQCSPVAPQRLARSQMWQQSSCHVMQQQCCQQLPQIPEQSRYE
AIRAIIYSIVLQEQQQGFVQPQQQQPQQSSQGVSQSQQQSQQQLRQCSFQQPQQQLGQQP
QQQQQQQVLQGTFLQPHQIAHLKVMTSIALRTLPRMCSVNVPLYSSTTSVPFGVGIGVGA
Y

>NR|gi:209972001||Poaceae_A.searsii||QRA
MKTLLILTILAMAITIATANMQVDPSGQVQWPHQQPFPQPHQPFSQQPQQIFPQPQQTFP
HQPQQQFPQTPQPQQQFLQPQQPFPQQPQQPYPQQPQQPFPQTQQPQQPFPQSKQPKQPF
PQPQQQFPQPQQPQQSFPQQQPSLIQQSLQQQLNPCKNFLLQQCKPVSLVSSLWSIILPR
SDCQVMRQQCCQQLAQIPQQLQCAAIHSVVHSIIMQQEQQEQLQGVQILVPLSQQQQVGQ
GILVQGQGIIQPQQPAQLEVIRSLVLQTLPTMCNVYVPPYCSTIRAPFASIVAGIGGQ

>NR|gi:209972003||Poaceae_A.searsii||QRA
MKTLLILTILAMATTIATANMQVDPSGQVQWPQQQPFPQPQQPFCQQPQQTIPQPHQTFH
HQPQQTFPQPQQTYPHQPQQQFPQTQQPQQPFPQPQQTFPQQPQLPFPQQPQQPFPQPQQ
PQQPSPQSQQPQQPFPQPQQQFPQPQQPQHSFPQQQQPVIQSFLQQQMNPCKNFLLQQCN
PVSLVSSLVSIILPRSDCQVMQQQCCQQLAQIPQQLQCAAIHSVAHSIIMQQEQQQGVSI
LRPLFQLAQGLGIIQPQQPAQLEGIRSLVLKTLPTMCNVYVPPDCSTINVPYASIDAGIG
GQ

>NR|gi:209972059||Poaceae_A.sharonensis||QRA
MKTLLILTILAMAITISTANMQVDPSGQVQWPQQQPIPLPQQPLSQQPQQAFPQPQQTFP
HQPQQQVPQPQQPQQPFLQPQQPFLQPQQPFPQQPQQPFPQQPQQPFPQTQQPQQPSPQT
QQPQQPFPQQPQQPFPQTQQPQQPFPQFQQPQQPFPQPQQQFPQPQQPQQSFPQQQRPFI
QPSLQQRLNPCKNILLQQCKPASLVSSLWSIIWPQSDCQVMRQQCCQQLAQIPQQLQCAA
IHSVVHSIIMQQQQQQQQQQQQQQQGVHILLPLSQQQQVGQGTLVQGQGIIQPQQPAQLE
AIRSLVLQTLPTMCNVYVPPECSIIRAPFSSIVAGIGGQ

>NR|gi:209972019||Poaceae_A.speltoides||QRA
MKTLLILTILAMATTIATANMQVDPSGQVQWPQQQPFPQPQQPFCQQPQQTIPQPHQTFP
HQPQQTSPQPQQTYPYQPQQQFPQTQQPQQPFPEPQQTFPQQPQLPFPQQPQQPLPQPQQ
PQQQFPQPQQPQQPFPQPQQQFTQPQQLQQSSPQQQQLLIQLSLQQQMNPCKNFLLQQCN
PVSLLSSIMSMILPRSDCQVMQQQCCQQLAQVPQQLQCAAIHSVVHSIIMQQEQRQGVQI
RRPLFQLVQGQGIIQPQQPAQLEVIRSLVLQTLPTMCNVYVSPDCSTINAPFASIVVGIG
GQ

>NR|gi:209972017||Poaceae_A.speltoides||QRA
MKTLLILTILAMAITISTANMQVDPSGQVQWPQQQLVPQLQQPLSQQPQQAFPQPQQTFP
HQPQQQVPQPQQPQQPFLQPQQAFPQQPQQPFPQTQQPQQPFPQQPQQPFPQTQQPQQLF
PQSQQPQQPYPQQPQQPFPQTQQPQQQFPQSQQPQQLFPQTQQPQQQFPQSQQPQQPFPQ
PQQQFPQPQQPQQSFPQQQRPFIQPSLQQRLNPCKNILLQQCEPASLVSSLWSIIWPQND
CQVMQQQCCQELAQIPQQLQCAAIHSVVHSIIMQQQQQQQQQQQQQQQGMHILLPLSQQQ
QVGQGTLVQGQGIIQPQQPAQLEAIRSLVMQTLPTMCNVYVPPECSMIRAPFASIVAGIG
GQ

>NR|gi:256862038||Poaceae_A.speltoides||QRA
MKTFLIFALLAVAATSAIAQMETSHILSLEKPLQQQPLPLQQILWYHQQQPIQQQPQPFP
QQPPCSQQQQPPLSQQQQPPFSQQQPPFSQQQQPPFSQQQQQFPQQQQQPPLPQQPPFSQ
QQPPLSQQQQQPVLPQQPPFSQQQQQQPVLPQQQQRPFWQQPILPQQPPFSQHQQPALPQ
QQIPSIQPSILQQLNPCKVFLQQQCSPVAMPQSLDRSQMLWQSSCHVMQQQCCRQLLQIP
EQSRYDAIRAIIYSIVLQEQQHGQGLNQPQQQQPQQSVQGVSQPQQQQKQLGQCSFQQPQ
QQQLGQWPQQQQVPQGTLLQPHQIAQLEVMTSIALRTLPTMCSVNVPVYGTTTIVPFGVG
TRVGAY

>NR|gi:148612407||Poaceae_A.tauschii||QRA
MKTFLILALLAIVATTATTAVRVPVPQLQPQNPSQQQPQEQVPLVQQQQFPGQQQPFPPQ
QPYAQPQPFPSQQPYPQPQPFPSQLPYPQPQPFPPQQPYPQPQPQYPQPQQPISQQQAQQ
AQQQQQQQQQQQQQQQQQQQQQQQQQQQQQQQQQQQQQQQQQQQQQQQQQQQQQQQQQQQ
QQQQILQQILQQQLIPCRDVVLQQHNIVHASSQVLQQSSYQLLQQLCCQQLWQIPEQSRC
QAIHNVVHAIILHQQQQQPSSQVSYQQPQQQYLSGQGSFEPSQQNPQAQGSVQPQQLPQF
EEIRNLALQTLPATCNVYIPPYCSTTIAPFGIFGTN

>NR|gi:209971773||Poaceae_A.tauschii||QRA
MKTLLILTILAMAITISTANMQVDPSGQVQWPQQQLVPQPQQPLYQQPQQAFPQPQQTFP
HQPQQQVPQPQQPQQPFLQPQQAFPQQPQQPFPQTQQQQQQPFPQQPQQPFPQTQQPQQQ
FPQQQQQPFPQTQQPQQPFPQQPQQPFPQTQQPQQPFPQFQQPQQPFRQPQQQLPQPQQP
QQSFPQQQRPFIQPSLQQRLNPCKNILLQQCKPASLVSSLWSIIWPQNDCQVMQQQCCQE
LAQIPQQLQCAAIHSVVHSIIMQQQQQQQQQQQQGMHILLPPSQQQQVGQGTLVQGQGII
QPQQPAQLEAIRSLVLQTLPTMCNVYVPPECSIIRAPFASIVAGIGGQ

>NR|gi:47717670||Poaceae_A.tauschii||QRA
MKTFLIFALLAVAATSAIAQMETSHIPGLEKPSQQQPLPLQQILWYHQQQPIQQQPQPFP
QQPPCSQQQQPPLSQQQQPPFSQQQPPFSQQELPILPQQPPFSQQQQPQFSQQQQPFPQQ
QQPLLLQQPPFSQQRPPFSQQQQQPVLPQQPPFLQQQQQQPILPQQPPFSQHQQPVLPQQ
QIPYVQPSILQQLNPCKVFLQQQCSPVAMPQSLARSQMLWQSSCHVMQQQCCQQLPRIPE
QSRYDAIRAIIYSIVLQEQQHGQGFNQPQQQQPQQSVQGVSQPQQQQKQLGQCSFQRPQQ
QQLGQWPQQQQVPQGTLLQPHQIAQLELMTSIALRTLPMMCSVNVPVYGTTTSVPFGVGT
QVGAY

>NR|gi:50313199||Poaceae_A.tauschii||QRA
MKTFLIFVLLAMAMKIATAARELNPSNKELQSPQQSFSHQQQPFPQQPYPQQPYPSQQPY
PSQQPFPTPQQQFPQQSQQPFTQPQQPTPLQPQQPFPQQPQQPQQPFPQPQQPFPWQPQQ
PFPQTQQSFPLQPQQPFPQQPQQPFPQPQLPFPQQSEQVIPQQPQQPFPLQPQQPFPQQP
QQPFPQPQQPIPVQPQQSFPQQSQQSQQPFAQPQQLFPELQQPIPQQPQQPFPLQPQQPF
PQQPQQPFPQQPQQSFPQQPQQPYPQQQQSFPQQPQQPFPPTTTKPFPQQPQQPFPLRPQ
QPFPQQPQQSQQSFLQPQPQQPQQPSILQPQQPLPQQPQQPFQQPQQQLSQQPEQTISQQ
PQQPFPQQPHQPQQPYPQQQPYGSSLTSIGGQ

>NR|gi:183229584||Poaceae_A.triuncialis||QRA
MKNFLVFALLAVVATSTIAQMETSCIPGLERPWQQQPLPPQQSFSQQPPFPQQQQPLPQQ
PSISQQEPPFLQQQGPPFSQQQQPVLPQQSPFSQQQQLVLPPQQQQQQLVQQQIPIVQPS
ILQQLNPCKVFLQQQCSPIAMPQRLARSQMWQQSSCHVMQQQCCQQLQQIPEQSRYEAIR
AIIYSIILQEQQQDFVQPQRQQPQQSGQSVSQSQQQSQQQLGQCSFQQPQQQLGQQPQQQ
QQQQVLQGTFLQPHQIAHLEVMTSIALRTLPSMCSVNVPLYSSTTSVPFGVGTGVGAY

>NR|gi:56387341||Poaceae_A.triuncialis||QRA
MKTFLVFALLAIAATSAIAQMETSRVPGLEKPWQQQPLPPQQQPPCSQQQQPFPQQQQPI
IILQQSPFSQQQQPVLPQQQPVIILQQPPFSQQQQPVLPQQPPFSQQQQQQQQQQQQQPP
FSQQQQPVLPQQPPFSQQQQPPFSQQQQPSSQQPPFPQQHQQFPQQQIPVVQPSVLQQLN
PCKVFLQQQCSHVAMSQRLARSQMWQQSSCHVMQQQCCQQLPQIPEQSRSEAIRAIVYSI
ILQEQQQGFVQPQQQQPQQSGQGVSQHQQQSQQQQQLGQCSFQQPQQLQQLGQQPQQQQI
PQGIFLQPHQISQLEVMTSIALRTLPTMCGVNVPLYSSTTIMPFSIGTGVGGY

>NR|gi:150023801||Poaceae_A.umbellulata||QRA
MKNFLVFALLAVVATSTIAQMETSCIPGLERPWQQQPLPPQQSFSQQPPFPQQQQPLPQQ
PSIPQQQPPFLQQQGPPFSQQQQPVLPQQSPFSQQQQLVLPPQQQQQLVQQQIPIVQPSI
LQQLNPCKVFLQQQCSPIAMPQRLARSQMWQQSSCHVMQQQCCQQLQQIPEQSRYEAIRA
IIYSIILQEQQQGFVRPQQQQPQQSGQSVSQSQQQSQQQLGQCSFQQPQQQLGQQPQQQQ
QQVLQGTFLQPHQIAHLEVMTSIALHTLPTMCSVNVPLYSSTTSVPFGVGTGVGAY

>NR|gi:183229588||Poaceae_A.uniaristata||QRA
MKTLLIFALLAVAATSAIAQMETSCIPGLERPWQQQPLPPQQTFPQQPLFSQQQQQPFPQ
QPSFSQQQPPFWQQQPPFSQQQPILPQQPPFSQQQQPVQPQQPPFSQQQQLVLPPQQPPF
PQQQQQQQQQQQQQLVQQQIPVVQPSILQQLNPCKVFLQQQCSPVAMPQRLARSQMLQQS
SCHVMQQQCCQQLPQIPQQSRYEAIRAIIYSIILQEQQQVQGSIQPQQQQPQQLGQCVSQ
PQQQLQQQLGQQPQQQQLAQGTFLQPHQIAQLEVMTSIALRILPTMCSVNVPLYRTTTSV
PFGVGTGVGAY

>NR|gi:247663679||Poaceae_A.ventricosa||QRA
FALIAVVATSAIAQMETSCISGLERPWQQQPLPPQQSFSQQPPFSQQQPLPQQPSFSQQQ
PPFSQQQPILSQQPPFSQQQQPVLPQQSPFSQQQQLVLPPQQQQQQQLVQQQIPIVQPSV
LQQLNPCKVFLQQQCSPVAMPQRLARSQMWQQSSCHVMQQQCCQQLQQIPEQSRYEAIRA
IIYSIILQEQQQGFVQPQQQQPQQSGQGVSQSQQQSQQQLGQCSFQQPQQ

>NR|gi:146160692||Poaceae_A.retrofractum||QRA
MKTFIILALLAIAATTATAVLRDPMSQLQPQNPSSQQPQLWFPGRQRQQFPGQQQPFPPQ
QPYPQPQPFPQPQPFPPQQPYPQPQPFPPQQPYPQLQQPISQQQGQQVPQQQTLQQFLQQ
QMIPCRAVLLQQRKVAPVRSQVLQQSSYQELQQQCCQQLWQIPEQSRCQAINSVVHAIIL
HQQLQQYPSGQGSIQQYPLSQGSFQQSQQQYPHGQGTVQPQQIPQFEEIRNLVLQTLPAL
CNVYVPPYCSTTTAPFGSTGSN

>NR|gi:158713839||Poaceae_A.retrofractum||QRA
MKTFIILALLAIVLTTATTAVRLPVPQLQPQNPSQQQPQEQIPVMQQQQFPGQQQPFPPQ
QPYPQPQPFPPQQPFPQPQPFPPQLPYPQPQPYPPQQSYPQPQQQYPQPQQPISQQQAQQ
EQQILQPFQQQQLIPCRDVVLQQHNIAHPSSQVWQQSSYQELQQLCCQQLWQIPVQSRCQ
AINSVVHAIILHQQQQQRQQQQQQQQLYPSSQGSFQQPQQYPSGQGSFRPTQQNPQGHSS
VQPQQLPQFEEIRNLALQTLPTMCNVYVPPYCSTTSTPFGSTGTN

>NR|gi:162424703||Poaceae_C.delileana||QRA
MKTFLILALLAIVATTATTAVRVPVPQLQPQNPSQQQPQEQVPLVQQLQFPGQQQPFPPQ
QPYPQPQPFPSQQPYPQPQPFPQPQPFPPQLPYQQPQPFPPQQPYPQLQQQYPQPQLPIS
LQQAQQQQQQQQQQQQILQQILQQQLIPCRDVILQQLNIAHPRSQVLQQLCCQQLWQTPE
QSRCQAIHNVVHAIILHQQQQQQQQQQRQPSSQVSYRQPQQQYPSGLGSFQPSQQNPQAQ
GSVLPQQLPQFEEIRNLALQTLPAMCNVYIPPYCSTTIAPFGIFGTN

>NR|gi:162424697||Poaceae_C.delileana||QRA
MKTFLVFALLAVVATSAIAQMETICNPGLERPWQQQPLPPQQTFPQQPPFSQQQPFPQQP
SFSQQQPPLSQQQQIQQPPFSQQQQPPFSQQQQPVLPPQQPPFPQQQHQQLLQQQIPVVQ
PSVLQQLNPCKVFLQQQCSPVAMPQRLPRSQMWQQSSCHVMQQQCCQQLPQIPEQSRYEA
IRAIIYSIILQEQQQGFVQPQQQQPQQSGQGVSQSQQQSQQQLRQCSFQQPQQQLGQHPQ
QQQVPQGTFLQPHQIAQLEVMTSIALRTLPTMCSVNVPLYSSTTSVPFRIVTGVGAY

>NR|gi:162424705||Poaceae_C.delileana||QRA
MKTLLILTILAMAITISTANMQVDPSGQVQWPQQQPVPQPQQPLSQQPQQPFPQPQQTFP
HQSQQEVPQPQQPQQPFLQPQQPFPQQPQQPFPQTQQPQQPFPQQPQQPFPQTQQPQQPF
PQFQEPQQPFPQPQQQFSQPQQPQQSFPQQQRPFIQPSLQQRLNPCKNIVLQQCKPVSLV
SSLWSIIWPQSDCQVMRQQCCQQLAQIPQQLQCAAIHSVVHSIIMQQQQQQQQQQQQQQQ
QGMHNLLPLSQQQQVGQGTLVQGQGIIQPQQPTQLEAIRSLVFQTLPTMCNVYVPPECSI
IRAPFASIVTGIGGQ

>NR|gi:163932257||Poaceae_D.breviaristatum||QRA
MKTFLILSLLAIVATTATTAVRVPVPQLQPQNPFQQQPQEQVPLMQQQEFPGQQQPIPPQ
QPYPQPQSFPSQQPYPQPQPFPPQQLFPQPQPFLPQLPYPQPQPFPPQQSYPQPQQQYPQ
QQQPILQQQEQQILQQLLQQRLNPCRDVVLQQHNIAHGNSQVLQQSSYQVLQQLCCQQLW
QIPEQSRCQAVHGVVHAIILHQQQQQQQQQLLSHGSFQQPQQQYPSGQGSFQPSQQNPQG
QSFVQPQQLPQFEEIRRLALQTLPTMCNVYVPTYCSTTIVPFGSISIN

>NR|gi:158344547||Poaceae_D.breviaristatum||QRA
MKTFIILSLLAIVATTATTAARVPVPQLQPQIPFQQQPQEQVPLMQQQEFPGQQQPIPPQ
QPYPQPQSFPSQQPYPQPQPFPPQQLFPQPQPFLPQLPYPQPQPFPPQQSYPQPQQQYPQ
QQQPILQQQEQQILQQLLQQRLNPCRDVVLQQHNIAHGNSQVLQQSSYQVLQQLCCQQLW
QIPKQSRCQAVHSVVHAIILHQQQQQQQQQQLLSQGSFQQPQQQYPSGQGSFQPSQQNPQ
GQSFVQPQQLPQFEEIRRLALQTLPTMCNVYVPTYCSTTIVPFGSIGIN

>NR|gi:163932247||Poaceae_D.breviaristatum||QRA
MKTFLILSLLAIVATTATTAVRVPVPQLQPQNPYQQQPQEQVPLMQQQQFPGQQQPIPPQ
QPYPQPQPFPSQQPYPQPQPFSPQQLFPQPQPFPPQLPYPQPEPFPPQQSYPQPQQQYTQ
QQQLILQQQEQQILQQQLSPCRDVVLQQHNIAHGSSQVLQQSSYQVLQQLCCQQLWQIPE
QSRCQAIHSVVQAIILHQQQQQQQQQQQQQQQQQQQQQQQLLSQGSFQQPQQQYPSGQGS
FQSSQQNPQGQSFIQPQQLPQFEEIRRLALQTLPAMCNVYVPTYCSTTIVPFGSIGIN

>NR|gi:163932229||Poaceae_D.villosum||QRA
MKTFLILSLLAIVATTATTAVRVPVPQLQPQNPYQQQSQEQVPLMQQQQFPGQQQPIPPQ
QPYPQPQPFPSQQPYPQPQPFPPQQLFPQPRPFLPQLTYPQPQQQYLQQQQPILQQQEQQ
ILQQLLQQRLNPCMDVVLQQHNIAHGSSQVLTQSSYQVLQQLCCQQLWQIPEQSRCQAVH
SVVHAIILHQQQQQQQQQQQQQQQQQQQQQQQQLLSQGSFQQPQQQYLSGQGSFQPSQQN
PQGQSFVQPQQLPQFEEIRRLALQTLPAMCNVYVPTYCSTTIVPFGSIGIN

>NR|gi:240000198||Poaceae_D.villosum||QRA
MKTFPIFCLLAVATTSAIAEMESNILISFLKPWLQQPLPPQQTLPQQQQPPVQQQPQPYP
QIPFPPQQSPCSQQQQPSFSQQQQPTFSQQQQPSYLQQQQPPFSQQEQPPFSQQEQPLVS
QQQQPVLPQQPPFSQQQQPVLPQQPPFLQQQQPVLPQQQIPFVHPYVLQQLNPCKIFLQQ
QCNPVAMPQRLARSQILQQSSCHVMQQQCCQQLPQIPEELRYDAIRAIVYSIILQQQQQQ
QGQGSVESQQQQSQQSGQGVSQPQQQSQLGQCAFQQPQLQQLGQQPQQQQAPLGTFLQPH
QIAQLEVMNSIALRTLPTMCSVNVPLYGTTTSVPFGVGTGVGAY

>NR|gi:222538182||Poaceae_E.ciliaris||QRA
MQTFFILALLAIVATTATTALRVQMAQLQPQNPSQQQPQEQSPLMQQVQFPWPQLPFPSQ
QPYPQQQPFPPRQPYPQPQPFPQPQPFPPQQPYPQPQPFPPQQPYPQPQQQYPLSQQTIS
QQQPQQGQQILQ

>NR|gi:240000202||Poaceae_E.ciliaris||QRA
MKTFLIFCLLAVAATSAIAEMESNIIISFLKPWLQQPLPPQQTLPQQRPIQQQPQPYPQT
PFPPQQPPCSQQQQPSFSQQQQPSFSQQQQPPFLQQEQPPFSQQQQPPFSQQQQPPFSQQ
QPPVSQQQQPVLPQQPPFLQQQQPVLPQQPPFSQQQQPVLPQQQIPFVHPSVLQQLNPCK
VFLQQLCSPVAMPQRLARSQMLQQSSCHVMQQQCCQQLPQIPKQSRYEAIRAIIYSIILQ
EQQQGQGSVESQQQQQPQQSGQGVSQPQQQSQLGQCAFQQPQLQQLGQQPQQQQAPHGTF
LQSHQIAQLEVMTSIALRTLTTMCSVNVPLYGTTTSVPFGVGTGVGAY

>NR|gi:153861760||Poaceae_E.sibiricus||QRA
MKTFLILALLAVVATTATIAVRVPVPQLRPQNPSQQQPQKQVPLVQQQQFPGQQQPFPPQ
QPYPQLQPFPSQQPYMQLQPFPQPQQPYPQPQLPYPQPQPFRPQQSYPQPQPQYSQPQQP
ISQQQQQQQQQQQILQQILQQQLIPCRDVVLQQHSIAHGSSQVLQQSTYQLVQQLCCQQL
WQIPEQSRCQAIHNVVHAIILHQQQQQQQQQPLSQVSFQQSQQQYPSGQGSFQPSQQNPQ
AQGSVQPQQLPQFEEIRNLALETLPAMCNVYIPPYCTIAPVGIFGTN

>NR|gi:51556914||Poaceae_H.brevisubulatum||QRA
MKTFLVFALLAVAATSSIAQMEISPISGLEGPWPQQPFPQQPYPQQPQPYPQQPQPYPQQ
PYLPQPQPYPQQPQPYPQQPQPYPSQQPFPQQSPFSQQQPCPQQQTPLQQVYQQLLQQQI
PFVQPFVLQQLNPCKVFLQQQCSPVAVPQRITRSQMLQQSSCYVLQQQCCQELPQIPEQS
RNEAIRAIVYSIILEQQRGEDFVQQQQQLQQSVQGVSQLQQAIQGVSQPQIGQCSFQQPQ
LQQLGQQPQQQQIPLAAFLQPHQIAQLEVMTSIALHTLPTMCNVNVPLYSTTTSAPFGVG
IGVGAY

>NR|gi:57118091||Poaceae_H.chilense||QRA
MKTFLIFALLAIAATNTIAQQQPFPQQPQPYPQQPQPYPQQPFPPQQPFPQQPPFWWQQP
VQSQQQPCQQQQTPLPQGQQYQPLLQQQIPFVHPSVLQQLNPCKVFLQQQCSPVPMPQRI
ARSQMLQQSSCHVLQQQCCKQLPQIPEQFRHEAIRAIIYSIILQEQQQVQDFVQPQQQQP
QQSVQGVSQSQQQSQQPQLGQCSFQQPQLQQLGQQPQQQQVPLWAFLQPQQMAQLEVMTS
VALRTLPTMCNVNVPLYGITTSVPLSVGTGVGPY

>NR|gi:110832715||Poaceae_H.vulgare||QRA
MKTFLVFALLVIAATSTIAQQQPFPQQPFPQQPQPYPQQPQPYPQQPFQPQQPFPQQPQP
YPQQPQPYPQQPQPFPQQPFPSQQPFPQQPPFWQQQPVLSQQQPCTQDQTPLLQEQQDQM
LLQVQIPFVHPSILQQLNPCKVFLQQQCSPVAMSQRIARSQMLQQSSCHVLQQQCCQQLP
QIPEQIRHEAVRAIVYSIVLQEQPLQLVQGVSQPQKQLGQQQVGQCSFQQPQPQQVGQQQ
QVPQSAFLQPHQIAQFEATTSIALRTLPTMCSVNVPSYRILRGVGPSVGV

>NR|gi:123464||Poaceae_H.vulgare||QRA
MKILIILTILAMATTFATSEMQVNPSVQVQPTQQQPYPESQQPFISQSQQQFPQPQQPFP
QQPQQPFPQSQQQCLQQPQHQFPQPTQQFPQRPLLPFTHPFLTFPDQLLPQPPHQSFPQP
PQSYPQPPLQPFPQPPQQKYPEQPQQPFPWQQPTIQLYLQQQLNPCKEFLLQQCRPVSLL
SYIWSKIVQQSSCRVMQQQCCLQLAQIPEQYKCTAIDSIVHAIFMQQGQRQGVQIVQQQP
QPQQVGQCVLVQGQGVVQPQQLAQMEAIRTLVLQSVPSMCNFNVPPNCSTIKAPFVGVVT
GVGGQ

>TC|TC154350||Poaceae_H.vulgare||QRA
MKIFLLFSLLGVATAITTTTMQFNPSGLELERPQQLFPQWQPLPQQPPFLQQEPEQPYPQ
QQPLPQQQPFPQQPQLPHQHQFPQQLPQQQFPQQMPLQPQQQPQFPQQKPFGQYQQPLTQ
QPYPQQQPLAQQQPSIEEQHQLNLCKEFLLQQCTLDEKVPLLQSVISFLRPHISQQNSCQ
LKRQQCCQQLANINEQSRCPAIQTIVHAIVMQQQQQQVQQQVGHGFVQSQLQQLGQGMPI
QLQQQPGQALVLPQQQAQFRVVGSLVIQTLSMLCNVHVPPYCSPFGKHGYRQGGHEMTRV
VYLIDGP

>NR|gi:442524||Poaceae_H.vulgare||QRA
MKTFLTFVLLAMVMSIVTTARQLNPSSQELQSPQQSYLQQPYPQNPYLPQQPFPVQQPFH
TPQQYFPYLPEELSPQYQIPTPLQPQQPFPQQPQQPLPRPQQPFPWQPQQPFPQPQQPIP
YQPQQPFNQQPQQIISQQPQQPFPQQPQQPFPQPQQPFPWQPQQPFPQPQQPFPLQPQQP
FPWQPQQPFPQPQQPIAHQPQQPFSFSQQPQQPFPLQPQQPFPQQPQQPFPQQPQQIIFQ
QPQQSYPVQPQQPFPQPQPVPQQRPQQASPLQPQQPFPQGSEQIIPQQPQQPFPLQPHQP
YTQQTIWSMV

>NR|gi:240000200||Poaceae_L.mollis||QRA
MKIVLIFALLAAAATSAIAQMETSRIPGLEKPWQQQPLPPQQTQEQPIPQQPQPYPQQPQ
PYPQQQQPFPQQPPFSQQQQPVLPQQQACPQQQPFPQQQTPLPQDQQLPQQQILFVQPSV
LQQLNPCKVFLQQQCSPVVMTQRLARSQMLQQSSCHVMQQQCCQQLPQIPEQSRYEAIRA
INYYIILQEQQQGQGFVQPQ

>NR|gi:194400021||Poaceae_L.elongatum||QRA
MKTFLILALFAIVATTATTAVRVPVPQLQPQNPSQQQPQEQVPLVQQQQFPGQQQPFPPQ
QPYPQPQPFPSQQPYPQLQPFPQPQPFPPQLPYPQPQPFPPQQPYPQPQPQYPQPQQPIS
QQQAQQQQQQQQQQQQQQQQQQQQQILQQILQQQLIPCRDVVLQQPNIAHASSQVLQQRY
QLLQQLCCQQLWQTPEQSRCQAIHNVVHAIILHQQQQQQQPSSQVSYQQPQQQYPSGQGS
FQPSQQNRQAQGSVQPQHLPQFEEIRNLALQTLPAMCNVYIPPYCSTTIAPFGIVDTN

>NR|gi:50952789||Poaceae_L.elongatum||QRA
MKTFLICALLAIAATSAIAQLPISQQQQPPFSTFLICALLAIAATSAIAQLPISQQQQPP
FSQQPQISQRQQQPPLSQQEQQPFSQQQQPPFSQQQQPPFLQQQQISQLQQPPFSQQQQP
PCSQQQQPPFSQQQPSFLQQQQPQISQHTNFTTTTTTILQQQQIPVIHPYVLQQLNPCKV
FLQQQCSPVAMQRGLVRSQMLQQGSCHVLQQQCCQQLPQIPEQSRHEAIRAIVYSIILQE
QQQGQGFIQPQQQQPQQSAQCVSQPQQQSQQQLGRQPQQQQLGQQPQQQQQVLQGTFLQP
HQIAQLEAMTSIALRTLPRMCSVNVPLYGTASSVSFGVGTGVGAY

>NR|gi:194307143||Poaceae_L.elongatum||QRA
MKTLLILCLLAVAATSAIAEMESNIIISFLKPWQQQPLPPQQTLPQQQQRPIQQQPQPYP
QIPFPPQQPPFSQQQQPSFSQQQQPSFSQQQQPSFSQQQQPPFSQQQQPSFSQQQQLPFS
QQQQPPFSQQQPPVSQQQQPVLPQQPPFSQQQQPVLPQQPPFSQQQQPVLPQQQIPFVHP
SVLQQLNPCKVFLQQLCSPVAMPQRLARSQMLQQSSCHVMQQQCCQQLPQIPEQLRYEAI
RAVIYSIILQEQQQGQGSVESQQQQPQQSGQGVSQPQQQSQLGQCAFQQPQLQQLGQQPQ
QQQAPHGTFLQPHQIAQLEVMTSIALRTLPTMCSVNVPLYGTTTSEPFGVGTGVGAY

>NR|gi:222538184||Poaceae_P.huashanica||QRA
MQTLFIFVLLAMAMSIATAARQLNPSGKELKSPQQPFPHQQQPFPEQQSYPQQPYPQQPY
PP

>NR|gi:222538173||Poaceae_P.huashanica||QRA
MKTFLVFALLAVAATSAIAQMETSRIPSLGKPWQQQPLPPQQTLSQQQPIPQQPQPYPQQ
PQPYPQHPQPYPQPFPPQQPPFSQQQQPFPQQPPFSQQQQPVLLQQPPFSQQQQPVLPQQ
QACPQQQPFPQQQTPLPQEQHRQQLPQQQIPFVQPSVL

>NR|gi:239829104||Poaceae_P.huashanica||QRA
MKTFLILALLAIAAGTATAVLSGPQQQLQLQQPCLQQPQQQIPLMQQQPFPLPQQPYPQT
QQPYPQPQLPISQQQQPFPLPQQPYPQQQLPISQPQGQQVPQQEQQILQPFLQQQLIPCR
DVLLQQRNAAHARSQVLQQSSYQELRQQCCQQLWQMPEQSRCQAIHSVVHAIILHHQQQQ
QQQWGVVTSVQPQQMQQLVQGTLVQPQQQNALGQGSIQQAHQQYPSCQGSIQQPQQQFQW
SQGSIEQPQQQYPSGQGSIQQPQQQYPSGQGSFQQPPQQYPLGQGSVQPQQLAQFEAIRK
LALQTLPALCNVYVPPYCSTTTAPFGIFGTN

>NR|gi:194400025||Poaceae_P.spicata||QRA
MKTFLILALLAIVATTATTAVRVPVPQLQPQNPSQQQPQEQVPLMQQQQFPGQQQPFPPQ
QPYPQPQPFPSQQPYPQPQPFPPQQPFPQPQPLPPQLPYPQPQPFPPQQPYPQPQQQYQQ
PQQQISQQQEQQILQQFLQQQLIPCRDVVLQQHDIAHASSQVLQQSNSQELQQLCCQQLW
QIPEQSRCQAIHSVVHAIILHHQQQQQQQQQQQQQQQPLSLGSFQQPQQQYPSGQGSFQP
SQQNPQGQGSVQPQQLPQFEEIRRLALQTLPTMCNVYVPPYCSTTIAPFGSVGIN

>NR|gi:229610190||Poaceae_S.cereale||QRA
MKTFLIFVLAMTMSIITTARQINPSEQELQSPQQPVPKGQSYPQQSYPSHQPFPTPQQYS
PYQPQQPFPQPQQPTPIQPQQPFPQQPQQPFPQPQQQLPLQPQQPFPQPQQPIRQQPQQS
FPQQPQRPEQQFPQQPQQIIPQQTQQPFPLQPQQPFPQQPQRPFAQQPEQIISQQPFPLQ
QQQPFSQPQQPFPQQPGQMIPQQPQQPSPLQPQQPFSQRPQRPQQPFPQQPQQIIPQQPQ
QPFPLQPQQPVPQQPQRPFGQQPEQIISQRPQQPFPLQPQQPFSQPQQPFPQQPGQIIPP
QPQQPFPLQPQQPFPQQPEQIISQQPQQPFPLQPQQPSPQQPQLPFPLPQQPFVVVV

>NR|gi:21204||Poaceae_S.cereale||QRA
MKTFLIFVLAMTMSIITTARQLNPSEQELQSPQQPVPKEQSYPQQPYPSHQPFPTPQQYS
PYQPQQPFPQPQQPAPIQPQQPFPQQPQQPFPQPQQQLPLQPQQPFPQPQQPIPQQPQQS
FPQQPQRPEQQFPQQPQQIIPQQTQQPFPLQPQQPFPQQPQRSFAQQPKQIISQQPFPLQ
PQQPFSQPQQPFPQQPGQIIPQQPQQPSPLQPQQPFSQQPQRPQQPFPQQPQQIIPQQPQ
QPFPLQPQQPVPQQPQRPFGQQPEQIISQRPQQPFPLQPQQPFSQPQQPLPQQPGQIIPQ
QPQQPFPLQPQQPFPQQSEQIIPQQPQQPFPLQPQQPSPQQPQLPFPQPQQPFVVVV

>NR|gi:11493665||Poaceae_S.cereale||QRA
MKTLLMLAILAMATTIATANMQVNPSGQVQCPQQQPFPQPQQSSPQQPQQPFPQQSQQPF
PQQPQQSSPQPQQPYPQQPFPQQPQQPYPQQPQQPFPQQPQQPYPQQPQQQFPQQPQQPV
PQQPLQQFPQQPQQPFPQQPLQQFPQQPQQPFPQQPQQPVPQQSQQPFPQTQQPQQPFPQ
PQQPQQLFPQTQQSSPQQPQQVTSQPQQPFPQAQPPQQSSPQSQQPYPQEPQQLFPQSQQ
PQQPFPQPQQPQQPFPQPQPQTQQSIPQPQQPFPQPQQPFPQSQEQFPQVHQPQQPSPQQ
QQPSIQLSLQQQLNPCKNVLLQQCSPVALVSSLRSKIFPQSECQVMQQQCCQQLAQIPQQ
LQCAAIHSVVHAIIMQQEQREGVQILLPQSHQQHVGQGALAQVQGIIQPQQLSQLEVVRS
LVLQNLPTMCNVYVPRQCSTIQAPFASIVTGIVGH

>NR|gi:130750677||Poaceae_S.strictum||QRA
MKTLLMLAILAMATTIATANMQVNPSGQVQCPQQQPFPQPQQSSPLQPQQPFPQQSQQPF
PHQPQQSSPQPQQPYPQQPFPQQPQQPYPQQPQQPFPQQPQQPYPQQPQQPFPQQPQQPV
PQQPQQQFPQQPQQPVPQQPLQQFPQQPQQPFPQQPLQQFPQQPQQPFPQQPQQPVPQQS
QQPFPQTQQPQQPFPQPQQPQQLFPQTQQSSPQQPQQVTSQPQQPFPQAQPPQQSSPQSQ
QPYPQEPQQLFPQSQQPQQPFPQPQQPQQPFPQPQPQTQQSIPQPQQPFPQPQQPFPQSQ
EQFPQVHQPQQPSPQQQQLSIQLSLQQQLNPCKNVLLQQCSPVALVSSLRSKIFPQSECQ
VMQQQCCQQLAQIPQQLQCAAIHSVVHAIIMQQEQREGVQILLPQSHQQHVGHGALAQVQ
GIIQPQQLSQLEVVRSLVLQNLPTVCNVYVPRQCSTIQAPFASIVTGIVGH

>NR|gi:130750612||Poaceae_S.sylvestre||QRA
MKTLLMLAILAMATTIATANMQVNPSGQVQCPQQQPSPQPQQSSPQQPQQPFPQQPQQPC
PQQPQQSSPQPLHQYPQNHFPRQPNHQYPQHQQQPFPQQPQQPFPQQPQQPVPQQPQQQF
PQLPQQPFPQQPQQPVPQQPLQQFPQQPQQPFPQQPQQPIPQQSQQPFPQTQQPQQPFPQ
PQQPQQLFPQPQQSSPQQPQQVISQPQQPFPQAQPPQQSSPQSQQPHPQEPQQLFPQPQQ
PQQPFPQPQQPQEPFPQLQPQQSIPQPQQPFPQPQQPFPQAQQPFPQVHQPQQPSPQQQQ
PSVQLSLQQQLNPCKNVLLQQCNPVALVSSLRSKIFPQSECQVMQQQCCQQLAQIPQQLQ
CAAIHSVVHAIIMQQEQQQGLQILLPQSHQQHVGQGALAQVQGIIQPQQLSQLEVVRSLV
LQNLPTMCNVYVPPQCSTIQAPFASIVTGIVGH

>NR|gi:130750657||Poaceae_S.vavilovii||QRA
MKTLLMLAILAMATTIATANMQVNPSGQVQCPQQQPFPQPQKSSPQQPQQPFPQQSQQPF
PQQPQQSSPQPQQPYPQQPFPQQPQQPYPQQPQQPFPQQPQQPYPQQPQQPFPQQPQQPV
PQQPQQQFPQQPQQPVPQQPLQQFPQQPQQPFPQQPLQQFPQQSQQPFPQTQQPQQPFPQ
PQQPQQLFPQPQQSSPQQPQQVTSQPQQPFPQAQPPQQSSPQSQQPHPQEPQQLFPQSQQ
PQQPFPQPQQPQQPFPQPQPQTQQSIPQPQQPFPQPQQPFPQYQEPFPQVHQPQQPSPQQ
QQPSIQLSLQQQLNPCKNVLLQQCSPVALVSSLRSKIFPQSECQVMQQQCCQQLAQIPQQ
LQCAAIHSVVHAIIMQQEQREGVQILLPQSHKQHVGQGALAQVQGIIQPQQLSQLEVVRS
LVLQNLPTMCNVYVPRQCSTIQAPFASIVTGIVGH

>NR|gi:194400029||Poaceae_T.intermedium||QRA
MKTFLILALLAIVATTATTAVRVPVPQLQPQNPSQQQPQEQVPLMQQQQFPGQQQPFPLQ
QPYPQPQPFPSQQPYPQPQPFPPQQLFPQPQPFPPQLPYPQPQPFPPQQPYPQPQQQYPQ
PQQPISLQQEQQILQQLLQQRLIPCRDVVLQQHNIAHGSSRVLQQSSYQELQQLCCQQLW
QIPEQSRCQAIHSVVHAIILHHHQQQQQQPSSQGSFQQPQQQYSSGQGSFQPSQQNPQGQ
SSVQPQQLPQFEEIRRLALQTLPTMCNVYVPPYCSTTSAPFSSLSIN

>NR|gi:56126399||Poaceae_T.ponticum||QRA
MKTFLVFDLLAVVATSAIAQMETSCIPGLERPWQQQPLQQKETFPQQPPSSQQQQPFPQQ
PPFLQQQPSLSQQPLFSQKQQPVLPQQPAFSQQQQTVLPQQPASSQQQHQQLLQQQIPIV
HPSILQQLNLCKVFLQQQCSPVAMPQHLARSQMWQQSSCNVMQQQCCQQLPRIPEQSRYE
AIRAIIFSIILQEQQQGFVQPQQQQPQQSVQGVYQPQQQSQQQLGQCSFQQPQQQLGQQP
QQQQVQKGTFLQPHQIARLEVMTSIALRTLPTMCSVNVPLYSSITSAPLGVGSRVGAY

>NR|gi:73427758||Poaceae_T.ponticum||QRA
MKTFLVFALIALGGTSAIAQNGRLDASLVWERPWQQQPLPPQQDISQQPLFSQQQQQQLF
PQQPSSWQQQPPFGQQPPFSQQQPILPQQPPFSQQQQLVLPQQPPFSQQQQPVLPPQQSP
FPQQQQQHQQLVQQQIPVVQPSILQQLNPCKVFLQQQCSPVAMPQRLARSQMLQQSSCHV
MQQQCCQQLPQIPQQSRYEAIRAIIYSIILQEQQQVQGSIQSQQQQPQQLGQCVSQPQQQ
SQQQLGQQPQQQQLAQGTFLQPHQIAQLEVMTSIALRILPTMCSVNVPLYRTTTSVPFGV
GTGVGAY

>NR|gi:154268848||Poaceae_T.ponticum||QRA
MKTFLILALLAIVATTATIAVRVPVPQLQPQNPSQQQPQEQVPLVQQQQFPGQQQPFPPQ
QPYPQPQPFPSQQPYLQLQPFPQPQLPYPQPQLPYPQPQLPYPQPQPFRPQQPYPQSQPQ
YSQPQQPISQQQQQQQQQQQKQQQQQQQQQILQQILQQQLIPCRDVVLQQHSIAYGSSQV
LQQSTYQLVQQLCCQQLWQIPEQSRCQAIHNVVHAIILHQQQQQQQQQQQKQPLSQVSFQ
QPQQQYPSGQGSFQPSQQNPQAQGSVQPQQLPQFEEIRNLALETLPAMCNVYIPPYCTIA
PVGIFGTN

>NR|gi:56126407||Poaceae_T.ponticum||QRA
MKTFLVFALLAVAATSAIAQMETRCIPGLERPWQQQPLPPQQTFPQQPLFSQQQQLFPQQ
PSFSQQQPPFWQQQPPFSQQQPILPQQPPFSQQQQLVLPQQPPFSQQQQPVLPPQQSPFP
QQQQHQQLVQQQIPVVQPSILQQLNPCKLFLQQQCSPVAMPQRLARSQMWQQSSCHVMQQ
QCCQQLQQIPEQSRYEAIRAIIYSIILQEHQQGFVQPQQQQPQQSGQGVSQSQQQSQQQL
GQCSFQQPQQQLGQQPQQQQQQVLQGTFLQPHQMAHLEAVTSIALRTLPTMCSVNVPLYS
ATTSVPFGVGTGVSAY

>NR|gi:157781831||Poaceae_T.ponticum||QRA
MKTFLIFALLATAATSAIAQMETSRVPGLEKPWQQQPLPPQQQPPCSQQQQPFPQQQQPI
IILQQSPFSQQQQPVLPQQQPVIILQQPPFSQQQQPVLPQQPPFSQQQQQQQQQQPPFSQ
QQQPVLPQQPPFSQQQQPPFSQQQQPSSQQPPFPQQHQQFPQQQIPVVQPSVLQQLNPCK
VFLQQQCSHVAMSQRLARSQMWQQSSCHVMQQQCCQQLPQIPEQSRSEAIRAIVYSIILQ
EQQQGFVQPQQQQPQQSGQGVSQHQQQSQQQQQLGQCSFQQPQQLQQLGQQPQQQQIPQG
IFLQPHQISQLEVMTSIALRTLPTMCGVNVPLYSSTTIMPFSIGTGVGAY

>TC|TC330341||Poaceae_T.aestivum||QRA
MKTLLILTILAMATTIATANMQVDPSGQVQWPQQQPFPQPQQPFCQQPQRTIPQPHQTFH
HQPQQTFPQPQQTYPHQPQQQFPQTQQPQQQIPQQPQQFPQQQFPQQQQFPQQQEFPQQQ
FPQQQFHQQQFPQQQFPQQQFPQQQQFPQQQQLTQQQFPRPQQSPEQQQFPQQPPQQFPQ
QQFPIPYPPQQSQEPSPYQQYPQQQPSGSDVISISGL

>NR|gi:31415655||Poaceae_T.aestivum||QRA
MKTFLVFALLAVVATSAIAQMETSCIPGLERPWQQQPLQQKETFPQQPPSSQQQQPFPQQ
PPFLQQQPSFSQQPLFSQKQQPVLPQQPPFSQQQQPVLPPQQSPFPQQQQQHQQLVQQQI
PVVQPSILQQLNPCKVFLQQQCNPVAMPQRLARSQMWQQSSCHVMQQQCCQQLPQIPEQS
RYDAIRAITYSIILQEQQQGFVQPQQQQPQQSVQGVYQPQQQSQQQLGQCSFQQPQQQLG
QQPQQQQVQKGTFLQPHQIARLEVMTSIALRTLPTMCSVNVPLYSSITSAPLGVGTGVGA
Y

>NR|gi:162316954||Poaceae_T.aestivum||QRA
MKTFLVFALLAVVATSAIAQMETSCIPGLERPWQHQPLQQKETFPQQPPSSQQQQQLLQQ
QQQPFPQQPPFLQQQPSFSQQPLFSQKQQQPVLPHQPAFSQQQQTVLPQQPAFSQQQHQQ
LLQQQQQIPIVHPSILQQLNPCKVFLQQQCIPVAMQRCLARSQVLQQSICHVMQQQCCQQ
LRQIPEQSRHESIRAIVYSIILQQQQQQQQQQQQRQSIIQYQQQQPQQLGQCVSQPQQQL
QQQLGQQPQQQQLTHGAFLQPHQIARLEVMTSIALRTLPTMCSVNVPLYSSTTSAPLGVG
TRVGAY

>NR|gi:147883544||Poaceae_T.aestivum||QRA
MKTFLILALLAIVATTATTAVRVPVPQLQPQNPSQQQPQEQVPLVQQQQFPGQQQQFPPQ
QPYPQPQPFPSQQPYLQLQPFPQPQPFPPQLPYPQPQSFPPQQPYPQQQPQYLQPQQPIS
QQQAQQQQQQQQQQQQQQQILQQILQQQLIPCRDVVLQQHNIAHASSQVLQQSTYQLLQQ
LCCQQLLQIPEQSRCQAIHNVAHAIIMHQQQQQQLQQQQQQQLQQQQQQQQQQQQPSSQV
SFQQPQQQYPSSQVSFQPSQLNPQAQGSVQPQQLPQFAEIRNLALQTLPAMCNVYIPPHC
STTIAPFGIFGTN

>NR|gi:194718433||Poaceae_T.aestivum||QRA
MKTFLVFALLAVAATSAIAQMETRCIPGLERPWQQQPLPPQQTFPQQPPFSQQQQQPFPQ
QPPFSQQQPPFSQQQQPVLPQQPSFSQQQLPPFSQQQPPFSQQQQPVLPQQPPFSQQQPV
LPPQQSPFPQQQQHQQLVQQQIPVVQPSILQQLNPCKLFLQQQCSPVAMPQRLARSQMLQ
QSSCHVMQQQCCQQLPQIPQQSRYEAIRAIIYSIILQEQQQVQGSIQSQQQQPQQLGQCV
SQPQQQSQQQLGQQPQQQQLAQGTFLQPHQIAQLEVMTSIALRILPTMCSVNVPLYRTTT
SVPFDVGTGVGAY

>NR|gi:209971871||Poaceae_T.aestivum||QRA
MKTLLILTILVMAVTIGTANMQVDPSGQVQWPQQQPVLLPQQPFSQQPQQTFPRPQQTFP
HQPQQQVPQPQQPQQPFLQPQQPFPQQPQQPFPQTQQPQQPFPQQPQQPFPQTQQPQQPF
PQQPQQPFPQTQQPQQPFPQLQQPQQPFPQPQQQLPQPQQPQQSFPQQQRSFIQPSLQQQ
LNPCKNILLQQCKPASLVSSLWSIIWPQSDCQVMRQQCCQQLAQIPQQLQCAAIHSVVHS
IIMQQQQQQQQQQGMHIFLPLSQQQQVGQGSLVQGQGIIQPQQPAQLEAIRSLVLQTLPS
MCNVYVPPECSIMRAPFASIVAGIGGQ

>NR|gi:56480737||Poaceae_T.aestivum||QRA
MKTFLIFALLAIAATSAVAHMETTRVPGLEKPWQQQPLPPQQQPPCSQQQQPFPQQQQPI
IILQQSPFSQQQQPVLPQQQPVIILQQPPFSQQQQPVLPQQPPFSQQQQQQQQQQQPPFS
QQQQPVLPQQPPFSQQQQPPFSQQQQPSSQQPPFPQQHQQFPQQQIPVVQPSVLQQLNPC
KVFLQQQCSHVAMSQRLARSQMWQQSSCHVMQQQCCQQLPQIPEQSRSEAIRAIVYSIIL
QEQQQGFVQPQQQQPQQSGQGVSQHQQQSQQQQQLGQCSFQQPQQLQQLGQQPQQQQIPQ
GIFLQPHQISQLEVMTSIALRTLPTMCGVNVPLYSSTTIMPFSIGTGVGGY

>NR|gi:229610215||Poaceae_T.aestivum||QRA
MKTFLIFVLAMTMSIVTTARQLNPSEQELESPQQPVLKEQSYPQQPYPSHQSFPTPQQYS
PYQPQQPFPHPQQPTTIQPQRPFPQQPQQPFPQPQQQLPLQPQQPFPQPEQPIPQQPQQL
FPQQPQRPQQQFPQQPQQIIPQQTQQPFPLQPQQPFPQQPQRPFAQQPEQIISQQPFPLQ
PQQPFSQPQQPFPQQPGQIIPQQPQQPSPLQPQQPFSQQPQRPQQPFPQQPQQIIPQQPR
QPFPLQPQKPVPQQPQRPFGQQPEQIISRRPQQPFPLQPQQPFSQPQQPFPQQPGQIIPQ
QPQQPFPLQPQQTFPQQSEQIISQQPQQPFPLQPQQPSPQQPQLPFPQPQQPFVVVV

>NR|gi:229610232||Poaceae_T.aestivum||QRA
MKTFLIFVLAMTMSIITTARQLNPSEQELQSPQQPVPKEQSYPQQPYPSHQPFPTPQQYS
PYQAQQPFPQPQQPTPIQPQQPFPQQPQQPFPQPQQQLPLQPQQPFPQPQHPIPQQPQQS
FPQQPQRPEQQFPQQLQQIIPQQTQQPFPLQPQQPFPQQPQRPFAQQPEQLISQQPFPLQ
PQQPFSQPQQPFPQQPGQIIPEQPQQPSPLQPQQPFSQQPQRPQQPFPQQPQQIIPQQPQ
RPFPLQPQQPVPQQPQRPFGQQPEQIISQRPQQPFPLQPQQPFSQPQQPFPQQPGQIIPQ
QPQQPFPLQPQQTFPQQSEQIISQQPQQPFPLQPQQPSPQQPQLPFPQPQQPFVVVV

>NR|gi:92429653||Poaceae_T.aestivum||QRA
MKTFLIFALLAVAATSAIAQMETSHIPGLEKPSQQQPLPLQQILWYHQQQPIQQQPQPFP
QQPPCSQQQQPPLSQQQQPPFSQQQPPFSQQELPILPQQPPFSQQQQPQFSQQQQPFPQQ
QQPLLLQQPPFSQQRPPFSQQQQQPVLPQQPPFSQQQQQQPILPQQPPFSRHQQPVLPQQ
QIPYVQPSILQQLNPCKVFLQQQCSPVAMPQSLARSQMLWQSSCHVMQQQCCQQLPRIPE
QSRYDAIRAIIYSIVLQEQQHGQGFNQPHQQQPQQSVQGVSQPQQQQKQLGQCFFQRPQQ
QQLGQWPQQQQVPQGTLLQPHQIAQLELMTSIALRTLPMMCSVNVPVYGTTTSVPFGVGT
QVGAY

>NR|gi:17425216||Poaceae_T.aestivum||QRA
MKTFLVFALLALAAARAVAQISQQQQQPPFSQQQQPPFSQQQQPPFSQQQQSPFSQQQEQ
QQQPPFLQQQQPPFSQQPPISQQQQPPFSQEQQPPFSQQQQPPFSQQQQPPYSQQQQPPF
SQQQQPPFSQQQQPPFSQQQQQTNKQQQQQPFTQQQPPFSQQPPISQQQQQQQQQQQPFT
QQQPPFSQQPPISQQQQPPFSQQQQTPFSQQQQIPVIHPSVLQQLNPCKVFLQQQCIPVA
MQRCLARSQMLQQSICHVMQQQCCQQLRQIPEQSRHESIRAIVYSIILQQQQQQQQQQQG
QSIIQYQQQQPQQLGQCVSQPQQQLQQQLGQQPQQQQLAHGTFLQPHQIAQLEVMTSIAL
HNLPMMCSVSVPLYETTTSVPLGIGIGVGVY

>NR|gi:169666933||Poaceae_T.aestivum||QRA
MKTFLIFALLAVAATSAIAQMENSHIPGLERPSQQQPLPPQQTLSHHHHQQPIQQQPQQF
PQQQPCSQQQQQPPLSQQQQPPFSQQQQPPFSQQQQPVLPQQPSFSQQQLPPFSQQQQPP
FSQQQQPVLPQQPSFSQQQLPPFSQQLPPFSQQQQPVLPQQPPFLQQQLPPFSQQLPPFS
QQQQPVLPQQPPFSQQQQQPILPQQPPFSQQQQPVLLQQQIPFVHPSILQQLNPCKVFLQ
QQCSPVAMPQSLARSQMLQQSSCHVMQQQCCQQLPQIPQQSRYEAIRAIVYSIILQEQQQ
VQGSIQTQQQQPQQLGQCVSQPQQQSQQQLGQQPQQQQLAQGTFLQPHQIAQLEVMTSIA
LRTLPTMCNVNVSLYRTTTRVPFGVGTGVGGY

>NR|gi:209971931||Poaceae_T.monococcum||QRA
MKTLLILTIFAASLTIATANIQVDPSGQVQWPQQQPFPQPQPFSQQPQQAFLQPQHTFPL
QPQQVFPQPQQPQQQFPQPQQPQQPFPQPQQPQLPFPQQPQQPFPQPQQPQQPFPQSQQP
QQPFPQPQQQFPQPQQPQQSFPQQQQPLIQPYLQQQMNPCKNYLLQQCNPVSLVSSLVSM
ILPRNDCQVMQQQCCQQLAQIPRQLQCTAIHSVVHAIIMQQEQQGIQILRPLFQLVQGQG
IIQPQQPAQYEVIRSLVLRTLPNMCNVYVRPDCSTINAPFASIVAGIGGQ

>NR|gi:217039703||Poaceae_T.monococcum||QRA
MKTFLILALLAIVATTATTAVRVPVPQLQPQNPSQQQPQEQVPVVQQQQQFLGQQQPFPP
QQPYPQPQPFPSQQPYLQLQPFPQPQLPYSQPQPFQPQQPYPQPQPQYSQPQQPISQQQQ
QQQQQQQQQQQQQQQQQQQQPQQILQQILQQQLIPCMDVVLQQHNIAQGRSQVLQQSTYQ
LLQELCCQHLWQIPEQSQCQAIHNVVHAIILHQQQKQQQQQQQPSSQVSFQQPQQQYPLG
QGSFRPSQQNPQAQGSVQPQQLPQFEEIRNLALQTLPAICDVYIPPYCTIAPFGHLGTN

>NR|gi:217039713||Poaceae_T.monococcum||QRA
MKTLLILTIIAVALTTTTANIQVNPSSQVQWPQQQQPFPQLQQPFSQQPQQIFPQPQQTF
PHQPQQAFPQPQQTFPHQPQQQFPQPQQPQQPFPQQPQQQFPQPQQPQQPFPQPQQPQLP
FLQQPQQPFPQPQQPQQPFPQLQQPQQPLAQPQQPQQPFPQQQQPLIQPYLQQQMNPCKN
CLLQQCNPVSLVSSLVSMILPRSDCQVMRQQCCQQLAQIPQQLQCAAIHGVVHSIIMQQE
QQQQQGIQILRPLFQLVQGQGIIQPLTTAQLEVIRSLVLGNSPKHVHVFVPTECSTTKGT
FASIVADIGGQ

>NR|gi:83752447||Poaceae_T.monococcum||QRA
MKTFLVFALLALAAASAVAQISQQQQQPPFSQQQQPPFSQQQQPPFSQQHQSPFSQQQEQ
QQQPPFSQQQQPPFSQQPPISQQQQPPFSQEQQPPFSHQQQPPFSQQQQPPYSQQQQPPF
SQQQQPPFSQQQQPPFSQQQQPFTQQQPPFSQQPPISLQQQPPFSQQQQTPFSQQQQIPA
IHPSVLQQLNPCKVFLQQQCIPVAMQRCLARSQMLQQSICHVMQQQCCQQLRQIPEQSRH
ESIRAIVYSIILQQQQQQQQQQQGQSIIQYQQQQPQQLGQCVSQPQQQLQQQLGQQPQQQ
QLTHGAFLQPHQIAQLEVMTSIALRNLPRMCSVNVPLYETTTSVPLGVGIGVGVY

>NR|gi:167375215||Poaceae_T.timopheevii||QRA
MKTFLVFALLAVVATSAIAQMETSCIPGLGETMGSNNHYNKKRHFHNKPPSSQQQQPFPQ
QPPFLQQQPSFSQQPLFSQKQQPVLPQQPAFSQQQQTVLPQQPAFPQQQHQQLLQQQIPI
VHPSILQQLNPCKVFLQQQCSPVAMPQHLARSQMWQQSSCNVMQQQCCQQLPRIPEQSRY
EAIRAIIFSIILQEQQQGFVQPQQQQPQQSVQGVYQPQQQSQQQLGQCSFQQPQQQLGQQ
PQQQQVQKGTFLQPHQIARLEVMTSIALRTLPTMCSVNVPLYSSITSAPLGVGSRVGAY

>NR|gi:205321006||Poaceae_T.timopheevii||QRA
MKTFLILSLLAIVATTATTAVRVPVPQLQPQNPSQQQPQEQVPLVQQLQYPGQQQPFPPQ
QPYPQPQPFPSQQPLPQPQPFLPQLPYPQPQPFPPQQPYPQPQPQYPQPQQPISQQQAQQ
AQQQQQQQQQQQQQQILQQILQQQQLIPCRDVVLQQHNIAHASSQVLQQSSYQLLQQLCC
QRLWQIPEQSRCQAIHNVVHAIILQQQQQQEQQQQQQQQQQQPSSQVSYQQPQQQYPSGQ
GSFQPSQQNPQAQGSVQPQQLPQFEEIRNLALQTLPAICNVYVPPYCSTTIAPSSIFGTN

>NR|gi:256259570||Poaceae_T.timopheevii||QRA
MRTFLVFALLALAAASAVAQISQQQQPPPFSQQQQPPFSQQQQPPFSQQQQSPFSQQQQP
PPFSQQQQPPFSQQPLISQQQQLPFSQQQQPQFSQQQQPPYSQQQQPPYSQQQQPPFSQQ
QQPPFSQQQQPSFSQQQQQPPFTQQLQPPFSQQSPISQQQQQQQQQQQQPFTQQQQPPFS
QQPPISQQQQPPFSQQQQPPFSQQQQIPVIHPSVLQQLNPCKVFLQQQCIPVAMQRCLAR
SQMLQQSICHVMQRQCCQQLRQTPEQSRHESIRAIIYSIILQQQQQQQQQQQQQQQQGQS
IIQYQQQPPQQLGQCVSQPQQQLQQQLGQQPQQQQLAHGTFLQPHQIAQLEVMTSIALRT
LPRMCSVNVPLYETTTSVPLGVGIGVGVY

>NR|gi:9931207||Poaceae_T.turgidum||QRA
MKTFLVFALLAVVATSAIAQMDTSCIPGLERPWQQQPLPPQQTFPQQPPFSQQQQQQPFP
QQPSFSQQQPPFSQQQPILPQGPPFSQQTQPVLPQQSPFSQQQQLILPPQQQQQLPQQQI
SIVQPSILQQLNPCKVFLQQQCSPMAMPQRLARSQMWQQSSCHVMQQQCCQQLSQIPEQS
RYDAIRAITYSIILQEQQQGFVQAQQQQPQQSGQGVSQSQQQSQQQLGQCSFQQPQQQLG
QQPQEQQVQQGTFLQPHQIAHLEVMTSIALRTLPTMCSVNVPLYSSTTSVPFGVGTGVGA
Y

>NR|gi:82880021||Poaceae_T.turgidum||QRA
MKTFLILALLAIVATTATTAVRVPVPQLQPQNPSQQQPQEQVPLVQQQQFPGQQQQFPPQ
QPYPQPQPFSSQQPYLQLQPFPQPQPFPPQLPYPQPQSFPPQQPYPQQQPQYLQPQQPIS
QQQAQQQQQQQQQQQQQQQQILQQILQQQLIPCRDVVLQQHNIAHASSQVLQQSTYQLLQ
QLCCQQLLQIPEQSRCQAIHNAAHAIIMHQQQQQQQEQQQQLQQQQQQQLHQQRQQPSSQ
VSFQQPQQQYPSSQVSFQPSQLNPQAQGSVQPQQLPQFAEIRNLALQTLPAMCNVYIPPH
CSTTIAPFGIFGTN

>NR|gi:209971803||Poaceae_T.turgidum||QRA
MKTLLIPTILAMAITISTANMQVDPSGQVQWPQQQLVPQPQQPLYQQPQQAFPQPQQTFP
HQPQQQVPQPQQPQQPFLQPQQAFPQQPQQPFPQTQQQQQQPFPQQPQQPFPQTQQPQQQ
FPQQQQQPFPQTQQPQQPFPQQPQQPLPQTQQPQQPFPQFQQPQQPFRQPQQQLPQPQQP
QQSFPQQQRPFIQPSLQQRLNPCKNILLQQCKPASLVSSLWSIIWPQNDCQVMQQQCCQE
LAQIPQQLQCAAIHSVVHSIIMQQQQQQQQQQQQGMHILLPLSQQQQVGQGTLVQGQGII
QPQQPAQLEAIRSLVLQTLPTMCNVYVPPECSTIRAPFASIVAGIGGQ

>NR|gi:217039793||Poaceae_T.turgidum||QRA
MKTFLVFALLAVAATSAIAQMETSHIPSLEKPLQQQPLPLQQILWYQQQQPIQQQPQPFP
QQPPCSQQQQPPLSQQQQPPFSQQQPPFSQQQQPVLPQQPPFSQQQQPFPQQQQPLLPQQ
PPFSQQQPPFSQQQQQPPFSQQRQQPILPQQPPFSQHQQPVLPQQQIPSVQPSILQQLNP
CKVFLQQQCSPVAMPQSLARSQMLWQSSCHVMQQQCCRQLPQIPEQSRYDAIRAIIYSIV
LQEQQRGQGLNQPQQQQPQQSVQGVSQPQQQQKQLGQCSFQQPQQQQLGQWPQQQQVPQG
TLLQPHQIARLEVMTSIAFHTPPTMCSTNVPVYGTTPSVPFGVGTRVGAY

>NR|gi:229610206||Poaceae_T.turgidum||QRA
MKTFLIFVLAMTMSIITTARQLNPSEQELQSPQQPVPKEQSYPQQPYPSHQPFPTPQQYS
PYQPQQPFPQPQQPTPIQPQQPFPQQPQQPFPQPQQQLPLQPQQPFPQPQQPIPQQPQQS
FPQQPQRPEQQFPQQPQQIIPQQTQQPFPLQPQQPFPQQPQRSFAQQPEQIISQQPFPLQ
PQQPFYQPQQPFPQQPGQIIPQQPQQPSPLQPQQPFSQQPQRPQQPFPQQPQQIIPQQPQ
QSFPLQPQQPVPQQPQRPFGQQPEQIISQRPQPPFPLQPQQPFSQPQQPFPQQPGQIIPQ
QPQQPFPLQPQQPFPQQPEQIIPQQPQQPFPLQPQQPSPQQPQLPFPQPQQPFVVVV

>NR|gi:133741921||Poaceae_T.turgidum||QRA
MKTFLIFALLAVAATSAIAQMENSHIPGLERPSQQQPLPPQQTLSHHQQQQPIQQQPQQF
PQQQPCSQQQQQPPLSQQQQPPFSQQQQPPFSQQQQPVLPQQPSFSQQQLPPFLQQQQPP
FSQQQQPVLPQQPSFSQQQLPPFSQQLPPFSQQQLPPFSQQLPPFSQQQQPVLPQQPPFS
QQQQSVLLQQQIPFVHPSILQQLNPCKVFLQQQCSPMAMPQSLARSQMLQQSSCHVMQQQ
CCQQLPQIPQQSRYEAIRAIVYSIILQEQQQVQGSIQTQQQQPQQLGQCVSQPQQQSQRQ
LGQQPQQQQLAQGTFLQPHQIAQLEVMTSIALRTLPTMCNVNVPLYRTTTRVPFSVGTGV
GGY

>NR|gi:30349131||Poaceae_T.turgidum||QRA
MKTFLVFALLALAAASAVAQISQQQQPPPFLQQQQPPFSQQQQPPFSQQQQSPFWQQQQQ
PPFSQQQQPPFSQQPVISQQQQLPFSQQQQPQFSQQQQPPYSQQQQPPYSQQQQPPFSQQ
QQPPFSQQQQPSFSQQQQQPPFTQQQQPPFSQQSPISQQQQQQQQQQQQPFTQQQQPPFS
QQPPISQQQQPPFSQQQQPPFSQQQQIPVIHPSVLQQLNPCKVFLQQQCIPVAMQRCLAR
SQMLQQSICHVMQRQCCQQLRQIPEQSRHESIRAIIYSIILQQQQQQQQQQQQQQQGQSI
IQYQQQPPQQLGQCVSQPQQQLQQQLGQQPQQQQLAHGTFLQPHQIAQLEVMTSIALRTL
PRMCSVNVPLYETTTSVPLGVGIGVGVY

>NR|gi:209971933||Poaceae_T.urartu||QRA
MKTLFILTILAMATTIATANMQVDPSGQVQWPQQQPFRQPQQPFYQQPQQTFPQPQQTFP
HQPQQQFPQPQQPQQQFLQPQQPQQPFPQPQQAQLPFPQQPQQPFPQPQQPQQPFPQSQQ
PQQPFPQPQQPQQSFPQQQQPLIQPYLQQQMNPCKNYLLQQCNPVSLVSSLVSMILPRSD
CQVMQQQCCQQLAQIPRQLQCAAIHSVVHSIIMQQEQQQGIQILRPLFQLVQGQGIIQPQ
QPAQYEVIRSLVLRTLPNMCNVYVRPDCSTINAPFASIVAGISGQ

>NR|gi:122831084||Poaceae_T.zhukovskyi||QRA
MKTFLIFALLAVAATSAIAQMETSHIPSLEKPLQQQPLPLQQILWYQQQQPIQQQPQPFP
QQPPCSQQQQPPLSQQQQPPFSQQQPPFSQQQQPVLPQQPPFSQQQQQFPQQQQPLSPQQ
PPFSQQQPPFSQQQQQPPFSQQQQQPVLLQQPPFSQHQQPVLPQQQIPSVQPSILQQLNP
CKVFLQQQCSPVAMPQSLARSQMLWQSSCHVMQQQCCRQLPQIPEQSRYDAIRAIIYSIV
LQEQQHGQGLNQPQQQQPQQSVQGVSQPQQQQKQLGQCSFQQPQQQQLGQWPQQQQVPQG
TLLQPHQIAQLEVMTSIALRTLPTMCSVNVPVYGATTIVPFGVGTRVGAY

**@ QRB Class [23 Proteins, 15 Species]**

>NR|gi:38373992||Poaceae_A.comosa||QRB
MAKRLVLFVAVVVALVALTLAEGEASGQLQCERELKACQQVMDQQLRDFSPECHPVVVSP
VAGQYQQQIVPKGGSFYPGETTPPQQLQQSIFWGIPALLKRYYPSITSPQQVSYYPGQAS
PQRPGQGQQPGQGQQSGQGQQGYYPTSPQQPGQWQQPEQGQPGYYPTSPQQPGQLQQPEQ
GQQAQQPGQGQPGYYPISSQLQPGQLQQPVQGQQGQQPGQGQQGQQPGQGQQPEQGQQGQ
QPGQGQQPGQGQQGQQLGQGQQGYYPTSLQQSGQGQPGYYPTSLQQPGQGQSEYYPTSLQ
QPGQGQQPGQLQQPGQGQQPGQLQQPAQGQQPGQGQQGQQPGQGQQGQQPGQGQQSRQGQ
PGYYPTSPQQSGQGQPGYYPTSSQQPGQSQQPGQGQQGQQLGQGQQAQQPGQGQQPGQRQ
PGYYPTSPQQSGQGQPGYYLTSPQQPGQLQQSAQGQEGQQPGQGQQGQQPGQGQQSQQPG
QGQQGQQPGQGQQGQQPGQGQPGYYPTSPQQSGQGQQPGQWQQPGPGYYPTSSLQPGQGQ
PGYDPTSPQQPGQGQQSGQLQQPAQEQQLAQGQQPAQGQQGQQPGQGQQGQQPGQGQQGQ
QPAQGQQGQQPAQGQQGQQPAQGQQPGQGQPGYYPTSPQEPGQGQQPGQWQQSGQGQPGY
YLTSLLQPGQGQQEYYPASLQQPGQWQQSGQGQQGYYSTPQLSGQGQQPGQWLQPGQGQQ
GYYPTSPQHSGQGQQPGQWLQPGQGQQGYYPTSPQQPEQGQQSGQGQQGYYSSYHVSAEH
QAPSLKVAKAQQLAAQLPAMCRLEGGDALSASQ

>NR|gi:18958491||Poaceae_A.markgrafii||QRB
MAKRLVLFVTVVIALVSLTAAEGEASRQLQCERELQESSLEACSQVVDQQLAGRLPWSTG
LQMRCCQQLRDVSAKCRPVAVSQVARQYEQTVLPPTGGSFYPRETTPLQQLQQGIFWGTS
SQTVQGYYPSVTSPHQGSYYPGQASSQQLGQGQQPGQGQQPGKWQEPGQGQQGYYLTYLQ
QPGQGQQGYYLTSLQQPGQGQQIGQWQQGYYPTSLQHPGQRQQLGQGQHIGQGQQPGQGQ
QIGQGQQPGHGQQGYYPTSPLQSGQGQQPGQWQQPGQGQQGYYTTSLQQPGQGQQGHYPA
SQHQPGQGQQGHYPASLQQSGQGQQGLHPASLQQPGQGQQTGQPGQRQQPGQGQQTGQGQ
QPEQEQQPGQGKQGYYPTSLWQPGQGQQPEQWQQPGQGQQGYDPSSLQQPGQEQQGHYPA
SVQQPGQGQQGHYPASVQQPGRKQPGQTQQPGQGQQLEQEQQPGQGQQLEQEQQPGQGKQ
GYYPTSPQQPGQGQQGYYPTSPQQPGQGQQPGQGQQGHCPTSPHQPGQAQQLGHGQQTGQ
VQQPGQGQQGYYPTSVQQPGQGQQSGQEQQSGQGHQPGQGQQSGQEQQGYDSPYHVSAEQ
QATNPKVAKAQHPAAQLPTMCQMEGGDALSASQ

>NR|gi:146291072||Poaceae_A.tauschii||QRB
MAKRLVLFVAVVVALVALTVAEGEASEQLQCERELQELQERELKACQQVMDQQLRDISPE
CHPVVVSPVAGQYEQQIVVPPKGGSFYPGETTPPQQLQQRIFWGIPALLKRYYPSVTSPQ
QVSYYPGQASPQRPGQGQQPGQGQQSGQGQQGYYPTSPQQPGQWQQPEQGQPGYYPTSPQ
QSGQLQQPAQGQQPGQGQQGQQPGQGQPGYYPTSSQLQPGQLQQPAQGQQGQQPGQGQQG
QQPGQGQQPGQGQQGQQPGQGQQPGQGQQGQQLGQGQQGYYPTSLQQSGQGQPGYYPTSL
QQLGQGQSGYYPTSPQQPGQGQQPGQLQQPAQGQQPGQGQQGRQPGQGQQGQQPGQGQQP
GQGQPGYYPTSPQQSGQGQPGYYPTSSQQPTQSQQPGQGQQGQQVGQGQQAQQPGQGQQP
GQGQPGYYPTSPQQSGQGQPGYYLTSPQQSGQGQQPGQLQQSAQGQKGQQPGQGQQPGQG
QQGQQPGQGQQGQQPGQGQPGYYPTSPQQSGQGQQPGQWQQPGQGQPGYYPTSPLQPGQG
QPGYDPTSPQQPGQGQQPGQLQQPAQGQQGQQLAQGQQGQQPAQVQQGQRPAQGQQGQQP
GQGQQGQQLGQGQQGQQPGQGQQGQQPAQGQQGQQPGQGQQGQQPGQGQQGQQPGQGQQP
GQGQPWYYPTSPQESGQGQQPGQWQQPGQGQPGYYLTSPLQLGQGQQGYYPTSLQQPGQG
QQPGQWQQSGQGQHWYYPTSPQLSGQGQRPGQWLQPGQGQQGYYPTSPQQPGQGQQLGQW
LQPGQGQQGYYPTSLQQTGQGQQSGQGQQGYYSPYHVSVEHQAASLKVAKAQQLAAQLPA
MCRLEGGDALSASQ

>NR|gi:55560063||Poaceae_C.delileana||QRB
MAKRLVLFAAVVVALVALTIAEGEASGQLQCERELQERSLKACRQVMDQQLRDVSPECHP
VVISPIARQYEQQIVVPPKGGSLYPGETTPPQQLQQSIFWGIPTLLRRYYPSVTSSQQAS
YYPGQASPQRPGQGQQPGQGQQPGPEQQGYYPISPQQPEQGQQPGQWQQPGQGQPGYYPT
SPQQPGQLQQPAQRQQGQQPGQGQQGQQPGQGQQSGQGQHGQQPGQGQQSGQRQQGYYPT
SPQQSGQGQHGQQPGQGQQSGQGQQGYYPTSPQQSGQGQQPGQRQPGYYPTSPQQPGQEQ
PGYYPTSPQQPGQLQQPAQGQQGQQPGQGQQGHQPGQGQQPGQGQPGYYPTSPQQPGQGQ
PGYYPTSPQQPGQGQQSAQGQQGQQPGQGQQGQQPGQEQQGQQPGQGQQPGQGQLGYYPT
SPQQSGQGQQPGQGQRLGQGQQPGQWQQPGQGQPGYYPTSPLQPGQGQQGYYPTSPRQPG
QGQQPGQWQQSGQGQQGYYPTSPQQSGQGQQPGQWLQPGQGQQGYYPTSPQQSGQGQQPG
QWLQPGQGQQPGQWLQPGQGQQPGQWLQPGQGQQGYYPTSPQQSGQGQQPGQWLQLGQGQ
QGYYPTSPQRPGQGQQSGQGQQGYYPTPPQQSGQGQQGYDSPYHVSAEHQAASLKVAKAQ
QLAAQLPAMCRLEGGDALSASQ

>NR|gi:145306451||Poaceae_D.breviaristatum||QRB
MAKRLVLFAAVVVALVALTAAEGGASAQLQCERELQASSLEACRRVVDQQLAIQLPWSTG
LQMRCCQQLRDVSPECRPVAVSQVVRQYEQQIVVPPKGGSFYPGETTPPQQLQQRILWGR
SSQTVQGYYPSVTSPQQGSYYPGQASLQQPGQGQQPGQWQEPGQGQQGYYPTSPQQPGQG
QQGYYPTSPQQPGQGYYPTFPHQSGQGQQPGQGQQPGQGYYPTFPQLPGQGQQGYYPTSP
QQSGQGQQPGQWQHPGQWQQPGQGQQGYYPTSPQQSGQGQQGYYPTSPRQPGQGYYPTFP
HQSGQGYYPTFPQLPGQGKQGYYPTSPQQSGQGQQPGQGQQSGQWQQPEQGQQSGQWQQP
GQGQQGYYTTFPQQPGQGQQLGQEQQGYNSPYHVSAEQQAASLMVAKAQQLAAQLPAMCR
LEGSAALSASQ

>NR|gi:222538169||Poaceae_D.villosum||QRB
MAKRVVLFAAVVVALVALTAAEGGASAQQRCERELQESSLEACRRVVDQQLAGQLPRSTG
LQMQCCQQLRDVSPECRPVAVSQVTRQYEQQIVVPPKGGSFYPGKTTPPQQLQQRILWGR
SSQTVQGYYPSVTSPQQGSYYPGQASPQQPGQGQQPGQWQEPGQGQHGYDPTSSQQLGQG
QQPGQRQQPGQGQQGYYPTSLQQPGQWQLPGQGQQGYYPTSPQQPGQGQQGYYPTSPQRS
GQWQLPGQGQQGYYPTSPQQPGQRQQGYYPTSPQQSGQGQQPGQGQQGYYPTSPQQSGQW
QQPGQGQQGYYPTYPRQPGQGQQSGQGQQGYYPTSPQQPGQGQQGYYPTSPQQPGQWQQP
GQGQQGYYPTSLQQPGQGQQTGQWQHPGQGQQRGQGYYPTFPQQPGQWQQGYYPTSPQQP
GQWQQPGQWQQGYYSTSPQQPGGQGQQLGQEQQGYNSPYHVSPEQQEASLMVAEAQQLAA
QLPAMCRLEGSAALSASQ

>NR|gi:225380770||Poaceae_L.mollis||QRB
MAKRLVLFAAVVIALVALAFGQLQCERELQESSLEACRRVVDQQLAGQLPWSTGLQMRCC
QQLRDISPECRPVAVSQVARQYEQQTPVPPKGGSFYPGETTQPQQLQQRIFWGRSLQPVQ
GSYPSVTSPQQGSYYAGQASPRQPGQGQQPGQGQQGYYPTSPQQPGQGQQGYYPTSPQQP
GQGQEPGQGQQGYYPTSPQQPGQGQQQGQGQQGYYPTSPQQPGQWQQPGQGQQGYYPTSP
LQPGQGQQGYYPTSPQQPGQGQQPEQRQQGYYPTSPQQPGEGQQGYYPTSPQQPGQGQQP
GQWQQPGQGHQPGQWQQPGQGQQGYYPTSPHQQPGRGQQLGQGQQSGQEQQGYDSPYHVS
VEQQAVSLKVAKAQQLAAQLPAMCRLEGGDALSTNQ

>NR|gi:124358366||Poaceae_L.elongatum||QRB
MAKRLVLFAAVVVALVALTTAEGEASRQLQCERELQESSLEACRQVVDQQLAGQLPWSTR
LQMRCCQQLRDVSAKCRPVAISQVARQYEQQTAMPPKGGSFYPGDTTPTQQLQQRIFWGR
SSQTVQGYYPSITSPQQGSYYPGQASPQQPGQGQQPGQGQQPGQGQQGYYPTSLHQLGHG
YYPTSPQQSRQRQQPGQGQQPEQRQQGYCPTSPQPGQGQPPGQGQQGYYPTSVQQPGQGQ
QTGQEQQSGQWQQPGKGHQGYYPTSPQQPGQGQQPGQWQQPGQGQQGYYPTSPQQPGQGQ
HEYYPTSPQQPGQGQQIGQGQQPEQGQQSGQGQQGYYPTSPQQSGQGQQPGQGQQPGQGQ
QSRQEQQGYKNPYHVSAGQQATGLKVAKAQQPAAQLPAMCRLEEGDALSASQ

>NR|gi:38492341||Poaceae_L.elongatum||QRB
MAKRLVLFAALVVALVALTAAEGEVSGQLQCERELQACQQVVDQQLRDASPECRPVAVSP
VTRQYEQQTVVPPKGGSFYPGETTPPQQLQQEIFWGIPTLLRRYYPSVTSPQQGSYYPGQ
TSPQRLGQGQQSGQGQQPEQGQQGQQPGQGQQPGQRQQGQQPGQGQQSGQGQQGYYPTSP
QQPGQGQQPGQEQQPGQGQPGYYPTSPQQPGQGQQSGQRQQPGQGQPGYYPTSPQQPGQG
QQSGHGQQPGQGQQSGQGQQCQQAEQGQQPGQGQQGYYPTSPQQPGQGQQPGQGQPGYYP
TSPQQPGQGQQSGQGQPGYYPTSPQQPGQGQQPGQGQQPGQGQQPGQGQQLGQGQQGQQP
RQGQQPGQGQQGYYPTSPQQPGQEQQSGQWQQPGQGQPGYYPTSPQQPRQGQQPGQEQQP
GQGQQSQQPRQGQQGHYPTSPQQPGENQQARQWQQPGQGQPGYYPTSPQQPGQGQRPGQA
QQSGQGQPGYYPSSPQQPGQLQQPTQGQQGYYPTSPQQTGQGQQGYYPTSPQQPGQGQQP
GQWQQPGQGQQGYYPTSPQQSGQGQQSGQWLQPGQGQQGYYPTSPQQLGQGQQSGHGQQP
GQWLQPGQGQQGYYPTSPQQSGQGQQSGQGQQGYYPTSLQQPAQGQQGYDRPYHVSAEYQ
AASLKVAKAQQLAAQLPAMCRLEGGDALSTSQ

>NR|gi:14329745||Poaceae_S.cereale||QRB
MAKRLVLFAAVVVALVALTVAEGEASGQLQCERELQERELEACRQIVDQQLRDTSPGCRP
VAVSPGTGQQEQQTVVPLKGGSFYPDETSPPQQLEQRILWGIPTLLKRYYPSVTSPHQGS
YYPGQTSLQQPGQAQQPGQGQQSGQAQQPGQGQQPGQGQQPEKGQQGYYPTTPQQPGQGQ
QPGQGQPGYYLTSSQQPGQGQQPGYYPTSPQQLGQGQQLGQGQQGQQPGQGQPGYYPTSP
QQPGQGQQPGQGQQPGQGQQGQQPAQGQQGQQPGQGQQPGEGQQGYCPTFPQQPGQVQQP
GQGQPGYYPTSPQQPGQGQQPGQEQQPGQRQQPGQGKPGYYPTSPQQSGQGQSGYYPTSP
QQPGQEQQPGQGQQVQQPGQGQQPGQGQQGYYPTSPQQSGQAQQPGQWQQPGQGQSGYYP
TSQQQPGQGQQPGQGQQPGQGQQGQQPGQGQQPGQGQQGYYPTSPQQPGQGQQPGQGQQP
GQGQTGYYPTSPQQPGQGQQTGQGQQPGQGQQPGQGQQGQQPGQGQQGYYPTSPQQPGQG
QQPGQGQLEYYPTSPQQPGQGQPGYYPTSPQLPGQLQQPAQGQQGYYPTSPQQPGQGQQK
YYPTSPQQPGQGQQGYYITSPQQSGQGQQPGRGLQPGQGQEGYYPTSGQQPGQWLQIGQG
QQGYYPTSPQQSGQGQQGYYLTSPQQPGQGKQPGQGQQSYDSPYHVSAEHQAASLKVAKA
QQLAAQLPAMCRLEGGDALLASQ

>NR|gi:14329729||Poaceae_S.cereale||QRB
MAKQLVLFAAVVVALVALTVAEGEASGQLQCERELQERELEACRQIVDQQLRDTSPGCRP
VAVSPGTGQHEQQTVVPLKGGSFYPDETSPPQQLEQRILWGIPTLLKRYYPSVTSPHQGS
YYPGQTSLQQPGQAQQPGQGQQPGQAQQPGQGQQPGQGQQPKKGQQGYYPTTPQQPGQEQ
QPGQGQQPGQGQPGYYLTSSQQPGQGQQPGQGQPGYYPTSPQQSGQGQQLGQGQQGQQPG
QGQPGYYPTSPQQPGQGQQPGQGQRPGQGQQGQQPGQGQQGQQSGQGQQPGEGQQGYYPT
FPQQPGQVQQPGEGQQPGQGQPGYYPTSPQQPGQGQQPGQRQQPGQGKPGYYPTSPQQSG
QGQQPGQGQSGYYPTSPQQPGQEQQPGQGQQVQQPGQGQQPGQGQQGYYPTSPQQSGQAQ
QPGQWQQPGQGQSGYYPTSQQQPGQGQQPGQGQQGQQQGQGQQPGQGQQGYYPTSPQQPG
QGQQPGQGQQPGQGQPGYYPTSPQQPGQGQQTGQGQQPGQGQQPGQGQQGQQPGQGQQGQ
QPGQGQQPGQGQQGYYPTSPQQPGQGQLEYYPTSPQQPGQGQPGYYPTSPQLPRQLQQPA
QGQQGYYSTSPRQPGQGQQEYYPTSPQQPGQWQQPGQGQQGYYITSPQQSGQGQQPGQGQ
QPGQWLQPEQGQEGYYPTSGQQPGQWLQIGQGQQGYYLTSPQQPGQGQQGYDSPYHVSAE
HQAASLKVAKAQQLAAQLPAMCRLEGGDALSASQ

>NR|gi:14329735||Poaceae_S.cereale||QRB
MAKRLVLFAAVVVAPVALTVAEGEASGQLQCERELQERELEACRQIVDQQLRDTSPGCRP
VAVSPGTGQQEQQTVVPLKGGSFYPDETSPPQQLEQRILWGIPTQLKRYYPSVTSPHQGP
YYPGQTSLQQPGQAQQPGQGQQPGQAQQPGHGQQSGQGQQPEKGQQGYYPTTPQQPGQGQ
QPGQGQPGYYLTSSQQPGQGQQPGQGQPGYYPTSSQQLGQGQQLGQGQQGQQPGQGQPGY
YPTSPQQPGQGQQPGQGQQPGQGQQGQQPGQGQQGQQPGQGQQPGEGQQGFYPTFPQQPG
QVQQPGQGQQPGQGQPGYYPTSPQQPGQGQQPGQEQQPGQRQQPGQGKPGYYPTSPQQSG
QGQSGYYPTSPQQPGQEQQPGQGQQVQQPGQGQQPGQGQQGCYPTSPQQSGQAQQPGQWQ
QPGQGQSGYYPTSQQQPGQGQQPGQGQQPGQGQQPGQGQQGQQPGQGQQAGQGQQGYYPT
SPQQLGQGQQPGQGQQPGQGQPGYYPTSPQQPGQGQQTGQGQQPGQGQQGQQPGQGQQPG
QGQQGYYPTSPQQPGQGQQPGQGQLEYYPTSPQQPGQGQPGYYPTFPQLPGQLQQPAQGQ
QGYYPTSPQQPGQGQQKYYPTSPQQPGQWQQPGQGQQGYYITSPQQSGQGQQPGQGQQPG
QGQEGYYPTLGQQPGQWLQIGQGQQGYYPTSPQQLGQGQQGYYLTSPQQPGQGKQPGQGQ
QSYDSPYHVSAEHQAASLKVAKAQQLAAQLPAMCRLEGGDALLASQ

>NR|gi:14329757||Poaceae_S.cereale||QRB
MAKRLVLFAAVVVALVALTVAEGEASGQLHCERELQERELEACRQIVDQQLRDTSPGCRP
VAVSPGTGQHEQQTVVPLKGGSFYPDETSPPQQLEQRILWGIPTLLKRYYPSVTSPHQGS
YYLGQTSLRQPGQAQQPGQGQQPGQAQQPGQGQQPGQRQQPEKGQQGYYPTTPQQPGQVQ
QPGQGQQPEQGQPGYYLTSLQQPGQGQQPGQGQPGYYPTSPQQSGQGQQLGQGQQGQQPG
QGQPGYYPTSPQQPGQGQQPGQGQQPGQGQQGQQPAQGQQGQQPGQGQQGQQPGQGQQPG
EGQQGYYPTFPQQPGQVQQPGQGQQPGQGQPGYYPTSPQQPGQGQQPGQGQQPGQRQQPG
QGKPRYYPTSPQQSGQGQQPGQGQSGYYPTSPQQPGQEQQPGQGQQVQQPGQGQQPGQGQ
QGYYPTSPQQPGQAQQPGQWQQPGQGQSGYYPTSQQQPGQGQQPGQGQQPGQGQQGQQPG
QGQQPGQGQQGYYLTSPQQPGQGQQPGQGQPGYYPTSPQQPGQGQQPGQGQQPGQGQQGQ
QPGQGQQPGQGQQGYYPTSLQQPGQGQQPGQGQLEYYPTSPQQPGQGQPGYYPTSPQLPG
QLQQPAQGQQGYYPTSPQQPGQGQQEYYPTSPQQPGQWQQPGQGQQGYYMTSPQQSGQGQ
QPGQGQHPGQGLQPGQGQEGYYPTSGQQPGQWLQIGQGQQGYYPTSSQQSGQGQQGYYLT
SPQQPGQGKQPGQGQQSYDSPYHVSAEHQAASLKVAKAQQLAAQLPAMCRLEGGDALLAS
Q

>NR|gi:14329731||Poaceae_S.cereale||QRB
MAKRLVLFAAVVVSLVALTVAEGEASGQLQCECELQERELEACRQIVDQKLQDTSPGCRP
IAVSPVTGQHEQQTVVPPKGGSFYPGETSPPQQLEQRILWGIPTLLKRYYPSVTSPHQGS
YYPGQTSPQQPGQAQQPGQEQQPGQGQQDQQPEQGQQPGKGQQGYYPITPQQPGQWQQPG
QVQPGYYLTPPQQTGQAQQPGQGQQPGQGQPGYYPTSPXQPGQGQQSGQGQQGQLPGQWQ
QPGQEQPGYNPTSPQQPGEGQQPGQGRQPGQGQQPGQGQQGQQPGQGKQPGQGQQGYYPT
SPQHPGQGQQPGQGQQPGQGKPGYYPTSPQLPGQGQQPGQGQSGYYPTSPQQLGQGQQPG
QGWQPGQGQQPGQGQQGQQPGQGQQPGQGQQGYYPTSPQHLGQGQQPGQGKPGSYPTSPQ
QPGQGQQPVQGQSGYYPTSPQQPGQGQQAGQGQQVQQPGQGQQSGQGQQGYYPTSPQLSG
QAQQPGQWQQPGQGQPGYYPTSQQQPGQGRQPGQEQQDQQPGQGQQQGQGQQGYYPTSPQ
QPEQGQQPGQGQQPGQGQPGYYPTSPQQPGQGQQPGQEQQPGQGQQPGQGQQGYYLTSPQ
QPGQGQQPGQWQQPGQGQLGYYPTSPQQPGQGQPGYYPTSPQLPGQLQQPAQGQQGYYPT
TPQQPGQGQQPGQGQQGYYPTSPQQSGRGQQPGQWLQPGQGQEGYYPTSPQQPGQGQQPG
QWLQIGQGQQGYYPTSPQQPGQGQQGYYLTSPQQPGQGKQSGQGQQGYDSPYHVSVEHQA
ASLKVAKAQQLAAQLPAMCRLEGGDALSASQ

>NR|gi:31075344||Poaceae_T.caput-medusae||QRB
MAKRLVLFAAVVVALVALTVAEGEASGQLQCERELKACRQVVDQQLRDVSPKCHPVIVSP
VARQYEQQIVVPPGETTPPQQLQQSIFWGIPTLLRRYYPSVTSPQQVSYYPGQVSPQQAT
QGQQVGQGQGQQGYYPTSPQQQEQGQQSEQWQQPGQGQPGYYPTSPQQAGQGQPGYYPTS
SQQPGQLQQPAQGQQGQQSGQGQQGQRSGQRQQGQQPGQGQQPEQGQQGQQPGPGQQGQQ
SGQGQQPGQGQQGYYPTSPQQSGQGQQPEQGQQLGQGQPGYYPTSPQQPGQGQPEYYPTS
PQQPGQLQQPAQGQQGQQGQQPGQGQQGQGQQPGQGQQGQGQQPGQGQQPGQGQPGYYPT
SPQQPGQGQPGYYPTSSQQPGQLQQPAQGQQEQQPGQGRQGQQPGQGQQPGQLQQPAQGQ
QGQQPGQGEQGQQPGQDQQPGQGQQGQQPGQGQQPVQGQPRNYLTSPQQLGQGQQPRQWQ
QPGQGQPGYYPTSPLQSGQGQQGYYPTSPQQPGQEQQPGQWQQSGQGQQGYYPTSPQQSG
QGQQPGQWLQPAQGQQGYYPTSPQQSGQGQQPGQGQQSGQGQQPGQGQQSGQGQQPGQWL
QPGQGQQGYYPTSPQQSGQGQQPGQWLQPGQGQQGYYPTSPQQSGQGQQPGQWLQPGQGQ
QGYYPTSPQQPGQEQQSGHGQQGYYPTSPQQPGEGQQGYDSPYHVSAEHQAASLKVAKAQ
QLAAQLPAMCRLEGGDALSASQ

>NR|gi:30230653||Poaceae_T.elongatum||QRB
MAKRLVLFAVVVVALVALTTAEGEASRQLQCERELQESSLEACRQVVDQQLAGQLPWSTG
LQMRCCQQLRDVSAKCRPIAVSQVARQYEQQTTMPPKGGSFYPGDTTPTQQLQQRIFWGR
SSQTVQGYYPSVTSPQQGSYYPGQASPQQPGQGQQPGQGQQPGQGQQGYYPTSLHQLGHG
YYPTSPQQSRQRQQPGQGQQPEQRQQGYCPTSPQPGQGQQGYYPTSLQQPGQGQQTGQEQ
QSGQWQQLGKGHQGYYPTSPQQPGQGQQPGQWQQPGQGQQGYYPTSPQQPGQGQQIGQGQ
QGYLPTSSQQSGQGQQGYYPTSLQQLGQGQQIGQVQQSGQWQQPGKGHQGYYPTSPQQPG
QGQQPGQWQQPGQGQQGYYPTSPQQPGQGQHEYYPTSPQQPGQGQQIGQGQQPEQGQQSG
RGQQGYYTTSPQQSGQGQQPGQGQQSGQEQQGYNNPYHVSTGQQATGLKVSKAQQPVAQL
PAMCRLEEGDALSASQ

>NR|gi:163964194||Poaceae_T.intermedium||QRB
MAKRLVLFVAVVVALVALTVAEGEASGQLQCERELQERELQACQQVMDRQLRDISPECHP
VVVSPVAGQYEQQIVVPPKGGSFYPGETTSPQQLQQSILWRIPALLKRYYPSVTSPQQVS
YYPGQASPQRPGQGQQPGQGQQSGQGQQGYYPTSPQQPGQWQQPEQGQPGYYPTSPQQPG
QLQQPAQGQQGQQPGQGQLGQQPGQGQQSGQGQQGQQPGQGQQPGQGQQGQQLGQGQQGY
YPTSLQQSGQGQPGYYPTSLQQLGQGQSGYYPTSPQQPGQGQQPGQLQQPAQGQQPGQGQ
QGQQPGQGQQGQQPGQGQQPGQGQPGYYPTSPQQSGQGQPGYYPTSSQQPTQSQQPGQGQ
QGQQVGQGQQAQQPGQGQQPGQGQPGYYPTSPQQSGQGQPGYYLTSPQQSGQGQQPGQLQ
QSAQGQKGQQPGQGQQPGQGQQGQQPGQGQQGQHPGQGQPGYHPTSPQQSGQGQQPGQWQ
QPGQGQPGYYPTSPLQPGQGQPGYDPTSPQQPGQGQQPGQLQQPAQGQQGQQLAQGQPWQ
QPAQGQPGQQLAQGQQGQQPGQGQQGQQPGQGQQGQQPGQGQQGQQPGQGQQGQQPGQGQ
QGQQPGQGQQGQQPGQGQQPGQGQPWYYPTSPQESGQGQQPGQWQQPGQGQPGYYLTSPL
QLGQGQQGYYPTSLQQPGQGQQPGQWQQSGQGQQGYYPTSPQLSGQGQRPGQWLQPGQGQ
QGYYPTSPQQSGQGQQLGQWLQPGQGQQGYYPTSLQQAGQGQQSGQGQQGYYSSYHVSAE
HQAASLKVAKAQQLAAQLPAMCRLEGGDALSASQ

>NR|gi:71159572||Poaceae_T.junceum||QRB
MAKRLVLFAAVIVALVALTAAEGEASEQLQCERELRERELEACQQVVDQQLRDASPECRP
VAVSPVARQYEQQTVVPPKGGSFYPGETTPPQQLQQGIFWGIPALLRRYYPSVTSSQQGS
YYPGQASPQQSGQGQQGQQPGQGQQRYYPTSPQQPGQWQQSGQRQPGYYPTSSQQPGQGQ
QPGQGQPGYYPTSPHQSGQGQQPGQGQQPGQGQQPGQGQQGQQPGQGQQSGQGQQGYYPT
SPQQPGQRQQPGQGQPGYYPTSPQQPGQGQQPGQGQQPGQGQQGYYPTSSQQSGQGQQPG
QGQPGYYPTSPQQPGQGQQPGQGQQSRQGQQPGQGQQGQQPGQGQQGYYPTSPQQSGQEQ
QARQWQQPGQGQPGYYQTSPQQPEQEQQPGQAQQSGQGQPVYYPTSPQQPGQLQQPAQGQ
QGYYPTSPQQSGQGQQGYYPTSPQQPGQGQQPRQGQQGYYPTSPQQSGQGQQPGQWLQPG
QGQQGYYPTSPRQLGQGQQSGHGQQSGQWLQPGQGQQGYYPTSPRQPGQGQQSGHGQQSG
QWLQPGQGQQGYYPTSPQQPGQGQQSGQGQQGYYPTSPQQPGQGQQSGQGQQGYDSPYHV
SAEYQAASLKVAKAQQLAAQLPAMCRLEGGDALSASQ

>NR|gi:109716080||Poaceae_T.ponticum||QRB
MAKRLVLFAALVVALVALTAAEGEASGQLQCERELQACQQVVDQQLRDASPECRPVAVSP
VARQYEQQTVVPPKGGSFYPGETTPPQQLQQEIFWGIPTLLRRYYPSVTSPQQGSYYPGQ
TSPQRLGQGQQSGQGQQPEQGQQGQQPGQGQQPGQRQQGQQPGQGQQSGQGQQGYYPTSP
QQPGQGQQPGQEQQPGQGQPGYYPTSPQQPGQGQQPGQRQQPGQGQPGYYPTSPQQPGQG
QQSGHGQQPGQGQQSGQGQQRQQVGQGQQPGQGQQGHYPTSPQQPGQEQQPGQGQPGYYP
TSPQQPGQGQQSGQGQPGYYPTSPQQPGRGQQPGQGQQPGQGQQPGQGQQLGQGQQGQQP
RQGQQPGQGQQGYYPTSPQQPGQEQQSGQWQQPGQGQPGYYPTSPQQPRQGQQPGQEQQP
GQGQQSQQPRQGQQGHYPTSPQQPGENQQARQWQQPGQGRPGYYPTSPQQPGQGQQPGQA
QQSGRGQPGYYPSSPQQPGQLRQPTQGQQGYYPTSPQQTGQGQQGYYPTSPQQPGQGQQP
VQWQQPGQGQQGYYPTSPQQSGQGQQSGQWLQPGQGQQGYYPTSPQQLGQGQQSGHGQQP
GQWLQPGQGQQGYYPTSPQQSGQGQQSGQGQQGYYPTSLQQPAQGQQGYDSPYHVSAEYQ
AASLKVAKAQQLAAQLPAMCRLEGGDALSASQ

>NR|gi:94315067||Poaceae_T.ponticum||QRB
MAKRLVLFVAVVVALVALTAAEGEASEQLQCERELQELQERELKACQQVMDQQLRDISPE
CHPVVVSPVAGQYEQQIVVPPKGGSFYPGETTPPQQLQQRIFWGIPALLKRYYPSITSPQ
QVSYYPGQASPQRPGQGQQPGQEQQSGQGQQGYYPTSPQQPGQWQQPEQGQPGYYPTSPQ
QPGQLQQPAQGQQPGQGQQGRQPGQGQPGYYPTPSQLQPGQLHQPAQGQQGQQPGQGQQG
QQPGQGQQPGQGQQGQQPGQGQQPGQGQQGQQLGQGQQGYYPTSLRQSGQGQPGYYPTSL
QQLGQGQSGYYPTSPQQPGQGQQPGQLQQPAQGQQPEQGQQGQQPGQGQQDQQPGQGQQP
GQGQPGYYPTSPQQSGQGQPGYYPTSSQQPTQSQQPGQGQQGQQVGQGQQARQPGQGQQP
GQGQPGYYPTSPLQSGQGQPGYYLTSPQQSGQGQQPGQLQQSAQGQKGQQPGQGQQPGQG
QQGQQPGQGQQGQQPGQGQPGYYPTSPQQSGQGQQPGQWQQPGQGQPGYYPTSPLQPGQG
QPGYDPTSPQQPGQGQQPGQLQQPAQGQQGQQLAQGQQGQQPAQVQQGQQPAQGQQGQQL
GQGQQGQQPGQGQQPARGQQGQQPGQGRQGQQPGQGQQPGQGQPWYYPTSPQESGQGQQP
GQWQQPGQWQQPGQGQPGYYLTSPLQLGQGQQGYYPTSLQQPGQGQQPGQWQQSGQGQHG
YYPTSPQLSGQGQRPGQWLQPGQGQQGYYPTSPQQSGQGQQLGQWLQPGQGQQGYYPTSL
QQTGQGQQSGQGQQGYYSSYHVSVEHQAASLKVAKAQQLAAQLPAMCRLEGGDALSTSQ

>NR|gi:6684162||Poaceae_T.aestivum||QRB
MAKRLVLFAAVVVALVALTVAEGEASGQLQCERELQERELEACRQIVDQQLRDTSPGCRP
VAVSPGTGQQEQQTVVPLKGGSFYPDETSPPQQLEQRILWGIPTLLKRYYPSVTSPHQGS
YYPGQTSLQQPGQAQQPGQGQQPGQAQQPGHGQQSGQGQQPEKGQQGYYPTTPQQPGQGQ
QPGQGQPGYYLTSSQQPGQGQQPGQGQPGYYPTSSQQLGQGQQLGQGQQGQQPGQGQPGY
YPTSPQQPGQGQQPGQGQHPGQGQQGQQPGQGQQGQQPGQGQQPGEGQQGYYPTFPQQPG
QVQQPGQGQQPGQGQPGYYPTSPQQPGQGQQPGQEQQPGQRQQPGQGKPGYYPTSPQQSG
QGQSGYYPTSPQQPGQGQQPGQGQQVQQPGQGQQPGQGQQGYYPTSPQQSGQAQQPGQWQ
QPGQGQSGYYPTSQQQPGQGQQPGQGQQPGQGQQPGQGQQGQQPGQGQQAGQGQQGYYPT
SPQQLGQGQQPGQGQQPGQGQPGYYPTSPQQPGQGQQTGQGQQPGQGQQGQQPGQGQQPG
QGQQGYYPTSPQQPGQGQQPGQGQLEYYPTSPQQPGQGQPGYYPTFPQLPGQLQQPAQGQ
QGYYPTSPQQPGQGQQKYYPTSPQQPGQWQQPGQGQQGYYITSPQQSGQGQQPGQGQQPG
QGQEGYYPTLGQQPGQWLQIGQGQQGYYPTSPQQLGQGQQGYYLTSPQQPGQGKQPGQGQ
QSYDSPYHVSAEHQAASLKVAKAQQLAAQLPAMCRLEGGDALLASQ

>NR|gi:14329761||Poaceae_T.aestivum||QRB
MAKRLVLFAAVVVSLVALTVAEGEASGQLQCERELQERELEACRQIVDQKLRDTSPGCRP
IAVSPVTGQHEQQTVVPPKGGSFYPGETSPPQQLEQRILWGIPTLLKRYYPSVTSPHQGS
YYPGQTSPQQPGQAQQPGQEQQPGQGQQDQQPEQGQQPGKGQQGYYPTTPQQPGQWQQPG
QVQPGYYLTPPQQTGQAQQPGQGQQPGQGQPGYYPTSPRQPGQGQQSGQGQQGQLPGQWQ
QPGQEQPGYNPTSPQQPGEGQQPGQGRQPGQGQQPGQGQQGQQPGQGKQPGQGQQGYYPT
SPQHPGQGQQPGQGQQPGQGKPGYYPTSPQLPGQGQQPGQGQSGYYPTSPQQLGQGQQPG
QGWQPGQGQQPGQGQQGQQPGQGQQPGQGQQGYYPTSPQHLGQGQQPGQGKPGSYPTSPQ
QPGQGQQPVQGQSGYYPTSPQQPGQGQQAGQGQQVQQPGQGQQSGQGQQGYYPTSPQLSG
QAQQPGQWQQPGQGQPGYYPTSQQQPGQGQQPGQEQQDQQPGQGQQQGQGQQGYYPTSPQ
QPEQGQQPGQGQQPGQGQPGYYPTSPQQPGQGQQPGQEQQPGQGQQPGQGQQGYYPTSPQ
QPGQGQQPGQWQQPGQGQLGYYPTSPQQPGQGQPGYYPTSPQLPGQLQQPAQGQQGYYPT
TPQQPGQGQQPGQGQQGYYPTSPQQSGQGQQPGQWLQPGQGQEGYYPTSPQQPGQGQQPG
QWLQIGQGQQGYYPTSPQQPGQGQQGYYLTSPQQPGQGKQSGQGQQGYDSPYHVSVEHQA
ASLKVAKAQQLAAQLPAMCRLEGGDALSASQ

>NR|gi:194077448||Poaceae_T.aestivum||QRB
MAKRLVLFVAVVVALVALTVAEGEASEQLQCERELQELQERELKACQQVMDQQLRDISPE
CHPVVVSPVAGQYEQQIVVPPKGGSFYPGETTPPQQLQQRIFWGIPALLKRYYPSVTSPQ
QVSYYPGQASPQRPGQGQQPGQGQQSGQGQQGYYPTSPQQPGQWQQPEQGQPGYYPTSPQ
QPGQLQQPAQGQQPGQGQQGRQPGQGQPGYYPTSSQLQPGQLQQPAQGQQGQQPGQGQQG
QQPGQGQQPGQGQQGQQPGQGQQPGQGQQGQQLGQGQQGYYPTSLQQSGQGQPGYYPTSL
QQLGQGQSGYYPTSPQQPGQGQQPGQLQQPAQGQQPEQGQQGQQPGQGQQGQQPGQGQQP
GQGQPGYYPTSPQQSGQGQPGYYPTSSQQPTQSQQPGQGQQGQQVGQGQQAQQPGQGQQP
GQGQPGYYPTSPLQSGQGQPGYYLTSPQQSGQGQQPGQLQQSAQGQKGQQPGQGQQPGQG
QQGQQPGQGQQGQQPGQGQPGYYPTSPQQSGQGQQPGQWQQPGQGQPGYYPTSPLQPGQG
QPGYDPTSPQQPGQGQQPGQLQQPAQGQQGQQLAQGQQGQQPAQVQQGQQPAQGQQGQQL
GQGQQGQQPGQGQQPAQGQQGQQPGQGQQGQQPGQGQQPGQGQPWYYPTSPQESGQGQQP
GQWQQPGQWQQPGQGQQGQQPGQGQPGYYPTSPQQSGQGQQPGQWQQPGQGQPGYYPTSP
LQPGQGQPGYDPTSPQQPGQGQQPGQLQQPAQGQQGQQLAQGQQGQQPAQVQQGQQPAQG
QQGQQLGQGQQGQQPGQGQQPAQGQQGQQPGQGQQGQQPGQGQQPGQGQPWYYPTSPQES
GQGQQPGQWQQPGQWQQPGQGQPGYYLTSPLQLGQGQQGYYPTSLQQPGQGQQPGQWQQS
GQGQHGYYPTSPQLSGQGQRPGQWLQPGQGQQGYYPTSPQQSGQGQQLGQWLQPGQGQQG
YYPTSLQQTGQGQQSGQGQQGYYSSYHVSVEHQAASLKVAKAQQLAAQLPAMCRLEGGDA
LSASQ

**@ SPAP Class [23 Proteins, 3 Species]**

>NR|gi:117606933||Chlamydomonadaceae_C.incerta||SPAP
MMRRQRAAPFVGAVNVLIVALAFVASANAQCVPGGIFNCPPSPAPPSPAPPSPPPSPLPP
SPAPPSPAPPSPTPPSPEPPSPAPPSPPPSPAPPSPAPPSPVPPSPAPPLPPSPVPPSPA
PPSPVPPSPAPPVPPSPAPPSPAPPLPPSPVPPSPAPPAPPSPAPPSPAPPSPPPSPAPP
SPEPPSPAPPSPPSPAPPSPAPPSPAPPSPAPPVPPSPAPPSPAPPSPPPSPAPPSPEPP
SPVPPSPAPPSPVPPSPAPPSPVPPSPPIPPSPAPPSPAPPSPVPPSPAPPSPVPPSPVP
PSPPPPPSPPPPPPPRPPFPANTPMPPSPPSPPPSPPPPTPPSPPSPPPPSPVPPSPAPG
TPVPPSPAPPSPAPSPIPPSPAPPSPPPSPAPPSPSPSPSPSPSPSPSPSPSPSPSPSPK
PSPSPSPSPSPVSVKLVWADDAIAFDDLNGTSTKPGSASRMIGEPNIASTQCKGNLNGWM
PKPNKNPRWGQALFSGGRTVGSVANVTIRVAFVTQTPKLVYSSIELVVYNSGATGGGSTG
AAIMRVPIAANVTRSAIKCPGFLTLAPVPVAGYPVGIDATTWPNWKIAGVRINMGVGNKK
PKTSIDAVGLNLK

>NR|gi:60678627||Chlamydomonadaceae_C.incerta||SPAP
MSQATHRSLPVCPAGRLQVSSARMLPASARALRPAGSPAWLVLLAISLACIASPAFGAPR
VPVDVSVTSNASLDAPSANAYLVALGDVMWNVPWQPNCLPDPRRRDDTPWPHRCALPAVQ
YGYYDETYIDATVYSGAALRPFYATCRYPASTDPYAFLQPYSMPWVDDYGLYTPIVDAFN
AWWDNTMCDSASAAKWTDPAWTAQGAWLGGTTLRDATWNRDAHYCSWPFVECAGCEDYQI
ADPNDPDKVAAGIAPPVITALDFRNASLYLYGLDFSMFPAGSLDNVWYLNLGFNFIGGGL
PPDLPTTFPQLQHLGLDHCRAAPGLRDAPSLQYGWQQYPGTGQSYEYCPDSDVVADDCYG
DVMSYTIVGFIPDSWGDAVAAAGNNASKPWGNLQTVRLANQDLQGPIPEGLRTSAAISSW
RLQGNAELCGQLPTYGLAINSLLYLGTKLGTHTWVWNNPADHRLGGQCLAPPPSPSPAPP
RPPPFPPSPPPNPRPPSPPVPPSPPPTPPSPPPTPPAPPPVPPLPPAPPPSPPLPPEPPF
IALCIRAGVLCAMPSPVPPLPSPPRPPSPQPPVPRPPPRAPRPPRPPSPPAPPAPPDPPT
APPSPDPPSPEPPSQPSPPSPDPPSPDPPSPPALPPSPKPPSPKPPTPAPPSPLPPSPLP
PSPAPPSPAPPQPPPAPPSPTPPSPAPPSPPPPSPAPPSPRPPSPEPPSPKPPSPEPPSP
TPPSPQPPSPAPPSPPPPSPEPPSPAPPSPPPPSPEPPSPAPPSPPPPSPEPPSPAPPSP
PPPSPEPPSPAPPSPPPPSPEPPSPAPPSPPPPSPEPPSPAPPSPPPPSPEPPSPAPPSP
PPPSPEPPSPAPPSPPPPSPEPPSPAPPSPPSPSPEPPSPAPPSPPPPSPAPPSPAPPSP
PPPSPEPPSPAPPSPPPPSPEPPSPASPSPPPPSPEPPSPAPPSPPPPSPEPPSPAPPSP
PPPSPEPPSPAPPSPPPPSPEPPSPAPPSPPPPSPEPPSPAPPSPTPPSPEPPSPAPPSP
PPPSPEPPSPAPPSPPPPSPEPPSPAPPSPPPPSPEPPSPAPPSPPPPSPEPPSPAPPSP
PPPSPEPSSPAPPSPPPPSPAPPSPEPQSSAPPSPEPQSSAPPSPVPPSPAPPSPAPPSP
EPPSPSPSPAPPIPAPPSPQPPSPAPQTPQPPSPDPPSPAPPSPAPPSPVPPSPIPPTPA
PPSPEPPSPVPPSPTPPAPQPPSPAPPAPQPPSPAPPSPNPPSPAPTTPASPEPPSPQPP
SPSPPVPPSPAPPSPAPLPPPSPDPPSPVPPSPAPPPSPPAPPSPEPIPPAPPPSPPEPP
SPAPPSPQPPQPPSPSPPSPAPPSPEPPSPIPPSPAPPSPAPPSPQPPSPEPPSPAPPSP
APLSPAPPSPAPPSPIPPAPPSPEPPSPAPPSPAPPSPAPPSPQPPSPAPPSPRPPSPAP
PSPAPPSPVPPSPQPPAPPSPAPPSPAPPSPAPPSPAPPSPASPSPVPPLPTPPSPAPAP
TTSPLPPSPTPALPAPPASPAPPPNPPRPPQPPAAPPPSPAAPPPSPAPLAPPPPQPQPQ
PTPAAAAPSPPLPPDCALLAQAALLSIPEAVNSMFVVAAGVPASAAASAPPELLASFCAV
CSCQLTATAISIVGGSSSSSTNGSSSSTGGSYYSTGGDAAIQRIAASNTTGRAGAAGSSG
NATSGWGAGTQTAAEWVVDAVQDGIYQLQLLIGGVSYTRTVVVDRTPPSVSGNVTLSARR
VKQEPSAVGEASLNALGSNQAMLLTIAFSEPVPTFDPATSLITSGALVTEWVASADKATY
YVLALTLPSEPAAGSSSSSSSSKSGGGGNGTAAAAAAPPPAALLGAAAGGRRRSLQQQQQ
QPQGGAPAPPPPPPATPATSGSGSLSDGGGGAAATTATRQQVHFRLPATAYADAARNPGR
NDLSLSVELSDGSLAPPAVGEALATTARVTAAAYPAVVASTTLVAAASSSFAQALRAKGS
LLQGSYHIQLLTMSLYLASRGVGREYGEYAVEFKYAVLGVKGNLGPAESALPTHEKQVTA
AEQAREVGGDLWPIGQDMLGGSSNTSSGSSSSSSSRYSPPRRPPPPPAGSAAGGLLFSSA
DAPPLAVAPTLPAPSPMAVAAASVAPPRVPAPPAPQPASLTTTGNSSSTGGLHRRQRQLA
QQALQPPPASASALVLQPPPPPPPPAQLLIQQASATYVSDMQDLLYTLVVAAMLITAVAA
GRLAAALAYRLLVSPQPHPFLAFPRLETTIVGLVLVALTFYSCMALGGPAADWHGSRTSA
YCVLAIGVVPYAVFLWWLALARAWMVPQYMLVEPLHMTVSSYASPRPSAFERAERTTPRA
SVVSITTAADIGGVPTGGGGGGGGGGRGGRS

>NR|gi:57472005||Chlamydomonadaceae_C.incerta||spap2-containing
MFQTMKGGWAGRRRLRRVAMAVAVAIAMFLPDLASAYGPWNATDDLNEQHKGLLAFILSG
DTSFWSRPEVATRLGFGTAPWRCISNCQTQIFTTDQQCAPDCESRTYCEPGGAALGSENT
TCCALSLLDQTYASSQPPSNTQPDWCSTYPGWGSRPRPSVCDFNVFAARGTTPPAGADDP
DLAVSCSTGTVPSYYISGTRYRANDTVYRIMHDGAVSSPANVTRNQVKRIKLRHSAMWHH
PSVNITSPPEFSLVSELACLPLEEIYLEDVALRADYNAFLAQPNTTLSNGMVFDLYNTTT
YPYNVSTLCLSVFCMLSLPDFMLHSFSTLEPASLNPDLAMRNTALRTCNFAPVTELHAMR
WTGQAQLDWSNEFAQALDFVRNGTQLRPRGWLPPVPEAVSGRPLLPDTLRRLSIRRTRDD
RHGAVQWTSRPLISGPLPAEWALFHNLEYLDLSDDMETGQIVGPIPSTWLMMTHLRVINM
TGHHNFCKDWHKLISWQIRSFYRSQTLENPLNVPHYYGPWGGNNKNMTRYNISVYDLNGH
GWQWYDETTATAGYVNVISPHGKCCWDTWSQTIKAHNADIYNPDGSRFGNHPNYDYTYGV
FYQDEEWCEPSTPPQPPSPPEPPSPPSPPNMPNVPPMPPSSPPAPSTPAAPPPMPPIPPA
SPRTPAAPPRPPLPPTSPGKWAGAWPFRAPFPPIPPSPAPPQPPPLPPPVASLPPPPSPK
PPKPSPRPPSPRPPSPIPPSPKPPTPAPPSPAPPRPNPPSPGPPNLNPPSPEPPSPLPPS
PAPPSPAPQPPSPSPPLPPSPAPSSPEPPSPAPPSPDPPSPTPPSPQPPSPIPHLPPPPE
PPSPEPPRPPSPEPPIPEPPSPAPLIPAPPSPAPPSPQPPSPVPPSPAPPSPEPPSPTPP
SPAPPSPAPPSPEPPSPTPPSPQPPSPAPALPTPPSPVPPSPAPPSPEPPSPFPPPPSPA
PPSPEPPSPTPPSPQPPSPAPALPAPPSPVPPSPAPPSPEPPSPFPPSPEPPNPAPPSPE
PPSPIPPSPQPPSPAPALPAPPSPVPPRPAPPSPEPPSPFPPPPSPAPPSPEPPSPAPPS
PQPPSPAPALPAPPSPVPPSPVPPSPSEPPSPFPSPPSPVPPSPEPPSPAPPRPEPPSPT
PPSPQPPSPAPALPAPRSPVPPSPAPPSPEPPSPFPPSPAQPSPAPPSPEPPSPTPPSPQ
PPSPAPALPAPPSPVPPSPAPPSPEPPSPFPPSPAPPRPTPPRPEPPSPTPQIPQPPSPA
PALPAPPSPVPPSPAPPSPEPPSPRPPSPEPPSPTPPIPPSPTPPSPQPLLPAPPSPEPP
SPAPPSPTPSVPAPPSPAPPSPTPPQLPPPAPSPQPPSQPPPSPALPSPHPLAPFPPSPQ
PPSPSAPAPPSPAPLEPAAPVPPGPPPQPPGAPAPPAPPPPSPAPPSPAPPSPEPRPPQP
PSPVPPAPPSPAPPSPVPPSPLPPSPEPPSPAPPSPAPNAPSPPSSAPPAPVPPSPGPPS
PQPPSPGPAPPVQLPPSPRPPSLEPPSPAPRPPQPSPSPPSNPSPPSRPPAPPPAPQPPS
PPLAPPPAPPPPAPPPSLPQPPGPTVTTQDCSSAAARTSFAVASASRGAFFIAVAVPGSS
PSYCQVCGCELSYAVLEAGAAQAYVIPSSYAGGGSPTAATTSSLSGTADAGSPSSAGANG
SASASGSLLKAEARHRTLVGMASGPTGDVTRYLMAASASTTDTNTTTDTNTTNTNTTNMT
LEDLLATGGSRSAGTGSSASRLQVVDVQTGALDPAGTGTGTGAYPTTNTTNGAGGEAGGG
GTVRYSAVGSGGSGGLDAAWRLTPGASGDYLLRLKVADQAWWRWVSVDMDPPRAAGQLLL
ARRSDGSNSSSTGSLAAAEAEVQAQVDAAHAAAGAAAVSAQAEVVRLVMAVITMSEPVQP
FSLNSALRLGGGARLISTQCFASATAAAQVAAAAGTVDADAPTQGTNDSSPASAPPANAT
AIGSTGLIGGDGVNAGYGSGSTGSGAATAVVQSCVALLFAVGGTSPELVLPPGALTDLRG
NTNAEPLILSAKLPTSVESLSTAERAGAPVAAAVMGGLFASAAFTSASASFLSAFSSRSS
LLQSGYHIQMLAMSSSLASPGISPAFRRIARYLRWSLLGIQGNIPLLDGAFSSSSGGATS
SGGSSSNSSSNSSGSSGLGEVDVAAVALDRLRLAVPPPAAPVTGGPPTASAQPLSSPPLA
AANGSAPIASRRSSRSLTQAASVSPSPPLLQPSAAAAATGATAPPDPVPPRPPRTPPSAP
PPLPALSLSSDRDALVSWLQQVGAIGGSSSSSSNGSSGTSGGNSSTAASVTAYSLGGAAA
SPRGDVVVLPDGRVVLGPGAGGGSAPPPPASPSTSGSDSDGAEMTTIHTNVQDLLYTLAI
AALLMVALVAAHLLLIGLWRLAVVYDVCGAAESGVQGLHPVLRFPRAEMVLGGLLLVALT
FYSALTLSGAASPRWGDNTAAGRLIAVLVLAVLVVPYGLLLWWLTVCRWYLQEEDIDDYM
LGPHWQAFDGIVPGGGGGAAGGDGGAEGGAGGGKAASGLPAAAGFGRSSGGGVAFAVSQQ
AEPGGGGDPAGSGGEGDFGLGPHWALAPAGAKTLDYESLAAPVAADAAAARNAGVGSTAR
AAGAASGAQSGKVRPRPLRSTDVPATATTAAGTATAGASTGPGGGSARDVGAAFQRRPGE
ADSDDDRDGDVDGEAGMLDMAPWRRRGLPADGAEDATPAASAAGTTGGRSDDGSRSAADE
RRRANRARKGSTDGAAVDDGDDDVALHSRPLPRRGMSYGDNLLTAGANTRGSALRSSPGP
DGDDAADTRTRIRRNVTYASGDNALTSGAASSSGRGAGPSSAADPDAPQAPPSPRRGAGL
GSNAYTAGASRFGGRPRQAADSPPPEPASPLSGGEVGAISEVGFNAMVSGGSRPPRSDSP
VMEDEASAIRLQQDAADPALLEAQRARRKKAGSVEEPQLALNPMAVPRASVLERPDVAAS
LAAAVAAKQGTVPLPPITEAAGAASGPGSGGPHDGRMMTTASVSHGRDTTSRLLHPMARP
RSLGASLVPASPGSGGSRGSNNGSTGAVPIAMPRPRVGGATSVPVARPDEDFVGELEAPV
ATSRTDVAAVSQSPNLPSPMMRLGSRLGLLVRSGSSSGAAQVVPSSPPPAKAASPPAAAA
RPRPPASVSTNAAVPMPPPQKAFRSPSEPVNNTHGAPAAATAVANGGGRELNPQQPIPIS
GNGSGNGNGSGSGGGNPLARALRYMSVPKPGAQPNTASDTPPPAAVAAAADTEPAPPSRH
ITAWGGGSISQPPAAPPSPPLAPQSPMRLPMPPPGAGAAAGSDFSPRASGRTAGGGPAVR
GGGSGGFTTRSPQASIVMETSPVGGPVWPPRRRRNRAAVIDEDGDDDEGGAAGPVAEGAE
DPERAGSLFGGWDEDPSLLLREAPRSPIMPPPPPLRAGTARGSSSAGGGMSAAGGSASYS
SRGGGGSGGGHHYMIHNPLSSPPLSSSAAGAAPLLLVSPPPMSSLLLPPRGFSQQAPGSP
RGMKLLQRSSTFAHGAGAPALGSAPTAVAMLGGYYSTRSLQSAVGEGVEGPLLPSAGALA
LVGGPEGGAAISAGGGRRKSVADLGAEEAEVLRQKQLAAALDDWGEVVDPGPGGPAAMVA
APPGVDKRLLPQYPGKELPMYRFRYRPGCWPLRLRLAPPVHFLARFEFLFEDAIGEGPQE
QQGRETPLILCVTAINFTHKALCAAVFGGFGLLERSVPQLGFLMGLQGAMLVYLAAVRPF
REALLQAVELTCHALEAGLFACALALVNARSNNGAATTYVMVACFFGVALLVIVYELRRL
VLLVLALWRMWRDRSTQQSRRASHAVIDEMEGGGVSAAAAAASRSPSALAPPVSVTARRS
SALKDGPVRGTGSFSDGGGGGGGAGVATSTPAVPQSPPSAAAMRRSRRSSAASDGAGGRQ
AAAAMAESSHIQEDAGPDAARSQASATAQPPEIVIERDHGAHPDAVAAPRGNRLPPITLP
VARPAAS

>TC|BU647440||Chlamydomonadaceae_C.reinhardtii||spap2-containing
MARSSASRPTMGALALLLVASCFVGGVFSARMAKLALDGKRGIDTWEGGCKQRLISDQGA
LATGSFRHVRLLSRSSPFLPSSLPPVPADSASVSDASASSADQQQELESVVSALAHMNAD
SEAMRRVLASGVYPPSPAPPSPSPSPPAPPSPSPPLPPGLALEAEHYVAGFSANATIVAD
LDVTSQASVDAFIAAFIQLTXNALTSTPPAC

>NR|gi:159468866||Chlamydomonadaceae_C.reinhardtii||spap2-containing
MVVSAAWRRPTGGGRCRLLAAVLLGAVVVMAAHGGPLGASAQEEKLGGTDAAVQFGAAPP
SPAPPSPSYPPSPAPPSPSYPPSPAPPSPSYPPSPAPPSPSYPPSPAPPSPSYPPSPAPP
SPAAHRPGAAHLLPVSAERKPCFKVFAWRKTLLYVQSEDRFTYNEAQEFCSDRGVFMVGY
DDPVKRLPLADLCYKNGNGCWVGGQVGDTCPYIDPKGNGASYPEKCERRHYAVCWGDLDK
QRNNPAIWDNNCPKNNKSQDSKKRDKNYD

>jgi|Chlre4|195673|FER_acegs_kg.scaffold_45000003||Chlamydomonadaceae_C.reinhardtii||SPAP
MRTSLWGHAQSGKMVFQFLTAAVVMVLATPAAAWHGSSSRYTAYPGYIINTPAIACWTGW
FTYTTNLTACLTPVEANGINNKADGLASICTNYVPGCLSFHLDTGMFYASNDIALLQASP
ADNVYLKSLSPPSPMPPKPSSPPSPTPPSPMPPSPAPPSPAPPSPLPPSPVPPSPAPPSP
APPSPAPPSPAPPSPRPPSPVPPSPAPPSPLPPSPAPPSPAPPSPEPPSPAPPSPEPPSP
EPPSPAPPSPEPPSPEPPSPAPPSPAPPSPVPPSPAPPSPVPPSPPPPSPSPPTPIITNG
QGTGQQQISSGGATPGPVSGAATLTTSAGNDDVVIPILAVLVPCVVLSMIGVFTWWMLRQ
RRLKAAATGVTSGAAAPQPQQGEGRV

>jgi|Chlre4|297436|au.g15031_t1||Chlamydomonadaceae_C.reinhardtii||SPAP
MARHTASAKALAVVTALLALSAATAAHARKTMATVGPRRQLPTADVCSTQQPSDYCNAFF
AQCATFTCSGNVVTLTLSNGGAICKDAGGYSWAACLKAGGAACGTQTNAQCSPGAATFKS
PGGTYCDSGASVASWTLDAGVTQCPLTNGGNGCYAGNSGCTVCEHIYNVDCSASSPPSPA
PPSPAPPSPAPPSPAPPSPAPPSPAPPSPAPPSPEPPSPAPPSPAPPSPEPPSPAPPSPA
PPSPEPPSPEPPSPAPPSPAPPSPEPPSPEPPSPAPPSPEPPSPEPPSPAPPSPAPPSPE
PPSPEPPSPAPPSPAPPSPAPASPEPPSPAPPSPAPPSPAPPSPAPPSPAPPSPLPPSPM
PPSPAPPLPNIASCVAPTSCPATSVMTAAGIPNAVAPYDRTMNVSCA

>jgi|Chlre4|196024|HAL_phc_0010||Chlamydomonadaceae_C.reinhardtii||SPAP
MGLSLSWAALVLAATLLVVADAQRQSGLYTNFPFCKCVARGRYSLDRVVDSVGAGQYCFT
IRVQTPAGCNGYCCTQADMHKLEFHAQPQCDINNGASVSATINGVPTRVGPAYDQPPDGP
PGGTILRLTQLGLTAATANGARLCIKLTAGRNGTGCGTLEQMCVPPAGSPPGVCTAAIFD
SGNGCCPQVQAGTPSPPSPLPPSPRPPSPKPPSPPPPPGPPPPSPAPPSPSPPSPPPPSP
QPPVPPSPAPPTPPGPPPPSPAPPNPPPPPSPAPPSPKPPSPEPPSPPPPPSPEPPSPKP
PSPEPPSPQPPLPPPPPSPYPPSPSPPSPPPPSPPPPSPAPPSPAPPSPPPPCECLAGER
ELTCAFLPPSPLPPSPAPPSPQPPLPPPPPSPYPPSPAPPVPPSPPPPSPYPPSPAPPAP
PSPPPPPPPPPPPPPPPPPFPPFPPPPSPPPPSPGPPSPAPPSPPPPPPPPPPSPYPPSP
APPLPPSPPPPSPYPPSPAPPVPPSPAPPSPEPPSPAPPSPPPPPSP

>jgi|Chlre4|404098|pasa_Sanger_mRNA13579||Chlamydomonadaceae_C.reinhardtii||spap2-containing
MAPRNGATVGAAVALAFVLFISGTAARKTALAAVDNPFATRRLQQSCPGGQSTFCQNMFA
SCISITCSGYQITVATTSASGCKSAYSWFGCVKNGGGNSCGSQPSKGDNAASYTWTLSPI
TQTTVGIQIHDGSFNGQGTETCPLTGGCVSPPPSPAPPSPNPPSPNPPSPAPPSPAPPSP
KPPSPLPPSPAPPSPRPPLPNIAACPAPSCPSTSQMTAVGIPANPCTPCTAQTECLKEST
DGQFTIPGSANNVLTVYNCNAKPQVAGAVPLVFSTTSGSSFPTLNAYLASSVVGQAAAVA
CYAPGDLSWTGPIVNYLYLAHVTPSACVCSTDTAWAVPLKPVMEGLVVGTGEQQLGAPYP
AGVYWTARSDATKANAWGGYFRITPIPHTSYVYEFDVCAGCGQNRVGAGFIMARLKFQIT
SANGQTSVTTFLAPSLTYPTYTPSPTASTTSSVLHMYQSFIAPPTLTPGQMTNSAKTTFS
LPITYGSSWTVTQVNTGTVKFGSTSVNVPATETANGLYVAIHLTVGGQFCY

>jgi|Chlre4|297157|au.g14769_t1||Chlamydomonadaceae_C.reinhardtii||spap2-containing
MGALALLLVASCFAGGVFSARMAKLALDDSASVSDASASSADQQQELESVVSALAHMNAD
SEAMRRVLASGVYPPSPAPPSPSPSPPAPPSPSPPLPPGLALEAEHYVAGFSANATIVAD
LDVTSQASVDAFIAAFIQLTATALNIDPANVYVTRVTKGGASSGRRRAALEQSGAAEHVW
SASELSALLPGAGLSLLALKRVTPADDATVAGLRSAKLELVLGPGAKLQQARRSAQQHQP
SSTADVIVDFTVVTLVEVALPPSPPPSPPLPPGVSAPPTAPVSPPSPPPRPPRPPPLNVA
VLTSALNASWVNEAPSPPPSPPPSPPSPSPPARVTSVRVSRMPAQGTLSGDSSKPVVWYN
DASFRSYRKPRSPLNLVFWSARRQCTATVGVCGGCPRAWAVPSNATQTTTTLPLYFREPM
QLDSITITQLQNPGVLSVELLPWPATPIPELPGVAPVSGPKGQPVYSAASDSTPCGGDLV
ISVPSDRSGSSESVAPRGSQSELPPRLRRTAVGGIRITVKAQAKGAKPTFISSVRFSGRV
LYPANPAAYDGM

>jgi|Chlre4|297158|au.g14770_t1||Chlamydomonadaceae_C.reinhardtii||spap2-containing
MGALALLLVASCFVGGVFSARMAKLALDDSASVSDASASSADQQQELESVVSALAHMNAD
SEAMRRVLASGVYPPSPAPPSPSPSPPAPPSPSPPLPPGLALEAEHYVAGFSANATIVAD
LDVTSQASVDAFIAAFIQLTATALNIDPANVYVTRVTKGGASSGRRRAALEQSGAAEHVW
SASELSALLPGAGLSLLALKRVTPADDATVAGLRSAKLELVLGPGAKLPQARRSAQQHQP
SSTADVIVDFTVVTLVEVALPPSPPPSPPLPPGVSAPPTAPVSPPSPPPRPPRPPPLNVA
VLTSALNASWVNEAPSPPPSPPPSPPSPSPPARVTSVRVSRMPAQGTLSGDSSKPVVWYN
DASFRSYRKPRSPLNLVFWSARRQCTATVGVCGGCPRAWAVPSNATQTTTTLPLYFREPM
QLDSITITQLQNPGVLSVELLPWPATPIPELPGVAPVSGPKGQPVYSAASDSTPCGGDLV
INVPSDRSGSSESVPPRGSQSELPPRLRRTAVGGIRITVKAQAKGAKPTFISSVRFSGRV
LYPANPAAYDGM

>NR|gi:20138131||Chlamydomonadaceae_C.reinhardtii||SPAP
MMRRQHAAPLVGAVNVLMVVLAFVASANAQCVPGGIFNCPPSPAPPSPAPPSPAPPSPAP
PSPAPPSPGPPSPAPPSPPSPAPPSPAPPSPAPPSPAPPSPAPPSPAPPSPAPPSPAPPS
PPSPAPPSPSPPAPPSPSPPSPAPPLPPSPAPPSPSPPVPPSPSPPVPPSPAPPSPTPPS
PSPPVPPSPAPPSPAPPVPPSPAPPSPAPPVPPSPAPPSPPSPAPPSPPSPAPPSPSPPA
PPSPVPPSPAPPSPAPPSPKPPAPPPPPSPPPPPPPRPPFPANTPMPPSPPSPPPSPAPP
TPPTPPSPSPPSPVPPSPAPVPPSPAPPSPAPSPPPSPAPPTPSPSPSPSPSPSPSPSPS
PSPSPSPSPIPSPSPKPSPSPVAVKLVWADDAIAFDDLNGTSTRPGSASRMVGEPDIAGT
KCKGNLKGWMPKPSRNPRWGQAVFSGGRTVGSVANVTIRVAFATEKPALIYSSIELVVYN
TGATLIRVPIAANVTRSQIRCPGFLTYGTTPIAGYPTGIDATTWPNWKIAGVRINMGAGN
KKPKTSIDAVGLNLK

>jgi|Chlre4|323341|pg.chromosome_1_#_130||Chlamydomonadaceae_C.reinhardtii||spap2-containing
MARPRRAGAALALLLLVAGAALMMMAPGADAGRQGISLASSNAQTLYQARRGLLACSLSC
SSLATCIKASCVSSDGVSTTVVKLDFTACKGSSISWYCCSAAATGAPGGCTTTSCNGVTI
AAGGYCDTVLTSTINVVYGATFVTLQVHDGQLSGNTDCGVQSTCAGCGGSCGDGVCSNVV
VPITGLAACTAKPPSPAPPSPKPPSPAPPSPAPPSPKPPSPAPPSPTPPSPAPPSPAPPS
PAPPSPAPPSPAPPSPAPPSPDPPSPAPPSPSPPVLTAANIPNMACSTCVPVGTCFVEAN
SNPQQFETDPARTVNGFFSAAGGCIAKPTGTYTYTDPLTGNTAATAPLYLMTDGTTQLQS
FLGYAVVGASKAIVCYNDTTYTTVSKYIYVERILTDTTNYACLSCSWFRLYTITKPSTCT
CRSDTAWAFPLKSVLDSWTLAQAASTTEILLPSGSYPGNVYWAARADTSAWGGYFRFAPA
TSSSPFKVGTTTTYTFDMCAGCAQNSIGKGYIMGKLSFVFTNANGAAATYVFFAPAPGAS
VTSSALQVYQSYISPPTFTPGQFKNFDTLSAVTIATGAVAYGSSTANVPTAMTSNGVYIA
VHMAVGGSFCDGNPPYNLVVPQ

>jgi|Chlre4|404311|pasa_Sanger_mRNA35235||Chlamydomonadaceae_C.reinhardtii||spap2-containing
MVLTTVACCALLRSAAGIALGSPPPPPSPPSPGSPSPAPPSPAPPSPEPPLPAPPSPTPP
SPEPPSPEPPSPAPPSPEPPLPAPPSPAPPSPEPPTPPSPPPTCRADPYLLYYHSITESI
SGPVRTSEPESRVLGTATLWPDYPVPGSVTLELEAAPGGSWTPGAVVHWALMTPLQYLND
VFTVPLPDLEGPPCHEPTSLPHNRTLTANDVSFFRVFTLTYAQLGVVDDCNPRALFPVVR
IDDGETSAWLAWDWVNPATGEGANICSGSPTAWGVGSFSLQSCPCQPPSPSPPPPAPPPC
GTTILSQESEPVASGYLSGPIRDTANGWEVGAATVHVEPSGPDAVTLSLSLPLPAGTVIK
YNFFDWVGYADAVFTLRPPALEGPNCHEPYDDSLLTAATVPAGTNEYNISVSYSSLQLSP
CSRKTFFPVVKITTPEGLTSWLAWDWVTPNDPENDVCGDSPTAWGVGYFDLYNCPCSPPP
SPPPPSPPPPPPPSPPPPPGSPPPSPPPPSPPPPSPPPPPPPSPPRQSPVPTAQLGTGFP
FCACRKRKPSLSPWRLGPLTGPTIAAPNSATEPTLATWCADITATAEPSPPTGVCAGMDL
FKIEFLVNHACYSSSPKAIRRVTVNGAQAFPTWSQKYIKGVYYGVFGVSRLAFTAADGPV
RLCLSFAAEAEGGCGTPAGLCYGSACTYSIFNKKQNCCPTSSISSSVDVRPGH

>jgi|Chlre4|391995|pasa_Sanger_mRNA30725||Chlamydomonadaceae_C.reinhardtii||SPAP
MVVSAAWRRPTGGGRCRLLAAVLLGAVVVMAAHGGPLGASAQEEKLGGTDAAVQFGAAPP
SPAPPSPSYPPSPAPPSPSYPPSPAPPSPSYPPSPAPPSPSYPPSPAPPSPSYPPSPAPP
SPAYPPSPAPPSPSPPSPSPSPRPPSPSPPSPSPRPPSPSPPSPSPRPPSPSPPSPSPRP
PSPSPPSPSPRPPSPSPPSPNPRPPSPSPPSPSPRPPSPSPPSPSPRPPSPSPPSPSPSP
SPPSPSPPSPRPPRPSPRPPRRSPRPPSPRPPSPSPNLRPRPSPRRRSPPPPERKPCFKV
FAWRKTLLYVQSEDRFTYNEAQEFCSDRGVFMVGYDDPVKRLPLADLCYKNGNGCWVGGQ
VGDTCPYIDPKGNGASYPENCERRHYAVCWGDLDKQRNNPAIWDNNCRKNNKNQDYKNLD
NKDNGDGKFNDNGGGSGRGNLNVQGNNGPDHQPCRLLAAVLLGAVVVMAAHGGPLGAAAQ
EEELGGTDAVVQFDARHVAVDKEHDKKSPEQSPSPRRRSPSPSPERRSPSPNRRRSPPSP
ERSPSPERRSPSPNRRRSPPPPSYPPSPPAPPSPPHPPSPAPPSPAYPPSPAPPSPLSPP
PSPAPPSPSPPGPSPPSPSPPGPSPPSPSPSPPSPSPRPRPPSPSPPSPRPPRPSPRPPR
RSPRPPSPRPPSPPPRPRPRPSPRRRSPPPPERKPCFKVFAWRKTLLYVQSEDRFTYNEA
QKFCSDKGVFMVGYDDPVKRLPLADLCYKNGNGCWVGGQVGDTCPYIDPKGNGASYPEKC
ERRHYAVCWGDLDKQRNNPAIWDNNCPKNNKSQDSKKRDKNYD

>jgi|Chlre4|284491|au.g2907_t1||Chlamydomonadaceae_C.reinhardtii||spap2-containing
MDQRIATFTSLPVGALTGSSLSAQGIELSAGSNPVSPAWGEVVDCAAGWSYGGGTIMTAA
GCTQALRFPYDDFSESFRPASPPSMFWSLSLTYRTTLPYVTLYLYPTSGQGNANACKVRL
NRTEPIASPRPPAFVYSASNSVTVTASECGWGAGSTFFSFSFYVGVEGSSAGIDVFELLL
DSFTMETGMAPSPSPSPPSPAPPSPEPQSPAPPSPEPPSPMPPSPEPPSPAPPSPEPPSP
APPSPAPRAFGSSSLTVSFDALPLGSLTLTSLEAQDIRLAHQDESNALPGWGQVVDCAAG
WTTDDSWAMTATGCNKTLRLTNALSDFFLPLSPPSTFWKLSITYRTTFAYTGLVLYDTGD
VSHMCVQALQTSDPTPLPNPTKFTYTAAPITFTMTPADCGWAAGSTFDRFAISIITFEAG
RDRVELLLDSFTIETGIAPSPPPSPAPPPSPEPPSPAPLAFGNTRAVAFSSLPVGALTSS
SLSAQGIELSAGSNPVSPAWGEVVDCAAGWSYGGGTIMTAAGCTQALRFPYDDFSESFRP
ASPPSMFWSLSLTYRTTLPYVTLYLYPTSGQGNANACKVRLNRTEPIASPRPPAFVYSAS
NSVTVTASECGWSAGSTFFSFSFYVGVEGSSAGIDVFELLLDSFTMVTGMAPSPSPSPPS
PAPPSPEPQSPAPPSPEPPSPMPPSPEPPSPAPPSPEPPSPAPPSPAPRAFGSSSLTVSF
DALPLGSLTLTSLEAQDIRLAHQNESNALPGWGQVVDCAAGWTTDDSWAMTATGCNKTLR
LTNALSDFFLPLSPPSTFWKLSITYRTTFRYTVLLLYGTGDVSHVCDRVLQRSDPTPLPN
PTKFTYTAAPITFTVTPADCGWAAGSTFDRFAISISTYLAFGDDRVDLLLGK

>jgi|Chlre4|327945|pg.chromosome_7_#_455||Chlamydomonadaceae_C.reinhardtii||SPAP
MYSPKAKPQAWGLAALPAVLLLLLAATLPTSTSAAGVRGVGSWGSGVGVGVGSSWGVRRT
VKRPSRNVVYCLRLEAKACTSQQQCCTLLANRVEALSLSMATTGPCNATSGVVSVTAGGV
QVERSLVYGSDYVDGSAADVSLVVRGLGAAADASGGIASMEVCYEVRAACAAWTDVCRPG
TPGAADNCRFAVNEADPSGDVACCNTCSANGLTFPAGGGTASPPPPPAPPPELPPAGPPP
LSPPPPDPPPPPPPDAPPPSPPPPPPPSPPPPSPPPPSPPPPDLPPPPPPEELPPPPSPT
PRLPSLLPPFPPPPSPPPPSPPPPEAPPPSPPPPDTPPPPSPPPPETPPPPSPPPPDASP
PPSPPPPSPPPPSPPPPSPPPPSPPPPSPPPPSPPPPDPPPPSPPPPSPPPPSPLPPSLP
PPSPEPPSPEPLAPPAPPSPRPPSPPSPAPPSPRPPSPPSPAPPSPKPPSPPSPPSPEPP
SPAPPSPPSPAPPSPRPPSPPSPAPPSPKPPSPPSPPSPAPPSPQPPSPPSPAPPSPLVP
SAPPPLPPAYPPLAPAYPPSYPPSYPPSYPPSYPPAYSPAPGLSPPAYMAATPPEPPSPE
PDTPPYPAGQKKPPSPPRPPSPPRNPSPPKAPRQPPRPPRPPSPPPSPPPSPPRPPRAPR
PPSPPMPDPPEPPSPPRPPRPPRPPPNPFPPPPSPKPPRPPRPPPLPPSPAPPPQPPRPP
SRPPRPPRPPSPMPPPTRPRRPLNPPRNPSPPRPPPRPPRPPSPPKPPNAPTRPPRPPRP
PSPPSPPSPAPRPPRPPSPPPQPPSPPSPEPPPLAPPNPPRPPRLPSPPPQPPRRPPKPP
PMPPGGISDGGGDDGYDSDPPSPSPMVEPPSPVTDGDEQDLEPPAGEVEPPPSPDVPAED
PDA

>jgi|Chlre4|344986|estExt_fgenesh1_pg.C_chromosome_80098||Chlamydomonadaceae_C.reinhardtii||spap2-containing
MARSSASRPTMGALALLLVASCFVGGVFSARMAKLALDDSASVSDASASSADQQQELESV
VTALAHMNADSEAMRRVLASGVYPPSPAPPSPSPSPPAPPSPSPPLPPGLALEAEHYVAG
FSANATIVADLDVTSQASVDAFIAAFIQLTATALNIDPANVYVTRVTKGGASSGRRRAAL
EQSGAAERVWSASELSALLPGAGLSLLALQRVTPADDATVAGLRSAKLELVLGPGAKLQQ
ARRSAQQQQPSSTADVIVDFTVVTLVEVALPPSPPPSPPLPPGVSAPPTAPVSPPSPPPR
PPRPPPLNVAVLTSALNASWVNEAPSPPPSPPPSPPSPSPPARVTSVRVSRMPAQGTLSG
DSSKPVVWYNDASFRSYRKPRSPLNLVFWSARRQCTATTTTTLPLYFREPMQLDSITITQ
LQNPGVLSVELLPWPATPIPELPGVAPVSGPKGQPVYSAASDSTPCGGDLVISVPSDRSG
SSESVPPRGSQSELPPRLRRTAVGGIRITVKAQAKGAKPTFISSVRFSGRVLYPANPAAY
DGIAPYTAILGCWDGTWLKIEGRSRLCVKCHDIRRVSVHAQHVTDSASVSDASASSADQQ
QELESVVSALAHMNADSEAMRRVLASGVYPPSPAPPSPSPSPPAPPSPSPPLPPGLALEV
EHYVAGFSANATIVADLDVTSQASVDAFIAAFIQLTATALNIDPANVYVTRVTKGGASSG
RRRAALEQSGAAERVWSASELSALLPGAGLSLLALKRVTPADDATVAGLRSAKLELVLGP
GAKLQQARRSAQQQQPSSTADVIVDFTVVTLVEVALPPSPPPSPPLPPGVSAPPTAPVSP
PSPPPRPPRPPPLNVAVLTSALNASWVNEAPSPPPSPPPSPPSPSPPARVTSVRVSRMPA
QGTLSGDSSKPVVWYNDASFRSYRKPRSPLNLVFWSARRQCTATVGVCGGCPRAWAVPSN
ATQTTTTLPLYFREPMQLDSITITQLQNPGVLSVELLPWPATPIPELPGVAPVSGPKGQP
VYSAASDSTPCGGDLVISVPSDRSGSSESVPPRGSQSELPPRLRRTAVGGIRITVKAQAK
GAKPTFISSVRFSGRVLYPANPAAYDGM

>jgi|Chlre4|393869|pasa_Sanger_mRNA5974||Chlamydomonadaceae_C.reinhardtii||spap2-containing
MHIQARGSVQLLVVVLLLLAHAHSSAADFAGYPYCTTSPTGTCQPCEVSSSGSSDVPCTT
GDAFGDDPAICTAYFSPNYPNNFGGADKCATTGGGSSTAWRAEVFAADPAGGGAPDIRVG
ELQAWEDTSGYMVVGFMPQCPYVLDLSAGAGGPLTISITDTAISGGATQGSWSTSIGSVA
AAAAAVAGGGGVPTDGGGNVPYLSCAGWRFQIRGTGAGQLAGAACQPYKRTVTLTAKLVT
VAPAGTGVCGVLSSVSSNISLSLTYVYSAGLSPPPPTPPSPAPPSPAPPRPPVLLLTQTT
TVSDADLEAAAAAAAATGNGSSSDSSSSTGAVSSAALAALFPGASNVTVTGRSVTFPIGL
VQLAVLPVSISTGGSGGGTIDVNGGTSTTTPGGANGGTSTPTTGGANDLCPGELAALARS
IRSELAARLGLNDSQVSRISCTLDVSPPSARRSLQQQPQGAPPPSVGSAAQQLCDAGGGG
GAGGGGNNRTAVAINVELTLPPDTNVTRLRTSIQEVVAALSAAPQNATAYTGAASSWVVC
SPPPSADSIRAVAGVAVTYAVPLTAEGAAAYAAQCGGDTSSGTTSSSTTSSSTAAAVTPL
QLSSSLTCALVPVAGGPTVPSDRDLMAAPPGVGGNTYAGGPPPAGDAGSGGGRSGIDRSL
AVILAGMAVGGAILITCSVTALVLVLRRRERRLEEEARRKRLREEGGEAAAAAAAAAAGG
GRGLQAKSPGGLEGSGGAMTPDRHTPGGGGGGGGAAPWTTAGGGLVTAGAAGAMHYGAVP
YSGSSDSSRLDGGGGVGGGSNQLAVQGGAAAAAAGQQGRTGVSRSGAAGKSVSLSGVVLQ
PAEPSIHAAALDAALAALVGGGRGLQWPGGPDGPASAAAVLVSSSNQQQPQPPAQPSPPP
RRSPNPFHLASQGPRLSGDGRSSLVNGGGHAQLASPSFGAGPGLPPGVGANGGARSAAHL
PWGANGGAGAAVPPGGDAGLGGSGSGSGGRSGGIGRWSSSGVMPAGAGAAAGAGAAMHPS
SQQPSRLRLASGSDALVGGGPALLLPTGSGSLGVMAGGAAGGGAVRSYRPALASPAATSN
PWRSMPIPSDPTADVSDVETLSQQGGSSGDEAGSLPIRLGSPSLAGAAGATAAAAAAAGV
RRQRTSGGLASPAAAASTPAGGGRSAVRRVLSSASRMMLGRSAGGGAAADGLPASGPQSA
SGASRHVLTTNVAFEPGPQQPQPQATAAVGAVAPGSAARAVPGSADVAAGTRRTKRVTLA
PAADPPAGGSAGTHGGSLPRRLHSHSGTSAAGAGPRSATVVGGMGVGPVTFSPGARAGAG
AAGVAPGRTGPGATGRAATDTGLLATGGQALPRPRPPASGLVEVGGLPGSPRRASLVMYE
QAPSPSR

>jgi|Chlre4|408398|pasa_Sanger_mRNA16565||Chlamydomonadaceae_C.reinhardtii||spap2-containing
MIRGWTARATAALLLLSAFGLAVGAASGPDDQAFNLRTSWFSGRKLTQSLPPVCVHNTTD
FTQVFPPDATGPADTCVDQAACLDIYYDTSTCKTTGGYLYCQVCIFWNQGRYNRCPKDVT
NTLSHTCRGDELDPGITAFGGAPQAQGGDTAKLNGWPSGVANRYCQWVRWLGEDTNPVNV
RFTVKDGSQSCGTATGNFKINGMTATCQPPRTLSSGAIAGCGRGDQANECLWTFSVPRIP
VCNTKSPPPPPPRPPPPPTALRPPNNKGGYACVSSDDADDAGHCQGTACGSLYINMIRKA
LDLAPIDADGILAVGSFTNGTSARSSLEEWVKAAGVSVSKITYADTAAKVAAANLTAFKL
LYVPSSYLQTNGGITDALNDALIAIKGKIADFVNLRGGSMIVLAQQGLLNGKSYGFFPTN
FTYVETDFYDSNNQPEMELVSPSTDNVNIDHRFWHGYFTGPQDWNGLRVVSYVANQCPTP
QGRGQKCNATVLCNLKTFLTKENCYNNIDDDGDGLIDKADPDCIRCGDGIVDPGEDCDDG
NMLDGDGCSSVCKFQAFAPPLRAPPPPFMESNDPDLDYCYQSSNAGCATCAGACETVDGF
WGSARACKLPKKLKGGLPDANVCKNDMGGLAVRVEDLYVTDNAGAVSSIVGQVHMFRTTG
GILTATVRMGCPQLLYSDASNTTDINQFMLVSYTAGGRFVSKTVPMVRPDVGKKRYACYS
FEYDMNEIVPGIGCTLVDMDVRITVRSSFTTSMVGSTTCSSAPDKLSQTTTMTERTSFLV
APQLPPDMPPSPQPPSPPPPRPPSPPPPPPPPSPSPPSPPPPSPSPPLPPSPPPPSPPPP
PPPSPPPPPPPSPPPPSPKPPSPLPPSPPPPPPPSPPPPSPPPPSPPPPNPPPPQPPSPL
PPAPSPPPPSPAPPLPPSPKPPAPPSPAPPSPGPSPPPPSPIPPSPVPPSPAPPLPPPPS
PPPPSPPPPSPPPPNPPPPPPPSPVPPSPKPPSPPPSPVPPSPAPPSPKPPLPPPPSPAP
PFPPSPPLFPVPPSPAPPPLPPVPPSPAPPGPPPPSPPPPSPPPPSPPPPNPPPPPPPSP
EPPSPKPPSPPPPPSPEPPLPPSPPPPSPVPPSPEPPVPPSPAPPSPPPPPPPSPEPPSP
KPPSPPPPPSPQPPLPPSPPPPSPAPPSPEPPVPPSPAPPSPPPPSPPPPSPPPPSPPPP
NPPPPPPPSPEPPSPKPPSPPPPPSPEPPLPPSPKPPLPPSPAPPLPPSPDPPSPEPPSP
EPPSPGPPRPPSPRPPSPEPPSPKPPVPPVPPSPAPPSPRPPSPNPPSPRPPSPMPPSPP
PPPSPEPPSPEPPSPEPPSPAPPSPEPPLPPSPAPPRPPSPRPPSPIPPSPAPPSPEPPS
PEPPFPPSPPLFPVPPSPPSPPSPPKPPVPPSPAPPSPPPPSPPPSPKPPSPPPPPSPGP
PSPPSPAPPSPAPPSPEPPVPPSPAPPSPPPPPPPSPEPPSPRPPSLPPPPSPSPPVPPS
PVPPSPPPPSPEPPSPEPPTPPPPSPPPPSPPPPSPPPPNPPPPPPSPEPPLPPPPSPQP
PLPPSPPLFPVPPNPPSPPMPPAPPSPVPPSPPPVPPSPEPPAPPSPEPPSPEPPSPAPP
SPAPPSPHPPSPRPPSPVPPSPPPPSPEPPVPPSPEPPLPPSPAPPTPPSPEPPLPPTAC
PAGAPSAPARPALAAAPAVSPSEPPSPAPPSPAPPSPHPPSPRPPSPVPPSPPPPSPEPP
VPPSPEPPLPPSPAPPTPPSPEPPLPPSPPPPNPPPPSPPPPNPPPPPPSPEPPSPPPPS
PPPPTPPLPPSPAPPQPPSPEPSPPPLPPSPRPPSPAPPSPEPPAPPPPSPPPPNPPPPS
PPPPFPPPPGPPPPSPPPPSPPPPPSPTPPSPEPPLPPSPTPPTPPSPPPPPSPAPPSPE
PPSPSPPPPSPPPPSPSPPSPPDAPPPSPEPSSPPPSAPPPPSPPLPPISSSCDCATKLF
APTNGACPDNHVILNFKAGNADRFDTTTPVRACVPRLGSNDDNTMYTFAFCWSTSCPVLS
PLTEAMSTVGLRIYVDQMSVLPAVADRLADKAVVFTNLHPTCAAPTSTLTLLNGLGTYKI
EASQSLRQIPAPGLDTLDVDVQAARNCDNEWVMVAMVPVHDIGADVPAPPPAACPCWQRT
YDGTCPGIKVPITLSIEGAVDPHLFPPSTTNTTIQQCIDPANFDVYTGRMAYSFCWRYAC
LPPVFASYPFRAVCKDLERWNIAKMEGEFLLQLQRTEGLEMFVEGKTKDPVLAGMAVVNY
TRNDDLTFISIPAGGVGNMTFTYVIPPGFDDLSAAVVMDSKPRPPPPPSPPSTRLTTCAC
PLTPPDTVGAPDAITCNGTYATMTARIANASTPEEQAAIAYQCVPWPNYDIFMKELAWSH
CWRYDCLPSIFFGKPYTLVIDRVTKHNIADIPAAGYELITFPAPYGMTMNVVLYGAADGA
AMEYNSTQGGLVVNGRLQVIQVPKLQSLTVQLNVPNIYGVDGMASWQQGRRDGTRGQGGE
EAAAVMLRYIEGSEMPLPPRPPSPPPAPPSPPPMPEGVPRVLITTDFMVTAKVPVETLIN
SSATITHSGYYVTVPIQYYSFEQLTDCSDASVAAYKQLVATTLGLNDTSTITVSCRFATN
TAGQAIARRLMAAATWVMESLGLRSQPGPLERVRRQLQTGTGAGASGDAKLDVTLTYGAP
KTVNPADSATTNCGVMMTAWGASCDASKVRAATRVTIRSEKADTSQSAVASACSSAAAEG
TAALLAASPTLSLNDLETMACQTAIVTTSGTSSNGNGNGNGGNGNALPPPPPAGAANPPG
ASGMSPGAIAGIVIGSVAGVVIVGFVALAAKNKIQQSRSGTQFVDPNSGRPGARRWRTYS
MQERAEEAVRGSRPVAGVSVYT

>NR|gi:41400384||Chlamydomonadaceae_C.reinhardtii||spap2-containing
MSRATHSSSSACPAGRLRLSRARMVRASVARNRPLQTGSLAWLVLLTFSLAWVTIPVSDA
LQFPVNVSVTSSAATDAPSAIAYIGALLDVLWNVPWQPNCINDTRRSYDAPWPSRCALPA
VQYGYYDETYIDATVYSGNSLRPFYSTCRYPASSDPYAFLEPYSMPWVDDFGIYQPVLDG
FDMWWGFTVNTVDSNLKWIDPAWTAQGAWLGGQDFRDAVWNFGTHYCSWPFVECSSCENY
DIADPYDPDKIANGIVPAVITALDFRNASLYIYYLDFGMFPAGSLDNVWYLNLAYNFIGG
PLPANLPTLLPSLQHLALDHCRATPDVRGTASLQYGWQQYPSTGQPYEYCSDGDVTGSDG
TEYVISGMIPDEWGDAVAAAASDSSLPWANLRTVRLSNQALYGPIPEGLRSASSSISSWR
LQGNTELCGPLPEFAAPINSLLYLGTKLGTHTWVWHDPADHTQGGECLAPPPPPSPPSPR
PPRPPPLPPSPPPPLLPPSPPVPPPSPPSPPSPPPSPPEPPSPPPLPPSPPSPTPVARCI
QVGGICDSPSPMPPSPRPPQPPSPPPPPPRPPPRAPRPSPPFHPPSPDSPPASSVPPSPE
PPSPKPPSPAPPSPAPPSPPPPSPAPPSPAPPSPAPPSPQPPSPVPPQPPSPVPPSPKPP
SPAPPSPVPPSPAPPSPAPPSPAPPNPAPPSPAPPLPPSPEPPSPAPPSPEPPSPAPPSP
APPSPAPPSPAPPSPAPPAPPSPEPPSPAPPSPEPPSPAPPSPAPPSPAPPSPAPPSPAP
PAPPSPEPPSPAPPSPEPPSPAPPSPAPPSPEPPSPSPPAPPSPEPPSPAPPSPFPPSPQ
PPSPEPPSPAPPSPVPPSPAPPSPAPPSPEPPSPEPPSPAPPSLEPPSPAPPSPAPPSPE
PPSPAPPSPAPPSPQPPSPEPPSPEPPSPPPPPSPAPPSPAPPSPEPPSPPPPPSPAPPS
PAPPSPTPPSPVPPSPAPPSPDPPSPAPPSPDPPSPAPPSPAPPSPNPPSPVPPTPPSPG
PPSPEPPSPAPSPPPPTPPTSPPPPPPEPPSPEPPSPAPPLPPSPEPPSPAPPSPAPPSP
QPPSPAPPSPAPPSPAPPSPAPPSPEPPSPAPPSPSPPAPPSPEPPSPAPLLPPSPDPPS
PAPPSPMPPPLPTSPPSPEPVPPTPPPSPPAPPSPAPPSPQPLEPPSPEPPSPAPPSPAP
PSPAPPSPEPPSPEPPSPEPPSPAPPSPAPPSPAPPSPAPPSPAPPSPAPPSPQPPSPAP
PSPEPPSPAPPSPAPPSPAPPSPAPPSPAPPSPAPPSPEPPSPAPPQPPSPVPPSPAPPS
PTPPAPAPAAALPPLPPSPAPPLPVPPASPAPSPSPLRPPQPQTPAMPPPSPAPPSPAPP
SPAPPGVPPPPPTPTPPLAPLPPDCTLLAQAALLSIPDAANSVFVVSAGLPTSVAPSTPP
ELLASFCTVCSCQLTATAISLVGGSSRGNNTNGSSSGGNYYSNGGDAAIQRIPAGNSTDR
GGSSSGSGGSSSWGPGTETAAEWAVDAVQDGTYQLQLSIGGVTYTRTVVVDRTPPSVSGN
VTLSANRIKQEPSAVGEASLNALGSKQAMLLTISFSEPVPAFDPAASLIVTGALVAEWVA
AADKMTFYVLAMTLPAELVATAAGSSSSSGTSRSGNGNGTAAAAAAAAPPPPAGTTGRRR
ALQQQAAAPPPPASGSSSSLSGAATANQQQRHVHFLLPATAYADAARNPGRNDLSLSVEL
TDNAVASPAVGEALATTARVTAATYPAVAATTTLVAAASSSFAQAIRAKGSLLQGSYHIQ
MLTMSLYLASRGVGREYGEYAVEFKYAVLGVKGNLGPAEAAMPTNEKEVTAAEQARQVGG
DLWPIGNDLLGGSSNTTASGSSSGSSSSSNSPPRRPPPPPAAGSTGLLFSNADASPPPLA
VATPALPAPLPSTIAAAATAAPPRLPSPPPPAVGSSTGVLPRRHLMQQMLQPPAAAVAAP
PPPPASSSALVLQPSPPPPPPPSQLLIQQASATYVSDMQDLLYTLVVAAMLITAVAAGRL
IAAVLYRLLVSPEPHPFLAFPRLETTIAGLILVALTFYSCMALGGPAADWHGSRTAAYCV
LTIAVVPYAAFLWWLALARAWMVPQFTLVEPMTTSSYASPRPSAFERADRTSPRPSTVSI
ATAADIGGTATGGGGGAASGACADVGAMAAPATVAAGADVPSEDDRYARGPHWKQFDGVL
PPTVASGVGGSGGGVVPLPPLVALPGSGRHLPLPPLLPAAGAATAAGGGAATAPGSPRGA
AAGEDTHQFGPHWRKFSVPDDGAATAGVGVDAAAGGAGGGGGRGSRGGSTDGVRFANGTR
TPSDDGAKGRSSSHGGGNGGNAMSSGAASFGASAGGGGAAAASRLGSRSGSPGDGDYNAM
SSGAAMRGGGAGASRFGPAGSRAGSPPEGAGGYAGGANAMFAGTAGRAGAISPAHDGSSR
PGSGSNEGGSRQMSYGSNNAMTAGAVGVGGGVRRPAGSLRNASDGDAPPPLPAPGTVPLP
FANLLTSGADVRSRRALNAEAVAAAAAGGGGGGGTSRSGSGNSNGSRAIGLASVPGTADR
QSLNVLPEEAAAVASQMPGFQKEQLVASGGSGGGSVAGSVVGGSVGGASRRVLAASASRP
GSLSGLAGGGIAAAGPVNGGAVRPRVGGMSLGGGGAADVNDAPGSPRQPRPRVGSTPTNV
PAAVAASAGAPRPPRITEVDEMGIAVVSAAEVAATAAASSSPPSPPLSPPMAGKRGFFLP
TPPPASLQAAAAAATAAANASAAAAERQAAGTVGMAPLPSTASARAAAGAGGGASLQRSR
TSLAPAASSEPVYGDGLGGAAGMLARRARVRRMVTLGDAASPGGDGSTAAAAAADRRGLR
FGSVPVATPSADAAAAKPPQKQARSNGWLNNDAGGSAAAGPVAEATEEDPAAAGGGAAAG
AAGAWRTRYASQLAAAGGAATDTTDSDGEGTQPWNRDSFRRRDRDAAAAAAARRRGKAPL
PPGADPHQFSGGAAIISTAAGEDLFVLPDADADAAARRKFSSHEDIVLQTDGGGGAAAAG
GGGRRSVALVTGYRYSRGYGRDGGGGCSRLLLPSPRLMAMFEFLFEDMLGSEPEQQLTRA
QRPARLVATALNFTHKAGCAAALGYWGLREQSWAQLGTLLGLQVVMLVYLLAVWPYAEWQ
LAAMEVVCHAAELGIFVAATAVANRFVHSSAATFAMIALFFLTLAAVLLYEVRRLYLLLK
EVWAVLRTKIASCRGSGAGSGGGGDGGGGCFGRFGGRGGGAAAAQEQGGKNAFADVHPPA
AILSAPQPVLLPQLQAAATAAGGDAAAAVASGPMGGMEAAAAAAAAQTRHPSVGNATTGA
PASATAAPAASATAAPAASATSATAAAVAATATHAQAATVGGRASTSNV

>NR|gi:41400381||Chlamydomonadaceae_C.reinhardtii||spap2-containing
MMLRKSTGKAGRHDLRRVAMALAFATLFFLPDLASTQTTYGPWNATEDLNEQHKGLLAFI
LSGDTSFWSRPEVATRLGFGTAPWRCISNCQTQIFTSDQQCAPDCESRTYCEPGGAALGS
ENTTCCALSLLDQTYASAQPPSSTQPAWCSTYPGWGARPRPSVCDFNVFAARGTTPPAGA
DDPDLAVSCSSGTVPTYSISGTRYRQNDTVYRIMHNGAVTNPANVTRNQVKSIKLRHSAM
WHHPSVNITSPPEFSLVSELACLPLEEIFLEDVALRADYSVLMAQPNTTLDNGKVFNLYD
PSTYPFNVTGLYNNVFSMLIEPSFMLHSSVSVKASSFNPGLALRNAALRTCNFAPFTELH
AMRWTGQAQLDWSAEYAQALSYVRDGSQLRPRGWLPPVIEPVSARLLLPDTLRRLTIRRT
RDEQHGAVQWTSRPLITGPLPGEWALLRNLEYLDLSDEMETGAIVGPIPSTWLMMSHLRV
INMTGHHNFCRDWHKIISWQIRMYYRAATHEPNLNVPHYYGPWGGNNGNMTRYNISVYDL
SGHGWQWYDEVTTEAGFVEVIAPHGQCCWDKWSQTIKDNNYEILYPDGSRFGNNVVQDEI
YGGFYQDEEWCEPTSPPQPPPPPAPPSPPSPPTTPDVPPMPPSSPPAPVMPPAPPPQPPI
PPASPLTPAAPPRPPLPPTWPGKWEGAWPFRPPIPPRPPRPPPPPLPPSPPLPPVTPSPP
PRPPPPKSPPPPKPSPPPRPSPPPRPPPRPLPPSPPPPPPLPPNPPSPAPPSPPPPPSPS
IPPPSPGPPSPEPPSPAPPSAAPPSPMPPSPAPPSPDPPSPKPPSPVPPSPLPPSPEPPS
PVPPSPPPASPEPTSPAPPSPPPPSPEPPSPAPPSPPPPSPEPPSPAPPSPPLPSPEPPS
PAPPLPPPPSPEPPSPAPPSPPPPSPEPPSPAPSPPPPSPEPPSPAPPSPPPPSPEPPSP
APPLPPPPSPEPPSPAPPSPPPPSPEPPSPAPLPPPPSPEPPSPAPPSPPPPSPEPPSPA
PPSPPPPSPEPPSPAPPSPPPPSPEPPSPAPPSPAPPSPEPPSPAPPSPAPPSPEPPSPA
PPSPAPPSPEPPSPAPPSPRPPSPEPPSPVPPSPPPPSPEPPSPAPPSPPPPSLEPPSPA
PPSPPPPSPEPPSPAPSPPPPSPEPPSPVPPSPPPPSPEPPSPAPPSPAPPLPLPPSPHT
QSPPSPVPPSPAPSAPSPPSPQPPSPLAPSPPSPAPQAPSSPFPPPQPTAPTAPPPPFPP
SPAPPSPTPPSPEPPAPQPPSPTPHAPPSPEPPSPTPPSPLPPSPEPPSPSPPSPAPSVP
SPPSPAPPSPMPPSPAPLAPQPPSPTPPSPAPPVPPSPEPPVPPGPDPPLPPSPTPPSPQ
PPVPPSPTPPSPQPPSPAPPSPAPSAPLQPSPDPPSPQPPSPAPGPPPSPPSPPSTPSPP
SPAPLAPAPPVPPMAPQPPSPPLPSPPFPPQPSPTITPASPQPAPAAAVLDCSAAATRTS
FAVASSSRGAFYIAVAVPASSPSYCQVCGCELSYAVLDPGASQQYVIPSSGSSSTAGGSP
TVAVTSSVSTPAGAGGLGNGTHGSTARRRALVVEATASSSGPAAGVGARHLLLAATANST
TLEGLLATGRSRSAAGAGMGMSRLQVVDVQTGGLDPVTAAPPTGTSPNTTSGAGEAGGSG
TVRYSSMGAGGSGGLDAAWRLTPGATGDYLLRLKVADQEWWRWVSVDIDPPRAAGQLLLA
RRTGGSSSSSNSTSGSGALAAAEDEVQVEVNAAHAAAGATAVSAQAAVVRLMMAVIAMSE
PVQPFSLTSALRLSGGARLLSTQCFASATAAAEVAAAAGTVDASTPPGSDASSSAATVAP
AAIAPVSGSTSGTSNSSTSGSAYVQSCVAVLFAEQDATPELLLPPGTLTDMHGNINAEPL
ILSVNLTASADSLSTVERAGAPVAAAVAGGVFASAAFTSASASFLSAFSSRSSLLQSGYH
IQMLAMSSSLASPGISPAFRRISRYLRWSLLGIQGNIPLLDGAFSSGSAAAGSGSGSSSS
NSSSGGLGDVDVAAVALDRLQLSVPPPLPAAGDAASQAQPPANLSPPPSASQLVADGSTA
LAGRRSRSLVQAAAPVAPSPPFTQAPPTPAPFGTTGAAPAPPPAPPRQPSTPPPAPPPMP
ALSLSGDRDVLVSWLQQVGAIGSGSNSSGGGSATSDSASVGAYSLGGAAASPRGDVVVLP
DGQLGLLGPGGSAPPPMQPGSGSGGQQSSGASVATDTTIHTNVQDLLYTLAIAALLMVAL
VAAHLLVIGLWRLAVMYDVCGAAESGVEGLHPVLRFPRAEMVLGGLLLVALTFYSALTLS
GAASPRWGDNTAAGRLIAVLVLAVLVVPYGLLLWWLTVCRWYLQEEEVDHYMLGPHWQAF
DGVIPGGGAGAGGDGGGHGASALPAVAGFGGTGGGGGVAFAACQAEPGSGDGGSGGGEDG
YGLGPHWALAPAGAKTLDYESIASPAAGAGSAAGATRTDAAGSTVGGAQSGKGRPRTLRS
TDVTAGGAAAGTAAAAATAAAAAAGAPAGAAGGDVGAAFQRRYGEADDNHGDSYDGEDGE
GDLDMAPWRRRGPPADGVDAAAPATAARKMAGSDDGAAGERRRANPHARKGSADGADGGA
GDPQAAGRPLLRRGVSYGDNLLTAGANTRGSALRSTPGPVADDDARSRFRRNVTYAAGDN
ALTSGAASAGAAPSSPTRTQPDSPQAPLSPRRGAAMGSNAYTAGASRFGGPRTRADSPPR
AASPPPEPASPLSGDAGAISEVGFNAMVSGGSRPPRSDSPTMEDEAAAIRIQQDAADPAV
EANRSRRKQAGSVEEQQVAINPMAVPRASVLERPDVAASLAAAVAASGAPPQGTVPLPPI
TEATGPNGLGSSPRDGRVMTTASVSHGRDTSRLLHPMARPRSLGASLVPASATTNGGSGN
SSGNGRESGSGSTSGGAPAAMPRPRVGGAPSNASAAAAAAAAAAAAQPAAHQDLFGELDT
TVLTSKMTADAGAASAPPNLPSPMMRLGSRLGLVRGGSNSGAAQVAPASPLPAKAPSPPA
AAARPRPPGAVTTNAAVPMPPPQKVFRSPSEPANARAQGAAPPAAAAVANGGGRELKQQP
AVQAVSSTTSSIRSNSSGSGNPLARALRYMSVPKPGAQPSTASNAAPAAAEAEPAAPPSR
HTAWGGSLSQPPAAAAAGAPPSPPIAPQSPMRLPMPPPGAGGAGGAGAKAGGGFTRSPQA
SIVMESSPAAGPVWPARRRNNRAAIMDEDDLVAEGGGAAGPVAEGAEAPDSAGHYFGGWE
DEDPRLLLREAPRSPVMPPPPPLRAGGAARGSSTGGMSAAAVSVHSYSPSSSARGGGSTA
AGHYMTYNPLSLPSAVSAAAEAAAVTPSALASPPLSLSLLPPRGRSQQAAGSPRSVLLLQ
RSSTFAPNPTAMAILGTRGGGGVGGCSTRSLQSAVGEGVEGPLLPSAGALAVVGSPKSAG
GAISVGGGGGGRKSVADLDAEEAEVMRQKQLAALDDWSEVVDPGPGGPAGAIAAPPGVDK
RLLPLYPGKELPMYRLGYRPGCWPLRLRLTPPVYLLARFEFLFEDAIGEGPREQHGRETP
LILCVTAVNFSHKVLCAAVFGGFGLLERSVPQLGFLMALQGAMLVYLAAVRPFREALLQA
VELTCHALEAGLFACALALVNAQPNNGAATTYVMVACFFGVALLVIVYELRRLALLVMAL
WRMWRERGTQARRASHAIIDEMEGGGVSAAAAAAAAASRTPSALAPPVSITARRSSALKE
GAVRTGVASSLSDGGGSTPTSPLGAQPQPSAAAPTRRSRRSSAAGGSSSSDGGTAGRQAA
AAIAELAQGQDGPGGDAVGEAATAQPETSTAAGEIAMRGATATGAGAAR

>jgi|Volca1|98666|fgenesh4_pg.C_scaffold_75000020||Volvocaceae_V.carteri||spap2-containing
MPFILITGVWLLSLSSLASGSHPKISSCGAVPVEAAASGAANTGSPASSPAPPSTTPPSP
APPSPAPFPLISPSASAFEKDTAKLAVLIAAAVSPKLGQGPPRKDPHLPPSPSAASSRRL
GFAQQAPRRRGAPASSSSSSSKTSSPSPSPRPSISSSSSNSNISVGSMEAAPFDDQAGER
PSYMRGTAASRAKASSTREPSRLGCSSAKLSSSSGAGTGPGGRALMSGAYRRSAQY
