# Supplementary material for: Global Analysis of Proline-Rich Tandem Repeat Proteins Reveals Broad Phylogenetic Diversity in Plant Secretomes
Source: PLoS One. 2011 Aug 2;6(8):e23167. doi: 10.1371/journal.pone.0023167 (PMC3149072; doi:10.1371/journal.pone.0023167)
Supplement: Text S4 — Additional genome sequence data. (DOC) [file pone.0023167.s027.doc]

**Text S4. Additional genome sequence data**

After building the TR and TRP taxonomies (see *Materials and Methods*; Tables S3-S8), several additional proteomes were downloaded, and positive hits to the taxonomy were determined by scanning the TR content of each protein sequence using the 38 regular expression queries we defined (Table S11).

Sequence data from the completed genomes of two model eudicots, *Arabidopsis thaliana* and *Medicago truncatula*, were downloaded from The Arabidopsis Information Resource (TAIR9_pep_20090619, downloaded 12/16/09 from ftp.arabidopsis.org) and the Medicago truncatula genome sequencing project website, respectively (Mt3.0_proteins_20090702_NAMED.fa, downloaded 12/16/09 from www.medicago.org).

To include genome sequences from lower plants and green algae, additional protein sequence data sets were obtained from the Joint Genome Institute Genome Portal (http://genome.jgi-psf.org/). The following data were produced by the US Department of Energy Joint Genome Institute http://www.jgi.doe.gov/ in collaboration with the user community (downloaded 02/10/10): Chlorella sp. NC64A (Chlorella_NC64A.best_proteins.fasta.gz), Chlorella vulgaris C-169 (Chlvu1_best_proteins_3.fasta.gz), Coccomyxa sp. C-169 v2.0 (Coccomyxa_C169_v2_filtered_proteins.fasta.gz), Micromonas pusilla CCMP1545 v2.0 (MicromonasCCMP1545.FrozenGeneCatalog_20090403.proteins.fasta.gz) [1], Micromonas sp. RCC299 v3.0 (MicromonasRCC299v3.FrozenGeneCatalog_20090404.proteins.fasta.gz) [1], Physcomitrella patens subsp patens v1.1 (proteins.Phypa1_1.FilteredModels3.fasta.gz) [2], Selaginella moellendorffii v1.0 (Selmo1_GeneModels_FilteredModels2_aa.fasta.gz), Volvox carteri f.nagariensis (proteins.FilteredModels2.fasta.gz), Chlamydomonas reinhardtii v4.0 (Chlre4_best_proteins.fasta.gz) [3]. These sequence data were produced by the US Department of Energy Joint Genome Institute http://www.jgi.doe.gov/ in collaboration with the user community (downloaded 02/10/10). Permission to use unpublished JGI genome sequence data in a publication on Pro-rich TRPs was obtained from all associated principal investigators.

**References**

1. Worden AZ, Lee JH, Mock T, Rouzé P, Simmons MP, et al. (2009) Green evolution and dynamic adaptations revealed by genomes of the marine picoeukaryotes *Micromonas*. Science 324: 268-272.
2. Rensing SA, Lang D, Zimmer AD, Terry A, Salamov A, et al. (2008) The *Physcomitrella* genome reveals evolutionary insights into the conquest of land by plants. Science 319: 64-69.
3. Merchant SS, Prochnik SE, Vallon O, Harris EH, Karpowicz SJ, et al. (2007) The *Chlamydomonas* genome reveals the evolution of key animal and plant functions. Science 318: 245-250.
