# Supplementary material for: Global Analysis of Proline-Rich Tandem Repeat Proteins Reveals Broad Phylogenetic Diversity in Plant Secretomes
Source: PLoS One. 2011 Aug 2;6(8):e23167. doi: 10.1371/journal.pone.0023167 (PMC3149072; doi:10.1371/journal.pone.0023167)

Dataset S1. TR architectures of representative proteins from 31 Pro-rich TRP classes.

A AGPA – TR-AGP type alpha.

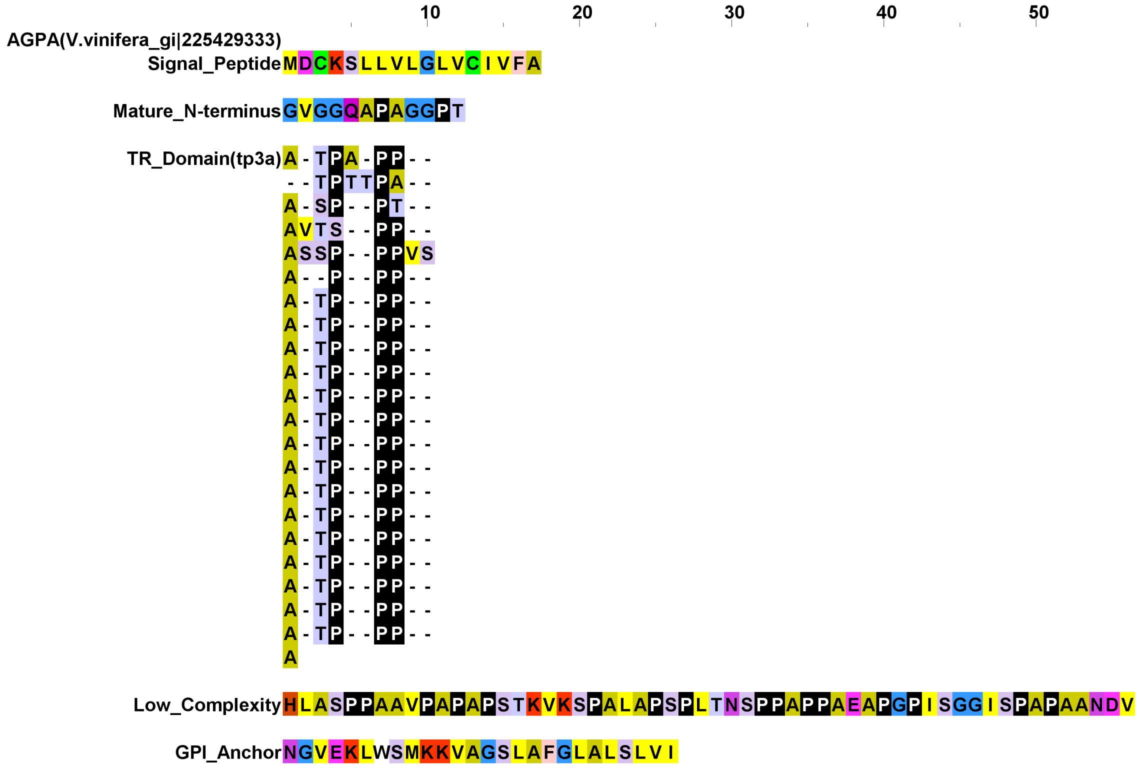

B AGPB – TR-AGP type beta.

```
                                10        20        30        40
AGPB(B.napus_TC77477)
       Signal_Peptide MASSSQAFLLLTLSMVLVHFSLA

   Mature_N-terminus QSPMMAPSGSMSMPPMPSGGS

    TR_Domain(mtp3) PMPMMTPP
                    PMPMMTPP
                    PMPMMTPP
                    PM-AMAPP
                    PMP-MTPP
                    PMP-M--A
                    PMPM-TP

       Low_Complexity SSSPMSPPTTMAPSPETVPDMASPPMMPGMDSSPSPGPMPPAMASPD

           GPI_Anchor SGAFNVRNDVVAISFLVAAHLLLV
```

C AGPC – TR-AGP type gamma.

```
                                           10        20        30        40        50
AGPC(T.aestivum_gi|62861391)
              Signal_Peptide MASPRGLAGLLCFALAAAAASA

Cu_bind_like_domain(PF02298) AQYRVGEQRGWSVPAAGAEPLNTWSARMRFIIGDQLLFVYPKDTDSVLLVDQAAYNACNT
                             TTYVSKFQGGSTVFTLDRSGPFFFISGNEASCKAEQKLIVVVLSVDHTPPGLLPPPP

          TR_Domain1(mtp2) PPSM-PPSSAPPMPSPMS
                           PPSMTPP-SAAPMPSPMS
                           PPSMTPP-SAAPMPSPMS
                           PPSMTPP-SAAPMPSPMS
                           PPSMTPP-SAAPMPSPMS
                           PPSMAPP-SSEPMPSPMT
                           P

                           AAAPGTTPVSPASPAGPAP

               TR_Domain2 SPGTPGGGS
                          SPGTPGSDT
                          S

                          SPPAPAADGANSTTPG

               GPI_Anchor SAAAPVTAGLIGTLAAGIGYAMLAI
```

D EXTA – Extensin type alpha.

```
                              10             20
EXTA(AtEXT6)
    Signal_Peptide  MGIENCIVYVVVLSAIAATVTS

Mature_N-terminus   YPYSSPQTPSYNSPSYEHKGPKYAPHPK

TR_Domain(sp4+,yxy) PYVKSSPPPQYYTPSPKVNYKSPPP
                    PYVYSSPPPPYYSPSPKVDYKSPPP
                    PYVYSSPPPPYYSPSPKVDYKSPPP
                    PYVYSSPPPPYYSPSPKVDYKSPPP
                    PYVYNSPPPPYYSPSPKVDYKSPPP
                    PYVYSSPPPPYYSPSPKVYYKSPPP
                    PYVYSSPPPPYYSPSPKVYYKSPPP
                    PYVYSSPPPPYYSPSPKVYYKSPPP
                    PYVYSSPPPPYYSPSPKVYYKSPPP
                    PYVYSSPPPPYYSPSPKVYYKSPPP
                    PYVYSSPPPPYYSPSPKVYYKSPPP
                    PYVYSSPPPPYYSPSPKVYYKSPPP
                    PYVYSSPPPPYYSPSPKVYYKSPPP
                    PYVYSSPPPPYYSPSPKVYYKSPPP
                    PYVYNSPPPPYYSPSPKVYYKSPPP
                    PYVYSSPPPPYYSPSPKVYYKSPPP
                    PYVYSSPPPPYYSPSPKVYYKSPPP
                    SYVYSSPPPPYYSPSPKVYYKSPPP
                    SYVYSSPPPPYYSPSPKVYYKSPPP
                    PYVYSSPPPPYYSPSPKVTYKSPPP
                    PYVY

C-terminus          KTPYY
```

E EXTB – Extensin type beta.

```
                                      10        20        30        40        50
EXTB(S.tuberosum_gi|296401)
             Signal_Peptide MGKMASLVATLLVVLVSLSLA

         Mature_N-terminus  SESSANYQYSSPPPPVHVYPSPPHHPVYKSPPPHHHHPVYKSPPPSEKPHYPPHTPVYKS
                            PPPHHHHPVYKSPPPPT

       TR_Domain1(sp4+,vyk) PVYKSPPPPKTPHY--PPHTPVYKSPPP-HHH-H
                            PVYKFPPPP-TPVYKSPP-PP--KD--PHYPPHT
                            PVYKSPPPP-TPVYKS---PP------P---P-T
                            PVYKSPPPP-TPVYKSPP-PPV-K---P-YHP-A
                            PVYKSPPPP-TPVYKS---PPV-K---P-YHP-A
                            PVYKSPPPP-TPIYKSPP-PPV-K---P-YHP

                            SPTPYHPK

       TR_Domain2(sp4+,vyk) PVYKSPPPPT-
                            PVYKS-PPPTH
                            YVYSSPPPP

                C-terminus  YHY
```

F EXTC – Extensin type gamma.

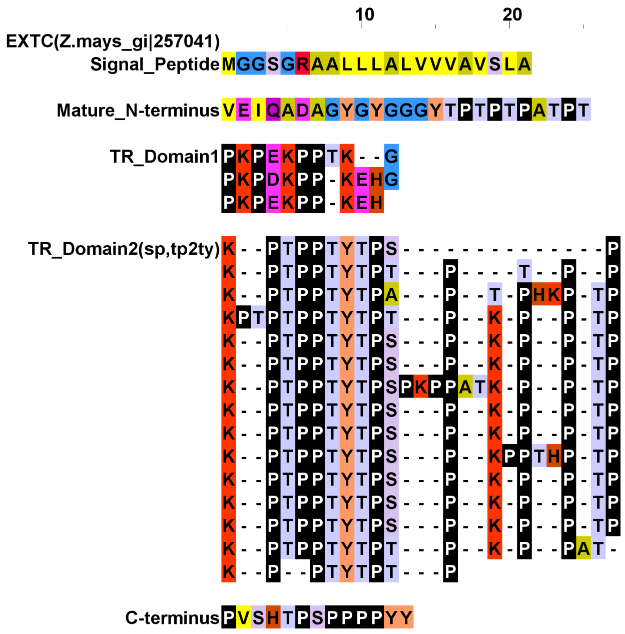

G EXTD – Extensin type delta.

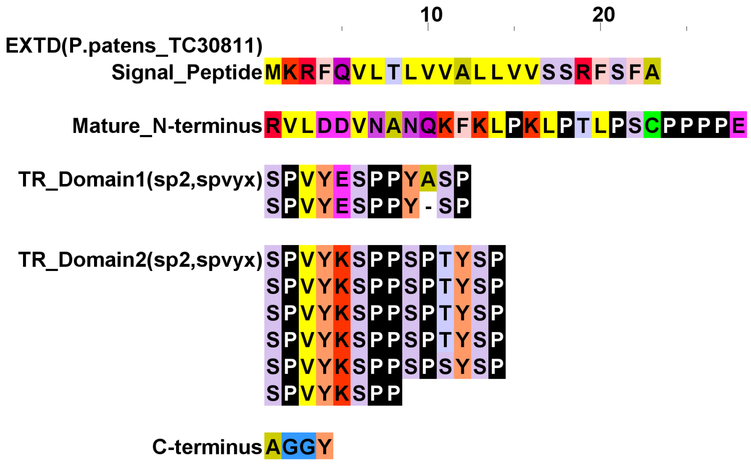

H EXTM – Extensin type miscellaneous (S/T)$P_{2,3}$.

```
                        10        20        30
EXTM(A.thaliana_AtEPR1)
          Signal_Peptide MRVPLIDFLRFLVLILSLSGASVA

      Mature_N-terminus ADATVKQNFNKYETDSGHAHPPPIYGAPPSYTTPPPPI

 TR_Domain(sp2,3-misc) YSPPIYPPPIQK-P-PT
                        YSPPIYPPPIQKPPTPT
                        YSPPIYPPPIQKPPTPT
                        YSPPIYPPPIQKPPTPT
                        YSPPIYPPPIQKPPTPS
                        YSPPVKPPPVQMPPTPT
                        YSPPIKPPPVHKPPTPT
                        YSPPIK-PPVHKPPTPI
                        YSPPIKPPPVHKPPTPI
                        YSPPIKPPPVHKPPTPT
                        YSPPVKPPPVHKPPTPI
                        YSPPIKPPPVHKPPTPI
                        YSPPVKPPPVQTPPTPI
                        YSPPVKPPPVHKPPTPT
                        YSPPVKSPPVQKPPTPT
                        YSPPIKPPPVQKPPTPT
                        YSPPIKPPPV-KPPTPI
                        YSPPVKPPPVHKPPTPI
                        YSPPVKPPPVHKPPTPI
                        YSPPVKPPPIQKPPTPT
                        YSPPIKPPPLQKPPTPT
                        YSPPIKLPPV-KPPTPI
                        YSPPVKPPPVHKPPTPI
                        YSPPVKPPPVHKPPTPT
                        YSPPIKPPPV-KPPTPT
                        YSPPVQPPPVQKPPTPT
                        YSPPVKPPPIQKPPTPT
                        YSPPIKPPPV-KPPTPT
                        YSPPIKPPPVHKPPTPT
                        YSPPIKPPPIHKPPTPT
                        YSPPIKPPPVHKPPTPT
                        YSPPIKPPPVHKPPTPT
                        YSPPIKPPPVHKPPTPT
                        YSPPIKPPPVHKPPTPT
                        YSPPIKPPPVHKPPTPT
                        YSPPIKPPPVQKPPTPT
                        YSPPVKPPPVQLPPTPT
                        YSPPVKPPPVQVPPTPT
                        YSPPVKPPPVQVPPTPT
                        YSPPIKPPPVQVPPTPT

             C-terminus TPSPPQGGYGTPPPYAYLSHPIDIRN
```

I HEXA – Hybrid Extensin type alpha.

```
                          10        20        30        40        50
HEX(AtPEX4)
Signal_Peptide     MPFYKQPWVFSKVFVLAMAKPPSFGCCFFLLFFSFLSSSFVSFA

LRR_Domain         LTDTEAAFIVQRQLLTLPDNGELPDDIEYEVDLKATFANTRLKRAYIALQAWKKAIFSDP
                   FNTTGNWHGPHVCGYTGVVCAPALDDSDVTVVAGVDLNGADIAGHLPAELGLMTDVAMFH
                   LNSNRFCGIIPKSFEKLKLMHEFDVSNNRFVGPFPNVVLSWPDVKYFDLRFNDFEGQVPP
                   ELFKKELDAIFLNDNRFTSVIPESLGESPASVVTFANNKFTGCIPKSIGNMKNLNEIVFM
                   DNDLGGCFPSEIGKLSNVTVFDASKNSFIGRLPTSFVGLTSVEEIDISGNKLTGLVPHNI
                   CQLPNLVNLTYSYNYFSGQGGSCVPGGSRKEIALDDTRNCLASRPEQRSAQECAVVINRP
                   VDCSKDKCAGGSSTPSKPS

TR_Domain1         PVHKPTPVPTT
                   PVHKPTPVPTT
                   PVQKPSPVPTT
                   PVQKPSPVPTT
                   PVHEPSPV

                   LATPVDKPSPVPSRPVQKPQPPKESPQPDDPYDQSPVTKRR

TR_Domain2(sp4vh)  S---PPPAPVN
                   S---PPP-PVY
                   SPPPPPP-PVH
                   S---PPP-PVH
                   SP--PPP-PVY
                   SPPPPPP-PVH
                   S---PPP-PVF
                   S---PPP-PVY
                   S---PPP-PVH
                   S---PPP-PVH
                   SP--PPPAPVH
                   S---PPP-PVH
                   SPP-PPP-PVY
                   S---PPP-PVF
                   S---PPP

                   SQSPPVVYSPPPRPPKINSPPVQSPPPAPVEKKETPPAHAPAPSDDEFIIPPFIGHQ

C-terminus         YASPPPPMFAGY
```

J HLTA – Hybrid PRP/Lipid Transfer Protein type alpha.

```
                                  10        20        30        40        50
HLTA(L.tulipifera_TA1304_3415)
              Signal_Peptide MDSSKISALLLIFMLFVSSA

       Mature_Signal_Peptide SPILACTYCKNPPKHKSPK

            TR_Domain(p2vtl) GKPPKGPINLPPVI
                             VK-P--PITRPPVV
                             GK-P--PITLPPVI
                             GK-P--PVTLPPVI
                             GK-P--PITLPPVI
                             GK-P--PVTLPPVI
                             GK-P--PITLPPVI
                             GK-P--PITLPPVI
                             GK-P--PITLPPVI
                             GK-P--PITLPPVI
                             GK-P--AMTLPPVI
                             GK-P--PITLPPVI
                             GK-P--PWTLPPVI
                             GK-P--PITLPPVI
                             GK-P--PITLPPVI
                             GK-P--PITLPPVI
                             GK-P--PITLPPV

                  LTP_Domain IGTPPTTKPPGCGCSPPPKEETCPIDTLKLGACVDLLGGLVHIGLGDPVVNTCCPLLQGL
                             VEIEAAVCLCTTIKLKLLNLKIFVPIALQLLATCGKTPPPGFTCSL
```

K HLTB – Hybrid PRP/Lipid Transfer Protein type beta.

```
                                       10        20        30        40        50
HLTB(M.sativa_PRP2)
         Signal_Peptide MANYALANVFILLLNLSTLLIVLA

     Mature_N-terminus CPYCPYPSPKPPTHHPPIVKPPVHKRRKYSPTPKPPVHKPPRYPPKPSPCPPPSST

            TR_Domain1 PKPPHV
                       PKPPHH
                       PKPP-V
                       VHPPHV
                       PKP

 TR_Domain2(p2yxpkp2) P-VHPPYVPKPPIVKP-
                       PIVHPPYVPKPP-V-VK
                       P---PPYVPKPP---V-
                       --VRPPYVPKPP-V-V-
                       P-VTPPYVPKPP---V-
                       --VRPPYVPKPP-V-V-
                       P-VTPPYVPKPP-I-V

            TR_Domain3 KPPIVFPPHVPLPPVVPSPPPYV-PSPPIV
                       KPPIVFPPHVPLPPVVPVTPPYVQP-PPIV
                       TPP

            LTP_Domain TPTPPIVTPPVVSPPTPPSETPCPPPPLVPYPPTPPAQQTCSIDALKLGACVDVLGGLIH
                       IGIGGSAKQTCCPLLQGLVDLDAAICLCTTIRLKLLNINLVIPLALQVLIDCGKTPPEGF
                       KCPAY
```

L HLTC – Hybrid PRP/Lipid Transfer Protein type gamma.

```
                          10        20        30        40        50
HLTC(A.thaliana_gi|18396941)
     Signal_Peptide MDSSKLSSLSLCLFLICIIYLPQHSLA

  Mature_N-terminus CGSCNPRKGGKHSPKA

  TR_Domain(p2vtv) PKLPVPPVTV
                   PKLPVPPVTV
                   PKLPVPPVTV
                   PKLPVPPVTI
                   PKLPVPPVTV
                   PKLPVPPVTV
                   PKLPVPPVTV
                   PKLPVPPVTV
                   PKLPVPPVTV
                   PKLPVPPVTV
                   PKLP

         LTP_Domain LPPISGLPIPPVVGPNLPLPPLPIVGPILPPGTTPPATGGKDCPPPPGSVKPPSGGGKAT
                    CPIDTLKLGACVDLLGGLVKIGLGDPAVNKCCPLLKGLVEVEAAACLCTTLKLKALDLNL
                    YVPVALQLLLTCGKNPPPGYTCSI
```

M HLTD – Hybrid PRP/Lipid Transfer Protein type delta.

```
                          10        20        30        40        50
HLTD(A.thaliana_gi|42565109)
     Signal_Peptide MGSRSQNLSFLVLLLLGFVAVSYA

  Mature_N-terminus CDCTPPKPSPAPH

 TR_Domain1(p2tvk) K--P-P-K-HP--V
                   K--P-P-K-PP-AV
                   K--P-P-K-PP-AV
                   KP-PTP-K-PP-TV
                   K--PHP-K-PP-TV
                   K--PHP-K-PP-TV
                   K--PHP-K-PP-TV
                   KP-PHP-K-PP-T-
                   KPHPHP-K-PP-IV
                   K

        TR_Domain2 PPTKPPPSTPK-
                   PPTKPPPSTPKP
                   PTTKPPPSTPKP
                   PHHKPPP-TPCP
                   PPT

        TR_Domain3 PTPTPPVVTP
                   PTPTPPVITP
                   PTPTPPVVTP
                   PTPTPPVITP
                   PTPTPPVITP
                   PTPTPPVVTP
                   PTPTPPVVTP
                   PTPTPP--TP

         LTP_Domain IPETCPIDTLKLGACVDVLGGLIHIGLGKSHAKAECCPVLGGLLDLDAAVCLCTTIKLKL
                    LNIDLVLPIALELLLDCGKTPPSDFKCPA
```

N HLTE – Hybrid PRP/Lipid Transfer Protein type epsilon.

```
                        10        20        30        40        50
HLTE(Z.mays_gi|226499476)
           Signal_Peptide MATSTSVPVLLLLAAAAGLLLLPGSLA

        Mature_N-terminus WQSSNCAPGAPGSGSPTWPSPGSGSGGGHSKPPKHHHGKPPKCPPCN

   TR_Domain(sp2yvp2y) PP-Y--VPPTPRP-S-PPYV
                       PP-Y--VPPTPRP-S-PPYV
                       PP-YVPVPPTPRP-S-PPYV
                       PP-YVPVPPTPRP-S-PPYV
                       PP-YVPVPPTPRP-S-PPYV
                       PPTP--RPPTP-PYV-PP-T
                       PP-Y--VPPTPRP-SPPPYV
                       PP-Y--VPPTPRP-S-PPYV
                       PP-Y--VPPTP

            LTP_Domain PAVRTCPIDTLKLNACVDVLSGLIHLVIGQEARSKCCPLVQGVADLDAALCLCTTIRARL
                       LNINIYLPIALNLLITCGKHAPSGFQCPPLYD
```

O HLTF – Hybrid PRP/Lipid Transfer Protein type phi.

```
                        10        20        30        40        50
HLTF(P.trichocarpa_TC89836)
           Signal_Peptide MAKFAVANLLILLLNLGALLTSLA

        Mature_N-terminus CPTCPY

        TR_Domain(p2vtp) TPHP---KP-PKRPPI-KPPKPPV--
                         TP-P-I-KP-PK-PPI-KPPKPPV--
                         TP-P-I-KP-PK-PPI-KPPKPPV--
                         TP-PVI--PIP--PTL-PPPKPPV--
                         TP-PVI----PT-PPILPPPEPPVIP
                         TP-P-IVKP-P--P

              LTP_Domain TPPKQETCPIDTLKLGACVDVLGGLVHIGIGSSAKDECCPLLEGLVDLDAAVCLCTVIKA
                         KLLNINLILPIALELLVDCGKNPPEGFKCPS
```

P HPOA – Hybrid PRP/Pollen Ole e I type alpha.

```
                                  10        20        30        40        50
HPOA(A.thaliana_PRP2)
              Signal_Peptide MRILPKSGGGALCLLFVFALCSVAHSLS

Pollen_Ole_e_I_domain(DUF1210) RDVKVVGDVEVIGYSEISKIKIPNAFSGLRVTIECKAADSKGHFVTRGSGEVEETGKFHL
                               NIPHDIVGDDGTLKEACYAHLQSAFGNPCPAHDGLEASKIVFLSKSGANHVLGLKQSLKF
                               SPEVCISKFWHMPKFPLPPPLNLPPLTFPKIKKPCP

          TR_Domain(p2vpvyk) PIY-----I--PPVV-IPKKPCPPKVAHKPIYKPPV
                             PIYKPPVPIYKPPVV-IPKKPCPPK-IHKPIYKPPV
                             PIY-------KPPVV-IPKKTFPP--LHKPIYKHPV
                             PIYK---PIFKPPVVVIPKKPCPP

                 C-terminus LPKFPHFPPKYIPHPKFGKWPPFPSHP
```

Q HPOB – Hybrid PRP/Pollen Ole e I type beta.

```
                                    10        20        30        40        50        60        70
HPOB(Z.mays_gi|162461641)
                  Signal_Peptide MAADALRGLLPGVYAVLMVIAVTSA

Pollen_Ole_e_I_domain(DUF1210) ASSDASSVVVGLAKCADCTRKNLKAEAAFKGLEVAIKCKNSKGEYESKAMGKLDGFGAFS
                               IPLSTDLHGADCVAQLHSAAGNPCPGQEPSRIVPQSSKGNFVVVPGKTDYPSKECASATL
                               CGSIKKHLLDHFHKKPVPPKPKPE

     TR_Domain(pepk-embed1) PKPE--LEPKPEPKPEPENHPPTPTYGSSTPIYHPP--SRQLFDKKHMF-DNFHKHHDYHHFFDHFHKKPVPPK
                            PKPEPKPEPKPKPQ--PEYHSPTPTYGSPTPIYHPPA--RHLFDKKP-LLDHFHKDHDYHHFLDHFHKKPVPQK
                            PK----LE--PKPQPKPEYHPPTPTYVSPTPIYHPPAETEMKYDKKH-F-DHFHKDHDYHHFFDHFHKKPVPPK
                            PKPE--PEPKPKPQPKPEYKPPMPTYGSPTPIYHPPAKKEVAFDKKH-FLDHFHKDHDYHHFFDHFHKKPVPPK
                            PKPE--PKPEPKPQPEPEYHPPTP

                 C-terminus TYGSPTPIYHPPAKH
```

R HPOC – Hybrid PRP/Pollen Ole e I type gamma (AGP).

```
                                       10        20        30        40        50
HPOC(A.thaliana_AGP31)
                  Signal_Peptide MGFIGKSVLVSLVALWCFTSSVFT

             Mature_N-terminus EEVNHKTQTPSLAPAPAPYHHG

                   TR_Domain1 HHHPHP-P
                              HHH-HPHP
                              HPHPHP-P

                              AKS

            TR_Domain2(p2vk) PVKPPVKAPVSPPAKPPVKP
                             PVYPPTKAPVKPPTKPPVKP
                             PVSPPAKPPVKPPVYPPTKA
                             PVKPPTKPPVKPPVYPPTKA
                             PVKPPTKPPVKPPVYPPTKA
                             PVK

            TR_Domain3(p2vk) PPTKPPVKPPVS
                             PPAKPPVKPPVY
                             PPTKAPVKPPVS
                             PPTKPPVTPPVY
                             PP

Pollen_Ole_e_I_domain(DUF1210) KFNRSLVAVRGTVYCKSCKYAAFNTLLGAKPIEGATVKLVCKSKKNITAETTTDKNGYFL
                               LLAPKTVTNFGFRGCRVYLVKSKDYKCSKVSKLFGGDVGAELKPEKKLGKSTVVVNKLVY
                               GLFNVGPFAFNPSCPK
```

S KPIP – KPIP domain protein.

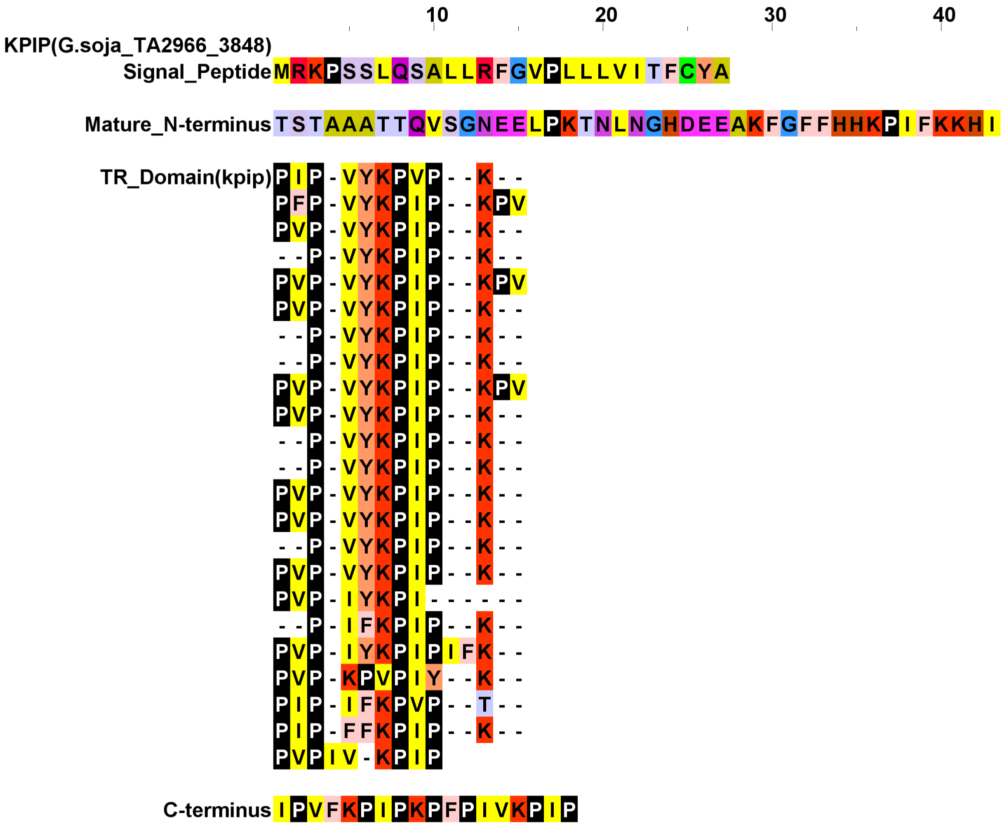

T MPAV – MPAV domain protein.

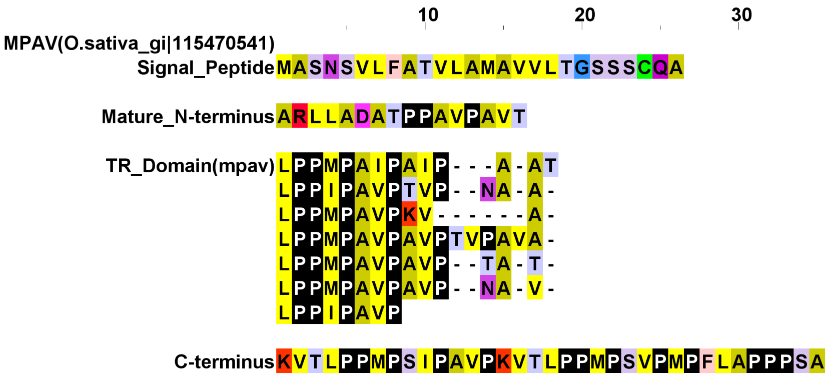

U PEHK – PHEK domain protein.

```
                                   10        20        30        40        50
PEHK(V.vinifera_gi|225433073)
                Signal_Peptide MSPTYLLVVLLGLVVLTAPSLA

              Mature_N-terminus DYPTHPPF

              TR_Domain(p3ehk1) EKPPPEHKPPV
                                EKPPPEHKPPV
                                EKPPPEHKPPV
                                EKPPPEHKPPV
                                EKPPPEHKPPV
                                EKPPPEHKPPV
                                EKPPPEHKPPV
                                EKPPPEHKPPV
                                EKPPPEHKPPV
                                EKPPPEHKPPV
                                EKPPPEHKPPV
                                EKPPPEHKPPV
                                EKPPPEHKPPV
                                EKPPPLHKPPV
                                EKPPPEHKPPV
                                EKPPPEHKPPV
                                EKPPPEHKPPV
                                EKPPPEHKPPV
                                EKPPPEHKPPV
                                EKPPPEHK---
                                EKPLPEHKPP

                    C-terminus TPVGKPPKGEKPPHCGHNPGHPPAENAEDSYKPPRKIKPPSTPAEKQPGPGKKLPTPPHK
                               PPHKPPTPTHPN
```

V PELPK – PELPK domain protein.

```
                                   10        20
PELPK(O.sativa_gi|115473185)
                Signal_Peptide MVFKKNAMSSSVLFLAALLLLSSSSMSSA

              Mature_N-terminus ARWLEEEY

              TR_Domain(pelpk2) PPHPT-VPELPKPEV
                                PPHPA-VPELPKHEE
                                PPHPV-VPELPKHEE
                                PPHPV-VPELPKPEL
                                PPHPV-VPELPKHEE
                                PPHPAVVPELPKHEE
                                PPHPAVVPEFPKHEE
                                PPHPA-VPELP-H--
                                ---PA-V---P---E
                                IPHPA-VPELPKHEE
                                PPHPV-VPELPK-PE
                                VPHAA-VPELPKPEL
                                PPHPA-VPELPKHEE
                                PPHPV-VPELPKHEE
                                PPHPV-VPELPKPEE
                                PHHPE-VPE

                    C-terminus HEQPPKPESHYPEVPMAKP
```

W PEPKA – PEPK type alpha.

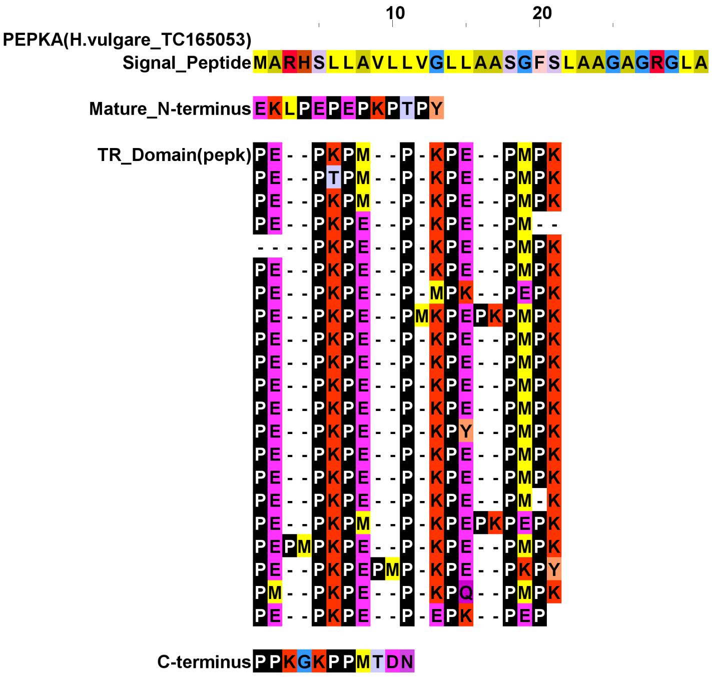

X PEPKB – PEPK type beta.

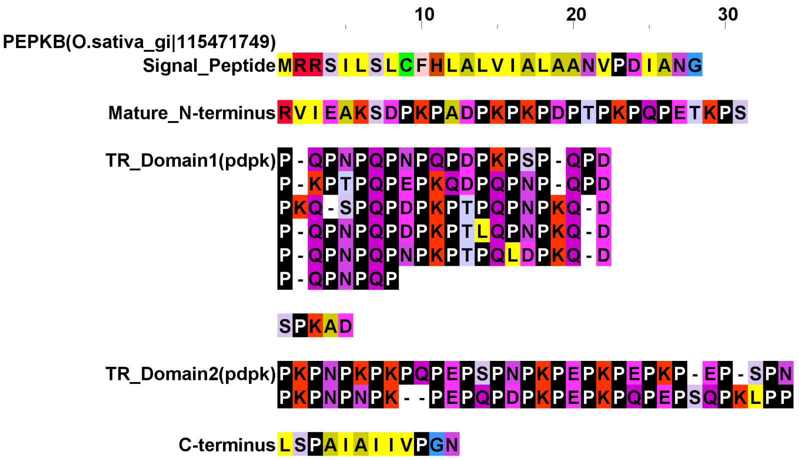

Y PEPKC – PEPK type gamma

```
                                       10        20        30        40        50
PEPKC(G.hirsutum_gi|119888004)
                Signal_Peptide MSTTHLLVFLLGVVTLTTPTFG

           Mature_N-terminus TYESPNYGKPPTPEFK

   TR_Domain(pepk-embed2) PPKVK----P-PPYEPKPPVYE
                          PPK-K----E-KP-EPKPPVYA
                          PPK-K----E-KP-GPKPPVYE
                          PPK-K----E-KP-EPKPPVYT
                          PPK-K----E-EP-KPKPPVYE
                          PPK-K----E-KP-EPKPPIYT
                          PPK-K----E-KP-EPKPPVYE
                          PPK-K----E-KP-EPKPPVYT
                          PPK-K----E-KP-EPKPPVYE
                          PPK-KPPMYEPKP--PKPPVYT
                          PPK-K----E-KP-EP

                C-terminus IPPMYEPPKKPPMCEPKPPKPPVYTPPKKEKSDPSQPCINLPQPTIYEPNHLSHH
```

Z PHEK – PHEK domain protein.

```
                                   10        20
PHEK(G.max_gi|128421)
            Signal_Peptide MTSVLHYSLLLLLLGVVILTTPVLA

         Mature_N-terminus NLKPRFF

              TR_Domain1 YEPPPIEKPPT
                         YEPPPFYKPP-
                         YYPPP

                         VHH

      TR_Domain2(p2hek1) PPPEY-QPPHEK-
                         TPPEYLPPPHEK-
                         PPPEY-LPPHEK-
                         PPPEY-QPPHEK-
                         -------PPHEN-
                         PPPEH-QPPHEK-
                         -PPEH-QPPHEK-
                         PPPEY-EPPHEK-
                         PPPEY-QPPHEK-
                         PPPEY-QPPHEK-
                         PPPEY-QPPHEK-
                         PPPEH-QPPHEK-
                         -PPEH-QPPHEK-
                         PPPEY-QPPHEK-
                         PPPEY-QPPQEK-
                         -------PPHEK-
                         PPPEY-QPPHEK-
                         PPPEH-QPPHEK-
                         PPPVY-PPPYEK-
                         PPPVY-EPPYEKP
                         PPVVY-PPPHEK-
                         -PPIYEPPPLEK-
                         PPV-Y-NPP

              C-terminus PYGRYPPSKKN
```

AA PRPA – Proline-Rich Protein type alpha.

```
                                  10        20
PRP(M.truncatula_gi|410107)
              Signal_Peptide MASSNFLVLLLFALFAIPRGLA

           Mature_N-terminus NYD

           TR_Domain(p2vyk) KPPVYQPPVY
                            KPPVEKPPVY
                            KPPVEKPPVY
                            KPPVEKPPVY
                            KPPVEKPPVY
                            KPPVEKPPVY
                            KPPVEKPPVY
                            KPPVEKPPVY
                            KPPVEKPPVY
                            KPPVEKPPVY
                            KPPVEKPPVY
                            KPPVEKPPVY
                            KPPVEKPPVY
                            KPPVEKPPVY
                            KPPVEKPPVY
                            KPPVEKPPVY
                            KPPVEKPPVY
                            KPPVEKPPVY
                            KPPVEKPPVY
                            KPPVEKPPVY
                            KPPVEKPPVY
                            KPPVEKPPVY
                            KPPVEKPPVY
                            KPPVEKPPIY
                            KPPVEKPPVY
                            KPPVEKPPVY
                            KPPVEKPPVY
                            KPPVEKPPVY
                            KPPVEKPPVY
                            KPPVYKPPVY
                            KPPVEKPPVY
                            KPPVYKPPVE
                            KPPVYKPPVY
                            KPPVEKPPVY

                 C-terminus GPPHHP
```

AB PRPB – Proline-Rich Protein type beta.

```
                                  10        20        30
PRPB(P.taeda_TC93219)
       Signal_Peptide MEFGIPGGMKFCKSLVIFLLSISCLFA

   Mature_N-terminus  SQTSASYGHPAPKPYAYISP

          TR_Domain1  PPPVIPPPVIK
                      PPPVITPPVTK
                      PPPVYPPP

                      HVVPKPPPHEISPVHPPPKSPSPPVYTPPVIHPPIHKPPIH

TR_Domain2(p3vyk)     KPP-IKPPPVY
                      TPP-IKPPPVY
                      TPPVI-HPPEH
                      KPP-IKPPPVY
                      IPP-IKPPPVY
                      IPP-IKPPPVY
                      VPP-IKPPPVY
                      VPP-IKPPPVY
                      VPP-IKPPPVY
                      KPP-IK

                      TPPGHKPPPHGISPVHPPPLVKP

TR_Domain3(p3vyk)     KPPPVYKPPVPVI------PPPVT
                      KPPPVYKPPVPVI------PPPVT
                      K-PPVYKPPVPVI------PPPVV
                      KPPPVYKPPVPVI------PPPVV
                      KPPPVYKPPVPVIPPPVVKPPPVV
                      KPPPVYKPPVPVI------PPPVV
                      KPPPVYKPPVPVI------PPPVV
                      KPPPVYKPPVPVI------PPPVV
                      KPPPVY

                      S
```

AC QRA – Gln-rich type alpha.

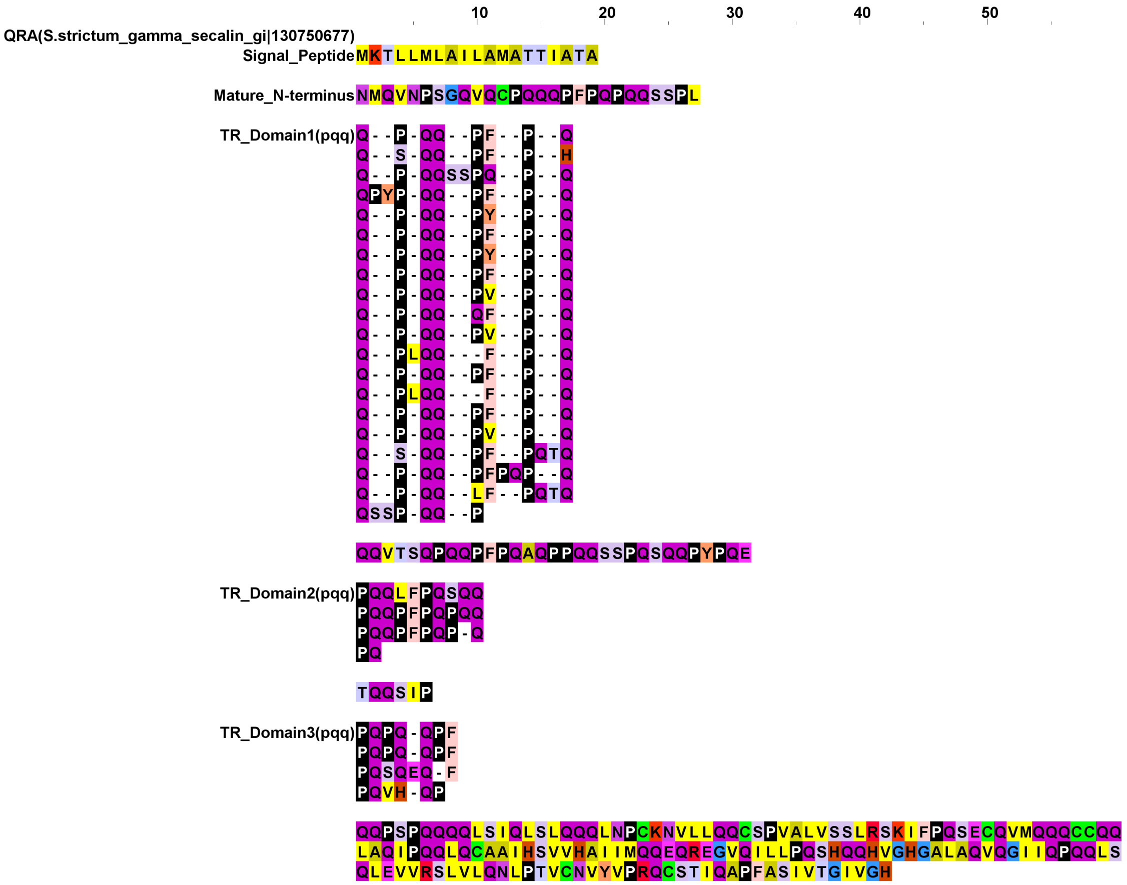

AD QRB – Gln-rich type beta.

```
                                                10          20          30          40          50
QRB(S.cereale_HMW_glutenin_gi|14329757)
                         Signal_Peptide MAKRLVLFAAVVVALVALTVA

                      Mature_N-terminus EGEASGQLHCERELQERELEACRQIVDQQLRDTSPGCRPVAVSPGTGQHEQQTVVPLKGG
                                        SFYPDETSPPQQLEQRILWGIPTLLKRYYPSVTSPHQGSYYLGQTSLRQPGQAQQPGQGQ
                                        QPGQA

                       TR_Domain1(gqq) QQPGQGQQ--PG-QRQQPEKGQQGYYPTTP
                                        QQPGQVQQ--PG-QGQQPEQGQPGYYLTSL
                                        ------QQ--PG-QGQQPGQGQPGYYPTSP
                                        QQSGQGQQLGQGQQGQQPGQGQPGYYPTSP
                                        QQPGQGQQ--PG-QGQQPGQGQQGQQPAQG
                                        -------Q-----QGQQP--GQ-G------
                                        QQ-G--QQ--PG-QGQQPGEGQQGYYPTFP
                                        QQPGQVQQ--PG-QGQQPGQGQPGYYPTSP
                                        QQPGQGQQ--PG-QG

                       TR_Domain2(gqq) QQPG---Q-R--QQPGQGKP-RYYPTSP
                                        QQSG---Q-G--QQPGQGQS-GYYPTSP
                                        QQPG---Q-E--QQPGQGQQ-------V
                                        QQPG---Q-G--QQPGQGQQ-GYYPTSP
                                        QQPGQAQQPGQWQQPGQGQS-GYYPTSQ
                                        QQPG---Q-G--QQPGQGQQPG-QGQQG
                                        QQPG---Q-G--QQPGQGQQ-GYYLTSP
                                        QQPG---Q-G--QQPGQGQP-GYYPTSP
                                        QQPG---Q-G--QQPGQGQQPG-QGQQG
                                        QQPG---Q-G--QQPGQGQQ-GYYPTSL
                                        QQPG---Q-G--QQPGQGQL-EYYPTS-
                                        --P---------QQPGQGQP-GYYPTSP
                                        QLPG---Q-L--QQPAQGQQ-GYYPTSP
                                        ------------QQPGQGQQ-EYYPTSP
                                        QQPG---Q-W--QQPGQGQQ-GYYMTSP
                                        QQSG---Q-G--QQPGQGQH

                       TR_Domain3(gqq) PGQGLQPGQGQEGYYPTSGQQ
                                        PGQWLQIGQGQQGYYPTSSQQ
                                        --SG-Q-GQ-Q-GYYLTSPQQ
                                        PGQGKQPGQGQ

                             C-terminus QSYDSPYHVSAEHQAASLKVAKAQQLAAQLPAMCRLEGGDALLASQ
```

AE SPAP – SPAP domain protein.

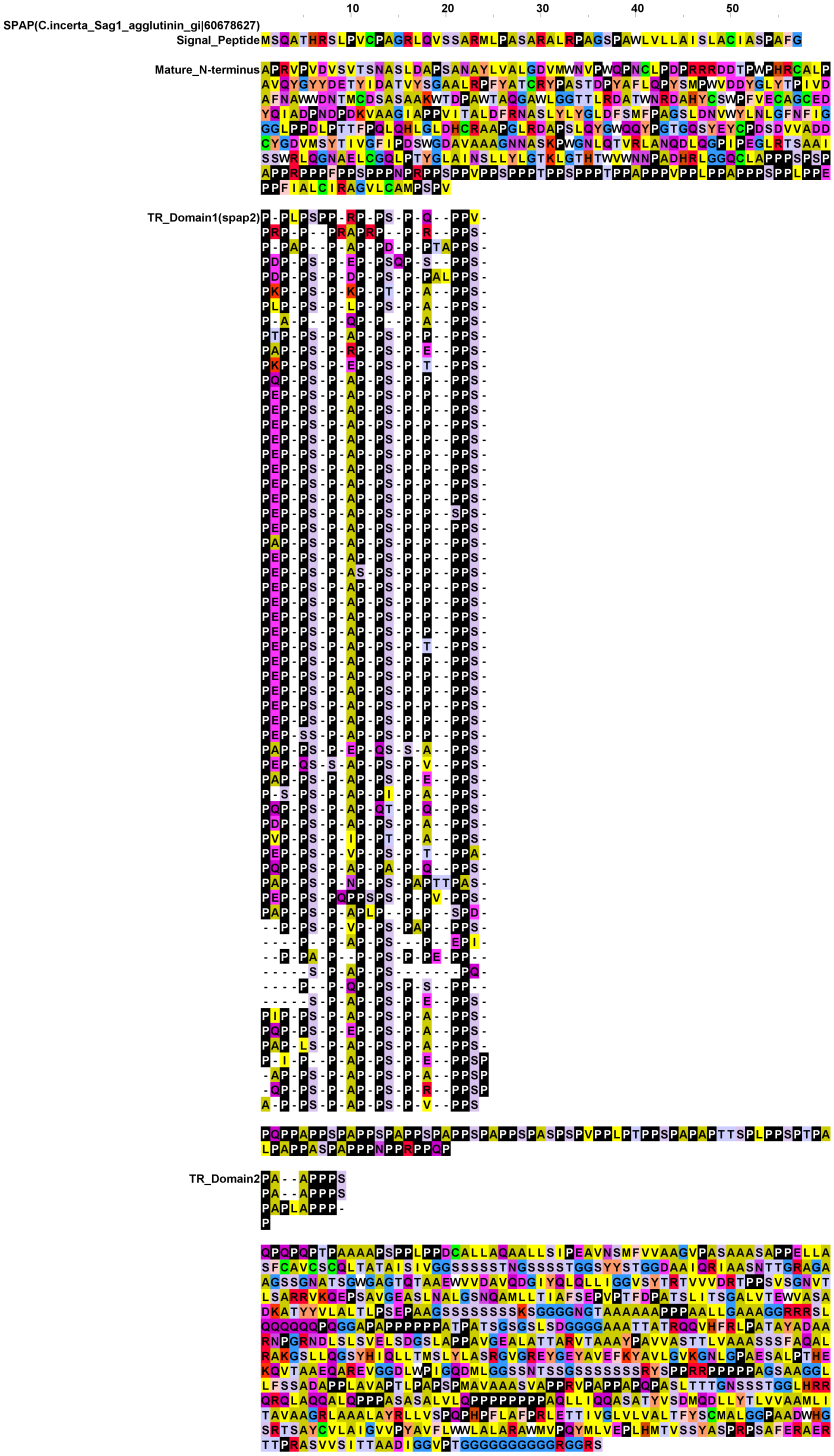

Supplement: Dataset S1 — TR architectures of representative proteins from 31 Pro-rich TRP classes. TR architectures of representative protein examples for each of 31 TRP classes (Tables S6, S7, S8) are illustrated. All TRs were identified and aligned using XSTREAM [47] using the same parameters described in Materials and Methods . Each protein is shown N-terminus to C-terminus, from left to right and top to bottom. Major sequence features, in addition to TR domains, are indicated. For TR domains classified by the TR taxonomy (see Tables S3, S4, S5), the corresponding TR class is given in parentheses, e.g. TR_Domain1(mtp2). The scale-bar on top shows the number of amino acids from left to right in a given row. All 31 representative proteins are listed in alphabetical order and all images were rendered with JalView [57]. (PDF) [file pone.0023167.s028.pdf]
