# Supplementary material for: Global Analysis of Proline-Rich Tandem Repeat Proteins Reveals Broad Phylogenetic Diversity in Plant Secretomes
Source: PLoS One. 2011 Aug 2;6(8):e23167. doi: 10.1371/journal.pone.0023167 (PMC3149072; doi:10.1371/journal.pone.0023167)
Supplement: Dataset S2 — Representative multiple sequence alignments. Multiple sequence alignments of the 50 N-terminal and 50 C-terminal amino acids of full-length ORFs from 26 of 31 TRP classes (see Tables S6, S7, S8) are shown. Sequences were preprocessed to remove most of the TR domain to increase alignment quality. (PHEK and PRPB classes are not shown due to low quantities of complete ORFs; both EXTM and SPAP are heterogeneous protein classes, and are therefore not shown; PEHKs are shown in Figure S3). Each aligned TRP sequence (obtained from Text S3) has a predicted secretion signal, and is either derived from a genome sequence project, the NR database, or an EST, in which case a predicted stop codon was required. All sequence alignments were created with MUSCLE [58] and rendered using JalView [57]. (PDF) [file pone.0023167.s029.pdf]

Dataset S2. Representative multiple sequence alignments.

A EXTAs

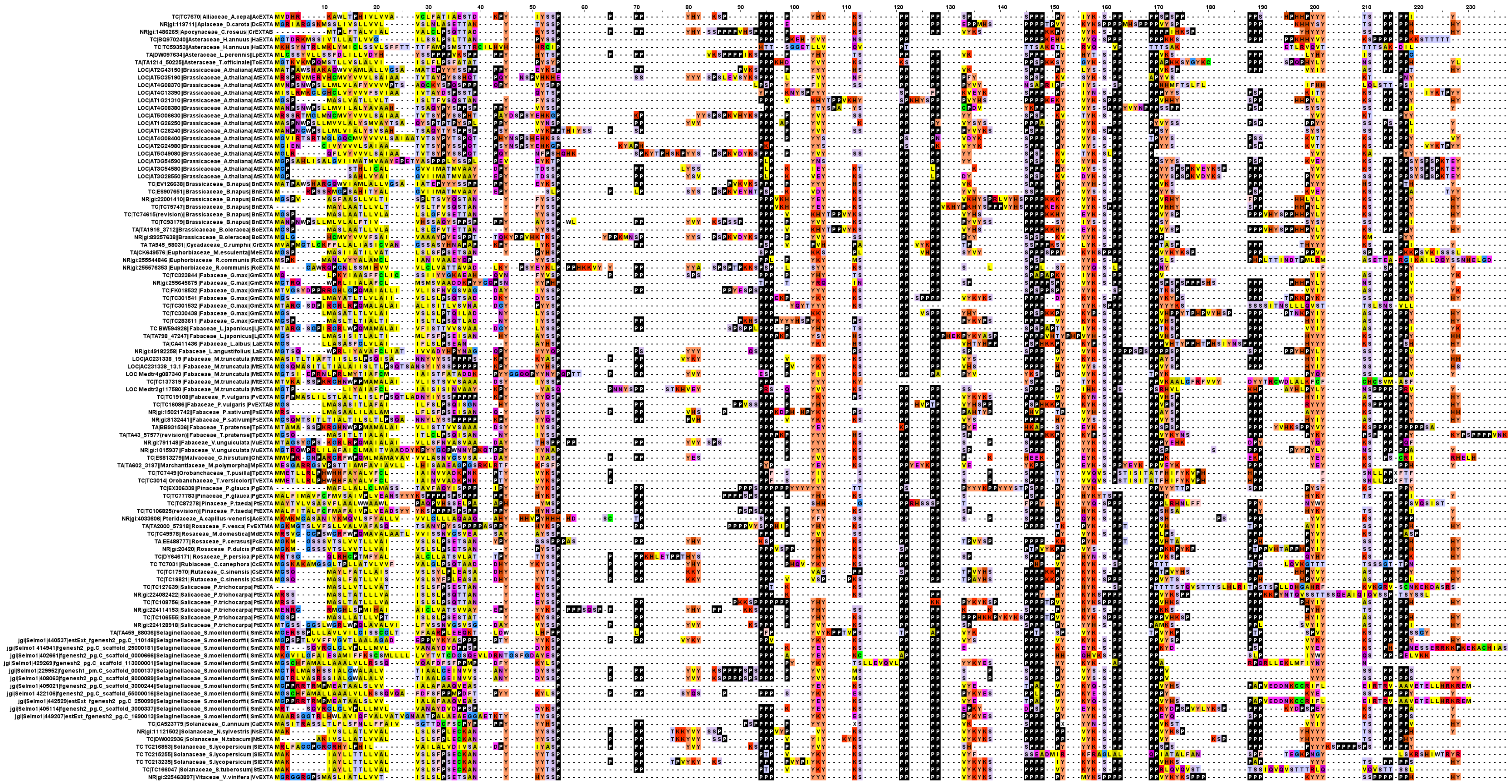

B EXTBs

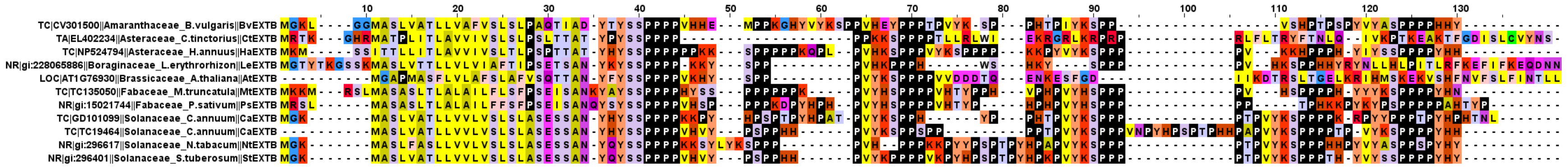

C EXTCs

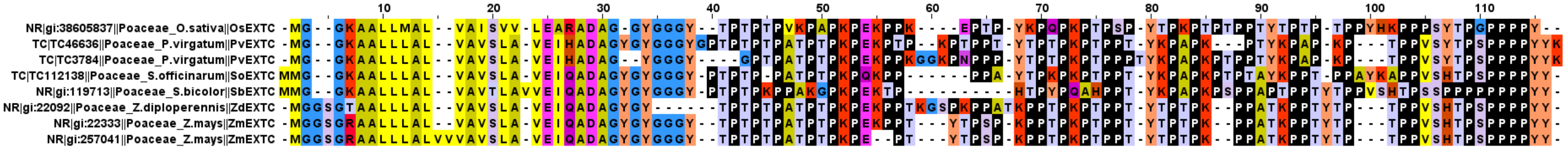

D EXTDs

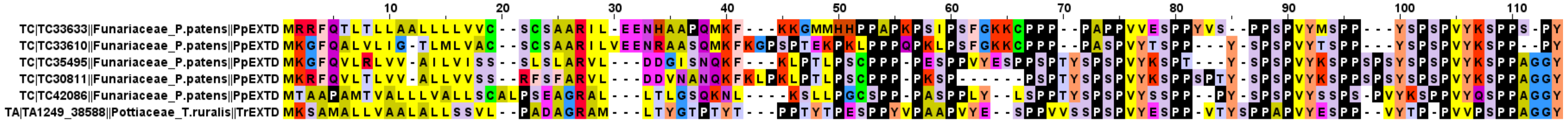

E HEXAs

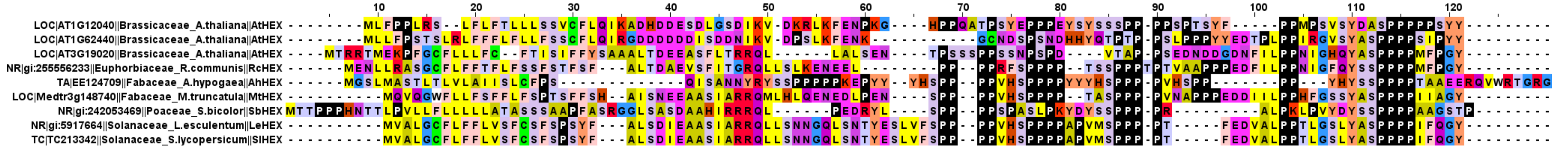

F AGPAs

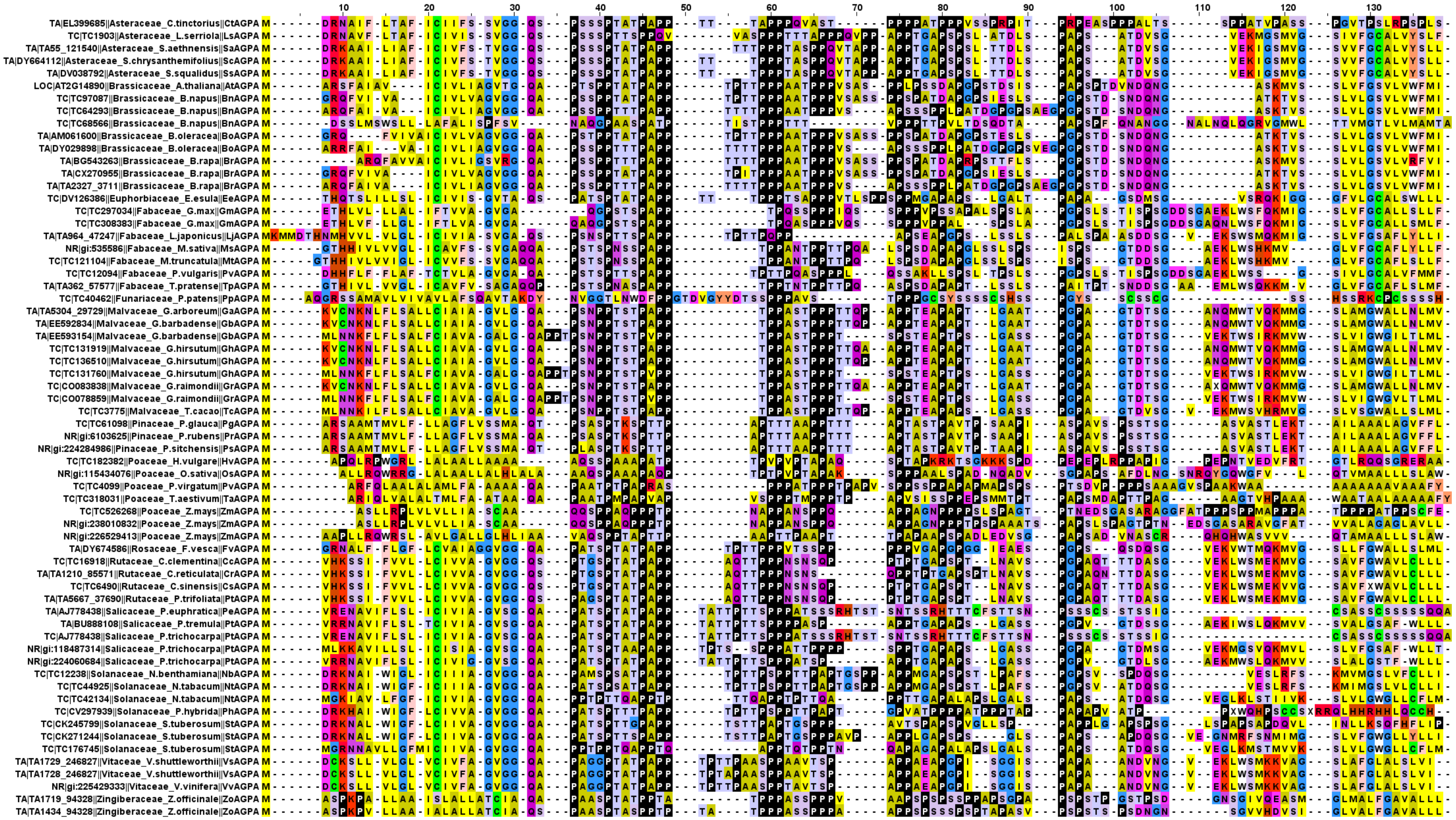
10 20 30 40 50 60 70 80 90 100 110 120 130
TA|EL399685||Asteraceae_C.tinctorius||CtAGPA
TC|TC1903||Asteraceae_L.serriola||LsAGPA
TA|TA55_121540||Asteraceae_S.aethnensis||SaAGPA
TA|DY664112||Asteraceae_S.chrysanthemifolius||ScAGPA
TA|DV038792||Asteraceae_S.squalidus||SsAGPA
LOC|AT2G14890||Brassicaceae_A.thaliana||AtAGPA
TC|TC97087||Brassicaceae_B.napus||BnAGPA
TC|TC64293||Brassicaceae_B.napus||BnAGPA
TC|TC68566||Brassicaceae_B.napus||BnAGPA
TA|AM061600||Brassicaceae_B.oleracea||BoAGPA
TA|DY029898||Brassicaceae_B.oleracea||BoAGPA
TA|BG543263||Brassicaceae_B.rapa||BrAGPA
TA|CX270955||Brassicaceae_B.rapa||BrAGPA
TA|TA2327_3711||Brassicaceae_B.rapa||BrAGPA
TC|DV126386||Euphorbiaceae_E.esula||EeAGPA
TC|TC297034||Fabaceae_G.max||GmAGPA
TC|TC308383||Fabaceae_G.max||GmAGPA
TA|TA964_47247||Fabaceae_L.japonicus||LjAGPA
NR|gi:535586||Fabaceae_M.sativa||MsAGPA
TC|TC121104||Fabaceae_M.truncatula||MtAGPA
TC|TC12094||Fabaceae_P.vulgaris||PvAGPA
TA|TA362_57577||Fabaceae_T.pratense||TpAGPA
TC|TC40462||Funariaceae_P.patens||PpAGPA
TA|TA5304_29729||Malvaceae_G.arboreum||GaAGPA
TA|EE592834||Malvaceae_G.barbadense||GbAGPA
TA|EE593154||Malvaceae_G.barbadense||GbAGPA
TC|TC131919||Malvaceae_G.hirsutum||GhAGPA
TC|TC136510||Malvaceae_G.hirsutum||GhAGPA
TC|TC131760||Malvaceae_G.hirsutum||GhAGPA
TC|CO083838||Malvaceae_G.raimondii||GrAGPA
TC|CO078859||Malvaceae_G.raimondii||GrAGPA
TC|TC3775||Malvaceae_T.cacao||TcAGPA
TC|TC61098||Pinaceae_P.glauca||PgAGPA
NR|gi:6103625||Pinaceae_P.rubens||PrAGPA
NR|gi:224284986||Pinaceae_P.sitchensis||PsAGPA
TC|TC182382||Poaceae_H.vulgare||HvAGPA
NR|gi:115434076||Poaceae_O.sativa||OsAGPA
TC|TC4099||Poaceae_P.virgatum||PvAGPA
TC|TC318031||Poaceae_T.aestivum||TaAGPA
TC|TC526268||Poaceae_Z.mays||ZmAGPA
NR|gi:238010832||Poaceae_Z.mays||ZmAGPA
NR|gi:226529413||Poaceae_Z.mays||ZmAGPA
TA|DY674586||Rosaceae_F.vesca||FvAGPA
TC|TC16918||Rutaceae_C.clementina||CcAGPA
TA|TA1210_85571||Rutaceae_C.reticulata||CrAGPA
TC|TC6490||Rutaceae_C.sinensis||CsAGPA
TA|TA5667_37690||Rutaceae_P.trifoliata||PtAGPA
TA|AJ778438||Salicaceae_P.euphratica||PeAGPA
TA|BU888108||Salicaceae_P.tremula||PtAGPA
TC|AJ778438||Salicaceae_P.trichocarpa||PtAGPA
NR|gi:118487314||Salicaceae_P.trichocarpa||PtAGPA
NR|gi:224060684||Salicaceae_P.trichocarpa||PtAGPA
TC|TC12238||Solanaceae_N.benthamiana||NbAGPA
TC|TC44925||Solanaceae_N.tabacum||NtAGPA
TC|TC42134||Solanaceae_N.tabacum||NtAGPA
TC|CV297939||Solanaceae_P.hybrida||PhAGPA
TC|CK245799||Solanaceae_S.tuberosum||StAGPA
TC|CK271244||Solanaceae_S.tuberosum||StAGPA
TC|TC176745||Solanaceae_S.tuberosum||StAGPA
TA|TA1729_246827||Vitaceae_V.shuttleworthii||VsAGPA
TA|TA1728_246827||Vitaceae_V.shuttleworthii||VsAGPA
NR|gi:225429333||Vitaceae_V.vinifera||VvAGPA
TA|TA1719_94328||Zingiberaceae_Z.officinale||ZoAGPA
TA|TA1434_94328||Zingiberaceae_Z.officinale||ZoAGPA

G AGPBs

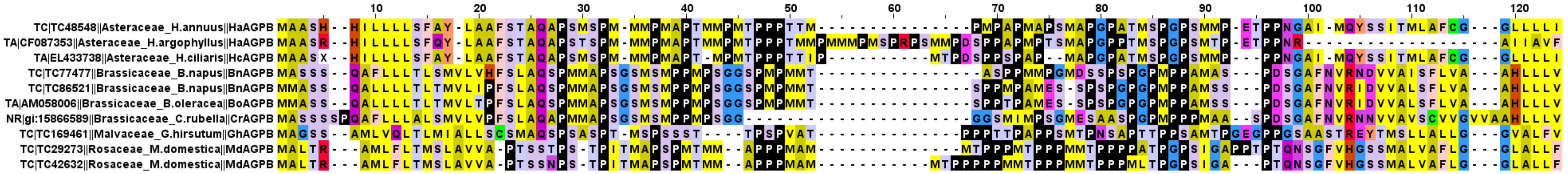

H AGPCs

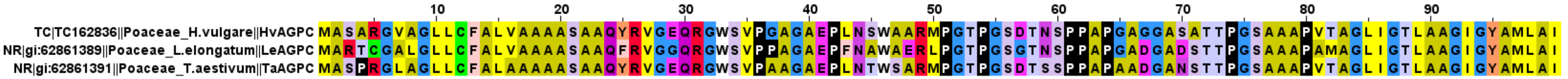

I HLTAs

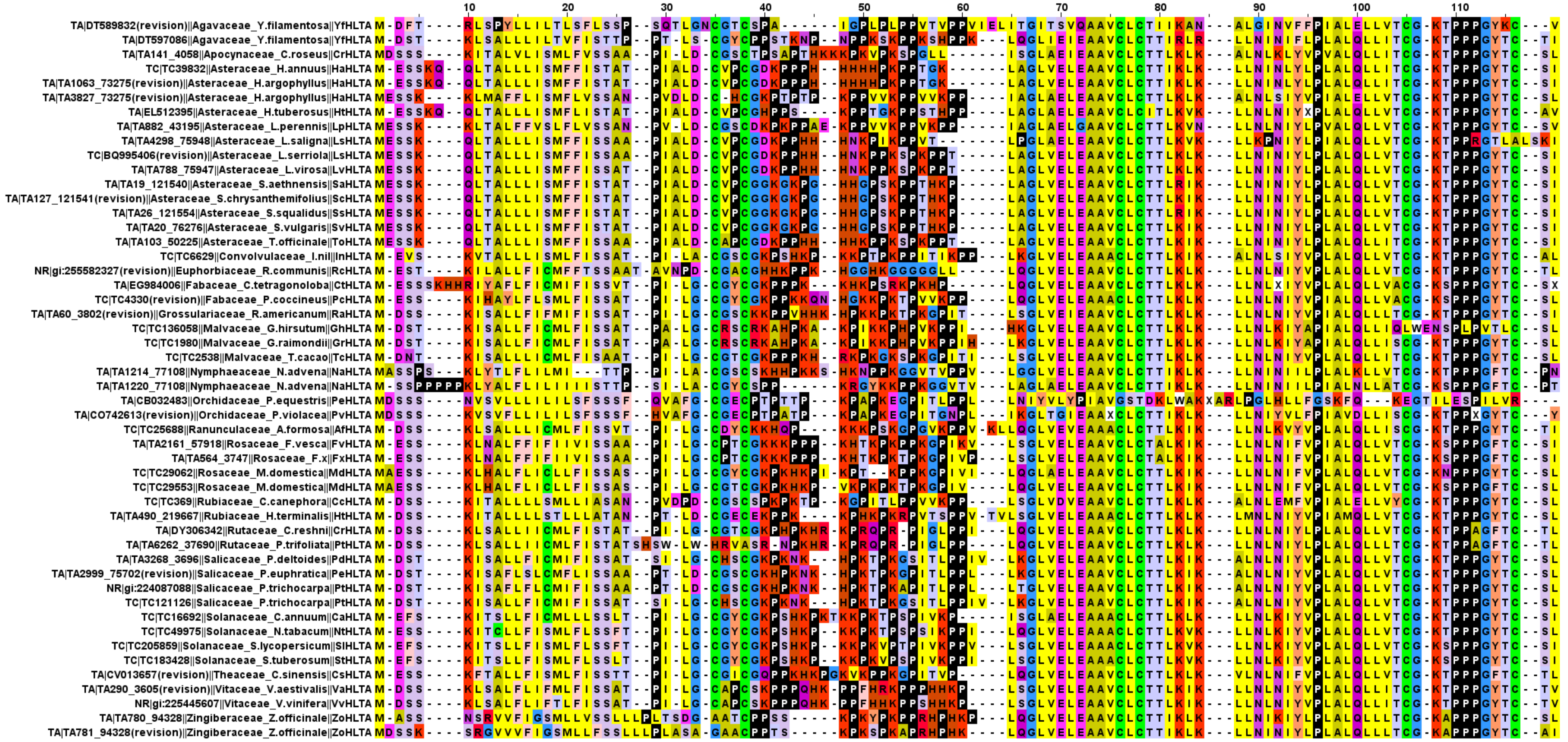

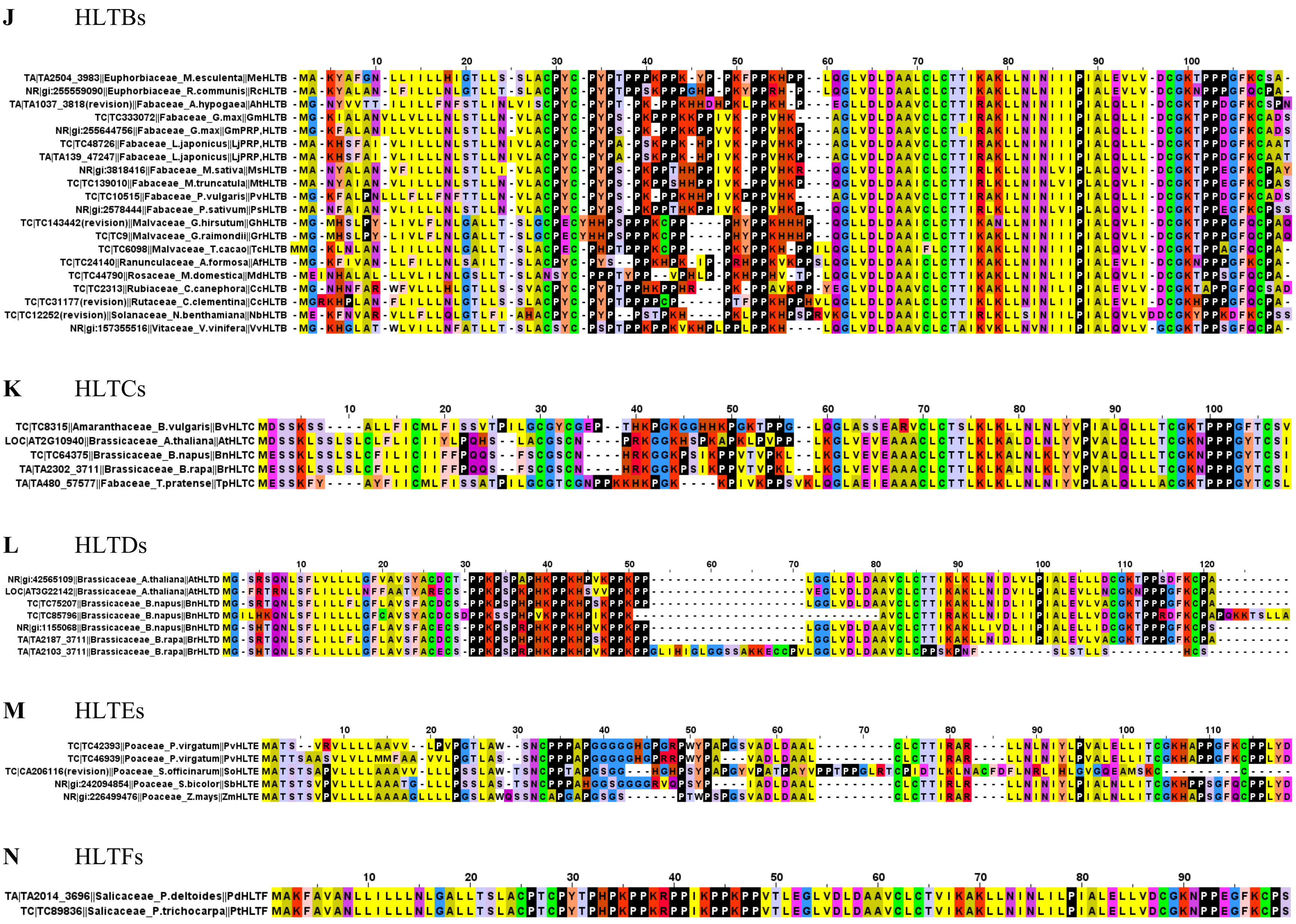
J HLTBs
K HLTCs
L HLTDs
M HLTEs
N HLTFs

O HPOAs

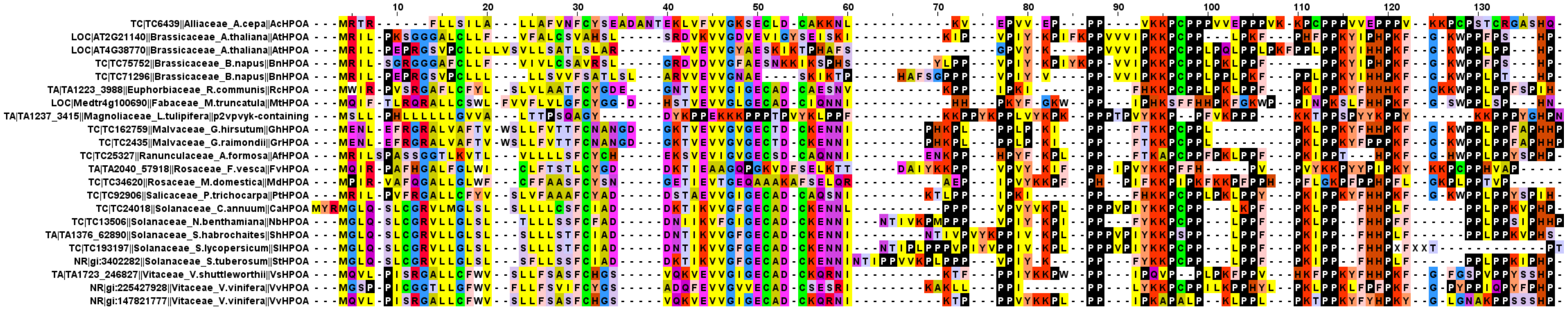

P HPOBs

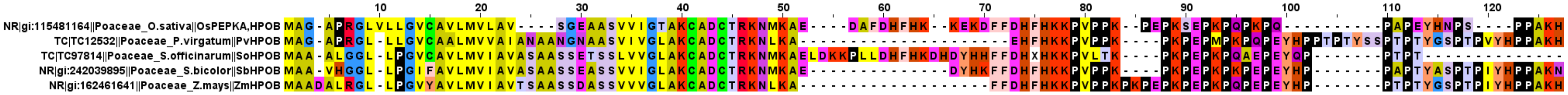

Q HPOCs

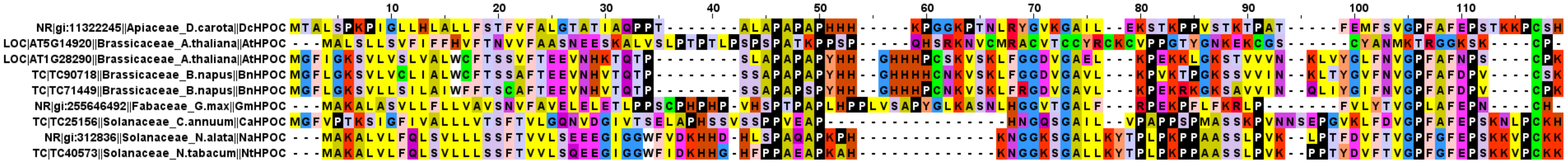

R KPIPs

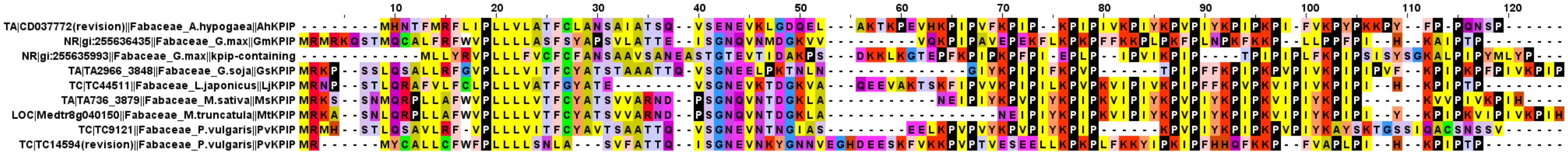

S MPAVs

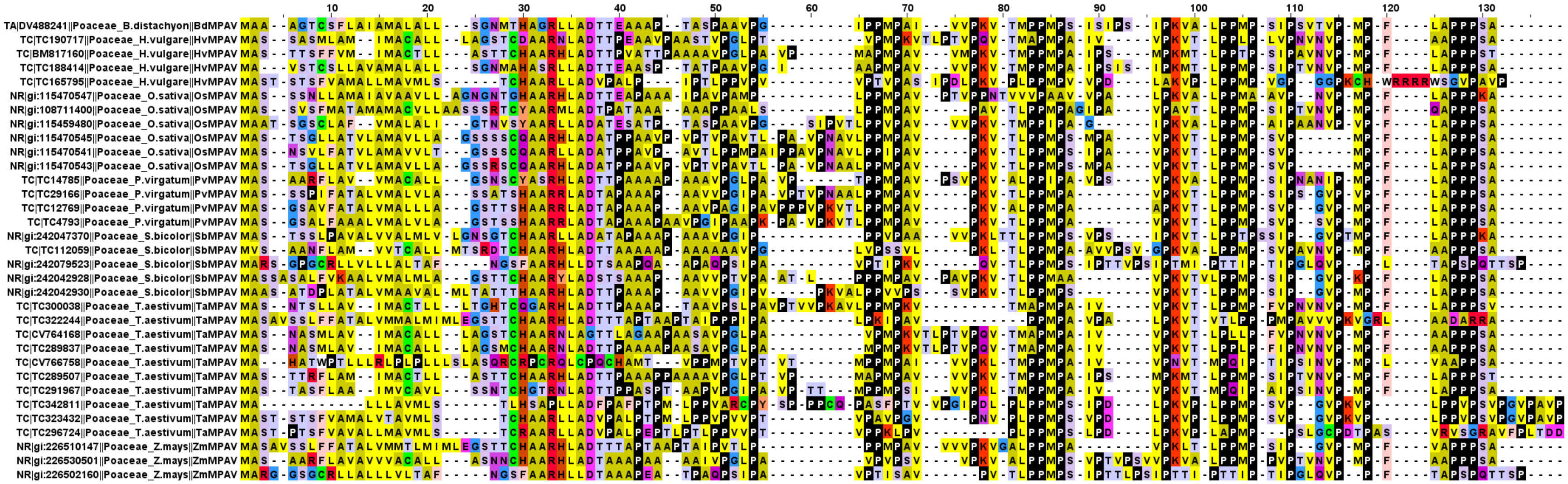

T PELPKs

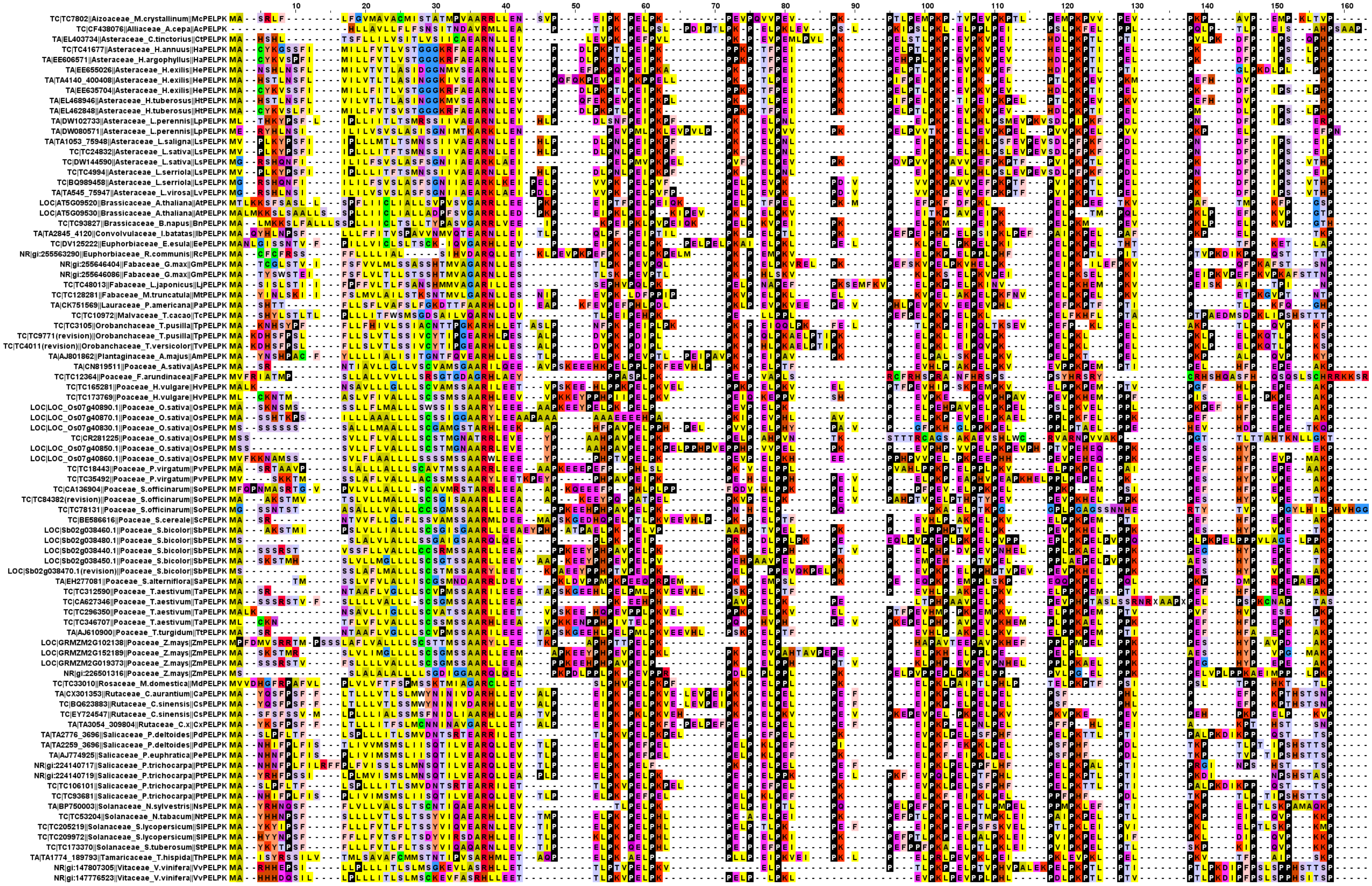

U PEPKAs

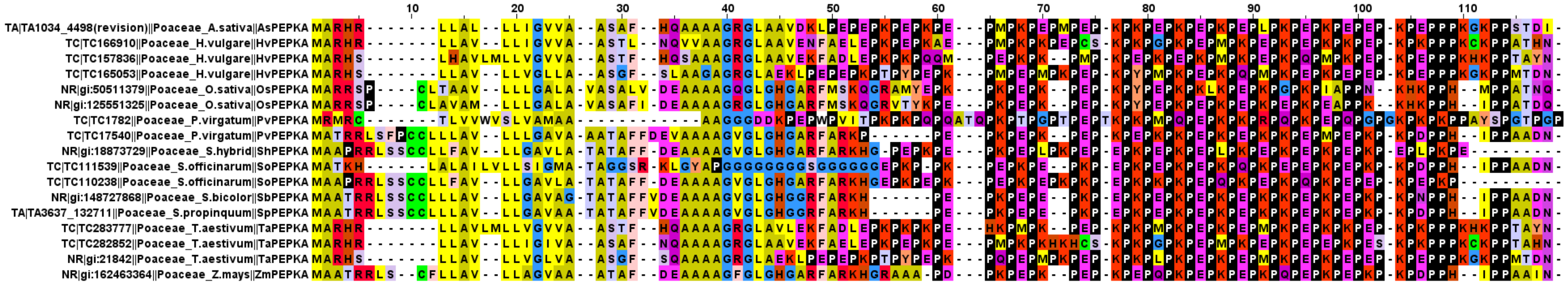

V PEPKBs

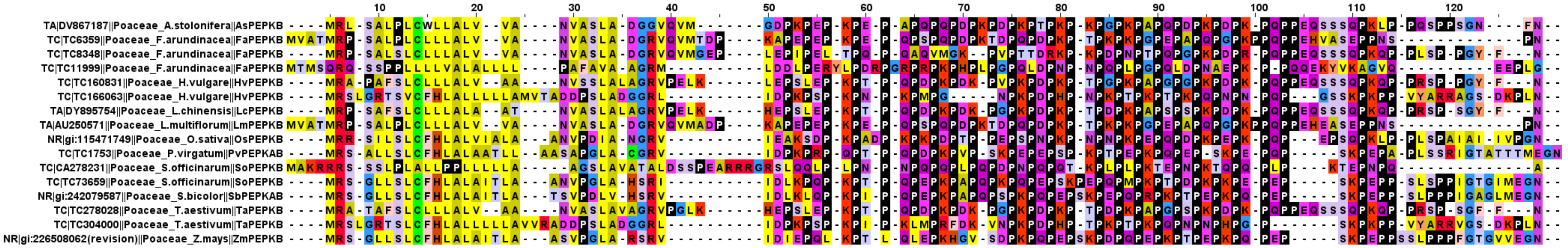

W **P**EPKCs

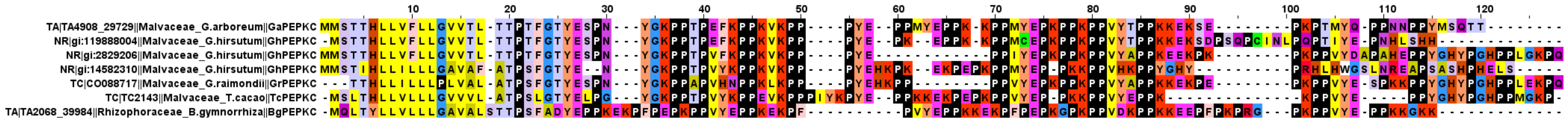

* *B.gymnorrhiza* PEPKC was predicted from a partial ORF lacking a stop codon.

X PRPAs

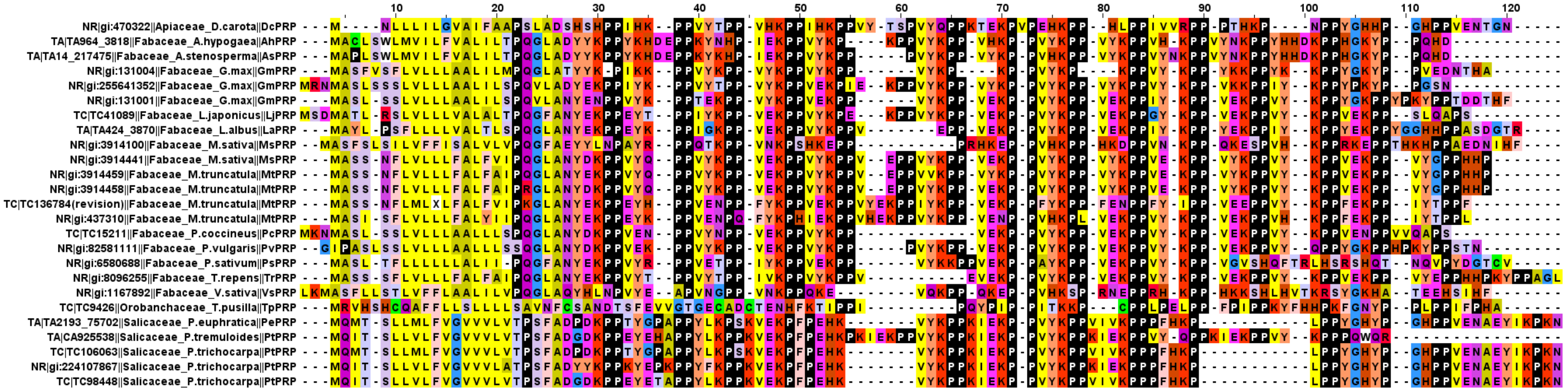

NR|gi:470322||Apiaceae_D.carota||DcPRP
TA|TA964_3818||Fabaceae_A.hypogaea||AhPRP
TA|TA14_217475||Fabaceae_A.stenosperma||AsPRP
NR|gi:131004||Fabaceae_G.max||GmPRP
NR|gi:255641352||Fabaceae_G.max||GmPRP
NR|gi:131001||Fabaceae_G.max||GmPRP
TC|TC41089||Fabaceae_L.japonicus||LjPRP
TA|TA424_3870||Fabaceae_L.albus||LaPRP
NR|gi:3914100||Fabaceae_M.sativa||MsPRP
NR|gi:3914441||Fabaceae_M.sativa||MsPRP
NR|gi:3914459||Fabaceae_M.truncatula||MtPRP
NR|gi:3914458||Fabaceae_M.truncatula||MtPRP
TC|TC136784(revision)||Fabaceae_M.truncatula||MtPRP
NR|gi:437310||Fabaceae_M.truncatula||MtPRP
TC|TC15211||Fabaceae_P.coccineus||PcPRP
NR|gi:82581111||Fabaceae_P.vulgaris||PvPRP
NR|gi:6580688||Fabaceae_P.sativum||PsPRP
NR|gi:8096255||Fabaceae_T.repens||TrPRP
NR|gi:1167892||Fabaceae_V.sativa||VsPRP
TC|TC9426||Orobanchaceae_T.pusilla||TpPRP
TA|TA2193_75702||Salicaceae_P.euphratica||PePRP
TA|CA925538||Salicaceae_P.tremuloides||PtPRP
TC|TC106063||Salicaceae_P.trichocarpa||PtPRP
NR|gi:224107867||Salicaceae_P.trichocarpa||PtPRP
TC|TC98448||Salicaceae_P.trichocarpa||PtPRP

Y QRAs

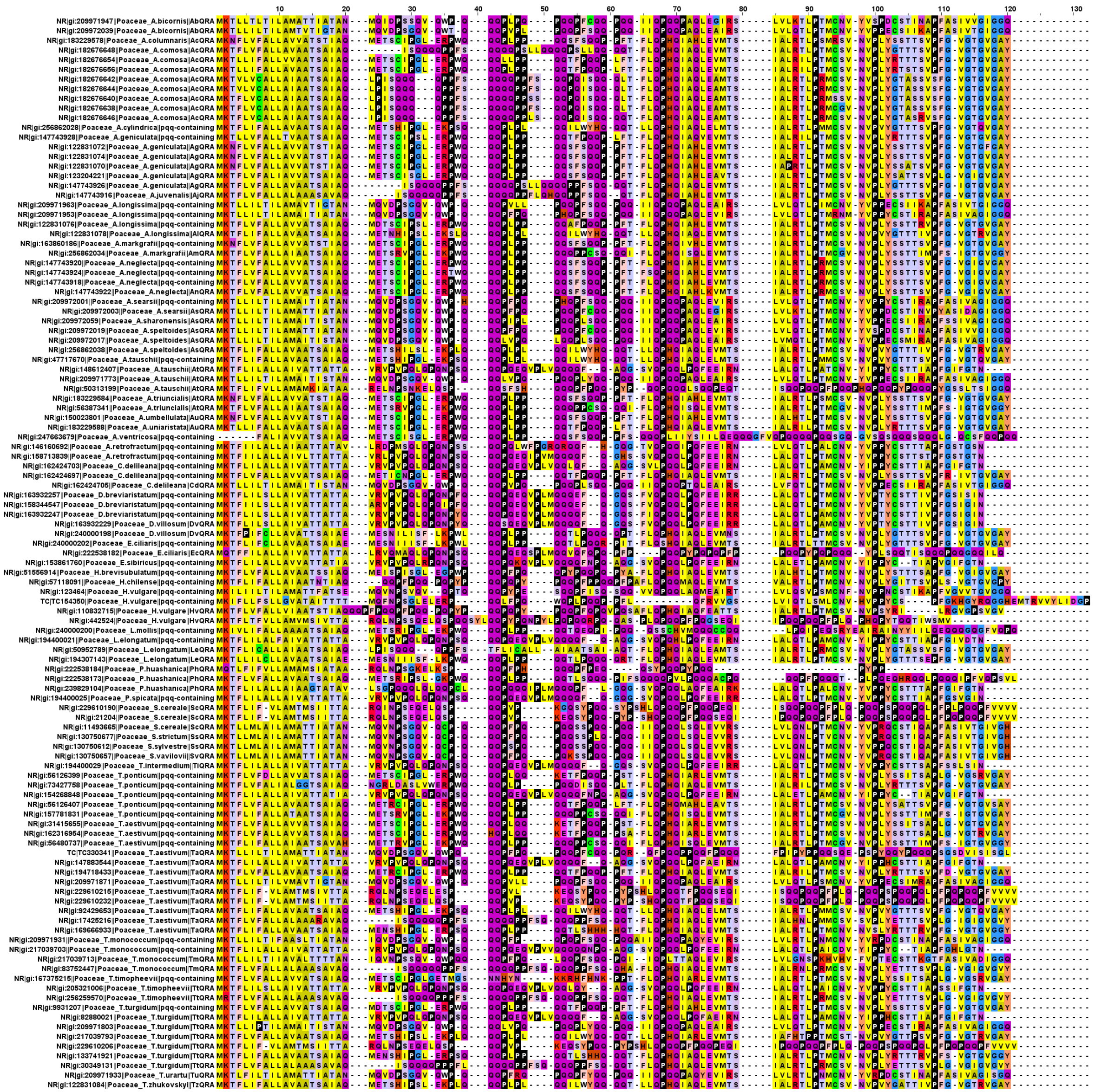

Z QRBs

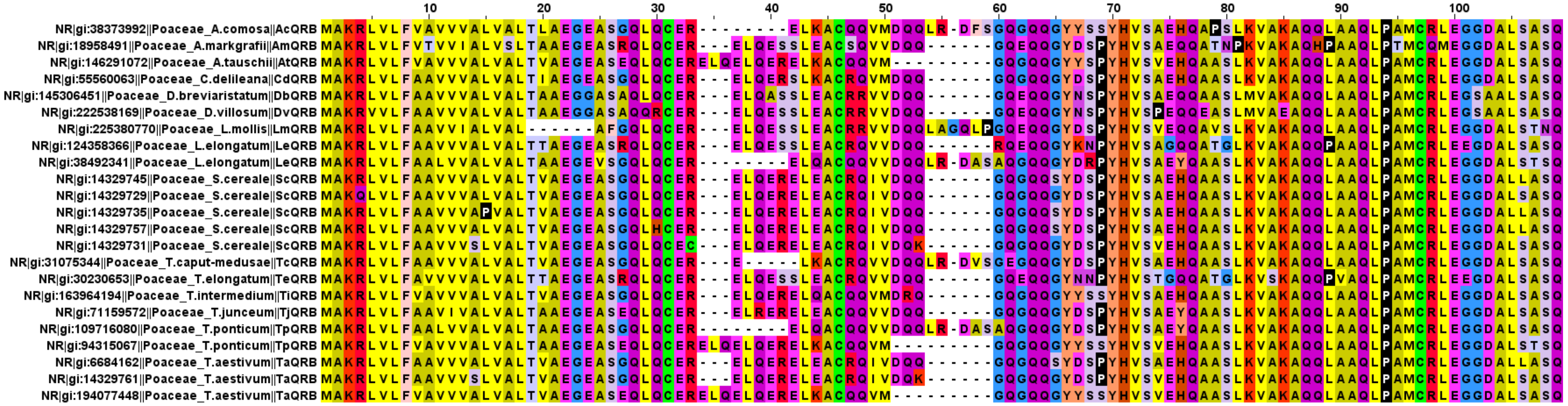
NR|gi:38373992||Poaceae_A.comosa||AcQRB
NR|gi:18958491||Poaceae_A.markgrafii||AmQRB
NR|gi:146291072||Poaceae_A.tauschii||AtQRB
NR|gi:55560063||Poaceae_C.delileana||CdQRB
NR|gi:145306451||Poaceae_D.breviaristatum||DbQRB
NR|gi:222538169||Poaceae_D.villosum||DvQRB
NR|gi:225380770||Poaceae_L.mollis||LmQRB
NR|gi:124358366||Poaceae_L.elongatum||LeQRB
NR|gi:38492341||Poaceae_L.elongatum||LeQRB
NR|gi:14329745||Poaceae_S.cereale||ScQRB
NR|gi:14329729||Poaceae_S.cereale||ScQRB
NR|gi:14329735||Poaceae_S.cereale||ScQRB
NR|gi:14329757||Poaceae_S.cereale||ScQRB
NR|gi:14329731||Poaceae_S.cereale||ScQRB
NR|gi:31075344||Poaceae_T.caput-medusae||TcQRB
NR|gi:30230653||Poaceae_T.elongatum||TeQRB
NR|gi:163964194||Poaceae_T.intermedium||TiQRB
NR|gi:71159572||Poaceae_T.junceum||TjQRB
NR|gi:109716080||Poaceae_T.ponticum||TpQRB
NR|gi:94315067||Poaceae_T.ponticum||TpQRB
NR|gi:6684162||Poaceae_T.aestivum||TaQRB
NR|gi:14329761||Poaceae_T.aestivum||TaQRB
NR|gi:194077448||Poaceae_T.aestivum||TaQRB
10
20
30
40
50
60
70
80
90
100
